# Genome-scale long noncoding RNA expression pattern in squamous cell lung cancer

**DOI:** 10.1038/srep11671

**Published:** 2015-07-10

**Authors:** Ying Wang, Chen-Yue Qian, Xiang-Ping Li, Yu Zhang, Hui He, Jing Wang, Juan Chen, Jia-Jia Cui, Rong Liu, Hui Zhou, Lin Xiao, Xiao-Jing Xu, Yi Zheng, Yi-Lan Fu, Zi-Yu Chen, Xiang Chen, Wei Zhang, Cheng-Cheng Ye, Hong-Hao Zhou, Ji-Ye Yin, Zhao-Qian Liu

**Affiliations:** 1 Department of Clinical Pharmacology, Xiangya Hospital, Central South University, Changsha 410008; P. R. China; Institute of Clinical Pharmacology, Central South University; Hunan Key Laboratory of Pharmacogenetics, Changsha 410078; P. R. China; 2 The Affiliated Cancer Hospital of XiangYa School of Medicine, Central South University, Changsha, Hunan 410014, P. R. China; 3 Medical college of Georgia, Georgia regents University.

## Abstract

In this study, we aimed to explore the long noncoding RNA expression pattern in squamous cell lung cancer (SQCC) on a genome-wide scale. Total RNAs were extracted from 16 lung SQCC patients’ normal and matched lung cancer tissues by Trizol reagent. The expression level of genome-wide scale lncRNA and mRNA was determined by microarray. qRT-PCR was used to validate the lncRNA expression level in 47 patients. Data analyses were performed using R and Bioconductor. A total of 2,748 up and 852 down regulated probes were identified to be significantly and differentially expressed in tumor tissues. The annotation result of their co-expressed mRNAs showed that the most significantly related category of GO analysis was development and differentiation, while the most significantly related pathway was cell cycle. Subgroup analysis identified that 46 and 18 probes were specifically differentially expressed in smoking and moderately differentiated tumors, respectively. Our study indicated that clusters of lncRNAs were significantly and differentially expressed in SQCC compared with normal tissues in the same subject. They may exert a significant role in lung cancer development and could be potential targets for future treatment of SQCC.

Lung cancer is the leading cause of cancer related death globally^1^. Non-small cell lung cancer (NSCLC) and small cell lung cancer (SCLC) are the two main forms, among newly diagnosed patients, approximately 85% are NSCLC^2^. Squamous cell carcinoma (SQCC) is one of the most common histological subtypes of NSCLC, and account for approximately 30% of all cases^3^. Although a large number of studies were conducted, the causes of lung cancer still remain unknown. It is now widely accepted that lung cancer is multifactorial and both genetic and environmental factors play pivotal roles in its occurrence.

Long non-coding RNAs (lncRNAs) are noncoding RNAs with more than 200 nucleotide in length^4^. Although without protein coding capability, lncRNAs play an important role in a number of biological processes, including tumor suppressors modulation, RNA-RNA interactions, epigenetic and post-transcription regulation^5^. It is also reported that lncRNAs played a role in carcinogenesis and could be diagnostic or prognostic biomarkers for lung cancer^5^. For example, metastasis-associated lung adenocarcinoma transcript 1 (MALAT1) was firstly identified in NSCLC and its expression was highly associated with NSCLC susceptibility, prognosis and metastasis^6^. Another lncRNA Hox transcript antisense intergenic RNA (HOTAIR) was also widely investigated, its expression was reported to be important in NSCLC development, prognosis and clinical relapse^7^,^8^. Some other reported NSCLC related lncRNAs includes growth arrest-specific transcript 5 (GAS5)^9^, Sox2 overlapping transcript (Sox2ot)^10^ and BRAF activated non-coding RNA (BANCR)^11^ *et al.* However, the knowledge of genome scale expression of lncRNAs and their potential biological function in SQCC is still lacking.

In the present study, we investigated the expression of lncRNAs in 16 squamous cell lung carcinoma tumors and matched adjacent non-tumor lung tissues (NTL) by microarray analysis. The results were further validated by quantitative reverse transcription-polymerase chain reaction (qRT-PCR) in another 31 paired tissues. Then, we identified lncRNAs that were significantly differentially expressed in lung SQCC tissues, and integrated mRNA expression data to predict the possible function of those lncRNAs. Finally, we conducted the subgroup according to patients’ age, smoking status, differentiation, complication and TNM stages, as well as an overlap analysis with two previously published data.

## Results

### Patients’ enrollment and lncRNA categorization

Our initial screening recruited a total of 262 patients’ tumor and matched NTL tissues, among which, 184 were other histological types of lung cancer than SQCC. Thus 78 paired samples were subjected to RNA extraction, however, half of the patients’ samples couldn’t be processed to further study because of low quality. At last, 47 paired samples were remained. The lncRNA and mRNA expression profile of 16 lung SQCC tumors and matched NTL tissues were detected using microarray, and remained 31 paired tissues were used as validation set. The overview of sample selection and analysis was indicated in Figure S1.

**Figure 1:**
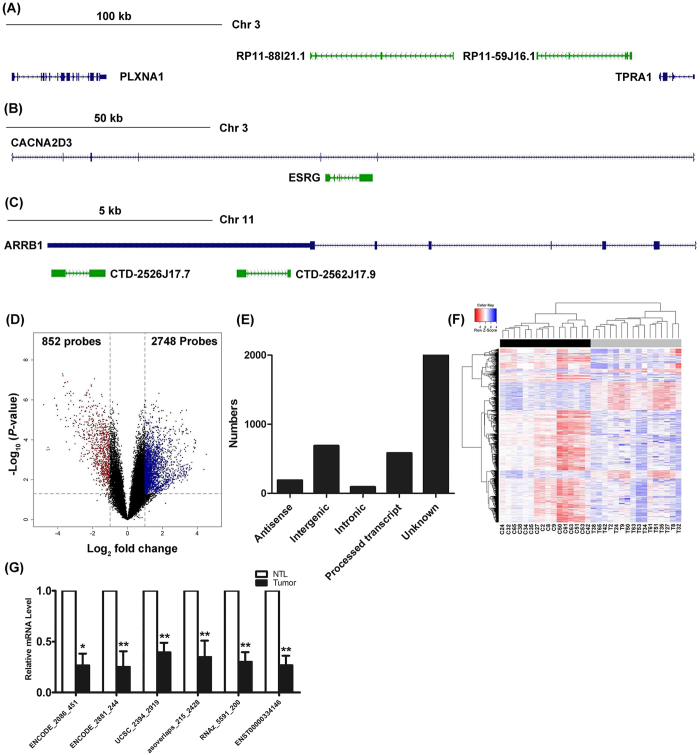
Identification of lncRNAs expression differences between lung SQCC tumors and NTL. (**A**–**C**) Schematic diagram of three different categories of lncRNA. (**A**) Intergenic lncRNA, RP11-88I21.1 and RP11-59J16.1, located between protein coding genes of PLXNA1 and TPRA1. (**B**) Intronic lncRNA, ESRG, located in intron of protein coding gene CACNA2D3. (**C**) Antisense lncRNAs, CTD-2526J17.7 and CTD-2562J17.9, overlaps protein coding gene ARRB1 on the opposite strand. (**D**) Volcano plot of the differential lncRNA expression analysis. X-axis: log_2_ fold change (median tumor-median NTL); Y-axis: −1 × log_10_ (FDR *p*-value) for each probes; Vertical dotted lines: fold change ≥2 or ≤2; Horizontal dotted line: the significance cutoff (FDR *p*-value = 0.05). (**E**) Distribution of lncRNAs differentially expressed in the lung SQCC tissue. (**F**) Two-dimensional hierarchical clustering of the significant and differentially expressed lncRNA probes in all samples (16 tumor tissues in gray vs. 16 NTL tissues in black). Probes are in rows; samples are in columns. Both up-regulated and down-regulated lncRNAs can be seen in tumors compared with normal tissues. (**G**) The expression of 6 randomly selected differentially expressed lncRNAs were validated by quantitative real-time PCR in all 47 patients. * p < 0.05, **p < 0.001 compared with NTL tissues.

In this study, we determined the expression of a total of 32,781 lncRNA probes. Based on Derrien *et al*’s categorization of lncRNAs in the GENCODE gene annotation^12^, we classified all tested lncRNAs into 5 groups: antisense, intergenic, intronic, processed transcript and unknown. The classification criteria was based on the lncRNA location with respect to protein-coding genes, 3 of them were exemplified in Fig. 1A–C.

### Identification of distinctive lncRNA expressions between lung SQCC tumors and matched NTL

The locus-by-locus lncRNA probes of tumors vs. NTL tissues were firstly analyzed. By the criteria of corrected *p*-value < 0.05 and absolute fold change >2, we identified 2,748 up regulated and 852 down regulated probes that were significantly and differentially expressed in tumor tissues compared with NTL (Table S1, Fig. 1D). We also analyzed the distinctive lncRNAs based on their categorizations, the results were indicated in Fig. 1E. Although more than half of the lncRNAs (56.89%) were not categorized, among the successfully annotated lncRNAs, more intergenic (19.11%) and processed transcript (16.17%) than antisense (5.25%) and intronic (2.58%) were differentially expressed. This pattern was also existed in the both up and down regulated lncRNAs (Figure S2), except that the processed transcript was more than intergenic in the down regulated lncRNAs.

Next, the two-dimensional (2D) hierarchical clustering of the 3600 lncRNAs was explored between lung SQCC tumors and NTL tissues (Fig. 1F). The separate clusters between tumors and NTL tissues indicated that these lncRNAs expression were substantially different between tumor and NTL tissues. We also constructed the heatmaps of these lncRNAs according to their categorizations. As indicated in Figure S3, separate clusters between tumors and NTL tissues were also observed for antisense, intronic, processed transcript and unknown lncRNAs. However, for intergenic lncRNAs, 7 NTL tissues were classified into the same cluster of tumor tissues.

Finally, to ensure that our results was not population and technical specific, we assessed the expression level of 6 lncRNAs using qRT-PCR in all 47 paired lung SQCC tumors and NTL tissues. These lncRNAs were randomly chosen from differentially expressed lncRNA transcripts. In consistent with microarray analysis, the results demonstrated that all the 6 lncRNAs were down-regulated in lung SQCC tissues vs. matched non-tumor tissues (Fig. 1G).

### Identification of potentially functionally lncRNAs in lung SQCC

**Figure 2:**
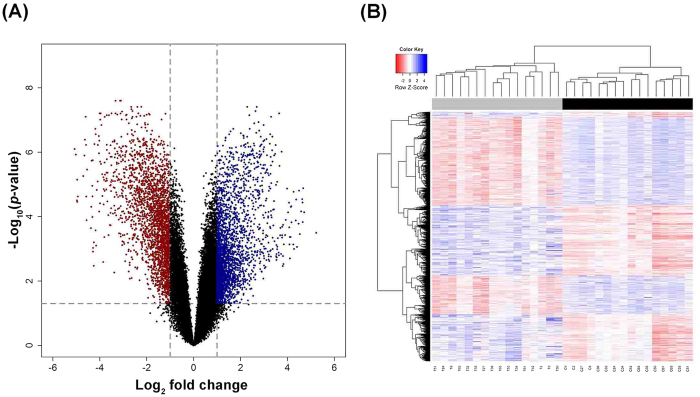
Significantly differentially expressed mRNAs in lung SQCC. (**A**) Volcano plot of the differential mRNA expression analysis. X-axis: log_2_ fold change (median tumor-median NTL); Y-axis: −1 × log_10_ (FDR *p*-value) for each probes; Vertical dotted lines: fold change ≥2 or ≤2; Horizontal dotted line: the significance cutoff (FDR *p*-value = 0.05). (**B**) Two-dimensional hierarchical clustering of the significant and differentially expressed mRNA probes in all samples (16 tumor tissues in gray vs. 16 NTL tissues in black). Probes are in rows; samples are in columns.

For these 3,600 differentially expressed lncRNA probes, the function of most of them remains unknown. Therefore, we predicted their potential function via the annotation of their co-expressed mRNAs. Firstly, the genome wide mRNA expression profile of these 16 lung SQCC and matched NTL tissues were detected. Also, The criteria of corrected *p*-value < 0.05 and absolute fold change >2 were used to identify significantly and differentially expressed mRNAs. Among the 30,201 detected probes, a total of 5,253 probes were found to be significantly differentially expressed (Fig. 2A, Table S2). 2,593 probes were up regulated, and 2,660 probes were down regulated. These differentially expressed probes were used to generate a heatmap, as indicated in Fig. 2B, they were clearly segregated into tumor and NTL clusters. This result suggested that these mRNAs were substantially different between tumor and NTL tissues.

**Figure 3:**
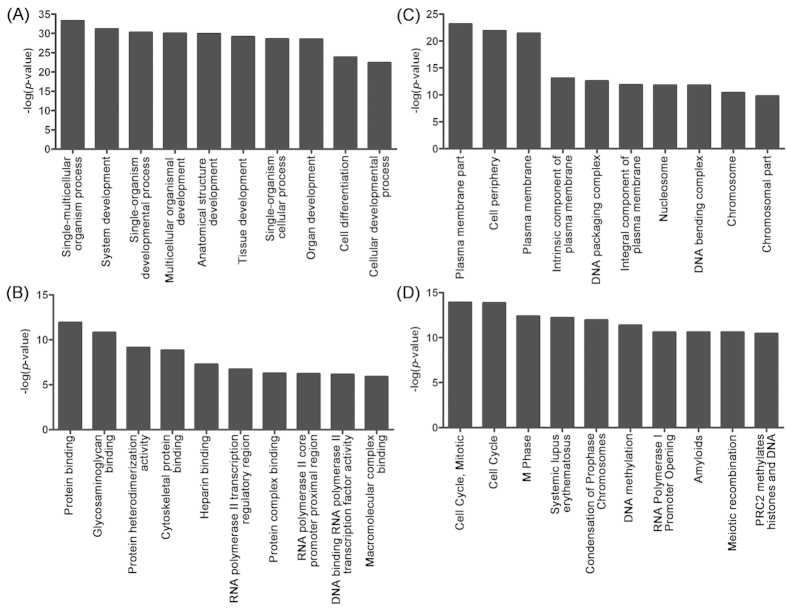
GO and pathway analysis of lncRNA co-expressed mRNAs. The top ten significantly enriched GO categories and pathways were calculated and plotted as the −1 × log_10_ (*p*-value). (**A**) Biological process, (**B**) Molecular function, (**C**) Cellular component, (**D**) Pathways.

We next investigated the correlation of differentially expressed lncRNAs with each dysregulated mRNAs. In order to predict the potential function of lncRNAs, their coexpressed mRNAs were subjected to GO annotation. In terms of the biological processes, most of the significantly related categories pointed to development and differentiation (Fig. 3A). 6 of the top 10 categories of molecular function were related to protein binding (Fig. 3B). With regards to cellular components, plasma membrane played most important role, indicated by its appearance in 4 most enriched categories (Fig. 3C). Then, the ConsensusPathDB database was used to identify which gene networks were affected by the aberrantly expressed lncRNAs, the top 10 significant pathways were indicated in Fig. 3D. It is interesting to note that top 3 pathways were all associated with cell cycle, suggesting that this pathway might be regulated by the aberrantly expressed lncRNAs in the NSCLC cancer tissues.

**Figure 4:**
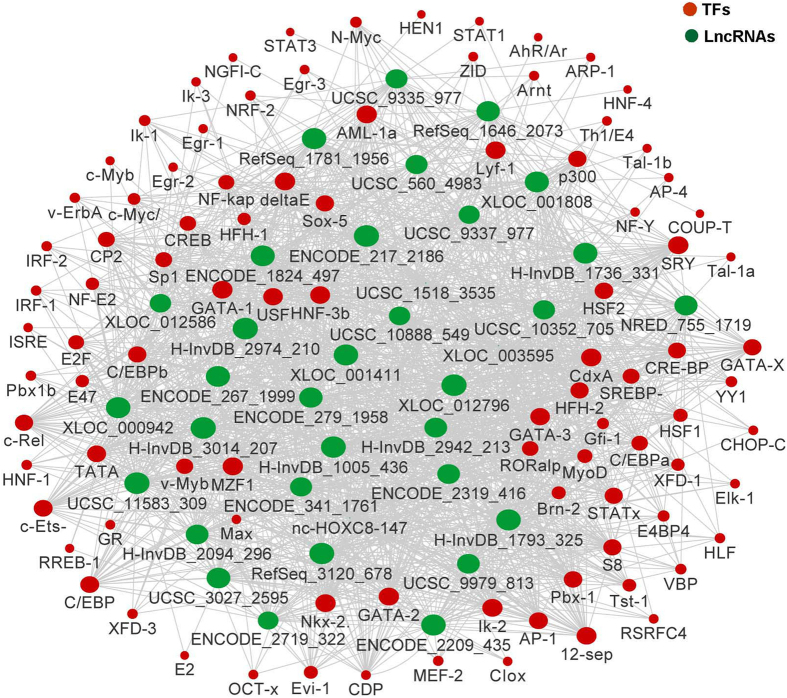
LncRNA-TFs network analysis. LncRNA-TFs network consist of 35 top correlated lncRNAs (PCC >0.95) and 95 their regulating TFs, they were connected by 1566 edges.

Previously, Guttman *et al.* indicated that some lncRNAs were transcriptionally regulated by transcription factors (TFs)^13^, we thus further predicted the TFs that could regulate these lncRNAs. The lncRNAs with PCC >0.95 were included in the analysis, as a result, a total of 35 lncRNAs were predicted to be regulated by 95 TFs. As indicated in Fig. 4, these lncRNAs were mostly regulated by 5 TFs: CdxA, AML-1a (RUNX1), GATA-2, GATA-1 and MZF1. We further did a literature search about these TFs, and found that AML-1a (RUNX1), GATA-2 and MZF1 were demonstrated to be correlated with lung cancer previously^14^,^15^,^16^.

### Subgroup and overlap analysis of lncRNA expressions

**Figure 5:**
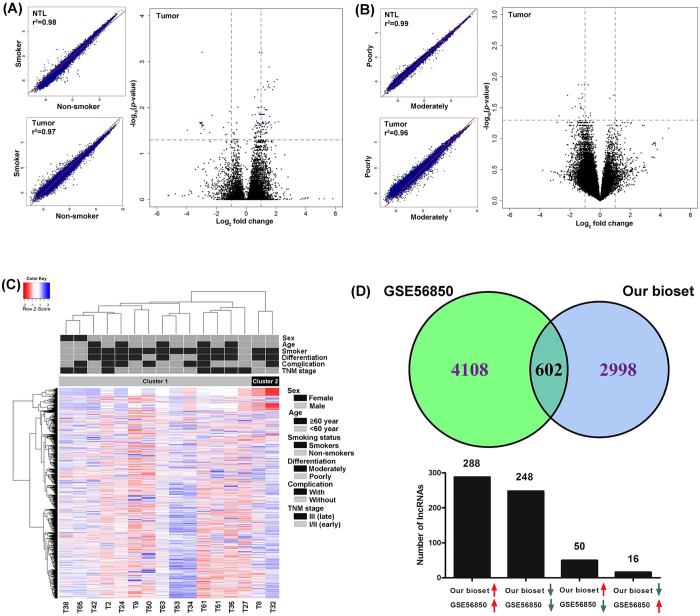
Subgroup and overlap analysis of lncRNA expressions. (**A**) and (**B**) Subgroup analysis of lncRNA expressions according to smoking status (**A**) and differentiation (**B**). Firstly, LncRNAs expression scatterplots of two different subgroups were drew according to the smoking status (smokers and non-smokers) and differentiation (low and moderate) in both tumor and NTL tissues. The correlation coefficient was given in the left corner. Then, volcano plot of the differential lncRNAs expression between subgroups based on smoking status and differentiation in tumor tissues were given. Vertical dotted lines: 2 fold changes in expression level; Horizontal dotted line: the significance cutoff. (**C**) Two-dimensional hierarchical clustering of the significantly different expressed lncRNA probes in tumors were performed (n = 16), two distinct clusters were identified. Probes are in rows; samples are in columns. Clinical characteristics were shown on the right, two clusters were indicated at the top of the heatmap. (**D**) Venn diagram shows the overlap of significantly expressed lncRNAs in our study versus the dataset of GSE56850. A total of 602 probes were found and their expression direction in two datasets was also indicated below. Red arrow: up regulation. Green arrow: down regulation.

To further verify the correlation of various lncRNA expression and lung SQCC, we next conducted subgroup analysis. The patients were firstly grouped according to their sex (male and female), age (<60 and ≥60 years old), smoking status (smokers and non-smokers), differentiation (low and moderate), complication (with and without) and TNM stages (I/II and III). Then, the correlation study was performed for each paired subgroups in both tumor and control tissues. As indicated in Fig. 5 and S4, very similar lncRNA expression profiles were observed in all paired subgroups. To further identify differential expressed lncRNA probes, we next conducted a locus-by-locus differential lncRNA expression analysis for these paired subgroups in both tumor and control tissues. Based on the criteria of corrected *p*-value < 0.05 and absolute fold change >2, the differentially expressed lncRNAs were only observed in different smoking status and differentiation patients. As indicated in Fig. 5A,B and Table S3, compared with non-smokers, smokers showed 23 down regulated and 23 up regulated probes in tumor tissues. While normal tissues showed no significantly expressed lncRNA between smokers and non-smokers, indicated that these lncRNAs were specifically correlated with smoking for cancers. It is interesting to note that most of the down regulated probes pointed to H19 (imprinted maternally expressed transcript), which was previously reported to be a tumor suppressor and its methylation status correlated with smoking^17^. On the other hand, compared with poor differentiation subgroups, 18 differentially expressed lncRNAs probes were observed in moderately differentiated tumors. Also, normal tissues showed no significantly expressed lncRNA between different differentiation subgroups, suggesting that these lncRNAs were specifically associated with tumor tissue differentiation. All of the 18 lncRNA probes were down regulated, however, most of them were not annotated.

Next, to identify potential lncRNA expression-based subclasses of the 16 tumors, we performed a hierarchical clustering analysis using the 3600 significantly deregulated lncRNA probes. As showed in Fig. 5C, two distinct clusters were indicated (14 vs. 2). To investigate the relationship between these two clusters and their clinical properties, we conducted the statistical analysis for sex, age, smoking status, differentiation, complication and TNM stage. However, no significant difference was observed between two clusters for these clinical characteristics.

To compare our study with other studies, we did a literature search in the GEO database, only 1 dataset (GSE56850) of lung cancer lncRNA expression was uploaded using the same microarray platform of ours^18^. We thus did an overlap analysis between these two studies. It was noteworthy that GSE56850 dataset was conducted in the adenocarcinoma. As indicated in Fig. 5D, a total of 602 lncRNA probes were significantly differentially expressed in both studies, most of the probes were expressed at the same direction. However, 50 up regulated probes were down regulated in the GSE56850 dataset, and 16 down regulated probes were up regulated in GSE56850 dataset. This result indicated that some lncRNAs may play different roles in different pathological types of NSCLC. The detail information of these 602 probes was listed in Table S4.

**Figure 6:**
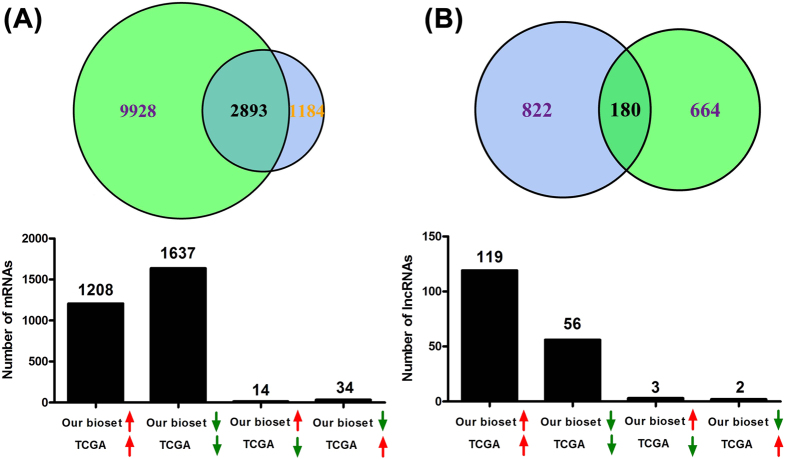
Overlap analysis between our study and TCGA data. Venn diagram shows the overlap of significantly expressed mRNAs (**A**) and lncRNAs (**B**) in our study versus the dataset of TCGA. A total of 2893 overlapped mRNAs and 180 overlapped lncRNAs were found and their expression direction in two datasets was also indicated below. Red arrow: up regulation. Green arrow: down regulation.

Previously, TCGA generated a large number of RNA-seq data of lung SQCC compared with non-tumor tissues^19^. To learn about the overlap between our microarray and TCGA RNA-seq results, we compared current study’s differentially expressed mRNAs and lncRNAs with their results. As showed in Fig. 6A and Table S5, TCGA data identified more differentially expressed mRNAs than ours, two studies owned an overlap of 2893 mRNAs, among which 98.34% probes were expressed at the same direction. Similar to this result, we also identified less significantly differentially expressed lncRNAs compared with TCGA data (Fig. 6B and Table S6). 180 lncRNAs were overlapped between two investigations, and only 5 lncRNAs had different expression direction. Taken together, these results suggested that TCGA RNA-seq experiment identified more differentially expressed mRNAs and lncRNAs than ours, however, two studies had high consistency for the overlapped probes.

## Discussion

In the present study, we investigated the genome-wide expression profile of lncRNAs in 16 squamous cell lung carcinoma tumors and matched adjacent NTL by microarray. A total of 2,748 up and 852 down regulated probes were identified to be significantly and differentially expressed in tumor tissues. The annotation result of their co-expressed mRNAs showed that the most significantly related category of GO analysis was development and differentiation, while the most correlated pathway were cell cycle. Co-expression network construct indicated that some of the mostly connected mRNAs and TFs were previously reported to be dysregulated in lung cancer, including SOX2, FOXF1, PTK2B, XRCC2, AML-1a (RUNX1), GATA-2 and MZF1. Our next subgroup analysis identified that 46 and 18 probes were specifically differentially expressed in smoking and moderately differentiated tumors, respectively. Finally, we conducted an overlap analysis with another adenocarcinoma study and TCGA RNA-seq results.

LncRNAs were emerging as a novel class of gene regulators to the cancers^5^. The expression pattern of lncRNAs in some certain types of cancers was investigated, including NSCLC^20^. NSCLC includes two major pathological types: adenocarcinoma and SQCC. However, the genome-scale lncRNA expression profile of lung SQCC was yet to be explored. In this study, we identified 3600 lncRNA probes showing significant differential expression in lung SQCC tissues. They can substantially distinguish lung SQCC and NTL tissues. Some certain dysregulated lncRNAs that were found in our study were also reported in previous other investigations, which supported the result for each other. One of the previously reported up regulated lncRNA was Sox2ot. In our study, 17 probes were designed to detect this lncRNA and 12 of them were up regulated, the average increase fold was 5.98. It was also noteworthy that mRNA Sox2 was increased 11.77 fold in the tumor tissues. Furthermore, based on our co-expression analysis, Sox2 was correlated with 9 Sox2ot probes. Our result showed that Sox2ot may regulate Sox2, and *vice versa*. Some previous studies also explored this possibility, however, the detail mechanism still remains unknown^10^,^21^. Another two examples were HOTAIR and MEG3. HOTAIR was widely reported as an oncogene, its expression enhanced NSCLC cells aggressive behavior and related to lymphatic invasion and relapse in SCLC patients^7^,^8^ . In agreement with these reports, our result also showed 2.35 fold increased expression of this lncRNA in SQCC tissues. MEG3 was reported as a tumor suppressor, its expression was decreased in NSCLC tumor tissues and associated with poor prognosis^22^. Our result also showed 2.71 fold decreasing of MEG3 in tumor tissues. However, most of the differentially expressed lncRNAs identified in this study were novel and need further study.

Although some lncRNAs are identified to play key roles in many biological processes such as cell differentiation, immune responses and tumorigenesis, most of their functions are not well understood^23^. One approach for the functional prediction of lncRNAs was construct coding-non-coding gene co-expression network^24^. This method was also used to predict the function of significantly dysregulated lncRNAs in the current study. In the present study, some of the mostly connected mRNAs were already reported to be correlated with lung cancer, including SOX2, FOXF1, PTK2B and VHL. We thus proposed that their predicted regulating lncRNAs may be correlated with lung cancer carcinogenesis through regulating these genes. Another network constructed in the current study was lncRNA-TFs network, AML-1a (RUNX1), GATA-2 and MZF1 were among the mostly correlated TFs. Previous investigation showed that they were correlated with lung cancer, however, the mechanisms were still unclear. We thus speculated that these TFs may participate in lung cancer carcinogenesis through regulating the lncRNAs predicted in this study. However, these two hypothesis needs to be further investigated.

In this study, we identified 46 and 18 significantly differentially expressed lncRNAs correlated with smoking and tumor differentiation, respectively. One of them was H19, which was strongly down regulated in smokers’ cancer tissues. This lncRNA was widely reported to be correlated with smoking, our result supported their investigation^25^. Except for H19, most of the other lncRNSs were not reported previously. However, these lncRNAs may be helpful to understand lung SQCC differentiation, as well as and the role of smoking in the lung cancer carcinogenesis. By performing hierarchical clustering analysis on tumors, we identified two distinct clusters (cluster1 vs. cluster2: 14 vs. 2). However, we didn’t further explore the different lncRNAs expression profiles between these two clusters, because of the too small sample size of cluster 2. This investigation need to be performed in a larger sample size in the future study to see if subtypes could be discovered in the lung SQCC. We also compared the dysregulated lncRNA probes in our investigation to those in a previous study conducted in adenocarcinoma. Although some overlaps existed between two studies, attention should be paid to the lncRNAs that were differently expressed in two datasets. These subtype specific lncRNAs were specifically dysregulated in one dataset or dysregulated to opposite direction in both datasets. Based on our analysis, most of the significantly differentially expressed lncRNAs were subtype specific, indicating that they play different role in SQCC and adenocarcinoma of lung. The function of these lncRNAs awaits further studies.

To further explore the overlap between microarray and RNA-seq results, we compared our findings with TCGA lung SQCC data. The comparison suggested that RNA-seq identified more significantly differentially expressed probes (both mRNA and lncRNA) than our microarray assay. For the overlapped probes, two studies had high consistency with each other. This result is in consistent with previous other investigations^26^,^27^, indicating that RNA-seq is a more sensitive and highly replicable assay compared with microarray.

Taken these together, we identified a panel of dysregulated lncRNAs that may be potential biomarkers and correlated with carcinogenesis of lung SQCC. They may be lung SQCC specific lncRNAs and thus indicate for potential changes to diagnosis or therapy. We are conducting *in vitro* functional studies for some of these lncRNAs to further explore their detail functions. In conclusion, this study lays a foundation for further diagnostic, therapeutic and functional research of lung SQCC lncRNAs .

## Materials and Methods

### Study samples

This project’s protocol was approved by the Ethics Committee of Xiangya School of Medicine, Central South University with registration number of CTXY-110008-3. All the patients were provided written informed consents in compliance with the code of ethics of the World Medical Association (Declaration of Helsinki) at the time of surgery for the donation of their tissue for this research. We obtained the clinical research admission on the Chinese Clinical Trial Registry (http://www.chictr.org/cn/) and the registration number is ChiCTR-RCC-12002830. All of the tissues were histologically confirmed and enrolled between September 2011 and September 2013 at Xiangya Hospital of Central South University and Hunan Province Tumor Hospital (Changsha, Hunan, China). All fresh tissues were frozen in liquid nitrogen immediately after resection and stored in −80 °C until use. Their basic clinical characteristics including age, smoking status, histology, sex, differentiation, complication and TNM stage were collected (Table 1).

### Array data production

The experiment was performed in the laboratory of CapitalBio Corporation (Beijing, China). In brief, total RNA was first extracted using Trizol reagent (Invitrogen, Carlsbad, California, USA) and then purified using the NucleoSpin® RNA clean-up Kit (Macherey-Nagel, Düren, Germany). Extracted RNA was quantified by using NanoDrop ND-1000 (Thermo Scientific, Wilmington, Delaware, USA) spectrophotometer and the integrity was evaluated by 1% formaldehyde denaturing gel electrophoresis. 1 g total RNA was used to synthesize double-stranded cDNAs by *in vitro* transcription. The cDNA was labeled by Cy3-dCTP or Cy5-dCTP (GE Healthcare, Piscataway, New Jersey, USA) and hybridized to the Human 4 × 180 K expression microarray (Agilent Technologies, Santa Clara, California, USA). The data was extracted by Agilent Feature Extraction (v10.7) and was summarized, normalized and quality controlled using GeneSpring GX program (v11.5).

### qRT-PCR

qRT-PCR was used to validate the lncRNA expression level as described previously^28^,^29^. In brief, the total RNA was isolated using Trizol reagent (Invitrogen) and then reverse transcribed to cDNA using PrimeScript^TM^ RT-PCR Kit (Takara, Dalian, China) in accordance to the manufacturer’s protocol. Real-time PCR was performed using SYBR® Premix DimerEraser™ (Perfect Real Time) (Takara, Dalian, China) in Roche LightCycler 480 II Real-Time PCR system (Roche Diagnostics Ltd., Rotkreuz, Switzerland). The threshold cycle value (Ct) of each product was determined and normalized against that of the internal control of GAPDH. The differences of lncRNAs expression level were compared by t test using SPSS 13.0 (SPSS Inc, Chicago, Illinois, USA). *P* < 0.05 was considered as statistically significant.

### Data analysis

All data analyses were performed using R (http://www.r-project.org/, version 2.15.3) and Bioconductor^30^.

#### Differential expression analysis

Differential expression analysis was assessed using t-test, and the *p*-values were corrected for False Discovery Rate (FDR) by Benjamini-Hochberg (BH) procedure. Statistical significance was defined as FDR *p*-value < 0.05. In addition, a two fold change of expression level between comparison of tumors and NTL was defined as significantly different lncRNAs or mRNAs. The heat map, locus-by-locus volcano plot, venn diagram and scatterplot of significant differentially expressed lncRNA were performed by gplots, lattice, venndiagram and ggplot2 packages in R, respectively.

#### Functional classification and pathway analyses

The Gene Ontology (GO) and pathway analysis was conducted by ConsensusPathDB database (http://consensuspathdb.org/). The GO analysis was divided into molecular function, biological process and cellular component. Also, a corrected *p*-value < 0.05 was the threshold for statistically significant correlation.

#### Co-expression network analysis and overlap analysis

To identify the co-expression lncRNA-mRNA pairs, we calculated the Pearson correlation coefficients (PCC) of each dysregulated lncRNA and mRNA probe. The PCC <−0.8 or >0.8 and corrected *p*-value < 0.05 was considered as statistically significant. In addition, we predicted the potential lncRNA regulating transcription factors (TFs) using TFSearch (http://www.cbrc.jp/research/db/TFSEARCH.html). The searching areas include 2 kb upstream and 500 bp downstream of each lncRNA gene loci. Finally, the lncRNA-mRNA and lncRNA-TFs network was drawn by Cytoscape (v3.1.1).

The overlap analyzed dataset of GSE56580 and TCGA were downloaded from gene expression omnibus (GEO) database (http://www.ncbi.nlm.nih.gov/geo/) and lncRNAtor (http://lncrnator.ewha.ac.kr/index.htm)^31^, respectively.

## Additional Information

**How to cite this article**: Wang, Y. *et al.* Genome-scale long noncoding RNA expression pattern in squamous cell lung cancer. *Sci. Rep.* **5**, 11671; doi: 10.1038/srep11671 (2015).

## Supplementary Material

Supplementary Information

## Figures and Tables

**Table 1:** Clinical and phenotypical characteristics of 16 lung SQCC patients.

Patient No.	Sex	Age at Diagnosis	Smoking status (pack-years value)	Differentiation	Location	Complication	TNM stage
1	Male	58	35	Moderately	Right, upper	Hypertension	IIIA
2	Male	59	40	Moderately	Left, upper	No	IB
3	Male	48	60	Moderately	Right, upper	No	IIB
4	Male	71	5	Moderately	Left, upper	Coronary heart disease	IB
5	Male	59	No	Poorly	Right, lower	No	IIIA
6	Male	57	115	Moderately	Left, lower	Hypertension	IIB
7	Male	53	30	Poorly	Right, lower	Chronic bronchitis	IIB
8	Male	65	80	Moderately	Left	No	IIIA
9	Female	47	No	Poorly	Left, lower	No	IIIA
10	Male	64	5	Moderately	Right, upper	No	IIB
11	Male	57	30	Poorly	Left, upper	Osteoarthritis	IB
12	Male	50	30	Moderately	Left, upper	No	IIIA
13	Male	49	45	Poorly	Left, lower	No	IB
14	Male	65	15	Moderately	Left, upper	Hypertension	IIIA
15	Male	68	30	Moderately	Right, upper	No	IB
16	Female	44	No	Poorly	Left, lower	Cholecystitis/Chronic epatitis C infection	IIIA
