# Supplementary material for: Genome-scale long noncoding RNA expression pattern in squamous cell lung cancer
Source: Sci Rep. 2015 Jul 10;5:11671. doi: 10.1038/srep11671 (PMC4498179; doi:10.1038/srep11671)
Supplement: Supplementary Information [file srep11671-s1.pdf]

Genome-scale long noncoding RNA expression pattern in squamous cell lung cancer

Ying Wang[1,2#], Chen-Yue Qian[1#], Xiang-Ping Li[1], Yu Zhang[1], Hui He[1], Jing Wang[2], Juan Chen[1], Jia-Jia Cui[1], Rong Liu[1], Hui Zhou[2], Lin Xiao[1], Xiao-Jing Xu[3], Yi Zheng[1], Yi-Lan Fu[1,2], Zi-Yu Chen[1,2], Xiang Chen[1], Wei Zhang[1], Cheng-Cheng Ye[3], Hong-Hao Zhou[1], Ji-Ye Yin[1]*, Zhao-Qian Liu[1]*

[1]Department of Clinical Pharmacology, Xiangya Hospital, Central South University, Changsha 410008; P. R. China; Institute of Clinical Pharmacology, Central South University; Hunan Key Laboratory of Pharmacogenetics, Changsha 410078; P. R. China; [2]The Affiliated Cancer Hospital of XiangYa School of Medicine, Central South University, Changsha, Hunan 410014, P. R. China. [3]Medical college of Georgia, Georgia regents University.

*To whom correspondence should be addressed: Professor Zhao-Qian Liu, Department of Clinical Pharmacology, Xiangya Hospital, Central South University, Changsha 410008; P. R. China; Institute of Clinical Pharmacology, Hunan Key Laboratory of Pharmacogenetics, Central South University, Changsha, Hunan 410078, P. R. China Tel: +86 731 84805380, Fax: +86 731 82354476, E-mail: liuzhaoqian63@126.com

Or Professor Ji-Ye Yin, Department of Clinical Pharmacology, Xiangya Hospital, Central South University, Changsha 410008; P. R. China; Institute of Clinical Pharmacology, Hunan Key Laboratory of Pharmacogenetics, Central South University, Changsha, Hunan 410078, P. R. China. Tel: +86 731 84805380, Fax: +86 731 82354476, E-mail: yinjiye@csu.edu.cn

[#] These authors contributed equally.

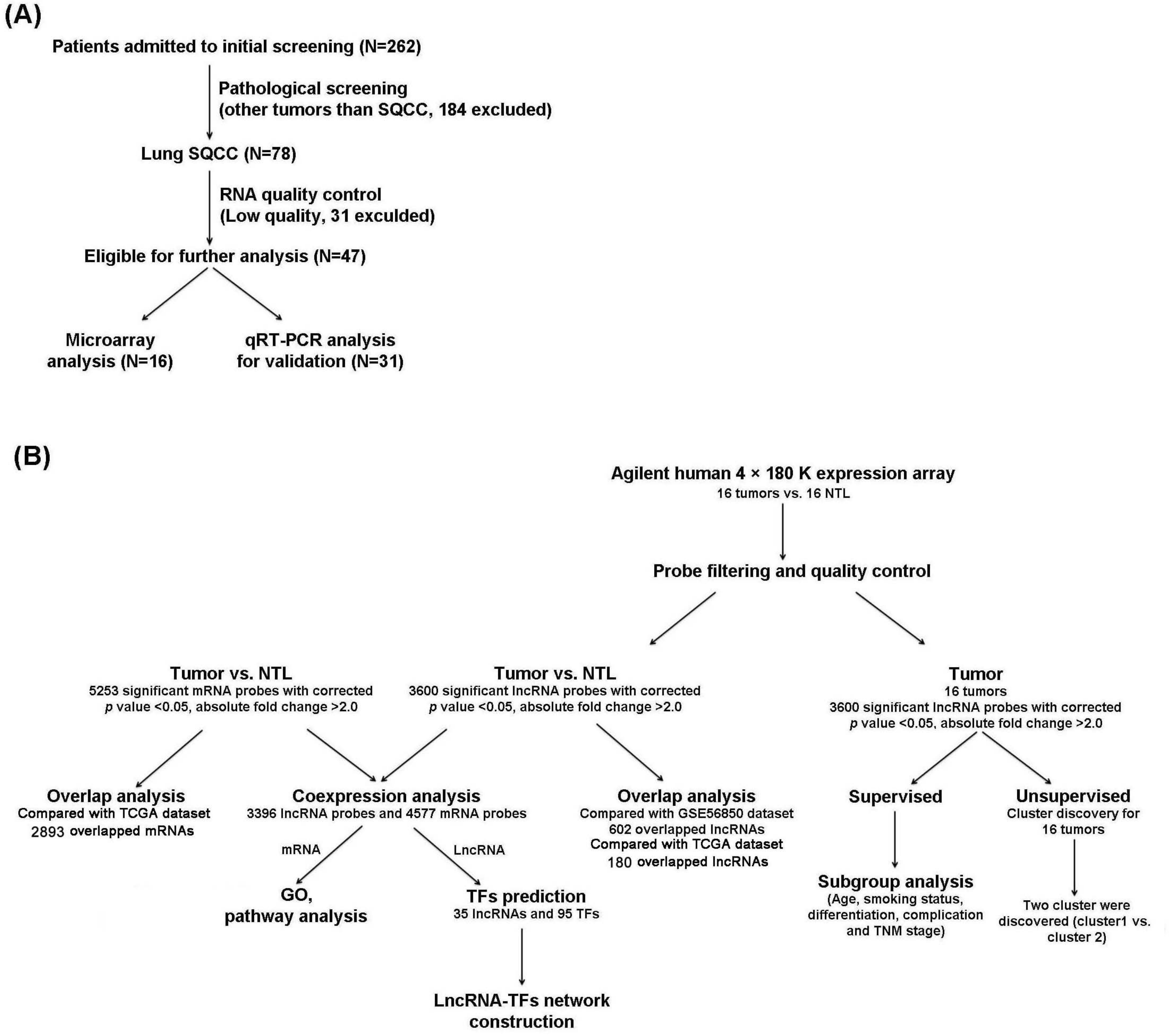

Figure S1: Flow diagram of sample selection (A) and analysis strategy (B).

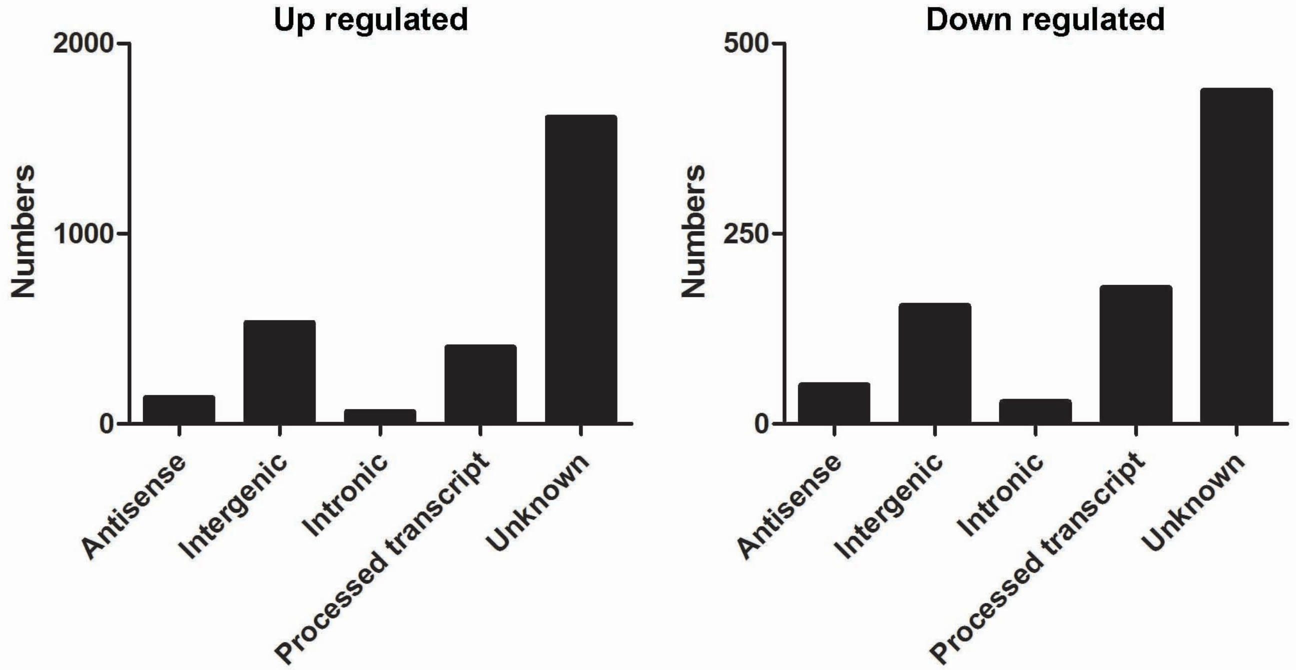

Figure S2: Distribution of the up regulated (A) and down regulated (B) lncRNAs differentially expressed in the lung SQCC tissue.

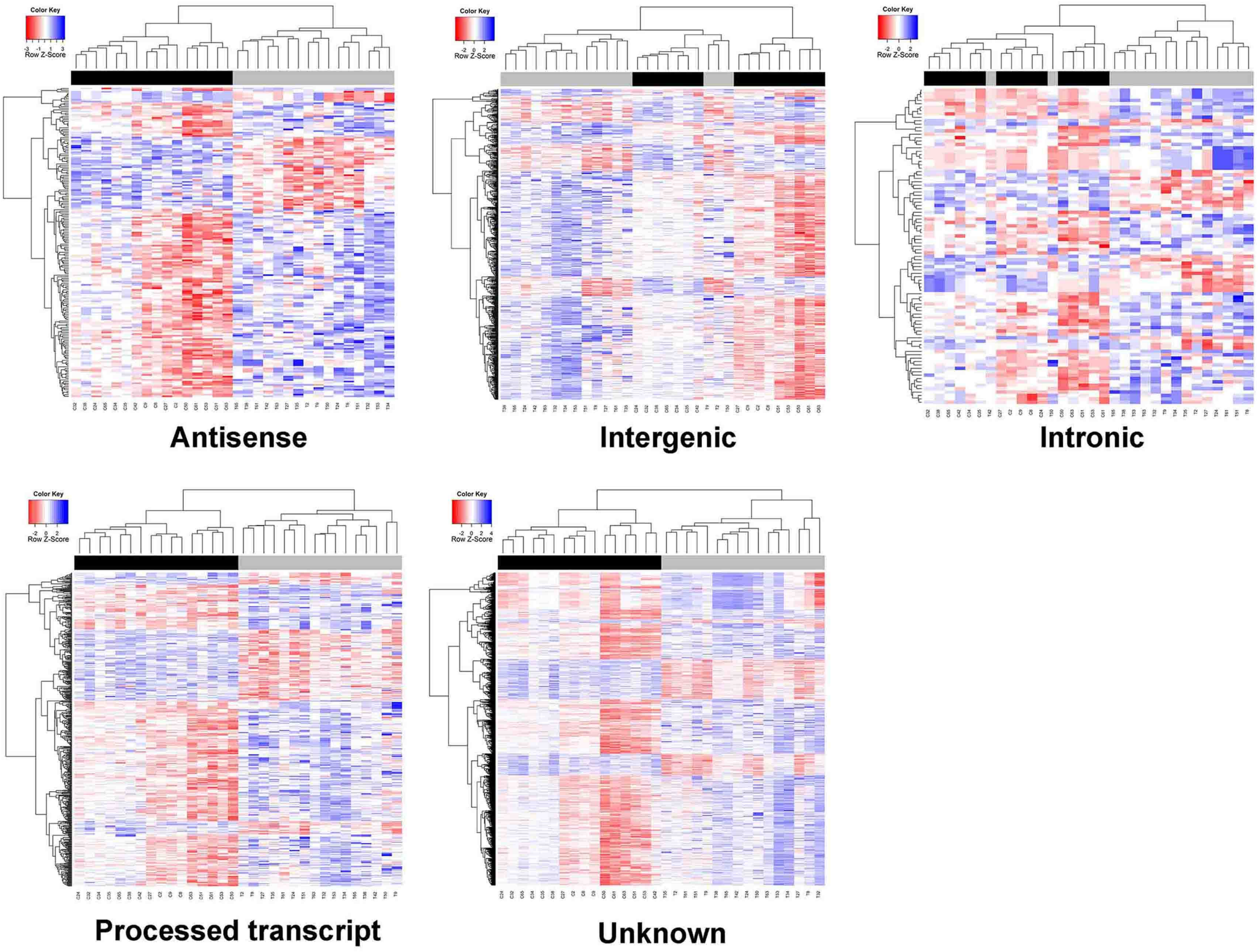

Figure S3: Two-dimensional hierarchical clustering of the significant and differentially expressed lncRNA probes in all samples according to their categorization. Probes are in rows; samples are in columns. 16 tumor tissues were in gray and 16 NTL tissues were in black.

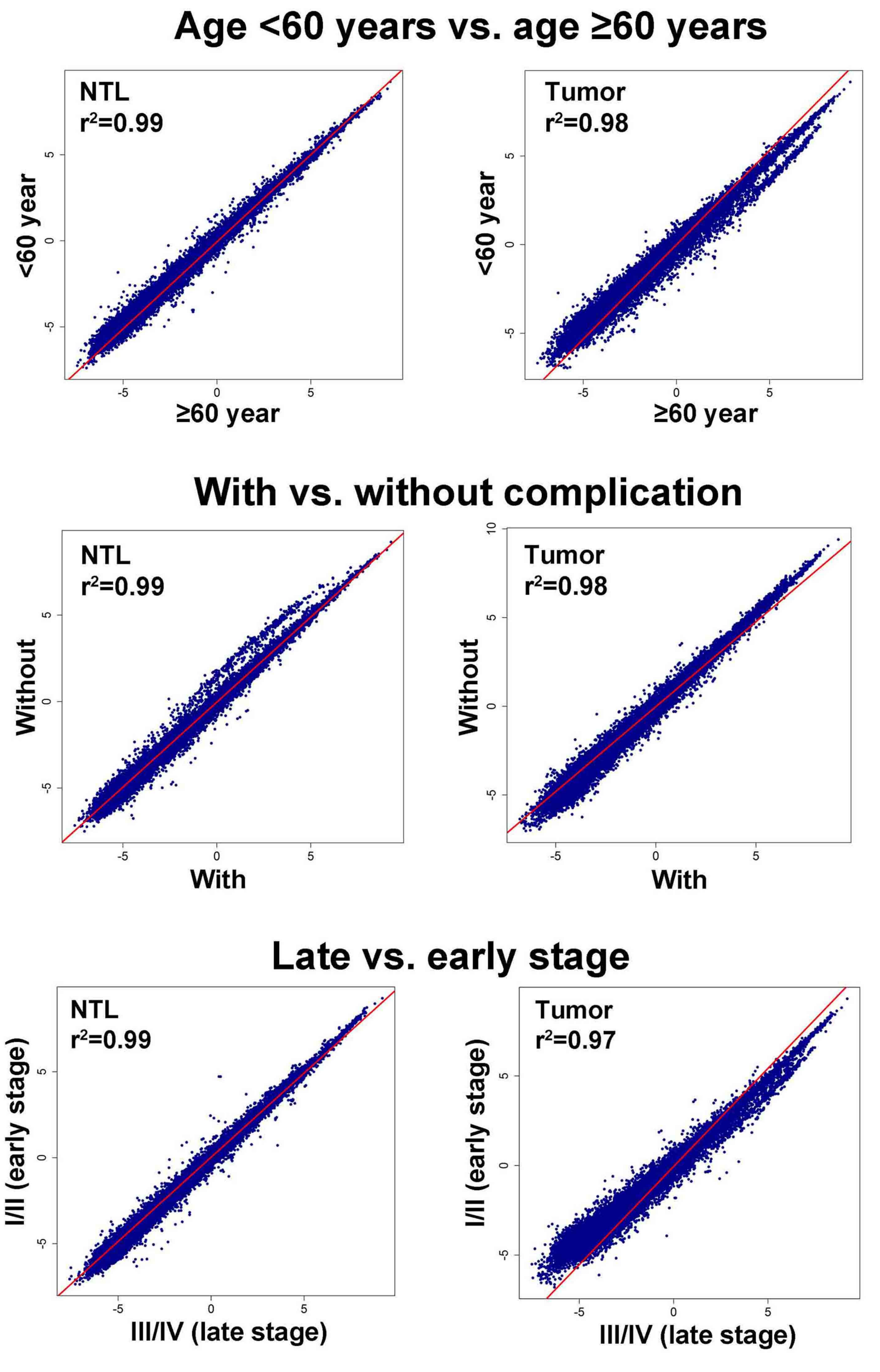

Figure S4: LncRNAs expression scatterplots of paired subgroups according to the age, complication and TNM stage in both tumor and NTL tissues. The correlation coefficient was given in the left corner.

Table S1: Significantly and differentially expressed lncRNA in tumor tissues compared with NTL

ProbeName	p (Corr)	p	FC (abs)	Regulation	Transcripts Ensemble_68	Biotype-Transcripts Ensemble_68	Name Ensemble_68
RNA178128|ENST00000554725	0.001754971	0.000275	2.047652	up	ENST00000554725	antisense	OTX2-AS1
RNA178250|ENST00000502430	0.002855752	0.000541	2.097585	up	ENST00000502430	antisense	CTD-2213F21.2
RNA176927|ENST00000423121	0.01144481	0.003713	2.107299	up	ENST00000423121	antisense	RP11-466F5.8
RNA175022|ENST00000453045	0.023053013	0.009487	2.138597	up	ENST00000453045	antisense	RP11-231K24.2
RNA176118|ENST00000424010	0.001012461	0.000129	2.185099	up	ENST00000542880	antisense	AC010980.2
RNA175852|ENST00000522961	0.014726242	0.005232	2.229099	up	ENST00000522961	antisense	RP11-65D13.1
RNA175466|ENST00000436286	0.012389709	0.004139	2.272382	up	ENST00000436286	antisense	RP4-669H2.1
RNA177197|ENST00000439545	0.000196347	1.39E-05	2.354757	up	ENST00000439545	antisense	HOTAIR
RNA176137|ENST00000441069	0.002937168	0.000563	2.404825	up	ENST00000441069	antisense	RP11-108L7.11
RNA177755|ENST00000531870	0.013577279	0.004698	3.037346	up	ENST00000500025	antisense	RP11-348A11.4
RNA176228|ENST00000499143	0.04970351	0.025361	3.331796	up	ENST00000499143	antisense	RP11-677M14.7
RNA178690|ENST00000425981	0.031848975	0.014405	3.616469	up	ENST00000425981	antisense	AC009264.1
RNA178523|ENST00000523825	0.002681739	0.000496	3.766667	up	ENST00000523825	antisense	RP11-382A18.1
RNA178689|ENST00000439694	0.000238067	1.79E-05	5.244164	up	ENST00000439694	antisense	AC009264.1
RNA178675|ENST00000454183	0.006516565	0.001705	8.035907	up	ENST00000454183	antisense	AC018730.4
RNA177363|ENST00000490916	0.002185563	0.000372	2.205665	up	ENST00000490916	antisense	RP11-204J18.3
RNA177768|ENST00000446911	0.001241468	0.00017	2.464455	up	ENST00000416200	antisense	AC017096.1
RNA177633|ENST00000536474	0.01119962	0.003609	2.768419	up	ENST00000536474	antisense	FAM222A-AS1
RNA177631|ENST00000541723	0.002350133	0.000411	2.865955	up	ENST00000536474	antisense	FAM222A-AS1
RNA177939|ENST00000420243	0.000119883	7.24E-06	5.610332	up	ENST00000420243	antisense	POU6F2-AS2
RNA176464|ENST00000450909	0.002092042	0.000352	2.090913	up	ENST00000450909	antisense	CTD-2574D22.2
RNA160868|XLOC_012342	0.000617974	6.56E-05	2.002824	up	ENST00000583492	antisense	MAFG-AS1
RNA163873|XLOC_004915	0.000460529	4.4E-05	2.047789	up	ENST00000431027	antisense	RP3-340N1.2
RNA165901|XLOC_008088	0.003188785	0.000631	2.059734	up	ENST00000572688	antisense	RP11-66N11.8
RNA158351|XLOC_001186	0.011062407	0.00355	2.069833	up	ENST00000424696	antisense	RP1-272L16.1
RNA165348|XLOC_007249	0.000474065	4.57E-05	2.086899	up	ENST00000517411	antisense	RP13-582O9.5
RNA162362|XLOC_014052	0.002995633	0.000579	2.116041	up	ENST00000437109	antisense	AP000260.4
RNA157930|XLOC_000812	0.00312608	0.000614	2.119637	up	ENST00000446167	antisense	RP11-7O11.3
RNA162514|XLOC_014338	0.002520939	0.000454	2.128641	up	ENST00000416352	antisense	RP1-76B20.11
RNA165097|XLOC_006763	0.011169238	0.003596	2.150691	up	ENST00000523365	antisense	RP11-363L24.3
RNA161835|XLOC_001755	0.0003406	2.9E-05	2.156154	up	ENST00000448117	antisense	AC104801.1

RNA160223|XLOC_011228	0.044308525	0.021951	2.162685	up	ENST00000499478	antisense	CTD-2382E5.1
RNA161865|XLOC_001777	0.004028681	0.000872	2.250261	up	ENST00000440371	antisense	AC013733.3
RNA158754|XLOC_008552	0.000480065	4.65E-05	2.264581	up	ENST00000451733	antisense	RP11-80H5.2
RNA165253|XLOC_006883	0.009065212	0.002702	2.278033	up	ENST00000523831	antisense	KB-1615E4.2
RNA159095|XLOC_009511	0.001940599	0.000317	2.337499	up	ENST00000555916	antisense	RP5-1021I20.5
RNA160459|XLOC_011686	0.001002444	0.000127	2.39503	up	ENST00000563540	antisense	RP11-146F11.5
RNA163057|XLOC_003399	0.000113917	6.77E-06	2.518874	up	ENST00000441644	antisense	AC023797.1
RNA158325|XLOC_000527	0.000981372	0.000123	2.53363	up	ENST00000425295	antisense	KDM5B-AS1
RNA164830|XLOC_006154	0.014656355	0.005199	2.550372	up	ENST00000438324	antisense	RP5-1185I7.1
RNA160294|XLOC_011306	4.75181E-05	1.92E-06	2.577542	up	ENST00000562965	antisense	RP11-941F15.1
RNA163913|XLOC_004492	0.001932302	0.000315	2.60572	up	ENST00000509656	antisense	RP11-58B2.1
RNA158004|XLOC_000228	0.007415045	0.002041	3.063855	up	ENST00000453229	antisense	RP11-230B22.1
RNA163588|XLOC_004238	0.000255578	1.97E-05	3.259296	up	ENST00000515085	antisense	RP11-310P5.2
RNA159943|XLOC_010988	0.002606725	0.000476	3.449297	up	ENST00000550118	antisense	RP11-1103G16.1
RNA162598|XLOC_014287	0.003113947	0.00061	4.158281	up	ENST00000434237	antisense	CTA-384D8.31
RNA159171|XLOC_009299	0.00062593	6.67E-05	5.573951	up	ENST00000533253	antisense	CTD-2523D13.2
RNA158966|XLOC_009398	0.000048985	2.02E-06	11.0536	up	ENST00000531363	antisense	RP11-1L12.3
RNA160870|XLOC_012590	0.000308407	2.54E-05	2.02677	up	ENST00000581303	antisense	RP13-516M14.2
RNA36428|ENCODE_1298_607	0.008919945	0.002643	2.004473	up	ENST00000447876	antisense	AC018730.1
RNA38049|ENCODE_2922_202	0.03380936	0.015563	2.006365	up	ENST00000430816	antisense	RP11-98L5.5
RNA40328|RefSeq_2373_1412	0.002023107	0.000335	2.01396	up	ENST00000502056	antisense	RP11-382A18.1
RNA46056|UCSC_4421_2127	0.000190806	1.33E-05	2.019132	up	ENST00000416416	antisense	RP11-576I22.2
RNA36330|ENCODE_1200_641	0.037784304	0.017939	2.019274	up	ENST00000453331	antisense	RP1-272L16.1
RNA44030|UCSC_1889_3272	0.007136887	0.001935	2.023434	up	ENST00000507062	antisense	RP11-471J12.1
RNA59028|asoverlaps_674_1272	0.010434295	0.003278	2.024073	up	ENST00000439444	antisense	RP4-553F4.6
RNA50055|UCSC_9258_992	0.033330534	0.015288	2.033847	up	ENST00000455933	antisense	CDKN2B-AS1
RNA59070|asoverlaps_727_1123	0.000833056	9.82E-05	2.03665	up	ENST00000524499	antisense	CTD-3065J16.9
RNA37791|ENCODE_2663_340	0.005312247	0.001282	2.049916	up	ENST00000582801	antisense	RP11-561O23.8
RNA37535|ENCODE_2407_399	0.001909523	0.00031	2.057269	up	ENST00000422764	antisense	RP11-384P7.6
RNA44857|UCSC_2912_2651	0.028622387	0.012558	2.062344	up	ENST00000439234	antisense	MIR1255B1
RNA40876|RefSeq_2969_845	0.001134644	0.00015	2.067499	up	ENST00000382313	antisense	CTD-2270P14.3
RNA40369|RefSeq_2418_1371	0.001186036	0.00016	2.069512	up	ENST00000382313	antisense	CTD-2270P14.3
RNA39964|RefSeq_1987_1753	0.005683607	0.001406	2.074786	up	ENST00000562866	antisense	RP11-368I7.2
RNA40617|RefSeq_2687_1104	0.000336444	2.85E-05	2.095819	up	ENST00000498297	antisense	RP11-723O4.2
RNA47302|UCSC_5930_1778	0.00125664	0.000173	2.114412	up	ENST00000382313	antisense	CTD-2270P14.3
RNA59135|asoverlaps_815_885	6.7425E-05	3.24E-06	2.114556	up	ENST00000561592	antisense	RP3-512B11.3

RNA49818|UCSC_8977_1052	0.011842921	0.003887	2.114695	up	ENST00000399966	antisense	WASIR1
RNA37643|ENCODE_2515_378	0.002002733	0.00033	2.127844	up	ENST00000414071	antisense	RP11-143G3.1
RNA58968|asoverlaps_600_1465	0.003079334	0.000601	2.131243	up	ENST00000577848	antisense	RP11-874J12.4
RNA50436|UCSC_9729_882	0.001945422	0.000318	2.142887	up	ENST00000515376	antisense	RP11-471J12.1
RNA37900|ENCODE_2772_304	0.014258948	0.005012	2.163551	up	ENST00000456631	antisense	RP1-137H15.2
RNA58988|asoverlaps_621_1420	0.000708686	7.93E-05	2.198729	up	ENST00000557335	antisense	RP11-84C10.4
RNA58688|asoverlaps_236_2344	0.000580414	6.02E-05	2.228351	up	ENST00000577270	antisense	RP11-156L14.1
RNA45891|UCSC_4223_2186	0.005195793	0.001245	2.237438	up	ENST00000424995	antisense	RP11-24J23.2
RNA49242|UCSC_8280_1210	0.007417089	0.002042	2.238378	up	ENST00000502430	antisense	CTD-2213F21.2
RNA37379|ENCODE_2250_429	0.000547321	5.55E-05	2.262856	up	ENST00000429305	antisense	RP11-302L19.1
RNA46604|UCSC_5107_1965	0.007901146	0.002238	2.264135	up	ENST00000454234	antisense	RP5-1185I7.1
RNA35405|ENCODE_268_1997	0.006781228	0.001802	2.291958	up	ENST00000419578	antisense	RP1-13P20.6
RNA44108|UCSC_1984_3195	0.003120469	0.000612	2.301977	up	ENST00000507062	antisense	RP11-471J12.1
RNA39340|RefSeq_1312_2380	0.002758502	0.000515	2.303117	up	ENST00000566699	antisense	RP4-659J6.2
RNA50625|UCSC_9958_819	0.000885316	0.000107	2.308364	up	ENST00000523330	antisense	RP11-539E17.4
RNA35154|ENCODE_8_5492	0.025143538	0.01062	2.353457	up	ENST00000438934	antisense	DGCR5
RNA52034|UCSC_11653_269	0.004706224	0.001082	2.35473	up	ENST00000435531	antisense	RP11-363N22.2
RNA61197|RNAz_1911_273	0.002115194	0.000357	2.364602	up	ENST00000439386	antisense	RP11-328M4.2
RNA47122|UCSC_5718_1826	0.007119387	0.001929	2.3672	up	ENST00000511755	antisense	RP11-414K1.3
RNA36146|ENCODE_1016_717	0.005470947	0.001334	2.389012	up	ENST00000414398	antisense	HCG24
RNA48216|UCSC_7059_1521	0.02157882	0.008683	2.400955	up	ENST00000559120	antisense	RP11-335K5.2
RNA36507|ENCODE_1377_586	0.000363793	3.18E-05	2.413002	up	ENST00000425089	antisense	RP11-524H19.2
RNA33827|lncRNAdb_34_1628	0.000633089	6.78E-05	2.469924	up	ENST00000522674	antisense	HOXA11-AS
RNA46949|UCSC_5504_1874	0.000867807	0.000104	2.474767	up	ENST00000436286	antisense	RP4-669H2.1
RNA59251|asoverlaps_973_424	0.02406165	0.010017	2.48696	up	ENST00000458509	antisense	AC004041.2
RNA36210|ENCODE_1080_691	0.000413449	3.81E-05	2.53125	up	ENST00000423274	antisense	BRWD1-AS1
RNA40555|RefSeq_2618_1158	0.003874832	0.000827	2.558058	up	ENST00000517519	antisense	RP11-539E17.4
RNA37106|ENCODE_1976_471	0.007556558	0.002098	2.583749	up	ENST00000414199	antisense	RP5-1066H13.4
RNA41061|RefSeq_3170_639	0.000402442	3.67E-05	2.622417	up	ENST00000565014	antisense	CTD-2574D22.5
RNA50117|UCSC_9330_978	0.00123406	0.000169	2.631105	up	ENST00000433702	antisense	TMEM106A-AS1
RNA55560|H-InvDB_2942_213	0.001203757	0.000163	2.680674	up	ENST00000439173	antisense	CSAG2
RNA58281|CombinedLit_215_852	0.000515464	5.13E-05	2.702248	up	ENST00000522674	antisense	HOXA11-AS
RNA36551|ENCODE_1421_577	4.44243E-05	1.74E-06	2.70465	up	ENST00000452525	antisense	AC022201.4
RNA45849|UCSC_4173_2203	0.005901623	0.001484	2.710304	up	ENST00000482372	antisense	RP11-147N17.1
RNA58272|CombinedLit_201_918	0.000306368	2.51E-05	2.756522	up	ENST00000522674	antisense	HOXA11-AS
RNA40177|RefSeq_2208_1577	0.00011896	7.18E-06	2.793048	up	ENST00000414089	antisense	AATK-AS1

RNA39532|RefSeq_1521_2183	0.000832243	9.81E-05	2.827805	up	ENST00000562760	antisense	RP11-22C11.2
RNA35676|ENCODE_544_1018	0.000712881	8E-05	2.848783	up	ENST00000430134	antisense	AC007386.4
RNA44369|UCSC_2313_2962	0.000103418	5.97E-06	2.891033	up	ENST00000565254	antisense	RP11-50D9.3
RNA58587|asoverlaps_109_2969	0.007456838	0.002058	2.936182	up	ENST00000500179	antisense	AC004053.1
RNA36349|ENCODE_1219_634	0.001047584	0.000135	2.939589	up	ENST00000425295	antisense	KDM5B-AS1
RNA40946|RefSeq_3044_762	0.000585442	6.09E-05	2.953618	up	ENST00000565014	antisense	CTD-2574D22.5
RNA48386|UCSC_7259_1470	0.007621315	0.002123	2.994015	up	ENST00000430027	antisense	DLX6-AS1
RNA58652|asoverlaps_186_2547	2.18771E-05	6.36E-07	3.017739	up	ENST00000565254	antisense	RP11-50D9.3
RNA50779|UCSC_10161_756	0.001518983	0.000226	3.113641	up	ENST00000439173	antisense	CSAG2
RNA36055|ENCODE_925_748	0.001734443	0.000271	3.137986	up	ENST00000425295	antisense	KDM5B-AS1
RNA50693|UCSC_10055_790	0.00205929	0.000344	3.211737	up	ENST00000523825	antisense	RP11-382A18.1
RNA36753|ENCODE_1623_540	0.001195479	0.000161	3.221257	up	ENST00000448017	antisense	RP11-556E13.1
RNA48545|UCSC_7453_1420	0.000694451	7.71E-05	3.241956	up	ENST00000523825	antisense	RP11-382A18.1
RNA59103|asoverlaps_775_970	0.000291923	2.36E-05	3.262944	up	ENST00000414354	antisense	MFI2-AS1
RNA59117|asoverlaps_791_942	0.001427138	0.000206	3.28293	up	ENST00000500498	antisense	RP11-165P7.1
RNA37817|ENCODE_2689_334	2.35922E-06	2.25E-08	3.368567	up	ENST00000428643	antisense	HMGA1P4
RNA50697|UCSC_10060_788	0.000833487	9.83E-05	3.379996	up	ENST00000439173	antisense	CSAG2
RNA37684|ENCODE_2556_369	0.000244286	1.86E-05	3.403284	up	ENST00000448017	antisense	RP11-556E13.1
RNA39745|RefSeq_1749_1990	0.001576377	0.000238	3.435281	up	ENST00000431497	antisense	DLX6-AS1
RNA50947|UCSC_10352_705	0.000513523	5.11E-05	3.460287	up	ENST00000439173	antisense	CSAG2
RNA50291|UCSC_9548_930	0.001697496	0.000263	3.55353	up	ENST00000523825	antisense	RP11-382A18.1
RNA39278|RefSeq_1246_2455	0.004637925	0.001061	3.670124	up	ENST00000539163	antisense	HNF1A-AS1
RNA44485|UCSC_2454_2884	0.021335296	0.008557	3.71896	up	ENST00000499143	antisense	RP11-677M14.7
RNA50362|UCSC_9635_907	0.002472646	0.000443	3.759732	up	ENST00000525867	antisense	AF131216.5
RNA50260|UCSC_9510_937	0.000750906	8.57E-05	4.090829	up	ENST00000480284	antisense	RP5-1121A15.1
RNA36620|ENCODE_1490_566	0.000640486	6.89E-05	4.955803	up	ENST00000448327	antisense	RP11-345L23.1
RNA49306|UCSC_8361_1189	0.000362121	3.16E-05	4.971036	up	ENST00000424208	antisense	SNHG14
RNA40191|RefSeq_2223_1560	0.000146056	9.4E-06	5.223177	up	ENST00000434346	antisense	RP11-184I16.2
RNA39123|RefSeq_1085_2653	0.003358687	0.000679	5.423403	up	ENST00000428449	antisense	RP11-560I19.4
RNA49923|UCSC_9100_1027	0.001732677	0.000271	6.140387	up	ENST00000428449	antisense	RP11-560I19.4
RNA47054|UCSC_5633_1845	9.44241E-06	1.87E-07	8.760973	up	ENST00000380330	antisense	RP11-116G8.4
RNA47381|UCSC_6025_1756	0.010627281	0.003364	2.107493	up	ENST00000479752	antisense	WWTR1-AS1
RNA40680|RefSeq_2755_1049	4.57143E-05	1.8E-06	2.323139	up	ENST00000508286	antisense	LEF1-AS1
RNA40058|RefSeq_2083_1679	1.30116E-05	2.96E-07	2.455337	up	ENST00000508286	antisense	LEF1-AS1
RNA40538|RefSeq_2601_1178	0.008301206	0.002394	2.995149	up	ENST00000536474	antisense	FAM222A-AS1
RNA177796|ENST00000545784	0.002904883	0.000555	2.002654	up	ENST00000502479	lincRNA	RP5-916L7.1

RNA177815|ENST00000537478	0.000992404	0.000125	2.007915	up	ENST00000537478	lincRNA		RP11-407A16.3
RNA175494|ENST00000420905	0.006497901	0.001698	2.022757	up	ENST00000420905	lincRNA		AC091814.3
RNA177693|ENST00000339037	0.021027414	0.008391	2.030835	up	ENST00000339037	lincRNA		AC017048.3
RNA175751|ENST00000415504	0.011884317	0.003908	2.048893	up	ENST00000440444	lincRNA		LINC00277
RNA177727|ENST00000547876	0.001204292	0.000163	2.057483	up	ENST00000547876	lincRNA		RP4-601P9.1
RNA177320|ENST00000489670	0.025358561	0.01073	2.059175	up	ENST00000489670	lincRNA		RP11-702L6.4
RNA175289|ENST00000507152	0.003358034	0.000679	2.094219	up	ENST00000507152	lincRNA		RP11-366M4.3
RNA175558|ENST00000541162	0.010790182	0.003432	2.094848	up	ENST00000541162	lincRNA		RP11-434C1.1
RNA176148|ENST00000418343	0.001478726	0.000217	2.096249	up	ENST00000418343	lincRNA		RP13-60M5.2
RNA175752|ENST00000440444	0.009367508	0.002831	2.096273	up	ENST00000440444	lincRNA		LINC00277
RNA177176|ENST00000546804	0.004270924	0.000948	2.117169	up	ENST00000546804	lincRNA		RP1-228P16.3
RNA177243|ENST00000546198	0.001463485	0.000214	2.178175	up	ENST00000546198	lincRNA		RP11-230G5.2
RNA175227|ENST00000554321	0.037530407	0.017792	2.179154	up	ENST00000554321	lincRNA		RP11-185P18.1
RNA177173|ENST00000550720	0.009966712	0.003084	2.190884	up	ENST00000550720	lincRNA		RP5-1057I20.2
RNA177636|ENST00000553177	0.03674339	0.017326	2.201494	up	ENST00000553177	lincRNA		RP1-46F2.2
RNA177701|ENST00000510694	0.008808561	0.002598	2.213364	up	ENST00000510694	lincRNA		RP11-438N16.1
RNA176189|ENST00000438128	0.000144828	9.3E-06	2.213536	up	ENST00000438128	lincRNA		AL589743.1
RNA177863|ENST00000541797	0.028059732	0.012222	2.21556	up	ENST00000541797	lincRNA		RP11-749H20.1
RNA177165|ENST00000552320	0.000652602	7.06E-05	2.234255	up	ENST00000552320	lincRNA		RP1-90J4.1
RNA177164|ENST00000550019	0.001862443	0.0003	2.280639	up	ENST00000550019	lincRNA		RP11-493L12.5
RNA177770|ENST00000538710	0.000856875	0.000102	2.291838	up	ENST00000538710	lincRNA		RP11-87C12.5
RNA175497|ENST00000535911	0.031949926	0.014461	2.297953	up	ENST00000535911	lincRNA		RP11-277P12.9
RNA175050|ENST00000540211	0.015816059	0.005756	2.308151	up	ENST00000540211	lincRNA		MIR7-3HG
RNA177876|ENST00000543651	0.00579107	0.001444	2.320902	up	ENST00000543651	lincRNA		RP11-349K16.1
RNA178143|ENST00000551588	0.003283012	0.000658	2.327125	up	ENST00000551588	lincRNA		CTD-2384A14.1
RNA175775|ENST00000551334	0.00034843	3E-05	2.330277	up	ENST00000551334	lincRNA		CTD-2314B22.3
RNA177213|ENST00000547552	0.000294286	2.38E-05	2.381551	up	ENST00000547552	lincRNA		RP11-756H6.1
RNA177475|ENST00000546710	0.002396883	0.000424	2.398658	up	ENST00000546710	lincRNA		RP11-587P21.2
RNA175782|ENST00000547285	0.001984566	0.000326	2.403423	up	ENST00000547285	lincRNA		CTD-2314B22.3
RNA177212|ENST00000547559	0.025874773	0.011012	2.458268	up	ENST00000547559	lincRNA		RP11-616L12.1
RNA178201|ENST00000373508	0.002818834	0.000532	2.476329	up	ENST00000373508	lincRNA		RP4-640H8.2
RNA175774|ENST00000551932	0.000430386	4.02E-05	2.506814	up	ENST00000551932	lincRNA		RP11-496I2.2
RNA177174|ENST00000548564	0.005231762	0.001256	2.522016	up	ENST00000548564	lincRNA		RP5-1057I20.2
RNA175601|ENST00000545400	0.027213505	0.011762	2.568839	up	ENST00000545400	lincRNA		RP11-153K16.1
RNA175594|ENST00000536744	0.009684372	0.002961	2.576711	up	ENST00000536744	lincRNA		RP11-114G22.1
RNA176180|ENST00000547220	0.000203558	1.46E-05	2.627724	up	ENST00000549065	lincRNA		CTD-2314B22.3

RNA177700|ENST00000550223	9.1918E-05	5.06E-06	2.662777	up	ENST00000550223	lincRNA	RP11-438N16.1
RNA175776|ENST00000546382	0.000197279	1.4E-05	2.711315	up	ENST00000546382	lincRNA	CTD-2314B22.3
RNA177266|ENST00000549058	0.008642696	0.002532	2.715817	up	ENST00000549058	lincRNA	RP11-314D7.4
RNA175598|ENST00000537293	0.01586828	0.005783	2.732561	up	ENST00000537293	lincRNA	RP11-449P1.1
RNA175786|ENST00000548903	0.000129018	7.99E-06	2.756198	up	ENST00000549065	lincRNA	CTD-2314B22.3
RNA177864|ENST00000536342	0.001705202	0.000265	2.756774	up	ENST00000536342	lincRNA	RP11-749H20.4
RNA177829|ENST00000546117	0.012983036	0.004412	2.808535	up	ENST00000546117	lincRNA	RP11-173C20.2
RNA176183|ENST00000418499	0.00060645	6.39E-05	2.881543	up	ENST00000418499	lincRNA	AL589743.1
RNA177371|ENST00000551726	0.016245048	0.005968	2.890482	up	ENST00000551726	lincRNA	RP11-81H3.2
RNA177265|ENST00000548029	0.001712016	0.000266	2.905975	up	ENST00000548029	lincRNA	RP11-314D7.3
RNA175593|ENST00000413794	0.00918216	0.002752	2.978163	up	ENST00000413794	lincRNA	RP11-114G22.1
RNA177691|ENST00000426615	0.004676706	0.001073	3.002177	up	ENST00000426615	lincRNA	AC009336.24
RNA177734|ENST00000552992	0.001936699	0.000316	3.00858	up	ENST00000552992	lincRNA	RP11-148B3.1
RNA178505|ENST00000515218	0.046627596	0.023388	3.018366	up	ENST00000515218	lincRNA	RP11-129M6.1
RNA177370|ENST00000515416	0.007417448	0.002042	3.153666	up	ENST00000551726	lincRNA	RP11-81H3.2
RNA175599|ENST00000541288	0.010500018	0.003306	3.172641	up	ENST00000545400	lincRNA	RP11-153K16.1
RNA175600|ENST00000534841	0.012495514	0.004186	3.245634	up	ENST00000534841	lincRNA	RP11-153K16.1
RNA174930|ENST00000508732	0.011637331	0.003798	3.353365	up	ENST00000508732	lincRNA	CTD-2536I1.1
RNA175377|ENST00000515614	0.002475009	0.000443	3.358038	up	ENST00000515614	lincRNA	RP3-340I3.1
RNA175797|ENST00000520619	0.000795745	9.26E-05	3.397729	up	ENST00000520619	lincRNA	SNHG6
RNA176192|ENST00000427798	0.000507612	5.02E-05	3.41622	up	ENST00000551545	lincRNA	LINC00516
RNA177252|ENST00000433116	0.006783618	0.001802	3.427003	up	ENST00000433116	lincRNA	RP11-637A17.2
RNA175340|ENST00000542763	0.000444083	4.18E-05	3.481206	up	ENST00000542763	lincRNA	RP11-417L19.2
RNA175785|ENST00000400192	0.00040571	3.71E-05	3.532194	up	ENST00000551545	lincRNA	LINC00516
RNA175787|ENST00000548057	3.75497E-05	1.36E-06	4.221399	up	ENST00000548057	lincRNA	CTD-2314B22.3
RNA175778|ENST00000553153	8.60573E-05	4.61E-06	5.261202	up	ENST00000553153	lincRNA	CTD-2314B22.3
RNA176190|ENST00000551881	3.1002E-05	1.03E-06	5.606118	up	ENST00000551881	lincRNA	AL589743.1
RNA175780|ENST00000549813	1.1749E-05	2.56E-07	6.872229	up	ENST00000549813	lincRNA	CTD-2314B22.3
RNA176187|ENST00000548475	4.37637E-06	5.86E-08	8.258044	up	ENST00000549813	lincRNA	CTD-2314B22.3
RNA178124|ENST00000457958	3.9579E-05	1.47E-06	8.808071	up	ENST00000457958	lincRNA	LINC00511
RNA178046|ENST00000537925	1.09776E-06	5.71E-09	2.05374	up	ENST00000537869	lincRNA	SNHG1
RNA178045|ENST00000540725	2.31158E-06	2.17E-08	2.07883	up	ENST00000537869	lincRNA	SNHG1
RNA178052|ENST00000545440	1.4367E-05	3.43E-07	2.314909	up	ENST00000541615	lincRNA	SNHG1
RNA178053|ENST00000542112	1.00649E-05	2.04E-07	2.342264	up	ENST00000541615	lincRNA	SNHG1
RNA176971|ENST00000432536	0.000590586	6.17E-05	2.461116	up	ENST00000432536	lincRNA	GAS5
RNA178054|ENST00000541615	3.82483E-05	1.4E-06	2.496894	up	ENST00000541615	lincRNA	SNHG1

RNA178056|ENST00000538266	1.4118E-05	3.35E-07	2.611327	up	ENST00000538266	lincRNA	SNHG1
RNA178063|ENST00000539303	6.91331E-06	1.16E-07	2.719819	up	ENST00000539303	lincRNA	SNHG1
RNA178062|ENST00000537024	2.75413E-06	2.86E-08	2.820103	up	ENST00000539303	lincRNA	SNHG1
RNA178047|ENST00000537068	3.36575E-06	3.96E-08	2.837343	up	ENST00000537068	lincRNA	SNHG1
RNA178055|ENST00000541578	2.51263E-06	2.48E-08	2.905677	up	ENST00000541578	lincRNA	SNHG1
RNA176374|ENST00000431060	0.005945982	0.001501	2.121505	up	ENST00000431060	lincRNA	RP11-66B24.2
RNA176490|ENST00000537498	0.001778142	0.00028	2.264472	up	ENST00000537498	lincRNA	CTD-2555A7.2
RNA162929|XLOC_003297	0.010965264	0.003507	2.00134	up	ENST00000478814	lincRNA	RP11-439C8.1
RNA163439|XLOC_004127	0.003053673	0.000594	2.003578	up	ENST00000504957	lincRNA	RP11-18H21.3
RNA165090|XLOC_007039	0.001920861	0.000313	2.010462	up	ENST00000506121	lincRNA	LINC00589
RNA159480|XLOC_010198	0.011586541	0.003776	2.014879	up	ENST00000548329	lincRNA	RP1-46F2.3
RNA158044|XLOC_000269	0.000426901	3.97E-05	2.017138	up	ENST00000452901	lincRNA	RP4-601K24.1
RNA159382|XLOC_009795	0.03267497	0.014897	2.018856	up	ENST00000544089	lincRNA	RP11-71J4.2
RNA161350|XLOC_001996	0.007463198	0.002062	2.021862	up	ENST00000433810	lincRNA	AC010145.3
RNA163264|XLOC_003988	0.019543642	0.007619	2.045454	up	ENST00000510744	lincRNA	RP11-567N4.2
RNA161113|XLOC_012981	0.019205937	0.00744	2.045868	up	ENST00000397381	lincRNA	UCA1
RNA161861|XLOC_001775	0.001361877	0.000193	2.048263	up	ENST00000456895	lincRNA	AC068196.1
RNA159552|XLOC_010246	0.000428182	3.99E-05	2.049889	up	ENST00000507482	lincRNA	RP11-983C2.2
RNA158941|XLOC_009070	0.01779123	0.00673	2.050035	up	ENST00000531749	lincRNA	RP11-98J9.1
RNA160412|XLOC_011624	0.005096301	0.001211	2.054379	up	ENST00000571152	lincRNA	LINC00514
RNA162332|XLOC_013885	0.017291449	0.00648	2.058093	up	ENST00000434859	lincRNA	AP000477.3
RNA162890|XLOC_002853	0.000243231	1.85E-05	2.060795	up	ENST00000494509	lincRNA	RP11-88H10.3
RNA163287|XLOC_003611	0.012077643	0.003997	2.061831	up	ENST00000514304	lincRNA	RP11-554D13.1
RNA164717|XLOC_006044	0.003299848	0.000663	2.073957	up	ENST00000446024	lincRNA	AC083864.3
RNA163418|XLOC_003726	0.003480309	0.000713	2.08193	up	ENST00000512752	lincRNA	RP11-292D4.1
RNA163506|XLOC_004178	0.008862837	0.002621	2.083314	up	ENST00000506984	lincRNA	RP11-598D14.1
RNA161455|XLOC_002078	0.013128582	0.004485	2.083864	up	ENST00000427020	lincRNA	AC009236.1
RNA163301|XLOC_003630	0.000141485	9.02E-06	2.087403	up	ENST00000510505	lincRNA	RP11-703G6.1
RNA163265|XLOC_003577	0.027752409	0.012046	2.09687	up	ENST00000507739	lincRNA	RP11-567N4.3
RNA163600|XLOC_004706	0.004028681	0.000872	2.097518	up	ENST00000509455	lincRNA	CTD-2194D22.2
RNA159898|XLOC_010507	0.002198953	0.000376	2.103446	up	ENST00000565936	lincRNA	RP11-315H15.3
RNA158350|XLOC_000547	0.028017351	0.012195	2.10515	up	ENST00000448988	lincRNA	RP11-372M18.1
RNA164863|XLOC_006192	0.003406773	0.000692	2.105218	up	ENST00000392471	lincRNA	RP11-44M6.1
RNA163342|XLOC_004058	0.01726303	0.006464	2.111581	up	ENST00000512219	lincRNA	RP11-236P13.1
RNA163017|XLOC_002962	0.02359798	0.009775	2.118887	up	ENST00000440726	lincRNA	RP11-567G11.1
RNA158705|XLOC_008840	0.003593631	0.000744	2.122409	up	ENST00000444086	lincRNA	RP11-153K11.3

RNA163220|XLOC_003542	0.008316718	0.0024	2.122742	up	ENST00000509711	lincRNA	RP11-463H12.2
RNA161503|XLOC_001493	0.004752627	0.001097	2.127024	up	ENST00000416845	lincRNA	AC093159.1
RNA163833|XLOC_004427	0.004356085	0.000973	2.12795	up	ENST00000512310	lincRNA	RP11-79P5.7
RNA163922|XLOC_004497	0.008362682	0.002418	2.129031	up	ENST00000522838	lincRNA	RP11-6N13.1
RNA161524|XLOC_002135	0.007083303	0.001915	2.135923	up	ENST00000420724	lincRNA	AC007389.3
RNA160375|XLOC_011596	0.002252027	0.000388	2.140621	up	ENST00000559394	lincRNA	RP11-315L6.1
RNA161845|XLOC_002402	0.008930029	0.002648	2.144063	up	ENST00000458254	lincRNA	AC016751.2
RNA160771|XLOC_012227	0.007578253	0.002106	2.152668	up	ENST00000478824	lincRNA	CTD-2377D24.6
RNA162759|XLOC_003167	0.048719123	0.024723	2.161921	up	ENST00000482003	lincRNA	RP11-47P18.1
RNA163355|XLOC_004062	0.006547892	0.001716	2.17039	up	ENST00000507299	lincRNA	CTD-2325B11.1
RNA158961|XLOC_009088	0.013303143	0.00457	2.174974	up	ENST00000532380	lincRNA	CTD-2140G10.1
RNA158602|XLOC_008420	0.006193885	0.001589	2.195935	up	ENST00000544033	lincRNA	RP13-16H11.1
RNA161912|XLOC_001826	0.001618827	0.000246	2.203931	up	ENST00000430494	lincRNA	AC007879.7
RNA163661|XLOC_004735	0.024148917	0.010065	2.222021	up	ENST00000503953	lincRNA	CTD-2154B17.1
RNA159882|XLOC_010722	0.00353027	0.000726	2.228406	up	ENST00000569854	lincRNA	RP11-120J20.4
RNA162886|XLOC_002848	0.009100293	0.002718	2.234101	up	ENST00000492307	lincRNA	RP11-80H8.3
RNA163041|XLOC_002978	0.004143948	0.000906	2.235141	up	ENST00000440556	lincRNA	RP11-513G11.2
RNA162417|XLOC_014097	0.00798983	0.002271	2.238068	up	ENST00000431150	lincRNA	AP001628.6
RNA163138|XLOC_003902	0.003605858	0.000748	2.238262	up	ENST00000508374	lincRNA	RP11-94H6.1
RNA163604|XLOC_004708	0.023059731	0.009491	2.243042	up	ENST00000511698	lincRNA	RP11-259O2.2
RNA163814|XLOC_004861	0.008514883	0.002481	2.256435	up	ENST00000479830	lincRNA	CTC-498J12.1
RNA163915|XLOC_004494	0.013416714	0.00462	2.258355	up	ENST00000501405	lincRNA	CTD-2340E1.2
RNA163329|XLOC_004051	0.002533752	0.000457	2.258845	up	ENST00000416680	lincRNA	AC107399.2
RNA162896|XLOC_002859	0.002228625	0.000383	2.265663	up	ENST00000485006	lincRNA	RP11-71N10.1
RNA163402|XLOC_004100	0.005315741	0.001284	2.272324	up	ENST00000561977	lincRNA	RP11-308D13.3
RNA163427|XLOC_003733	0.000610498	6.45E-05	2.280005	up	ENST00000505586	lincRNA	RP11-423J7.1
RNA163796|XLOC_004847	0.00573946	0.001425	2.285674	up	ENST00000510261	lincRNA	CTD-2116N20.1
RNA159597|XLOC_010519	0.00790539	0.00224	2.28674	up	ENST00000412714	lincRNA	LINC00388
RNA163481|XLOC_004157	0.009112908	0.002723	2.288909	up	ENST00000515840	lincRNA	RP11-322J23.1
RNA163644|XLOC_004279	0.005217223	0.001252	2.296368	up	ENST00000504494	lincRNA	CTD-2296D1.1
RNA161319|XLOC_001982	0.001497051	0.000221	2.301574	up	ENST00000474667	lincRNA	RP11-521D12.5
RNA160660|XLOC_012091	0.000924578	0.000114	2.305463	up	ENST00000571506	lincRNA	CTC-524C5.5
RNA163637|XLOC_004272	0.000536917	5.41E-05	2.30698	up	ENST00000506093	lincRNA	RP11-332J15.3
RNA160830|XLOC_012298	0.000679834	7.48E-05	2.312926	up	ENST00000442627	lincRNA	AC118653.2
RNA161962|XLOC_001887	0.019365236	0.007524	2.322664	up	ENST00000431435	lincRNA	AC067961.1
RNA160229|XLOC_011470	0.005206993	0.001248	2.325813	up	ENST00000558792	lincRNA	RP11-198M11.2

RNA165916|XLOC_008311	0.003124978	0.000614	2.326259	up	ENST00000417585	lincRNA	AC006335.12
RNA165538|XLOC_007769	0.003449704	0.000705	2.338419	up	ENST00000425666	lincRNA	RP11-389K14.3
RNA163271|XLOC_003588	0.00332545	0.00067	2.339118	up	ENST00000506460	lincRNA	RP11-452C8.1
RNA163060|XLOC_003402	0.014517648	0.005134	2.341779	up	ENST00000438408	lincRNA	AC128709.3
RNA162606|XLOC_003016	0.000192551	1.35E-05	2.345826	up	ENST00000418972	lincRNA	RP11-204C23.1
RNA159013|XLOC_009436	0.001460559	0.000213	2.34702	up	ENST00000530941	lincRNA	RP11-702F3.4
RNA163032|XLOC_002970	0.021749115	0.008773	2.357953	up	ENST00000412486	lincRNA	RP11-655G22.1
RNA161920|XLOC_001839	0.003012451	0.000584	2.362918	up	ENST00000448086	lincRNA	AC073284.4
RNA162175|XLOC_013775	0.007568855	0.002102	2.366948	up	ENST00000451572	lincRNA	RP1-66N13.2
RNA162928|XLOC_003296	0.008696435	0.002554	2.373675	up	ENST00000498604	lincRNA	RP11-656A15.1
RNA158469|XLOC_001287	0.007283814	0.001992	2.422003	up	ENST00000441338	lincRNA	RP11-278H7.4
RNA162380|XLOC_014066	0.002464464	0.000441	2.43918	up	ENST00000457157	lincRNA	AP000688.8
RNA163782|XLOC_004397	0.001103572	0.000145	2.443523	up	ENST00000509844	lincRNA	CTD-2023N9.3
RNA162757|XLOC_002714	0.004598104	0.001047	2.444229	up	ENST00000461186	lincRNA	RP11-354H21.2
RNA163723|XLOC_004789	0.006513373	0.001704	2.461048	up	ENST00000510938	lincRNA	CTD-2194L12.3
RNA161288|XLOC_001956	0.003443823	0.000703	2.470773	up	ENST00000412134	lincRNA	AC022311.1
RNA162952|XLOC_003317	0.001456401	0.000212	2.472118	up	ENST00000495159	lincRNA	RP11-450H5.1
RNA164992|XLOC_006310	0.002040954	0.00034	2.497542	up	ENST00000427073	lincRNA	RP5-1121A15.3
RNA162706|XLOC_002664	0.017405283	0.00654	2.502611	up	ENST00000492683	lincRNA	RP11-64C1.1
RNA159064|XLOC_009191	0.00040254	3.67E-05	2.517942	up	ENST00000561588	lincRNA	RP11-211G23.2
RNA164879|XLOC_006561	0.008752306	0.002577	2.558041	up	ENST00000435466	lincRNA	AC003088.1
RNA158001|XLOC_000855	0.009533044	0.0029	2.581073	up	ENST00000439156	lincRNA	RP11-776H12.1
RNA159225|XLOC_009615	0.008708762	0.00256	2.632786	up	ENST00000515614	lincRNA	RP3-340I3.1
RNA163709|XLOC_004772	0.025424981	0.010765	2.642621	up	ENST00000511840	lincRNA	CTD-2066L21.2
RNA163994|XLOC_004565	0.00092842	0.000114	2.648771	up	ENST00000506592	lincRNA	RP11-119J18.1
RNA164662|XLOC_005988	0.02027034	0.007991	2.675177	up	ENST00000411542	lincRNA	AC011288.2
RNA159455|XLOC_010175	0.005566826	0.001366	2.687705	up	ENST00000547179	lincRNA	LINC00485
RNA163990|XLOC_005009	0.001790229	0.000283	2.720287	up	ENST00000507035	lincRNA	CTC-276P9.1
RNA161977|XLOC_002523	0.019182628	0.007427	2.72376	up	ENST00000435896	lincRNA	AC010148.1
RNA158155|XLOC_000364	0.007859441	0.002221	2.755163	up	ENST00000442182	lincRNA	RP11-39H13.1
RNA161847|XLOC_001763	0.013573819	0.004697	2.775797	up	ENST00000426615	lincRNA	AC009336.24
RNA162916|XLOC_002877	0.00511497	0.001217	2.78832	up	ENST00000496084	lincRNA	RP11-788A4.1
RNA163721|XLOC_004341	0.032771975	0.014954	2.875727	up	ENST00000510986	lincRNA	GDNF-AS1
RNA165188|XLOC_006829	0.000297352	2.42E-05	2.890491	up	ENST00000520619	lincRNA	SNHG6
RNA164746|XLOC_006083	0.018067433	0.006869	2.914065	up	ENST00000436266	lincRNA	AC092657.2
RNA164089|XLOC_004651	0.000496256	4.87E-05	2.946699	up	ENST00000510150	lincRNA	CTD-2532K18.2

RNA159909|XLOC_010747	0.001131616	0.00015	2.972206	up	ENST00000551565	lincRNA	CTD-2311B13.1
RNA161797|XLOC_001713	0.002612753	0.000478	2.983133	up	ENST00000429916	lincRNA	AC079150.3
RNA163020|XLOC_003365	0.001170738	0.000157	2.986206	up	ENST00000446091	lincRNA	RP11-132N15.3
RNA165113|XLOC_007057	0.000882184	0.000106	2.987417	up	ENST00000519764	lincRNA	RP11-675F6.4
RNA158244|XLOC_001083	0.00108434	0.000141	2.997857	up	ENST00000441851	lincRNA	RP5-968D22.1
RNA160712|XLOC_012422	0.002841339	0.000538	3.002433	up	ENST00000578977	lincRNA	RP11-1109M24.16
RNA163987|XLOC_005008	0.009157551	0.002743	3.004481	up	ENST00000512748	lincRNA	RP11-215P8.4
RNA161530|XLOC_001507	0.000441777	4.16E-05	3.0254	up	ENST00000449673	lincRNA	AC092669.6
RNA160560|XLOC_012004	0.000407254	3.73E-05	3.030227	up	ENST00000564508	lincRNA	AC004158.3
RNA162932|XLOC_002887	0.001270008	0.000175	3.134892	up	ENST00000470024	lincRNA	RP11-451G4.3
RNA159988|XLOC_010827	1.27473E-05	2.86E-07	3.171272	up	ENST00000555801	lincRNA	RP11-255G12.1
RNA159969|XLOC_010813	0.026444998	0.01132	3.224676	up	ENST00000515218	lincRNA	RP11-129M6.1
RNA163708|XLOC_004331	0.005854092	0.001467	3.456397	up	ENST00000511054	lincRNA	CTD-2066L21.1
RNA164997|XLOC_006676	4.69869E-07	1.11E-09	3.490897	up	ENST00000442017	lincRNA	RP5-1142J19.1
RNA162817|XLOC_003218	0.013988288	0.00489	3.614773	up	ENST00000481624	lincRNA	RP11-572C15.5
RNA161949|XLOC_001872	5.59328E-05	2.47E-06	3.69554	up	ENST00000414512	lincRNA	AC067956.1
RNA160411|XLOC_011856	0.000176007	1.2E-05	3.760432	up	ENST00000575693	lincRNA	LA16c-325D7.2
RNA160892|XLOC_012746	0.000966011	0.000121	3.955201	up	ENST00000558690	lincRNA	RP11-838N2.3
RNA162116|XLOC_013502	0.004204694	0.000925	4.220669	up	ENST00000449316	lincRNA	RP4-760C5.5
RNA163263|XLOC_003576	0.028527897	0.012498	4.251579	up	ENST00000512043	lincRNA	RP11-542G1.3
RNA164736|XLOC_006422	0.000270633	2.14E-05	4.283275	up	ENST00000456114	lincRNA	AC005537.2
RNA162652|XLOC_002603	0.001677448	0.000259	4.472795	up	ENST00000414382	lincRNA	AC098973.2
RNA163300|XLOC_004031	0.02108573	0.008419	4.753486	up	ENST00000509399	lincRNA	RP11-297P16.4
RNA159487|XLOC_010204	0.000191563	1.34E-05	5.003696	up	ENST00000547963	lincRNA	RP11-438N16.1
RNA158247|XLOC_000455	0.000316408	2.63E-05	5.011541	up	ENST00000420691	lincRNA	RP1-10C16.2
RNA162877|XLOC_003263	0.005104427	0.001214	6.24234	up	ENST00000466206	lincRNA	RP11-657O9.1
RNA158023|XLOC_000254	0.000212035	1.54E-05	6.383864	up	ENST00000418078	lincRNA	RP11-262K1.1
RNA159238|XLOC_009627	3.2693E-06	3.7E-08	10.40551	up	ENST00000537192	lincRNA	RP11-1038A11.3
RNA161926|XLOC_001851	3.09961E-05	1.03E-06	10.63038	up	ENST00000415479	lincRNA	AC093850.2
RNA163182|XLOC_003506	0.011669108	0.003812	2.368498	up	ENST00000562049	lincRNA	RP11-103J17.2
RNA165174|XLOC_006823	0.001603155	0.000243	2.721766	up	ENST00000520799	lincRNA	RP11-32K4.2
RNA35797|ENCODE_666_880	0.005174829	0.001238	2.001588	up	ENST00000434458	lincRNA	RP13-16H11.1
RNA36184|ENCODE_1054_702	0.001596927	0.000242	2.001804	up	ENST00000413352	lincRNA	RP11-375O18.2
RNA37982|ENCODE_2855_265	0.006838482	0.001822	2.006094	up	ENST00000424506	lincRNA	RP3-463P15.1
RNA39969|RefSeq_1993_1751	0.003884752	0.000831	2.007381	up	ENST00000580197	lincRNA	RP11-146G7.3
RNA37207|ENCODE_2077_453	0.010362142	0.003248	2.00854	up	ENST00000455010	lincRNA	RP11-90C4.3

RNA40380|RefSeq_2431_1362	0.002530728	0.000456	2.010285	up	ENST00000440089	lincRNA	AC105339.1
RNA37990|ENCODE_2863_259	0.002118267	0.000357	2.010672	up	ENST00000319701	lincRNA	RP11-380B22.1
RNA46785|UCSC_5312_1920	0.000374846	3.31E-05	2.010942	up	ENST00000540895	lincRNA	RP11-114G22.1
RNA36741|ENCODE_1611_543	0.04607794	0.023044	2.01437	up	ENST00000438072	lincRNA	RP11-111F5.3
RNA35305|ENCODE_164_2398	0.000952845	0.000118	2.016075	up	ENST00000423455	lincRNA	RP11-54A22.1
RNA46433|UCSC_4895_2014	0.013361844	0.004596	2.016359	up	ENST00000448740	lincRNA	RP3-486I3.7
RNA35202|ENCODE_57_3328	0.038697068	0.018491	2.017823	up	ENST00000380888	lincRNA	RP4-610C12.4
RNA37932|ENCODE_2805_290	0.003898351	0.000834	2.019466	up	ENST00000458480	lincRNA	RP11-452B18.2
RNA39479|RefSeq_1464_2231	0.004506312	0.001019	2.020991	up	ENST00000412816	lincRNA	LINC00470
RNA43135|UCSC_818_4388	0.00257223	0.000467	2.021688	up	ENST00000567749	lincRNA	RP13-735L24.1
RNA39389|RefSeq_1365_2322	0.009797878	0.003008	2.026418	up	ENST00000518580	lincRNA	RP11-109J4.1
RNA37639|ENCODE_2511_379	0.002002733	0.00033	2.027301	up	ENST00000420601	lincRNA	RP11-235G24.3
RNA37799|ENCODE_2671_339	0.026961822	0.011612	2.028581	up	ENST00000425153	lincRNA	RP11-205K6.2
RNA38811|RefSeq_752_3221	0.004728516	0.001089	2.029461	up	ENST00000571152	lincRNA	LINC00514
RNA37057|ENCODE_1927_478	0.003141308	0.000618	2.030143	up	ENST00000437466	lincRNA	RP11-524P6.1
RNA36585|ENCODE_1455_571	0.001627812	0.000248	2.030576	up	ENST00000413244	lincRNA	CTA-929C8.8
RNA43181|UCSC_871_4285	0.044555143	0.022109	2.031563	up	ENST00000562917	lincRNA	RP11-1C8.5
RNA39529|RefSeq_1518_2185	0.003538649	0.000729	2.032651	up	ENST00000412816	lincRNA	LINC00470
RNA51780|UCSC_11373_383	0.008989017	0.002671	2.035482	up	ENST00000581430	lincRNA	LINC00470
RNA35860|ENCODE_730_835	0.004379168	0.000979	2.036397	up	ENST00000452501	lincRNA	RP5-1172N10.2
RNA47093|UCSC_5682_1835	0.026881427	0.011568	2.036667	up	ENST00000447529	lincRNA	AC078842.4
RNA37384|ENCODE_2255_428	0.019501047	0.007596	2.039709	up	ENST00000419779	lincRNA	RP11-508N22.9
RNA37949|ENCODE_2822_282	0.042286217	0.020682	2.040415	up	ENST00000443621	lincRNA	LINC00393
RNA37940|ENCODE_2813_285	0.00197962	0.000325	2.041076	up	ENST00000437471	lincRNA	RP11-23D5.1
RNA44648|UCSC_2653_2781	0.002898438	0.000553	2.041504	up	ENST00000572380	lincRNA	AP000479.1
RNA41032|RefSeq_3140_659	0.040473066	0.019557	2.042093	up	ENST00000330337	lincRNA	TTTY13
RNA51387|UCSC_10907_544	0.003011519	0.000583	2.042636	up	ENST00000564830	lincRNA	RP11-417E7.2
RNA36470|ENCODE_1340_596	0.029158007	0.012872	2.043243	up	ENST00000423925	lincRNA	AC008069.1
RNA40942|RefSeq_3040_767	0.004192798	0.000921	2.043403	up	ENST00000568256	lincRNA	CDRT7
RNA34105|NRED_236_2899	0.011534952	0.003753	2.043822	up	ENST00000443246	lincRNA	RP11-478H13.2
RNA36121|ENCODE_991_726	0.03165434	0.014291	2.045276	up	ENST00000436530	lincRNA	AL163953.2
RNA40368|RefSeq_2417_1371	0.016041925	0.005868	2.045931	up	ENST00000569242	lincRNA	RP11-488I20.3
RNA45401|UCSC_3598_2376	0.003574427	0.000738	2.046214	up	ENST00000545202	lincRNA	AP000439.1
RNA37973|ENCODE_2846_271	0.000165361	1.1E-05	2.04912	up	ENST00000416563	lincRNA	RP13-329D4.3
RNA36777|ENCODE_1647_535	0.009572216	0.002916	2.050216	up	ENST00000425850	lincRNA	AC116609.2
RNA36978|ENCODE_1848_492	0.015536706	0.005623	2.054558	up	ENST00000448988	lincRNA	RP11-372M18.1

RNA40746|RefSeq_2826_982	0.008886438	0.00263	2.055662	up	ENST00000520457	lincRNA	HPYR1
RNA37247|ENCODE_2117_446	0.008826297	0.002607	2.057642	up	ENST00000456559	lincRNA	RP11-733O18.1
RNA35176|ENCODE_30_4049	0.011921218	0.003926	2.057817	up	ENST00000444158	lincRNA	AC006372.4
RNA35944|ENCODE_814_794	0.009377325	0.002836	2.060216	up	ENST00000438722	lincRNA	AC018737.3
RNA35495|ENCODE_359_1712	0.000729914	8.26E-05	2.061711	up	ENST00000452901	lincRNA	RP4-601K24.1
RNA46378|UCSC_4820_2031	0.005763628	0.001434	2.061851	up	ENST00000412816	lincRNA	LINC00470
RNA37348|ENCODE_2219_433	0.009959419	0.00308	2.062083	up	ENST00000453798	lincRNA	AC073094.4
RNA48076|UCSC_6879_1564	0.008611918	0.002519	2.063524	up	ENST00000420529	lincRNA	RP5-828H9.3
RNA38050|ENCODE_2923_202	0.002680953	0.000496	2.064069	up	ENST00000425157	lincRNA	RP11-436F21.3
RNA37623|ENCODE_2495_381	0.01232307	0.004106	2.065031	up	ENST00000425900	lincRNA	RP4-764O22.1
RNA35967|ENCODE_837_787	0.047379836	0.023859	2.065675	up	ENST00000435843	lincRNA	RP11-34F20.5
RNA35526|ENCODE_390_1542	0.009274238	0.002791	2.065882	up	ENST00000420529	lincRNA	RP5-828H9.3
RNA37214|ENCODE_2084_451	0.00874201	0.002573	2.067234	up	ENST00000438185	lincRNA	LL22NC03-102D1.18
RNA36067|ENCODE_937_745	0.030490933	0.013618	2.073288	up	ENST00000580244	lincRNA	RP11-561O23.5
RNA52871|H-InvDB_126_921	0.006736268	0.001785	2.073413	up	ENST00000567343	lincRNA	RP11-33B1.4
RNA61083|RNAz_1797_275	0.0142511	0.005008	2.073779	up	ENST00000562945	lincRNA	RP11-488I20.9
RNA49434|UCSC_8513_1156	0.016069073	0.005882	2.079876	up	ENST00000295549	lincRNA	AC017048.3
RNA47301|UCSC_5929_1779	0.00140702	0.000202	2.080923	up	ENST00000505329	lincRNA	RP11-344G13.1
RNA37571|ENCODE_2443_391	0.010568031	0.003339	2.082011	up	ENST00000441660	lincRNA	RP5-827L5.1
RNA37816|ENCODE_2688_334	0.013187927	0.004513	2.082928	up	ENST00000423349	lincRNA	RP11-272J7.4
RNA35919|ENCODE_789_805	0.007478625	0.002068	2.083417	up	ENST00000432834	lincRNA	RP5-860P4.2
RNA36345|ENCODE_1215_635	0.00105259	0.000136	2.085451	up	ENST00000443419	lincRNA	AC073628.1
RNA36628|ENCODE_1498_564	0.011725763	0.003838	2.092544	up	ENST00000423925	lincRNA	AC008069.1
RNA50836|UCSC_10222_739	0.021170069	0.008466	2.093069	up	ENST00000564762	lincRNA	CTD-2008P7.1
RNA59122|asoverlaps_798_925	0.003596345	0.000745	2.093295	up	ENST00000565518	lincRNA	RP11-923I11.3
RNA37516|ENCODE_2388_403	0.021156875	0.008457	2.096978	up	ENST00000412647	lincRNA	RP11-543E8.1
RNA35300|ENCODE_159_2434	0.004060314	0.000882	2.099823	up	ENST00000425879	lincRNA	AC068057.2
RNA35954|ENCODE_824_791	0.004540471	0.001029	2.100264	up	ENST00000449990	lincRNA	RP11-555F9.2
RNA44612|UCSC_2602_2806	0.001947549	0.000318	2.1034	up	ENST00000512833	lincRNA	RP13-539F13.3
RNA37696|ENCODE_2568_365	0.004499637	0.001016	2.113603	up	ENST00000454056	lincRNA	GS1-756B1.2
RNA35280|ENCODE_139_2562	0.013584622	0.004701	2.113708	up	ENST00000412609	lincRNA	AC093063.1
RNA37380|ENCODE_2251_428	0.000731454	8.29E-05	2.117337	up	ENST00000418660	lincRNA	LINC00404
RNA51451|UCSC_10992_518	0.003072841	0.000599	2.117851	up	ENST00000566054	lincRNA	RP11-65J21.1
RNA37093|ENCODE_1963_473	0.025821298	0.010983	2.118693	up	ENST00000443565	lincRNA	RP5-887A10.1
RNA37309|ENCODE_2180_438	0.04623781	0.023148	2.118805	up	ENST00000451295	lincRNA	RP11-282I1.2
RNA34338|NRED_469_2211	9.76672E-05	5.52E-06	2.12103	up	ENST00000562231	lincRNA	RP11-314O13.1

RNA37541|ENCODE_2413_398	7.58901E-05	3.84E-06	2.122252	up	ENST00000426283	lincRNA	RP11-92J19.3
RNA35899|ENCODE_769_816	0.000554436	5.64E-05	2.123245	up	ENST00000454128	lincRNA	AF127936.3
RNA35316|ENCODE_175_2344	0.001649665	0.000252	2.124472	up	ENST00000457478	lincRNA	AC019118.2
RNA36249|ENCODE_1119_675	0.030039085	0.013372	2.124862	up	ENST00000452465	lincRNA	RP11-98G7.1
RNA40260|RefSeq_2299_1482	0.000404925	3.7E-05	2.129597	up	ENST00000507482	lincRNA	RP11-983C2.2
RNA36543|ENCODE_1413_579	0.003769873	0.000796	2.129912	up	ENST00000447908	lincRNA	RP4-704D23.1
RNA36314|ENCODE_1184_649	0.00121883	0.000166	2.132193	up	ENST00000422203	lincRNA	AC096669.1
RNA50854|UCSC_10246_732	0.000960075	0.000119	2.140252	up	ENST00000565798	lincRNA	CTD-2118P12.1
RNA36520|ENCODE_1390_582	0.002462693	0.00044	2.140554	up	ENST00000458577	lincRNA	AC004070.1
RNA36633|ENCODE_1503_562	0.020360388	0.008039	2.142414	up	ENST00000454135	lincRNA	RP1-28O17.1
RNA35542|ENCODE_408_1484	0.003508515	0.00072	2.149983	up	ENST00000415932	lincRNA	RP11-199O14.1
RNA37338|ENCODE_2209_435	0.013995934	0.004895	2.151518	up	ENST00000444130	lincRNA	AP000705.7
RNA36675|ENCODE_1545_554	0.001205516	0.000163	2.151693	up	ENST00000453579	lincRNA	RP1-257I9.2
RNA36919|ENCODE_1789_504	0.00037225	3.28E-05	2.15386	up	ENST00000457901	lincRNA	AC067959.1
RNA44460|UCSC_2424_2901	0.002264439	0.000391	2.156787	up	ENST00000572380	lincRNA	AP000479.1
RNA35168|ENCODE_22_4394	0.038879793	0.018603	2.160398	up	ENST00000414159	lincRNA	AC114776.3
RNA37069|ENCODE_1939_477	0.027362118	0.011841	2.160643	up	ENST00000366224	lincRNA	RP11-571E6.3
RNA35934|ENCODE_804_799	0.026575724	0.011393	2.163009	up	ENST00000432431	lincRNA	AC023128.1
RNA37890|ENCODE_2762_308	0.001860927	0.000299	2.171071	up	ENST00000448241	lincRNA	AC023137.2
RNA36722|ENCODE_1592_546	0.014087235	0.004937	2.171392	up	ENST00000414401	lincRNA	CECR7
RNA38830|RefSeq_771_3182	0.026976949	0.011622	2.179025	up	ENST00000580244	lincRNA	RP11-561O23.5
RNA39107|RefSeq_1068_2678	0.001847656	0.000296	2.183212	up	ENST00000501259	lincRNA	RP11-461O7.1
RNA36037|ENCODE_907_756	0.00030073	2.45E-05	2.185074	up	ENST00000456037	lincRNA	RP11-149B9.2
RNA50616|UCSC_9948_822	0.001047351	0.000135	2.186584	up	ENST00000523792	lincRNA	RP11-398G24.2
RNA51124|UCSC_10574_638	0.013126693	0.004484	2.192123	up	ENST00000576745	lincRNA	RP11-388M20.9
RNA45289|UCSC_3461_2422	0.00774147	0.002171	2.193635	up	ENST00000507152	lincRNA	RP11-366M4.3
RNA35864|ENCODE_734_834	0.009143935	0.002737	2.196581	up	ENST00000440848	lincRNA	RP11-145H9.3
RNA47782|UCSC_6531_1641	0.025472296	0.010793	2.201484	up	ENST00000580244	lincRNA	RP11-561O23.5
RNA36051|ENCODE_921_750	0.013918018	0.004855	2.201978	up	ENST00000456235	lincRNA	AC087393.1
RNA35686|ENCODE_554_1013	0.010695344	0.003395	2.202922	up	ENST00000425666	lincRNA	RP11-389K14.3
RNA37710|ENCODE_2582_362	0.048958935	0.02487	2.212977	up	ENST00000429884	lincRNA	BX004987.6
RNA36733|ENCODE_1603_544	5.16001E-05	2.18E-06	2.214772	up	ENST00000420096	lincRNA	Z83851.4
RNA36647|ENCODE_1517_561	0.000412456	3.79E-05	2.216274	up	ENST00000442627	lincRNA	AC118653.2
RNA37439|ENCODE_2310_418	0.000758794	8.7E-05	2.217825	up	ENST00000414126	lincRNA	AC073316.2
RNA35128|NRED_1269_319	0.007446734	0.002054	2.217902	up	ENST00000536536	lincRNA	LINC00302
RNA35829|ENCODE_699_852	0.001121552	0.000148	2.219514	up	ENST00000532591	lincRNA	LINC00301

RNA38637|RefSeq_565_3678	0.004619741	0.001055	2.228295	up	ENST00000509760	lincRNA	RP11-143E21.7
RNA35840|ENCODE_710_847	0.009840744	0.003027	2.229144	up	ENST00000434859	lincRNA	AP000477.3
RNA39673|RefSeq_1670_2043	0.01409368	0.00494	2.231047	up	ENST00000458627	lincRNA	FAM41AY2
RNA37147|ENCODE_2017_465	0.014522121	0.005136	2.231216	up	ENST00000431987	lincRNA	RP11-168P8.4
RNA36072|ENCODE_942_743	0.000400635	3.64E-05	2.231604	up	ENST00000454128	lincRNA	AF127936.3
RNA50879|UCSC_10276_724	0.0425876	0.020872	2.23242	up	ENST00000565798	lincRNA	CTD-2118P12.1
RNA40583|RefSeq_2648_1130	0.009907174	0.003057	2.233618	up	ENST00000412609	lincRNA	AC093063.1
RNA36548|ENCODE_1418_578	0.004628004	0.001057	2.248345	up	ENST00000437610	lincRNA	AC011995.3
RNA35778|ENCODE_647_897	0.004909609	0.001148	2.258719	up	ENST00000420255	lincRNA	AP000472.2
RNA37693|ENCODE_2565_366	0.004329415	0.000965	2.26433	up	ENST00000411785	lincRNA	AC118345.1
RNA36835|ENCODE_1705_523	0.004394728	0.000984	2.270321	up	ENST00000441085	lincRNA	RP11-98G7.1
RNA40529|RefSeq_2589_1189	0.011298743	0.00365	2.274296	up	ENST00000488852	lincRNA	RP11-631B21.1
RNA35498|ENCODE_362_1709	0.03398538	0.015672	2.28105	up	ENST00000443897	lincRNA	AC010096.1
RNA35904|ENCODE_774_814	0.000882817	0.000106	2.287773	up	ENST00000433081	lincRNA	RP11-458D21.1
RNA41176|RefSeq_3297_512	0.014280906	0.005021	2.293851	up	ENST00000452889	lincRNA	TTTY23
RNA35875|ENCODE_745_829	0.00293701	0.000563	2.294564	up	ENST00000419299	lincRNA	LINC00320
RNA37159|ENCODE_2029_462	0.007832968	0.002209	2.303012	up	ENST00000431460	lincRNA	RP4-710H13.2
RNA37946|ENCODE_2819_282	0.000984477	0.000124	2.306966	up	ENST00000450669	lincRNA	RP11-107I14.5
RNA44372|UCSC_2317_2957	0.009008954	0.00268	2.307827	up	ENST00000436078	lincRNA	RP11-91K9.1
RNA49073|UCSC_8082_1260	0.001842164	0.000295	2.310321	up	ENST00000414416	lincRNA	LINC00116
RNA46112|UCSC_4490_2112	0.013656954	0.004734	2.310749	up	ENST00000412134	lincRNA	AC022311.1
RNA35485|ENCODE_349_1730	0.007941143	0.002253	2.313669	up	ENST00000428118	lincRNA	LA16c-2F2.8
RNA37632|ENCODE_2504_380	0.008551302	0.002495	2.31497	up	ENST00000457385	lincRNA	AC093822.1
RNA37813|ENCODE_2685_335	0.009210853	0.002764	2.318802	up	ENST00000436077	lincRNA	RP11-20F24.2
RNA49734|UCSC_8869_1077	0.003808572	0.000808	2.3189	up	ENST00000566930	lincRNA	RP11-215E13.1
RNA36696|ENCODE_1566_551	1.56771E-05	3.92E-07	2.321451	up	ENST00000446578	lincRNA	Z83851.3
RNA49058|UCSC_8065_1263	0.00028103	2.25E-05	2.321859	up	ENST00000501050	lincRNA	AC006445.8
RNA48207|UCSC_7047_1524	0.000198972	1.41E-05	2.323884	up	ENST00000446091	lincRNA	RP11-132N15.3
RNA37745|ENCODE_2617_353	0.010038286	0.003112	2.326921	up	ENST00000442108	lincRNA	RP11-12M5.4
RNA37405|ENCODE_2276_424	0.023419466	0.009678	2.333842	up	ENST00000413151	lincRNA	AC009505.4
RNA37827|ENCODE_2699_330	0.013662872	0.004737	2.338725	up	ENST00000434117	lincRNA	LINC00353
RNA37576|ENCODE_2448_390	0.025287023	0.010696	2.340604	up	ENST00000429507	lincRNA	RP5-855F14.2
RNA45818|UCSC_4129_2214	0.001333286	0.000188	2.350843	up	ENST00000508484	lincRNA	AC006552.1
RNA36851|ENCODE_1721_519	0.016282476	0.005986	2.353424	up	ENST00000453395	lincRNA	LA16c-83F12.6
RNA37434|ENCODE_2305_419	0.006408083	0.001665	2.354986	up	ENST00000424162	lincRNA	RP1-60O19.2
RNA37937|ENCODE_2810_289	0.001479823	0.000218	2.355617	up	ENST00000456368	lincRNA	AC093063.1

RNA37373|ENCODE_2244_429	0.003706048	0.000776	2.367364	up	ENST00000443366	lincRNA	RP3-399C22.1
RNA36618|ENCODE_1488_566	0.039611444	0.019026	2.374178	up	ENST00000412563	lincRNA	AC007091.1
RNA36463|ENCODE_1333_598	0.001587985	0.00024	2.376823	up	ENST00000437967	lincRNA	AC006037.2
RNA36649|ENCODE_1519_560	0.003864121	0.000824	2.377598	up	ENST00000447784	lincRNA	RP11-442J17.2
RNA36718|ENCODE_1588_547	0.008116	0.002321	2.377911	up	ENST00000414065	lincRNA	AC018685.1
RNA36409|ENCODE_1279_613	0.000728146	8.23E-05	2.379343	up	ENST00000450623	lincRNA	RP5-1010E17.1
RNA36130|ENCODE_1000_722	0.003751017	0.00079	2.389623	up	ENST00000418612	lincRNA	AC051649.6
RNA39973|RefSeq_1997_1749	0.000835401	9.87E-05	2.39148	up	ENST00000501050	lincRNA	AC006445.8
RNA36697|ENCODE_1567_551	0.004520042	0.001023	2.397847	up	ENST00000451572	lincRNA	RP1-66N13.2
RNA44431|UCSC_2388_2922	0.001826825	0.000291	2.399715	up	ENST00000509760	lincRNA	RP11-143E21.7
RNA37580|ENCODE_2452_389	0.002637593	0.000484	2.400111	up	ENST00000422548	lincRNA	RP5-968D22.1
RNA36494|ENCODE_1364_591	0.010132995	0.003154	2.403345	up	ENST00000443565	lincRNA	RP5-887A10.1
RNA45183|UCSC_3317_2481	0.008306019	0.002396	2.404038	up	ENST00000501520	lincRNA	RP11-57A19.2
RNA34139|NRED_270_2729	0.000224994	1.66E-05	2.404283	up	ENST00000551932	lincRNA	RP11-496I2.2
RNA35614|ENCODE_482_1184	0.000288196	2.32E-05	2.413331	up	ENST00000420725	lincRNA	RP11-707P20.1
RNA35972|ENCODE_842_786	0.007724517	0.002165	2.413853	up	ENST00000445585	lincRNA	RP5-899B16.2
RNA36149|ENCODE_1019_717	0.028491355	0.012478	2.418221	up	ENST00000426008	lincRNA	OSTM1-AS1
RNA48590|UCSC_7506_1406	0.001614379	0.000245	2.421698	up	ENST00000547285	lincRNA	CTD-2314B22.3
RNA37837|ENCODE_2709_326	0.03029893	0.013511	2.42552	up	ENST00000447257	lincRNA	RP11-372M18.2
RNA48593|UCSC_7509_1405	0.002624296	0.000481	2.425678	up	ENST00000547285	lincRNA	CTD-2314B22.3
RNA36262|ENCODE_1132_671	0.023919186	0.009941	2.426619	up	ENST00000420549	lincRNA	RP11-147G16.1
RNA37718|ENCODE_2590_359	0.011471229	0.003725	2.431904	up	ENST00000434321	lincRNA	AC005019.3
RNA35718|ENCODE_586_957	0.022799512	0.009341	2.435	up	ENST00000447613	lincRNA	RP11-327I22.5
RNA37049|ENCODE_1919_479	0.002059814	0.000344	2.437637	up	ENST00000442558	lincRNA	RP11-147C23.1
RNA40296|RefSeq_2339_1438	0.006165333	0.001579	2.446557	up	ENST00000568609	lincRNA	PWRN1
RNA36188|ENCODE_1058_698	0.001861492	0.000299	2.451871	up	ENST00000455487	lincRNA	RP11-54H7.2
RNA37402|ENCODE_2273_424	0.001403866	0.000201	2.462515	up	ENST00000416845	lincRNA	AC093159.1
RNA35807|ENCODE_676_872	0.003159561	0.000623	2.466234	up	ENST00000419817	lincRNA	RP11-458D21.1
RNA37943|ENCODE_2816_284	0.00205929	0.000344	2.468885	up	ENST00000437233	lincRNA	AC084149.2
RNA35785|ENCODE_654_890	0.00219772	0.000375	2.474147	up	ENST00000427918	lincRNA	RP11-501G7.1
RNA37036|ENCODE_1906_480	0.001539704	0.00023	2.478496	up	ENST00000416182	lincRNA	AF241725.6
RNA45565|UCSC_3806_2316	0.000768308	8.85E-05	2.486498	up	ENST00000517491	lincRNA	AC145110.1
RNA49455|UCSC_8538_1149	0.005109799	0.001215	2.488042	up	ENST00000515614	lincRNA	RP3-340I3.1
RNA33834|lncRNAdb_41_1441	0.009166346	0.002746	2.490655	up	ENST00000397381	lincRNA	UCA1
RNA38048|ENCODE_2921_204	0.002004868	0.000331	2.492889	up	ENST00000430058	lincRNA	RP11-23B15.1
RNA44573|UCSC_2554_2833	0.00473077	0.00109	2.493999	up	ENST00000432163	lincRNA	AC098973.1

RNA39448|RefSeq_1428_2265	0.001308428	0.000183	2.497945	up	ENST00000581549	lincRNA	LINC00511
RNA37686|ENCODE_2558_369	0.006875952	0.001837	2.501507	up	ENST00000439483	lincRNA	AP000770.1
RNA35677|ENCODE_545_1017	0.001817163	0.000289	2.508267	up	ENST00000437648	lincRNA	RP11-146I2.1
RNA48284|UCSC_7142_1500	0.003372822	0.000682	2.509532	up	ENST00000510694	lincRNA	RP11-438N16.1
RNA37604|ENCODE_2476_385	0.001252139	0.000172	2.520982	up	ENST00000419064	lincRNA	RP11-13P5.2
RNA36026|ENCODE_896_762	0.005963034	0.001507	2.522171	up	ENST00000449069	lincRNA	RP1-97J1.2
RNA35971|ENCODE_841_786	0.000919723	0.000113	2.527082	up	ENST00000438867	lincRNA	RP11-157D23.2
RNA44597|UCSC_2584_2815	0.000441772	4.16E-05	2.528342	up	ENST00000517491	lincRNA	AC145110.1
RNA46564|UCSC_5061_1975	0.003013015	0.000584	2.534044	up	ENST00000550678	lincRNA	RP11-767I20.1
RNA38018|ENCODE_2891_232	0.020448523	0.00809	2.534808	up	ENST00000455092	lincRNA	AC013248.2
RNA35606|ENCODE_474_1210	0.013214486	0.004526	2.540476	up	ENST00000428781	lincRNA	RP4-758J18.9
RNA35599|ENCODE_467_1234	0.011300267	0.003651	2.550854	up	ENST00000413763	lincRNA	RP11-212D3.4
RNA41020|RefSeq_3127_667	0.002766511	0.000518	2.551824	up	ENST00000573037	lincRNA	BCAR4
RNA36222|ENCODE_1092_684	0.004081452	0.000888	2.552951	up	ENST00000440735	lincRNA	RP11-365P13.3
RNA36856|ENCODE_1726_517	0.002897906	0.000553	2.596925	up	ENST00000438499	lincRNA	RP11-1M18.1
RNA33983|NRED_113_3736	0.005931724	0.001494	2.598334	up	ENST00000547179	lincRNA	LINC00485
RNA35410|ENCODE_273_1977	0.006152971	0.001575	2.607011	up	ENST00000445865	lincRNA	AC007880.1
RNA35643|ENCODE_511_1113	0.025159651	0.01063	2.615943	up	ENST00000423943	lincRNA	RP11-48O20.4
RNA51299|UCSC_10795_575	0.0003136	2.6E-05	2.617738	up	ENST00000549065	lincRNA	CTD-2314B22.3
RNA37109|ENCODE_1979_471	0.008944778	0.002654	2.621466	up	ENST00000457239	lincRNA	RP11-71G12.1
RNA47609|UCSC_6325_1687	0.002422908	0.00043	2.624382	up	ENST00000502427	lincRNA	RP11-774D14.1
RNA35741|ENCODE_610_929	0.003193576	0.000633	2.629012	up	ENST00000447557	lincRNA	RP1-287H17.1
RNA36932|ENCODE_1802_501	0.004744864	0.001094	2.637663	up	ENST00000435444	lincRNA	RP11-29B9.2
RNA50845|UCSC_10234_736	0.010446692	0.003283	2.660078	up	ENST00000549905	lincRNA	RP11-81H3.2
RNA38807|RefSeq_748_3242	0.046659708	0.023411	2.670234	up	ENST00000448106	lincRNA	AC009232.2
RNA38362|RefSeq_277_4745	0.020517522	0.008126	2.679299	up	ENST00000445791	lincRNA	AC074093.1
RNA35197|ENCODE_52_3503	0.004894004	0.001143	2.680775	up	ENST00000418420	lincRNA	LINC00276
RNA35265|ENCODE_123_2663	0.000896742	0.000109	2.685656	up	ENST00000412134	lincRNA	AC022311.1
RNA36905|ENCODE_1775_507	0.001792497	0.000283	2.688483	up	ENST00000445974	lincRNA	RP11-437J19.1
RNA51310|UCSC_10810_570	0.000191563	1.34E-05	2.696167	up	ENST00000549065	lincRNA	CTD-2314B22.3
RNA37448|ENCODE_2319_416	0.000743176	8.45E-05	2.704547	up	ENST00000419347	lincRNA	AC019118.3
RNA38051|ENCODE_2924_201	0.008952729	0.002658	2.775476	up	ENST00000421181	lincRNA	AC080094.1
RNA51691|UCSC_11277_423	0.001303527	0.000182	2.779668	up	ENST00000443364	lincRNA	RP11-48O20.4
RNA36155|ENCODE_1025_713	0.01717655	0.006421	2.787663	up	ENST00000446028	lincRNA	RP11-431N15.2
RNA42543|UCSC_98_7863	4.23293E-05	1.63E-06	2.791871	up	ENST00000567749	lincRNA	RP13-735L24.1
RNA36093|ENCODE_963_737	0.002228509	0.000383	2.803112	up	ENST00000411615	lincRNA	RP11-356I2.1

RNA50882|UCSC_10279_723	0.030511819	0.013632	2.822593	up	ENST00000413842	lincRNA	AC010148.1
RNA35464|ENCODE_328_1796	0.00742969	0.002047	2.837801	up	ENST00000439156	lincRNA	RP11-776H12.1
RNA36162|ENCODE_1032_711	0.001116173	0.000147	2.83816	up	ENST00000437608	lincRNA	AC093838.7
RNA35775|ENCODE_644_900	0.02576153	0.010952	2.854549	up	ENST00000433310	lincRNA	AF131217.1
RNA35404|ENCODE_267_1999	0.010877577	0.00347	2.867945	up	ENST00000413042	lincRNA	AC005082.12
RNA37904|ENCODE_2777_302	0.012957163	0.004399	2.874613	up	ENST00000458375	lincRNA	XXbac-BPG254F23.
RNA34694|NRED_826_1613	0.000458387	4.37E-05	2.875394	up	ENST00000546186	lincRNA	CTD-2335A18.1
RNA36500|ENCODE_1370_589	0.002233339	0.000384	2.879619	up	ENST00000423489	lincRNA	RP3-359N14.2
RNA37315|ENCODE_2186_437	0.003290753	0.00066	2.880301	up	ENST00000444868	lincRNA	AP000473.6
RNA35462|ENCODE_326_1801	0.001529588	0.000228	2.88391	up	ENST00000441851	lincRNA	RP5-968D22.1
RNA48517|UCSC_7418_1426	0.002614745	0.000478	2.898036	up	ENST00000562167	lincRNA	AC011525.2
RNA43669|UCSC_1456_3590	0.001710589	0.000266	2.902631	up	ENST00000482787	lincRNA	RP11-416O18.1
RNA36110|ENCODE_980_731	0.000645019	6.95E-05	2.904442	up	ENST00000428276	lincRNA	RP11-175B12.2
RNA36228|ENCODE_1098_683	0.021271478	0.008522	2.926097	up	ENST00000433310	lincRNA	AF131217.1
RNA36270|ENCODE_1140_666	0.009207921	0.002763	2.944018	up	ENST00000425048	lincRNA	RP11-114G1.2
RNA37648|ENCODE_2520_377	0.000496828	4.88E-05	2.955786	up	ENST00000430064	lincRNA	AF212831.2
RNA36198|ENCODE_1068_694	0.000792101	9.21E-05	2.97923	up	ENST00000427017	lincRNA	RP1-182D15.2
RNA48055|UCSC_6856_1569	0.005243194	0.001261	2.980372	up	ENST00000417483	lincRNA	RP11-557H15.3
RNA45363|UCSC_3552_2391	0.00043177	4.04E-05	2.986949	up	ENST00000455088	lincRNA	CTD-2314B22.3
RNA41206|RefSeq_3334_461	0.006909705	0.00185	2.9941	up	ENST00000447175	lincRNA	CHODL-AS1
RNA35649|ENCODE_517_1096	0.000507547	5.02E-05	2.996129	up	ENST00000426539	lincRNA	AC130710.1
RNA36816|ENCODE_1686_526	0.004554475	0.001034	3.004286	up	ENST00000411542	lincRNA	AC011288.2
RNA48920|UCSC_7911_1298	0.000385246	3.44E-05	3.012949	up	ENST00000411439	lincRNA	AL022344.4
RNA44403|UCSC_2353_2941	0.00190734	0.000309	3.035171	up	ENST00000564508	lincRNA	AC004158.3
RNA45351|UCSC_3536_2395	0.00037608	3.33E-05	3.057658	up	ENST00000455088	lincRNA	CTD-2314B22.3
RNA36147|ENCODE_1017_717	0.001120453	0.000148	3.061645	up	ENST00000421581	lincRNA	AC016768.1
RNA34683|NRED_815_1629	0.008474144	0.002464	3.071659	up	ENST00000545400	lincRNA	RP11-153K16.1
RNA37752|ENCODE_2624_349	0.007070745	0.001911	3.10821	up	ENST00000437057	lincRNA	RP11-408E5.5
RNA35294|ENCODE_153_2490	0.002190779	0.000374	3.113139	up	ENST00000457009	lincRNA	RP4-568F9.6
RNA47413|UCSC_6067_1745	0.002985886	0.000576	3.122505	up	ENST00000428651	lincRNA	AC093375.1
RNA34970|NRED_1111_987	0.00658227	0.001729	3.12592	up	ENST00000551726	lincRNA	RP11-81H3.2
RNA46618|UCSC_5122_1962	0.005577783	0.00137	3.128816	up	ENST00000500092	lincRNA	RP11-519M16.1
RNA37337|ENCODE_2208_435	0.000637452	6.84E-05	3.155659	up	ENST00000454530	lincRNA	AC019118.4
RNA37530|ENCODE_2402_400	0.024359195	0.010183	3.172138	up	ENST00000437057	lincRNA	RP11-408E5.5
RNA34696|NRED_828_1611	0.000247363	1.89E-05	3.181129	up	ENST00000546186	lincRNA	CTD-2335A18.1
RNA36849|ENCODE_1719_519	0.000188731	1.31E-05	3.246597	up	ENST00000422253	lincRNA	RP5-968D22.3

RNA50470|UCSC_9767_871	0.021860078	0.008836	3.260084	up	ENST00000515218	lincRNA	RP11-129M6.1
RNA35898|ENCODE_768_817	0.000225203	1.66E-05	3.261333	up	ENST00000433664	lincRNA	RP11-501G6.1
RNA38017|ENCODE_2890_236	0.002838457	0.000537	3.278359	up	ENST00000443030	lincRNA	AC092168.4
RNA46537|UCSC_5025_1984	0.004773925	0.001104	3.305291	up	ENST00000417483	lincRNA	RP11-557H15.3
RNA36352|ENCODE_1222_632	0.016050389	0.005873	3.308145	up	ENST00000419801	lincRNA	RP1-128O3.4
RNA36791|ENCODE_1661_532	0.002775292	0.00052	3.317079	up	ENST00000453395	lincRNA	LA16c-83F12.6
RNA36174|ENCODE_1044_708	0.00019169	1.34E-05	3.342069	up	ENST00000432697	lincRNA	LINC00358
RNA36317|ENCODE_1187_647	0.000175753	1.2E-05	3.371626	up	ENST00000439703	lincRNA	RP11-417E7.1
RNA52128|UCSC_11757_206	0.005456667	0.00133	3.385955	up	ENST00000529451	lincRNA	RP11-780O24.1
RNA36379|ENCODE_1249_624	0.000396577	3.59E-05	3.396615	up	ENST00000453395	lincRNA	LA16c-83F12.6
RNA54342|H-InvDB_1652_339	0.000507173	5.01E-05	3.429104	up	ENST00000551545	lincRNA	LINC00516
RNA36240|ENCODE_1110_678	0.006933452	0.001859	3.431524	up	ENST00000432481	lincRNA	AC009410.1
RNA35167|ENCODE_21_4486	0.001570271	0.000237	3.445265	up	ENST00000453420	lincRNA	AL035610.2
RNA49402|UCSC_8478_1165	0.016240783	0.005965	3.463096	up	ENST00000563172	lincRNA	CTD-2354A18.1
RNA58740|asoverlaps_297_2128	0.000157898	1.04E-05	3.527871	up	ENST00000566070	lincRNA	RP11-426C22.4
RNA37078|ENCODE_1948_475	1.37066E-05	3.21E-07	3.575229	up	ENST00000415205	lincRNA	Z83851.3
RNA35196|ENCODE_51_3516	0.000668361	7.3E-05	3.642797	up	ENST00000431979	lincRNA	AC093838.7
RNA35594|ENCODE_462_1251	0.001422235	0.000205	3.709873	up	ENST00000435106	lincRNA	RP11-271F18.2
RNA35990|ENCODE_860_777	0.00649008	0.001695	3.729768	up	ENST00000438372	lincRNA	RP11-69C17.1
RNA37847|ENCODE_2719_322	0.004227018	0.000933	3.739784	up	ENST00000440802	lincRNA	AC097532.2
RNA35768|ENCODE_637_905	0.000960771	0.00012	3.839575	up	ENST00000449673	lincRNA	AC092669.6
RNA37806|ENCODE_2678_337	0.003620269	0.000752	3.954108	up	ENST00000428415	lincRNA	AP000344.3
RNA39845|RefSeq_1855_1875	0.002673411	0.000494	3.95524	up	ENST00000521663	lincRNA	LINC00588
RNA35772|ENCODE_641_901	0.000851171	0.000101	3.976286	up	ENST00000436710	lincRNA	RP11-65J3.1
RNA37988|ENCODE_2861_260	0.001037215	0.000133	3.983699	up	ENST00000435151	lincRNA	RP11-46B11.4
RNA44456|UCSC_2420_2904	0.002283902	0.000396	4.005827	up	ENST00000482787	lincRNA	RP11-416O18.1
RNA37014|ENCODE_1884_485	0.006694566	0.001768	4.187201	up	ENST00000452852	lincRNA	LINC00393
RNA36468|ENCODE_1338_597	0.016367937	0.006028	4.413157	up	ENST00000415883	lincRNA	RP11-346D19.1
RNA35924|ENCODE_794_804	0.00155146	0.000232	4.462955	up	ENST00000443364	lincRNA	RP11-48O20.4
RNA36000|ENCODE_870_774	0.001808839	0.000288	4.526314	up	ENST00000437790	lincRNA	AC092669.2
RNA35648|ENCODE_516_1103	0.000299555	2.44E-05	4.544315	up	ENST00000439259	lincRNA	AC012494.1
RNA34974|NRED_1115_979	0.001058673	0.000137	4.551831	up	ENST00000420691	lincRNA	RP1-10C16.2
RNA35166|ENCODE_20_4604	0.003194553	0.000633	4.656465	up	ENST00000449316	lincRNA	RP4-760C5.5
RNA37143|ENCODE_2013_465	9.76672E-05	5.52E-06	4.676756	up	ENST00000452675	lincRNA	RP11-367G18.1
RNA36301|ENCODE_1171_655	0.001396141	0.0002	4.719087	up	ENST00000418764	lincRNA	AC004543.2
RNA37299|ENCODE_2170_439	0.003235599	0.000644	4.781088	up	ENST00000439450	lincRNA	RP5-1097P24.1

RNA35031|NRED_1172_839	0.000448028	4.24E-05	4.792342	up	ENST00000420691	lincRNA	RP1-10C16.2
RNA34973|NRED_1114_981	0.000718314	8.08E-05	4.83718	up	ENST00000420691	lincRNA	RP1-10C16.2
RNA35580|ENCODE_448_1331	4.57143E-05	1.8E-06	4.848393	up	ENST00000418078	lincRNA	RP11-262K1.1
RNA48654|UCSC_7583_1386	3.87244E-05	1.43E-06	5.18079	up	ENST00000551881	lincRNA	AL589743.1
RNA48658|UCSC_7588_1385	3.13827E-05	1.05E-06	5.398819	up	ENST00000551881	lincRNA	AL589743.1
RNA43257|UCSC_962_4142	2.95532E-05	9.71E-07	5.405892	up	ENST00000552602	lincRNA	CTD-2314B22.3
RNA46190|UCSC_4583_2088	0.000170844	1.15E-05	5.540492	up	ENST00000547963	lincRNA	RP11-438N16.1
RNA54569|H-InvDB_1894_318	0.000173846	1.18E-05	5.694105	up	ENST00000445613	lincRNA	AC104088.1
RNA43032|UCSC_692_4651	2.18818E-05	6.37E-07	6.176914	up	ENST00000552602	lincRNA	CTD-2314B22.3
RNA48659|UCSC_7589_1385	3.62666E-05	1.3E-06	6.616329	up	ENST00000548057	lincRNA	CTD-2314B22.3
RNA48226|UCSC_7075_1516	5.32621E-05	2.29E-06	6.745658	up	ENST00000522365	lincRNA	RP11-51M18.1
RNA36944|ENCODE_1814_499	0.000565215	5.8E-05	7.395793	up	ENST00000445976	lincRNA	RP4-660H19.1
RNA37834|ENCODE_2706_327	0.000147953	9.56E-06	7.64244	up	ENST00000427157	lincRNA	RP11-367G18.1
RNA34315|NRED_446_2251	9.33604E-05	5.17E-06	7.802774	up	ENST00000457958	lincRNA	LINC00511
RNA34459|NRED_591_1986	2.91297E-05	9.51E-07	7.823907	up	ENST00000457958	lincRNA	LINC00511
RNA34616|NRED_748_1731	5.08797E-05	2.13E-06	8.868822	up	ENST00000457958	lincRNA	LINC00511
RNA34647|NRED_779_1681	4.12836E-05	1.57E-06	9.075793	up	ENST00000457958	lincRNA	LINC00511
RNA43730|UCSC_1530_3527	0.000155514	1.02E-05	9.653973	up	ENST00000557399	lincRNA	RP11-572M18.1
RNA36892|ENCODE_1762_510	0.000374855	3.31E-05	10.1267	up	ENST00000446484	lincRNA	AC009262.2
RNA49481|UCSC_8570_1142	0.00039273	3.54E-05	13.76971	up	ENST00000523313	lincRNA	RP11-697M17.1
RNA35041|NRED_1182_805	4.4714E-06	6.07E-08	2.078151	up	ENST00000537869	lincRNA	SNHG1
RNA45833|UCSC_4153_2210	0.013457338	0.004639	2.112504	up	ENST00000519741	lincRNA	RP11-32K4.2
RNA34814|NRED_947_1395	1.46347E-05	3.54E-07	2.137111	up	ENST00000537869	lincRNA	SNHG1
RNA34813|NRED_946_1396	1.86107E-05	5.07E-07	2.139897	up	ENST00000537869	lincRNA	SNHG1
RNA33838|lncRNAdb_45_1134	1.51034E-05	3.71E-07	2.191032	up	ENST00000537869	lincRNA	SNHG1
RNA44970|UCSC_3049_2582	0.03050145	0.013625	2.222763	up	ENST00000582581	lincRNA	CTC-525D6.1
RNA34983|NRED_1124_955	1.35077E-05	3.14E-07	2.256162	up	ENST00000539975	lincRNA	SNHG1
RNA40615|RefSeq_2685_1105	0.002199247	0.000376	2.461126	up	ENST00000520799	lincRNA	RP11-32K4.2
RNA175132|ENST00000314629	0.019694602	0.007702	2.000359	up	ENST00000422540	processed_transcript	LINC00521
RNA176860|ENST00000428411	0.002282611	0.000395	2.004391	up	ENST00000428411	processed_transcript	RP11-31F15.1
RNA175882|ENST00000458525	0.011493139	0.003733	2.005602	up	ENST00000458525	processed_transcript	RP1-274L7.1
RNA178442|ENST00000446867	0.003979585	0.000858	2.005907	up	ENST00000536718	processed_transcript	TMEM191C
RNA177328|ENST00000502049	0.045681406	0.022806	2.010813	up	ENST00000502049	processed_transcript	RP11-411D10.1
RNA178210|ENST00000449469	4.78232E-05	1.94E-06	2.012517	up	ENST00000449469	processed_transcript	RP4-564F22.2
RNA178271|ENST00000455642	0.008796222	0.002594	2.01297	up	ENST00000455642	processed_transcript	RP5-881L22.6
RNA178204|ENST00000423536	0.000155514	1.02E-05	2.014577	up	ENST00000436764	processed_transcript	RP4-564F22.2

RNA178640|ENST00000445096	0.013594779	0.004707	2.023204	up	ENST00000545531	processed_transcript	SLC38A3
RNA176261|ENST00000509405	0.040508594	0.019578	2.030496	up	ENST00000509405	processed_transcript	LINC00461
RNA178520|ENST00000423600	0.025287023	0.010696	2.041516	up	ENST00000423600	processed_transcript	AC069513.4
RNA178539|ENST00000523427	0.00285611	0.000542	2.043949	up	ENST00000523427	processed_transcript	PVT1
RNA177134|ENST00000500527	0.001773307	0.000279	2.053677	up	ENST00000500527	processed_transcript	RP11-551L14.5
RNA177809|ENST00000539969	0.000862437	0.000103	2.059565	up	ENST00000540774	processed_transcript	RP5-944M2.3
RNA175889|ENST00000445415	0.004100651	0.000894	2.060896	up	ENST00000445415	processed_transcript	AC004383.4
RNA178642|ENST00000445325	0.007837222	0.002211	2.062366	up	ENST00000445325	processed_transcript	SLC38A3
RNA174241|ENST00000538380	0.007151746	0.001941	2.068288	up	ENST00000538380	processed_transcript	AC091878.1
RNA175804|ENST00000504833	7.516E-05	3.79E-06	2.082502	up	ENST00000504833	processed_transcript	CTD-2001C12.1
RNA175951|ENST00000444958	0.00018762	1.3E-05	2.085824	up	ENST00000444958	processed_transcript	DANCR
RNA174238|ENST00000509037	0.04177443	0.020358	2.08827	up	ENST00000509037	processed_transcript	RP11-19O2.2
RNA177970|ENST00000483650	0.008561386	0.002501	2.088362	up	ENST00000483650	processed_transcript	RP11-548O1.3
RNA175988|ENST00000439875	0.005342681	0.001292	2.091908	up	ENST00000439875	processed_transcript	LINC00256B
RNA178297|ENST00000422059	0.011891665	0.003912	2.101766	up	ENST00000422059	processed_transcript	RP5-1120P11.1
RNA178644|ENST00000545531	0.011932188	0.003931	2.108141	up	ENST00000545531	processed_transcript	SLC38A3
RNA178477|ENST00000314140	0.021725642	0.008761	2.115915	up	ENST00000314140	processed_transcript	GPHB5
RNA177541|ENST00000532831	0.003579942	0.00074	2.116129	up	ENST00000527321	processed_transcript	RP11-705O3.1
RNA176037|ENST00000445737	0.003255952	0.000651	2.125298	up	ENST00000445737	processed_transcript	UBAC2-AS1
RNA175654|ENST00000511465	0.011325575	0.003662	2.131627	up	ENST00000511465	processed_transcript	RP11-290F5.2
RNA178242|ENST00000437991	0.015494599	0.005601	2.140463	up	ENST00000437991	processed_transcript	AC005041.9
RNA175080|ENST00000505000	0.00977399	0.002999	2.146504	up	ENST00000505000	processed_transcript	RP11-673E1.1
RNA175994|ENST00000510576	0.03714114	0.017562	2.164593	up	ENST00000510576	processed_transcript	CTB-113P19.1
RNA178674|ENST00000414529	0.001424149	0.000206	2.16825	up	ENST00000414529	processed_transcript	AC092937.2
RNA175468|ENST00000515403	0.043056954	0.021175	2.169567	up	ENST00000513919	processed_transcript	RP11-844P9.2
RNA178152|ENST00000421891	0.009356062	0.002827	2.175765	up	ENST00000421891	processed_transcript	RP3-399L15.3
RNA177064|ENST00000419143	0.00463294	0.001059	2.184506	up	ENST00000419143	processed_transcript	MIR205HG
RNA178323|ENST00000419168	0.01009318	0.003137	2.187233	up	ENST00000419168	processed_transcript	RP11-715G15.1
RNA178389|ENST00000534540	0.003653742	0.000761	2.187544	up	ENST00000534540	processed_transcript	MIR210HG
RNA178549|ENST00000512617	0.017097577	0.006381	2.187595	up	ENST00000521600	processed_transcript	PVT1
RNA177135|ENST00000535870	0.001746062	0.000273	2.188412	up	ENST00000535870	processed_transcript	RP11-551L14.5
RNA175131|ENST00000449472	0.011574701	0.00377	2.19483	up	ENST00000422540	processed_transcript	LINC00521
RNA178550|ENST00000521600	0.002902547	0.000554	2.205471	up	ENST00000521600	processed_transcript	PVT1
RNA178488|ENST00000420947	0.003921143	0.000841	2.212494	up	ENST00000420947	processed_transcript	RP11-407B7.1
RNA175414|ENST00000452184	6.07412E-05	2.79E-06	2.21921	up	ENST00000452184	processed_transcript	RP11-262H14.1
RNA178154|ENST00000519104	0.014797956	0.005264	2.233984	up	ENST00000519104	processed_transcript	RP3-399L15.3

RNA178450|ENST00000517864	0.000296297	2.4E-05	2.264845	up	ENST00000517864	processed_transcript	RP11-320N21.1
RNA174235|ENST00000505877	0.012679078	0.004269	2.269749	up	ENST00000505877	processed_transcript	RP11-308B16.1
RNA177921|ENST00000423737	0.001226779	0.000167	2.272996	up	ENST00000423737	processed_transcript	RP11-191L9.4
RNA177887|ENST00000540739	0.013943516	0.004868	2.276473	up	ENST00000540739	processed_transcript	RP11-669N7.2
RNA176035|ENST00000526458	0.019074962	0.007371	2.276703	up	ENST00000526458	processed_transcript	RP11-109D9.3
RNA176052|ENST00000500538	0.008294994	0.002391	2.288246	up	ENST00000500538	processed_transcript	RP11-453E17.1
RNA177889|ENST00000360083	0.005271871	0.001269	2.30775	up	ENST00000360083	processed_transcript	AC079610.2
RNA178637|ENST00000417851	0.006011261	0.001523	2.329267	up	ENST00000541861	processed_transcript	SLC38A3
RNA178090|ENST00000526611	0.001796109	0.000284	2.336679	up	ENST00000526611	processed_transcript	RP1-179N16.6
RNA178636|ENST00000417121	0.006498975	0.001699	2.343544	up	ENST00000545531	processed_transcript	SLC38A3
RNA178542|ENST00000518528	0.000528863	5.3E-05	2.356038	up	ENST00000518528	processed_transcript	PVT1
RNA177566|ENST00000548760	0.006682061	0.001763	2.365173	up	ENST00000548760	processed_transcript	RP11-181C3.2
RNA177923|ENST00000438810	0.008717631	0.002563	2.368295	up	ENST00000438810	processed_transcript	RP11-191L9.4
RNA178132|ENST00000360785	0.006985897	0.001879	2.386565	up	ENST00000360785	processed_transcript	C20orf203
RNA178600|ENST00000453023	0.001027126	0.000132	2.388585	up	ENST00000453023	processed_transcript	MIAT
RNA178673|ENST00000430666	0.003163063	0.000624	2.392815	up	ENST00000430666	processed_transcript	AC092937.2
RNA175838|ENST00000430140	0.010825771	0.003447	2.395836	up	ENST00000430140	processed_transcript	RP6-191P20.4
RNA177534|ENST00000493116	0.000653949	7.08E-05	2.414071	up	ENST00000493116	processed_transcript	SOX2-OT
RNA178591|ENST00000422403	0.000108773	6.37E-06	2.449142	up	ENST00000453023	processed_transcript	MIAT
RNA177533|ENST00000490005	0.004092242	0.000891	2.454133	up	ENST00000490005	processed_transcript	SOX2-OT
RNA175822|ENST00000530194	0.006980033	0.001877	2.464723	up	ENST00000530194	processed_transcript	RP11-159H10.3
RNA178543|ENST00000523068	0.000898734	0.000109	2.479013	up	ENST00000523068	processed_transcript	PVT1
RNA178538|ENST00000517525	0.002674671	0.000494	2.484597	up	ENST00000517525	processed_transcript	PVT1
RNA175383|ENST00000429250	0.002750046	0.000513	2.490778	up	ENST00000429250	processed_transcript	RP11-90J7.3
RNA178586|ENST00000440347	0.002551169	0.000462	2.492781	up	ENST00000453023	processed_transcript	MIAT
RNA178545|ENST00000513868	0.000372691	3.28E-05	2.514839	up	ENST00000522414	processed_transcript	PVT1
RNA177413|ENST00000437488	0.006449943	0.00168	2.520784	up	ENST00000437488	processed_transcript	RP11-385J1.2
RNA177006|ENST00000436905	0.000715861	8.04E-05	2.523071	up	ENST00000436905	processed_transcript	RP11-547I7.2
RNA178599|ENST00000449717	0.003594044	0.000744	2.524181	up	ENST00000453023	processed_transcript	MIAT
RNA178535|ENST00000524165	0.000387051	3.46E-05	2.527659	up	ENST00000522963	processed_transcript	PVT1
RNA178155|ENST00000520554	0.001036614	0.000133	2.539055	up	ENST00000520554	processed_transcript	RP3-399L15.3
RNA175844|ENST00000436013	0.01273258	0.004294	2.556378	up	ENST00000436013	processed_transcript	RP6-24A23.3
RNA174258|ENST00000458666	0.011714199	0.003833	2.560203	up	ENST00000458666	processed_transcript	AC087859.1
RNA178598|ENST00000419237	0.001014679	0.000129	2.615326	up	ENST00000419237	processed_transcript	MIAT
RNA177642|ENST00000435328	0.005720594	0.001419	2.659019	up	ENST00000435328	processed_transcript	AC018712.3
RNA178593|ENST00000451141	0.00136853	0.000195	2.684138	up	ENST00000419237	processed_transcript	MIAT

RNA176656|ENST00000458151	0.000400348	3.64E-05	2.685751	up	ENST00000458151	processed_transcript	RP5-1024G6.5
RNA175703|ENST00000508156	0.014634989	0.00519	2.696647	up	ENST00000508156	processed_transcript	AF146191.4
RNA175375|ENST00000473776	0.002305041	0.000401	2.738583	up	ENST00000473776	processed_transcript	RP5-894A10.5
RNA178594|ENST00000455640	0.000659539	7.16E-05	2.769644	up	ENST00000419237	processed_transcript	MIAT
RNA178014|ENST00000437384	0.01482051	0.005275	2.780825	up	ENST00000437384	processed_transcript	RP11-77C3.3
RNA178536|ENST00000523328	0.00015379	1E-05	2.795964	up	ENST00000522963	processed_transcript	PVT1
RNA178584|ENST00000421867	0.001058627	0.000137	2.802511	up	ENST00000419237	processed_transcript	MIAT
RNA178595|ENST00000452429	0.001700181	0.000264	2.817721	up	ENST00000419237	processed_transcript	MIAT
RNA175469|ENST00000513919	7.29745E-05	3.63E-06	2.822789	up	ENST00000513919	processed_transcript	RP11-844P9.2
RNA178537|ENST00000521951	0.00037504	3.31E-05	2.832749	up	ENST00000521951	processed_transcript	PVT1
RNA177230|ENST00000453562	0.001382044	0.000197	2.900608	up	ENST00000453562	processed_transcript	AC003102.3
RNA177387|ENST00000526617	0.004600422	0.001048	2.922115	up	ENST00000526617	processed_transcript	RP11-540A21.3
RNA177005|ENST00000424735	0.000477909	4.62E-05	2.928451	up	ENST00000436905	processed_transcript	RP11-547I7.2
RNA175053|ENST00000419766	0.010067642	0.003125	2.946589	up	ENST00000419766	processed_transcript	NPSR1-AS1
RNA178540|ENST00000517790	0.002125902	0.000359	2.955174	up	ENST00000517790	processed_transcript	PVT1
RNA178588|ENST00000430483	0.002968672	0.000571	2.983526	up	ENST00000419237	processed_transcript	MIAT
RNA175489|ENST00000427794	0.000422583	3.92E-05	3.049327	up	ENST00000427794	processed_transcript	RP5-907D15.2
RNA178589|ENST00000439738	0.000361415	3.15E-05	3.058817	up	ENST00000419237	processed_transcript	MIAT
RNA178585|ENST00000418918	0.000236812	1.78E-05	3.107092	up	ENST00000453023	processed_transcript	MIAT
RNA178546|ENST00000520913	0.001116271	0.000147	3.135526	up	ENST00000517838	processed_transcript	PVT1
RNA175060|ENST00000537560	0.00253277	0.000457	3.181711	up	ENST00000537560	processed_transcript	NPSR1-AS1
RNA178534|ENST00000504719	0.000966011	0.000121	3.267851	up	ENST00000517838	processed_transcript	PVT1
RNA178596|ENST00000413665	0.000487933	4.75E-05	3.281546	up	ENST00000430080	processed_transcript	MIAT
RNA178590|ENST00000436238	7.38552E-05	3.7E-06	3.311622	up	ENST00000430080	processed_transcript	MIAT
RNA178592|ENST00000425476	0.000496406	4.87E-05	3.34446	up	ENST00000419237	processed_transcript	MIAT
RNA178544|ENST00000521122	7.27544E-05	3.61E-06	3.374777	up	ENST00000520913	processed_transcript	PVT1
RNA178597|ENST00000458302	0.000348532	3E-05	3.457694	up	ENST00000419237	processed_transcript	MIAT
RNA174250|ENST00000414438	0.00610252	0.001557	3.472349	up	ENST00000414438	processed_transcript	AC069277.2
RNA175183|ENST00000456880	0.002351794	0.000412	3.505526	up	ENST00000456880	processed_transcript	AP001065.2
RNA178548|ENST00000517838	0.000856589	0.000102	3.534055	up	ENST00000517838	processed_transcript	PVT1
RNA175036|ENST00000452148	6.04612E-05	2.77E-06	3.561887	up	ENST00000452148	processed_transcript	RP11-342C23.4
RNA177414|ENST00000451742	0.001559375	0.000234	3.603136	up	ENST00000432385	processed_transcript	RP11-385J1.2
RNA178587|ENST00000450203	0.000306515	2.52E-05	3.628078	up	ENST00000419237	processed_transcript	MIAT
RNA178553|ENST00000522414	0.000100597	5.75E-06	3.723149	up	ENST00000522414	processed_transcript	PVT1
RNA175018|ENST00000510016	0.002030891	0.000337	3.823304	up	ENST00000510016	processed_transcript	RP11-8L2.1
RNA175061|ENST00000535640	0.001356603	0.000193	3.8551	up	ENST00000442669	processed_transcript	NPSR1-AS1

RNA175062|ENST00000544556	0.000981622	0.000123	3.942585	up	ENST00000442669	processed_transcript	NPSR1-AS1
RNA178547|ENST00000519481	0.000280678	2.24E-05	3.954472	up	ENST00000519481	processed_transcript	PVT1
RNA178552|ENST00000523190	0.000328595	2.77E-05	3.993547	up	ENST00000523190	processed_transcript	PVT1
RNA178551|ENST00000522875	9.71261E-05	5.47E-06	4.302713	up	ENST00000522875	processed_transcript	PVT1
RNA175059|ENST00000539747	0.000978219	0.000123	4.343778	up	ENST00000537560	processed_transcript	NPSR1-AS1
RNA174251|ENST00000414603	0.002714525	0.000504	4.376468	up	ENST00000414603	processed_transcript	AC069277.2
RNA176105|ENST00000534398	7.25349E-05	3.6E-06	4.400547	up	ENST00000435097	processed_transcript	RP11-429J17.6
RNA176106|ENST00000435097	5.97062E-05	2.72E-06	4.573842	up	ENST00000435097	processed_transcript	RP11-429J17.6
RNA177520|ENST00000460739	0.00253482	0.000458	5.036425	up	ENST00000460739	processed_transcript	SOX2-OT
RNA176030|ENST00000438290	0.005477035	0.001336	5.305142	up	ENST00000438290	processed_transcript	SOX21-AS1
RNA177418|ENST00000527945	0.000134554	8.43E-06	5.360729	up	ENST00000527945	processed_transcript	CTC-497E21.4
RNA175016|ENST00000508406	9.04011E-05	4.94E-06	5.517909	up	ENST00000508406	processed_transcript	RP11-8L2.1
RNA177417|ENST00000432385	0.000318645	2.65E-05	6.058812	up	ENST00000432385	processed_transcript	RP11-385J1.2
RNA177524|ENST00000498731	0.001283478	0.000178	6.47473	up	ENST00000498731	processed_transcript	SOX2-OT
RNA175828|ENST00000500779	0.003299848	0.000663	7.614603	up	ENST00000500779	processed_transcript	CTC-426B10.1
RNA177525|ENST00000492337	0.001196795	0.000161	8.102027	up	ENST00000492337	processed_transcript	SOX2-OT
RNA177061|ENST00000451937	2.08528E-05	5.94E-07	8.522191	up	ENST00000366437	processed_transcript	MIR205HG
RNA177065|ENST00000458250	1.6513E-05	4.26E-07	8.973103	up	ENST00000458250	processed_transcript	MIR205HG
RNA177424|ENST00000526388	3.85993E-05	1.42E-06	9.222317	up	ENST00000526388	processed_transcript	CTC-497E21.4
RNA176010|ENST00000506795	0.005121219	0.001219	9.270447	up	ENST00000450161	processed_transcript	RPL34-AS1
RNA177422|ENST00000529328	3.30939E-05	1.14E-06	10.24391	up	ENST00000526388	processed_transcript	CTC-497E21.4
RNA177423|ENST00000531402	4.75265E-05	1.92E-06	10.53625	up	ENST00000526388	processed_transcript	CTC-497E21.4
RNA177421|ENST00000534477	3.9579E-05	1.47E-06	12.28134	up	ENST00000534477	processed_transcript	CTC-497E21.4
RNA177063|ENST00000444286	0.000230016	1.71E-05	13.99112	up	ENST00000419143	processed_transcript	MIR205HG
RNA177060|ENST00000429156	0.000260847	2.03E-05	14.55737	up	ENST00000366437	processed_transcript	MIR205HG
RNA177067|ENST00000366437	0.000245315	1.87E-05	14.63077	up	ENST00000366437	processed_transcript	MIR205HG
RNA177062|ENST00000431096	0.000151006	9.82E-06	14.74743	up	ENST00000366437	processed_transcript	MIR205HG
RNA177066|ENST00000440276	0.000137511	8.7E-06	14.89794	up	ENST00000366437	processed_transcript	MIR205HG
RNA177420|ENST00000532541	0.00011033	6.49E-06	15.04321	up	ENST00000532541	processed_transcript	CTC-497E21.4
RNA177059|ENST00000433108	0.00053578	5.39E-05	22.97576	up	ENST00000433108	processed_transcript	MIR205HG
RNA178455|ENST00000393515	0.016509887	0.0061	2.03192	up	ENST00000393515	processed_transcript	ANKRD36BP2
RNA178453|ENST00000393525	0.007529572	0.002088	2.30576	up	ENST00000393525	processed_transcript	ANKRD36BP2
RNA162083|XLOC_013684	0.000207269	1.49E-05	2.037871	up	ENST00000420070	processed_transcript	RP11-216C10.1
RNA158300|XLOC_000505	0.002987023	0.000576	2.050029	up	ENST00000417409	processed_transcript	RP11-161I10.1
RNA165826|XLOC_008185	0.0331013	0.015156	2.067587	up	ENST00000417942	processed_transcript	XIST
RNA159260|XLOC_009653	0.000765082	8.8E-05	2.122484	up	ENST00000500527	processed_transcript	RP11-551L14.5

RNA163539|XLOC_004204	0.006308576	0.001631	2.182036	up	ENST00000317596	processed_transcript	RP11-290F5.2
RNA158682|XLOC_008823	0.019221764	0.007448	2.261848	up	ENST00000435813	processed_transcript	RP11-346D6.6
RNA158786|XLOC_008583	0.000351966	3.05E-05	2.41833	up	ENST00000454935	processed_transcript	LINC00263
RNA163065|XLOC_003407	0.028256841	0.012336	2.428613	up	ENST00000424493	processed_transcript	AC024560.2
RNA162064|XLOC_013669	0.042974237	0.021125	2.431747	up	ENST00000412553	processed_transcript	RP5-1068E13.3
RNA160776|XLOC_012236	0.002096497	0.000353	2.448935	up	ENST00000515692	processed_transcript	RP11-304F15.4
RNA162613|XLOC_002569	0.005566309	0.001366	3.388933	up	ENST00000414438	processed_transcript	AC069277.2
RNA162925|XLOC_003293	0.01374111	0.004775	3.457706	up	ENST00000493214	processed_transcript	RP11-23D24.2
RNA165312|XLOC_006925	5.95484E-05	2.71E-06	3.522234	up	ENST00000522414	processed_transcript	PVT1
RNA161931|XLOC_001856	9.35792E-05	5.19E-06	2.008333	up	ENST00000427280	processed_transcript	AC073321.5
RNA162205|XLOC_013566	0.011908847	0.00392	2.16074	up	ENST00000428009	processed_transcript	RP4-723E3.1
RNA162966|XLOC_002916	0.00266303	0.000491	2.165079	up	ENST00000469301	processed_transcript	RP11-379K17.4
RNA165197|XLOC_006836	0.022530716	0.009193	2.015214	up	ENST00000520273	processed_transcript	XKR9
RNA160661|XLOC_012093	0.004585536	0.001044	2.097589	up	ENST00000563763	processed_transcript	WSCD1
RNA161747|XLOC_001671	0.000985775	0.000124	2.778216	up	ENST00000325390	processed_transcript	AC018865.8
RNA170370|ENST00000410569	0.000448753	4.25E-05	4.235259	up	ENST00000523190	processed_transcript	PVT1
RNA50171|UCSC_9396_963	0.008209241	0.002357	2.012927	up	ENST00000457079	processed_transcript	AC139666.1
RNA46605|UCSC_5108_1965	0.001913248	0.000311	2.015511	up	ENST00000500447	processed_transcript	MIR210HG
RNA39570|RefSeq_1560_2144	0.028044619	0.012213	2.026642	up	ENST00000437991	processed_transcript	AC005041.9
RNA41058|RefSeq_3167_641	0.00212777	0.000359	2.034611	up	ENST00000502301	processed_transcript	LINC00461
RNA35318|ENCODE_177_2333	0.005683506	0.001405	2.035181	up	ENST00000439050	processed_transcript	AC009299.3
RNA51259|UCSC_10744_591	0.036174282	0.016985	2.05021	up	ENST00000540739	processed_transcript	RP11-669N7.2
RNA35147|ENCODE_1_9124	0.000670118	7.33E-05	2.063762	up	ENST00000427447	processed_transcript	AP000569.8
RNA35692|ENCODE_560_1008	0.000606909	6.4E-05	2.07652	up	ENST00000422414	processed_transcript	RP4-781K5.7
RNA50100|UCSC_9310_982	0.004290487	0.000953	2.078406	up	ENST00000457079	processed_transcript	AC139666.1
RNA38887|RefSeq_830_3069	0.002608778	0.000477	2.081768	up	ENST00000488315	processed_transcript	RP4-751H13.6
RNA47599|UCSC_6313_1690	0.001012461	0.000129	2.084013	up	ENST00000417359	processed_transcript	RP11-379F12.3
RNA49675|UCSC_8800_1093	0.001612686	0.000245	2.086527	up	ENST00000455304	processed_transcript	LINC00466
RNA45985|UCSC_4326_2153	0.009501581	0.002888	2.097679	up	ENST00000317596	processed_transcript	RP11-290F5.2
RNA58816|asoverlaps_402_1912	0.005407301	0.001312	2.106782	up	ENST00000577137	processed_transcript	RP11-629G13.1
RNA35608|ENCODE_476_1204	0.002715611	0.000505	2.111866	up	ENST00000412871	processed_transcript	RP11-354K1.1
RNA45274|UCSC_3434_2432	0.001205516	0.000163	2.118506	up	ENST00000510832	processed_transcript	RP11-219G10.3
RNA58789|asoverlaps_369_1983	0.031726968	0.01433	2.124405	up	ENST00000510576	processed_transcript	CTB-113P19.1
RNA34955|NRED_1096_1038	1.30885E-05	2.99E-07	2.129751	up	ENST00000417578	processed_transcript	RP4-564F22.2
RNA48915|UCSC_7905_1300	0.030690437	0.013737	2.15009	up	ENST00000512067	processed_transcript	RP11-46C20.1
RNA40539|RefSeq_2602_1177	0.024179963	0.010081	2.151153	up	ENST00000509473	processed_transcript	RP11-807H7.1

RNA34432|NRED_564_2019	0.012907865	0.004374	2.153847	up	ENST00000417942	processed_transcript	XIST
RNA52781|H-InvDB_34_1182	0.004784938	0.001107	2.159192	up	ENST00000548760	processed_transcript	RP11-181C3.2
RNA36126|ENCODE_996_724	0.000907275	0.000111	2.159738	up	ENST00000434384	processed_transcript	AC004383.5
RNA35704|ENCODE_572_972	0.038677786	0.018481	2.20502	up	ENST00000435813	processed_transcript	RP11-346D6.6
RNA43420|UCSC_1153_3868	0.016687877	0.006185	2.221859	up	ENST00000360785	processed_transcript	C20orf203
RNA35117|NRED_1258_383	0.018031863	0.006851	2.238983	up	ENST00000417942	processed_transcript	XIST
RNA34976|NRED_1117_974	1.35896E-05	3.17E-07	2.240059	up	ENST00000417578	processed_transcript	RP4-564F22.2
RNA46793|UCSC_5320_1918	0.011102024	0.003566	2.277218	up	ENST00000420224	processed_transcript	RP11-3B12.3
RNA33794|lncRNAdb_1_19271	0.002390167	0.000422	2.28223	up	ENST00000417942	processed_transcript	XIST
RNA43836|UCSC_1653_3432	0.002338229	0.000409	2.285427	up	ENST00000500538	processed_transcript	RP11-453E17.1
RNA47271|UCSC_5894_1786	0.000750186	8.55E-05	2.312692	up	ENST00000423737	processed_transcript	RP11-191L9.4
RNA35108|NRED_1249_444	0.000404661	3.69E-05	2.338251	up	ENST00000519481	processed_transcript	PVT1
RNA49668|UCSC_8792_1094	0.000731843	8.3E-05	2.368877	up	ENST00000464958	processed_transcript	RP5-1157M23.2
RNA37495|ENCODE_2366_407	0.005490148	0.00134	2.387208	up	ENST00000412855	processed_transcript	RP11-396C23.2
RNA35282|ENCODE_141_2551	8.06216E-05	4.19E-06	2.401545	up	ENST00000454935	processed_transcript	LINC00263
RNA35214|ENCODE_70_3177	0.001259571	0.000174	2.4083	up	ENST00000417975	processed_transcript	RP11-475O6.1
RNA49246|UCSC_8284_1209	0.03280463	0.014974	2.417581	up	ENST00000430694	processed_transcript	IGKJ5
RNA48100|UCSC_6908_1558	0.000935133	0.000115	2.44248	up	ENST00000504230	processed_transcript	CTC-497E21.4
RNA34914|NRED_1048_1149	0.003604515	0.000747	2.449225	up	ENST00000490441	processed_transcript	RP11-446H18.3
RNA46460|UCSC_4932_2007	0.002765442	0.000518	2.459223	up	ENST00000519104	processed_transcript	RP3-399L15.3
RNA46670|UCSC_5185_1951	0.018824061	0.007239	2.482231	up	ENST00000412553	processed_transcript	RP5-1068E13.3
RNA38840|RefSeq_781_3161	0.00253277	0.000457	2.498777	up	ENST00000548760	processed_transcript	RP11-181C3.2
RNA50424|UCSC_9711_886	0.000615322	6.52E-05	2.508559	up	ENST00000437488	processed_transcript	RP11-385J1.2
RNA38458|RefSeq_375_4292	0.027786622	0.012066	2.516058	up	ENST00000513919	processed_transcript	RP11-844P9.2
RNA58709|asoverlaps_262_2240	0.010506476	0.00331	2.528634	up	ENST00000422059	processed_transcript	RP5-1120P11.1
RNA35414|ENCODE_277_1960	0.027218472	0.011764	2.567181	up	ENST00000414772	processed_transcript	RP5-1158E12.3
RNA34624|NRED_756_1716	0.000408504	3.75E-05	2.588548	up	ENST00000522414	processed_transcript	PVT1
RNA35494|ENCODE_358_1713	0.037540857	0.017798	2.61023	up	ENST00000412553	processed_transcript	RP5-1068E13.3
RNA51532|UCSC_11096_482	0.000648978	7.01E-05	2.613509	up	ENST00000522963	processed_transcript	PVT1
RNA38367|RefSeq_282_4709	0.011177029	0.0036	2.622066	up	ENST00000419766	processed_transcript	NPSR1-AS1
RNA36311|ENCODE_1181_650	0.012645835	0.004252	2.733199	up	ENST00000418134	processed_transcript	RP1-60O19.1
RNA35346|ENCODE_207_2229	0.002329101	0.000407	2.851279	up	ENST00000418344	processed_transcript	RP5-1070A16.1
RNA34888|NRED_1022_1208	0.000312047	2.58E-05	2.874089	up	ENST00000458151	processed_transcript	RP5-1024G6.5
RNA38384|RefSeq_299_4622	0.002517662	0.000453	2.912142	up	ENST00000435328	processed_transcript	AC018712.3
RNA34740|NRED_872_1536	0.000486269	4.73E-05	2.978275	up	ENST00000521951	processed_transcript	PVT1
RNA51818|UCSC_11411_369	0.00121789	0.000166	3.029789	up	ENST00000431669	processed_transcript	NPSR1-AS1

RNA38353|RefSeq_268_4859	0.005739621	0.001425	3.098712	up	ENST00000419766	processed_transcript	NPSR1-AS1
RNA39817|RefSeq_1825_1901	0.000301622	2.46E-05	3.165613	up	ENST00000513868	processed_transcript	PVT1
RNA36250|ENCODE_1120_674	0.003812568	0.000809	3.283334	up	ENST00000437892	processed_transcript	RP11-124L5.7
RNA50909|UCSC_10307_716	0.000229514	1.71E-05	3.426	up	ENST00000512839	processed_transcript	RP11-219G10.3
RNA37662|ENCODE_2534_375	0.01371315	0.004761	3.430771	up	ENST00000414772	processed_transcript	RP5-1158E12.3
RNA58410|CombinedLit_408_341	0.003740478	0.000787	3.554075	up	ENST00000442669	processed_transcript	NPSR1-AS1
RNA36595|ENCODE_1465_570	0.011951482	0.003941	3.708605	up	ENST00000435813	processed_transcript	RP11-346D6.6
RNA50596|UCSC_9921_829	0.001746578	0.000273	3.752325	up	ENST00000537560	processed_transcript	NPSR1-AS1
RNA35314|ENCODE_173_2366	0.006564093	0.001722	3.774894	up	ENST00000366181	processed_transcript	RP11-296A18.3
RNA50585|UCSC_9907_833	0.000707181	7.9E-05	3.891871	up	ENST00000537560	processed_transcript	NPSR1-AS1
RNA51636|UCSC_11214_442	0.001163963	0.000155	3.905749	up	ENST00000456627	processed_transcript	LINC00355
RNA37424|ENCODE_2295_421	0.000700515	7.8E-05	3.973673	up	ENST00000449012	processed_transcript	RP4-781K5.4
RNA50682|UCSC_10038_798	0.000162041	1.07E-05	4.043389	up	ENST00000537560	processed_transcript	NPSR1-AS1
RNA50223|UCSC_9460_948	0.002052936	0.000342	4.1488	up	ENST00000537560	processed_transcript	NPSR1-AS1
RNA58401|CombinedLit_393_376	0.002582709	0.00047	4.248	up	ENST00000442669	processed_transcript	NPSR1-AS1
RNA39074|RefSeq_1032_2737	2.60678E-05	8.22E-07	4.538217	up	ENST00000435097	processed_transcript	RP11-429J17.6
RNA35434|ENCODE_298_1878	0.004802466	0.001113	4.872703	up	ENST00000456627	processed_transcript	LINC00355
RNA58351|CombinedLit_316_550	0.000670096	7.33E-05	5.296575	up	ENST00000358772	processed_transcript	NPSR1-AS1
RNA44563|UCSC_2544_2838	0.000120008	7.25E-06	5.449033	up	ENST00000508406	processed_transcript	RP11-8L2.1
RNA39246|RefSeq_1214_2495	0.001288077	0.000179	7.23221	up	ENST00000492337	processed_transcript	SOX2-OT
RNA46043|UCSC_4403_2131	0.003372891	0.000683	8.531747	up	ENST00000500112	processed_transcript	RP11-255B23.3
RNA48095|UCSC_6903_1559	9.78778E-05	5.54E-06	14.27874	up	ENST00000532541	processed_transcript	CTC-497E21.4
RNA37628|ENCODE_2500_381	0.002354572	0.000412	2.007991	up	ENST00000420819	processed_transcript	RP1-27K12.2
RNA42896|UCSC_531_5053	1.10399E-05	2.36E-07	2.02567	up	ENST00000578417	processed_transcript	RP11-403I13.4
RNA35116|NRED_1257_392	0.001061129	0.000137	2.054302	up	ENST00000400476	processed_transcript	AC007965.12
RNA51983|UCSC_11599_304	0.005735396	0.001424	2.082516	up	ENST00000527329	processed_transcript	RP11-652L8.2
RNA34176|NRED_307_2612	0.000396153	3.58E-05	2.119014	up	ENST00000437681	processed_transcript	SNHG3
RNA35023|NRED_1164_849	0.000382143	3.4E-05	2.145702	up	ENST00000437681	processed_transcript	SNHG3
RNA35013|NRED_1154_888	0.000398471	3.61E-05	2.15972	up	ENST00000437681	processed_transcript	SNHG3
RNA35017|NRED_1158_877	0.000281771	2.25E-05	2.160494	up	ENST00000437681	processed_transcript	SNHG3
RNA35016|NRED_1157_878	0.000274414	2.17E-05	2.173909	up	ENST00000437681	processed_transcript	SNHG3
RNA47704|UCSC_6437_1662	0.000958153	0.000119	2.179585	up	ENST00000427391	processed_transcript	RP11-453F18__B.1
RNA35014|NRED_1155_885	0.000147363	9.51E-06	2.188083	up	ENST00000437681	processed_transcript	SNHG3
RNA35012|NRED_1153_889	0.000508906	5.04E-05	2.201339	up	ENST00000437681	processed_transcript	SNHG3
RNA48606|UCSC_7524_1402	0.001807026	0.000287	2.271723	up	ENST00000428809	processed_transcript	AP001347.6
RNA47767|UCSC_6512_1645	0.002559864	0.000464	2.288528	up	ENST00000547804	processed_transcript	RP11-77I22.3

RNA37336|ENCODE_2207_435	0.000235527	1.77E-05	2.342604	up	ENST00000425510	processed_transcript	RP11-357H14.20
RNA37132|ENCODE_2002_467	0.005731142	0.001422	2.48344	up	ENST00000446055	processed_transcript	RP5-837M10.2
RNA45035|UCSC_3133_2550	0.000577757	5.98E-05	2.598112	up	ENST00000444070	processed_transcript	AL353997.3
RNA51311|UCSC_10811_570	0.004647883	0.001064	2.680554	up	ENST00000447037	processed_transcript	AP001469.9
RNA38962|RefSeq_909_2928	0.000127485	7.87E-06	2.819182	up	ENST00000427391	processed_transcript	RP11-453F18__B.1
RNA46655|UCSC_5168_1956	0.003806556	0.000807	3.151483	up	ENST00000445817	processed_transcript	CTD-2090I13.1
RNA51077|UCSC_10510_656	0.000217067	1.58E-05	3.158627	up	ENST00000416066	processed_transcript	RP11-498P14.3
RNA51086|UCSC_10522_652	0.000169925	1.14E-05	3.190651	up	ENST00000416066	processed_transcript	RP11-498P14.3
RNA45675|UCSC_3948_2272	0.006712562	0.001777	3.401128	up	ENST00000490357	processed_transcript	RP11-10O22.1
RNA44357|UCSC_2301_2968	0.000124178	7.57E-06	3.465025	up	ENST00000436123	processed_transcript	AC098971.2
RNA43707|UCSC_1502_3551	0.000461533	4.41E-05	3.516776	up	ENST00000444312	processed_transcript	ATG9B
RNA35277|ENCODE_136_2597	0.000943929	0.000117	5.630406	up	ENST00000431554	processed_transcript	RP11-411K7.1
RNA44606|UCSC_2594_2810	0.000110135	6.48E-06	5.920756	up	ENST00000518128	processed_transcript	RP11-758M4.4
RNA51357|UCSC_10865_556	0.034747414	0.016138	2.00269	up	ENST00000514683	processed_transcript	MFAP3L
RNA47909|UCSC_6683_1607	0.004506017	0.001019	2.012966	up	ENST00000468167	processed_transcript	TAF1
RNA48186|UCSC_7024_1530	0.00153962	0.00023	2.014997	up	ENST00000496717	processed_transcript	C3orf65
RNA48044|UCSC_6843_1572	0.00525378	0.001264	2.018647	up	ENST00000469813	processed_transcript	C1orf61
RNA51426|UCSC_10960_529	0.020914683	0.008334	2.029058	up	ENST00000548086	processed_transcript	SCN8A
RNA34843|NRED_977_1318	0.000555865	5.67E-05	2.079432	up	ENST00000545509	processed_transcript	ITFG2
RNA51286|UCSC_10777_583	0.003649825	0.00076	2.087266	up	ENST00000483169	processed_transcript	DLEU1
RNA55074|H-InvDB_2439_256	0.001016221	0.00013	2.100393	up	ENST00000532966	processed_transcript	AMPD3
RNA46991|UCSC_5554_1862	0.000915402	0.000112	2.104251	up	ENST00000461681	processed_transcript	ITGB3BP
RNA51620|UCSC_11197_447	0.003118596	0.000612	2.110994	up	ENST00000405106	processed_transcript	JMJD7
RNA58403|CombinedLit_397_365	0.029023375	0.012798	2.11796	up	ENST00000484869	processed_transcript	DLEU1
RNA51641|UCSC_11219_441	0.012154355	0.004033	2.120085	up	ENST00000470239	processed_transcript	TFB1M
RNA43350|UCSC_1070_3969	0.019455973	0.007574	2.12828	up	ENST00000474063	processed_transcript	DIRC3
RNA51021|UCSC_10443_676	0.041101985	0.019941	2.135065	up	ENST00000560857	processed_transcript	FOXB1
RNA51898|UCSC_11508_339	0.009292766	0.002799	2.141151	up	ENST00000515675	processed_transcript	AFP
RNA49042|UCSC_8046_1269	0.000191692	1.34E-05	2.146559	up	ENST00000473131	processed_transcript	TRMT6
RNA58306|CombinedLit_251_719	0.001553893	0.000233	2.16003	up	ENST00000472136	processed_transcript	DLEU1
RNA34808|NRED_941_1403	0.003001252	0.00058	2.184367	up	ENST00000479850	processed_transcript	CCDC169
RNA58331|CombinedLit_287_612	0.001218688	0.000166	2.193591	up	ENST00000479420	processed_transcript	DLEU1
RNA51884|UCSC_11485_346	0.001330661	0.000187	2.195256	up	ENST00000532966	processed_transcript	AMPD3
RNA49538|UCSC_8641_1125	0.003634481	0.000756	2.201321	up	ENST00000528161	processed_transcript	IMMP1L
RNA41149|RefSeq_3267_548	0.000321498	2.69E-05	2.220929	up	ENST00000470242	processed_transcript	PRRT1
RNA50649|UCSC_9989_811	5.61194E-05	2.48E-06	2.223446	up	ENST00000461527	processed_transcript	DLEU1

RNA41067|RefSeq_3179_628	0.003188897	0.000631	2.246402	up	ENST00000425537	processed_transcript	HMGB4
RNA50019|UCSC_9211_1001	0.005863986	0.001471	2.262983	up	ENST00000525411	processed_transcript	PATE4
RNA55023|H-InvDB_2386_262	0.000861825	0.000103	2.266439	up	ENST00000483169	processed_transcript	DLEU1
RNA50447|UCSC_9740_879	0.00289093	0.000551	2.302632	up	ENST00000487472	processed_transcript	PTH2R
RNA50857|UCSC_10250_731	0.00088674	0.000107	2.317544	up	ENST00000462427	processed_transcript	DLEU1
RNA50716|UCSC_10082_780	0.00010157	5.83E-06	2.321929	up	ENST00000483169	processed_transcript	DLEU1
RNA58346|CombinedLit_310_566	0.000471176	4.54E-05	2.339656	up	ENST00000498557	processed_transcript	DLEU1
RNA50586|UCSC_9908_833	0.018109018	0.006888	2.356105	up	ENST00000462559	processed_transcript	B3GNT5
RNA49320|UCSC_8383_1186	0.000359103	3.13E-05	2.368226	up	ENST00000462427	processed_transcript	DLEU1
RNA44600|UCSC_2587_2814	0.000490527	4.79E-05	2.380892	up	ENST00000476966	processed_transcript	C1orf61
RNA51670|UCSC_11251_431	0.000447323	4.23E-05	2.394283	up	ENST00000462427	processed_transcript	DLEU1
RNA50842|UCSC_10231_736	0.000666654	7.27E-05	2.409052	up	ENST00000462427	processed_transcript	DLEU1
RNA58383|CombinedLit_362_453	0.005781723	0.001441	2.458015	up	ENST00000480262	processed_transcript	DLEU1
RNA47369|UCSC_6009_1761	0.022655314	0.009257	2.484796	up	ENST00000461897	processed_transcript	COL4A6
RNA51318|UCSC_10820_568	0.002641742	0.000486	2.538328	up	ENST00000479817	processed_transcript	MNX1
RNA58444|CombinedLit_465_209	0.002390544	0.000422	2.564449	up	ENST00000479817	processed_transcript	MNX1
RNA49575|UCSC_8683_1119	0.000220865	1.62E-05	2.577845	up	ENST00000462427	processed_transcript	DLEU1
RNA45612|UCSC_3871_2293	0.008790868	0.002592	2.601017	up	ENST00000455723	processed_transcript	TMSB15B
RNA58438|CombinedLit_455_248	7.33928E-05	3.67E-06	2.634581	up	ENST00000483169	processed_transcript	DLEU1
RNA47753|UCSC_6493_1650	0.00180438	0.000286	2.681809	up	ENST00000562840	processed_transcript	C16orf95
RNA43652|UCSC_1434_3600	0.006275753	0.00162	2.697423	up	ENST00000471537	processed_transcript	CCBP2
RNA46898|UCSC_5447_1887	0.014254867	0.00501	2.723988	up	ENST00000461897	processed_transcript	COL4A6
RNA49070|UCSC_8079_1261	0.006635539	0.001748	2.726267	up	ENST00000500334	processed_transcript	HOMER2
RNA47319|UCSC_5950_1774	0.000775765	8.96E-05	2.83987	up	ENST00000492852	processed_transcript	CYP19A1
RNA40293|RefSeq_2336_1444	0.00179384	0.000284	2.852623	up	ENST00000484801	processed_transcript	KHDC1
RNA50458|UCSC_9753_875	0.000606909	6.4E-05	2.983826	up	ENST00000462427	processed_transcript	DLEU1
RNA51413|UCSC_10940_534	0.00045988	4.39E-05	3.00504	up	ENST00000484606	processed_transcript	DLEU1
RNA58317|CombinedLit_267_667	0.000962783	0.00012	3.021306	up	ENST00000470593	processed_transcript	DLEU1
RNA48322|UCSC_7187_1487	0.000590291	6.16E-05	3.045087	up	ENST00000470593	processed_transcript	DLEU1
RNA51741|UCSC_11332_400	0.00105473	0.000136	3.111849	up	ENST00000484529	processed_transcript	DLEU1
RNA58326|CombinedLit_279_638	3.4361E-05	1.2E-06	3.149832	up	ENST00000498557	processed_transcript	DLEU1
RNA49163|UCSC_8188_1233	0.000318152	2.65E-05	3.187026	up	ENST00000470593	processed_transcript	DLEU1
RNA58432|CombinedLit_440_285	0.000915064	0.000112	3.216449	up	ENST00000470726	processed_transcript	DLEU1
RNA48768|UCSC_7735_1344	0.000237395	1.79E-05	3.21873	up	ENST00000470593	processed_transcript	DLEU1
RNA48757|UCSC_7722_1348	0.000265883	2.08E-05	3.239653	up	ENST00000470593	processed_transcript	DLEU1
RNA49892|UCSC_9065_1034	0.000485405	4.72E-05	3.263811	up	ENST00000470593	processed_transcript	DLEU1

RNA48369|UCSC_7240_1473	2.08528E-05	5.94E-07	3.285537	up	ENST00000476901	processed_transcript	TRMU
RNA58316|CombinedLit_266_668	0.000868729	0.000104	3.291315	up	ENST00000462427	processed_transcript	DLEU1
RNA58406|CombinedLit_400_355	0.000278731	2.22E-05	3.324163	up	ENST00000470593	processed_transcript	DLEU1
RNA51029|UCSC_10451_674	3.15427E-05	1.06E-06	3.408218	up	ENST00000505522	processed_transcript	MATR3
RNA51234|UCSC_10712_598	2.42922E-05	7.41E-07	3.422241	up	ENST00000482879	processed_transcript	SDC1
RNA49825|UCSC_8985_1051	0.000263594	2.06E-05	3.442479	up	ENST00000468522	processed_transcript	DLEU1
RNA49449|UCSC_8531_1150	4.60107E-05	1.82E-06	3.47791	up	ENST00000476901	processed_transcript	TRMU
RNA40683|RefSeq_2758_1047	8.59918E-06	1.64E-07	3.500428	up	ENST00000507197	processed_transcript	MATR3
RNA58397|CombinedLit_387_387	0.001221208	0.000166	3.517756	up	ENST00000498557	processed_transcript	DLEU1
RNA48901|UCSC_7891_1304	0.001159423	0.000155	3.771082	up	ENST00000483169	processed_transcript	DLEU1
RNA35113|NRED_1254_417	0.007692058	0.002151	3.825724	up	ENST00000488388	processed_transcript	CISD1
RNA47735|UCSC_6472_1652	0.001369251	0.000195	3.831414	up	ENST00000484869	processed_transcript	DLEU1
RNA51874|UCSC_11474_352	0.000111718	6.61E-06	3.903081	up	ENST00000508735	processed_transcript	MATR3
RNA50251|UCSC_9498_940	0.00594885	0.001502	3.92769	up	ENST00000463474	processed_transcript	DLEU1
RNA49904|UCSC_9080_1031	0.006070556	0.001545	4.525998	up	ENST00000462427	processed_transcript	DLEU1
RNA54791|H-InvDB_2132_291	0.001290397	0.00018	4.967174	up	ENST00000432435	processed_transcript	MTL5
RNA51575|UCSC_11144_466	0.000107212	6.26E-06	5.292925	up	ENST00000216414	processed_transcript	CDKN3
RNA51864|UCSC_11463_356	0.002875558	0.000546	6.124056	up	ENST00000489590	processed_transcript	OFCC1
RNA51824|UCSC_11419_366	0.000757181	8.67E-05	6.585046	up	ENST00000515325	processed_transcript	NMU
RNA38979|RefSeq_929_2897	0.002032537	0.000337	2.011257	up	ENST00000435620	processed_transcript	ANKRD18DP
RNA46160|UCSC_4545_2096	0.005663728	0.001398	2.011805	up	ENST00000455565	processed_transcript	AC126544.2
RNA45124|UCSC_3235_2513	0.000712815	8E-05	2.012972	up	ENST00000568567	processed_transcript	RP11-17M15.2
RNA44870|UCSC_2926_2642	0.000908234	0.000111	2.016591	up	ENST00000435620	processed_transcript	ANKRD18DP
RNA39990|RefSeq_2014_1730	0.013423802	0.004624	2.021608	up	ENST00000393515	processed_transcript	ANKRD36BP2
RNA44924|UCSC_2991_2607	0.003248648	0.000648	2.078625	up	ENST00000455565	processed_transcript	AC126544.2
RNA46124|UCSC_4502_2107	0.033364702	0.015307	2.090901	up	ENST00000443839	processed_transcript	SMPD4P1
RNA46249|UCSC_4663_2070	0.032008942	0.014493	2.170253	up	ENST00000449066	processed_transcript	AC140481.1
RNA40573|RefSeq_2638_1143	0.01789514	0.006784	2.180556	up	ENST00000489520	processed_transcript	RPSAP52
RNA38606|RefSeq_532_3763	0.000573258	5.92E-05	2.199693	up	ENST00000495140	processed_transcript	C3P1
RNA38402|RefSeq_317_4522	0.000418173	3.87E-05	2.239346	up	ENST00000456785	processed_transcript	AC018865.8
RNA35451|ENCODE_315_1832	0.012016472	0.00397	2.24236	up	ENST00000573162	processed_transcript	AC145141.1
RNA40030|RefSeq_2055_1705	0.00946246	0.002871	2.25542	up	ENST00000498728	processed_transcript	ADAM5P
RNA48427|UCSC_7308_1455	0.010919704	0.003487	2.258697	up	ENST00000498728	processed_transcript	ADAM5P
RNA48548|UCSC_7456_1419	8.55287E-05	4.57E-06	2.263366	up	ENST00000442266	processed_transcript	CCT6P1
RNA39462|RefSeq_1444_2249	0.023959711	0.009963	2.276162	up	ENST00000446690	processed_transcript	COL6A4P1
RNA42823|UCSC_437_5360	0.008495528	0.002473	2.284364	up	ENST00000393525	processed_transcript	ANKRD36BP2

RNA36797|ENCODE_1667_531	0.002538206	0.000458	2.295723	up	ENST00000485590	processed_transcript	GUCY1B2
RNA46250|UCSC_4664_2070	0.026160084	0.011166	2.303495	up	ENST00000449066	processed_transcript	AC140481.1
RNA47791|UCSC_6541_1639	0.008636435	0.00253	2.311684	up	ENST00000498728	processed_transcript	ADAM5P
RNA48214|UCSC_7057_1521	0.009261911	0.002784	2.334277	up	ENST00000498728	processed_transcript	ADAM5P
RNA43956|UCSC_1799_3336	0.01303635	0.004436	2.369447	up	ENST00000562009	processed_transcript	CTD-2311M21.2
RNA40741|RefSeq_2821_985	0.005947929	0.001502	2.376622	up	ENST00000411440	processed_transcript	CELP
RNA40801|RefSeq_2885_925	0.002816101	0.000531	2.428161	up	ENST00000569142	processed_transcript	SLC25A3P1
RNA40061|RefSeq_2087_1675	0.00319274	0.000633	2.454714	up	ENST00000435380	processed_transcript	NEURL3
RNA40981|RefSeq_3082_711	0.012634187	0.004247	2.485332	up	ENST00000429807	processed_transcript	RP11-210H10__A.1
RNA39060|RefSeq_1014_2768	0.02782245	0.012087	2.531285	up	ENST00000575787	processed_transcript	ALOX12P2
RNA40382|RefSeq_2435_1357	0.001660008	0.000255	2.545106	up	ENST00000525246	processed_transcript	TDH
RNA40679|RefSeq_2754_1049	0.002641922	0.000486	2.656947	up	ENST00000504307	processed_transcript	HILS1
RNA46725|UCSC_5246_1937	0.001408074	0.000202	2.660982	up	ENST00000359790	processed_transcript	ADAM5P
RNA45816|UCSC_4127_2215	0.02107271	0.008412	2.675859	up	ENST00000575787	processed_transcript	ALOX12P2
RNA47831|UCSC_6589_1630	0.00147019	0.000215	2.69499	up	ENST00000359790	processed_transcript	ADAM5P
RNA39583|RefSeq_1573_2136	0.043660227	0.021539	2.707078	up	ENST00000439871	processed_transcript	CXCR2P1
RNA44569|UCSC_2550_2834	0.025393263	0.01075	2.711942	up	ENST00000458685	processed_transcript	NOS2P3
RNA40275|RefSeq_2316_1473	0.02577072	0.010957	2.787732	up	ENST00000494676	processed_transcript	RPL13AP3
RNA47109|UCSC_5702_1829	0.003167624	0.000625	2.80057	up	ENST00000417510	processed_transcript	AC022596.6
RNA38691|RefSeq_624_3525	0.002021084	0.000335	2.887807	up	ENST00000496593	processed_transcript	RPLP0P2
RNA40376|RefSeq_2427_1362	0.016672477	0.006177	3.040598	up	ENST00000477875	processed_transcript	FMO9P
RNA61330|RNAz_2044_270	0.002090057	0.000351	3.122174	up	ENST00000557452	processed_transcript	RP11-74M13.4
RNA39743|RefSeq_1746_1992	6.11622E-05	2.82E-06	3.517709	up	ENST00000438684	processed_transcript	MSL3P1
RNA40894|RefSeq_2991_815	0.002392125	0.000423	3.650698	up	ENST00000421868	processed_transcript	AC114812.5
RNA49759|UCSC_8900_1070	0.001018215	0.00013	3.719605	up	ENST00000570835	processed_transcript	ALOX12P2
RNA40700|RefSeq_2778_1030	0.000296722	2.41E-05	3.772451	up	ENST00000421868	processed_transcript	AC114812.5
RNA38111|RefSeq_11_11312	0.022032289	0.008923	4.019129	up	ENST00000423298	processed_transcript	RP11-443P15.2
RNA41014|RefSeq_3121_678	0.000996692	0.000126	4.042944	up	ENST00000421868	processed_transcript	AC114812.5
RNA44949|UCSC_3027_2595	2.60252E-05	8.18E-07	4.33176	up	ENST00000494840	processed_transcript	RP11-219E24.1
RNA33960|NRED_90_4062	0.013809107	0.004806	4.738697	up	ENST00000423298	processed_transcript	RP11-443P15.2
RNA50350|UCSC_9620_911	0.00475908	0.001099	4.945671	up	ENST00000579693	processed_transcript	MTND1P15
RNA39981|RefSeq_2005_1739	0.000427375	3.98E-05	5.583176	up	ENST00000560285	processed_transcript	AC131180.4
RNA40475|RefSeq_2532_1257	0.009342096	0.00282	6.106606	up	ENST00000378770	processed_transcript	HSP90AA4P
RNA51496|UCSC_11050_498	0.000267256	2.1E-05	6.927998	up	ENST00000556890	processed_transcript	CTD-3006G17.2
RNA41127|RefSeq_3244_575	0.001071616	0.000139	8.393152	up	ENST00000405395	processed_transcript	KRT19P2
RNA177331|ENST00000469070	0.007971196	0.002264	2.045347	up	ENST00000469070	intronic	RP11-18H7.1

RNA177484|ENST00000437589	0.005381438	0.001304	2.076233	up	ENST00000424351	intronic	AC017076.4
RNA175291|ENST00000510062	0.013479681	0.004649	2.091306	up	ENST00000515405	intronic	RP11-366M4.3
RNA175134|ENST00000501224	0.033940088	0.015647	2.125673	up	ENST00000501224	intronic	EXTL3-AS1
RNA175796|ENST00000502076	0.007166674	0.001946	2.228594	up	ENST00000502076	intronic	SNHG6
RNA177284|ENST00000300458	0.010182159	0.003173	2.314406	up	ENST00000300458	intronic	LINC00483
RNA175948|ENST00000411630	8.31923E-05	4.39E-06	2.397095	up	ENST00000425653	intronic	DANCR
RNA177482|ENST00000439208	0.000875185	0.000105	2.399858	up	ENST00000424351	intronic	AC017076.4
RNA175950|ENST00000425653	8.28092E-05	4.36E-06	2.454119	up	ENST00000425653	intronic	DANCR
RNA177765|ENST00000442967	0.001707676	0.000265	2.849583	up	ENST00000442967	intronic	AC017096.1
RNA175057|ENST00000428922	0.019455202	0.007573	2.960309	up	ENST00000428922	intronic	NPSR1-AS1
RNA177922|ENST00000426452	0.00092176	0.000113	4.300632	up	ENST00000426452	intronic	RP11-191L9.4
RNA174905|ENST00000435967	0.001148067	0.000153	4.661341	up	ENST00000435967	intronic	AC007128.1
RNA177416|ENST00000425330	0.000429037	4E-05	5.466144	up	ENST00000425330	intronic	RP11-385J1.2
RNA177527|ENST00000491282	0.002691038	0.000499	5.846481	up	ENST00000466034	intronic	SOX2-OT
RNA177526|ENST00000476964	0.005143905	0.001227	6.314817	up	ENST00000466034	intronic	SOX2-OT
RNA177518|ENST00000469278	0.001327167	0.000186	6.502741	up	ENST00000466034	intronic	SOX2-OT
RNA177531|ENST00000466034	0.003212676	0.000638	6.868008	up	ENST00000466034	intronic	SOX2-OT
RNA177519|ENST00000493521	0.001888345	0.000305	7.085335	up	ENST00000477928	intronic	SOX2-OT
RNA177530|ENST00000477928	0.001996391	0.000329	7.417943	up	ENST00000477928	intronic	SOX2-OT
RNA177415|ENST00000421498	0.000237084	1.78E-05	10.07914	up	ENST00000421498	intronic	RP11-385J1.2
RNA176970|ENST00000421068	0.002686829	0.000497	2.495016	up	ENST00000421068	intronic	GAS5
RNA178060|ENST00000540865	7.17269E-06	1.24E-07	2.515414	up	ENST00000540865	intronic	SNHG1
RNA178059|ENST00000545920	1.76727E-06	1.42E-08	2.602466	up	ENST00000545920	intronic	SNHG1
RNA178058|ENST00000544550	1.69392E-06	1.31E-08	2.795303	up	ENST00000544550	intronic	SNHG1
RNA165082|XLOC_007033	0.039931804	0.019224	2.193013	up	ENST00000501224	intronic	EXTL3-AS1
RNA161055|XLOC_012723	0.002711325	0.000504	2.274015	up	ENST00000578560	intronic	RP11-1O2.1
RNA160768|XLOC_012503	6.57537E-05	3.13E-06	2.736793	up	ENST00000578660	intronic	RP5-890E16.2
RNA38969|RefSeq_916_2922	0.028887743	0.012714	2.156464	up	ENST00000517675	intronic	LINC00599
RNA43572|UCSC_1336_3678	0.027145976	0.011723	2.180299	up	ENST00000501224	intronic	EXTL3-AS1
RNA40861|RefSeq_2951_855	5.17361E-05	2.19E-06	2.345506	up	ENST00000425653	intronic	DANCR
RNA44380|UCSC_2325_2954	0.009003846	0.002677	2.594586	up	ENST00000424351	intronic	AC017076.4
RNA51477|UCSC_11027_506	0.019729048	0.007718	2.690265	up	ENST00000428922	intronic	NPSR1-AS1
RNA49390|UCSC_8465_1168	0.001912971	0.000311	5.330582	up	ENST00000426452	intronic	RP11-191L9.4
RNA54270|H-InvDB_1573_346	0.000106839	6.23E-06	5.86087	up	ENST00000421498	intronic	RP11-385J1.2
RNA34812|NRED_945_1396	2.96957E-05	9.79E-07	2.386068	up	ENST00000535076	intronic	SNHG1
RNA36477|ENCODE_1347_595	0.024236748	0.010112	2.816376	up	ENST00000567101	intronic	RP11-357H14.19

RNA33839|lncRNAdb_46_1100	0.000237835	1.79E-05	2.023286	up	ENST00000514110	intronic	MATR3
RNA44147|UCSC_2033_3160	0.028743943	0.012631	2.027033	up	ENST00000556729	intronic	DHRS2
RNA96612|EvoFold_451_218	4.1423E-05	1.58E-06	2.028847	up	ENST00000477232	intronic	PSMD14
RNA49360|UCSC_8431_1177	0.007271751	0.001987	2.054287	up	ENST00000380416	intronic	PMS2
RNA48476|UCSC_7368_1438	0.002639875	0.000485	2.111172	up	ENST00000507616	intronic	EME1
RNA53031|H-InvDB_290_681	0.000242219	1.84E-05	2.135272	up	ENST00000514110	intronic	MATR3
RNA40863|RefSeq_2954_852	0.03435168	0.015895	2.157107	up	ENST00000484440	intronic	GPAT2
RNA60951|RNAz_1665_277	0.00099656	0.000126	2.318157	up	ENST00000478257	intronic	AIF1L
RNA44919|UCSC_2985_2611	0.005009975	0.001182	2.414808	up	ENST00000584132	intronic	SOCS7
RNA57697|UCRs_12_442	2.60071E-05	8.16E-07	2.435861	up	ENST00000477327	intronic	HAT1
RNA44365|UCSC_2309_2963	0.000981583	0.000123	2.501056	up	ENST00000468005	intronic	IGF2BP3
RNA48236|UCSC_7088_1513	0.000168458	1.13E-05	2.703902	up	ENST00000497422	intronic	ZDHHC19
RNA48790|UCSC_7762_1338	0.003734584	0.000785	2.724175	up	ENST00000466777	intronic	TNFSF10
RNA47291|UCSC_5917_1781	0.001016221	0.00013	2.884207	up	ENST00000468653	intronic	SMC4
RNA38769|RefSeq_707_3337	2.25527E-05	6.67E-07	3.216959	up	ENST00000488743	intronic	SLC5A6
RNA45809|UCSC_4119_2218	0.004256249	0.000943	2.076811	up	ENST00000412452	intronic	FER1L4
RNA38199|RefSeq_107_6579	0.015197679	0.005451	2.198139	up	ENST00000400465	intronic	FER1L4
RNA44287|UCSC_2208_3031	0.005222225	0.001254	2.375679	up	ENST00000556948	intronic	NBEAP1
RNA43479|UCSC_1224_3793	0.002017116	0.000333	2.651346	up	ENST00000449170	intronic	AC018865.8
RNA49296|UCSC_8349_1192	0.014478671	0.005114	4.576722	up	ENST00000445507	intronic	RP11-443P15.2
RNA159270|XLOC_010019	0.008694664	0.002553	2.111784	up	ENST00000540635	intronic	RP11-711K1.7
RNA36094|ENCODE_964_736	0.00766208	0.002139	2.281453	up	ENST00000439318	intronic	AC007003.1
RNA36298|ENCODE_1168_656	0.006561406	0.001721	2.285725	up	ENST00000433058	intronic	RP11-496N12.6
RNA36325|ENCODE_1195_643	0.025684565	0.010906	2.467641	up	ENST00000441845	intronic	MLIP-IT1
RNA50641|UCSC_9979_813	0.004553727	0.001034	2.535074	up	ENST00000561624	intronic	CTD-2270L9.2
RNA38855|RefSeq_797_3138	0.02480365	0.010426	2.649547	up	ENST00000583516	intronic	ESRG
RNA47073|UCSC_5660_1839	0.000450678	4.27E-05	3.219366	up	ENST00000322220	intronic	HECW1-IT1
RNA176411|ENST00000431462	9.38722E-05	5.23E-06	2.015513	up			
RNA176793|ENST00000416157	0.002562271	0.000464	2.027996	up			
RNA176779|ENST00000541710	0.0014289	0.000207	2.061911	up			
RNA176752|ENST00000507442	0.002962448	0.000569	2.100098	up			
RNA176566|ENST00000510453	0.012351542	0.00412	2.107134	up			
RNA176418|ENST00000500519	0.015823051	0.00576	2.146596	up			
RNA176605|ENST00000502946	0.005328339	0.001287	2.205013	up			
RNA176403|ENST00000509547	0.043517698	0.021445	2.250823	up			
RNA176479|ENST00000530512	0.015623688	0.005664	2.299625	up			

RNA176519|ENST00000422709	0.001733966	0.000271	2.422805	up			
RNA176517|ENST00000502335	0.013380876	0.004606	2.483892	up			
RNA176780|ENST00000507720	0.003143887	0.000619	2.486891	up			
RNA176376|ENST00000493038	0.000377925	3.35E-05	3.24657	up			
RNA176518|ENST00000510201	0.007509225	0.002081	3.523171	up			
RNA176757|ENST00000537266	2.60515E-05	8.19E-07	3.775523	up			
RNA176351|ENST00000499528	5.64941E-05	2.51E-06	4.245873	up			
RNA176807|ENST00000508149	2.69037E-05	8.56E-07	10.34914	up			
RNA165076|XLOC_006740	0.01714951	0.006408	2.000912	up			
RNA165394|XLOC_007654	0.04361528	0.021507	2.001489	up			
RNA160851|XLOC_012317	0.002607625	0.000477	2.001683	up			
RNA160512|XLOC_011951	0.030474588	0.013608	2.002858	up			
RNA158093|XLOC_000927	0.016453883	0.006069	2.003269	up			
RNA160179|XLOC_011190	0.014393212	0.005073	2.003856	up			
RNA165386|XLOC_007282	0.007926296	0.002248	2.003933	up			
RNA165081|XLOC_006745	0.003874832	0.000827	2.004211	up			
RNA161419|XLOC_001418	0.008527176	0.002485	2.00593	up			
RNA158666|XLOC_008802	0.011131892	0.00358	2.005971	up			
RNA159522|XLOC_009914	0.013504734	0.004661	2.006534	up			
RNA164426|XLOC_005434	0.000579	6E-05	2.007247	up			
RNA161672|XLOC_001621	0.02303176	0.009472	2.007805	up			
RNA160732|XLOC_012441	0.001984566	0.000326	2.007896	up			
RNA164397|XLOC_005397	0.004430971	0.000995	2.008213	up			
RNA158659|XLOC_008792	0.009703298	0.002969	2.008833	up			
RNA160645|XLOC_012064	9.37674E-05	5.21E-06	2.009258	up			
RNA160291|XLOC_011304	0.008275953	0.002383	2.0106	up			
RNA160448|XLOC_011673	0.007421835	0.002044	2.013343	up			
RNA165128|XLOC_007078	0.04252574	0.020836	2.013613	up			
RNA160722|XLOC_012162	0.001153311	0.000153	2.013686	up			
RNA160148|XLOC_011393	0.005447549	0.001326	2.013922	up			
RNA160259|XLOC_011494	0.002315666	0.000403	2.014924	up			
RNA161047|XLOC_012717	0.01783125	0.006752	2.01544	up			
RNA160048|XLOC_010880	0.011493274	0.003734	2.017561	up			
RNA162360|XLOC_013910	0.025613612	0.010871	2.018532	up			
RNA164770|XLOC_006444	0.005540997	0.001357	2.020051	up			
RNA159295|XLOC_009696	0.022685874	0.009273	2.022563	up			

RNA165738|XLOC_007925	0.000343631	2.94E-05	2.022683	up				
RNA165334|XLOC_006939	0.00626998	0.001618	2.026717	up				
RNA160578|XLOC_012014	0.011630774	0.003795	2.027257	up				
RNA164803|XLOC_006126	0.015763097	0.005729	2.02815	up				
RNA165020|XLOC_006689	0.008251631	0.002375	2.030229	up				
RNA157935|XLOC_000169	0.00415389	0.000909	2.030272	up				
RNA164808|XLOC_006133	0.006133492	0.001568	2.032105	up				
RNA165411|XLOC_007667	0.000321498	2.69E-05	2.034751	up				
RNA158805|XLOC_008603	0.000677319	7.44E-05	2.03483	up				
RNA163184|XLOC_003926	0.006601967	0.001736	2.035447	up				
RNA165160|XLOC_007094	0.000891745	0.000108	2.035528	up				
RNA165613|XLOC_007516	0.037193645	0.017592	2.036243	up				
RNA163172|XLOC_003497	0.002603057	0.000475	2.039102	up				
RNA161216|XLOC_013370	0.010850568	0.003458	2.039482	up				
RNA161265|XLOC_001935	0.001778559	0.00028	2.039504	up				
RNA165196|XLOC_007119	0.010706675	0.003399	2.039545	up				
RNA163111|XLOC_003453	1.0414E-05	2.15E-07	2.040134	up				
RNA157908|XLOC_000782	0.007171623	0.001948	2.040306	up				
RNA165093|XLOC_006757	0.024972707	0.010527	2.040548	up				
RNA159009|XLOC_009124	0.001906122	0.000309	2.041301	up				
RNA164066|XLOC_004629	0.004265248	0.000945	2.042341	up				
RNA161871|XLOC_001780	0.002359187	0.000413	2.04297	up				
RNA159067|XLOC_009485	0.014483942	0.005116	2.043711	up				
RNA160586|XLOC_012018	0.035055466	0.016324	2.043974	up				
RNA164157|XLOC_005143	0.002415227	0.000428	2.044319	up				
RNA158148|XLOC_000361	0.030828973	0.013814	2.045276	up				
RNA159627|XLOC_010312	0.004855716	0.001131	2.045936	up				
RNA160759|XLOC_012207	0.014293788	0.005028	2.04623	up				
RNA161710|XLOC_002300	0.001984095	0.000326	2.046714	up				
RNA160820|XLOC_012558	0.005118804	0.001219	2.04821	up				
RNA164300|XLOC_005290	0.009945848	0.003074	2.049884	up				
RNA157931|XLOC_000814	0.002664076	0.000491	2.050167	up				
RNA161413|XLOC_001414	0.004667949	0.00107	2.053119	up				
RNA160817|XLOC_012554	0.007816325	0.002202	2.053563	up				
RNA157880|XLOC_000757	0.000156852	1.03E-05	2.053598	up				
RNA159228|XLOC_009981	0.017663628	0.006666	2.055511	up				

RNA157926|XLOC_000160	0.016121779	0.005907	2.055522	up				
RNA157811|XLOC_000042	0.002947975	0.000566	2.056699	up				
RNA161743|XLOC_001668	0.019758545	0.007732	2.057043	up				
RNA164124|XLOC_005121	0.005518674	0.001349	2.05717	up				
RNA157845|XLOC_000077	0.01366953	0.00474	2.057906	up				
RNA159446|XLOC_009849	0.012707236	0.004282	2.059005	up				
RNA161714|XLOC_001649	0.01827634	0.00697	2.059439	up				
RNA158765|XLOC_008559	0.015977727	0.005835	2.059674	up				
RNA159810|XLOC_010672	0.001362787	0.000194	2.061033	up				
RNA164632|XLOC_005958	0.002713888	0.000504	2.061858	up				
RNA158946|XLOC_009075	0.007497422	0.002075	2.063395	up				
RNA161140|XLOC_013273	0.045557473	0.022724	2.064863	up				
RNA163069|XLOC_003417	0.002844406	0.000538	2.06511	up				
RNA160251|XLOC_011271	0.009015998	0.002683	2.0656	up				
RNA165748|XLOC_008116	0.023730416	0.009846	2.068479	up				
RNA161919|XLOC_001836	0.008631643	0.002527	2.068764	up				
RNA159262|XLOC_010013	0.007881181	0.00223	2.069545	up				
RNA164600|XLOC_005939	0.015678963	0.005691	2.070243	up				
RNA162532|XLOC_014219	0.022712613	0.009288	2.07036	up				
RNA159241|XLOC_009994	0.028173113	0.012286	2.070472	up				
RNA161783|XLOC_001701	0.02247973	0.009164	2.072669	up				
RNA164572|XLOC_005921	0.01719222	0.00643	2.074925	up				
RNA160764|XLOC_012216	0.010217645	0.003186	2.075769	up				
RNA162810|XLOC_002749	0.04270788	0.020946	2.076182	up				
RNA162639|XLOC_002592	0.005895696	0.001481	2.076659	up				
RNA164502|XLOC_005866	0.013269539	0.004553	2.076978	up				
RNA160743|XLOC_012184	3.80108E-05	1.38E-06	2.077313	up				
RNA165653|XLOC_007850	0.003162003	0.000624	2.077601	up				
RNA162560|XLOC_014390	0.000214849	1.56E-05	2.078459	up				
RNA165553|XLOC_007462	0.026605576	0.011411	2.079652	up				
RNA163657|XLOC_004292	0.006376245	0.001655	2.081315	up				
RNA165320|XLOC_006929	0.005793172	0.001445	2.081882	up				
RNA164002|XLOC_004573	0.004579973	0.001042	2.08337	up				
RNA161814|XLOC_001733	0.00354622	0.000731	2.0848	up				
RNA162222|XLOC_013577	0.010512138	0.003313	2.085491	up				
RNA160609|XLOC_012040	0.005177097	0.001239	2.085782	up				

RNA159426|XLOC_009830	0.025250522	0.010675	2.086086	up				
RNA162835|XLOC_002780	0.008133538	0.002327	2.086201	up				
RNA161587|XLOC_001550	0.00063741	6.84E-05	2.086462	up				
RNA161987|XLOC_001909	0.002363948	0.000415	2.086533	up				
RNA160920|XLOC_012769	0.005746798	0.001428	2.086711	up				
RNA165385|XLOC_007281	0.012664331	0.004263	2.087303	up				
RNA163487|XLOC_004161	0.006989877	0.00188	2.087361	up				
RNA159327|XLOC_009728	0.009744178	0.002985	2.087532	up				
RNA162220|XLOC_013808	0.000560956	5.75E-05	2.087786	up				
RNA160633|XLOC_011822	0.004983385	0.001173	2.087858	up				
RNA163067|XLOC_003411	0.005532833	0.001354	2.087977	up				
RNA161472|XLOC_001465	0.008199783	0.002354	2.088221	up				
RNA159140|XLOC_009543	0.023815451	0.009888	2.08969	up				
RNA161599|XLOC_002198	0.001453226	0.000212	2.090612	up				
RNA163474|XLOC_003772	0.012961417	0.004401	2.091971	up				
RNA162271|XLOC_013612	0.008180839	0.002345	2.094642	up				
RNA162403|XLOC_013945	0.002229111	0.000383	2.097517	up				
RNA163567|XLOC_003830	0.00293701	0.000563	2.101578	up				
RNA164784|XLOC_006110	0.005327237	0.001287	2.101755	up				
RNA164629|XLOC_006329	0.009365634	0.00283	2.105122	up				
RNA163489|XLOC_003780	0.011860434	0.003898	2.10546	up				
RNA158888|XLOC_008674	0.005840244	0.001462	2.107624	up				
RNA165087|XLOC_007036	0.011182342	0.003602	2.1098	up				
RNA159406|XLOC_009814	0.014581464	0.005164	2.110785	up				
RNA162953|XLOC_003318	0.000555105	5.65E-05	2.110873	up				
RNA162545|XLOC_014237	0.003576847	0.000739	2.114348	up				
RNA161119|XLOC_012999	0.02524698	0.010673	2.114539	up				
RNA159096|XLOC_009222	0.016551934	0.006121	2.114841	up				
RNA159880|XLOC_010497	0.010985109	0.003516	2.116239	up				
RNA160180|XLOC_011407	0.006750074	0.00179	2.116696	up				
RNA159470|XLOC_010190	0.022056036	0.008936	2.116954	up				
RNA164022|XLOC_005041	0.001155936	0.000154	2.11876	up				
RNA159503|XLOC_009896	0.006936622	0.001861	2.120964	up				
RNA159939|XLOC_010784	0.00754926	0.002095	2.123338	up				
RNA159814|XLOC_010458	0.001409828	0.000202	2.125447	up				
RNA165215|XLOC_007131	0.029657293	0.013161	2.127107	up				

RNA165798|XLOC_008164	0.001826446	0.000291	2.127276	up			
RNA164856|XLOC_006181	0.00422586	0.000933	2.127288	up			
RNA160674|XLOC_012385	0.007007496	0.001887	2.135105	up			
RNA164944|XLOC_006618	0.001824325	0.000291	2.139814	up			
RNA164689|XLOC_006385	0.008472737	0.002463	2.140508	up			
RNA161817|XLOC_002378	0.00165626	0.000254	2.141893	up			
RNA159119|XLOC_009254	0.008158304	0.002336	2.141926	up			
RNA159288|XLOC_010028	0.001114937	0.000147	2.142104	up			
RNA159525|XLOC_010229	0.010369993	0.003251	2.143294	up			
RNA162032|XLOC_013450	0.03272096	0.014924	2.146297	up			
RNA159592|XLOC_010275	0.006032158	0.001531	2.14635	up			
RNA161804|XLOC_002366	0.003384219	0.000686	2.149598	up			
RNA163701|XLOC_004764	0.00662835	0.001746	2.150061	up			
RNA158006|XLOC_000857	0.009467727	0.002873	2.150112	up			
RNA165322|XLOC_006931	0.004773925	0.001104	2.15037	up			
RNA165530|XLOC_007446	0.013878887	0.004838	2.150416	up			
RNA160522|XLOC_011743	0.03425946	0.015837	2.150593	up			
RNA160322|XLOC_011333	0.003345743	0.000676	2.151252	up			
RNA159220|XLOC_009345	0.000681913	7.51E-05	2.151325	up			
RNA162773|XLOC_003175	0.000712315	7.99E-05	2.1535	up			
RNA163938|XLOC_004514	0.003705532	0.000776	2.154764	up			
RNA159337|XLOC_010071	0.004250491	0.00094	2.155726	up			
RNA164596|XLOC_005552	0.004482336	0.001011	2.156253	up			
RNA164926|XLOC_006249	0.01303635	0.004436	2.156366	up			
RNA160847|XLOC_012312	0.001947952	0.000319	2.15748	up			
RNA164411|XLOC_005414	0.005536473	0.001355	2.158397	up			
RNA160418|XLOC_011633	0.011386469	0.003689	2.158975	up			
RNA161334|XLOC_001990	0.017731464	0.006702	2.160983	up			
RNA165402|XLOC_007306	0.007622155	0.002123	2.161545	up			
RNA158346|XLOC_000544	0.003932059	0.000845	2.162207	up			
RNA161213|XLOC_013099	0.004947069	0.00116	2.162426	up			
RNA159978|XLOC_010816	0.035866372	0.0168	2.166944	up			
RNA158654|XLOC_008466	0.003940105	0.000847	2.166985	up			
RNA165278|XLOC_006905	0.003819444	0.000811	2.167069	up			
RNA161409|XLOC_001411	0.01573786	0.005717	2.167213	up			
RNA159458|XLOC_009857	0.012948727	0.004395	2.167395	up			

RNA165248|XLOC_007168	0.013783359	0.004794	2.167871	up			
RNA161520|XLOC_002132	0.000472859	4.56E-05	2.168338	up			
RNA165720|XLOC_007908	0.00018214	1.26E-05	2.169098	up			
RNA158641|XLOC_008451	0.008378721	0.002425	2.169982	up			
RNA164698|XLOC_006028	0.001177865	0.000158	2.171888	up			
RNA162478|XLOC_014155	0.007826028	0.002207	2.172794	up			
RNA161039|XLOC_012877	0.003740025	0.000787	2.177211	up			
RNA160492|XLOC_011721	0.007912541	0.002243	2.18061	up			
RNA164342|XLOC_005338	0.013018586	0.004428	2.181537	up			
RNA161579|XLOC_001546	0.00785847	0.00222	2.183054	up			
RNA161398|XLOC_001401	0.000630486	6.74E-05	2.184364	up			
RNA158094|XLOC_000308	0.013247632	0.004543	2.186205	up			
RNA165930|XLOC_008317	0.006547892	0.001716	2.186601	up			
RNA160584|XLOC_011798	0.008142866	0.00233	2.187436	up			
RNA163705|XLOC_004327	0.016830055	0.006252	2.188314	up			
RNA164230|XLOC_005207	0.004835579	0.001124	2.188562	up			
RNA163113|XLOC_003455	0.042951945	0.021104	2.190487	up			
RNA162201|XLOC_013795	0.002939489	0.000564	2.191084	up			
RNA161009|XLOC_012693	0.026352147	0.011271	2.192209	up			
RNA160624|XLOC_012049	0.003025882	0.000588	2.192472	up			
RNA161003|XLOC_012686	0.006888562	0.001841	2.192898	up			
RNA161254|XLOC_001306	0.009254006	0.002781	2.198338	up			
RNA157954|XLOC_000185	0.009439362	0.002861	2.198422	up			
RNA164245|XLOC_005220	0.000759526	8.71E-05	2.200284	up			
RNA158695|XLOC_008833	0.006542572	0.001714	2.200789	up			
RNA158356|XLOC_001190	0.022744155	0.00931	2.201022	up			
RNA164915|XLOC_006239	0.003358034	0.000679	2.204341	up			
RNA159528|XLOC_009927	0.000303163	2.48E-05	2.205501	up			
RNA160022|XLOC_011052	0.000835401	9.86E-05	2.206195	up			
RNA159430|XLOC_010150	0.000445428	4.2E-05	2.206872	up			
RNA160146|XLOC_011157	0.02267016	0.009266	2.207466	up			
RNA162084|XLOC_013685	0.001609823	0.000244	2.208147	up			
RNA159183|XLOC_009308	0.007350102	0.002016	2.209329	up			
RNA163030|XLOC_003375	0.013951153	0.004872	2.209493	up			
RNA159578|XLOC_010264	0.002246872	0.000387	2.211318	up			
RNA161697|XLOC_002291	0.012252583	0.004075	2.212429	up			

RNA161264|XLOC_001934	0.005627979	0.001387	2.215183	up			
RNA159174|XLOC_009302	0.014116046	0.00495	2.215594	up			
RNA165251|XLOC_007170	0.006937199	0.001861	2.215907	up			
RNA160857|XLOC_012324	0.000846314	0.0001	2.218845	up			
RNA162439|XLOC_014114	0.025506426	0.010811	2.22151	up			
RNA161578|XLOC_001545	0.008095169	0.002313	2.222065	up			
RNA161169|XLOC_013037	0.005367318	0.0013	2.223048	up			
RNA162967|XLOC_002918	0.009115788	0.002725	2.223078	up			
RNA165633|XLOC_007537	0.001140675	0.000151	2.22328	up			
RNA160498|XLOC_011724	0.002775169	0.00052	2.223315	up			
RNA165801|XLOC_007982	0.012655918	0.004258	2.224508	up			
RNA164056|XLOC_005071	0.003785311	0.000801	2.225741	up			
RNA158919|XLOC_009038	0.020184537	0.00795	2.227935	up			
RNA165213|XLOC_006853	0.004183841	0.000919	2.229127	up			
RNA161736|XLOC_001664	0.008817743	0.002603	2.229132	up			
RNA161187|XLOC_013310	0.00447842	0.001009	2.230163	up			
RNA163638|XLOC_004273	0.000195137	1.37E-05	2.230592	up			
RNA163071|XLOC_003849	0.006685755	0.001765	2.230983	up			
RNA165325|XLOC_006933	0.00514367	0.001227	2.232741	up			
RNA164371|XLOC_005775	5.98434E-05	2.73E-06	2.233046	up			
RNA161910|XLOC_002473	0.020965097	0.00836	2.235773	up			
RNA162106|XLOC_013699	0.004552684	0.001033	2.236432	up			
RNA159394|XLOC_009805	0.016933976	0.0063	2.237473	up			
RNA158933|XLOC_009066	0.001430625	0.000207	2.237805	up			
RNA164324|XLOC_005322	0.001288605	0.000179	2.23801	up			
RNA158032|XLOC_000876	0.026523773	0.011366	2.238483	up			
RNA163181|XLOC_003505	0.00685931	0.001829	2.238574	up			
RNA157816|XLOC_000698	0.012924142	0.004383	2.239474	up			
RNA159858|XLOC_010706	0.00252392	0.000455	2.240524	up			
RNA161913|XLOC_001833	0.005865879	0.001471	2.242007	up			
RNA162992|XLOC_003346	0.002685761	0.000497	2.242507	up			
RNA162235|XLOC_013584	0.024684737	0.010361	2.244201	up			
RNA161233|XLOC_013162	0.001845804	0.000296	2.245142	up			
RNA158835|XLOC_008964	0.015947927	0.005821	2.24684	up			
RNA164365|XLOC_005365	0.005866028	0.001472	2.249902	up			
RNA164545|XLOC_005523	0.000874021	0.000105	2.250403	up			

RNA158410|XLOC_001230	0.004940508	0.001158	2.250943	up			
RNA159365|XLOC_009783	0.003720206	0.00078	2.253438	up			
RNA160310|XLOC_011326	0.000425892	3.96E-05	2.253768	up			
RNA164645|XLOC_006340	0.000348469	3E-05	2.256442	up			
RNA159883|XLOC_010500	0.006060805	0.001542	2.261159	up			
RNA162556|XLOC_014255	0.00251829	0.000453	2.262236	up			
RNA159636|XLOC_010540	0.005928346	0.001493	2.26274	up			
RNA164393|XLOC_005787	0.004352619	0.000972	2.27093	up			
RNA165284|XLOC_007193	0.004842257	0.001126	2.273415	up			
RNA160238|XLOC_011480	0.000490527	4.79E-05	2.275651	up			
RNA158278|XLOC_001117	0.004017217	0.000868	2.275704	up			
RNA158159|XLOC_000983	0.00791669	0.002244	2.276039	up			
RNA159837|XLOC_010469	0.00287886	0.000548	2.282034	up			
RNA162793|XLOC_002742	0.018734816	0.007194	2.282395	up			
RNA163735|XLOC_004799	0.0111656	0.003595	2.286026	up			
RNA162475|XLOC_014296	0.013717162	0.004763	2.286071	up			
RNA164916|XLOC_006597	0.009763134	0.002995	2.286465	up			
RNA163249|XLOC_003973	0.00862827	0.002525	2.287474	up			
RNA163392|XLOC_003704	0.004491312	0.001013	2.291327	up			
RNA163739|XLOC_004802	0.003193378	0.000633	2.294399	up			
RNA160994|XLOC_012848	0.007454275	0.002057	2.294743	up			
RNA164094|XLOC_004657	0.003425976	0.000697	2.297457	up			
RNA161046|XLOC_012716	0.034319934	0.015873	2.298492	up			
RNA165220|XLOC_006862	0.000304129	2.49E-05	2.299473	up			
RNA162559|XLOC_014257	0.012806537	0.004329	2.300074	up			
RNA160469|XLOC_011697	0.02634013	0.011265	2.300408	up			
RNA165769|XLOC_007952	0.003532104	0.000727	2.303512	up			
RNA158702|XLOC_008500	0.003360215	0.00068	2.304963	up			
RNA160262|XLOC_011281	0.000120133	7.26E-06	2.307001	up			
RNA158787|XLOC_008922	0.000413449	3.81E-05	2.309186	up			
RNA161171|XLOC_013297	0.0106949	0.003394	2.310031	up			
RNA161883|XLOC_002442	0.001575157	0.000238	2.312048	up			
RNA165505|XLOC_007419	0.000308946	2.54E-05	2.313027	up			
RNA165131|XLOC_007080	0.003424897	0.000697	2.315274	up			
RNA161126|XLOC_013265	0.010244794	0.003197	2.316944	up			
RNA164743|XLOC_006079	0.001284733	0.000179	2.317118	up			

RNA165623|XLOC_007530	0.000355979	3.09E-05	2.317566	up				
RNA161392|XLOC_002022	0.002941069	0.000564	2.318764	up				
RNA157836|XLOC_000061	0.000732491	8.31E-05	2.319734	up				
RNA163808|XLOC_004859	0.008108462	0.002318	2.320773	up				
RNA160470|XLOC_011699	0.011343605	0.003671	2.322434	up				
RNA165125|XLOC_007067	0.010372123	0.003252	2.329289	up				
RNA159544|XLOC_010240	0.006685755	0.001765	2.330751	up				
RNA165686|XLOC_007882	0.005512203	0.001347	2.331607	up				
RNA162158|XLOC_013766	0.000206889	1.49E-05	2.333102	up				
RNA158460|XLOC_001277	0.015968133	0.005831	2.335121	up				
RNA158103|XLOC_000933	0.001232282	0.000169	2.338244	up				
RNA165238|XLOC_007162	0.000501352	4.94E-05	2.339755	up				
RNA159937|XLOC_010985	0.023243846	0.009586	2.34053	up				
RNA159108|XLOC_009517	0.01730177	0.006485	2.341783	up				
RNA160863|XLOC_012586	0.003396954	0.000689	2.342836	up				
RNA163029|XLOC_003374	0.000413449	3.81E-05	2.342862	up				
RNA162315|XLOC_013876	0.005139675	0.001225	2.344142	up				
RNA161388|XLOC_002019	0.002873651	0.000546	2.345758	up				
RNA160968|XLOC_012663	0.03225629	0.014644	2.345972	up				
RNA161828|XLOC_001746	0.01286881	0.004357	2.347874	up				
RNA160063|XLOC_011092	0.003914499	0.000838	2.348102	up				
RNA164233|XLOC_005209	0.019625433	0.007662	2.34972	up				
RNA162792|XLOC_003194	0.004580345	0.001042	2.351445	up				
RNA160338|XLOC_011570	0.000535056	5.38E-05	2.352048	up				
RNA159733|XLOC_010397	0.002373617	0.000417	2.353434	up				
RNA161382|XLOC_002013	0.007876568	0.002228	2.35786	up				
RNA160319|XLOC_011551	0.021619301	0.008707	2.362031	up				
RNA163678|XLOC_004309	0.015199428	0.005452	2.364531	up				
RNA165283|XLOC_007192	0.006520381	0.001706	2.365426	up				
RNA159042|XLOC_009464	0.010418348	0.003271	2.366214	up				
RNA164472|XLOC_005473	0.00155156	0.000233	2.369488	up				
RNA158646|XLOC_008781	0.006427215	0.001672	2.38479	up				
RNA163552|XLOC_003820	0.006073652	0.001546	2.392162	up				
RNA162079|XLOC_013680	0.00060804	6.41E-05	2.392474	up				
RNA161201|XLOC_013337	0.000475955	4.6E-05	2.392928	up				
RNA160860|XLOC_012333	0.002819168	0.000532	2.393518	up				

RNA159805|XLOC_010670	0.000839468	9.92E-05	2.39441	up				
RNA162691|XLOC_003114	0.002191335	0.000374	2.39945	up				
RNA165256|XLOC_006886	0.004922401	0.001152	2.399846	up				
RNA161870|XLOC_002424	0.005843033	0.001463	2.401323	up				
RNA162975|XLOC_003330	0.002549357	0.000461	2.401839	up				
RNA161085|XLOC_012912	0.002855942	0.000542	2.404992	up				
RNA159371|XLOC_010109	7.1007E-05	3.49E-06	2.407556	up				
RNA165210|XLOC_007125	0.009516215	0.002894	2.411427	up				
RNA165569|XLOC_007795	0.008219942	0.002361	2.415247	up				
RNA159190|XLOC_009319	0.002971159	0.000572	2.416413	up				
RNA160017|XLOC_010851	0.026448663	0.011323	2.417671	up				
RNA160318|XLOC_011550	0.014302554	0.005031	2.418663	up				
RNA165711|XLOC_007614	0.008493556	0.002472	2.419221	up				
RNA162931|XLOC_002886	0.002378186	0.000419	2.422298	up				
RNA164065|XLOC_005081	0.001651033	0.000253	2.427022	up				
RNA165926|XLOC_008285	0.004265198	0.000945	2.430072	up				
RNA159315|XLOC_010054	0.001779834	0.000281	2.433667	up				
RNA164565|XLOC_005534	0.013456759	0.004639	2.433801	up				
RNA163856|XLOC_004441	0.00454124	0.00103	2.435094	up				
RNA158819|XLOC_008616	0.004703505	0.001081	2.439427	up				
RNA165285|XLOC_006908	0.000200068	1.42E-05	2.458164	up				
RNA165577|XLOC_007806	0.002416542	0.000428	2.460965	up				
RNA160717|XLOC_012428	0.002618841	0.00048	2.462604	up				
RNA159456|XLOC_010176	0.000604066	6.36E-05	2.465372	up				
RNA162515|XLOC_014207	0.001139086	0.000151	2.466014	up				
RNA164534|XLOC_005893	0.000238442	1.8E-05	2.469801	up				
RNA164399|XLOC_005791	0.00614172	0.00157	2.477315	up				
RNA160657|XLOC_012362	0.000545507	5.53E-05	2.477894	up				
RNA165309|XLOC_006922	0.001984325	0.000326	2.483121	up				
RNA164182|XLOC_005607	0.009207926	0.002763	2.483419	up				
RNA163974|XLOC_004991	0.000333025	2.82E-05	2.484826	up				
RNA161957|XLOC_001877	0.009351904	0.002825	2.487201	up				
RNA161323|XLOC_001342	0.016668484	0.006175	2.491575	up				
RNA160718|XLOC_012429	0.016481236	0.006085	2.496723	up				
RNA160888|XLOC_012608	0.000128554	7.95E-06	2.502002	up				
RNA157924|XLOC_000796	0.003113769	0.00061	2.506925	up				

RNA163400|XLOC_003712	0.001991894	0.000328	2.507325	up			
RNA162836|XLOC_003225	0.002672427	0.000493	2.514718	up			
RNA163702|XLOC_004325	0.001706961	0.000265	2.515467	up			
RNA159229|XLOC_009622	0.008925571	0.002647	2.516715	up			
RNA161457|XLOC_001453	0.006185438	0.001586	2.524794	up			
RNA165098|XLOC_006764	0.01189866	0.003916	2.526624	up			
RNA158557|XLOC_008382	0.007110502	0.001926	2.530826	up			
RNA159079|XLOC_009497	0.000665142	7.24E-05	2.536939	up			
RNA161799|XLOC_002360	0.009114753	0.002725	2.537293	up			
RNA163993|XLOC_004562	0.017843734	0.00676	2.540863	up			
RNA162273|XLOC_013845	0.004850844	0.001128	2.545085	up			
RNA161000|XLOC_012685	0.020763334	0.008254	2.545358	up			
RNA164547|XLOC_005900	0.009469862	0.002874	2.546574	up			
RNA161368|XLOC_001376	0.000748959	8.54E-05	2.551295	up			
RNA164977|XLOC_006301	0.033234637	0.015233	2.553829	up			
RNA162137|XLOC_013734	0.000565304	5.81E-05	2.558755	up			
RNA161628|XLOC_001595	0.046126127	0.023073	2.562164	up			
RNA160618|XLOC_012045	0.00036426	3.19E-05	2.564602	up			
RNA160984|XLOC_012841	0.005074914	0.001203	2.575734	up			
RNA163794|XLOC_004846	0.005411156	0.001313	2.590148	up			
RNA162998|XLOC_003350	0.012277398	0.004086	2.595197	up			
RNA165311|XLOC_006924	0.003299481	0.000663	2.60019	up			
RNA162312|XLOC_014016	0.010345769	0.003241	2.6013	up			
RNA165107|XLOC_007053	0.000341365	2.91E-05	2.603841	up			
RNA163916|XLOC_004495	0.001330931	0.000187	2.607846	up			
RNA157856|XLOC_000091	0.012442504	0.004164	2.609287	up			
RNA160342|XLOC_011346	0.011989027	0.003957	2.614079	up			
RNA160525|XLOC_011745	0.002840862	0.000537	2.615013	up			
RNA162641|XLOC_003063	0.00173559	0.000271	2.615401	up			
RNA160216|XLOC_011222	0.01237883	0.004133	2.617523	up			
RNA159862|XLOC_010709	7.58903E-05	3.84E-06	2.636682	up			
RNA162761|XLOC_002717	0.000839306	9.92E-05	2.637676	up			
RNA163048|XLOC_002988	0.002633212	0.000483	2.64518	up			
RNA162851|XLOC_002801	0.014547459	0.005148	2.651282	up			
RNA161832|XLOC_002390	0.000491597	4.81E-05	2.660202	up			
RNA157894|XLOC_000768	0.006326188	0.001638	2.6637	up			

RNA164082|XLOC_005087	0.006670485	0.00176	2.666131	up			
RNA159023|XLOC_009142	0.015054525	0.005385	2.683085	up			
RNA163663|XLOC_004737	0.004791043	0.001109	2.683799	up			
RNA159942|XLOC_010785	0.004626029	0.001056	2.69489	up			
RNA158688|XLOC_008829	0.005244761	0.001261	2.697432	up			
RNA164389|XLOC_005392	2.4783E-05	7.67E-07	2.706201	up			
RNA161504|XLOC_002116	0.001727652	0.00027	2.710518	up			
RNA160558|XLOC_012002	3.82483E-05	1.39E-06	2.712222	up			
RNA162902|XLOC_002866	0.000871587	0.000105	2.715451	up			
RNA159512|XLOC_010215	0.00808666	0.002309	2.733321	up			
RNA157922|XLOC_000155	0.001447889	0.000211	2.734767	up			
RNA160707|XLOC_012142	0.000805469	9.4E-05	2.735693	up			
RNA162156|XLOC_013763	0.008862042	0.00262	2.74598	up			
RNA162882|XLOC_002840	0.02362644	0.009791	2.74954	up			
RNA159838|XLOC_010688	0.024909854	0.010488	2.751479	up			
RNA165357|XLOC_007622	0.004756188	0.001098	2.765172	up			
RNA163345|XLOC_003675	0.00601044	0.001523	2.771844	up			
RNA161567|XLOC_002173	0.000516988	5.15E-05	2.785061	up			
RNA162781|XLOC_003181	0.006991732	0.001881	2.789478	up			
RNA159851|XLOC_010699	0.002854551	0.000541	2.789674	up			
RNA164794|XLOC_006118	0.000420315	3.89E-05	2.796529	up			
RNA159846|XLOC_010695	0.000866293	0.000104	2.797139	up			
RNA164664|XLOC_005989	0.015202128	0.005454	2.798736	up			
RNA164241|XLOC_005217	0.001505779	0.000223	2.80649	up			
RNA164299|XLOC_005289	0.001215598	0.000165	2.809296	up			
RNA165743|XLOC_008111	0.002878823	0.000547	2.810128	up			
RNA161595|XLOC_002196	0.000429593	4.01E-05	2.818795	up			
RNA165923|XLOC_008282	0.002140522	0.000362	2.82632	up			
RNA165149|XLOC_007089	0.007350877	0.002016	2.834327	up			
RNA157897|XLOC_000771	0.000297091	2.41E-05	2.85217	up			
RNA162161|XLOC_013770	0.000353726	3.07E-05	2.854582	up			
RNA159637|XLOC_010541	0.004702146	0.001081	2.865126	up			
RNA159305|XLOC_010048	0.014439795	0.005095	2.866921	up			
RNA165265|XLOC_007177	0.005459967	0.001331	2.867549	up			
RNA160995|XLOC_012849	0.010300867	0.003221	2.868434	up			
RNA160360|XLOC_011360	0.00276983	0.000519	2.873051	up			

RNA164786|XLOC_006459	0.004331816	0.000966	2.883608	up			
RNA165796|XLOC_007978	0.009902471	0.003054	2.886389	up			
RNA164840|XLOC_006516	0.004034028	0.000874	2.888009	up			
RNA159162|XLOC_009564	0.007181773	0.001953	2.890323	up			
RNA161951|XLOC_002497	0.010154324	0.003162	2.890639	up			
RNA165307|XLOC_007215	0.007561036	0.002099	2.895969	up			
RNA162407|XLOC_013947	0.006699421	0.001771	2.900357	up			
RNA162946|XLOC_003312	0.001427117	0.000206	2.910979	up			
RNA164804|XLOC_006128	0.003873901	0.000827	2.91497	up			
RNA161683|XLOC_001630	0.000542161	5.48E-05	2.930903	up			
RNA161803|XLOC_001716	0.00786288	0.002222	2.955548	up			
RNA164368|XLOC_005366	0.001736712	0.000272	2.977128	up			
RNA159457|XLOC_009856	0.000747907	8.52E-05	2.981547	up			
RNA161483|XLOC_001476	0.009176608	0.00275	2.996114	up			
RNA165305|XLOC_007213	0.00073093	8.28E-05	3.022808	up			
RNA165493|XLOC_007739	0.020500736	0.008117	3.024516	up			
RNA165310|XLOC_006923	0.001689029	0.000261	3.03711	up			
RNA161838|XLOC_002392	0.001065411	0.000138	3.042785	up			
RNA163049|XLOC_002989	0.004141362	0.000905	3.087608	up			
RNA164942|XLOC_006270	0.007302825	0.001999	3.088187	up			
RNA165306|XLOC_007214	0.001448425	0.000211	3.089641	up			
RNA162922|XLOC_002882	0.000596297	6.25E-05	3.089847	up			
RNA159454|XLOC_009855	0.000694668	7.71E-05	3.098209	up			
RNA160547|XLOC_011763	0.004970782	0.001168	3.100016	up			
RNA161860|XLOC_001773	0.01728051	0.006473	3.148794	up			
RNA161795|XLOC_001711	0.005236247	0.001258	3.152612	up			
RNA165427|XLOC_007680	0.0054988	0.001343	3.171154	up			
RNA165364|XLOC_007265	0.001002442	0.000127	3.186027	up			
RNA164325|XLOC_005323	0.000304416	2.49E-05	3.1865	up			
RNA158248|XLOC_000456	0.002424823	0.00043	3.204851	up			
RNA157902|XLOC_000777	0.00406795	0.000884	3.231973	up			
RNA160948|XLOC_012796	0.01203525	0.003978	3.24052	up			
RNA158024|XLOC_000255	0.000630477	6.74E-05	3.2489	up			
RNA161124|XLOC_013007	0.01196768	0.003947	3.249841	up			
RNA161320|XLOC_001983	0.00261282	0.000478	3.258946	up			
RNA163674|XLOC_004306	0.0078452	0.002215	3.267449	up			

RNA160320|XLOC_011331	0.00091109	0.000111	3.296566	up			
RNA164525|XLOC_005511	0.000216761	1.58E-05	3.299166	up			
RNA162996|XLOC_002943	0.0061753	0.001582	3.315739	up			
RNA165737|XLOC_008109	0.001556381	0.000234	3.32357	up			
RNA165875|XLOC_008237	7.95905E-05	4.11E-06	3.335784	up			
RNA162933|XLOC_002888	0.005057193	0.001197	3.337106	up			
RNA160855|XLOC_012322	0.000671072	7.35E-05	3.339946	up			
RNA165712|XLOC_007896	0.000240703	1.82E-05	3.378085	up			
RNA161601|XLOC_002204	0.000911684	0.000111	3.379484	up			
RNA165192|XLOC_007116	0.013680525	0.004745	3.380075	up			
RNA164207|XLOC_005623	0.00230175	0.0004	3.393153	up			
RNA163248|XLOC_003562	0.002765442	0.000518	3.398906	up			
RNA157927|XLOC_000162	3.91099E-06	4.97E-08	3.457281	up			
RNA160835|XLOC_012568	3.82507E-06	4.82E-08	3.501882	up			
RNA164419|XLOC_005425	0.006901056	0.001847	3.505115	up			
RNA160969|XLOC_012664	0.002996166	0.000579	3.53411	up			
RNA164691|XLOC_006019	0.025709309	0.010921	3.59306	up			
RNA164329|XLOC_005326	0.000171167	1.15E-05	3.595244	up			
RNA165208|XLOC_007124	0.002040658	0.000339	3.788633	up			
RNA158289|XLOC_000494	0.000708686	7.93E-05	3.87064	up			
RNA164595|XLOC_005935	8.3177E-06	1.55E-07	3.891853	up			
RNA163325|XLOC_003653	0.005739904	0.001426	3.905798	up			
RNA163867|XLOC_004451	0.00035587	3.09E-05	3.928218	up			
RNA159725|XLOC_010390	0.002272529	0.000393	3.950745	up			
RNA165127|XLOC_007077	0.008380121	0.002425	4.083202	up			
RNA160316|XLOC_011548	0.00036188	3.16E-05	4.084599	up			
RNA162162|XLOC_013771	0.002935243	0.000562	4.135304	up			
RNA162171|XLOC_013549	0.001759842	0.000276	4.153924	up			
RNA163299|XLOC_003629	0.001373995	0.000196	4.242733	up			
RNA160208|XLOC_011215	0.003824404	0.000813	4.269345	up			
RNA160998|XLOC_012684	0.000792349	9.21E-05	4.282859	up			
RNA164425|XLOC_005433	0.007189271	0.001956	4.581765	up			
RNA159364|XLOC_009782	0.001209286	0.000164	4.60843	up			
RNA165483|XLOC_007734	0.003284538	0.000659	4.693991	up			
RNA164432|XLOC_005810	6.34234E-06	1.03E-07	4.756653	up			
RNA157822|XLOC_000702	0.001179505	0.000158	4.870056	up			

RNA165259|XLOC_006890	0.012987996	0.004414	5.045724	up			
RNA160997|XLOC_012683	8.84758E-05	4.8E-06	5.105635	up			
RNA163019|XLOC_003364	0.006057425	0.001541	5.302031	up			
RNA159414|XLOC_009820	0.003095669	0.000606	5.384716	up			
RNA160972|XLOC_012827	0.000488993	4.77E-05	5.672297	up			
RNA160452|XLOC_011677	0.001767404	0.000278	5.829276	up			
RNA160996|XLOC_012682	0.000231171	1.72E-05	6.770382	up			
RNA158940|XLOC_009378	0.00020194	1.44E-05	11.04028	up			
RNA161731|XLOC_001659	0.002040658	0.000339	11.82876	up			
RNA166054|ENST00000493911	0.032240056	0.014634	2.217898	up			
RNA166038|ENST00000474745	0.013291652	0.004565	2.299671	up			
RNA166032|ENST00000487797	0.01716409	0.006415	2.94209	up			
RNA166111|ENST00000470010	0.013693831	0.004751	2.969797	up			
RNA166001|ENST00000485443	0.007577375	0.002105	3.395108	up			
RNA165966|ENST00000478115	0.005048656	0.001194	3.397718	up			
RNA166062|ENST00000483424	0.00704052	0.001899	4.604212	up			
RNA166135|ENST00000465779	0.012559928	0.004214	4.909412	up			
RNA166004|ENST00000496828	0.004936131	0.001156	5.087182	up			
RNA54549|H-InvDB_1872_321	0.039052773	0.018699	2.849818	up			
RNA54121|H-InvDB_1417_369	0.032962453	0.01507	3.362768	up			
RNA54677|H-InvDB_2009_306	0.013543148	0.004682	3.424273	up			
RNA54533|H-InvDB_1856_321	0.001831122	0.000292	4.830445	up			
RNA53066|H-InvDB_325_665	0.00039725	3.6E-05	8.169448	up			
RNA53770|H-InvDB_1048_426	0.007433991	0.00205	8.403696	up			
RNA53092|H-InvDB_351_656	0.006353707	0.001647	8.813622	up			
RNA53710|H-InvDB_987_441	0.002274747	0.000393	9.121472	up			
RNA53109|H-InvDB_368_650	0.006280354	0.001622	9.360508	up			
RNA53071|H-InvDB_330_662	0.003928649	0.000844	9.825444	up			
RNA61062|RNAz_1776_275	0.003147965	0.00062	2.000254	up			
RNA34276|NRED_407_2336	0.022995515	0.009451	2.000534	up			
RNA41179|RefSeq_3301_506	0.002363189	0.000414	2.001712	up			
RNA54423|H-InvDB_1736_331	0.048571985	0.024624	2.00214	up			
RNA96496|EvoFold_323_239	0.002882386	0.000548	2.002722	up			
RNA57692|UCRs_7_489	0.001785384	0.000282	2.003319	up			
RNA64888|RNAz_5609_200	0.00169	0.000261	2.003526	up			
RNA46171|UCSC_4561_2093	0.018480549	0.007074	2.004093	up			

RNA61900|RNAz_2615_245	0.005868747	0.001473	2.004397	up			
RNA61300|RNAz_2014_271	0.000320946	2.68E-05	2.004413	up			
RNA58221|CombinedLit_123_1257	0.013126693	0.004483	2.004467	up			
RNA39184|RefSeq_1149_2566	0.018753193	0.007203	2.005001	up			
RNA51363|UCSC_10874_554	0.00118177	0.000159	2.007832	up			
RNA58916|asoverlaps_530_1643	0.02499604	0.010538	2.00822	up			
RNA62797|RNAz_3515_236	0.002380194	0.000419	2.008776	up			
RNA40576|RefSeq_2641_1142	0.003170288	0.000626	2.008834	up			
RNA34581|NRED_713_1773	0.001529172	0.000228	2.009041	up			
RNA59406|RNAz_117_440	0.001418175	0.000204	2.009303	up			
RNA61569|RNAz_2283_262	0.023244552	0.009587	2.009481	up			
RNA64392|RNAz_5113_200	0.02402447	0.009997	2.011285	up			
RNA61252|RNAz_1966_272	0.009203564	0.002761	2.012376	up			
RNA55660|H-InvDB_3046_205	0.001626383	0.000248	2.013058	up			
RNA62046|RNAz_2761_240	7.58804E-05	3.84E-06	2.013187	up			
RNA63313|RNAz_4031_229	0.04592989	0.022959	2.013834	up			
RNA50326|UCSC_9594_919	0.001039765	0.000134	2.014307	up			
RNA63210|RNAz_3928_231	0.010782698	0.003428	2.014437	up			
RNA59713|RNAz_425_359	0.005838158	0.001461	2.014764	up			
RNA147027|nc-HOXA6-72	0.001349892	0.000191	2.014945	up			
RNA60454|RNAz_1166_300	0.002547202	0.000461	2.015386	up			
RNA50560|UCSC_9875_843	0.007074357	0.001912	2.015713	up			
RNA62054|RNAz_2769_240	0.007589622	0.00211	2.015767	up			
RNA96247|EvoFold_58_306	0.024357723	0.010182	2.016304	up			
RNA63225|RNAz_3943_230	0.009987236	0.003091	2.016581	up			
RNA60288|RNAz_1000_310	0.005707008	0.001414	2.017742	up			
RNA51591|UCSC_11162_460	1.39774E-05	3.29E-07	2.019767	up			
RNA62537|RNAz_3255_238	0.013483809	0.004652	2.02009	up			
RNA44634|UCSC_2634_2791	0.002386316	0.000421	2.021201	up			
RNA60723|RNAz_1437_280	0.016473683	0.006081	2.021442	up			
RNA64126|RNAz_4846_209	0.000366112	3.21E-05	2.022065	up			
RNA61140|RNAz_1854_274	0.008543049	0.002491	2.022299	up			
RNA62274|RNAz_2992_239	0.004302687	0.000957	2.023035	up			
RNA61286|RNAz_2000_271	0.008084156	0.002308	2.023569	up			
RNA33903|NRED_33_5369	0.013457852	0.00464	2.024148	up			
RNA61374|RNAz_2088_268	0.000679675	7.48E-05	2.024383	up			

RNA61588|RNAz_2302_261	0.027869148	0.012113	2.024779	up			
RNA147143|nc-HOXC8-147	0.010467295	0.003292	2.025343	up			
RNA64448|RNAz_5169_200	0.017438525	0.006556	2.025966	up			
RNA57959|UCRs_274_235	0.020076547	0.007893	2.026007	up			
RNA60744|RNAz_1458_280	0.033985414	0.015673	2.026531	up			
RNA62863|RNAz_3581_235	0.003721	0.000781	2.026959	up			
RNA51349|UCSC_10857_557	0.010899167	0.003479	2.027376	up			
RNA61433|RNAz_2147_267	0.010668998	0.003382	2.027881	up			
RNA52017|UCSC_11633_280	0.021122769	0.008438	2.02953	up			
RNA64169|RNAz_4889_208	0.011452934	0.003718	2.030553	up			
RNA59636|RNAz_347_372	0.005567205	0.001366	2.030826	up			
RNA44688|UCSC_2704_2760	0.013798905	0.004801	2.034014	up			
RNA61447|RNAz_2161_266	0.000364974	3.2E-05	2.034601	up			
RNA64810|RNAz_5531_200	0.001079166	0.000141	2.035464	up			
RNA64011|RNAz_4731_213	0.002906419	0.000555	2.035517	up			
RNA62598|RNAz_3316_237	0.006131918	0.001567	2.035693	up			
RNA58987|asoverlaps_620_1422	0.048939742	0.024858	2.036649	up			
RNA60028|RNAz_740_320	0.006611107	0.001739	2.037294	up			
RNA64050|RNAz_4770_212	0.024051601	0.010011	2.037865	up			
RNA61360|RNAz_2074_269	0.03791647	0.018015	2.039337	up			
RNA62806|RNAz_3524_236	0.003930444	0.000844	2.040935	up			
RNA64583|RNAz_5304_200	0.008110008	0.002318	2.044804	up			
RNA63888|RNAz_4608_217	0.008724825	0.002565	2.045234	up			
RNA63951|RNAz_4671_215	0.000406971	3.73E-05	2.047215	up			
RNA60645|RNAz_1358_280	0.007369348	0.002023	2.047219	up			
RNA59829|RNAz_541_349	0.021243317	0.008504	2.047389	up			
RNA59543|RNAz_254_395	0.000426324	3.96E-05	2.047416	up			
RNA63228|RNAz_3946_230	0.004716065	0.001085	2.047823	up			
RNA50756|UCSC_10134_763	0.002563261	0.000465	2.049116	up			
RNA59617|RNAz_328_379	0.000942772	0.000117	2.049271	up			
RNA51070|UCSC_10502_658	0.005830038	0.001458	2.04964	up			
RNA61463|RNAz_2177_266	0.00113304	0.00015	2.050064	up			
RNA147028|hox-HOXA7-15	0.002960629	0.000569	2.050712	up			
RNA61788|RNAz_2502_252	0.010627237	0.003363	2.054388	up			
RNA51041|UCSC_10463_671	0.021365514	0.008573	2.054476	up			
RNA49614|UCSC_8731_1108	0.044278495	0.02193	2.055481	up			

RNA64618|RNAz_5339_200	0.003878942	0.000828	2.056376	up			
RNA47851|UCSC_6612_1625	0.004247491	0.000939	2.056377	up			
RNA64269|RNAz_4990_205	0.024372565	0.010191	2.057423	up			
RNA47185|UCSC_5790_1811	0.017545998	0.006609	2.057554	up			
RNA63924|RNAz_4644_216	0.018059827	0.006865	2.057657	up			
RNA59096|asoverlaps_765_986	0.01510828	0.005408	2.058404	up			
RNA147235|nc-HOXB4-167	0.001752289	0.000275	2.059516	up			
RNA96349|EvoFold_168_270	8.54698E-05	4.56E-06	2.059562	up			
RNA59114|asoverlaps_788_946	0.005578462	0.001371	2.059844	up			
RNA40804|RefSeq_2888_923	7.44017E-05	3.73E-06	2.060268	up			
RNA44257|UCSC_2171_3067	0.012943685	0.004392	2.060665	up			
RNA47245|UCSC_5856_1796	0.000958533	0.000119	2.06093	up			
RNA50264|UCSC_9514_935	0.020337438	0.008028	2.061907	up			
RNA64400|RNAz_5121_200	0.000673163	7.38E-05	2.061968	up			
RNA51651|UCSC_11230_437	0.038866688	0.018595	2.062979	up			
RNA51329|UCSC_10833_564	0.004137961	0.000904	2.063319	up			
RNA62493|RNAz_3211_238	0.006596097	0.001734	2.063331	up			
RNA60224|RNAz_936_313	0.002525829	0.000455	2.0642	up			
RNA60302|RNAz_1014_309	0.011791904	0.003866	2.064498	up			
RNA63581|RNAz_4301_223	0.014872553	0.0053	2.065239	up			
RNA60450|RNAz_1162_300	0.03680515	0.017361	2.065844	up			
RNA62284|RNAz_3002_239	0.020849131	0.008299	2.066012	up			
RNA64072|RNAz_4792_211	0.002362679	0.000414	2.067229	up			
RNA55559|H-InvDB_2940_213	0.005872727	0.001474	2.067236	up			
RNA47866|UCSC_6628_1620	0.006222379	0.0016	2.067652	up			
RNA61747|RNAz_2461_253	0.011123343	0.003576	2.068392	up			
RNA58986|asoverlaps_619_1423	0.000364925	3.19E-05	2.068984	up			
RNA57887|UCRs_202_252	0.014654127	0.005198	2.069775	up			
RNA59966|RNAz_678_329	0.026076987	0.011122	2.069881	up			
RNA58895|asoverlaps_499_1705	5.08797E-05	2.13E-06	2.069934	up			
RNA96645|EvoFold_486_213	0.014754032	0.005244	2.070331	up			
RNA43520|UCSC_1271_3740	0.002567195	0.000466	2.071153	up			
RNA63746|RNAz_4466_220	0.000346865	2.98E-05	2.071482	up			
RNA54709|H-InvDB_2043_301	0.009261271	0.002784	2.072363	up			
RNA96281|EvoFold_92_292	0.006439232	0.001676	2.072454	up			
RNA62706|RNAz_3424_237	0.0483917	0.024501	2.076695	up			

RNA53565|H-InvDB_836_492	0.01807397	0.006872	2.078065	up			
RNA62755|RNAz_3473_236	0.001746062	0.000273	2.078982	up			
RNA57915|UCRs_230_246	0.009085304	0.00271	2.079052	up			
RNA63715|RNAz_4435_221	0.02705558	0.011666	2.079353	up			
RNA64560|RNAz_5281_200	0.001045259	0.000135	2.080017	up			
RNA146946|nc-HOXD4-36	0.00812128	0.002323	2.080206	up			
RNA62477|RNAz_3195_238	0.002099583	0.000353	2.080552	up			
RNA45551|UCSC_3786_2322	0.000657441	7.13E-05	2.080597	up			
RNA59915|RNAz_627_337	0.01322748	0.004531	2.080992	up			
RNA62351|RNAz_3069_239	0.001306553	0.000183	2.081786	up			
RNA60118|RNAz_830_317	0.000146056	9.39E-06	2.082086	up			
RNA61660|RNAz_2374_258	0.023501767	0.009723	2.083176	up			
RNA59799|RNAz_511_353	0.0013957	0.0002	2.08332	up			
RNA61989|RNAz_2704_240	0.006699016	0.00177	2.083864	up			
RNA96458|EvoFold_283_245	0.002055081	0.000343	2.085074	up			
RNA39915|RefSeq_1936_1794	0.001376773	0.000196	2.086448	up			
RNA50428|UCSC_9716_885	0.004855823	0.001131	2.087696	up			
RNA48233|UCSC_7085_1514	0.013687229	0.004748	2.087728	up			
RNA57781|UCRs_96_299	0.02428293	0.010136	2.087835	up			
RNA60250|RNAz_962_312	0.004955736	0.001162	2.089486	up			
RNA62252|RNAz_2970_239	0.004636517	0.00106	2.090177	up			
RNA47206|UCSC_5814_1806	0.017901838	0.006787	2.091895	up			
RNA60351|RNAz_1063_306	0.018049499	0.00686	2.092905	up			
RNA62062|RNAz_2777_240	0.003742806	0.000788	2.093594	up			
RNA48142|UCSC_6966_1545	0.001852163	0.000297	2.09399	up			
RNA62298|RNAz_3016_239	0.005509801	0.001346	2.09534	up			
RNA46727|UCSC_5248_1937	0.00092842	0.000114	2.095386	up			
RNA60825|RNAz_1539_279	0.003091036	0.000604	2.096497	up			
RNA60565|RNAz_1277_289	0.014542361	0.005145	2.097462	up			
RNA58867|asoverlaps_467_1774	0.004933544	0.001156	2.098655	up			
RNA38641|RefSeq_569_3670	0.001632067	0.000249	2.098791	up			
RNA61214|RNAz_1928_273	0.007200189	0.00196	2.100778	up			
RNA43838|UCSC_1655_3431	0.006536084	0.001712	2.103171	up			
RNA62503|RNAz_3221_238	0.004089488	0.00089	2.103876	up			
RNA61412|RNAz_2126_267	0.006073981	0.001547	2.104333	up			
RNA63415|RNAz_4133_227	0.007666076	0.002141	2.104503	up			

RNA61802|RNAz_2516_251	0.02067744	0.008209	2.104593	up			
RNA62693|RNAz_3411_237	0.001829121	0.000292	2.105203	up			
RNA61484|RNAz_2198_265	0.000509341	5.04E-05	2.105504	up			
RNA51222|UCSC_10697_604	0.00410813	0.000896	2.105957	up			
RNA63413|RNAz_4131_227	0.003695114	0.000773	2.10631	up			
RNA62322|RNAz_3040_239	0.015593753	0.005651	2.107238	up			
RNA47105|UCSC_5698_1830	0.04096093	0.019854	2.114394	up			
RNA64679|RNAz_5400_200	0.011192894	0.003606	2.114459	up			
RNA58903|asoverlaps_509_1687	0.025169216	0.010635	2.114645	up			
RNA60092|RNAz_804_318	0.017309325	0.006489	2.114831	up			
RNA63250|RNAz_3968_230	0.011389522	0.00369	2.116914	up			
RNA59973|RNAz_685_328	0.005939738	0.001498	2.117157	up			
RNA50265|UCSC_9515_935	0.000633934	6.79E-05	2.117242	up			
RNA47511|UCSC_6189_1720	0.003192659	0.000632	2.117817	up			
RNA61212|RNAz_1926_273	0.000471441	4.54E-05	2.118342	up			
RNA61287|RNAz_2001_271	0.000141176	8.99E-06	2.11862	up			
RNA45766|UCSC_4066_2233	0.001012461	0.000129	2.121055	up			
RNA50119|UCSC_9332_978	0.001008169	0.000128	2.122155	up			
RNA62170|RNAz_2888_240	0.037602037	0.017836	2.122347	up			
RNA49253|UCSC_8292_1208	0.007499381	0.002076	2.124015	up			
RNA61251|RNAz_1965_272	0.007495171	0.002075	2.12419	up			
RNA64058|RNAz_4778_211	0.009576329	0.002918	2.125209	up			
RNA61794|RNAz_2508_251	0.001845804	0.000295	2.126593	up			
RNA54094|H-InvDB_1388_373	0.024345573	0.010173	2.126618	up			
RNA39313|RefSeq_1285_2415	0.011543441	0.003756	2.127078	up			
RNA40353|RefSeq_2399_1388	0.004960428	0.001164	2.128152	up			
RNA63607|RNAz_4327_223	0.005006215	0.00118	2.133451	up			
RNA62409|RNAz_3127_239	0.007359522	0.00202	2.133794	up			
RNA40229|RefSeq_2267_1518	0.001694645	0.000262	2.134455	up			
RNA64397|RNAz_5118_200	0.001389975	0.000199	2.134836	up			
RNA59380|RNAz_91_461	0.000349744	3.02E-05	2.135409	up			
RNA34026|NRED_157_3330	0.011429437	0.003706	2.138173	up			
RNA43961|UCSC_1806_3331	0.01913228	0.007401	2.138236	up			
RNA59746|RNAz_458_357	0.008047293	0.002292	2.140659	up			
RNA57690|UCRs_5_499	0.024464373	0.010242	2.14071	up			
RNA61585|RNAz_2299_262	0.000336444	2.85E-05	2.141152	up			

RNA34941|NRED_1079_1071	0.002149718	0.000364	2.143069	up			
RNA59791|RNAz_503_354	0.002696404	0.0005	2.143612	up			
RNA50669|UCSC_10017_804	0.02719869	0.011754	2.143745	up			
RNA61325|RNAz_2039_270	0.004394151	0.000984	2.144678	up			
RNA147168|nc-HOXC6-252	0.023570634	0.009761	2.145349	up			
RNA47336|UCSC_5970_1770	0.014690299	0.005216	2.146008	up			
RNA33841|lncRNAdb_48_1047	0.004012721	0.000867	2.146656	up			
RNA146952|nc-HOXD3-38	0.001008245	0.000128	2.146716	up			
RNA59052|asoverlaps_709_1184	0.008902725	0.002636	2.147229	up			
RNA96736|EvoFold_587_201	0.00538625	0.001306	2.1478	up			
RNA34171|NRED_302_2618	0.001896694	0.000307	2.14825	up			
RNA146939|nc-HOXD4-29	0.008844643	0.002614	2.148723	up			
RNA96233|EvoFold_43_321	0.004193164	0.000922	2.149573	up			
RNA62097|RNAz_2813_240	0.029895151	0.013293	2.149918	up			
RNA64636|RNAz_5357_200	0.001295007	0.000181	2.150954	up			
RNA96701|EvoFold_548_205	0.017793844	0.006732	2.152292	up			
RNA46768|UCSC_5295_1925	0.014823678	0.005277	2.155269	up			
RNA61041|RNAz_1755_276	0.003039585	0.000591	2.155526	up			
RNA96636|EvoFold_477_215	0.005297878	0.001278	2.156085	up			
RNA96199|EvoFold_9_417	0.002868182	0.000545	2.156438	up			
RNA47563|UCSC_6272_1700	0.000159405	1.05E-05	2.15895	up			
RNA59527|RNAz_238_398	0.002462693	0.00044	2.160255	up			
RNA48417|UCSC_7297_1459	0.008977723	0.002667	2.16224	up			
RNA64713|RNAz_5434_200	0.011277007	0.003642	2.16349	up			
RNA63944|RNAz_4664_215	0.03336226	0.015305	2.164206	up			
RNA63508|RNAz_4227_225	0.014621864	0.005184	2.164828	up			
RNA61255|RNAz_1969_272	0.007432157	0.002048	2.165487	up			
RNA61906|RNAz_2621_245	0.000406539	3.72E-05	2.166457	up			
RNA46344|UCSC_4778_2043	0.00915331	0.002741	2.167277	up			
RNA53528|H-InvDB_798_504	0.002731135	0.000508	2.168782	up			
RNA147213|int-HOXB3-79	0.00776522	0.002183	2.170145	up			
RNA62016|RNAz_2731_240	0.018105647	0.006887	2.172908	up			
RNA50612|UCSC_9944_823	0.013769366	0.004788	2.173139	up			
RNA96283|EvoFold_95_291	0.001158447	0.000154	2.174337	up			
RNA51175|UCSC_10642_619	0.002734273	0.000509	2.17624	up			
RNA147017|nc-HOXA5-67	0.007786796	0.002191	2.177365	up			

RNA51373|UCSC_10888_549	0.015861772	0.005779	2.178244	up			
RNA64548|RNAz_5269_200	0.03694375	0.01745	2.18082	up			
RNA60924|RNAz_1638_278	0.013362187	0.004596	2.18087	up			
RNA61148|RNAz_1862_274	0.00044706	4.22E-05	2.184484	up			
RNA44278|UCSC_2195_3039	0.018987535	0.007325	2.184513	up			
RNA62385|RNAz_3103_239	0.020669784	0.008205	2.184651	up			
RNA39707|RefSeq_1705_2020	0.007466215	0.002063	2.185616	up			
RNA38816|RefSeq_757_3216	4.02449E-05	1.51E-06	2.185616	up			
RNA51553|UCSC_11119_474	0.0036159	0.000751	2.186406	up			
RNA64164|RNAz_4884_208	0.002309375	0.000402	2.186637	up			
RNA53606|H-InvDB_878_475	0.03935518	0.018878	2.186846	up			
RNA39369|RefSeq_1343_2344	0.000061697	2.86E-06	2.189387	up			
RNA45092|UCSC_3197_2523	0.018751105	0.007202	2.189919	up			
RNA43953|UCSC_1796_3339	8.35181E-05	4.41E-06	2.190569	up			
RNA64537|RNAz_5258_200	0.004429809	0.000994	2.193137	up			
RNA146925|hox-HOXD9-38	0.000285693	2.3E-05	2.19329	up			
RNA46912|UCSC_5462_1884	0.006412236	0.001667	2.193589	up			
RNA60669|RNAz_1383_280	0.011843643	0.003888	2.193912	up			
RNA147259|int-HOXB7-97	0.002372478	0.000417	2.194464	up			
RNA60293|RNAz_1005_309	0.000117335	7.04E-06	2.194673	up			
RNA35680|ENCODE_548_1016	0.003788529	0.000802	2.196534	up			
RNA57888|UCRs_203_252	0.001704788	0.000265	2.198036	up			
RNA60137|RNAz_849_317	0.000336444	2.85E-05	2.198185	up			
RNA51034|UCSC_10456_673	0.001415726	0.000204	2.198598	up			
RNA96230|EvoFold_40_322	5.23625E-05	2.23E-06	2.199086	up			
RNA50598|UCSC_9926_828	0.027561244	0.011949	2.201365	up			
RNA96545|EvoFold_380_227	0.021575196	0.008681	2.202509	up			
RNA50015|UCSC_9207_1003	0.007442001	0.002053	2.203203	up			
RNA64356|RNAz_5077_201	0.001639457	0.000251	2.203423	up			
RNA62654|RNAz_3372_237	0.008129319	0.002326	2.204568	up			
RNA62892|RNAz_3610_235	0.002090056	0.000351	2.204738	up			
RNA54591|H-InvDB_1918_315	3.87244E-05	1.43E-06	2.205302	up			
RNA59827|RNAz_539_349	0.007252061	0.001979	2.207066	up			
RNA61548|RNAz_2262_263	0.001857586	0.000298	2.20747	up			
RNA37258|ENCODE_2129_444	0.00420813	0.000927	2.208418	up			
RNA61576|RNAz_2290_262	0.004674894	0.001072	2.208542	up			

RNA58505|asoverlaps_9_5250	0.000180946	1.25E-05	2.208554	up			
RNA147162|nc-HOXC6-246	0.00627035	0.001618	2.210439	up			
RNA48266|UCSC_7122_1505	0.014815954	0.005272	2.213121	up			
RNA60637|RNAz_1350_281	0.003076119	0.0006	2.21652	up			
RNA64667|RNAz_5388_200	0.003207541	0.000637	2.217834	up			
RNA63597|RNAz_4317_223	0.006408083	0.001665	2.217992	up			
RNA64579|RNAz_5300_200	0.005993468	0.001517	2.218654	up			
RNA61501|RNAz_2215_265	6.92977E-05	3.37E-06	2.219463	up			
RNA64497|RNAz_5218_200	0.000159625	1.05E-05	2.221129	up			
RNA62495|RNAz_3213_238	0.016287502	0.005989	2.221614	up			
RNA49124|UCSC_8140_1244	0.032305047	0.014676	2.222913	up			
RNA41064|RefSeq_3173_633	0.004848946	0.001128	2.223329	up			
RNA60799|RNAz_1513_279	0.00080909	9.46E-05	2.225283	up			
RNA59582|RNAz_293_387	0.01437255	0.005063	2.228321	up			
RNA33951|NRED_81_4222	0.001060931	0.000137	2.228936	up			
RNA53360|H-InvDB_627_567	0.000939046	0.000116	2.229033	up			
RNA60414|RNAz_1126_302	0.008756957	0.002578	2.229406	up			
RNA58342|CombinedLit_305_572	0.01409108	0.004939	2.230413	up			
RNA61814|RNAz_2528_250	0.000230041	1.71E-05	2.231246	up			
RNA59020|asoverlaps_664_1300	0.000210907	1.53E-05	2.231522	up			
RNA48490|UCSC_7385_1433	0.010714682	0.003403	2.2323	up			
RNA58566|asoverlaps_81_3257	0.000411222	3.78E-05	2.233605	up			
RNA59969|RNAz_681_328	0.005652121	0.001394	2.234512	up			
RNA64080|RNAz_4800_211	0.005314006	0.001283	2.237209	up			
RNA61575|RNAz_2289_262	0.001226432	0.000167	2.237938	up			
RNA40236|RefSeq_2275_1512	0.000398929	3.62E-05	2.238371	up			
RNA96495|EvoFold_322_239	0.01424801	0.005006	2.239436	up			
RNA51879|UCSC_11479_351	0.004204694	0.000925	2.240015	up			
RNA46977|UCSC_5539_1866	0.032119505	0.014563	2.241928	up			
RNA51100|UCSC_10536_648	0.000199364	1.42E-05	2.243443	up			
RNA63164|RNAz_3882_231	0.014336265	0.005046	2.243531	up			
RNA60957|RNAz_1671_277	0.031098701	0.013973	2.245176	up			
RNA60785|RNAz_1499_279	0.023179777	0.009551	2.245771	up			
RNA147043|nc-HOXA9-81	0.007110315	0.001925	2.246098	up			
RNA53904|H-InvDB_1186_401	0.008964091	0.002662	2.247223	up			
RNA63012|RNAz_3730_233	0.001731603	0.00027	2.250717	up			

RNA59661|RNAz_372_364	0.007045583	0.001901	2.254013	up			
RNA53052|H-InvDB_311_671	0.00329054	0.00066	2.254662	up			
RNA58112|UCRs_427_206	0.00038069	3.38E-05	2.255067	up			
RNA64598|RNAz_5319_200	0.002898438	0.000553	2.255925	up			
RNA61830|RNAz_2544_249	0.010021737	0.003104	2.256131	up			
RNA44756|UCSC_2786_2715	0.001062371	0.000138	2.257173	up			
RNA40350|RefSeq_2396_1389	0.020092592	0.007904	2.257537	up			
RNA38110|RefSeq_10_11721	0.007123327	0.00193	2.258732	up			
RNA50389|UCSC_9667_896	0.044344716	0.021978	2.262064	up			
RNA63277|RNAz_3995_230	0.009646826	0.002946	2.263213	up			
RNA64152|RNAz_4872_208	9.36687E-05	5.2E-06	2.264774	up			
RNA54968|H-InvDB_2329_268	0.003705498	0.000776	2.264971	up			
RNA60769|RNAz_1483_279	0.004800273	0.001112	2.267883	up			
RNA60589|RNAz_1302_287	0.003255947	0.00065	2.27087	up			
RNA41069|RefSeq_3181_627	0.000297352	2.42E-05	2.271057	up			
RNA57936|UCRs_251_239	0.008351802	0.002414	2.275602	up			
RNA62148|RNAz_2866_240	0.021380264	0.008581	2.275899	up			
RNA64331|RNAz_5052_202	0.007912541	0.002243	2.278107	up			
RNA39216|RefSeq_1181_2526	0.003914658	0.000839	2.279624	up			
RNA63307|RNAz_4025_229	0.001216236	0.000165	2.279827	up			
RNA61748|RNAz_2462_253	0.030492652	0.013619	2.280171	up			
RNA39566|RefSeq_1556_2147	0.03638549	0.01711	2.280294	up			
RNA60602|RNAz_1315_286	0.001892109	0.000306	2.281492	up			
RNA40880|RefSeq_2973_835	1.62028E-05	4.12E-07	2.28151	up			
RNA43300|UCSC_1008_4071	0.006017045	0.001525	2.282991	up			
RNA58099|UCRs_414_207	0.000448595	4.24E-05	2.283524	up			
RNA58881|asoverlaps_482_1750	0.000277756	2.21E-05	2.283819	up			
RNA64909|RNAz_5630_200	0.001666976	0.000256	2.286459	up			
RNA147220|int-HOXB3-86	0.039422836	0.018918	2.288462	up			
RNA38557|RefSeq_478_3892	0.032545876	0.014822	2.289516	up			
RNA62037|RNAz_2752_240	0.006414304	0.001668	2.289932	up			
RNA62836|RNAz_3554_235	0.00021997	1.61E-05	2.291188	up			
RNA50253|UCSC_9500_938	0.003389475	0.000687	2.294567	up			
RNA58069|UCRs_384_211	4.21808E-05	1.62E-06	2.295404	up			
RNA64145|RNAz_4865_209	0.001163173	0.000155	2.295687	up			
RNA53562|H-InvDB_832_495	0.000421277	3.9E-05	2.297985	up			

RNA57704|UCRs_19_397	0.00050519	4.98E-05	2.298233	up			
RNA63077|RNAz_3795_232	0.000231887	1.73E-05	2.299474	up			
RNA96206|EvoFold_16_365	0.00487086	0.001136	2.302179	up			
RNA147075|hox-HOXC8-64	0.024306275	0.010148	2.302709	up			
RNA60880|RNAz_1594_278	0.03571377	0.01671	2.303813	up			
RNA46680|UCSC_5197_1948	0.001396179	0.0002	2.306904	up			
RNA96546|EvoFold_381_227	0.009294406	0.0028	2.307127	up			
RNA63882|RNAz_4602_217	0.002581495	0.00047	2.308978	up			
RNA53159|H-InvDB_421_643	0.00827181	0.002382	2.309362	up			
RNA62234|RNAz_2952_240	0.012893662	0.004368	2.312758	up			
RNA96235|EvoFold_45_320	0.001445588	0.00021	2.318281	up			
RNA60163|RNAz_875_316	0.006768123	0.001796	2.318665	up			
RNA59562|RNAz_273_391	0.001403728	0.000201	2.319448	up			
RNA34942|NRED_1081_1068	0.000685404	7.57E-05	2.319573	up			
RNA64864|RNAz_5585_200	0.007578253	0.002106	2.319952	up			
RNA62865|RNAz_3583_235	0.003426895	0.000698	2.323252	up			
RNA64699|RNAz_5420_200	0.000173172	1.17E-05	2.331071	up			
RNA38425|RefSeq_341_4414	5.31477E-05	2.29E-06	2.332884	up			
RNA61483|RNAz_2197_265	0.008431695	0.002448	2.333554	up			
RNA54038|H-InvDB_1322_383	0.001853917	0.000298	2.334205	up			
RNA53928|H-InvDB_1210_398	0.038423784	0.018322	2.335951	up			
RNA62645|RNAz_3363_237	0.015262953	0.005483	2.336125	up			
RNA63798|RNAz_4518_219	0.033027425	0.015113	2.341214	up			
RNA58246|CombinedLit_161_1078	0.000541132	5.46E-05	2.343528	up			
RNA61091|RNAz_1805_275	0.001792543	0.000283	2.34563	up			
RNA53917|H-InvDB_1199_399	0.002829194	0.000534	2.346744	up			
RNA96198|EvoFold_8_420	0.024473041	0.010246	2.347492	up			
RNA38177|RefSeq_85_6836	0.002265301	0.000391	2.347698	up			
RNA55680|H-InvDB_3066_204	0.026911175	0.011583	2.350615	up			
RNA64043|RNAz_4763_212	0.002162952	0.000367	2.353603	up			
RNA62211|RNAz_2929_240	0.000598105	6.27E-05	2.355567	up			
RNA63946|RNAz_4666_215	0.003785248	0.000801	2.355779	up			
RNA49661|UCSC_8785_1095	0.000437068	4.1E-05	2.36144	up			
RNA63073|RNAz_3791_233	0.013961057	0.004877	2.366882	up			
RNA43692|UCSC_1485_3565	0.006959451	0.001869	2.367308	up			
RNA63510|RNAz_4229_225	0.003441598	0.000702	2.368115	up			

RNA59623|RNAz_334_377	0.00081567	9.56E-05	2.373467	up			
RNA48973|UCSC_7972_1285	0.021771904	0.008787	2.375152	up			
RNA47066|UCSC_5650_1841	0.002386316	0.000421	2.37796	up			
RNA52085|UCSC_11709_237	0.025506426	0.010811	2.378002	up			
RNA47491|UCSC_6165_1725	0.002791671	0.000525	2.379197	up			
RNA60753|RNAz_1467_279	0.005019519	0.001184	2.384621	up			
RNA53818|H-InvDB_1098_417	0.027053207	0.011663	2.385299	up			
RNA147148|nc-HOXC6-351	0.001582554	0.000239	2.386096	up			
RNA50597|UCSC_9925_828	0.009343657	0.002821	2.387093	up			
RNA49331|UCSC_8395_1183	0.026715446	0.011475	2.388353	up			
RNA61409|RNAz_2123_268	0.005709771	0.001414	2.39207	up			
RNA40375|RefSeq_2426_1364	0.005486125	0.001339	2.393381	up			
RNA60836|RNAz_1550_279	0.008565479	0.002502	2.395826	up			
RNA58887|asoverlaps_488_1736	0.000746102	8.49E-05	2.398033	up			
RNA58203|CombinedLit_71_1878	0.008770027	0.002583	2.398217	up			
RNA62939|RNAz_3657_234	0.016912103	0.00629	2.398347	up			
RNA46910|UCSC_5460_1885	0.00895893	0.00266	2.39919	up			
RNA61059|RNAz_1773_276	0.0005288	5.3E-05	2.400754	up			
RNA51518|UCSC_11080_488	0.006854355	0.001828	2.407763	up			
RNA61032|RNAz_1746_276	0.008558478	0.002499	2.409215	up			
RNA62555|RNAz_3273_238	0.000227481	1.69E-05	2.410473	up			
RNA146938|nc-HOXD4-28	0.006258488	0.001613	2.411334	up			
RNA47778|UCSC_6524_1642	0.000269883	2.13E-05	2.412246	up			
RNA60889|RNAz_1603_278	0.001123339	0.000148	2.413683	up			
RNA49690|UCSC_8820_1086	0.004275407	0.000949	2.414919	up			
RNA62401|RNAz_3119_239	0.006620039	0.001743	2.415653	up			
RNA49977|UCSC_9160_1012	0.015823051	0.005761	2.41742	up			
RNA60126|RNAz_838_317	0.002606725	0.000476	2.420577	up			
RNA53067|H-InvDB_326_664	0.015495176	0.005602	2.421624	up			
RNA40818|RefSeq_2904_905	0.00591284	0.001488	2.421806	up			
RNA34398|NRED_530_2098	0.000570232	5.88E-05	2.422061	up			
RNA47858|UCSC_6620_1623	0.001584683	0.000239	2.422334	up			
RNA34911|NRED_1045_1161	0.018915387	0.007287	2.426221	up			
RNA61261|RNAz_1975_271	0.008324282	0.002403	2.427873	up			
RNA51212|UCSC_10684_608	0.000541968	5.47E-05	2.429437	up			
RNA43404|UCSC_1135_3885	0.000543696	5.5E-05	2.429765	up			

RNA40653|RefSeq_2728_1070	0.000482054	4.68E-05	2.431228	up			
RNA44066|UCSC_1935_3233	0.012619306	0.004241	2.43269	up			
RNA49918|UCSC_9095_1029	0.041311778	0.020077	2.433027	up			
RNA54159|H-InvDB_1459_362	0.012973756	0.004407	2.434714	up			
RNA58091|UCRs_406_208	2.02399E-05	5.69E-07	2.441781	up			
RNA39712|RefSeq_1712_2017	0.013873666	0.004835	2.442946	up			
RNA63693|RNAz_4413_221	0.000743116	8.45E-05	2.448608	up			
RNA147196|nc-HOXB2-164	0.014444032	0.005097	2.449868	up			
RNA47696|UCSC_6428_1665	7.0548E-05	3.45E-06	2.452308	up			
RNA63469|RNAz_4188_226	0.003170205	0.000626	2.454679	up			
RNA96192|EvoFold_1_451	0.004173335	0.000915	2.455186	up			
RNA34375|NRED_507_2132	0.008688365	0.00255	2.45632	up			
RNA50786|UCSC_10169_754	0.02621407	0.011199	2.458545	up			
RNA64367|RNAz_5088_200	0.000700515	7.8E-05	2.460507	up			
RNA53389|H-InvDB_658_551	0.002379314	0.000419	2.46094	up			
RNA59067|asoverlaps_724_1132	4.92462E-06	7.06E-08	2.461234	up			
RNA147058|nc-HOXA11-87	0.016189493	0.005941	2.461962	up			
RNA38841|RefSeq_782_3160	0.024551718	0.010287	2.474653	up			
RNA64306|RNAz_5027_203	0.00028512	2.29E-05	2.479639	up			
RNA94923|RNS_5_200	0.002284809	0.000396	2.48469	up			
RNA47573|UCSC_6284_1698	0.003920414	0.000841	2.485475	up			
RNA64600|RNAz_5321_200	0.003282212	0.000658	2.489712	up			
RNA50578|UCSC_9897_836	0.005946957	0.001501	2.490439	up			
RNA62643|RNAz_3361_237	0.001026656	0.000132	2.490726	up			
RNA48354|UCSC_7224_1476	0.003358034	0.000679	2.490843	up			
RNA62105|RNAz_2821_240	0.005096601	0.001211	2.492011	up			
RNA60162|RNAz_874_316	0.000270134	2.13E-05	2.494329	up			
RNA39759|RefSeq_1763_1980	0.01489393	0.005309	2.494742	up			
RNA35122|NRED_1263_344	0.008423942	0.002444	2.495535	up			
RNA146918|hox-HOXD10-35	0.000564396	5.79E-05	2.499095	up			
RNA59493|RNAz_204_410	0.000519433	5.18E-05	2.501466	up			
RNA47169|UCSC_5771_1814	0.017115656	0.006391	2.502864	up			
RNA33798|lncRNAdb_5_9848	0.010713178	0.003402	2.505452	up			
RNA64425|RNAz_5146_200	0.032588623	0.01485	2.509752	up			
RNA64627|RNAz_5348_200	0.00165231	0.000253	2.511813	up			
RNA49793|UCSC_8945_1062	0.015393579	0.005549	2.518726	up			

RNA38198|RefSeq_106_6583	0.001289627	0.000179	2.519328	up				
RNA63245|RNAz_3963_230	0.001727652	0.00027	2.519709	up				
RNA147177|hox-HOXC5-260	0.002766129	0.000518	2.519763	up				
RNA50286|UCSC_9540_932	0.001506771	0.000223	2.527376	up				
RNA45386|UCSC_3579_2380	0.002081913	0.000349	2.529436	up				
RNA34708|NRED_840_1588	0.017882565	0.006778	2.531388	up				
RNA61728|RNAz_2442_254	0.014780663	0.005257	2.531835	up				
RNA61513|RNAz_2227_264	0.004012721	0.000867	2.534266	up				
RNA64846|RNAz_5567_200	0.002128162	0.00036	2.536382	up				
RNA50394|UCSC_9675_894	0.001763632	0.000277	2.536829	up				
RNA51295|UCSC_10789_578	3.31718E-05	1.15E-06	2.53726	up				
RNA62536|RNAz_3254_238	0.002404142	0.000425	2.547485	up				
RNA60904|RNAz_1618_278	0.000163002	1.08E-05	2.549303	up				
RNA59958|RNAz_670_331	0.000793326	9.23E-05	2.555728	up				
RNA45645|UCSC_3910_2284	0.002488007	0.000446	2.557262	up				
RNA48538|UCSC_7445_1421	0.01963982	0.007672	2.557278	up				
RNA39848|RefSeq_1859_1873	0.023853257	0.009908	2.558594	up				
RNA53227|H-InvDB_490_631	0.001161986	0.000155	2.560516	up				
RNA58426|CombinedLit_433_300	0.000260939	2.03E-05	2.5616	up				
RNA61936|RNAz_2651_243	0.004502057	0.001018	2.564149	up				
RNA53022|H-InvDB_281_686	0.000764741	8.79E-05	2.564318	up				
RNA64496|RNAz_5217_200	0.012857852	0.004352	2.565028	up				
RNA58960|asoverlaps_592_1486	0.001535612	0.000229	2.566015	up				
RNA61980|RNAz_2695_240	0.03357955	0.01543	2.56769	up				
RNA59442|RNAz_153_431	0.006942034	0.001863	2.568495	up				
RNA51608|UCSC_11183_453	0.005182859	0.001241	2.572014	up				
RNA47608|UCSC_6324_1688	0.006697049	0.001769	2.580428	up				
RNA57970|UCRs_285_232	0.023549108	0.009749	2.581327	up				
RNA60349|RNAz_1061_306	7.69756E-05	3.93E-06	2.587492	up				
RNA58192|CombinedLit_49_2299	0.003189353	0.000631	2.587731	up				
RNA58133|UCRs_448_203	0.000263733	2.06E-05	2.589099	up				
RNA146948|hox-HOXD4-43	0.002263608	0.000391	2.590216	up				
RNA62004|RNAz_2719_240	0.00155229	0.000233	2.590631	up				
RNA57910|UCRs_225_247	0.002134685	0.000361	2.60145	up				
RNA64278|RNAz_4999_204	0.007927522	0.002249	2.603237	up				
RNA64401|RNAz_5122_200	0.004853213	0.00113	2.61316	up				

RNA147225|int-HOXB3-91	0.027354034	0.011836	2.617522	up				
RNA43750|UCSC_1552_3513	0.013354976	0.004592	2.62039	up				
RNA58296|CombinedLit_237_764	0.001804593	0.000287	2.622801	up				
RNA58024|UCRs_339_219	0.003463734	0.000708	2.622969	up				
RNA60289|RNAz_1001_310	0.001317621	0.000185	2.630084	up				
RNA61633|RNAz_2347_259	0.013950913	0.004871	2.631193	up				
RNA35085|NRED_1226_577	0.003256569	0.000651	2.631257	up				
RNA64380|RNAz_5101_200	0.022883946	0.009391	2.632489	up				
RNA53941|H-InvDB_1223_396	0.029894823	0.013292	2.638201	up				
RNA61426|RNAz_2140_267	0.005934472	0.001495	2.638204	up				
RNA146976|hox-HOXA1-1	0.027833465	0.012092	2.639662	up				
RNA64362|RNAz_5083_201	0.004101801	0.000894	2.646295	up				
RNA51145|UCSC_10601_631	0.015217898	0.005461	2.649611	up				
RNA34832|NRED_966_1346	0.011695514	0.003824	2.651518	up				
RNA48043|UCSC_6842_1573	0.016651437	0.006167	2.656427	up				
RNA47378|UCSC_6021_1757	0.005952469	0.001503	2.661443	up				
RNA63739|RNAz_4459_220	0.0068283	0.001818	2.663475	up				
RNA54595|H-InvDB_1922_315	0.031828724	0.014391	2.668166	up				
RNA48755|UCSC_7720_1350	0.010299483	0.003221	2.670169	up				
RNA60576|RNAz_1289_288	0.00899306	0.002673	2.671425	up				
RNA59964|RNAz_676_329	0.012649481	0.004254	2.674123	up				
RNA62196|RNAz_2914_240	0.00054175	5.47E-05	2.679909	up				
RNA58929|asoverlaps_546_1603	6.19164E-05	2.88E-06	2.681089	up				
RNA53759|H-InvDB_1037_429	0.001334533	0.000188	2.6822	up				
RNA34867|NRED_1001_1263	0.025568595	0.010845	2.682472	up				
RNA63513|RNAz_4232_225	0.00042905	4E-05	2.684119	up				
RNA54759|H-InvDB_2094_296	0.012731786	0.004293	2.691165	up				
RNA64920|RNAz_5641_200	0.000423329	3.93E-05	2.691453	up				
RNA57815|UCRs_130_277	7.65428E-06	1.37E-07	2.693714	up				
RNA62072|RNAz_2787_240	0.000138327	8.76E-06	2.705933	up				
RNA61175|RNAz_1889_273	0.01788895	0.006781	2.709553	up				
RNA64784|RNAz_5505_200	0.001749143	0.000274	2.709855	up				
RNA46428|UCSC_4888_2015	0.001185456	0.000159	2.715509	up				
RNA59841|RNAz_553_348	0.001902801	0.000308	2.720483	up				
RNA62413|RNAz_3131_239	0.006347112	0.001645	2.724028	up				
RNA54592|H-InvDB_1919_315	2.4244E-05	7.38E-07	2.729021	up				

RNA38550|RefSeq_471_3909	0.000513869	5.11E-05	2.732493	up				
RNA45866|UCSC_4193_2197	0.015808621	0.005751	2.733175	up				
RNA53472|H-InvDB_741_527	0.032091353	0.014543	2.735884	up				
RNA147164|nc-HOXC6-248	0.003859875	0.000823	2.736622	up				
RNA59754|RNAz_466_356	8.60573E-05	4.62E-06	2.739893	up				
RNA60443|RNAz_1155_300	0.007716235	0.002161	2.743792	up				
RNA64155|RNAz_4875_208	0.020676456	0.008209	2.743979	up				
RNA61296|RNAz_2010_271	0.002606887	0.000476	2.746486	up				
RNA45956|UCSC_4293_2165	0.000639515	6.87E-05	2.747679	up				
RNA38357|RefSeq_272_4798	5.22609E-05	2.23E-06	2.752632	up				
RNA55139|H-InvDB_2510_248	0.046727877	0.023452	2.758483	up				
RNA146928|nc-HOXD8-20	0.001163173	0.000155	2.76054	up				
RNA53019|H-InvDB_278_686	0.0474468	0.023901	2.76092	up				
RNA43117|UCSC_798_4435	0.006073652	0.001546	2.761643	up				
RNA53178|H-InvDB_440_641	0.002529148	0.000456	2.762703	up				
RNA61860|RNAz_2574_248	0.001204339	0.000163	2.764837	up				
RNA54166|H-InvDB_1467_360	0.02627246	0.011229	2.766561	up				
RNA54477|H-InvDB_1797_324	0.021753808	0.008776	2.767081	up				
RNA59371|RNAz_82_469	0.021163043	0.008461	2.767547	up				
RNA40750|RefSeq_2830_978	0.001540219	0.00023	2.768676	up				
RNA38616|RefSeq_543_3726	0.003726194	0.000782	2.779794	up				
RNA63880|RNAz_4600_217	8.79639E-05	4.75E-06	2.78482	up				
RNA63051|RNAz_3769_233	0.000184366	1.28E-05	2.788964	up				
RNA63836|RNAz_4556_218	0.000365266	3.2E-05	2.789234	up				
RNA60727|RNAz_1441_280	0.008155691	0.002335	2.7896	up				
RNA64196|RNAz_4917_207	0.008240466	0.002369	2.80111	up				
RNA63860|RNAz_4580_217	0.000528863	5.3E-05	2.807441	up				
RNA60179|RNAz_891_315	0.000974106	0.000122	2.808024	up				
RNA54167|H-InvDB_1468_360	0.026674984	0.011451	2.809822	up				
RNA146961|int-HOXD3-3	0.005411156	0.001313	2.810845	up				
RNA59033|asoverlaps_682_1255	0.001402273	0.000201	2.82158	up				
RNA60724|RNAz_1438_280	0.007760601	0.002181	2.826849	up				
RNA59882|RNAz_594_342	0.012153463	0.004032	2.832566	up				
RNA34674|NRED_806_1652	0.001701233	0.000264	2.836211	up				
RNA96551|EvoFold_388_227	4.27431E-05	1.65E-06	2.837351	up				
RNA51619|UCSC_11196_448	0.000172012	1.16E-05	2.847076	up				

RNA55135|H-InvDB_2505_248	0.004242581	0.000937	2.847454	up			
RNA40418|RefSeq_2473_1324	2.59785E-05	8.15E-07	2.851774	up			
RNA57794|UCRs_109_294	0.023630712	0.009793	2.854018	up			
RNA146964|int-HOXD3-5	0.003687451	0.000771	2.855982	up			
RNA43003|UCSC_659_4708	2.29353E-05	6.84E-07	2.856141	up			
RNA33931|NRED_61_4665	0.026801907	0.011524	2.857784	up			
RNA53090|H-InvDB_349_656	0.003422758	0.000696	2.870843	up			
RNA54154|H-InvDB_1454_363	0.022190295	0.009008	2.870887	up			
RNA46474|UCSC_4950_2004	0.001441851	0.000209	2.871032	up			
RNA59715|RNAz_427_359	0.005277444	0.001271	2.875191	up			
RNA96253|EvoFold_64_304	0.017724838	0.006697	2.883628	up			
RNA38910|RefSeq_854_3016	0.007988952	0.00227	2.884738	up			
RNA41212|RefSeq_3341_451	0.004181708	0.000918	2.889214	up			
RNA39960|RefSeq_1983_1758	0.031969093	0.014472	2.909866	up			
RNA54112|H-InvDB_1406_370	0.038475826	0.018357	2.922002	up			
RNA33924|NRED_54_4930	0.001495065	0.000221	2.924686	up			
RNA60815|RNAz_1529_279	0.010632406	0.003367	2.927156	up			
RNA54352|H-InvDB_1662_339	0.04907225	0.024942	2.927865	up			
RNA58209|CombinedLit_82_1626	0.005320198	0.001285	2.933395	up			
RNA61078|RNAz_1792_275	0.00096246	0.00012	2.935242	up			
RNA39717|RefSeq_1717_2016	0.000219388	1.61E-05	2.936879	up			
RNA62443|RNAz_3161_238	0.03177581	0.01436	2.937173	up			
RNA54176|H-InvDB_1477_359	0.001646756	0.000252	2.943887	up			
RNA54708|H-InvDB_2042_301	0.033205662	0.015216	2.951732	up			
RNA46007|UCSC_4359_2143	0.005327031	0.001287	2.959653	up			
RNA64451|RNAz_5172_200	0.000373742	3.3E-05	2.960152	up			
RNA45872|UCSC_4199_2195	0.003046568	0.000593	2.962275	up			
RNA50870|UCSC_10267_726	0.000406914	3.73E-05	2.969516	up			
RNA47784|UCSC_6533_1640	0.009422096	0.002854	2.972566	up			
RNA34244|NRED_375_2425	0.001792047	0.000283	2.974112	up			
RNA64538|RNAz_5259_200	0.008293073	0.00239	2.980557	up			
RNA54151|H-InvDB_1451_363	0.004431909	0.000995	2.982841	up			
RNA63014|RNAz_3732_233	0.002086041	0.00035	2.983029	up			
RNA34609|NRED_741_1740	0.000786821	9.13E-05	2.984758	up			
RNA35088|NRED_1229_575	0.000256524	1.98E-05	2.986665	up			
RNA53202|H-InvDB_465_636	0.03124382	0.014054	2.987783	up			

RNA54765|H-InvDB_2100_294	0.03457291	0.016031	2.988635	up			
RNA53182|H-InvDB_444_641	0.038833935	0.018574	2.99118	up			
RNA64088|RNAz_4808_211	0.005176942	0.001239	2.997098	up			
RNA96193|EvoFold_2_450	0.014232147	0.004999	2.999051	up			
RNA53824|H-InvDB_1104_416	0.001225785	0.000167	3.002673	up			
RNA54310|H-InvDB_1617_342	0.001593917	0.000241	3.008611	up			
RNA147166|nc-HOXC6-250	0.00081072	9.48E-05	3.018636	up			
RNA59553|RNAz_264_393	0.009279176	0.002793	3.02327	up			
RNA44994|UCSC_3080_2568	0.006853008	0.001827	3.026954	up			
RNA53955|H-InvDB_1237_393	0.005065633	0.0012	3.027678	up			
RNA54486|H-InvDB_1807_324	0.034798	0.016167	3.029466	up			
RNA54940|H-InvDB_2296_272	0.022567978	0.009213	3.029579	up			
RNA58876|asoverlaps_476_1755	0.001301152	0.000182	3.029966	up			
RNA54032|H-InvDB_1314_384	0.017173732	0.00642	3.03207	up			
RNA54519|H-InvDB_1842_321	0.033042897	0.015123	3.036009	up			
RNA54482|H-InvDB_1803_324	0.043924697	0.021702	3.041602	up			
RNA54537|H-InvDB_1860_321	0.02877	0.012647	3.042833	up			
RNA50966|UCSC_10376_699	0.001316477	0.000185	3.046006	up			
RNA53301|H-InvDB_567_596	0.008166934	0.00234	3.051195	up			
RNA57789|UCRs_104_296	0.002327842	0.000406	3.054962	up			
RNA54059|H-InvDB_1351_380	0.018856207	0.007254	3.058999	up			
RNA57961|UCRs_276_234	2.08726E-06	1.82E-08	3.060081	up			
RNA53122|H-InvDB_382_649	0.00379033	0.000803	3.070099	up			
RNA54493|H-InvDB_1814_323	0.005444613	0.001325	3.078708	up			
RNA63279|RNAz_3997_230	0.000226037	1.67E-05	3.078842	up			
RNA54524|H-InvDB_1847_321	0.033596803	0.015441	3.092039	up			
RNA59196|asoverlaps_903_630	0.010547617	0.003328	3.092597	up			
RNA58692|asoverlaps_240_2336	0.000503908	4.97E-05	3.093628	up			
RNA96347|EvoFold_166_270	0.002096552	0.000353	3.097214	up			
RNA54538|H-InvDB_1861_321	0.026709465	0.011471	3.098661	up			
RNA58829|asoverlaps_422_1865	0.011528788	0.003749	3.108263	up			
RNA54534|H-InvDB_1857_321	0.02986192	0.013275	3.108287	up			
RNA54485|H-InvDB_1806_324	0.031492356	0.014201	3.108481	up			
RNA53231|H-InvDB_494_630	2.23575E-05	6.59E-07	3.110326	up			
RNA53180|H-InvDB_442_641	0.03987723	0.019186	3.120134	up			
RNA39275|RefSeq_1243_2458	0.000466345	4.47E-05	3.120477	up			

RNA34648|NRED_780_1681	0.000530431	5.32E-05	3.130118	up			
RNA96624|EvoFold_465_216	0.004748246	0.001095	3.132022	up			
RNA53949|H-InvDB_1231_395	0.042141795	0.02059	3.139304	up			
RNA62933|RNAz_3651_234	0.004977034	0.001171	3.143889	up			
RNA53996|H-InvDB_1278_388	0.019637661	0.00767	3.145665	up			
RNA54585|H-InvDB_1912_317	0.023409987	0.009672	3.147756	up			
RNA63505|RNAz_4224_225	1.15239E-06	6.45E-09	3.150587	up			
RNA54413|H-InvDB_1726_332	0.040526245	0.019591	3.153747	up			
RNA44512|UCSC_2487_2863	0.004409461	0.000988	3.158831	up			
RNA61356|RNAz_2070_269	0.000588698	6.14E-05	3.162186	up			
RNA62260|RNAz_2978_239	0.001043791	0.000134	3.163899	up			
RNA38685|RefSeq_618_3554	0.000130075	8.08E-06	3.169841	up			
RNA46624|UCSC_5130_1961	0.001800643	0.000286	3.18185	up			
RNA54168|H-InvDB_1469_360	0.016346926	0.006018	3.182433	up			
RNA54420|H-InvDB_1733_332	0.035696927	0.016701	3.185085	up			
RNA54636|H-InvDB_1967_311	0.02673035	0.011484	3.186058	up			
RNA54542|H-InvDB_1865_321	0.029312264	0.012957	3.188499	up			
RNA54336|H-InvDB_1644_340	0.034787774	0.016161	3.190957	up			
RNA36451|ENCODE_1321_601	0.010927038	0.003491	3.201893	up			
RNA54126|H-InvDB_1422_368	0.04947089	0.025206	3.211304	up			
RNA64758|RNAz_5479_200	0.000245923	1.87E-05	3.213412	up			
RNA54334|H-InvDB_1642_340	0.037994284	0.018059	3.219392	up			
RNA54183|H-InvDB_1484_358	0.042414345	0.020761	3.224372	up			
RNA54525|H-InvDB_1848_321	0.030375684	0.013552	3.228954	up			
RNA54286|H-InvDB_1591_345	0.006156769	0.001576	3.253959	up			
RNA54514|H-InvDB_1837_321	0.02485715	0.010455	3.253998	up			
RNA54522|H-InvDB_1845_321	0.025936147	0.011045	3.264681	up			
RNA59992|RNAz_704_323	0.010705953	0.003399	3.265004	up			
RNA53845|H-InvDB_1126_411	0.016715767	0.006198	3.267521	up			
RNA54353|H-InvDB_1663_339	0.03779271	0.017945	3.274626	up			
RNA54284|H-InvDB_1589_345	0.024221687	0.010104	3.277395	up			
RNA34385|NRED_517_2120	0.000268139	2.1E-05	3.278801	up			
RNA54541|H-InvDB_1864_321	0.030139431	0.013425	3.279733	up			
RNA53921|H-InvDB_1203_399	0.005662674	0.001397	3.28212	up			
RNA48643|UCSC_7568_1390	0.005012963	0.001183	3.284411	up			
RNA58651|asoverlaps_185_2551	2.4339E-05	7.45E-07	3.292388	up			

RNA64925|RNAz_5646_200	0.001181088	0.000158	3.294363	up			
RNA60285|RNAz_997_310	0.000994654	0.000126	3.299043	up			
RNA54040|H-InvDB_1324_383	0.022476496	0.009161	3.301013	up			
RNA54122|H-InvDB_1418_369	0.024436893	0.010225	3.303929	up			
RNA45497|UCSC_3717_2342	0.009362601	0.002829	3.304467	up			
RNA64694|RNAz_5415_200	0.003248648	0.000648	3.30729	up			
RNA40986|RefSeq_3087_710	0.005842235	0.001463	3.308384	up			
RNA53974|H-InvDB_1256_390	0.025888972	0.011019	3.310392	up			
RNA33995|NRED_126_3551	4.32983E-05	1.69E-06	3.311952	up			
RNA54480|H-InvDB_1800_324	0.00410739	0.000896	3.312826	up			
RNA54470|H-InvDB_1790_325	0.028955854	0.012755	3.313796	up			
RNA40984|RefSeq_3085_710	0.005683506	0.001406	3.315428	up			
RNA54601|H-InvDB_1928_315	0.032217823	0.01462	3.315736	up			
RNA54523|H-InvDB_1846_321	0.02640774	0.011298	3.32753	up			
RNA54238|H-InvDB_1541_351	0.017605906	0.006639	3.335596	up			
RNA64499|RNAz_5220_200	0.001409898	0.000203	3.339541	up			
RNA53145|H-InvDB_407_646	0.007507119	0.00208	3.343872	up			
RNA59294|RNAz_5_883	0.009259377	0.002783	3.35214	up			
RNA54516|H-InvDB_1839_321	0.025236413	0.010666	3.353366	up			
RNA46729|UCSC_5250_1937	0.000574317	5.94E-05	3.365929	up			
RNA34114|NRED_245_2837	0.000340756	2.91E-05	3.378548	up			
RNA40109|RefSeq_2137_1634	9.99866E-05	5.7E-06	3.387845	up			
RNA58599|asoverlaps_126_2815	0.006237921	0.001605	3.394092	up			
RNA53278|H-InvDB_542_614	0.005379079	0.001303	3.395069	up			
RNA54472|H-InvDB_1792_325	0.022724502	0.009295	3.400317	up			
RNA53987|H-InvDB_1269_389	0.001857442	0.000298	3.402133	up			
RNA54521|H-InvDB_1844_321	0.027164316	0.011732	3.40464	up			
RNA64281|RNAz_5002_204	0.013474449	0.004647	3.410228	up			
RNA44341|UCSC_2282_2984	0.000725953	8.2E-05	3.419125	up			
RNA34166|NRED_297_2627	0.000256524	1.98E-05	3.419247	up			
RNA58259|CombinedLit_181_987	0.003291883	0.000661	3.424686	up			
RNA54100|H-InvDB_1394_372	0.019461192	0.007577	3.427201	up			
RNA53865|H-InvDB_1146_408	0.015197679	0.005451	3.428368	up			
RNA54495|H-InvDB_1816_323	0.037500136	0.017772	3.442919	up			
RNA53980|H-InvDB_1262_390	0.025296107	0.010701	3.446396	up			
RNA54603|H-InvDB_1930_315	0.0288942	0.012719	3.446749	up			

RNA54471|H-InvDB_1791_325	0.024345586	0.010175	3.448334	up				
RNA55175|H-InvDB_2546_243	0.028752662	0.012636	3.458511	up				
RNA96384|EvoFold_206_261	0.000139081	8.83E-06	3.464995	up				
RNA49317|UCSC_8378_1186	0.000392864	3.54E-05	3.479172	up				
RNA53504|H-InvDB_774_515	0.007172356	0.001949	3.501497	up				
RNA54517|H-InvDB_1840_321	0.022156233	0.00899	3.503054	up				
RNA63030|RNAz_3748_233	0.003083974	0.000602	3.508298	up				
RNA61207|RNAz_1921_273	0.004848045	0.001127	3.508678	up				
RNA54109|H-InvDB_1403_370	0.04168834	0.020306	3.508723	up				
RNA64590|RNAz_5311_200	0.008047847	0.002292	3.511869	up				
RNA45425|UCSC_3626_2370	0.00064838	6.99E-05	3.519875	up				
RNA59833|RNAz_545_348	1.11015E-05	2.39E-07	3.527819	up				
RNA49586|UCSC_8696_1116	0.03157519	0.014248	3.543153	up				
RNA54235|H-InvDB_1538_351	0.012303369	0.004098	3.545087	up				
RNA54488|H-InvDB_1809_324	0.03485941	0.016204	3.551603	up				
RNA49498|UCSC_8588_1138	8.16395E-06	1.51E-07	3.564473	up				
RNA49917|UCSC_9094_1029	0.000239474	1.81E-05	3.565432	up				
RNA54087|H-InvDB_1380_375	0.00646358	0.001685	3.567924	up				
RNA44915|UCSC_2980_2612	9.80254E-06	1.97E-07	3.568756	up				
RNA60665|RNAz_1379_280	0.000136544	8.61E-06	3.571426	up				
RNA53204|H-InvDB_467_636	0.024996903	0.010539	3.578336	up				
RNA54616|H-InvDB_1946_312	0.011823273	0.003879	3.580846	up				
RNA59374|RNAz_85_466	0.000730968	8.28E-05	3.580948	up				
RNA34065|NRED_196_3056	0.000442437	4.17E-05	3.582332	up				
RNA59320|RNAz_31_543	0.043940086	0.021718	3.585952	up				
RNA63592|RNAz_4312_223	0.006634323	0.001748	3.592081	up				
RNA53183|H-InvDB_445_641	0.001297068	0.000181	3.599274	up				
RNA61416|RNAz_2130_267	9.06323E-05	4.96E-06	3.609803	up				
RNA39048|RefSeq_1001_2794	3.65121E-05	1.31E-06	3.622795	up				
RNA53201|H-InvDB_464_636	0.023742411	0.009852	3.626019	up				
RNA61015|RNAz_1729_276	0.004729331	0.001089	3.631145	up				
RNA54647|H-InvDB_1978_309	0.025283877	0.010694	3.636657	up				
RNA54068|H-InvDB_1361_378	0.000206219	1.48E-05	3.637224	up				
RNA44555|UCSC_2535_2841	0.001300375	0.000181	3.647664	up				
RNA96511|EvoFold_340_234	0.003275237	0.000656	3.662049	up				
RNA54378|H-InvDB_1688_336	0.020043999	0.007876	3.671881	up				

RNA54029|H-InvDB_1311_384	0.024120646	0.010051	3.675331	up			
RNA64372|RNAz_5093_200	0.00312377	0.000613	3.682502	up			
RNA58597|asoverlaps_124_2830	0.006356275	0.001648	3.688995	up			
RNA54648|H-InvDB_1979_309	0.021709435	0.008751	3.714255	up			
RNA34860|NRED_994_1275	0.00037225	3.27E-05	3.72801	up			
RNA54707|H-InvDB_2041_301	0.033893824	0.015618	3.72922	up			
RNA54335|H-InvDB_1643_340	0.026691157	0.011461	3.753668	up			
RNA55147|H-InvDB_2518_246	0.024464373	0.010242	3.77264	up			
RNA44217|UCSC_2122_3100	0.000818251	9.6E-05	3.77583	up			
RNA54441|H-InvDB_1756_329	0.019434206	0.007558	3.783221	up			
RNA54468|H-InvDB_1788_325	0.03648971	0.01717	3.796905	up			
RNA53999|H-InvDB_1281_388	0.026421677	0.011305	3.803951	up			
RNA54333|H-InvDB_1641_340	0.021721356	0.008758	3.805664	up			
RNA54528|H-InvDB_1851_321	0.021421751	0.008604	3.82139	up			
RNA62481|RNAz_3199_238	0.015034221	0.005374	3.827832	up			
RNA63315|RNAz_4033_229	0.000783878	9.09E-05	3.82801	up			
RNA54316|H-InvDB_1623_342	0.029348414	0.01298	3.84109	up			
RNA34335|NRED_466_2211	0.001872639	0.000302	3.846659	up			
RNA51988|UCSC_11604_300	0.000308228	2.53E-05	3.847159	up			
RNA54633|H-InvDB_1964_311	0.020393392	0.008063	3.852699	up			
RNA64787|RNAz_5508_200	0.001207059	0.000164	3.852784	up			
RNA54030|H-InvDB_1312_384	0.015510292	0.005609	3.866608	up			
RNA60122|RNAz_834_317	0.000503757	4.96E-05	3.869407	up			
RNA48868|UCSC_7851_1316	0.001158447	0.000154	3.879064	up			
RNA54447|H-InvDB_1764_327	0.033915702	0.015633	3.886205	up			
RNA48021|UCSC_6814_1580	3.47178E-06	4.22E-08	3.887707	up			
RNA63354|RNAz_4072_228	0.001650941	0.000253	3.891362	up			
RNA41192|RefSeq_3314_493	0.000554909	5.65E-05	3.892491	up			
RNA53989|H-InvDB_1271_389	0.003193576	0.000633	3.93442	up			
RNA61311|RNAz_2025_270	0.000167843	1.12E-05	3.935457	up			
RNA54501|H-InvDB_1822_322	0.010920119	0.003488	3.94254	up			
RNA62287|RNAz_3005_239	0.002441805	0.000435	3.945172	up			
RNA43654|UCSC_1437_3599	0.003700691	0.000775	3.960146	up			
RNA54427|H-InvDB_1742_330	0.032581273	0.014846	3.962202	up			
RNA58613|asoverlaps_141_2736	0.001150304	0.000153	3.971057	up			
RNA54338|H-InvDB_1646_340	0.021156875	0.008458	3.971362	up			

RNA54977|H-InvDB_2338_267	0.033239316	0.015236	3.973342	up			
RNA54596|H-InvDB_1923_315	0.016383812	0.006034	3.97411	up			
RNA50004|UCSC_9192_1006	0.002211644	0.000378	3.992169	up			
RNA53727|H-InvDB_1004_436	0.02135133	0.008565	4.002082	up			
RNA34193|NRED_324_2571	0.029509846	0.013071	4.006612	up			
RNA43061|UCSC_734_4558	0.000151157	9.83E-06	4.010711	up			
RNA53570|H-InvDB_841_490	0.000249037	1.91E-05	4.010762	up			
RNA54530|H-InvDB_1853_321	0.019122222	0.007395	4.023253	up			
RNA54819|H-InvDB_2163_288	0.017041558	0.006352	4.028472	up			
RNA54426|H-InvDB_1741_330	0.019663764	0.007683	4.04058	up			
RNA53870|H-InvDB_1151_408	0.007700216	0.002155	4.040834	up			
RNA53805|H-InvDB_1084_420	0.017813822	0.006743	4.042077	up			
RNA54452|H-InvDB_1770_327	0.03178368	0.014365	4.052251	up			
RNA64476|RNAz_5197_200	0.004309714	0.000959	4.061791	up			
RNA54502|H-InvDB_1823_322	0.019659456	0.007681	4.076202	up			
RNA54222|H-InvDB_1525_352	0.016658692	0.00617	4.081425	up			
RNA54315|H-InvDB_1622_342	0.020454466	0.008095	4.083231	up			
RNA54279|H-InvDB_1584_345	0.01713434	0.0064	4.105338	up			
RNA54956|H-InvDB_2314_269	3.44838E-06	4.11E-08	4.115807	up			
RNA54134|H-InvDB_1430_366	0.0207685	0.008257	4.118752	up			
RNA61841|RNAz_2555_249	0.002384438	0.00042	4.132103	up			
RNA55130|H-InvDB_2500_249	0.020865085	0.008307	4.14859	up			
RNA54072|H-InvDB_1365_377	0.007724517	0.002165	4.176166	up			
RNA54597|H-InvDB_1924_315	0.01777845	0.006724	4.17819	up			
RNA54629|H-InvDB_1960_311	0.017843734	0.00676	4.194061	up			
RNA58155|UCRs_470_201	0.004863523	0.001133	4.209311	up			
RNA53138|H-InvDB_400_647	0.002085054	0.00035	4.2147	up			
RNA54429|H-InvDB_1744_330	0.01956624	0.007631	4.220964	up			
RNA64731|RNAz_5452_200	0.00127323	0.000176	4.223502	up			
RNA54508|H-InvDB_1829_322	0.014060776	0.004925	4.243556	up			
RNA60842|RNAz_1556_279	0.002502802	0.00045	4.265969	up			
RNA54196|H-InvDB_1498_357	0.010000716	0.003096	4.277926	up			
RNA51737|UCSC_11327_403	0.000118755	7.16E-06	4.333757	up			
RNA51771|UCSC_11364_387	6.8183E-05	3.29E-06	4.3368	up			
RNA54958|H-InvDB_2318_269	0.015673239	0.005688	4.337194	up			
RNA54077|H-InvDB_1370_376	0.0001158	6.91E-06	4.366462	up			

RNA54449|H-InvDB_1767_327	0.012190918	0.004048	4.390646	up			
RNA54851|H-InvDB_2196_285	0.016175501	0.005933	4.398437	up			
RNA54317|H-InvDB_1624_342	0.029793875	0.013236	4.415568	up			
RNA53831|H-InvDB_1112_414	0.009478525	0.002878	4.417327	up			
RNA53215|H-InvDB_478_634	0.016034601	0.005863	4.419399	up			
RNA54037|H-InvDB_1321_383	0.003729814	0.000784	4.428683	up			
RNA53260|H-InvDB_523_623	0.009094806	0.002715	4.438772	up			
RNA64539|RNAz_5260_200	0.001278071	0.000177	4.453262	up			
RNA54742|H-InvDB_2077_297	0.011293524	0.003648	4.467343	up			
RNA54424|H-InvDB_1737_331	0.018059162	0.006864	4.472755	up			
RNA43539|UCSC_1295_3718	0.000400371	3.64E-05	4.480459	up			
RNA53997|H-InvDB_1279_388	0.023204299	0.009564	4.480886	up			
RNA54422|H-InvDB_1735_331	0.01473851	0.005238	4.506484	up			
RNA63969|RNAz_4689_215	0.00914433	0.002738	4.513753	up			
RNA38517|RefSeq_436_4033	0.014545879	0.005147	4.515031	up			
RNA54152|H-InvDB_1452_363	0.002090056	0.000351	4.522296	up			
RNA54351|H-InvDB_1661_339	0.013309593	0.004573	4.543378	up			
RNA53986|H-InvDB_1268_389	0.017241914	0.006454	4.548202	up			
RNA63097|RNAz_3815_232	0.001639625	0.000251	4.560654	up			
RNA53687|H-InvDB_963_448	0.002264439	0.000391	4.560737	up			
RNA54227|H-InvDB_1530_351	0.004470847	0.001007	4.582901	up			
RNA51454|UCSC_10995_517	2.33292E-05	7.03E-07	4.593932	up			
RNA54004|H-InvDB_1286_387	0.030452238	0.013595	4.634549	up			
RNA54506|H-InvDB_1827_322	0.009783397	0.003002	4.641935	up			
RNA51293|UCSC_10787_579	0.005541693	0.001358	4.646138	up			
RNA54379|H-InvDB_1689_336	0.017122786	0.006395	4.659958	up			
RNA57809|UCRs_124_279	0.0064948	0.001697	4.669653	up			
RNA54050|H-InvDB_1334_381	0.010547156	0.003328	4.67589	up			
RNA59550|RNAz_261_393	0.006445468	0.001679	4.676687	up			
RNA54615|H-InvDB_1945_312	0.004695926	0.001079	4.723026	up			
RNA43129|UCSC_812_4399	1.08283E-05	2.29E-07	4.7306	up			
RNA54318|H-InvDB_1625_342	0.014104025	0.004944	4.734278	up			
RNA54556|H-InvDB_1879_320	0.014032523	0.004913	4.736765	up			
RNA54396|H-InvDB_1709_333	0.014665493	0.005205	4.738999	up			
RNA53988|H-InvDB_1270_389	0.011535242	0.003753	4.748777	up			
RNA54815|H-InvDB_2158_288	0.003392056	0.000688	4.756309	up			

RNA53241|H-InvDB_504_629	0.012408264	0.004147	4.765614	up			
RNA53806|H-InvDB_1085_420	0.013841519	0.004821	4.772438	up			
RNA60960|RNAz_1674_277	0.001471325	0.000216	4.786718	up			
RNA53901|H-InvDB_1183_402	0.002779832	0.000522	4.814459	up			
RNA54000|H-InvDB_1282_388	0.009842103	0.003028	4.838424	up			
RNA54658|H-InvDB_1989_308	0.012784989	0.004319	4.843205	up			
RNA54818|H-InvDB_2161_288	0.009644052	0.002945	4.844806	up			
RNA54571|H-InvDB_1896_318	0.013200413	0.004519	4.857818	up			
RNA54258|H-InvDB_1561_348	0.014153918	0.004967	4.872747	up			
RNA54526|H-InvDB_1849_321	0.007756487	0.002179	4.882963	up			
RNA53398|H-InvDB_667_547	0.01306047	0.00445	4.936308	up			
RNA54483|H-InvDB_1804_324	0.006886106	0.00184	4.947211	up			
RNA54606|H-InvDB_1935_314	0.010297085	0.003219	4.977476	up			
RNA54509|H-InvDB_1830_322	0.027461082	0.011893	4.986294	up			
RNA54363|H-InvDB_1673_338	0.017041964	0.006352	4.991296	up			
RNA54543|H-InvDB_1866_321	0.003001252	0.00058	4.997	up			
RNA53229|H-InvDB_492_631	0.006736268	0.001785	4.998825	up			
RNA54638|H-InvDB_1969_311	0.01056791	0.003339	5.051099	up			
RNA54285|H-InvDB_1590_345	0.011640833	0.0038	5.051834	up			
RNA53652|H-InvDB_925_459	0.013060345	0.004449	5.054689	up			
RNA54377|H-InvDB_1687_336	0.023716869	0.009837	5.076205	up			
RNA54028|H-InvDB_1310_384	0.009461639	0.002871	5.076506	up			
RNA54354|H-InvDB_1664_339	0.01736372	0.006518	5.103735	up			
RNA39291|RefSeq_1259_2441	0.000881159	0.000106	5.152812	up			
RNA54444|H-InvDB_1761_328	0.008071888	0.002303	5.163444	up			
RNA49348|UCSC_8416_1179	0.000380491	3.38E-05	5.182807	up			
RNA54009|H-InvDB_1291_387	0.006285471	0.001624	5.189086	up			
RNA53944|H-InvDB_1226_396	0.021544889	0.008666	5.190857	up			
RNA53970|H-InvDB_1252_391	0.018159399	0.006915	5.203783	up			
RNA53233|H-InvDB_496_630	0.009705416	0.00297	5.233512	up			
RNA54576|H-InvDB_1902_318	0.006445197	0.001679	5.235971	up			
RNA53866|H-InvDB_1147_408	0.019729927	0.007719	5.23881	up			
RNA54341|H-InvDB_1651_339	0.006603205	0.001737	5.279241	up			
RNA58082|UCRs_397_210	0.000359366	3.13E-05	5.356766	up			
RNA53242|H-InvDB_505_629	0.011159231	0.003591	5.35949	up			
RNA53784|H-InvDB_1063_424	0.009367608	0.002832	5.382645	up			

RNA54939|H-InvDB_2295_272	0.011725458	0.003838	5.402562	up			
RNA54012|H-InvDB_1294_387	0.003640078	0.000758	5.435854	up			
RNA59687|RNAz_398_360	5.82832E-05	2.62E-06	5.439735	up			
RNA54391|H-InvDB_1704_334	0.000735568	8.35E-05	5.46654	up			
RNA54383|H-InvDB_1696_335	0.01587305	0.005785	5.496001	up			
RNA54096|H-InvDB_1390_373	0.004018237	0.000869	5.533379	up			
RNA39483|RefSeq_1469_2228	4.42781E-06	5.98E-08	5.553546	up			
RNA53065|H-InvDB_324_665	0.010435779	0.003279	5.566739	up			
RNA53957|H-InvDB_1239_393	0.007699754	0.002154	5.593338	up			
RNA53064|H-InvDB_323_665	0.008250727	0.002374	5.622056	up			
RNA39715|RefSeq_1715_2017	0.000224544	1.65E-05	5.639213	up			
RNA54570|H-InvDB_1895_318	0.008151028	0.002333	5.650479	up			
RNA63641|RNAz_4361_222	0.001429759	0.000207	5.683107	up			
RNA64197|RNAz_4918_207	0.00037799	3.35E-05	5.70558	up			
RNA53574|H-InvDB_846_488	0.001856111	0.000298	5.708002	up			
RNA53803|H-InvDB_1082_420	0.001239161	0.00017	5.770122	up			
RNA53214|H-InvDB_477_634	0.006038842	0.001533	5.801279	up			
RNA54693|H-InvDB_2026_303	0.005195793	0.001245	5.834549	up			
RNA54011|H-InvDB_1293_387	0.01453149	0.005141	5.835044	up			
RNA54505|H-InvDB_1826_322	0.012953986	0.004398	5.905451	up			
RNA53758|H-InvDB_1036_429	0.013805339	0.004803	5.937355	up			
RNA53154|H-InvDB_416_644	0.011568442	0.003767	5.975799	up			
RNA53167|H-InvDB_429_642	0.009522536	0.002896	5.996712	up			
RNA54287|H-InvDB_1592_345	0.008669348	0.002543	6.083848	up			
RNA96374|EvoFold_194_265	0.010000716	0.003096	6.087801	up			
RNA54376|H-InvDB_1686_336	0.002994388	0.000578	6.091085	up			
RNA54041|H-InvDB_1325_383	0.004162627	0.000912	6.091653	up			
RNA147065|hox-HOXA13-27	0.006580333	0.001728	6.118026	up			
RNA53062|H-InvDB_321_666	2.96233E-05	9.75E-07	6.15743	up			
RNA53995|H-InvDB_1277_388	0.007306027	0.002	6.193141	up			
RNA53757|H-InvDB_1035_429	0.001280797	0.000178	6.195153	up			
RNA53856|H-InvDB_1137_410	0.009756587	0.002992	6.245521	up			
RNA53663|H-InvDB_937_455	0.015002946	0.005359	6.291156	up			
RNA54398|H-InvDB_1711_333	0.014666945	0.005205	6.298887	up			
RNA54831|H-InvDB_2175_287	0.025581744	0.010855	6.449158	up			
RNA54202|H-InvDB_1504_356	0.004546707	0.001032	6.456091	up			

RNA54657|H-InvDB_1988_308	0.00973697	0.002982	6.472706	up			
RNA54164|H-InvDB_1464_360	0.006150849	0.001574	6.49078	up			
RNA53102|H-InvDB_361_653	4.73705E-05	1.91E-06	6.512308	up			
RNA54443|H-InvDB_1760_328	0.003640078	0.000758	6.531108	up			
RNA40019|RefSeq_2044_1712	0.000342571	2.93E-05	6.595993	up			
RNA53101|H-InvDB_360_653	0.005445565	0.001325	6.644933	up			
RNA54404|H-InvDB_1717_333	0.00572867	0.001421	6.734564	up			
RNA53238|H-InvDB_501_629	0.006270395	0.001618	6.74445	up			
RNA53194|H-InvDB_457_638	0.009262311	0.002785	6.909934	up			
RNA53040|H-InvDB_299_676	0.000720533	8.12E-05	6.970309	up			
RNA54915|H-InvDB_2267_276	0.000977148	0.000122	7.066098	up			
RNA53191|H-InvDB_454_640	0.003766339	0.000795	7.354358	up			
RNA53001|H-InvDB_260_701	0.008178111	0.002344	7.372225	up			
RNA53956|H-InvDB_1238_393	0.007263615	0.001983	7.403139	up			
RNA53220|H-InvDB_483_633	0.006707792	0.001774	7.413369	up			
RNA54102|H-InvDB_1396_372	0.005812416	0.001452	7.489078	up			
RNA54301|H-InvDB_1607_343	0.00835995	0.002417	7.52173	up			
RNA53133|H-InvDB_395_647	0.002639875	0.000485	7.551438	up			
RNA53703|H-InvDB_980_444	0.001600131	0.000242	7.69665	up			
RNA53255|H-InvDB_518_624	0.000484805	4.71E-05	7.714823	up			
RNA53198|H-InvDB_461_636	0.006057425	0.001541	7.785313	up			
RNA61404|RNAz_2118_268	3.65669E-05	1.31E-06	7.891542	up			
RNA53985|H-InvDB_1267_389	0.010996513	0.00352	7.921994	up			
RNA54237|H-InvDB_1540_351	0.004512701	0.001021	8.010195	up			
RNA54981|H-InvDB_2343_267	0.001785822	0.000282	8.077625	up			
RNA53150|H-InvDB_412_645	0.004103518	0.000895	8.153579	up			
RNA53240|H-InvDB_503_629	0.002278921	0.000395	8.180814	up			
RNA53089|H-InvDB_348_656	0.006574302	0.001726	8.235762	up			
RNA47773|UCSC_6518_1643	0.0006118	6.46E-05	8.268704	up			
RNA53168|H-InvDB_430_642	0.002949772	0.000566	8.28856	up			
RNA53100|H-InvDB_359_653	0.00033468	2.83E-05	8.327739	up			
RNA35126|NRED_1267_321	0.004894731	0.001144	8.450408	up			
RNA53257|H-InvDB_520_624	0.005159305	0.001233	8.49742	up			
RNA54405|H-InvDB_1718_333	0.013303143	0.00457	8.528814	up			
RNA53176|H-InvDB_438_641	0.002228625	0.000383	8.635202	up			
RNA53177|H-InvDB_439_641	0.002287437	0.000397	8.666364	up			

RNA49584|UCSC_8693_1117	3.46104E-06	4.16E-08	8.709083	up			
RNA63795|RNAz_4515_219	3.97037E-05	1.48E-06	8.798893	up			
RNA53169|H-InvDB_431_642	0.002113681	0.000356	8.846189	up			
RNA53579|H-InvDB_851_486	0.000764285	8.79E-05	8.914071	up			
RNA53021|H-InvDB_280_686	0.010101046	0.00314	9.01747	up			
RNA53014|H-InvDB_273_692	0.003796015	0.000804	9.102148	up			
RNA54135|H-InvDB_1431_366	0.003110337	0.00061	9.105448	up			
RNA54473|H-InvDB_1793_325	0.002795745	0.000526	9.273284	up			
RNA54814|H-InvDB_2156_288	0.004265797	0.000946	9.429549	up			
RNA53147|H-InvDB_409_646	0.001924153	0.000313	9.476368	up			
RNA53680|H-InvDB_955_450	0.003635197	0.000756	9.524359	up			
RNA53108|H-InvDB_367_651	0.005224172	0.001254	9.572802	up			
RNA55342|H-InvDB_2717_223	0.000297267	2.42E-05	9.615895	up			
RNA53042|H-InvDB_301_675	0.006750003	0.00179	9.871515	up			
RNA53139|H-InvDB_401_647	0.007856334	0.002219	9.87925	up			
RNA54197|H-InvDB_1499_357	0.005670109	0.0014	9.887764	up			
RNA53184|H-InvDB_446_641	0.006043924	0.001536	9.995324	up			
RNA53080|H-InvDB_339_659	0.001779058	0.00028	10.3234	up			
RNA54484|H-InvDB_1805_324	0.00511356	0.001217	10.32769	up			
RNA53068|H-InvDB_327_664	0.002649366	0.000488	10.33823	up			
RNA53057|H-InvDB_316_668	0.002911803	0.000556	10.42742	up			
RNA53975|H-InvDB_1257_390	0.003921857	0.000841	10.43751	up			
RNA53155|H-InvDB_417_644	0.004339386	0.000968	10.46133	up			
RNA54507|H-InvDB_1828_322	0.01051665	0.003315	10.75592	up			
RNA41013|RefSeq_3120_678	0.002530459	0.000456	10.85102	up			
RNA53195|H-InvDB_458_638	0.005908557	0.001486	10.97583	up			
RNA53045|H-InvDB_304_674	0.001666883	0.000256	11.00042	up			
RNA53207|H-InvDB_470_635	0.003258531	0.000651	11.5289	up			
RNA53081|H-InvDB_340_659	0.00248117	0.000445	11.8133	up			
RNA53206|H-InvDB_469_635	0.003011883	0.000583	11.96994	up			
RNA53123|H-InvDB_383_649	0.002745749	0.000512	12.65904	up			
RNA53672|H-InvDB_946_453	0.001353112	0.000192	12.90539	up			
RNA53141|H-InvDB_403_647	0.000262486	2.05E-05	14.40034	up			
RNA53016|H-InvDB_275_689	0.003026764	0.000588	20.87192	up			
RNA143598|rRNA_37_380	0.001689029	0.000261	2.168113	up			
RNA143579|rRNA_18_718	0.028235998	0.012324	2.406827	up			

RNA143595|rRNA_34_468	0.028452389	0.012454	2.439413	up			
RNA143600|rRNA_39_354	0.004068074	0.000884	2.636031	up			
RNA143588|rRNA_27_581	0.049783308	0.025411	2.732687	up			
RNA143592|rRNA_31_484	0.012293379	0.004094	4.185278	up			
RNA144104|snRNA_57_318	0.006936356	0.00186	2.020451	up			
RNA144117|snRNA_70_315	0.003376918	0.000684	2.220143	up			
RNA144065|snRNA_18_331	0.007378648	0.002026	2.241203	up			
RNA144163|snRNA_116_279	0.004923719	0.001153	2.38078	up			
RNA145890|SRP_RNA_4_310	0.003804583	0.000807	2.2376	up			
RNA146075|SRP_RNA_189_211	0.007771324	0.002185	3.45822	up			
RNA147785|p0889_imsncRNA704	0.01354419	0.004683	2.002621	up			
RNA146935|p0039_imsncRNA85	1.12468E-05	2.42E-07	2.005541	up			
RNA147756|p0860_imsncRNA668	0.008110722	0.002319	2.008107	up			
RNA147494|p0598_imsncRNA307	0.015696323	0.005699	2.009082	up			
RNA147316|p0420_imsncRNA832	0.04926965	0.025072	2.009191	up			
RNA147788|p0892_imsncRNA708	0.006781125	0.001801	2.010666	up			
RNA147090|p0194_imsncRNA293	0.001130449	0.00015	2.011723	up			
RNA147112|p0216_imsncRNA329	0.002368893	0.000416	2.018513	up			
RNA147664|p0768_imsncRNA553	0.018104518	0.006886	2.020237	up			
RNA147534|p0638_imsncRNA382	0.002218555	0.00038	2.020467	up			
RNA147719|p0823_imsncRNA623	0.002168219	0.000368	2.026344	up			
RNA147718|p0822_imsncRNA619	0.000694722	7.71E-05	2.029789	up			
RNA147418|p0522_imsncRNA111	7.84569E-05	4.03E-06	2.05998	up			
RNA147461|p0565_imsncRNA229	0.039263148	0.018827	2.064407	up			
RNA146984|p0088_imsncRNA153	0.004916545	0.00115	2.070195	up			
RNA147383|p0487_imsncRNA35	0.000203944	1.46E-05	2.088355	up			
RNA147052|p0156_imsncRNA246	0.001679322	0.000259	2.091177	up			
RNA147523|p0627_imsncRNA361	0.004146723	0.000907	2.093901	up			
RNA147441|p0545_imsncRNA172	0.010226939	0.00319	2.099552	up			
RNA147728|p0832_imsncRNA633	0.00578216	0.001441	2.100399	up			
RNA147267|p0371_imsncRNA788	0.004351114	0.000971	2.10152	up			
RNA147321|p0425_imsncRNA836	0.006512405	0.001703	2.114652	up			
RNA147565|p0669_imsncRNA429	0.018478693	0.007073	2.129514	up			
RNA147647|p0751_imsncRNA533	0.001104624	0.000145	2.131343	up			
RNA147752|p0856_imsncRNA662	0.000866293	0.000104	2.133647	up			
RNA147720|p0824_imsncRNA624	0.000283175	2.27E-05	2.137471	up			

RNA147667|p0771_imsncRNA556	0.005541106	0.001357	2.148487	up			
RNA147107|p0211_imsncRNA320	0.012962962	0.004402	2.151264	up			
RNA147796|p0900_imsncRNA719	0.004051652	0.000879	2.160693	up			
RNA147102|p0206_imsncRNA315	0.002351794	0.000412	2.16625	up			
RNA147031|p0135_imsncRNA215	0.005828813	0.001458	2.174974	up			
RNA147341|p0445_imsncRNA845	0.028231552	0.01232	2.19055	up			
RNA147005|p0109_imsncRNA184	0.001023146	0.000131	2.193241	up			
RNA147228|p0332_imsncRNA734	0.006498615	0.001698	2.199139	up			
RNA147068|p0172_imsncRNA264	0.011103159	0.003568	2.225446	up			
RNA147093|p0197_imsncRNA298	5.81184E-05	2.61E-06	2.231925	up			
RNA147598|p0702_imsncRNA473	0.01037841	0.003255	2.246883	up			
RNA147556|p0660_imsncRNA417	0.005763582	0.001434	2.257935	up			
RNA147656|p0760_imsncRNA543	0.022057936	0.008937	2.274308	up			
RNA147617|p0721_imsncRNA496	0.00337553	0.000684	2.291678	up			
RNA147041|p0145_imsncRNA232	0.011412666	0.003699	2.304241	up			
RNA147254|p0358_imsncRNA776	0.034642447	0.016075	2.328206	up			
RNA147020|p0124_imsncRNA201	0.003167209	0.000625	2.332843	up			
RNA147770|p0874_imsncRNA684	0.013751853	0.00478	2.360186	up			
RNA147627|p0731_imsncRNA507	0.000813159	9.52E-05	2.365274	up			
RNA147124|p0228_imsncRNA356	0.009959419	0.00308	2.366503	up			
RNA147330|p0434_imsncRNA841	0.000158811	1.04E-05	2.401524	up			
RNA146942|p0046_imsncRNA95	0.000868729	0.000104	2.405906	up			
RNA147772|p0876_imsncRNA687	0.003190979	0.000632	2.4129	up			
RNA147064|p0168_imsncRNA259	7.78017E-05	3.98E-06	2.431898	up			
RNA146964|p0068_imsncRNA128	0.000408492	3.75E-05	2.441774	up			
RNA146987|p0091_imsncRNA157	0.017760508	0.006717	2.454115	up			
RNA147292|p0396_imsncRNA814	0.001112297	0.000146	2.497962	up			
RNA147304|p0408_imsncRNA823	0.008452427	0.002455	2.536416	up			
RNA147327|p0431_imsncRNA841	0.007885091	0.002231	2.544881	up			
RNA147506|p0610_imsncRNA332	0.005097515	0.001211	2.590824	up			
RNA147614|p0718_imsncRNA492	0.00126275	0.000174	2.599521	up			
RNA147125|p0229_imsncRNA356	0.002156979	0.000366	2.65854	up			
RNA147482|p0586_imsncRNA285	0.000236574	1.78E-05	2.665887	up			
RNA147051|p0155_imsncRNA246	0.001165757	0.000156	2.690673	up			
RNA147527|p0631_imsncRNA366	0.001244283	0.000171	2.740874	up			
RNA147213|p0317_imsncRNA698	0.002241993	0.000386	2.798812	up			

RNA146971|p0075_imsncRNA136	0.00289038	0.000551	2.875327	up			
RNA147315|p0419_imsncRNA832	0.00542967	0.00132	2.906235	up			
RNA147584|p0688_imsncRNA452	0.001076435	0.00014	2.926864	up			
RNA147459|p0563_imsncRNA225	0.007509225	0.002081	3.074174	up			
RNA147307|p0411_imsncRNA825	0.000118514	7.13E-06	3.105586	up			
RNA147529|p0633_imsncRNA370	0.004852598	0.001129	3.186415	up			
RNA147283|p0387_imsncRNA804	0.001124019	0.000149	3.287224	up			
RNA146923|p0027_imsncRNA71	0.003442248	0.000703	3.369211	up			
RNA147632|p0736_imsncRNA513	0.001367309	0.000194	3.452674	up			
RNA146941|p0045_imsncRNA95	0.000266212	2.09E-05	3.608729	up			
RNA147396|p0500_imsncRNA62	0.000847803	0.000101	3.621916	up			
RNA146993|p0097_imsncRNA168	9.43943E-05	5.27E-06	4.04211	up			
RNA147678|p0782_imsncRNA568	0.00045031	4.27E-05	4.131673	up			
RNA147470|p0574_imsncRNA252	7.46204E-05	3.75E-06	4.373186	up			
RNA147398|p0502_imsncRNA64	0.012735883	0.004297	4.702748	up			
RNA147471|p0575_imsncRNA253	0.000101243	5.8E-06	5.911053	up			
RNA147284|p0388_imsncRNA805	0.000102687	5.91E-06	7.139074	up			
RNA164434|XLOC_005438	0.001256584	0.000173	3.058149	down	ENST00000314481	antisense	RP11-544L8__B.4
RNA39733|RefSeq_1735_2006	0.00166203	0.000255	3.131732	down	ENST00000314481	antisense	RP11-544L8__B.4
RNA34333|NRED_464_2213	5.76191E-05	2.58E-06	2.299337	down	ENST00000318291	antisense	RP11-218M22.1
RNA35496|ENCODE_360_1711	0.000659539	7.17E-05	2.05471	down	ENST00000366278	antisense	AC105053.3
RNA44295|UCSC_2216_3026	0.002136779	0.000361	2.367994	down	ENST00000376593	antisense	AC096670.3
RNA48089|UCSC_6895_1560	6.91547E-05	3.36E-06	6.441174	down	ENST00000377178	antisense	RP11-195E11.3
RNA164854|XLOC_006178	0.000352548	3.05E-05	3.112582	down	ENST00000411728	antisense	AC002451.3
RNA35086|NRED_1227_577	9.46306E-06	1.88E-07	4.239173	down	ENST00000411904	antisense	RP11-251M1.1
RNA177677|ENST00000413969	0.002188399	0.000373	2.786308	down	ENST00000413969	antisense	HOXD-AS1
RNA177718|ENST00000415561	0.000351966	3.04E-05	2.266852	down	ENST00000415561	antisense	AC009948.3
RNA34957|NRED_1098_1031	0.001633917	0.000249	2.642328	down	ENST00000415675	antisense	CTA-134P22.2
RNA37321|ENCODE_2192_437	0.000265778	2.08E-05	3.514385	down	ENST00000418076	antisense	RP11-37E23.5
RNA177713|ENST00000419746	0.000190648	1.33E-05	2.230556	down	ENST00000419746	antisense	AC009948.3
RNA36169|ENCODE_1039_708	0.000235013	1.76E-05	2.570377	down	ENST00000423551	antisense	RP11-354E11.2
RNA177680|ENST00000425005	0.003580856	0.00074	2.71105	down	ENST00000425005	antisense	HOXD-AS1
RNA35881|ENCODE_751_823	0.005136149	0.001224	2.212347	down	ENST00000431157	antisense	RP11-354E11.2
RNA158110|XLOC_000942	5.71063E-07	1.65E-09	5.4228	down	ENST00000432195	antisense	RP4-575N6.4
RNA159857|XLOC_010477	0.013733456	0.00477	2.304385	down	ENST00000434547	antisense	STK24-AS1
RNA35572|ENCODE_440_1351	5.40564E-05	2.34E-06	2.353758	down	ENST00000437232	antisense	RP11-124N14.4

RNA43178|UCSC_866_4298	0.000182821	1.26E-05	3.354892	down	ENST00000438047	antisense	AC011899.9
RNA36191|ENCODE_1061_697	0.03164567	0.014285	2.755506	down	ENST00000442176	antisense	AC005550.4
RNA58628|asoverlaps_158_2673	0.000280358	2.24E-05	2.913988	down	ENST00000442449	antisense	RP1-69D17.3
RNA45883|UCSC_4215_2191	0.000501462	4.94E-05	2.200033	down	ENST00000446442	antisense	ATP11A-AS1
RNA177681|ENST00000452365	0.004838353	0.001124	2.74996	down	ENST00000452365	antisense	HOXD-AS1
RNA35794|ENCODE_663_882	0.002665458	0.000492	2.085981	down	ENST00000452647	antisense	RP1-199J3.3
RNA45198|UCSC_3336_2471	4.57143E-05	1.8E-06	2.538941	down	ENST00000452714	antisense	AC003077.1
RNA36954|ENCODE_1824_497	0.000831555	9.79E-05	3.300369	down	ENST00000454305	antisense	RP4-706A16.2
RNA177678|ENST00000552156	0.002476834	0.000444	2.667649	down	ENST00000456876	antisense	HOXD-AS1
RNA177679|ENST00000456876	0.001984566	0.000326	2.738782	down	ENST00000456876	antisense	HOXD-AS1
RNA40994|RefSeq_3099_702	0.04904381	0.024923	2.000106	down	ENST00000480632	antisense	RP4-555L14.5
RNA40918|RefSeq_3016_787	0.031265862	0.014068	2.077673	down	ENST00000480632	antisense	RP4-555L14.5
RNA162831|XLOC_002774	0.000257657	1.99E-05	4.169873	down	ENST00000482766	antisense	RP11-768G7.3
RNA162823|XLOC_002767	0.018185817	0.006926	2.66877	down	ENST00000497854	antisense	LSAMP-AS2
RNA34491|NRED_623_1917	1.69851E-05	4.46E-07	3.07111	down	ENST00000502125	antisense	RP11-327J17.3
RNA34490|NRED_622_1917	3.5716E-05	1.26E-06	3.071259	down	ENST00000502125	antisense	RP11-327J17.3
RNA163848|XLOC_004435	0.000358737	3.12E-05	2.047945	down	ENST00000512650	antisense	CTC-564N23.2
RNA59094|asoverlaps_762_993	2.44071E-05	7.48E-07	2.366718	down	ENST00000512856	antisense	CTD-2260A17.1
RNA49236|UCSC_8272_1212	0.004049895	0.000878	2.099216	down	ENST00000514864	antisense	RP11-806K15.1
RNA163306|XLOC_004036	0.025004754	0.010544	2.115035	down	ENST00000514879	antisense	AC004066.3
RNA50370|UCSC_9646_902	0.000222594	1.64E-05	2.521612	down	ENST00000519753	antisense	CTD-3107M8.4
RNA174886|ENST00000522193	0.000212556	1.54E-05	2.033186	down	ENST00000523364	antisense	HOXA-AS2
RNA51820|UCSC_11413_368	1.22744E-06	7.55E-09	4.712183	down	ENST00000524304	antisense	HOXA-AS3
RNA43630|UCSC_1402_3620	1.20227E-05	2.64E-07	4.053482	down	ENST00000528549	antisense	RP11-385N17.1
RNA34269|NRED_400_2352	1.33656E-05	3.09E-07	7.659514	down	ENST00000529215	antisense	CTD-2562J17.7
RNA50695|UCSC_10058_789	0.001352436	0.000192	2.411541	down	ENST00000549804	antisense	SNHG14
RNA46135|UCSC_4513_2102	0.008258127	0.002377	3.20758	down	ENST00000553843	antisense	RP4-755D9.1
RNA34533|NRED_665_1838	2.09116E-05	5.97E-07	3.900556	down	ENST00000560778	antisense	RP11-2E17.1
RNA34846|NRED_980_1313	2.46297E-05	7.61E-07	4.107547	down	ENST00000560778	antisense	RP11-2E17.1
RNA35030|NRED_1171_840	4.75119E-05	1.92E-06	2.748876	down	ENST00000561344	antisense	RP11-327J17.3
RNA160278|XLOC_011516	0.000388527	3.48E-05	2.20482	down	ENST00000563004	antisense	RP11-352D13.6
RNA58847|asoverlaps_444_1823	4.05669E-06	5.23E-08	4.528671	down	ENST00000581080	antisense	CTD-2267D19.2
RNA175350|ENST00000376608	9.00144E-05	4.91E-06	2.788703	down	ENST00000376608	lincRNA	RP13-554M15.6
RNA175346|ENST00000538369	0.001896885	0.000307	2.857031	down	ENST00000376608	lincRNA	RP13-554M15.6
RNA46144|UCSC_4522_2100	0.000659916	7.17E-05	2.151387	down	ENST00000411727	lincRNA	RP11-33A14.1
RNA35290|ENCODE_149_2503	1.93873E-05	5.39E-07	4.467717	down	ENST00000412204	lincRNA	BX571672.2

RNA165746|XLOC_008115	0.011712302	0.003831	2.826963	down	ENST00000412485	lincRNA	GS1-600G8.5
RNA35539|ENCODE_404_1497	0.012915546	0.004379	3.020781	down	ENST00000412485	lincRNA	GS1-600G8.5
RNA40525|RefSeq_2585_1194	6.78473E-07	2.58E-09	2.752739	down	ENST00000413221	lincRNA	EPB41L4A-AS1
RNA35646|ENCODE_514_1109	0.000129385	8.02E-06	2.906822	down	ENST00000413897	lincRNA	BX571672.2
RNA35765|ENCODE_634_906	0.021024078	0.008389	2.097721	down	ENST00000414407	lincRNA	LINC00398
RNA36815|ENCODE_1685_527	3.97305E-05	1.49E-06	2.662165	down	ENST00000415575	lincRNA	RP11-203H2.2
RNA176670|ENST00000444349	0.000242507	1.84E-05	4.017549	down	ENST00000415842	lincRNA	RP11-182I10.3
RNA36651|ENCODE_1521_559	0.00065293	7.06E-05	6.329492	down	ENST00000417422	lincRNA	AC079630.2
RNA159313|XLOC_009712	0.001136546	0.000151	6.657894	down	ENST00000417422	lincRNA	AC079630.2
RNA36676|ENCODE_1546_554	0.009015998	0.002683	2.34051	down	ENST00000417650	lincRNA	RP11-439L18.1
RNA37246|ENCODE_2116_446	4.78293E-06	6.76E-08	3.388409	down	ENST00000418372	lincRNA	RP11-67B16.1
RNA37726|ENCODE_2598_358	0.000167649	1.12E-05	2.424303	down	ENST00000419061	lincRNA	RP1-69D17.4
RNA37353|ENCODE_2224_432	6.41963E-05	3.02E-06	3.723586	down	ENST00000419428	lincRNA	RP11-356N1.2
RNA36658|ENCODE_1528_557	0.000758101	8.68E-05	2.646622	down	ENST00000420598	lincRNA	BX571672.2
RNA36771|ENCODE_1641_537	4.62145E-07	1.06E-09	5.044694	down	ENST00000420825	lincRNA	RP1-251M9.2
RNA36740|ENCODE_1610_543	2.0187E-05	5.67E-07	8.513246	down	ENST00000420988	lincRNA	U82670.4
RNA35746|ENCODE_615_921	8.46695E-06	1.6E-07	9.675834	down	ENST00000420988	lincRNA	U82670.4
RNA165900|XLOC_008260	2.1815E-05	6.33E-07	11.77531	down	ENST00000420988	lincRNA	U82670.4
RNA36220|ENCODE_1090_686	0.00232805	0.000406	2.729574	down	ENST00000421019	lincRNA	RP11-290F20.3
RNA35355|ENCODE_217_2186	0.000489298	4.77E-05	2.422116	down	ENST00000421976	lincRNA	AC109642.1
RNA48238|UCSC_7090_1512	0.000679834	7.49E-05	2.672286	down	ENST00000421976	lincRNA	AC109642.1
RNA51017|UCSC_10438_679	2.23263E-06	2.05E-08	5.852733	down	ENST00000421976	lincRNA	AC109642.1
RNA162447|XLOC_014121	0.032565344	0.014834	2.339845	down	ENST00000422199	lincRNA	LINC00162
RNA35396|ENCODE_259_2010	5.64544E-05	2.5E-06	4.427707	down	ENST00000422459	lincRNA	RP11-290F20.3
RNA35662|ENCODE_530_1058	5.46245E-05	2.38E-06	4.609896	down	ENST00000422459	lincRNA	RP11-290F20.3
RNA36760|ENCODE_1630_539	4.09707E-05	1.55E-06	4.990395	down	ENST00000422459	lincRNA	RP11-290F20.3
RNA165545|XLOC_007775	0.002767674	0.000518	2.360068	down	ENST00000423380	lincRNA	RP11-305L7.1
RNA37401|ENCODE_2272_425	0.000585933	6.1E-05	3.582183	down	ENST00000423924	lincRNA	RP11-535M15.1
RNA37812|ENCODE_2684_335	0.00245458	0.000438	3.348531	down	ENST00000424181	lincRNA	RP11-160H22.3
RNA36716|ENCODE_1586_547	0.001023721	0.000131	2.025606	down	ENST00000424725	lincRNA	RP4-782L23.1
RNA36878|ENCODE_1748_513	0.000108621	6.36E-06	2.395465	down	ENST00000424820	lincRNA	BX004987.3
RNA37453|ENCODE_2324_415	5.19476E-05	2.21E-06	2.320661	down	ENST00000426012	lincRNA	RP1-122P22.2
RNA165915|XLOC_008278	0.005009975	0.001182	2.333714	down	ENST00000426699	lincRNA	AC010084.1
RNA39266|RefSeq_1234_2469	0.000349182	3.01E-05	2.573563	down	ENST00000427111	lincRNA	RP11-118B22.2
RNA175456|ENST00000427111	0.000105144	6.1E-06	2.647787	down	ENST00000427111	lincRNA	RP11-118B22.2
RNA36612|ENCODE_1482_567	1.04443E-05	2.16E-07	2.998606	down	ENST00000427579	lincRNA	AC007403.3

RNA49278|UCSC_8328_1198	0.00023945	1.81E-05	3.034917	down	ENST00000428624	lincRNA	BX004987.5
RNA35291|ENCODE_150_2495	1.03714E-05	2.13E-07	4.607122	down	ENST00000428624	lincRNA	BX004987.5
RNA36115|ENCODE_985_728	0.001916502	0.000312	2.16638	down	ENST00000430296	lincRNA	RP1-69D17.4
RNA37925|ENCODE_2798_292	0.000511712	5.08E-05	2.20394	down	ENST00000431700	lincRNA	BX004987.5
RNA36933|ENCODE_1803_501	0.000175753	1.2E-05	2.532802	down	ENST00000431700	lincRNA	BX004987.5
RNA37939|ENCODE_2812_287	1.57255E-05	3.95E-07	2.42238	down	ENST00000432334	lincRNA	RP1-122P22.2
RNA37517|ENCODE_2389_403	0.013292379	0.004565	2.254769	down	ENST00000432452	lincRNA	RP11-464C19.3
RNA36976|ENCODE_1846_493	0.000697975	7.76E-05	3.002094	down	ENST00000433724	lincRNA	AC116609.1
RNA35645|ENCODE_513_1109	0.000102105	5.87E-06	2.345725	down	ENST00000433980	lincRNA	BX004987.5
RNA161446|XLOC_002070	0.000455668	4.34E-05	2.590525	down	ENST00000434020	lincRNA	AC093609.1
RNA35720|ENCODE_589_952	0.000495987	4.86E-05	2.629906	down	ENST00000434020	lincRNA	AC093609.1
RNA161896|XLOC_001808	1.68792E-05	4.4E-07	2.581361	down	ENST00000434645	lincRNA	AC007283.5
RNA35243|ENCODE_100_2860	0.015533738	0.005621	2.010498	down	ENST00000435574	lincRNA	RP5-855F14.1
RNA38798|RefSeq_739_3273	0.012277398	0.004086	2.021276	down	ENST00000435944	lincRNA	CASC2
RNA38835|RefSeq_776_3173	0.002043687	0.00034	2.546066	down	ENST00000435944	lincRNA	CASC2
RNA175001|ENST00000435944	0.004393306	0.000984	2.809295	down	ENST00000435944	lincRNA	CASC2
RNA39607|RefSeq_1599_2113	1.85402E-05	5.04E-07	3.309511	down	ENST00000437047	lincRNA	AC034187.2
RNA36499|ENCODE_1369_589	0.016617408	0.00615	3.450039	down	ENST00000439046	lincRNA	AC019117.2
RNA175005|ENST00000439517	0.000648978	7.01E-05	2.485169	down	ENST00000439517	lincRNA	CASC2
RNA40691|RefSeq_2768_1042	0.000361345	3.15E-05	3.456508	down	ENST00000441855	lincRNA	RP1-251M9.1
RNA40792|RefSeq_2875_931	9.77981E-05	5.53E-06	3.506958	down	ENST00000441855	lincRNA	RP1-251M9.1
RNA36874|ENCODE_1744_514	0.000273571	2.16E-05	3.631078	down	ENST00000441855	lincRNA	RP1-251M9.1
RNA35668|ENCODE_536_1044	0.000210545	1.52E-05	4.102016	down	ENST00000441855	lincRNA	RP1-251M9.1
RNA37957|ENCODE_2830_279	0.000400348	3.64E-05	2.514672	down	ENST00000442663	lincRNA	BX004987.3
RNA161927|XLOC_001852	0.010042493	0.003114	2.511796	down	ENST00000443971	lincRNA	AC122136.2
RNA37801|ENCODE_2673_338	0.005297624	0.001278	2.022461	down	ENST00000446290	lincRNA	RP11-274B18.2
RNA165476|XLOC_007722	0.007724021	0.002164	2.028382	down	ENST00000446290	lincRNA	RP11-274B18.2
RNA36964|ENCODE_1834_495	0.003693711	0.000773	2.117145	down	ENST00000446290	lincRNA	RP11-274B18.2
RNA41002|RefSeq_3109_693	4.69869E-07	1.11E-09	11.04391	down	ENST00000446372	lincRNA	SFTA1P
RNA37539|ENCODE_2411_399	1.36461E-07	1.34E-10	11.8381	down	ENST00000446372	lincRNA	SFTA1P
RNA35806|ENCODE_675_873	1.39086E-07	1.39E-10	12.20437	down	ENST00000446372	lincRNA	SFTA1P
RNA37998|ENCODE_2871_254	2.13163E-07	2.74E-10	12.67512	down	ENST00000446372	lincRNA	SFTA1P
RNA36454|ENCODE_1324_600	0.000462158	4.42E-05	2.473378	down	ENST00000446476	lincRNA	RP5-826L7.1
RNA37240|ENCODE_2110_447	0.004445919	0.000999	3.149861	down	ENST00000448570	lincRNA	RP11-370B11.3
RNA36362|ENCODE_1232_628	0.003467832	0.000709	2.644081	down	ENST00000449235	lincRNA	RP11-151D14.1
RNA36087|ENCODE_957_740	9.63392E-06	1.93E-07	2.574482	down	ENST00000450174	lincRNA	RP1-251M9.1

RNA35477|ENCODE_341_1761	2.36671E-05	7.17E-07	6.986776	down	ENST00000450911	lincRNA	RP11-327I22.8
RNA37385|ENCODE_2256_428	0.000166713	1.11E-05	3.729437	down	ENST00000451023	lincRNA	RP11-356N1.2
RNA36504|ENCODE_1374_588	2.00967E-06	1.72E-08	4.096801	down	ENST00000451152	lincRNA	RP11-63P12.6
RNA36983|ENCODE_1853_491	0.000531611	5.34E-05	5.338602	down	ENST00000451341	lincRNA	RP1-35C21.1
RNA36260|ENCODE_1130_672	2.33441E-06	2.22E-08	4.374843	down	ENST00000451596	lincRNA	RP11-63P12.6
RNA37277|ENCODE_2148_442	0.010959299	0.003505	3.459704	down	ENST00000451792	lincRNA	AC017060.1
RNA36292|ENCODE_1162_658	0.009448529	0.002865	2.630459	down	ENST00000452366	lincRNA	RP3-471M13.2
RNA36322|ENCODE_1192_645	0.000274774	2.18E-05	2.807815	down	ENST00000452503	lincRNA	SKINTL
RNA36519|ENCODE_1389_583	0.004816049	0.001118	2.61479	down	ENST00000453787	lincRNA	RP11-151D14.1
RNA36483|ENCODE_1353_594	0.006062242	0.001542	3.004717	down	ENST00000453787	lincRNA	RP11-151D14.1
RNA37587|ENCODE_2459_388	0.004636749	0.00106	3.375362	down	ENST00000454003	lincRNA	AC019117.2
RNA175000|ENST00000414722	0.000933261	0.000115	2.382753	down	ENST00000454857	lincRNA	CASC2
RNA37202|ENCODE_2072_453	0.00151151	0.000224	3.492016	down	ENST00000455011	lincRNA	RP3-460G2.2
RNA40118|RefSeq_2146_1628	0.002167353	0.000368	2.175898	down	ENST00000455084	lincRNA	TTTY10
RNA175257|ENST00000522771	0.009965538	0.003083	2.576788	down	ENST00000455531	lincRNA	MEG3
RNA44830|UCSC_2881_2670	0.005776101	0.001439	2.627326	down	ENST00000455531	lincRNA	MEG3
RNA175258|ENST00000424076	0.00712899	0.001932	2.692771	down	ENST00000455531	lincRNA	MEG3
RNA175275|ENST00000455531	0.005773818	0.001438	2.798882	down	ENST00000455531	lincRNA	MEG3
RNA36517|ENCODE_1387_583	3.07365E-05	1.02E-06	2.392471	down	ENST00000455981	lincRNA	RP11-344B5.2
RNA37590|ENCODE_2462_388	0.000857904	0.000102	3.918365	down	ENST00000456460	lincRNA	BX004987.5
RNA37748|ENCODE_2620_352	5.81184E-05	2.6E-06	4.138145	down	ENST00000456795	lincRNA	RP3-331H24.4
RNA35416|ENCODE_279_1958	0.000271125	2.14E-05	2.889174	down	ENST00000457033	lincRNA	AC073130.1
RNA37621|ENCODE_2493_381	0.000275027	2.18E-05	4.031837	down	ENST00000457890	lincRNA	AC063976.3
RNA37116|ENCODE_1986_470	0.001110203	0.000146	3.769969	down	ENST00000457989	lincRNA	AC079630.2
RNA177347|ENST00000480258	0.000218422	1.59E-05	2.052921	down	ENST00000480258	lincRNA	DCP1A
RNA42718|UCSC_308_5871	7.69468E-05	3.93E-06	2.062933	down	ENST00000480258	lincRNA	DCP1A
RNA162852|XLOC_003239	6.34237E-06	1.03E-07	7.609409	down	ENST00000488425	lincRNA	RP11-88I21.2
RNA50305|UCSC_9565_926	1.03077E-05	2.11E-07	8.154584	down	ENST00000488425	lincRNA	RP11-88I21.2
RNA45432|UCSC_3636_2366	1.91398E-05	5.28E-07	7.045373	down	ENST00000499425	lincRNA	RP11-16M8.2
RNA160542|XLOC_011755	0.005373922	0.001302	2.749474	down	ENST00000499966	lincRNA	CTD-2258A20.4
RNA45994|UCSC_4341_2147	0.001163365	0.000155	2.88806	down	ENST00000499966	lincRNA	CTD-2258A20.4
RNA163503|XLOC_004176	0.000359366	3.13E-05	4.607297	down	ENST00000502661	lincRNA	RP11-287F9.2
RNA34324|NRED_455_2237	2.26028E-05	6.7E-07	2.063602	down	ENST00000507241	lincRNA	CTC-428G20.3
RNA40363|RefSeq_2411_1378	0.000133069	8.31E-06	4.101718	down	ENST00000508732	lincRNA	CTD-2536I1.1
RNA165150|XLOC_006808	0.000540591	5.45E-05	3.419119	down	ENST00000520370	lincRNA	RP11-246K15.1
RNA33799|lncRNAdb_6_8708	0.002020947	0.000334	2.244836	down	ENST00000534336	lincRNA	MALAT1

RNA175326|ENST00000542980	0.019339412	0.00751	2.019943	down	ENST00000536673	lincRNA	RP11-76C10.5
RNA175327|ENST00000536673	0.021878673	0.008845	2.029547	down	ENST00000536673	lincRNA	RP11-76C10.5
RNA175605|ENST00000536729	0.007238566	0.001974	2.249471	down	ENST00000536729	lincRNA	RP11-444D3.1
RNA175604|ENST00000446891	0.005812539	0.001452	2.270787	down	ENST00000536729	lincRNA	RP11-444D3.1
RNA175363|ENST00000537961	0.0001313	8.18E-06	2.216443	down	ENST00000537961	lincRNA	RP11-598F7.6
RNA175325|ENST00000537998	6.51172E-06	1.07E-07	5.865062	down	ENST00000537998	lincRNA	RP11-243M5.1
RNA39616|RefSeq_1609_2099	0.020914683	0.008331	2.0543	down	ENST00000541282	lincRNA	RMST
RNA177177|ENST00000547523	0.000219804	1.61E-05	5.813101	down	ENST00000547523	lincRNA	RP11-370I10.2
RNA177166|ENST00000547799	2.55856E-06	2.55E-08	5.046492	down	ENST00000547799	lincRNA	RP1-197B17.3
RNA159483|XLOC_009880	0.000655946	7.11E-05	2.053875	down	ENST00000548846	lincRNA	RP3-473L9.4
RNA49836|UCSC_8997_1047	0.000678413	7.46E-05	5.632857	down	ENST00000548886	lincRNA	RMST
RNA177651|ENST00000552663	0.001796086	0.000284	2.367106	down	ENST00000552663	lincRNA	RP3-473L9.4
RNA33806|lncRNAdb_13_3454	0.007434522	0.00205	2.029642	down	ENST00000553575	lincRNA	DIO3OS
RNA50090|UCSC_9298_985	0.002288709	0.000397	2.041875	down	ENST00000553575	lincRNA	DIO3OS
RNA174929|ENST00000555332	5.81184E-05	2.61E-06	4.640434	down	ENST00000555332	lincRNA	CTD-2536I1.1
RNA159916|XLOC_010967	0.001234083	0.000169	2.516383	down	ENST00000555688	lincRNA	CTD-2552B11.4
RNA54481|H-InvDB_1801_324	2.75065E-05	8.81E-07	2.065074	down	ENST00000555882	lincRNA	DIO3OS
RNA174931|ENST00000556899	5.72543E-05	2.55E-06	4.356473	down	ENST00000556899	lincRNA	CTD-2536I1.1
RNA160052|XLOC_011081	0.034215238	0.015812	2.250022	down	ENST00000557526	lincRNA	RP11-7F17.3
RNA47155|UCSC_5756_1817	0.012078458	0.003998	2.52934	down	ENST00000557526	lincRNA	RP11-7F17.3
RNA51798|UCSC_11391_376	4.67807E-05	1.87E-06	3.020037	down	ENST00000558575	lincRNA	RP11-293M10.5
RNA159312|XLOC_010052	0.000336444	2.85E-05	5.034549	down	ENST00000563933	lincRNA	RP11-476D10.1
RNA160163|XLOC_011175	0.006233981	0.001604	3.007935	down	ENST00000565893	lincRNA	PWRN1
RNA160424|XLOC_011872	0.00051188	5.08E-05	3.834046	down	ENST00000566787	lincRNA	RP11-27M24.3
RNA160167|XLOC_011178	0.008397834	0.002432	2.149767	down	ENST00000567246	lincRNA	PWRN2
RNA58866|asoverlaps_465_1777	0.000547758	5.56E-05	3.286769	down	ENST00000568686	lincRNA	RP4-555D20.2
RNA34602|NRED_734_1746	0.000386325	3.45E-05	2.54426	down	ENST00000570269	lincRNA	RP11-553L6.5
RNA34568|NRED_700_1794	3.21442E-05	1.09E-06	2.595839	down	ENST00000570269	lincRNA	RP11-553L6.5
RNA34550|NRED_682_1823	6.25003E-06	1.01E-07	2.741113	down	ENST00000570269	lincRNA	RP11-553L6.5
RNA39869|RefSeq_1882_1847	0.00017776	1.22E-05	2.077983	down	ENST00000571091	lincRNA	MIR22HG
RNA40302|RefSeq_2345_1434	9.96943E-05	5.67E-06	2.079049	down	ENST00000571091	lincRNA	MIR22HG
RNA39121|RefSeq_1083_2654	0.00013113	8.16E-06	2.089427	down	ENST00000571091	lincRNA	MIR22HG
RNA40388|RefSeq_2441_1351	0.000105333	6.12E-06	2.11227	down	ENST00000571091	lincRNA	MIR22HG
RNA177351|ENST00000334146	0.000109597	6.44E-06	2.138189	down	ENST00000571091	lincRNA	MIR22HG
RNA178567|ENST00000435523	1.52717E-06	1.12E-08	3.064241	down	ENST00000579072	lincRNA	AC058791.2
RNA178568|ENST00000416999	1.86722E-06	1.56E-08	3.537238	down	ENST00000580227	lincRNA	AC058791.2

RNA178569|ENST00000431189	1.08442E-05	2.3E-07	2.255309	down	ENST00000581076	lincRNA	AC058791.2
RNA34905|NRED_1039_1173	2.22796E-05	6.55E-07	2.949045	down	ENST00000581076	lincRNA	AC058791.2
RNA34858|NRED_992_1278	4.82156E-05	1.96E-06	2.544783	down	ENST00000581174	lincRNA	AC058791.2
RNA38951|RefSeq_897_2948	5.29123E-05	2.27E-06	2.556672	down	ENST00000581174	lincRNA	AC058791.2
RNA176513|ENST00000501730	5.69568E-05	2.53E-06	3.634049	down	ENST00000583490	lincRNA	RP11-627G18.3
RNA178566|ENST00000423414	1.21869E-05	2.69E-07	2.651021	down	ENST00000584405	lincRNA	AC058791.2
RNA178565|ENST00000429901	2.73615E-06	2.82E-08	3.481043	down	ENST00000585254	lincRNA	AC058791.2
RNA40182|RefSeq_2214_1569	3.51855E-05	1.24E-06	2.688792	down	ENST00000318186	processed_transcript	WDFY3-AS2
RNA175024|ENST00000318186	1.21174E-05	2.67E-07	2.923846	down	ENST00000318186	processed_transcript	WDFY3-AS2
RNA47431|UCSC_6093_1739	1.42063E-05	3.38E-07	3.090735	down	ENST00000318186	processed_transcript	WDFY3-AS2
RNA42917|UCSC_560_4983	1.10125E-06	5.78E-09	6.716901	down	ENST00000342456	processed_transcript	ALDH3B1
RNA47881|UCSC_6648_1616	0.000343631	2.94E-05	2.972755	down	ENST00000399104	processed_transcript	LINC00612
RNA175711|ENST00000400178	0.000175769	1.2E-05	3.173385	down	ENST00000400178	processed_transcript	LINC00478
RNA36999|ENCODE_1869_488	5.04548E-05	2.11E-06	2.811025	down	ENST00000413093	processed_transcript	CYP4B1
RNA158031|XLOC_000264	3.87574E-05	1.43E-06	7.771329	down	ENST00000413519	processed_transcript	RP11-183M13.1
RNA40526|RefSeq_2586_1191	7.54587E-06	1.33E-07	11.76984	down	ENST00000413519	processed_transcript	RP11-183M13.1
RNA177870|ENST00000416105	0.004072893	0.000885	2.510078	down	ENST00000416105	processed_transcript	AL078621.4
RNA36508|ENCODE_1378_586	0.01329124	0.004564	2.291752	down	ENST00000418775	processed_transcript	RP11-348F1.2
RNA175717|ENST00000418813	0.000508092	5.02E-05	2.89768	down	ENST00000418813	processed_transcript	LINC00478
RNA38008|ENCODE_2881_244	4.97101E-08	8.72E-12	13.23769	down	ENST00000419627	processed_transcript	RP3-323P13.2
RNA43360|UCSC_1084_3948	0.000308946	2.54E-05	2.269163	down	ENST00000420823	processed_transcript	AC090044.1
RNA178412|ENST00000420870	2.72206E-05	8.7E-07	2.647752	down	ENST00000420870	processed_transcript	AC093415.2
RNA37216|ENCODE_2086_451	1.04192E-06	5.27E-09	6.552258	down	ENST00000421704	processed_transcript	LINC00472
RNA177625|ENST00000423519	0.000555509	5.66E-05	5.635645	down	ENST00000423519	processed_transcript	AC007277.3
RNA43719|UCSC_1518_3535	5.06385E-05	2.12E-06	4.776401	down	ENST00000426132	processed_transcript	AC064853.4
RNA38842|RefSeq_783_3160	0.010159113	0.003163	3.840307	down	ENST00000426250	processed_transcript	AC087886.1
RNA177257|ENST00000550334	0.001195211	0.000161	5.94414	down	ENST00000426250	processed_transcript	AC087886.1
RNA35234|ENCODE_91_2957	4.61171E-05	1.83E-06	4.046017	down	ENST00000426635	processed_transcript	LINC00472
RNA175712|ENST00000419952	0.000122589	7.45E-06	3.234766	down	ENST00000428669	processed_transcript	LINC00478
RNA48696|UCSC_7642_1367	0.000496095	4.87E-05	2.017051	down	ENST00000428783	processed_transcript	EEF1DP3
RNA37004|ENCODE_1874_487	9.35792E-05	5.19E-06	3.397016	down	ENST00000429601	processed_transcript	RP11-368D24__A.1
RNA37117|ENCODE_1987_470	0.002239564	0.000385	2.219076	down	ENST00000430333	processed_transcript	RP5-1022P6.6
RNA178030|ENST00000432414	0.007307167	0.002	2.019945	down	ENST00000432414	processed_transcript	AC010890.1
RNA51338|UCSC_10846_560	0.00507584	0.001203	2.191225	down	ENST00000432414	processed_transcript	AC010890.1
RNA177270|ENST00000432793	3.29034E-07	5.79E-10	4.765204	down	ENST00000432793	processed_transcript	AC007743.1
RNA176916|ENST00000436211	0.006445197	0.001679	2.056762	down	ENST00000436211	processed_transcript	BX248398.1

RNA178163|ENST00000436602	0.000131279	8.18E-06	2.188014	down	ENST00000436602	processed_transcript	AC087590.3
RNA178162|ENST00000413977	0.000100205	5.72E-06	2.254483	down	ENST00000436602	processed_transcript	AC087590.3
RNA45482|UCSC_3695_2347	0.000489986	4.78E-05	4.80733	down	ENST00000436786	processed_transcript	RP11-399D6.2
RNA48896|UCSC_7884_1306	0.009792601	0.003005	2.1665	down	ENST00000437183	processed_transcript	HLA-DRB6
RNA178649|ENST00000438676	1.1309E-06	6.16E-09	3.891462	down	ENST00000438676	processed_transcript	RP1-234P15.4
RNA178408|ENST00000439074	7.82291E-05	4.01E-06	5.551692	down	ENST00000439074	processed_transcript	RP11-528A4.2
RNA44760|UCSC_2792_2713	0.004423989	0.000993	2.076824	down	ENST00000441044	processed_transcript	MTMR9LP
RNA48573|UCSC_7487_1410	0.004157484	0.00091	2.183403	down	ENST00000441044	processed_transcript	MTMR9LP
RNA39387|RefSeq_1363_2324	0.002998422	0.000579	2.251553	down	ENST00000441044	processed_transcript	MTMR9LP
RNA37084|ENCODE_1954_475	3.75708E-06	4.71E-08	5.445424	down	ENST00000441570	processed_transcript	LINC00472
RNA34425|NRED_557_2026	0.001240534	0.00017	2.29856	down	ENST00000442456	processed_transcript	AC007405.4
RNA34426|NRED_558_2026	0.003974331	0.000856	2.457702	down	ENST00000442456	processed_transcript	AC007405.4
RNA174243|ENST00000442809	2.29301E-05	6.83E-07	3.548525	down	ENST00000442809	processed_transcript	AC090044.1
RNA175165|ENST00000442815	0.003187814	0.000631	2.031337	down	ENST00000442815	processed_transcript	AP001046.5
RNA40205|RefSeq_2240_1546	1.28939E-05	2.91E-07	5.440764	down	ENST00000443779	processed_transcript	RP11-327L3.1
RNA178409|ENST00000445168	0.000132728	8.28E-06	3.613245	down	ENST00000445168	processed_transcript	RP11-528A4.2
RNA163034|XLOC_002973	5.20909E-05	2.21E-06	4.701448	down	ENST00000445168	processed_transcript	RP11-528A4.2
RNA38632|RefSeq_560_3690	0.001205417	0.000163	3.061217	down	ENST00000445174	processed_transcript	LINC00607
RNA176917|ENST00000418974	0.004227486	0.000933	2.195895	down	ENST00000445243	processed_transcript	RP11-495P10.1
RNA38622|RefSeq_550_3708	0.005378152	0.001303	2.046831	down	ENST00000445317	processed_transcript	RP1-163G9.1
RNA45800|UCSC_4109_2222	0.003012652	0.000584	2.057741	down	ENST00000445317	processed_transcript	RP1-163G9.1
RNA175713|ENST00000445461	0.000496321	4.87E-05	2.918767	down	ENST00000445461	processed_transcript	LINC00478
RNA46961|UCSC_5521_1870	2.31158E-06	2.17E-08	6.704081	down	ENST00000446840	processed_transcript	AC003090.1
RNA35681|ENCODE_549_1016	0.045391034	0.02262	2.01697	down	ENST00000447250	processed_transcript	RP4-625H18.2
RNA177271|ENST00000447423	3.76889E-05	1.37E-06	5.733681	down	ENST00000447423	processed_transcript	AC007743.1
RNA175754|ENST00000447950	0.01191944	0.003924	2.067059	down	ENST00000447950	processed_transcript	RP11-75C9.1
RNA162038|XLOC_013457	1.17978E-05	2.57E-07	7.885917	down	ENST00000449270	processed_transcript	RP5-839B4.8
RNA178406|ENST00000449586	0.002783274	0.000522	2.900392	down	ENST00000449586	processed_transcript	AC093415.3
RNA50426|UCSC_9714_885	0.014151452	0.004965	2.366376	down	ENST00000450321	processed_transcript	GPR98
RNA175716|ENST00000453910	0.000605057	6.37E-05	2.575344	down	ENST00000453910	processed_transcript	LINC00478
RNA40251|RefSeq_2290_1495	0.003437971	0.000701	2.420084	down	ENST00000454875	processed_transcript	TTTY14
RNA165944|XLOC_008326	0.005034789	0.001189	2.497799	down	ENST00000454875	processed_transcript	TTTY14
RNA37375|ENCODE_2246_429	1.50159E-05	3.67E-07	8.707478	down	ENST00000454974	processed_transcript	RP11-336K24.6
RNA46445|UCSC_4909_2012	0.001418208	0.000204	3.385702	down	ENST00000458097	processed_transcript	RP11-76N22.2
RNA38794|RefSeq_735_3278	0.001972464	0.000324	3.140063	down	ENST00000458468	processed_transcript	LINC00478
RNA38712|RefSeq_645_3475	0.001565567	0.000235	3.238123	down	ENST00000458468	processed_transcript	LINC00478

RNA162301|XLOC_013869	0.001180578	0.000158	3.345691	down	ENST00000458468	processed_transcript	LINC00478
RNA50782|UCSC_10164_755	0.004890129	0.001142	2.65102	down	ENST00000462876	processed_transcript	CAV2
RNA55221|H-InvDB_2592_237	9.56527E-06	1.91E-07	2.675135	down	ENST00000464796	processed_transcript	TAL1
RNA38920|RefSeq_865_3008	9.99866E-05	5.69E-06	2.428869	down	ENST00000465070	processed_transcript	ZEB2
RNA50645|UCSC_9985_811	0.010409441	0.003268	2.188947	down	ENST00000466872	processed_transcript	C9orf135
RNA49169|UCSC_8194_1231	0.006102374	0.001557	2.620653	down	ENST00000467035	processed_transcript	CAV2
RNA50287|UCSC_9541_931	0.001892004	0.000306	3.644651	down	ENST00000467134	processed_transcript	MUC1
RNA50183|UCSC_9411_958	0.001406171	0.000202	3.705271	down	ENST00000467134	processed_transcript	MUC1
RNA50122|UCSC_9335_977	0.000933261	0.000115	3.730343	down	ENST00000467134	processed_transcript	MUC1
RNA48028|UCSC_6825_1577	0.002747036	0.000512	2.8637	down	ENST00000468377	processed_transcript	RP11-475N22.4
RNA177342|ENST00000468377	0.001219139	0.000166	3.098049	down	ENST00000468377	processed_transcript	RP11-475N22.4
RNA58953|asoverlaps_584_1509	0.015742391	0.005719	2.539321	down	ENST00000469196	processed_transcript	RP11-6F2.5
RNA162936|XLOC_002891	0.005195793	0.001245	2.566409	down	ENST00000469196	processed_transcript	RP11-6F2.5
RNA44678|UCSC_2694_2764	3.61233E-05	1.29E-06	4.548218	down	ENST00000472173	processed_transcript	LONRF3
RNA51967|UCSC_11580_312	0.003251154	0.000649	2.386794	down	ENST00000472848	processed_transcript	CFTR
RNA177410|ENST00000474768	1.27071E-05	2.85E-07	5.135562	down	ENST00000474768	processed_transcript	ADAMTS9-AS2
RNA50451|UCSC_9744_877	9.52955E-06	1.9E-07	3.959367	down	ENST00000479784	processed_transcript	CALCRL
RNA35896|ENCODE_766_818	0.000603658	6.35E-05	2.382704	down	ENST00000480805	processed_transcript	MBL1P
RNA177409|ENST00000481312	5.08464E-05	2.13E-06	3.29448	down	ENST00000481312	processed_transcript	ADAMTS9-AS2
RNA45915|UCSC_4250_2179	1.58068E-05	3.98E-07	3.493808	down	ENST00000481312	processed_transcript	ADAMTS9-AS2
RNA49480|UCSC_8569_1143	3.77604E-05	1.37E-06	3.419189	down	ENST00000482258	processed_transcript	PAPSS2
RNA39319|RefSeq_1291_2410	0.007561036	0.0021	2.121055	down	ENST00000483658	processed_transcript	KALRN
RNA44254|UCSC_2168_3068	0.004183014	0.000919	2.140058	down	ENST00000483658	processed_transcript	KALRN
RNA51483|UCSC_11033_504	5.96246E-07	1.87E-09	5.090725	down	ENST00000484236	processed_transcript	SHROOM3
RNA45862|UCSC_4189_2198	0.000280678	2.24E-05	3.376043	down	ENST00000484327	processed_transcript	C1orf168
RNA44499|UCSC_2471_2871	0.00257992	0.000469	2.741203	down	ENST00000485055	processed_transcript	CCDC39
RNA177412|ENST00000485174	0.000233363	1.75E-05	2.079428	down	ENST00000485174	processed_transcript	ADAMTS9-AS2
RNA47550|UCSC_6252_1706	9.1002E-05	4.99E-06	3.342061	down	ENST00000485493	processed_transcript	ERG
RNA55591|H-InvDB_2974_210	0.000238867	1.8E-05	23.63417	down	ENST00000486566	processed_transcript	SFTPA2
RNA175673|ENST00000486954	0.000588686	6.14E-05	2.074367	down	ENST00000486954	processed_transcript	RP11-511P7.2
RNA51970|UCSC_11583_309	0.00030614	2.51E-05	22.67082	down	ENST00000491208	processed_transcript	SFTPA2
RNA55628|H-InvDB_3014_207	0.000319119	2.66E-05	24.56389	down	ENST00000491208	processed_transcript	SFTPA2
RNA34582|NRED_714_1773	0.000425062	3.95E-05	2.545649	down	ENST00000491442	processed_transcript	HIVEP3
RNA34632|NRED_764_1705	0.000612714	6.48E-05	2.570807	down	ENST00000491442	processed_transcript	HIVEP3
RNA35039|NRED_1180_814	0.000277795	2.21E-05	2.625278	down	ENST00000491442	processed_transcript	HIVEP3
RNA34580|NRED_712_1775	0.000505886	4.99E-05	2.643156	down	ENST00000491442	processed_transcript	HIVEP3

RNA34608|NRED_740_1741	0.000404661	3.7E-05	2.699106	down	ENST00000491442	processed_transcript	HIVEP3
RNA34962|NRED_1103_1014	9.877E-05	5.61E-06	2.984526	down	ENST00000491442	processed_transcript	HIVEP3
RNA50608|UCSC_9938_824	0.000186306	1.29E-05	2.204033	down	ENST00000492808	processed_transcript	CD52
RNA50174|UCSC_9400_961	0.000247975	1.9E-05	2.094451	down	ENST00000496640	processed_transcript	RP11-81N13.1
RNA177439|ENST00000496640	2.76076E-05	8.87E-07	2.618517	down	ENST00000496640	processed_transcript	RP11-81N13.1
RNA176057|ENST00000517335	0.000450785	4.28E-05	3.233739	down	ENST00000499583	processed_transcript	RP11-175K6.1
RNA59275|asoverlaps_1016_224	0.001170497	0.000157	3.562736	down	ENST00000500817	processed_transcript	LIFR-AS1
RNA175046|ENST00000502083	0.000711029	7.97E-05	2.16245	down	ENST00000502083	processed_transcript	RP11-875O11.1
RNA43680|UCSC_1472_3576	0.000108766	6.37E-06	2.508328	down	ENST00000502665	processed_transcript	CYP4V2
RNA175088|ENST00000503066	6.93201E-06	1.17E-07	4.442924	down	ENST00000503066	processed_transcript	RP11-291L15.2
RNA177407|ENST00000503469	0.018786443	0.00722	2.034489	down	ENST00000503469	processed_transcript	CTD-2003C8.2
RNA48687|UCSC_7628_1373	4.61313E-05	1.84E-06	2.227209	down	ENST00000504086	processed_transcript	ITGA1
RNA176292|ENST00000513055	1.63869E-05	4.21E-07	2.704679	down	ENST00000504474	processed_transcript	RP11-65F13.2
RNA39065|RefSeq_1020_2755	4.95849E-05	2.06E-06	2.898771	down	ENST00000504474	processed_transcript	RP11-65F13.2
RNA176295|ENST00000504474	8.60573E-05	4.62E-06	2.919148	down	ENST00000504474	processed_transcript	RP11-65F13.2
RNA176293|ENST00000503134	6.54509E-06	1.07E-07	3.123421	down	ENST00000504474	processed_transcript	RP11-65F13.2
RNA176226|ENST00000504932	4.3079E-05	1.67E-06	2.615247	down	ENST00000504932	processed_transcript	RP11-677M14.3
RNA176133|ENST00000504984	0.003424229	0.000697	2.219489	down	ENST00000504984	processed_transcript	RP11-420A23.1
RNA34445|NRED_577_1999	0.000048985	2.02E-06	2.058215	down	ENST00000505731	processed_transcript	NOP14-AS1
RNA33982|NRED_112_3751	5.99552E-05	2.74E-06	3.266338	down	ENST00000506258	processed_transcript	FAM105A
RNA176285|ENST00000506665	2.32757E-05	6.99E-07	2.71684	down	ENST00000506665	processed_transcript	CTC-454M9.1
RNA40958|RefSeq_3056_748	9.47794E-05	5.29E-06	2.588793	down	ENST00000506741	processed_transcript	RP11-701P16.1
RNA176114|ENST00000508111	0.009894363	0.003051	2.36013	down	ENST00000508111	processed_transcript	RP11-93L9.1
RNA34938|NRED_1076_1076	3.41832E-05	1.19E-06	5.602822	down	ENST00000508269	processed_transcript	RP11-291L15.2
RNA34915|NRED_1049_1145	4.75267E-06	6.66E-08	6.601449	down	ENST00000508269	processed_transcript	RP11-291L15.2
RNA175087|ENST00000508269	1.5744E-05	3.96E-07	6.664025	down	ENST00000508269	processed_transcript	RP11-291L15.2
RNA34919|NRED_1053_1126	9.92289E-06	2E-07	6.680639	down	ENST00000508269	processed_transcript	RP11-291L15.2
RNA48455|UCSC_7342_1444	1.54572E-05	3.83E-07	2.544689	down	ENST00000508462	processed_transcript	RP4-671G15.3
RNA176280|ENST00000509179	0.000287886	2.32E-05	2.238859	down	ENST00000508521	processed_transcript	CTC-454M9.1
RNA176286|ENST00000508521	6.68537E-05	3.2E-06	2.469768	down	ENST00000508521	processed_transcript	CTC-454M9.1
RNA176283|ENST00000508718	0.001474529	0.000216	2.819153	down	ENST00000508718	processed_transcript	CTC-454M9.1
RNA175675|ENST00000510284	0.000341768	2.92E-05	2.280859	down	ENST00000510284	processed_transcript	RP11-701P16.5
RNA175674|ENST00000511703	0.000146131	9.4E-06	2.327883	down	ENST00000510284	processed_transcript	RP11-701P16.5
RNA51141|UCSC_10595_632	6.76547E-05	3.26E-06	2.345464	down	ENST00000510284	processed_transcript	RP11-701P16.5
RNA164339|XLOC_005755	1.51958E-05	3.75E-07	8.432889	down	ENST00000511849	processed_transcript	RP1-240B8.3
RNA176282|ENST00000511876	0.00016314	1.08E-05	2.059131	down	ENST00000511876	processed_transcript	CTC-454M9.1

RNA175086|ENST00000512359	6.33013E-05	2.96E-06	8.335382	down	ENST00000512359	processed_transcript	RP11-291L15.2
RNA175815|ENST00000512519	0.001562782	0.000235	2.427144	down	ENST00000512519	processed_transcript	CTD-2127H9.1
RNA58127|UCRs_442_204	4.30447E-05	1.67E-06	2.350891	down	ENST00000513055	processed_transcript	RP11-65F13.2
RNA176134|ENST00000513851	0.001128432	0.000149	2.663914	down	ENST00000513851	processed_transcript	RP11-420A23.1
RNA176284|ENST00000514158	0.000584795	6.08E-05	2.109241	down	ENST00000514158	processed_transcript	CTC-454M9.1
RNA176287|ENST00000514571	0.020034702	0.00787	2.0459	down	ENST00000514571	processed_transcript	CTC-454M9.1
RNA46562|UCSC_5057_1975	0.000175834	1.2E-05	2.064952	down	ENST00000515743	processed_transcript	WDR1
RNA175009|ENST00000521775	0.007523749	0.002086	2.336896	down	ENST00000517798	processed_transcript	CTD-2547L16.1
RNA175975|ENST00000519898	0.009816059	0.003016	2.102645	down	ENST00000519898	processed_transcript	MIR143HG
RNA176082|ENST00000520067	0.045720913	0.022832	2.024723	down	ENST00000520067	processed_transcript	CTC-308K20.1
RNA40160|RefSeq_2189_1594	0.006320986	0.001637	2.048498	down	ENST00000520067	processed_transcript	CTC-308K20.1
RNA40759|RefSeq_2840_967	0.011265836	0.003637	2.085108	down	ENST00000520067	processed_transcript	CTC-308K20.1
RNA50807|UCSC_10193_747	5.47122E-06	8.21E-08	3.09271	down	ENST00000527474	processed_transcript	RP11-820L6.1
RNA40416|RefSeq_2471_1330	0.001819042	0.00029	3.091913	down	ENST00000527780	processed_transcript	NAPSB
RNA175118|ENST00000527780	0.000930929	0.000115	3.393462	down	ENST00000527780	processed_transcript	NAPSB
RNA39235|RefSeq_1202_2503	0.010630143	0.003365	2.955566	down	ENST00000527847	processed_transcript	RP11-494M8.4
RNA177398|ENST00000530344	0.000557755	5.7E-05	2.049341	down	ENST00000530344	processed_transcript	CTD-2003C8.1
RNA178035|ENST00000532353	0.000428182	3.99E-05	2.220868	down	ENST00000532353	processed_transcript	TRAF3IP2-AS1
RNA176227|ENST00000532579	0.000120649	7.3E-06	4.620166	down	ENST00000532579	processed_transcript	RP11-677M14.3
RNA47856|UCSC_6618_1623	0.001481275	0.000218	2.360225	down	ENST00000539983	processed_transcript	PZP
RNA147089|nc-HOXC11-109	8.48228E-05	4.51E-06	4.71829	down	ENST00000548657	processed_transcript	KRT7
RNA45611|UCSC_3870_2294	1.91398E-05	5.29E-07	3.659007	down	ENST00000556961	processed_transcript	FBLN5
RNA50160|UCSC_9383_966	6.73443E-05	3.24E-06	2.269258	down	ENST00000560198	processed_transcript	RP11-1008C21.2
RNA176334|ENST00000434223	2.24595E-05	6.63E-07	2.290599	down	ENST00000560198	processed_transcript	RP11-1008C21.2
RNA63736|RNAz_4456_220	0.002730323	0.000508	2.551402	down	ENST00000561399	processed_transcript	PKNOX2
RNA39649|RefSeq_1646_2073	1.1309E-06	6.17E-09	9.53017	down	ENST00000563852	processed_transcript	RP11-506G7.1
RNA50124|UCSC_9337_977	1.46278E-05	3.53E-07	4.779593	down	ENST00000564564	processed_transcript	MT1JP
RNA51341|UCSC_10849_559	0.000278058	2.21E-05	5.990304	down	ENST00000564974	processed_transcript	MT1X
RNA41226|RefSeq_3355_426	3.4233E-05	1.19E-06	3.263114	down	ENST00000565768	processed_transcript	MT1L
RNA35347|ENCODE_208_2228	5.21901E-06	7.74E-08	3.959904	down	ENST00000579235	processed_transcript	AC058791.1
RNA34041|NRED_172_3224	7.71184E-05	3.94E-06	2.45454	down	ENST00000580522	processed_transcript	AC058791.2
RNA34454|NRED_586_1992	9.99866E-05	5.7E-06	2.493891	down	ENST00000580522	processed_transcript	AC058791.2
RNA34868|NRED_1002_1262	9.20712E-05	5.07E-06	2.532646	down	ENST00000580522	processed_transcript	AC058791.2
RNA178570|ENST00000443623	9.35792E-05	5.19E-06	2.583301	down	ENST00000580522	processed_transcript	AC058791.2
RNA178563|ENST00000451786	4.80521E-05	1.95E-06	2.604409	down	ENST00000580522	processed_transcript	AC058791.2
RNA34299|NRED_430_2282	5.78482E-05	2.59E-06	2.635221	down	ENST00000580522	processed_transcript	AC058791.2

RNA34316|NRED_447_2251	4.89432E-05	2.01E-06	2.650695	down	ENST00000580522	processed_transcript	AC058791.2
RNA178561|ENST00000447307	3.00807E-06	3.24E-08	4.267532	down	ENST00000580851	processed_transcript	AC058791.1
RNA178562|ENST00000444003	2.08726E-06	1.82E-08	4.697102	down	ENST00000582369	processed_transcript	AC058791.1
RNA47872|UCSC_6636_1618	2.50213E-05	7.76E-07	2.869444	down	ENST00000584325	processed_transcript	AC016831.7
RNA178558|ENST00000447430	2.22796E-05	6.55E-07	2.976191	down	ENST00000584325	processed_transcript	AC016831.7
RNA176977|ENST00000445243	0.005128822	0.001222	2.04892	down	ENST00000584786	processed_transcript	RP11-495P10.1
RNA40762|RefSeq_2843_962	0.005704807	0.001413	2.142902	down	ENST00000416624	intronic	CD69
RNA47512|UCSC_6191_1720	0.007692058	0.002151	2.334616	down	ENST00000427517	intronic	RP3-527F8.2
RNA165882|XLOC_008077	0.004616546	0.001053	2.479816	down	ENST00000427517	intronic	RP3-527F8.2
RNA43345|UCSC_1064_3978	5.12811E-06	7.57E-08	3.391689	down	ENST00000452320	intronic	MAGI2-AS3
RNA177411|ENST00000460833	9.71261E-05	5.46E-06	5.502624	down	ENST00000460833	intronic	ADAMTS9-AS2
RNA51069|UCSC_10501_658	0.002090056	0.000351	2.796665	down	ENST00000461836	intronic	DPP4
RNA162829|XLOC_003223	5.56123E-05	2.44E-06	5.555232	down	ENST00000462528	intronic	RP11-768G7.2
RNA62213|RNAz_2931_240	0.003878942	0.000829	2.919995	down	ENST00000470802	intronic	ROBO2
RNA34252|NRED_383_2389	0.001204827	0.000163	2.369684	down	ENST00000485668	intronic	BTBD19
RNA34416|NRED_548_2063	2.51709E-05	7.83E-07	3.340239	down	ENST00000492600	intronic	RCAN1
RNA58167|CombinedLit_3_13251	1.2841E-05	2.89E-07	4.849215	down	ENST00000505589	intronic	NEDD9
RNA53590|H-InvDB_862_482	3.10081E-05	1.04E-06	2.132528	down	ENST00000506361	intronic	MYLK
RNA176294|ENST00000507963	2.00391E-05	5.62E-07	2.838268	down	ENST00000507963	intronic	RP11-65F13.2
RNA48512|UCSC_7413_1427	0.000138895	8.8E-06	2.67872	down	ENST00000524231	intronic	ABCA10
RNA34660|NRED_792_1666	0.002784366	0.000523	2.048885	down	ENST00000524877	intronic	BEST1
RNA175117|ENST00000531692	0.001508999	0.000224	2.896172	down	ENST00000531692	intronic	NAPSB
RNA60608|RNAz_1321_285	0.00080101	9.34E-05	2.453159	down	ENST00000531862	intronic	PHLDB1
RNA48683|UCSC_7624_1374	0.00280204	0.000527	2.821242	down	ENST00000547946	intronic	RMST
RNA58202|CombinedLit_70_1881	0.001065863	0.000138	3.096547	down	ENST00000547946	intronic	RMST
RNA177556|ENST00000547946	0.00169775	0.000263	3.208784	down	ENST00000547946	intronic	RMST
RNA177551|ENST00000538559	0.001309844	0.000183	3.297905	down	ENST00000547946	intronic	RMST
RNA177554|ENST00000547996	0.001415885	0.000204	3.894736	down	ENST00000547996	intronic	RMST
RNA177539|ENST00000549723	0.016191445	0.005942	2.213262	down	ENST00000549723	intronic	RP11-887P2.1
RNA43186|UCSC_876_4275	4.89432E-05	2.01E-06	3.428527	down	ENST00000555379	intronic	CTD-2552B11.4
RNA39777|RefSeq_1781_1956	0.001226851	0.000168	2.122834	down	ENST00000556438	intronic	LGALS3
RNA178560|ENST00000418546	7.42989E-07	3E-09	4.451917	down	ENST00000577311	intronic	AC058791.1
RNA34782|NRED_915_1440	0.000753812	8.61E-05	2.483693	down	ENST00000584798	intronic	EIF4A1
RNA162828|XLOC_003222	6.00832E-06	9.46E-08	8.284711	down	ENST00000493545	intronic	RP11-768G7.2
RNA51253|UCSC_10738_593	0.000475955	4.6E-05	2.830173	down	ENST00000515882	intronic	SLIT2-IT1
RNA34739|NRED_871_1541	6.09367E-05	2.8E-06	4.645016	down	ENST00000560449	intronic	RASGRF1

RNA160356|XLOC_011585	8.06616E-05	4.19E-06	4.531222	down	ENST00000565107	intronic	RP11-386M24.6
RNA158751|XLOC_008895	0.02818816	0.012296	2.000532	down			
RNA159263|XLOC_009659	0.014019098	0.004905	2.004726	down			
RNA160407|XLOC_011620	0.001855461	0.000298	2.006347	down			
RNA163365|XLOC_003686	0.045051575	0.022418	2.010583	down			
RNA43817|UCSC_1630_3449	0.000312384	2.58E-05	2.010649	down			
RNA45357|UCSC_3545_2393	3.0129E-05	9.94E-07	2.013083	down			
RNA164349|XLOC_005763	0.00823403	0.002366	2.020385	down			
RNA159952|XLOC_010798	0.012042946	0.003982	2.022484	down			
RNA45632|UCSC_3895_2286	0.000194045	1.36E-05	2.023773	down			
RNA45421|UCSC_3622_2372	0.001575157	0.000238	2.026742	down			
RNA34559|NRED_691_1807	0.000259065	2.01E-05	2.027368	down			
RNA34251|NRED_382_2401	0.00169341	0.000262	2.031465	down			
RNA63777|RNAz_4497_219	0.000093166	5.15E-06	2.031644	down			
RNA55344|H-InvDB_2720_223	0.000368831	3.24E-05	2.035382	down			
RNA64880|RNAz_5601_200	0.000273456	2.16E-05	2.036001	down			
RNA60498|RNAz_1210_296	0.022359397	0.009098	2.036146	down			
RNA162633|XLOC_002585	0.007867183	0.002224	2.037124	down			
RNA164899|XLOC_006588	0.000402442	3.67E-05	2.039434	down			
RNA63567|RNAz_4287_224	0.000914234	0.000112	2.039631	down			
RNA38336|RefSeq_250_4981	0.003475409	0.000712	2.043531	down			
RNA57728|UCRs_43_339	0.003222364	0.000641	2.045139	down			
RNA160203|XLOC_011430	0.007648327	0.002132	2.04647	down			
RNA46025|UCSC_4384_2138	0.000268751	2.11E-05	2.047862	down			
RNA163548|XLOC_003810	0.032309998	0.014679	2.052244	down			
RNA34738|NRED_870_1542	6.58428E-05	3.13E-06	2.052497	down			
RNA64443|RNAz_5164_200	0.006875952	0.001836	2.052998	down			
RNA38421|RefSeq_337_4427	7.03245E-05	3.44E-06	2.056092	down			
RNA64634|RNAz_5355_200	0.024635853	0.010335	2.056245	down			
RNA165478|XLOC_007725	0.00411586	0.000898	2.059804	down			
RNA162659|XLOC_003079	0.002686715	0.000497	2.060887	down			
RNA63917|RNAz_4637_216	0.004670092	0.001071	2.061474	down			
RNA38276|RefSeq_185_5484	0.000720617	8.12E-05	2.064447	down			
RNA64870|RNAz_5591_200	0.022045432	0.008931	2.065177	down			
RNA159675|XLOC_010574	8.88721E-05	4.83E-06	2.066228	down			
RNA159329|XLOC_009734	0.015763097	0.005729	2.06966	down			

RNA164279|XLOC_005275	2.17206E-05	6.28E-07	2.071235	down				
RNA42987|UCSC_642_4747	0.00024634	1.88E-05	2.072831	down				
RNA96251|EvoFold_62_304	0.0002022	1.44E-05	2.073378	down				
RNA33985|NRED_116_3665	0.000981383	0.000123	2.077247	down				
RNA63008|RNAz_3726_233	0.008364386	0.002419	2.078525	down				
RNA59341|RNAz_52_507	0.005731555	0.001422	2.080456	down				
RNA160593|XLOC_011803	0.00226358	0.000391	2.081132	down				
RNA62542|RNAz_3260_238	0.001483903	0.000218	2.081934	down				
RNA49210|UCSC_8243_1219	5.87996E-05	2.66E-06	2.083363	down				
RNA63356|RNAz_4074_228	7.59306E-06	1.34E-07	2.084663	down				
RNA64835|RNAz_5556_200	0.011123343	0.003576	2.085724	down				
RNA161369|XLOC_002005	0.028551918	0.012514	2.085976	down				
RNA62543|RNAz_3261_238	0.004466623	0.001006	2.088732	down				
RNA160158|XLOC_011401	0.013829446	0.004815	2.090378	down				
RNA43263|UCSC_968_4134	0.000475114	4.59E-05	2.091359	down				
RNA45594|UCSC_3850_2301	0.001181274	0.000159	2.094655	down				
RNA158365|XLOC_001195	0.022002013	0.008907	2.095225	down				
RNA47146|UCSC_5747_1819	4.07337E-05	1.54E-06	2.097977	down				
RNA64897|RNAz_5618_200	0.007026824	0.001895	2.099601	down				
RNA165234|XLOC_006870	0.014618325	0.005183	2.100886	down				
RNA62111|RNAz_2827_240	0.01317998	0.004508	2.102425	down				
RNA58049|UCRs_364_215	0.00011786	7.08E-06	2.103415	down				
RNA96492|EvoFold_319_239	0.00016406	1.09E-05	2.107037	down				
RNA160539|XLOC_011979	0.001763467	0.000277	2.107517	down				
RNA160534|XLOC_011753	0.006256346	0.001612	2.114047	down				
RNA34213|NRED_344_2518	0.003619703	0.000752	2.115757	down				
RNA60172|RNAz_884_315	6.81143E-06	1.13E-07	2.117027	down				
RNA38257|RefSeq_165_5771	0.000324815	2.72E-05	2.118193	down				
RNA40116|RefSeq_2144_1632	0.002978158	0.000574	2.119798	down				
RNA43323|UCSC_1034_4026	0.001623925	0.000247	2.120955	down				
RNA47424|UCSC_6082_1741	0.001636945	0.00025	2.125799	down				
RNA58808|asoverlaps_393_1943	5.46937E-05	2.39E-06	2.127166	down				
RNA63177|RNAz_3895_231	0.001070983	0.000139	2.127421	down				
RNA49025|UCSC_8028_1273	0.000197954	1.4E-05	2.129874	down				
RNA63987|RNAz_4707_214	0.015765859	0.005731	2.129982	down				
RNA162778|XLOC_002726	0.001483859	0.000218	2.13412	down				

RNA147455|p0559_imsncRNA217	0.000763922	8.78E-05	2.135282	down				
RNA147681|p0785_imsncRNA572	0.005344647	0.001293	2.137997	down				
RNA161895|XLOC_002461	0.007555964	0.002098	2.138131	down				
RNA46255|UCSC_4669_2069	0.008051742	0.002294	2.138965	down				
RNA45695|UCSC_3974_2264	0.009816059	0.003017	2.14208	down				
RNA34520|NRED_652_1868	8.95884E-05	4.88E-06	2.147158	down				
RNA163554|XLOC_003821	0.00243491	0.000433	2.148066	down				
RNA60249|RNAz_961_312	0.000258728	2.01E-05	2.15234	down				
RNA164976|XLOC_006299	0.001674933	0.000258	2.152919	down				
RNA58032|UCRs_347_218	0.00023079	1.72E-05	2.15693	down				
RNA49051|UCSC_8057_1266	0.000241825	1.83E-05	2.156945	down				
RNA63813|RNAz_4533_218	0.000475955	4.6E-05	2.160359	down				
RNA176787|ENST00000503960	1.63112E-05	4.17E-07	2.160944	down				
RNA147105|p0209_imsncRNA319	0.000188471	1.31E-05	2.162549	down				
RNA45231|UCSC_3376_2456	0.000889965	0.000108	2.164352	down				
RNA64726|RNAz_5447_200	0.002323603	0.000405	2.166205	down				
RNA96641|EvoFold_482_214	4.12882E-05	1.57E-06	2.166709	down				
RNA34519|NRED_651_1878	0.000162172	1.07E-05	2.168014	down				
RNA64161|RNAz_4881_208	0.000206723	1.49E-05	2.172888	down				
RNA59766|RNAz_478_356	0.00161967	0.000246	2.174082	down				
RNA164445|XLOC_005449	0.000999555	0.000126	2.175946	down				
RNA96364|EvoFold_183_267	0.002327083	0.000406	2.181135	down				
RNA96337|EvoFold_155_273	5.29721E-05	2.27E-06	2.182961	down				
RNA165121|XLOC_006781	0.012499425	0.004187	2.183693	down				
RNA34348|NRED_479_2184	0.00376155	0.000793	2.186822	down				
RNA57735|UCRs_50_335	0.0007553	8.63E-05	2.187733	down				
RNA61265|RNAz_1979_271	0.000312095	2.58E-05	2.190987	down				
RNA165496|XLOC_007412	0.000710771	7.96E-05	2.194411	down				
RNA64801|RNAz_5522_200	0.029135965	0.01286	2.20126	down				
RNA48609|UCSC_7527_1401	0.000113256	6.73E-06	2.204859	down				
RNA34855|NRED_989_1284	0.003866984	0.000825	2.205499	down				
RNA96732|EvoFold_583_201	0.0102603	0.003203	2.205799	down				
RNA146925|p0029_imsncRNA74	0.000733693	8.32E-05	2.20719	down				
RNA61970|RNAz_2685_241	0.004758018	0.001099	2.208108	down				
RNA96301|EvoFold_115_283	0.003255952	0.000651	2.210676	down				
RNA96465|EvoFold_290_244	0.0320962	0.014547	2.213968	down				

RNA61730|RNAz_2444_254	0.008494933	0.002473	2.215716	down			
RNA165061|XLOC_006726	0.005500861	0.001343	2.216296	down			
RNA46197|UCSC_4590_2087	0.011471229	0.003725	2.217199	down			
RNA61162|RNAz_1876_274	0.011228554	0.00362	2.217862	down			
RNA158984|XLOC_009415	0.008171338	0.002341	2.220624	down			
RNA160646|XLOC_012065	0.003417667	0.000695	2.222061	down			
RNA62405|RNAz_3123_239	0.029066691	0.012821	2.224485	down			
RNA58036|UCRs_351_218	0.0452884	0.022556	2.224906	down			
RNA63851|RNAz_4571_217	0.00769311	0.002152	2.226504	down			
RNA60017|RNAz_729_320	2.1962E-05	6.41E-07	2.226728	down			
RNA162682|XLOC_003102	0.002363189	0.000414	2.228264	down			
RNA63791|RNAz_4511_219	0.000281916	2.26E-05	2.232264	down			
RNA60106|RNAz_818_318	4.23293E-05	1.63E-06	2.232994	down			
RNA61791|RNAz_2505_251	0.004922178	0.001152	2.233626	down			
RNA63123|RNAz_3841_232	0.000323199	2.7E-05	2.242118	down			
RNA160844|XLOC_012308	0.001344426	0.00019	2.243506	down			
RNA162292|XLOC_014002	0.013748312	0.004779	2.245558	down			
RNA44176|UCSC_2073_3135	0.002046113	0.000341	2.246718	down			
RNA34595|NRED_727_1750	0.000158693	1.04E-05	2.251157	down			
RNA62775|RNAz_3493_236	0.003778229	0.000799	2.253705	down			
RNA163024|XLOC_002967	0.028904755	0.012726	2.262946	down			
RNA51170|UCSC_10637_621	0.004616483	0.001053	2.264512	down			
RNA147089|p0193_imsncRNA292	0.006563216	0.001722	2.264679	down			
RNA146930|p0034_imsncRNA77	0.002386316	0.000421	2.270196	down			
RNA38261|RefSeq_169_5712	6.47501E-05	3.05E-06	2.272754	down			
RNA165319|XLOC_006928	0.002877388	0.000547	2.274168	down			
RNA51882|UCSC_11482_347	0.004028681	0.000872	2.275173	down			
RNA58156|UCRs_471_201	0.000967112	0.000121	2.277076	down			
RNA57869|UCRs_184_257	0.000861553	0.000103	2.277932	down			
RNA59762|RNAz_474_356	0.003007296	0.000582	2.278246	down			
RNA162645|XLOC_002597	0.009409932	0.002849	2.280791	down			
RNA61767|RNAz_2481_253	0.004204694	0.000925	2.2814	down			
RNA46305|UCSC_4731_2053	0.004816049	0.001118	2.284562	down			
RNA160810|XLOC_012269	0.005221617	0.001253	2.292392	down			
RNA62925|RNAz_3643_234	7.96707E-05	4.12E-06	2.297194	down			
RNA162015|XLOC_013623	0.00020523	1.47E-05	2.306816	down			

RNA43105|UCSC_786_4462	1.04826E-05	2.18E-07	2.307101	down				
RNA165778|XLOC_007958	0.001550681	0.000232	2.307168	down				
RNA62692|RNAz_3410_237	0.000274185	2.17E-05	2.307277	down				
RNA34725|NRED_857_1572	1.0414E-05	2.15E-07	2.307366	down				
RNA147360|p0464_imsncRNA7	0.001779588	0.000281	2.307986	down				
RNA96650|EvoFold_492_212	0.000268139	2.1E-05	2.313316	down				
RNA165056|XLOC_007012	0.048207175	0.02438	2.314788	down				
RNA59822|RNAz_534_350	0.003545731	0.000731	2.319653	down				
RNA64715|RNAz_5436_200	7.44033E-05	3.73E-06	2.326519	down				
RNA58565|asoverlaps_80_3270	3.71358E-05	1.34E-06	2.33086	down				
RNA147574|p0678_imsncRNA439	0.00781828	0.002203	2.336913	down				
RNA59697|RNAz_409_360	5.58579E-05	2.46E-06	2.338501	down				
RNA158342|XLOC_001181	1.03077E-05	2.11E-07	2.34507	down				
RNA63309|RNAz_4027_229	0.001964329	0.000322	2.346345	down				
RNA161775|XLOC_002344	0.003006904	0.000582	2.348637	down				
RNA58520|asoverlaps_29_4142	0.001956317	0.00032	2.348916	down				
RNA50893|UCSC_10290_721	0.002663947	0.000491	2.349572	down				
RNA60843|RNAz_1557_279	0.000707192	7.9E-05	2.350457	down				
RNA59409|RNAz_120_439	0.001425605	0.000206	2.352929	down				
RNA63909|RNAz_4629_216	0.008838616	0.002611	2.355622	down				
RNA34834|NRED_968_1341	0.001444365	0.00021	2.358164	down				
RNA159182|XLOC_009307	0.001700543	0.000264	2.359616	down				
RNA158204|XLOC_001035	0.001840735	0.000294	2.365821	down				
RNA60008|RNAz_720_320	0.000161664	1.07E-05	2.373762	down				
RNA40648|RefSeq_2723_1072	0.000227009	1.68E-05	2.379097	down				
RNA39212|RefSeq_1177_2535	0.000297094	2.41E-05	2.382995	down				
RNA61709|RNAz_2423_255	3.25873E-05	1.11E-06	2.385566	down				
RNA63203|RNAz_3921_231	0.004996555	0.001177	2.392326	down				
RNA45585|UCSC_3836_2305	1.90947E-05	5.25E-07	2.394846	down				
RNA159181|XLOC_009583	0.005627979	0.001387	2.39771	down				
RNA48345|UCSC_7213_1481	2.0888E-06	1.83E-08	2.399308	down				
RNA165425|XLOC_007342	0.018994905	0.007329	2.407502	down				
RNA146946|p0050_imsncRNA100	0.001917623	0.000312	2.409841	down				
RNA64118|RNAz_4838_210	0.009261184	0.002784	2.414727	down				
RNA50463|UCSC_9759_873	0.000707181	7.9E-05	2.416478	down				
RNA45666|UCSC_3937_2278	0.006029873	0.00153	2.416564	down				

RNA53275|H-InvDB_539_615	0.002616479	0.000479	2.416827	down			
RNA48289|UCSC_7148_1499	2.34722E-05	7.09E-07	2.42183	down			
RNA158570|XLOC_008730	0.002175923	0.00037	2.422937	down			
RNA47383|UCSC_6027_1755	0.01962763	0.007664	2.429764	down			
RNA46297|UCSC_4721_2054	2.13534E-05	6.12E-07	2.433469	down			
RNA55136|H-InvDB_2506_248	0.00023666	1.78E-05	2.440964	down			
RNA34769|NRED_902_1463	0.000558368	5.71E-05	2.442569	down			
RNA34059|NRED_190_3099	0.004843124	0.001126	2.461163	down			
RNA147249|p0353_imsncRNA771	0.000238221	1.79E-05	2.467282	down			
RNA64143|RNAz_4863_209	4.25121E-06	5.61E-08	2.471043	down			
RNA160614|XLOC_011812	0.000579	6E-05	2.472103	down			
RNA34636|NRED_768_1699	0.000106772	6.22E-06	2.472452	down			
RNA34655|NRED_787_1672	0.001686146	0.00026	2.474026	down			
RNA60616|RNAz_1329_284	5.12851E-05	2.16E-06	2.476535	down			
RNA49791|UCSC_8941_1062	0.001326509	0.000186	2.478795	down			
RNA63935|RNAz_4655_216	8.01174E-05	4.15E-06	2.488662	down			
RNA162724|XLOC_003148	0.005830551	0.001459	2.489847	down			
RNA165814|XLOC_008001	3.27828E-06	3.74E-08	2.493107	down			
RNA62559|RNAz_3277_238	0.004953733	0.001162	2.494011	down			
RNA46746|UCSC_5273_1930	0.017266454	0.006466	2.495215	down			
RNA48014|UCSC_6805_1582	0.04926336	0.025067	2.498929	down			
RNA161985|XLOC_002528	0.006923423	0.001856	2.508773	down			
RNA44435|UCSC_2394_2919	3.20783E-05	1.09E-06	2.510213	down			
RNA63714|RNAz_4434_221	2.36705E-05	7.18E-07	2.510658	down			
RNA162534|XLOC_014361	0.000135101	8.47E-06	2.51149	down			
RNA64234|RNAz_4955_206	0.000467437	4.49E-05	2.512655	down			
RNA48315|UCSC_7180_1489	0.003237441	0.000645	2.522866	down			
RNA34100|NRED_231_2922	0.003047703	0.000593	2.524696	down			
RNA96216|EvoFold_26_347	0.000183493	1.27E-05	2.527646	down			
RNA60945|RNAz_1659_277	0.000184326	1.27E-05	2.534277	down			
RNA55243|H-InvDB_2614_234	0.002048624	0.000341	2.535009	down			
RNA54245|H-InvDB_1548_350	0.008130978	0.002326	2.545357	down			
RNA53572|H-InvDB_844_489	0.00074405	8.47E-05	2.545951	down			
RNA45412|UCSC_3611_2375	0.003797192	0.000805	2.555112	down			
RNA60519|RNAz_1231_294	4.09328E-06	5.3E-08	2.55801	down			
RNA158936|XLOC_009374	0.012799951	0.004325	2.562814	down			

RNA158909|XLOC_009026	0.020774398	0.008261	2.568815	down				
RNA164317|XLOC_005311	0.012625389	0.004244	2.580778	down				
RNA47490|UCSC_6164_1726	0.003773308	0.000797	2.589639	down				
RNA59612|RNAz_323_381	9.7219E-05	5.48E-06	2.591505	down				
RNA164213|XLOC_005634	0.011216444	0.003616	2.592639	down				
RNA44413|UCSC_2363_2936	3.91654E-06	4.98E-08	2.594096	down				
RNA147246|p0350_imsncRNA767	0.002201515	0.000376	2.597875	down				
RNA39309|RefSeq_1280_2420	0.002400224	0.000424	2.603504	down				
RNA165612|XLOC_007827	0.000285398	2.29E-05	2.604238	down				
RNA34622|NRED_754_1722	7.23518E-05	3.59E-06	2.605384	down				
RNA61370|RNAz_2084_269	0.000941917	0.000117	2.608277	down				
RNA162090|XLOC_013688	0.000263594	2.06E-05	2.612751	down				
RNA61808|RNAz_2522_250	0.000333291	2.82E-05	2.619215	down				
RNA64013|RNAz_4733_213	0.001459056	0.000213	2.623167	down				
RNA160157|XLOC_011171	0.002617608	0.000479	2.623769	down				
RNA58577|asoverlaps_92_3152	2.36662E-05	7.16E-07	2.628923	down				
RNA161437|XLOC_001443	0.000510215	5.06E-05	2.629387	down				
RNA45795|UCSC_4102_2222	8.25884E-05	4.34E-06	2.630557	down				
RNA47687|UCSC_6417_1668	0.009607191	0.00293	2.632407	down				
RNA96369|EvoFold_189_266	0.001337854	0.000188	2.636795	down				
RNA158775|XLOC_008568	0.004274936	0.000949	2.651574	down				
RNA61781|RNAz_2495_252	0.001145182	0.000152	2.651705	down				
RNA159676|XLOC_010575	0.00030073	2.45E-05	2.658637	down				
RNA158571|XLOC_008731	0.005241688	0.00126	2.665824	down				
RNA57974|UCRs_289_231	0.002378487	0.000419	2.667673	down				
RNA44848|UCSC_2902_2659	0.002322072	0.000405	2.674635	down				
RNA158553|XLOC_008379	0.000351966	3.04E-05	2.675552	down				
RNA61937|RNAz_2652_243	4.544E-05	1.79E-06	2.683833	down				
RNA159871|XLOC_010490	0.01950276	0.007598	2.694411	down				
RNA147280|p0384_imsncRNA801	0.003921143	0.000841	2.698317	down				
RNA161090|XLOC_012931	0.002897906	0.000553	2.710316	down				
RNA164654|XLOC_006349	0.000202476	1.45E-05	2.724403	down				
RNA158959|XLOC_009087	0.017839642	0.006757	2.73	down				
RNA45944|UCSC_4280_2168	0.002308172	0.000402	2.733903	down				
RNA160809|XLOC_012542	0.001694977	0.000262	2.734079	down				
RNA59290|RNAz_1_1128	0.000102118	5.87E-06	2.738297	down				

RNA43299|UCSC_1007_4076	5.55933E-06	8.48E-08	2.741727	down			
RNA48674|UCSC_7612_1379	0.000277702	2.21E-05	2.74665	down			
RNA44663|UCSC_2676_2772	0.001169435	0.000156	2.749825	down			
RNA147235|p0339_imsncRNA761	2.64752E-06	2.69E-08	2.754719	down			
RNA160267|XLOC_011287	7.61811E-05	3.86E-06	2.760482	down			
RNA60563|RNAz_1275_289	0.003097873	0.000606	2.76212	down			
RNA164614|XLOC_005563	0.000272217	2.15E-05	2.76291	down			
RNA34522|NRED_654_1866	0.000118514	7.13E-06	2.775435	down			
RNA40341|RefSeq_2387_1396	8.46206E-05	4.49E-06	2.77973	down			
RNA164113|XLOC_004685	0.003553221	0.000733	2.78341	down			
RNA96234|EvoFold_44_321	0.000495103	4.85E-05	2.80098	down			
RNA34486|NRED_618_1924	0.002537213	0.000458	2.805801	down			
RNA46358|UCSC_4795_2039	0.000171766	1.16E-05	2.806091	down			
RNA62324|RNAz_3042_239	6.11005E-05	2.82E-06	2.806705	down			
RNA63336|RNAz_4054_229	5.40963E-07	1.46E-09	2.823078	down			
RNA40663|RefSeq_2738_1064	3.57442E-05	1.27E-06	2.845755	down			
RNA63570|RNAz_4290_224	0.000393967	3.55E-05	2.846076	down			
RNA34195|NRED_326_2560	1.5932E-05	4.03E-07	2.846993	down			
RNA160936|XLOC_012786	0.000390993	3.52E-05	2.854186	down			
RNA50346|UCSC_9615_913	0.001195479	0.000161	2.857525	down			
RNA147457|p0561_imsncRNA222	0.000133442	8.34E-06	2.858227	down			
RNA63514|RNAz_4233_225	0.00098262	0.000123	2.863084	down			
RNA160430|XLOC_011880	0.005004723	0.00118	2.872577	down			
RNA64932|RNAz_5653_200	0.000247573	1.89E-05	2.886505	down			
RNA39460|RefSeq_1442_2253	0.000394458	3.56E-05	2.889221	down			
RNA165145|XLOC_007087	4.63612E-05	1.85E-06	2.905911	down			
RNA60828|RNAz_1542_279	0.013039995	0.004438	2.914274	down			
RNA59895|RNAz_607_340	0.000223481	1.65E-05	2.921336	down			
RNA46623|UCSC_5129_1961	0.000349515	3.01E-05	2.924861	down			
RNA44662|UCSC_2675_2772	0.000325749	2.73E-05	2.938367	down			
RNA158823|XLOC_008955	0.000174669	1.19E-05	2.94212	down			
RNA34765|NRED_898_1473	3.8583E-05	1.42E-06	2.942745	down			
RNA164928|XLOC_006253	5.04313E-05	2.1E-06	2.953494	down			
RNA38127|RefSeq_32_8733	0.000195465	1.38E-05	2.999083	down			
RNA147248|p0352_imsncRNA771	5.81184E-05	2.61E-06	3.012812	down			
RNA58858|asoverlaps_457_1803	0.000270029	2.13E-05	3.013008	down			

RNA165832|XLOC_008190	1.68792E-05	4.4E-07	3.013901	down			
RNA147467|p0571_imsncRNA243	0.001058877	0.000137	3.035837	down			
RNA34420|NRED_552_2059	0.019061297	0.007364	3.055115	down			
RNA147091|nc-HOXC11-111	0.001222195	0.000167	3.059174	down			
RNA147604|p0708_imsncRNA479	0.000374405	3.3E-05	3.062569	down			
RNA39525|RefSeq_1514_2189	0.000448753	4.25E-05	3.089074	down			
RNA58784|asoverlaps_364_2003	0.011647875	0.003803	3.098538	down			
RNA158216|XLOC_001052	0.008548178	0.002494	3.106553	down			
RNA64002|RNAz_4722_214	2.45197E-05	7.55E-07	3.113137	down			
RNA42557|UCSC_113_7541	1.40933E-05	3.33E-07	3.134536	down			
RNA160371|XLOC_011369	0.00151823	0.000225	3.13727	down			
RNA157949|XLOC_000828	1.0876E-05	2.31E-07	3.154682	down			
RNA34434|NRED_566_2018	0.000448789	4.25E-05	3.163831	down			
RNA161830|XLOC_001748	0.004735897	0.001091	3.180015	down			
RNA39914|RefSeq_1935_1795	0.000221176	1.63E-05	3.196585	down			
RNA161482|XLOC_001475	0.000653627	7.07E-05	3.205273	down			
RNA147407|p0511_imsncRNA86	0.00026053	2.02E-05	3.239804	down			
RNA161027|XLOC_012707	5.59328E-05	2.47E-06	3.245987	down			
RNA52933|H-InvDB_191_799	6.34918E-06	1.03E-07	3.253273	down			
RNA159508|XLOC_009900	0.000269523	2.12E-05	3.27191	down			
RNA147236|p0340_imsncRNA761	2.08352E-06	1.8E-08	3.284167	down			
RNA42796|UCSC_406_5476	0.000135178	8.48E-06	3.284447	down			
RNA34309|NRED_440_2266	0.000685697	7.58E-05	3.297519	down			
RNA61395|RNAz_2109_268	2.06621E-06	1.78E-08	3.327528	down			
RNA160043|XLOC_011075	3.31782E-05	1.15E-06	3.330924	down			
RNA162830|XLOC_002773	0.002782582	0.000522	3.333955	down			
RNA50438|UCSC_9731_881	0.003019595	0.000586	3.336799	down			
RNA40073|RefSeq_2101_1659	0.001805117	0.000287	3.350563	down			
RNA52103|UCSC_11731_220	4.13173E-06	5.39E-08	3.362306	down			
RNA64672|RNAz_5393_200	0.002687668	0.000498	3.375178	down			
RNA176492|ENST00000506034	3.61233E-05	1.29E-06	3.40001	down			
RNA160438|XLOC_011889	0.008804108	0.002597	3.405342	down			
RNA147159|p0263_imsncRNA449	0.000512298	5.09E-05	3.411493	down			
RNA47162|UCSC_5763_1815	7.81139E-05	4E-06	3.412641	down			
RNA63911|RNAz_4631_216	0.00067345	7.39E-05	3.414772	down			
RNA61782|RNAz_2496_252	0.000111911	6.62E-06	3.434544	down			

RNA162794|XLOC_002743	0.000764489	8.79E-05	3.454693	down				
RNA63414|RNAz_4132_227	0.000913925	0.000112	3.457686	down				
RNA48980|UCSC_7980_1282	0.000643762	6.93E-05	3.493878	down				
RNA147439|p0543_imsncRNA163	0.000179214	1.23E-05	3.494851	down				
RNA46243|UCSC_4655_2071	0.001228837	0.000168	3.515582	down				
RNA34492|NRED_624_1917	0.000950862	0.000118	3.546781	down				
RNA159879|XLOC_010719	0.001798025	0.000285	3.556827	down				
RNA162550|XLOC_014378	0.002212385	0.000379	3.60467	down				
RNA163546|XLOC_004208	1.95345E-05	5.44E-07	3.614494	down				
RNA161410|XLOC_001412	2.936E-05	9.63E-07	3.64486	down				
RNA160332|XLOC_011341	0.001537273	0.000229	3.648575	down				
RNA96723|EvoFold_573_203	0.000323145	2.7E-05	3.64969	down				
RNA62237|RNAz_2955_240	0.001525978	0.000227	3.656115	down				
RNA64045|RNAz_4765_212	0.000323162	2.7E-05	3.659238	down				
RNA160538|XLOC_011978	5.22609E-05	2.23E-06	3.67163	down				
RNA34291|NRED_422_2303	8.85176E-08	6.6E-11	3.712493	down				
RNA47598|UCSC_6311_1690	6.11645E-05	2.82E-06	3.712736	down				
RNA39151|RefSeq_1115_2608	0.003255952	0.00065	3.770528	down				
RNA63067|RNAz_3785_233	0.000143351	9.19E-06	3.774846	down				
RNA49721|UCSC_8854_1079	0.002101598	0.000354	3.780878	down				
RNA43437|UCSC_1173_3851	1.87231E-06	1.56E-08	3.808113	down				
RNA165547|XLOC_007456	0.000473929	4.57E-05	3.870544	down				
RNA159918|XLOC_010767	1.73869E-05	4.6E-07	3.873168	down				
RNA61053|RNAz_1767_276	1.89805E-05	5.21E-07	3.875094	down				
RNA43037|UCSC_698_4630	0.000265624	2.08E-05	3.881237	down				
RNA162869|XLOC_002825	5.93213E-06	9.29E-08	3.886982	down				
RNA158845|XLOC_008645	0.002196086	0.000375	3.889041	down				
RNA59662|RNAz_373_364	1.05896E-05	2.22E-07	3.897175	down				
RNA61617|RNAz_2331_260	8.3802E-05	4.44E-06	3.901163	down				
RNA50334|UCSC_9602_917	6.77667E-05	3.26E-06	3.916286	down				
RNA166136|ENST00000497150	1.18145E-05	2.58E-07	3.946444	down				
RNA60710|RNAz_1424_280	0.000184079	1.27E-05	3.961293	down				
RNA39480|RefSeq_1465_2231	0.000107418	6.28E-06	3.97266	down				
RNA43954|UCSC_1797_3337	2.1773E-06	1.95E-08	3.978845	down				
RNA61321|RNAz_2035_270	3.54817E-05	1.25E-06	3.989935	down				
RNA163148|XLOC_003910	0.000670657	7.34E-05	3.991542	down				

RNA64449|RNAz_5170_200	1.48417E-05	3.62E-07	4.008947	down			
RNA49951|UCSC_9132_1018	0.000395486	3.57E-05	4.064694	down			
RNA50049|UCSC_9246_994	0.000206852	1.49E-05	4.094498	down			
RNA162987|XLOC_003341	8.61834E-06	1.64E-07	4.099771	down			
RNA62021|RNAz_2736_240	0.001666385	0.000256	4.148251	down			
RNA44712|UCSC_2734_2743	3.16796E-05	1.07E-06	4.161163	down			
RNA60606|RNAz_1319_285	8.86886E-05	4.81E-06	4.167293	down			
RNA147023|p0127_imsncRNA205	0.005290212	0.001275	4.17835	down			
RNA162826|XLOC_002771	0.000171181	1.15E-05	4.200608	down			
RNA163073|XLOC_003852	2.04363E-07	2.49E-10	4.2099	down			
RNA35081|NRED_1222_606	2.48953E-05	7.71E-07	4.220274	down			
RNA165146|XLOC_007088	0.000839468	9.92E-05	4.228041	down			
RNA59040|asoverlaps_692_1235	1.7831E-07	2.04E-10	4.228967	down			
RNA61729|RNAz_2443_254	1.75556E-05	4.67E-07	4.240573	down			
RNA60898|RNAz_1612_278	0.000123011	7.49E-06	4.414322	down			
RNA163274|XLOC_003595	0.000835003	9.86E-05	4.416974	down			
RNA61925|RNAz_2640_244	5.19621E-07	1.36E-09	4.525614	down			
RNA61264|RNAz_1978_271	6.35157E-05	2.97E-06	4.556218	down			
RNA45738|UCSC_4028_2247	8.80224E-06	1.69E-07	4.565077	down			
RNA158640|XLOC_008450	0.000440419	4.14E-05	4.575962	down			
RNA162827|XLOC_002772	3.71358E-05	1.34E-06	4.607642	down			
RNA46277|UCSC_4696_2062	6.50484E-06	1.06E-07	4.653231	down			
RNA62442|RNAz_3160_238	8.60573E-05	4.61E-06	4.657827	down			
RNA60760|RNAz_1474_279	1.89132E-05	5.17E-07	4.749589	down			
RNA34650|NRED_782_1677	0.002639875	0.000485	4.751975	down			
RNA48132|UCSC_6948_1549	1.7682E-05	4.72E-07	4.865464	down			
RNA51840|UCSC_11438_359	0.000508876	5.04E-05	4.903184	down			
RNA147090|nc-HOXC11-110	0.00096985	0.000121	4.9158	down			
RNA34785|NRED_918_1438	4.22363E-07	8.93E-10	5.033478	down			
RNA96217|EvoFold_27_346	9.73151E-05	5.49E-06	5.045712	down			
RNA34643|NRED_775_1683	0.000161282	1.06E-05	5.057112	down			
RNA63357|RNAz_4075_228	2.42852E-06	2.35E-08	5.091064	down			
RNA58575|asoverlaps_90_3155	9.25634E-05	5.11E-06	5.099837	down			
RNA41082|RefSeq_3194_620	2.82914E-05	9.14E-07	5.115536	down			
RNA158418|XLOC_001242	5.67111E-07	1.62E-09	5.21768	down			
RNA46348|UCSC_4783_2041	3.7727E-06	4.73E-08	5.261055	down			

RNA63287|RNAz_4005_229	3.55427E-06	4.37E-08	5.303057	down				
RNA59657|RNAz_368_366	0.000238379	1.8E-05	5.567378	down				
RNA165822|XLOC_008182	6.09847E-05	2.81E-06	5.63512	down				
RNA34800|NRED_933_1416	1.75682E-05	4.68E-07	5.639698	down				
RNA62057|RNAz_2772_240	8.05463E-05	4.18E-06	5.697906	down				
RNA159951|XLOC_010797	5.16001E-05	2.18E-06	5.735771	down				
RNA164010|XLOC_005034	8.09177E-05	4.21E-06	5.941367	down				
RNA59994|RNAz_706_322	3.09025E-05	1.03E-06	6.000975	down				
RNA53341|H-InvDB_607_577	1.16168E-06	6.59E-09	6.094193	down				
RNA164350|XLOC_005764	2.23263E-06	2.05E-08	6.166314	down				
RNA45671|UCSC_3942_2276	3.46104E-06	4.15E-08	6.21152	down				
RNA160160|XLOC_011172	0.000466641	4.48E-05	6.252511	down				
RNA51469|UCSC_11018_508	1.72367E-05	4.54E-07	6.294493	down				
RNA64482|RNAz_5203_200	0.000933261	0.000115	6.354741	down				
RNA60258|RNAz_970_311	9.16257E-06	1.79E-07	6.632392	down				
RNA53538|H-InvDB_808_501	2.37988E-06	2.28E-08	6.782416	down				
RNA51523|UCSC_11085_486	5.98434E-05	2.73E-06	6.7862	down				
RNA58672|asoverlaps_215_2428	4.4572E-05	1.74E-06	6.879971	down				
RNA160620|XLOC_012047	3.97477E-05	1.49E-06	7.164741	down				
RNA58543|asoverlaps_56_3602	0.000159297	1.05E-05	8.056541	down				
RNA58977|asoverlaps_609_1447	1.75393E-06	1.39E-08	8.57328	down				
RNA46481|UCSC_4960_2001	4.69869E-07	1.13E-09	8.653803	down				
RNA161481|XLOC_001474	6.14541E-07	2.02E-09	9.522642	down				
RNA176487|ENST00000499071	3.83333E-07	7.37E-10	9.599827	down				
RNA176485|ENST00000499142	4.76149E-07	1.2E-09	10.07562	down				
RNA60377|RNAz_1089_304	0.000311004	2.57E-05	10.11505	down				
RNA45103|UCSC_3211_2517	1.69079E-06	1.3E-08	10.42342	down				
RNA96413|EvoFold_236_255	2.89036E-06	3.06E-08	10.435	down				
RNA163502|XLOC_004175	2.17206E-05	6.28E-07	10.60027	down				
RNA53704|H-InvDB_981_444	1.23352E-07	1.11E-10	10.8266	down				
RNA34623|NRED_755_1719	1.08816E-05	2.31E-07	11.30485	down				
RNA39891|RefSeq_1908_1832	0.00012638	7.76E-06	11.37915	down				
RNA39109|RefSeq_1070_2676	5.93718E-07	1.79E-09	11.7986	down				
RNA163894|XLOC_004926	6.22146E-08	1.71E-11	13.01283	down				
RNA49576|UCSC_8684_1119	9.50797E-06	1.89E-07	14.18826	down				
RNA176486|ENST00000501311	3.02726E-06	3.28E-08	16.1293	down				

RNA38975|RefSeq_925_2910	6.00858E-06	9.48E-08	16.23977	down			
RNA38871|RefSeq_813_3099	2.02002E-06	1.74E-08	16.71151	down			
RNA160619|XLOC_012046	1.81378E-06	1.48E-08	16.98744	down			
RNA163361|XLOC_004067	8.22811E-07	3.61E-09	18.25316	down			
RNA53728|H-InvDB_1005_436	1.75095E-06	1.38E-08	28.20718	down			

Table S2: Significantly and differentially expressed mRNA in tumor tissues compared with NTL

ProbeName	p (Corr)	p	FC (abs)	Regulation	Entrez GeneID	Gene Symbol	
CB_004233	0.049639564	0.02531603	2.185746	up			
CB_007907	0.048648644	0.02467685	2.189062	up	222962	SLC29A4	
CB_020109	0.04864431	0.02467187	2.504026	up	55530	SVOP	
CB_026141	0.048526745	0.02459448	2.161952	up	145482	PTGR2	
CB_019034	0.048514962	0.02458394	3.008995	up	50940	PDE11A	
CB_002229	0.048514962	0.02458351	3.065834	up			
CB_020631	0.04850777	0.0245757	2.494927	up	57126	CD177	
CB_008307	0.0483917	0.02449974	2.008884	up	390748	PABPN1L	
CB_020091	0.04832788	0.02445775	2.15827	up	57214	KIAA1199	
CB_014488	0.048005123	0.02426101	2.824818	up	6370	CCL25	
CB_018205	0.047907244	0.02420235	3.921805	up	26085	KLK13	
CB_030376	0.047292534	0.02380033	2.196423	up	149992	ANKRD30BP2	
CB_029014	0.047159526	0.02371991	2.794377	up	387129	NPSR1	
CB_003313	0.047019605	0.02363242	3.337253	up			
CB_021407	0.046948474	0.02358997	2.125306	up	2898	GRIK2	
CB_025871	0.04694732	0.02358864	2.278057	up	54715	RBFOX1	
CB_021011	0.046928883	0.02357641	2.357296	up	57709	SLC7A14	
CB_003062	0.046664353	0.02341466	2.838748	up			
CB_008568	0.04664763	0.02340109	2.621854	up	728118	FAM22A	
CB_003063	0.046619307	0.02338319	2.866632	up			
CB_010101	0.046370078	0.02322878	2.575517	up	83416	FCRL5	
CB_021329	0.046278536	0.02317412	3.938586	up	5015	OTX2	
CB_026729	0.045977283	0.02298609	2.048044	up	29949	IL19	
CB_023678	0.045618992	0.02276896	2.025228	up	84654	SPZ1	
CB_028467	0.045618992	0.02276906	2.423965	up	346689	KLRG2	
CB_018418	0.045270704	0.02254385	2.309394	up	51617	HMP19	
CB_017733	0.045121353	0.02245589	2.247964	up	23105	FSTL4	
CB_027667	0.045097537	0.02244332	2.385831	up	339983	NAT8L	
CB_018138	0.04484888	0.02228758	3.289143	up	25984	KRT23	
CB_030549	0.044622645	0.02215593	2.183568	up	284424	C19orf30	
CB_024965	0.044208676	0.02188231	2.029942	up	160065	PATE1	
CB_012738	0.043925673	0.02170531	2.49329	up	8745	ADAM23	
CB_002014	0.043925673	0.0217041	2.60469	up			
CB_031457	0.04392147	0.02169929	2.061565	up	100130539	LOC100130539	
CB_023984	0.043875758	0.02166835	2.118228	up	56667	MUC13	
CB_010861	0.043850474	0.02165031	2.305268	up	1404	HAPLN1	
CB_006959	0.04370229	0.02156398	3.37302	up	338879	RNASE10	
CB_020090	0.043673724	0.02154642	2.08026	up	57214	KIAA1199	
CB_009446	0.043501362	0.02143366	2.031565	up	729993	SHISA9	
CB_006121	0.043073226	0.02118462	2.079051	up	312	ANXA13	
CB_023762	0.04295463	0.02111116	3.60542	up	9118	INA	
CB_013316	0.04258828	0.02087325	2.853809	up	2045	EPHA7	
CB_027884	0.04177443	0.0203584	2.194746	up	5570	PKIB	
CB_025225	0.041606776	0.02025759	2.081123	up	130951	C2orf65	
CB_015097	0.041474484	0.0201728	2.05924	up	5544	PRB3	
CB_017637	0.041121535	0.01995419	3.518616	up	9515	STXBP5L	
CB_004414	0.041083366	0.01993111	2.524662	up			
CB_018932	0.040251784	0.01942111	2.496876	up	51557	LGSN	
CB_009767	0.040205017	0.01939027	2.738561	up	57057	TBX20	
CB_015786	0.0398582	0.01917245	4.983169	up	7348	UPK1B	

CB_013863	0.039682336	0.0190715	2.338417	up	3798	KIF5A	
CB_014883	0.03959879	0.01901566	2.540233	up	9951	HS3ST4	
CB_014087	0.039361104	0.01888155	2.00953	up	1747	DLX3	
CB_025842	0.03926777	0.01882993	2.206435	up	55964	3-Sep	
CB_014153	0.03903058	0.01868649	2.058078	up	2831	NPBWR1	
CB_006380	0.03863629	0.01845607	2.086424	up	219487	OR5M11	
CB_029017	0.038617708	0.01844475	2.003435	up	676	BRDT	
CB_008157	0.03854265	0.01839912	2.028505	up	7412	VCAM1	
CB_027501	0.03720153	0.01759614	4.119896	up	347733	TUBB2B	
CB_020904	0.037094597	0.01753498	2.576063	up	57586	SYT13	
CB_014992	0.037035484	0.0174994	2.842918	up	4747	NEFL	
CB_012986	0.036802057	0.01735877	2.014503	up	1690	COCH	
CB_003147	0.03676676	0.0173392	2.917137	up			
CB_011262	0.03668145	0.01728443	2.328313	up	3880	KRT19	
CB_008354	0.036632262	0.01725487	2.268486	up	644150	WIPF3	
CB_017021	0.036604445	0.01723538	2.066424	up	26253	CLEC4E	
CB_007255	0.035771422	0.01674051	2.28681	up	554235	ASPDH	
CB_003384	0.035739616	0.01672279	2.507057	up			
CB_006182	0.03542338	0.01654001	2.073008	up	440730	TRIM67	
CB_009343	0.03517964	0.01639218	2.132937	up	399968	PATE4	
CB_029748	0.034745485	0.01613591	2.180124	up	339788	LOC339788	
CB_016696	0.034536324	0.01600592	2.166379	up	29881	NPC1L1	
CB_008776	0.03444844	0.01595154	2.233383	up	91646	TDRD12	
CB_021403	0.034331404	0.01587993	2.444201	up	1996	ELAVL4	
CB_009151	0.033845283	0.01558593	2.363683	up	120376	C11orf93	
CB_014518	0.03381043	0.01556485	3.242602	up	6999	TDO2	
CB_003396	0.033719342	0.01551557	2.043992	up			
CB_018368	0.033659793	0.01547611	2.308226	up	51059	FAM135B	
CB_030042	0.033379663	0.01531694	2.013497	up	731779	LOC731779	
CB_017481	0.033333376	0.01528936	3.059576	up	9892	SNAP91	
CB_023321	0.033286512	0.01526258	2.102731	up	84070	FAM186B	
CB_001202	0.033151392	0.01518434	2.182797	up	728114	LOC728114	
CB_005392	0.03307072	0.01513922	2.015833	up	5897	RAG2	
CB_029796	0.03298002	0.01508075	2.726011	up	9808	KIAA0087	
CB_031434	0.03211119	0.01455722	2.043388	up	100130863	ZNF840	
CB_022818	0.032094255	0.01454474	2.227674	up	54361	WNT4	
CB_012385	0.03195818	0.01446556	2.356782	up	7712	ZNF157	
CB_027547	0.031945344	0.01445823	2.072942	up	340596	LHFPL1	
CB_022737	0.03185465	0.01440809	2.772724	up	80741	LY6G5C	
CB_006439	0.03176843	0.01435499	2.007171	up	254879	OR2T6	
CB_009426	0.031753905	0.01434591	2.033344	up	440854	CAPN14	
CB_024076	0.031751893	0.014344	3.358024	up	10317	B3GALT5	
CB_026383	0.031664297	0.01429589	2.272484	up	3205	HOXA9	
CB_013763	0.03163882	0.01428107	2.350817	up	9536	PTGES	
CB_004825	0.03149197	0.01420012	2.087808	up	653492	PSG10P	
CB_002754	0.031097706	0.01397255	2.78618	up			
CB_003325	0.030968294	0.01390115	2.128712	up			
CB_003488	0.030963048	0.0138983	2.196598	up	100132074	FOXO6	
CB_000231	0.030920321	0.01386883	2.607289	up	158434	LOC158434	
CB_024440	0.030910807	0.01386309	2.420975	up	114088	TRIM9	
CB_003051	0.030895222	0.01385267	2.999193	up	651536	LOC651536	
CB_003145	0.030688232	0.01373505	3.102583	up			
CB_027322	0.030315755	0.01351932	2.032836	up	10214	SSX3	
CB_030879	0.03016997	0.0134414	2.046359	up	644623	TPTE2P2	
CB_016538	0.029796151	0.01323754	2.730589	up	176	ACAN	
CB_025659	0.02975929	0.01321597	2.300809	up	138065	RNF183	

CB_032056	0.029578162	0.01311397	2.030855	up	727944	LOC727944	
CB_003039	0.029380906	0.01300043	3.38046	up			
CB_023323	0.029118717	0.0128491	2.324958	up	84072	HORMAD1	
CB_021718	0.029077426	0.012827	2.215856	up	6785	ELOVL4	
CB_010801	0.028867569	0.01270144	5.812151	up	1109	AKR1C4	
CB_021610	0.028634364	0.01256569	2.896383	up	64208	POPDC3	
CB_010053	0.02863366	0.01256463	2.083916	up	4897	NRCAM	
CB_003195	0.028456759	0.01245708	2.149383	up	100288884	LOC100288884	
CB_024581	0.028407663	0.01242478	2.648712	up	7477	WNT7B	
CB_024666	0.028298	0.01235797	2.339525	up	6899	TBX1	
CB_024022	0.028246153	0.01232979	2.25848	up	92211	CDHR1	
CB_031344	0.028214352	0.01231026	2.429489	up	401847	LOC401847	
CB_007639	0.028022561	0.01219815	2.173447	up	245929	DEFB115	
CB_015402	0.027839191	0.01209671	2.010771	up	10647	SCGB1D2	
CB_024974	0.027795421	0.0120702	2.015883	up	162494	RHBDL3	
CB_018137	0.027638858	0.01198863	3.142438	up	25984	KRT23	
CB_003043	0.027538812	0.01193912	2.914772	up			
CB_004048	0.027519548	0.01192856	2.567574	up	729461	LOC729461	
CB_013197	0.027485283	0.0119069	2.833533	up	429	ASCL1	
CB_016613	0.027376704	0.01184844	2.00722	up	29906	ST8SIA5	
CB_000928	0.027330602	0.01182329	2.158154	up	100128063	LOC100128063	
CB_031834	0.027283067	0.01179999	2.221977	up			
CB_029392	0.02728225	0.01179848	2.42846	up	245	ALOX12P2	
CB_031149	0.027229212	0.01177209	2.578957	up	160313	KRT19P2	
CB_010721	0.02719869	0.01175415	2.17307	up	735	C9	
CB_026889	0.02705558	0.01166565	2.685425	up	3756	KCNH1	
CB_030926	0.026976949	0.01162197	2.087437	up	401233	LOC401233	
CB_003813	0.026945781	0.01160256	2.203278	up			
CB_012513	0.026940947	0.01160006	2.135823	up	8419	BFSP2	
CB_010596	0.026914239	0.01158514	2.106412	up	119	ADD2	
CB_002002	0.026865778	0.0115596	4.358854	up			
CB_030063	0.026831668	0.01154024	2.418918	up	730811	LOC730811	
CB_008978	0.026780874	0.01151151	2.082914	up	5673	PSG5	
CB_026090	0.026575724	0.01139351	2.324198	up	132141	IQCF1	
CB_006809	0.02646078	0.01132913	2.034614	up	440603	BCL2L15	
CB_004133	0.02629791	0.0112423	2.022285	up			
CB_005750	0.026269494	0.01122641	2.012998	up	5406	PNLIP	
CB_008663	0.026107758	0.01113825	2.007245	up	51317	PHF21A	
CB_020231	0.026093535	0.01113094	3.51112	up	54474	KRT20	
CB_030524	0.026027769	0.01109464	2.132458	up	285375	LOC285375	
CB_029276	0.02592395	0.01103805	2.330986	up	339240	LOC339240	
CB_025750	0.025785847	0.01096615	2.087431	up	130399	ACVR1C	
CB_030488	0.025687167	0.01090829	2.095808	up	440040	LOC440040	
CB_018658	0.02566329	0.01089615	3.254912	up	51702	PADI3	
CB_022715	0.025632236	0.01088017	2.367924	up	50964	SOST	
CB_025258	0.025509518	0.01081266	3.106711	up	140469	MYO3B	
CB_021934	0.025493758	0.01080436	2.429665	up	3897	L1CAM	
CB_004575	0.025408987	0.01075756	2.113226	up			
CB_011998	0.02527617	0.01068851	2.298021	up	6423	SFRP2	
CB_004567	0.025065226	0.01057785	2.901464	up			
CB_006594	0.025053186	0.01056959	2.164295	up	347252	IGFBPL1	
CB_025788	0.025052661	0.01056897	2.325464	up	202865	C7orf33	
CB_022495	0.02496826	0.01052268	3.694886	up	79983	POF1B	
CB_013933	0.024890443	0.01047647	3.285437	up	5746	PTH2R	
CB_014925	0.024874559	0.01046545	2.263166	up	10369	CACNG2	
CB_013978	0.024799686	0.01042372	2.094578	up	9242	MSC	

CB_000157	0.024703877	0.01037484	2.967636	up			
CB_011018	0.024701364	0.0103709	2.249524	up	2596	GAP43	
CB_026490	0.024612112	0.01032104	4.384974	up	260436	C4orf7	
CB_006346	0.024603313	0.01031661	2.494665	up	125962	OR7G1	
CB_030617	0.024551138	0.01028548	2.304569	up	161635	CSNK1A1P1	
CB_014748	0.024227021	0.01010698	2.144424	up	4160	MC4R	
CB_002392	0.02421131	0.01009812	2.114136	up			
CB_020507	0.024021314	0.00999451	2.004709	up	56961	SHD	
CB_029233	0.024002083	0.00998422	2.349949	up			
CB_002122	0.02398711	0.00997685	2.238763	up			
CB_004979	0.023946231	0.00995567	2.431278	up	2261	FGFR3	
CB_000304	0.023755431	0.00985865	2.085919	up			
CB_029512	0.023480829	0.00971119	2.008779	up	649946	RPL23AP64	
CB_013243	0.02325918	0.00959657	2.246988	up	1047	CLGN	
CB_031550	0.023257064	0.00959367	2.17679	up	100506209	LOC100506209	
CB_017910	0.023088584	0.00950797	2.265633	up	23314	SATB2	
CB_011225	0.023079569	0.00950224	2.005742	up	3775	KCNK1	
CB_030672	0.022959061	0.00943226	3.65606	up	440173	LOC440173	
CB_001578	0.02290754	0.00940364	2.273016	up			
CB_004829	0.022848241	0.00936604	2.142401	up	228	ALDOAP2	
CB_029246	0.02280347	0.00934429	2.363539	up			
CB_006368	0.022796744	0.00933828	2.210472	up	448831	FRG2	
CB_029176	0.022784896	0.00933053	2.396148	up	401138	AMTN	
CB_008841	0.02273392	0.00930389	3.774802	up	57451	ODZ2	
CB_003966	0.0227331	0.00930307	2.188445	up			
CB_020439	0.022618044	0.00923962	2.287618	up	56896	DPYSL5	
CB_022488	0.022568604	0.00921334	2.28045	up	79977	GRHL2	
CB_000600	0.022517495	0.00918605	2.944268	up	100128386	LOC100128386	
CB_013735	0.022449296	0.00914613	2.309691	up	9480	ONECUT2	
CB_009329	0.022417096	0.00913013	2.007589	up	56474	CTPS2	
CB_025843	0.022333663	0.00908344	2.451321	up	55964	3-Sep	
CB_025486	0.02230621	0.00906909	2.765954	up	144406	WDR66	
CB_027841	0.022247069	0.00903709	2.099986	up	148066	ZNRF4	
CB_018613	0.02214139	0.00898005	2.015181	up	51151	SLC45A2	
CB_030902	0.021961143	0.00888702	2.167097	up	100129427	LOC100129427	
CB_025743	0.021921042	0.00886628	2.258445	up	124220	ZG16B	
CB_004678	0.021783018	0.00879387	2.225	up			
CB_000297	0.021766093	0.00878325	2.851861	up	100292680	LOC100292680	
CB_003033	0.021758206	0.00877937	3.059873	up			
CB_003346	0.02175553	0.00877691	2.527087	up			
CB_022494	0.021740125	0.00876797	3.622789	up	79983	POF1B	
CB_029403	0.021710765	0.00875231	2.224047	up	10740	RFPL1S	
CB_030365	0.021693757	0.00874374	3.788428	up	201283	AMZ2P1	
CB_024860	0.021664288	0.00872911	2.896843	up	170850	KCNG3	
CB_009244	0.021660374	0.00872651	2.042481	up	54798	DCHS2	
CB_000658	0.021649908	0.00872126	2.069732	up	730338	LOC730338	
CB_008413	0.021575196	0.00868062	2.374949	up	152138	PYDC2	
CB_007253	0.021372473	0.00857664	2.038334	up	389549	FEZF1	
CB_010662	0.021297773	0.00853552	2.846229	up	479	ATP12A	
CB_015095	0.021147253	0.00845173	2.762603	up	653247	PRB2	
CB_003695	0.02113561	0.00844607	2.544117	up			
CB_004769	0.021094596	0.00842399	2.003909	up			
CB_001260	0.020966997	0.00836107	2.36753	up	401220	LOC401220	
CB_031191	0.020914683	0.00833335	2.07576	up	7577	ZNF29P	
CB_005546	0.020849131	0.00829823	2.357649	up	777	CACNA1E	
CB_000277	0.020849131	0.00829887	2.749621	up	730020	LOC730020	

CB_012587	0.02081774	0.00828274	2.926639	up	11001	SLC27A2	
CB_004309	0.020688334	0.00821486	2.094293	up			
CB_003170	0.020662207	0.00820022	2.730685	up			
CB_022119	0.020635867	0.00818683	2.301428	up	79570	NKAIN1	
CB_007636	0.020559633	0.00814876	2.541361	up	338809	C12orf74	
CB_029795	0.020382453	0.00805786	2.179593	up	100144604	LOC100144604	
CB_031368	0.020162817	0.0079397	2.130337	up	100128088	LOC100128088	
CB_004354	0.020090533	0.007902	2.056288	up			
CB_009098	0.019927153	0.00781437	2.094513	up	118461	C10orf71	
CB_029614	0.019914666	0.00780726	2.253797	up	650655	ABCA17P	
CB_003160	0.019598885	0.00764506	2.928582	up			
CB_024112	0.019444153	0.00756549	2.113866	up	2712	GK2	
CB_002192	0.01943699	0.00756022	2.655395	up			
CB_030862	0.019329766	0.00750441	2.533139	up	404744	AAA1	
CB_010831	0.019319523	0.0074986	2.051107	up	1299	COL9A3	
CB_016823	0.019282734	0.00748065	2.25305	up	54989	ZNF770	
CB_002088	0.019242942	0.00745942	2.04718	up	338324	S100A7A	
CB_024861	0.019239927	0.00745776	2.342464	up	170850	KCNG3	
CB_013826	0.01910265	0.00738589	2.439557	up	1833	EPYC	
CB_006688	0.019078547	0.00737361	2.481203	up	8576	STK16	
CB_005265	0.019009283	0.007336	2.20084	up	3273	HRG	
CB_009360	0.018941047	0.00730062	2.445428	up	54549	SDK2	
CB_001556	0.01887595	0.00726479	2.355249	up	399832	DKFZp686M1136	
CB_003173	0.018710738	0.00718222	2.585239	up			
CB_029033	0.01868801	0.00717202	2.275084	up	131920	TMEM207	
CB_030007	0.018612716	0.00713752	2.015118	up	728723	LOC728723	
CB_011263	0.018512363	0.00708979	3.383653	up	3881	KRT31	
CB_008619	0.01845267	0.00705859	2.401131	up	23244	PDS5A	
CB_003520	0.018445607	0.00705443	2.264553	up			
CB_000975	0.018425634	0.00704474	4.193852	up	100129717	LOC100129717	
CB_010702	0.018417649	0.00704105	2.650365	up	656	BMP8B	
CB_021289	0.018373441	0.00701943	3.120939	up	59082	CARD18	
CB_014006	0.018336333	0.00700032	3.618035	up	9982	FGFBP1	
CB_017764	0.018118337	0.00689394	2.219839	up	23132	RAD54L2	
CB_000851	0.018008057	0.00683884	2.231215	up	643551	LOC643551	
CB_000603	0.017910358	0.00679308	2.342775	up	100128727	LOC100128727	
CB_005518	0.017802507	0.00673679	4.734831	up	218	ALDH3A1	
CB_002793	0.017758023	0.00671494	2.648221	up			
CB_020542	0.01775681	0.00671415	5.313235	up	57016	AKR1B10	
CB_031153	0.01769634	0.00668147	2.000107	up	283888	LOC283888	
CB_020181	0.01768737	0.0066778	2.642816	up	53820	DSCR6	
CB_012870	0.017679915	0.00667387	2.259785	up	8941	CDK5R2	
CB_022763	0.017581478	0.00662525	2.943609	up	29785	CYP2S1	
CB_031878	0.017550308	0.00661103	2.213715	up			
CB_022817	0.017542096	0.00660682	2.675715	up	53637	S1PR5	
CB_006337	0.017510945	0.00659216	2.040888	up	390326	OR6C76	
CB_030729	0.017510613	0.00659164	2.512824	up	340618	FAM41AY1	
CB_010604	0.01733875	0.00650413	2.475599	up	197	AHSG	
CB_021181	0.01732505	0.00649734	2.286595	up	10309	CCNO	
CB_013012	0.017317949	0.0064933	2.571115	up	2257	FGF12	
CB_020577	0.017304111	0.00648627	2.122859	up	26507	CNNM1	
CB_024002	0.01730177	0.00648496	2.495215	up	85300	ATCAY	
CB_029316	0.017263772	0.00646423	2.112022	up	7503	XIST	
CB_001530	0.01719222	0.00643	2.110956	up			
CB_001737	0.017167574	0.00641678	2.068898	up			
CB_031022	0.017087946	0.00637564	2.826502	up	338739	LOC338739	

CB_029943	0.017053563	0.00635822	2.094019	up	157627	LOC157627	
CB_022693	0.017043613	0.00635343	2.302655	up	80326	WNT10A	
CB_008056	0.01704295	0.00635276	2.136912	up	613212	CTXN3	
CB_001377	0.01704295	0.00635291	2.153598	up	400058	MKRN9P	
CB_013805	0.017041452	0.00635127	5.513941	up	793	CALB1	
CB_000153	0.016970651	0.00631708	2.482276	up	594839	SNORA33	
CB_025043	0.016922759	0.00629471	2.011568	up	93408	MYL10	
CB_031291	0.016881388	0.00627529	2.232285	up	729722	LOC729722	
CB_031135	0.01686489	0.00626782	2.462675	up	729966	LOC729966	
CB_000062	0.016783703	0.00622807	3.203826	up			
CB_009519	0.016699994	0.00619143	2.313173	up	389493	LOC389493	
CB_003668	0.016691783	0.00618655	2.243589	up			
CB_005893	0.016579896	0.00613378	2.53318	up	93273	LEMD1	
CB_019921	0.01646751	0.00607732	2.054599	up	55347	ABHD10	
CB_029561	0.016389424	0.00603838	2.593385	up	100132596	XGPY2	
CB_029039	0.01630817	0.00599913	4.222098	up	145741	C2CD4A	
CB_017815	0.01628525	0.0059876	2.860162	up	114088	TRIM9	
CB_002672	0.016253171	0.00597151	2.100749	up			
CB_003306	0.016247138	0.00596869	2.542049	up			
CB_010291	0.01624278	0.00596632	2.389568	up	1116	CHI3L1	
CB_007689	0.016213639	0.00595305	2.24181	up	343071	PRAMEF10	
CB_014163	0.01618995	0.00594127	2.026926	up	2847	MCHR1	
CB_031877	0.01614824	0.00591905	2.019107	up			
CB_004716	0.016112974	0.00590178	2.072147	up			
CB_013201	0.016041925	0.00586834	3.998767	up	547	KIF1A	
CB_004781	0.0160409	0.00586597	2.606161	up			
CB_031131	0.015965093	0.00582939	2.665074	up	283422	C12orf36	
CB_011207	0.015964095	0.00582878	2.005423	up	3714	JAG2	
CB_009096	0.015947927	0.00582128	2.009644	up	118461	C10orf71	
CB_003970	0.01594481	0.00581895	2.180392	up			
CB_012739	0.01590417	0.00580135	2.848253	up	8745	ADAM23	
CB_006899	0.015894428	0.00579679	2.110515	up	402381	SOHLH1	
CB_016023	0.015891658	0.00579553	2.187191	up	11255	HRH3	
CB_006275	0.015823051	0.00576021	2.61024	up	256148	OR4S1	
CB_002926	0.01574606	0.00572176	3.269098	up			
CB_014501	0.015742974	0.00572012	2.098562	up	6756	SSX1	
CB_011615	0.015679197	0.00569145	4.504324	up	5266	PI3	
CB_005861	0.015672944	0.00568744	3.217531	up	414061	DNAJB3	
CB_021588	0.015620489	0.00566246	2.192375	up	10913	EDAR	
CB_004664	0.015617183	0.00566102	2.439328	up			
CB_013360	0.015575847	0.00564183	2.331873	up	2591	GALNT3	
CB_020983	0.015568485	0.00563843	2.120745	up	65267	WNK3	
CB_001000	0.015558369	0.00563279	2.384037	up	873	CBR1	
CB_009547	0.015534863	0.00562132	2.025509	up	100288060	KRTAP16-1	
CB_023999	0.015508636	0.00560839	2.208393	up	85291	KRTAP4-2	
CB_031529	0.015480837	0.00559345	3.386185	up	100291104	LOC100291104	
CB_021879	0.015450452	0.00557645	2.760814	up	668	FOXL2	
CB_014262	0.015395015	0.00555071	2.677768	up	6373	CXCL11	
CB_019002	0.015354891	0.00553162	2.391262	up	5079	PAX5	
CB_029452	0.015340767	0.00552264	2.394133	up	359822	NCRNA00099	
CB_001121	0.015061765	0.00538805	2.399225	up	5827	PXMP2	
CB_030943	0.015017896	0.00536593	2.016618	up	285441	LOC285441	
CB_000241	0.01494421	0.00533344	2.173264	up	151438	LOC151438	
CB_010787	0.014898697	0.00531177	2.303672	up	1044	CDX1	
CB_000635	0.014895589	0.00531019	2.590187	up	9905	SGSM2	
CB_023324	0.014833058	0.00528084	2.129659	up	84072	HORMAD1	

CB_026215	0.014780209	0.00525664	3.188732	up	152404	IGSF11	
CB_031510	0.014759506	0.00524717	3.891361	up			
CB_002004	0.014732049	0.00523531	2.131723	up			
CB_010290	0.014649078	0.00519607	3.879248	up	1113	CHGA	
CB_030458	0.014556917	0.00515311	2.258031	up	387097	C6orf147	
CB_000191	0.014556917	0.00515308	2.298399	up	286126	LOC286126	
CB_026452	0.014551102	0.00515012	2.167395	up	166012	CHST13	
CB_004562	0.014493732	0.0051219	2.11888	up			
CB_015552	0.014483942	0.0051166	2.4321	up	10900	RUNDC3A	
CB_031380	0.014439951	0.00509503	2.001603	up	100130800	LOC100130800	
CB_001113	0.014422279	0.00508659	2.114804	up	84786	MGC12488	
CB_014302	0.01439884	0.00507535	2.043639	up	9210	BMP15	
CB_030482	0.014358446	0.00505665	2.066042	up	144486	LOC144486	
CB_028709	0.014325189	0.00504114	2.99387	up	170692	ADAMTS18	
CB_021880	0.014325189	0.00504122	3.767117	up	668	FOXL2	
CB_003372	0.014322868	0.00503994	2.430172	up			
CB_027106	0.014317586	0.00503763	2.753526	up	285051	C2orf61	
CB_022599	0.014312025	0.00503543	2.550412	up	80168	MOGAT2	
CB_009187	0.014281541	0.00502196	2.037967	up	127833	SYT2	
CB_007057	0.014258448	0.00501208	3.741684	up	6706	SPRR2G	
CB_014078	0.014238781	0.005002	3.331127	up	1475	CSTA	
CB_003837	0.014192884	0.00498183	2.126279	up			
CB_001412	0.01419173	0.00498098	2.292744	up			
CB_003777	0.014148917	0.00496393	2.059066	up			
CB_022642	0.014123663	0.0049535	2.378875	up	23120	ATP10B	
CB_025054	0.014029611	0.00491118	2.380095	up	112885	PHF21B	
CB_028618	0.01398073	0.00488542	2.014323	up	375612	LHFPL3	
CB_006149	0.013963414	0.00487804	2.569266	up	118611	C10orf90	
CB_013317	0.013955253	0.00487386	3.619193	up	2045	EPHA7	
CB_030842	0.013904226	0.00485009	2.672606	up	440044	SLC22A20	
CB_012784	0.013896676	0.00484702	2.258088	up	8811	GALR2	
CB_000807	0.013894657	0.00484549	2.051353	up	100129938	LOC100129938	
CB_024450	0.013879281	0.00483809	2.240998	up	116931	MED12L	
CB_003057	0.013868324	0.00483207	3.944602	up			
CB_027205	0.013839127	0.00481927	2.474896	up	7401	CLRN1	
CB_003386	0.013788015	0.00479578	2.301869	up			
CB_022995	0.013734818	0.00477156	2.013096	up	81931	ZNF93	
CB_000410	0.013678411	0.00474355	2.412281	up	284628	LOC284628	
CB_029990	0.013650186	0.0047314	2.988081	up	80307	FER1L4	
CB_010908	0.013645997	0.00472908	2.208585	up	1828	DSG1	
CB_031479	0.013594779	0.00470671	2.996208	up	400682	LOC400682	
CB_001714	0.013584751	0.0047014	2.344853	up			
CB_012339	0.013543148	0.00468202	2.244521	up	7474	WNT5A	
CB_002427	0.013518501	0.00466754	2.920381	up			
CB_003155	0.013440011	0.00463255	3.079144	up			
CB_028458	0.013307562	0.00457213	2.421711	up	341032	C11orf53	
CB_026912	0.013271545	0.00455471	3.429204	up	163778	SPRR4	
CB_003823	0.013240795	0.00453843	5.195307	up			
CB_003939	0.013230846	0.00453256	2.028657	up			
CB_025751	0.013192632	0.00451529	2.924941	up	130399	ACVR1C	
CB_027987	0.013169305	0.00450272	2.345808	up	254263	CNIH2	
CB_026413	0.013092919	0.00446497	2.037675	up	256130	TMEM196	
CB_011211	0.013057579	0.00444692	2.224092	up	3728	JUP	
CB_001318	0.013042082	0.00443962	2.090655	up	338579	LOC338579	
CB_005565	0.012981134	0.00441057	2.166954	up	1141	CHRNB2	
CB_027462	0.012972668	0.00440636	4.086727	up	94033	FTMT	

CB_024810	0.012961417	0.00440103	3.075706	up	129684	CNTNAP5	
CB_030846	0.012910654	0.00437621	2.06857	up	284116	KRT42P	
CB_025517	0.01290051	0.00437154	2.977809	up	480	ATP1A4	
CB_027340	0.012838959	0.00434438	2.100751	up	256646	C15orf55	
CB_019746	0.012812632	0.004331	2.551413	up	55220	KLHDC8A	
CB_022562	0.012788761	0.00432111	2.201171	up	80117	ARL14	
CB_005530	0.012773185	0.00431382	2.002094	up	495	ATP4A	
CB_014683	0.01276187	0.00430919	2.762903	up	10249	GLYAT	
CB_030401	0.012739893	0.00430015	2.329866	up	283332	LOC283332	
CB_029989	0.012738423	0.00429905	2.30352	up	80307	FER1L4	
CB_006807	0.012731816	0.00429298	2.350509	up	441168	FAM26F	
CB_004393	0.012698004	0.00427863	3.141627	up			
CB_027941	0.012679078	0.004269	2.61081	up	132724	TMPRSS11B	
CB_009857	0.01266393	0.00426267	4.338561	up	92211	CDHR1	
CB_025378	0.01264564	0.0042517	2.339213	up	11202	KLK8	
CB_004224	0.012635233	0.0042478	2.321192	up			
CB_003158	0.012616499	0.0042393	2.847753	up			
CB_000957	0.012612611	0.00423679	3.660151	up	100130442	LOC100130442	
CB_009361	0.012607985	0.00423453	2.361419	up	54549	SDK2	
CB_031999	0.012607985	0.00423484	2.487069	up	197196	TMEM148	
CB_023022	0.012591663	0.00422616	2.93519	up	83417	FCRL4	
CB_005576	0.012531117	0.00420122	2.094318	up	1544	CYP1A2	
CB_029876	0.012423633	0.00415421	2.071134	up	439934	C4orf11	
CB_015400	0.012391334	0.00414007	2.024895	up	10644	IGF2BP2	
CB_014555	0.012389709	0.00413923	2.606496	up	10057	ABCC5	
CB_022479	0.012388636	0.00413779	2.267769	up	79966	SCD5	
CB_003711	0.012380634	0.00413414	2.271651	up			
CB_000901	0.01228777	0.00409125	2.472946	up			
CB_021457	0.012286763	0.00409072	2.647505	up	43847	KLK14	
CB_003156	0.012229931	0.0040654	3.366827	up	100290415	LOC100290415	
CB_003852	0.012213139	0.00405924	3.711364	up			
CB_015080	0.012194705	0.0040504	2.513711	up	5457	POU4F1	
CB_028888	0.012014034	0.00396906	3.889365	up	10683	DLL3	
CB_026996	0.011993561	0.00395983	2.17119	up	148753	FAM163A	
CB_006260	0.011964768	0.00394558	2.263048	up	119749	OR4C46	
CB_014269	0.011939895	0.00393586	4.963901	up	6707	SPRR3	
CB_003956	0.011935702	0.0039329	2.204201	up			
CB_030342	0.011925191	0.00392818	2.233724	up	145978	NCRNA00052	
CB_005915	0.011920912	0.00392564	2.028018	up	346517	OR6V1	
CB_016815	0.0118914	0.00391134	2.763648	up	170680	PSORS1C2	
CB_031672	0.011857634	0.00389596	2.290832	up	729863	LOC729863	
CB_003006	0.011850812	0.00389203	2.000454	up			
CB_004923	0.011849756	0.00389053	2.172032	up	1278	COL1A2	
CB_011738	0.011849756	0.00389056	2.250075	up	5655	KLK10	
CB_014206	0.011818498	0.00387655	2.181718	up	3149	HMGB3	
CB_031703	0.011773733	0.00385739	2.504397	up	283710	LOC283710	
CB_029015	0.011727915	0.00383977	2.863615	up	387129	NPSR1	
CB_000890	0.011712926	0.00383171	2.236412	up	2185	PTK2B	
CB_002101	0.011708517	0.00382952	2.157809	up			
CB_004415	0.011703919	0.00382715	3.08055	up			
CB_025741	0.011640833	0.00379963	2.104175	up	122651	RNASE11	
CB_014825	0.011637705	0.00379806	4.323119	up	6698	SPRR1A	
CB_003041	0.011632921	0.00379558	2.685617	up			
CB_009546	0.0115503	0.00375983	2.061693	up	128387	TATDN3	
CB_012709	0.01154381	0.00375662	2.006786	up	8712	PAGE1	
CB_012961	0.011526024	0.00374773	2.663359	up	1015	CDH17	

CB_031180	0.011525837	0.00374748	2.286779	up	26783	SNORA65	
CB_029479	0.011445246	0.00371368	2.259861	up	148709	LOC148709	
CB_004265	0.011371077	0.00368238	2.25348	up			
CB_004377	0.011337701	0.00366816	2.336571	up			
CB_005566	0.011329793	0.00366452	2.253597	up	1142	CHRNB3	
CB_002432	0.011229648	0.00362145	2.075064	up			
CB_001295	0.01120957	0.00361267	2.286612	up	400622	LOC400622	
CB_029736	0.011157878	0.00359052	2.163198	up	151300	LOC151300	
CB_003038	0.011121529	0.0035753	2.47691	up			
CB_010002	0.01103439	0.00353837	2.425603	up	2668	GDNF	
CB_000697	0.011009454	0.00352688	2.419249	up	100131048	LOC100131048	
CB_007824	0.011003215	0.00352331	3.130361	up	401089	C3orf72	
CB_020731	0.010940713	0.00349705	2.062107	up	56675	NRIP3	
CB_004228	0.010936184	0.00349439	2.069944	up			
CB_004038	0.010921009	0.00348798	2.518803	up			
CB_009398	0.010909957	0.00348307	2.849844	up	5328	PLAU	
CB_003151	0.010884406	0.00347336	3.394757	up			
CB_031522	0.010869971	0.00346651	3.463996	up	100289079	LOC100289079	
CB_007177	0.01085382	0.00345947	3.282262	up	7253	TSHR	
CB_027361	0.010850785	0.00345833	3.200162	up	4105	MAGEA6	
CB_003251	0.010720178	0.00340481	2.14613	up			
CB_000980	0.010713178	0.00340182	2.110076	up	100133286	LOC100133286	
CB_007738	0.010703377	0.00339795	2.726961	up	399473	SPRED3	
CB_013086	0.0106949	0.00339407	2.049456	up	9066	SYT7	
CB_011347	0.010693698	0.00339301	2.00155	up	4113	MAGEB2	
CB_015854	0.010686964	0.00339036	2.0552	up	11081	KERA	
CB_029287	0.010660316	0.00337819	2.463519	up	84672	TTTY6	
CB_021928	0.010651156	0.00337461	2.87661	up	66004	LYNX1	
CB_006244	0.010630217	0.00336579	2.01027	up	26246	OR2L2	
CB_003674	0.010616554	0.00335911	2.059201	up			
CB_011261	0.01060741	0.00335504	4.602764	up	3866	KRT15	
CB_023629	0.01056884	0.00333965	2.317257	up	84553	C6orf168	
CB_005542	0.01056402	0.00333679	2.746892	up	774	CACNA1B	
CB_019167	0.01056367	0.00333635	3.371995	up	54769	DIRAS2	
CB_025070	0.010560352	0.00333496	2.722806	up	113730	KLHDC7B	
CB_007844	0.010510507	0.00331173	2.023214	up	8710	SERPINB7	
CB_004349	0.010510507	0.00331189	3.184436	up			
CB_006768	0.010491547	0.00330259	3.11275	up	253017	TECRL	
CB_014854	0.010448326	0.00328402	4.420073	up	8128	ST8SIA2	
CB_023161	0.010440163	0.00328079	3.627453	up	53940	FTHL17	
CB_015409	0.01040081	0.00326397	3.940327	up	10655	DMRT2	
CB_002939	0.010281689	0.00321175	2.112612	up			
CB_008335	0.01026207	0.00320416	2.078715	up	1370	CPN2	
CB_010384	0.010196531	0.00317836	2.171461	up	1769	DNAH8	
CB_002566	0.0101683	0.00316779	2.016836	up			
CB_018193	0.010123952	0.00314948	2.243159	up	26059	ERC2	
CB_010803	0.010117653	0.00314688	3.398958	up	1114	CHGB	
CB_003422	0.010101046	0.00314005	2.382056	up			
CB_000835	0.01009078	0.00313532	2.460848	up	388560	FLJ45949	
CB_022231	0.010067642	0.00312526	2.307391	up	79698	ZMAT4	
CB_002020	0.010050194	0.00311745	2.015988	up			
CB_019038	0.010023589	0.00310523	3.098864	up	3229	HOXC13	
CB_015387	0.009991101	0.00309231	2.441504	up	8190	MIA	
CB_014424	0.009945181	0.003074	3.774623	up	284217	LAMA1	
CB_030871	0.00994167	0.00307197	5.217551	up	344887	LOC344887	
CB_025259	0.009907174	0.00305676	3.709623	up	140469	MYO3B	

CB_008695	0.009872504	0.00304137	2.366546	up	89	ACTN3	
CB_001245	0.009865222	0.00303865	2.767236	up	1588	CYP19A1	
CB_031680	0.00984242	0.00302788	2.030705	up	100510642	LOC100510642	
CB_030353	0.009835381	0.00302463	2.235545	up	26070	DKFZP434K028	
CB_030233	0.009817046	0.00301697	2.793671	up	440905	LOC440905	
CB_003153	0.009755488	0.00299093	4.303061	up			
CB_004119	0.009752194	0.00298924	2.65213	up			
CB_031208	0.009749373	0.00298798	2.631001	up	728528	LOC728528	
CB_031256	0.009749041	0.00298757	2.041586	up	653203	LOC653203	
CB_029110	0.009745454	0.00298616	3.18797	up	389208	TMPRSS11F	
CB_016875	0.009738253	0.00298318	4.060566	up	1834	DSPP	
CB_026621	0.009737019	0.00298203	2.045747	up	161514	TBC1D21	
CB_016816	0.009699359	0.00296727	2.60458	up	29113	C6orf15	
CB_021056	0.009640115	0.00294322	2.56814	up	6326	SCN2A	
CB_001705	0.009613614	0.00293235	2.193894	up	387055	C6orf38	
CB_021267	0.009564978	0.00291281	3.256953	up	58524	DMRT3	
CB_027880	0.009504063	0.00288914	2.036027	up	341567	H1FNT	
CB_004927	0.009499106	0.00288688	2.467279	up	1289	COL5A1	
CB_010696	0.009498365	0.00288651	4.700477	up	646	BNC1	
CB_008140	0.009477579	0.00287688	2.838094	up	150786	LOC150786	
CB_001964	0.009462502	0.00287156	3.397344	up			
CB_008142	0.009460989	0.0028705	2.132892	up	255798	C3orf43	
CB_031108	0.009430788	0.00285745	3.18815	up	643201	LOC643201	
CB_001546	0.009430788	0.0028574	3.726298	up			
CB_002449	0.009428454	0.00285644	2.303	up			
CB_031758	0.009395679	0.00284277	2.559036	up			
CB_028768	0.009373847	0.0028341	2.697085	up	166824	RASSF6	
CB_021871	0.009367608	0.00283176	2.196154	up	773	CACNA1A	
CB_022467	0.009367608	0.00283168	2.613577	up	79953	TMEM90B	
CB_030942	0.009344728	0.00282145	2.094675	up	440518	LOC440518	
CB_029967	0.009336499	0.00281689	2.949256	up	54075	NCRNA00157	
CB_009140	0.00925439	0.00278141	2.086646	up	57553	MICAL3	
CB_001234	0.009242067	0.00277683	2.139014	up	253832	ZDHHC20	
CB_023710	0.009228498	0.00277187	2.002568	up	84694	GJA10	
CB_026348	0.009214746	0.00276584	4.537425	up	154664	ABCA13	
CB_017998	0.00919622	0.0027578	2.037728	up	23509	POFUT1	
CB_004635	0.009188522	0.00275491	2.84307	up			
CB_000891	0.009187333	0.00275441	2.794092	up			
CB_004617	0.009157073	0.00274287	2.779501	up			
CB_011376	0.009151354	0.00274029	2.15219	up	4232	MEST	
CB_017040	0.00914433	0.00273775	4.867232	up	26998	FETUB	
CB_027273	0.009116248	0.00272587	2.168349	up	283576	ZDHHC22	
CB_030851	0.009115788	0.00272535	3.060391	up	94236	HEJ1	
CB_008106	0.009093466	0.00271315	3.123975	up	90161	HS6ST2	
CB_003735	0.00909261	0.00271275	2.274142	up	84978	FRMD5	
CB_015218	0.009062661	0.00270123	3.525498	up	10481	HOXB13	
CB_003521	0.009040169	0.00269323	2.087708	up			
CB_027183	0.009020552	0.00268556	2.043196	up	3557	IL1RN	
CB_003623	0.009015583	0.00268291	2.247044	up	285966	FAM115C	
CB_014286	0.009009021	0.00268032	2.522035	up	7516	XRCC2	
CB_003594	0.008997232	0.00267488	2.215912	up	2274	FHL2	
CB_020379	0.00899608	0.00267422	6.165541	up	28234	SLCO1B3	
CB_001457	0.008993807	0.00267329	2.117143	up	730139	LOC730139	
CB_000437	0.008964405	0.00266214	2.456102	up	284014	LOC284014	
CB_020437	0.008961479	0.00266098	3.09804	up	56896	DPYSL5	
CB_014221	0.008946697	0.00265489	3.501537	up	4108	MAGEA9	

CB_004244	0.008910389	0.00264002	2.71579	up			
CB_000021	0.008895457	0.00263334	2.376209	up	85368	KIAA1654	
CB_008973	0.008876001	0.00262617	4.336653	up	401136	TMPRSS11BNL	
CB_025452	0.00886849	0.00262283	2.14356	up	131096	KCNH8	
CB_007497	0.008752125	0.00257648	5.45891	up	391365	SULT6B1	
CB_000056	0.008724174	0.00256493	2.010562	up	283131	NEAT1	
CB_003059	0.008696344	0.00255399	4.55789	up			
CB_031749	0.008683136	0.00254805	3.692919	up			
CB_018907	0.008676733	0.00254589	2.194524	up	51764	GNG13	
CB_025260	0.008663133	0.00254037	3.228136	up	81832	NETO1	
CB_003161	0.00864783	0.00253426	4.59302	up			
CB_003570	0.008630485	0.0025266	2.034991	up			
CB_029134	0.008629493	0.00252615	2.646874	up	400360	C15orf54	
CB_013104	0.008574172	0.00250635	3.718411	up	9248	GPR50	
CB_031100	0.008573748	0.00250589	2.176187	up	100128881	LOC100128881	
CB_006794	0.008573183	0.00250559	3.216775	up	389396	GLYATL3	
CB_003733	0.008558893	0.00249911	2.104356	up			
CB_025319	0.008552482	0.00249571	2.297479	up	199713	NLRP7	
CB_020416	0.008545498	0.00249235	2.876298	up	56884	FSTL5	
CB_005366	0.008495528	0.00247346	2.233012	up	2668	GDNF	
CB_009982	0.008484629	0.00246852	2.299498	up	579	NKX3-2	
CB_010241	0.008472737	0.0024632	4.235367	up	771	CA12	
CB_031934	0.008470953	0.00246202	2.453377	up	100130169	LOC100130169	
CB_015067	0.008425086	0.00244527	2.244543	up	5368	PNOC	
CB_027983	0.008419879	0.0024417	2.344788	up	222662	LHFPL5	
CB_031403	0.008411499	0.0024378	2.235712	up	100129144	LOC100129144	
CB_004242	0.008405017	0.00243552	7.853265	up			
CB_002721	0.008401222	0.00243349	2.136655	up			
CB_022232	0.008400717	0.00243308	3.091815	up	79698	ZMAT4	
CB_012341	0.0083334	0.00240605	2.172607	up	7480	WNT10B	
CB_016019	0.008312905	0.00239869	3.337501	up	11250	GPR45	
CB_001768	0.008306093	0.00239606	2.531471	up	724066	ATXN8	
CB_020053	0.008305839	0.00239546	3.607292	up	55502	HES6	
CB_030866	0.008290396	0.00238864	2.022928	up	57212	KIAA0495	
CB_008838	0.00826995	0.00238131	2.433272	up	5825	ABCD3	
CB_031756	0.008205483	0.00235611	3.392139	up			
CB_009292	0.008205255	0.00235592	2.964326	up	80313	LRRC27	
CB_030015	0.008164293	0.00233833	2.277676	up	100128164	LOC100128164	
CB_025134	0.008159049	0.00233644	2.050321	up	64919	BCL11B	
CB_030256	0.008060466	0.00229799	2.096797	up	100129482	ZNF37BP	
CB_007862	0.008060466	0.00229812	5.386255	up	154215	NKAIN2	
CB_000544	0.008051995	0.00229403	2.257683	up	57582	KCNT1	
CB_030250	0.00802925	0.00228527	2.57423	up	26138	C8orf71	
CB_030154	0.008008553	0.00227773	2.206003	up	92014	MCART1	
CB_007678	0.008007325	0.00227725	2.029561	up	431704	RGS21	
CB_000353	0.007940228	0.0022528	2.0805	up	100131581	LOC100131581	
CB_011936	0.007936878	0.00225156	5.388558	up	6278	S100A7	
CB_017635	0.007901146	0.00223766	3.687715	up	9515	STXBP5L	
CB_012034	0.007875335	0.00222759	3.243694	up	6549	SLC9A2	
CB_009143	0.007861004	0.00222118	2.095565	up	5349	FXYD3	
CB_001270	0.00785847	0.00222009	2.773592	up	100270679	LOC100270679	
CB_006475	0.007822659	0.00220444	2.110273	up	390190	OR5B2	
CB_001159	0.007817358	0.00220234	2.747563	up	11158	RABL2B	
CB_029327	0.00776522	0.00218322	2.279258	up	387590	psiTPTE22	
CB_030510	0.007759234	0.00217988	2.537825	up	283856	LOC283856	
CB_014504	0.007716956	0.00216128	2.01393	up	6875	TAF4B	

CB_013953	0.007712584	0.00215932	3.150219	up	6511	SLC1A6	
CB_021949	0.007704899	0.00215656	2.663478	up	78988	MRP63	
CB_017500	0.007700216	0.00215507	5.906891	up	9911	TMCC2	
CB_005441	0.00769142	0.00215073	2.856591	up	3485	IGFBP2	
CB_022031	0.007687626	0.00214912	2.107937	up	79097	TRIM48	
CB_000229	0.007678258	0.00214496	2.43583	up	157278	LOC157278	
CB_013850	0.007655551	0.00213501	2.191695	up	3483	IGFALS	
CB_015732	0.007648194	0.00213183	2.663362	up	1087	CEACAM7	
CB_000379	0.007628317	0.0021252	2.313351	up	100130920	LOC100130920	
CB_026714	0.007624453	0.00212401	2.484458	up	158297	FAM154A	
CB_031971	0.007597048	0.00211372	2.458188	up	729739	LOC729739	
CB_030929	0.007504521	0.00207857	2.610675	up	644660	NCRNA00255	
CB_027303	0.007489425	0.00207274	3.194621	up	7499	XG	
CB_013070	0.007457131	0.0020586	3.717036	up	7345	UCHL1	
CB_026315	0.007456838	0.00205805	2.100687	up	199699	DAND5	
CB_025903	0.007446048	0.00205401	2.130375	up	253650	ANKRD18A	
CB_001537	0.007433991	0.00204958	2.53559	up	100128233	LOC100128233	
CB_012154	0.007428553	0.00204635	3.424916	up	6861	SYT5	
CB_030789	0.007419752	0.00204299	2.023856	up	641518	LOC641518	
CB_014533	0.007418633	0.00204244	2.780941	up	7681	MKRN3	
CB_021045	0.007417655	0.00204206	2.075002	up	30848	CTAG2	
CB_023673	0.007403652	0.00203703	2.484256	up	84648	LCE3D	
CB_014993	0.007390599	0.00203107	2.492691	up	4753	NELL2	
CB_003706	0.007377966	0.00202598	2.39084	up			
CB_005202	0.007377966	0.00202596	2.437075	up	6662	SOX9	
CB_030113	0.007375519	0.00202484	2.232242	up	100128675	LOC100128675	
CB_004928	0.007359522	0.00201975	2.955914	up	1289	COL5A1	
CB_031678	0.007351468	0.00201649	2.014893	up	100510286	LOC100510286	
CB_028612	0.007350877	0.00201622	2.078056	up	342667	STAC2	
CB_000005	0.007343924	0.00201373	2.317899	up			
CB_014937	0.007339654	0.00201197	3.346585	up	10389	SCML2	
CB_010164	0.007338527	0.00201132	2.305785	up	728591	C13orf38	
CB_030949	0.007307963	0.00200074	3.18808	up	100128593	LOC100128593	
CB_016340	0.007292651	0.00199481	2.481461	up	24141	C20orf103	
CB_005060	0.007287893	0.00199293	2.146524	up	3795	KHK	
CB_029239	0.007279984	0.00199029	2.814802	up			
CB_026384	0.007268584	0.00198523	2.039745	up	3205	HOXA9	
CB_000122	0.007247707	0.00197712	4.378278	up	59347	FKSG2	
CB_031633	0.007208982	0.00196358	3.961668	up	100508797	LOC100508797	
CB_023301	0.007206504	0.00196268	4.462192	up	63950	DMRTA2	
CB_031773	0.007200497	0.00196024	2.551298	up			
CB_032189	0.007137666	0.00193578	2.185155	up	100129484	LOC100129484	
CB_010100	0.007137666	0.00193568	2.622914	up	83416	FCRL5	
CB_023767	0.007137666	0.00193567	2.813642	up	144453	BEST3	
CB_003480	0.00711541	0.00192727	2.4352	up			
CB_001460	0.007087476	0.00191723	2.371765	up			
CB_022483	0.007065962	0.00190836	2.435577	up	79968	WDR76	
CB_021152	0.007056018	0.00190534	2.207793	up	2579	GAGE7	
CB_000850	0.007040138	0.00189873	2.970885	up	647008	FLJ46120	
CB_004803	0.007029746	0.00189548	3.621123	up			
CB_012982	0.007026824	0.00189454	2.605761	up	1607	DGKB	
CB_000198	0.007021176	0.00189228	2.138076	up	246318	FLJ13773	
CB_016759	0.00699842	0.00188347	2.110508	up	9641	IKBKE	
CB_021336	0.006998414	0.00188326	2.581644	up	10863	ADAM28	
CB_005688	0.006985897	0.00187876	3.932215	up	3358	HTR2C	
CB_015914	0.006963312	0.00187072	2.404541	up	7130	TNFAIP6	

CB_024532	0.006961196	0.00186994	5.167264	up	1592	CYP26A1	
CB_007691	0.006945599	0.00186443	2.735947	up	253012	HEPACAM2	
CB_015120	0.006931608	0.00185854	3.650441	up	6354	CCL7	
CB_001505	0.006917679	0.0018532	2.19049	up	440864	LOC440864	
CB_028515	0.006909201	0.00185014	2.536332	up	375519	GJB7	
CB_028786	0.006897839	0.00184565	2.198941	up	26470	SEZ6L2	
CB_013177	0.00688203	0.00183867	2.62509	up	9615	GDA	
CB_002399	0.006868487	0.0018336	2.004745	up			
CB_004659	0.006833782	0.00182029	2.386488	up			
CB_005052	0.006828846	0.00181836	2.252908	up	182	JAG1	
CB_003557	0.006824353	0.00181673	2.437936	up			
CB_014466	0.006812841	0.00181259	2.187811	up	5454	POU3F2	
CB_021714	0.006776107	0.00179906	2.033493	up	8220	DGCR14	
CB_014384	0.006773446	0.00179803	2.077555	up	3195	TLX1	
CB_005010	0.006771917	0.00179725	2.11575	up	2779	GNAT1	
CB_001007	0.006740761	0.00178657	2.198699	up	26069	DKFZP586B0319	
CB_008371	0.006730839	0.0017832	2.22165	up	83445	GSG1	
CB_004660	0.006727065	0.00178166	2.22276	up			
CB_032076	0.006715165	0.00177755	3.331625	up	553137	LOC553137	
CB_028356	0.0067115	0.00177605	2.239838	up	8838	WISP3	
CB_004753	0.00671006	0.00177546	2.791088	up			
CB_027958	0.00671006	0.00177539	3.228171	up	151647	FAM19A4	
CB_008554	0.006705136	0.00177309	2.1506	up	497189	TIFAB	
CB_031708	0.006699016	0.00177045	2.017272	up	402269	LOC402269	
CB_029469	0.006682686	0.00176345	2.411375	up	574029	DUSP5P	
CB_031827	0.006678593	0.00176216	2.001523	up			
CB_022175	0.006666397	0.0017583	2.815598	up	79639	TMEM53	
CB_003019	0.006653306	0.0017538	2.088638	up	644815	FAM83G	
CB_016571	0.006641673	0.00175041	2.929997	up	29114	TAGLN3	
CB_030535	0.006637617	0.00174913	2.200722	up	100129075	C14orf33	
CB_027362	0.006631734	0.00174685	4.457961	up	4105	MAGEA6	
CB_024734	0.006620657	0.0017431	2.367833	up	140883	ZNF280B	
CB_017176	0.006618585	0.00174212	2.116589	up	29850	TRPM5	
CB_008355	0.006617828	0.00174182	2.587397	up	644150	WIPF3	
CB_031025	0.006587416	0.00173047	2.202459	up	389906	LOC389906	
CB_013594	0.006580333	0.00172776	2.645367	up	9162	DGKI	
CB_030159	0.006577789	0.0017269	3.267096	up	114041	C21orf88	
CB_018154	0.006564726	0.00172264	2.0534	up	26007	DAK	
CB_015933	0.006533261	0.00171055	2.397616	up	7634	ZNF80	
CB_027186	0.006506585	0.0017015	2.176641	up	138046	RALYL	
CB_006437	0.006497901	0.00169763	2.161445	up	441608	OR5B3	
CB_022752	0.006466728	0.00168678	2.313903	up	80775	TMEM177	
CB_030571	0.006463142	0.00168482	3.491732	up	100101938	LOC100101938	
CB_001939	0.006450933	0.00168071	2.383258	up			
CB_003056	0.006445282	0.00167873	3.196495	up			
CB_010829	0.006438068	0.00167576	2.793102	up	1297	COL9A1	
CB_006842	0.006433719	0.00167439	2.52926	up	4110	MAGEA11	
CB_032117	0.006427409	0.00167244	2.21257	up	100129596	LOC100129596	
CB_004020	0.006370814	0.00165348	6.156283	up			
CB_025905	0.006367821	0.0016523	2.253198	up	259239	WFDC11	
CB_003747	0.006353707	0.00164732	3.792634	up			
CB_032149	0.006323382	0.00163746	2.063639	up	400454	UNQ9370	
CB_032073	0.006310899	0.00163236	2.666902	up	100132501	LOC100132501	
CB_005924	0.00630172	0.00162905	2.435243	up	400935	IL17REL	
CB_000418	0.006282988	0.00162311	2.21699	up	729051	LOC729051	
CB_024572	0.00627862	0.00162139	3.953885	up	1029	CDKN2A	

CB_005410	0.006274256	0.00161966	2.302046	up	7428	VHL	
CB_002823	0.006262834	0.00161532	2.035545	up			
CB_024246	0.006258916	0.00161369	2.448337	up	90293	KLHL13	
CB_011310	0.006255014	0.00161152	2.239736	up	4017	LOXL2	
CB_004930	0.006249938	0.00160942	2.466883	up	1311	COMP	
CB_010924	0.006238119	0.00160559	3.196095	up	1917	EEF1A2	
CB_015367	0.006238119	0.00160532	3.406236	up	6336	SCN10A	
CB_031514	0.006237774	0.001605	2.543625	up	644717	LOC644717	
CB_003476	0.006230928	0.00160285	2.135972	up			
CB_002451	0.006228845	0.00160221	2.205526	up			
CB_029047	0.006213961	0.00159701	2.184476	up	152519	NIPAL1	
CB_002896	0.006212265	0.00159593	2.790896	up	100132159	LOC100132159	
CB_024621	0.006208986	0.00159484	5.087011	up	89777	SERPINB12	
CB_002398	0.006187707	0.00158692	2.586996	up			
CB_031682	0.006167025	0.00157966	2.342093	up	100510596	LOC100510596	
CB_008025	0.006154666	0.00157523	6.213675	up	758	MPPED1	
CB_019039	0.006149584	0.00157295	2.07514	up	7976	FZD3	
CB_000707	0.006122952	0.0015641	2.111776	up	100130657	LOC100130657	
CB_004227	0.006122781	0.00156396	2.124113	up			
CB_013632	0.00611691	0.00156208	5.066552	up	9245	GCNT3	
CB_010281	0.006099756	0.00155624	2.238762	up	1041	CDSN	
CB_016880	0.006093798	0.00155376	2.447659	up	3227	HOXC11	
CB_000592	0.00609367	0.00155363	2.017754	up	100131043	LOC100131043	
CB_030265	0.006093253	0.00155336	2.524448	up	78998	C8orf51	
CB_028462	0.006091188	0.00155271	2.04578	up	344838	PAQR9	
CB_013278	0.006073246	0.00154602	2.968817	up	1746	DLX2	
CB_030002	0.006067016	0.00154404	3.189717	up	647946	LOC647946	
CB_014386	0.006048684	0.00153727	2.091722	up	3207	HOXA11	
CB_011814	0.00602792	0.00152902	2.039183	up	5798	PTPRN	
CB_013256	0.00602792	0.00152918	2.093097	up	1381	CRABP1	
CB_000579	0.006015697	0.00152435	2.436359	up			
CB_030213	0.005989825	0.00151585	2.459911	up	284800	FAM182A	
CB_004701	0.005985498	0.00151409	2.228873	up			
CB_011399	0.005966806	0.00150851	5.460282	up	4319	MMP10	
CB_001716	0.005966736	0.00150831	2.566083	up			
CB_016505	0.005954066	0.00150426	2.525676	up	26872	STEAP1	
CB_027187	0.005939738	0.00149812	2.751357	up	138046	RALYL	
CB_006663	0.005937451	0.00149695	2.186156	up	494143	CHAC2	
CB_002168	0.005914878	0.00148892	2.446873	up			
CB_002458	0.005911654	0.00148773	2.006534	up			
CB_003745	0.005909504	0.00148654	4.414542	up			
CB_016882	0.005873061	0.00147401	2.088849	up	3235	HOXD9	
CB_018617	0.005866729	0.00147204	2.187496	up	51412	ACTL6B	
CB_004565	0.005859394	0.00146854	2.337268	up			
CB_031893	0.005847239	0.00146474	2.079432	up	647215	C1orf81	
CB_003599	0.005847239	0.00146457	2.291906	up			
CB_013356	0.005837156	0.00146077	2.05355	up	2346	FOLH1	
CB_009439	0.005829662	0.00145813	2.794436	up	134111	UBE2QL1	
CB_030110	0.005828813	0.00145771	2.049946	up	100128977	LOC100128977	
CB_031266	0.005814516	0.00145317	2.432482	up			
CB_002848	0.005808904	0.00145119	2.453723	up			
CB_012307	0.005790373	0.00144344	2.990443	up	7368	UGT8	
CB_006310	0.005779498	0.00144027	2.082272	up	119692	OR51S1	
CB_000829	0.005747003	0.00142861	2.327933	up	100130152	LOC100130152	
CB_001367	0.005739904	0.00142576	2.73522	up	100127904	LOC100127904	
CB_009152	0.005732376	0.00142271	2.258379	up	149281	METTL11B	

CB_010849	0.005720593	0.00141897	3.066256	up	1358	CPA2	
CB_004284	0.005711145	0.00141599	2.164191	up			
CB_009980	0.005710003	0.00141503	2.020505	up	574	BAGE	
CB_001354	0.005710003	0.00141493	2.19497	up	401490	LOC401490	
CB_031385	0.005699278	0.00141097	2.141327	up	100131354	LOC100131354	
CB_006822	0.005693324	0.00140932	2.003814	up	55653	BCAS4	
CB_031818	0.005678372	0.00140364	4.355625	up			
CB_020281	0.005666429	0.0013988	3.315866	up	54544	CRCT1	
CB_026642	0.005665057	0.00139819	2.552875	up	5308	PITX2	
CB_000052	0.005630498	0.00138823	2.350796	up			
CB_031152	0.005623202	0.0013859	2.109752	up	154442	C6orf112	
CB_008649	0.00561266	0.0013825	2.029101	up	390594	KBTBD13	
CB_001235	0.005599547	0.00137838	2.012373	up	283665	LOC283665	
CB_005644	0.005594111	0.00137669	2.064111	up	2899	GRIK3	
CB_001539	0.005590897	0.00137501	2.235898	up	644919	LOC644919	
CB_023974	0.005589081	0.00137441	2.499745	up	66037	BOLL	
CB_023018	0.005577839	0.00137065	2.352275	up	56163	RNF17	
CB_015979	0.005530732	0.00135355	2.391295	up	11187	PKP3	
CB_015498	0.005522027	0.00135063	2.780389	up	10799	RPP40	
CB_032101	0.005519614	0.00134969	2.197418	up	100128300	LOC100128300	
CB_011033	0.005461188	0.00133125	2.017238	up	2730	GCLM	
CB_000386	0.005459967	0.00133087	2.186971	up			
CB_016435	0.005456667	0.00132967	2.392178	up	26212	OR2B6	
CB_013943	0.005448076	0.00132659	2.912348	up	6123	RPL3L	
CB_008351	0.005447054	0.00132599	2.167728	up	646480	FABP9	
CB_024117	0.005426141	0.0013188	2.096326	up	2567	GABRG3	
CB_008064	0.005419732	0.00131633	2.088968	up	6755	SSTR5	
CB_010387	0.005419732	0.0013163	2.486913	up	1775	DNASE1L2	
CB_003174	0.005407976	0.00131211	3.898513	up			
CB_028901	0.005406105	0.00131148	2.856422	up	3547	IGSF1	
CB_014185	0.005390786	0.00130701	2.970446	up	3010	HIST1H1T	
CB_003926	0.005390181	0.00130677	2.199389	up			
CB_012332	0.005359956	0.00129782	3.897112	up	7447	VSNL1	
CB_000774	0.005342681	0.00129182	2.701631	up			
CB_028596	0.005338694	0.00129047	2.083857	up	374393	FAM111B	
CB_018732	0.00533759	0.00128994	2.911244	up	51458	RHCG	
CB_001548	0.005323532	0.00128588	2.189599	up			
CB_002861	0.005297624	0.00127751	2.119977	up	54991	C1orf159	
CB_017826	0.00528999	0.00127468	2.065548	up	23217	ZFR2	
CB_005887	0.005251758	0.00126338	2.345727	up	6620	SNCB	
CB_021283	0.005243194	0.00126057	2.366971	up	58157	NGB	
CB_011004	0.005239645	0.00125931	2.140915	up	2444	FRK	
CB_003654	0.00521335	0.00125034	2.363868	up			
CB_014067	0.005212938	0.00125016	2.382671	up	1237	CCR8	
CB_018818	0.005208395	0.00124882	2.225025	up	26499	PLEK2	
CB_001454	0.005183432	0.00124078	2.080046	up	644242	LOC644242	
CB_009721	0.00516917	0.00123598	2.790864	up	4703	NEB	
CB_005186	0.005166066	0.0012345	2.184073	up	6331	SCN5A	
CB_005281	0.005156683	0.00123185	2.73201	up	4014	LOR	
CB_008718	0.005155122	0.00123131	7.51574	up	282973	JAKMIP3	
CB_010705	0.005154145	0.00123089	4.101328	up	667	DST	
CB_015777	0.005154145	0.00123077	4.336811	up	6703	SPRR2D	
CB_029521	0.00515137	0.00122992	2.126425	up	653399	GSTTP2	
CB_002537	0.005146427	0.00122825	2.005889	up			
CB_002041	0.005143905	0.00122708	2.346426	up			
CB_027810	0.005136149	0.00122427	2.16087	up	337960	KRTAP13-3	

CB_005599	0.005118811	0.00121866	3.289973	up	1594	CYP27B1	
CB_001348	0.005118123	0.00121833	2.863001	up			
CB_001459	0.005106942	0.0012143	2.766042	up			
CB_002777	0.005096301	0.00121064	2.200968	up	6518	SLC2A5	
CB_003877	0.005092171	0.00120877	2.279135	up			
CB_003166	0.005087107	0.00120724	5.768849	up			
CB_026076	0.005082769	0.00120565	2.114105	up	130162	C2orf63	
CB_030157	0.005080638	0.00120498	2.243641	up	114041	C21orf88	
CB_001569	0.005075913	0.0012037	2.854585	up	100128006	LOC100128006	
CB_009813	0.00507584	0.00120336	2.028376	up	401097	LOC401097	
CB_007131	0.00507305	0.00120214	2.921303	up	3776	KCNK2	
CB_022849	0.005060826	0.00119884	2.072268	up	81551	STMN4	
CB_000172	0.005060742	0.00119874	2.169877	up	647070	LOC647070	
CB_028613	0.005058864	0.00119781	2.272622	up	343641	TGM6	
CB_029674	0.005055218	0.00119643	2.365277	up	644145	LOC644145	
CB_020928	0.005049961	0.00119482	2.342735	up	57615	ZNF492	
CB_000166	0.00504965	0.00119467	3.075297	up			
CB_029849	0.005044247	0.00119315	2.237641	up	729522	AACSP1	
CB_002545	0.005042287	0.00119237	2.318469	up			
CB_015774	0.005026565	0.00118682	7.422284	up	6665	SOX15	
CB_022670	0.005012014	0.00118227	2.065398	up	80264	ZNF430	
CB_026987	0.004985579	0.00117392	2.613292	up	143279	HECTD2	
CB_032133	0.004975786	0.00117002	2.533634	up	100507199	LOC100507199	
CB_017298	0.004957492	0.0011632	4.676814	up	9705	ST18	
CB_014802	0.004924452	0.00115315	2.034322	up	4544	MTNR1B	
CB_001101	0.004924452	0.00115318	4.292009	up	541611	HSP90AB6P	
CB_014233	0.004922255	0.0011522	2.190045	up	4610	MYCL1	
CB_031513	0.004910328	0.0011484	2.345645	up			
CB_013131	0.004885309	0.00114046	2.144401	up	9368	SLC9A3R1	
CB_027192	0.004883472	0.00113995	2.289418	up	200634	KRTCAP3	
CB_030816	0.004879971	0.00113874	2.447774	up	400798	C1orf220	
CB_010706	0.004871153	0.00113607	4.016424	up	667	DST	
CB_003003	0.004859284	0.00113184	3.08734	up			
CB_023021	0.00485566	0.00113038	2.854729	up	83416	FCRL5	
CB_001635	0.004835579	0.00112371	2.183373	up	2974	GUCY1B2	
CB_023556	0.004833409	0.00112282	2.314494	up	84419	C15orf48	
CB_013320	0.004821285	0.00111924	2.732217	up	2048	EPHB2	
CB_027948	0.004813001	0.00111625	4.011859	up	144501	KRT80	
CB_030684	0.004811989	0.00111586	2.011946	up	84808	C1orf170	
CB_007471	0.00480372	0.00111341	2.276141	up	157855	KCNU1	
CB_002438	0.004798112	0.0011114	2.166721	up			
CB_000479	0.004794054	0.00110996	2.204705	up	286154	LOC286154	
CB_028135	0.004773925	0.00110397	2.315536	up	5352	PLOD2	
CB_019468	0.004772046	0.0011032	2.049411	up	55001	TTC22	
CB_023210	0.004769659	0.00110227	2.251303	up	83881	MIXL1	
CB_015260	0.004755661	0.00109783	2.809859	up	10537	UBD	
CB_002426	0.004755661	0.00109782	4.593906	up			
CB_001632	0.004755286	0.00109744	2.126508	up	100128988	LOC100128988	
CB_024075	0.004745701	0.00109425	2.033376	up	2253	FGF8	
CB_005016	0.004738711	0.00109211	2.255972	up	3000	GUCY2D	
CB_020406	0.00472442	0.00108739	2.558609	up	56604	TUBB4Q	
CB_015778	0.004715226	0.00108487	3.079763	up	6703	SPRR2D	
CB_007916	0.004708584	0.00108263	2.077551	up	284612	SYPL2	
CB_007562	0.004691677	0.00107705	2.470925	up	548313	SSX4B	
CB_001598	0.004688341	0.00107612	2.651869	up			
CB_009631	0.00468216	0.0010744	2.585519	up	100130988	C7orf72	

CB_008220	0.004675679	0.00107269	2.002291	up	7652	ZNF99	
CB_016869	0.00465945	0.0010672	3.172012	up	6334	SCN8A	
CB_026750	0.004656564	0.00106631	2.432757	up	200407	CREG2	
CB_023232	0.004628588	0.00105726	2.228086	up	83897	KRTAP3-2	
CB_027052	0.004617355	0.00105375	2.335916	up	89766	UMODL1	
CB_021136	0.004600592	0.00104832	5.593141	up	9076	CLDN1	
CB_026663	0.004598424	0.00104746	2.983283	up	266740	MAGEA2B	
CB_020508	0.004579973	0.00104197	2.279856	up	10509	SEMA4B	
CB_018718	0.004562234	0.0010369	3.056089	up	51450	PRRX2	
CB_027032	0.004554475	0.00103423	2.249494	up	203111	C8orf47	
CB_008840	0.004553815	0.00103398	3.679082	up	57451	ODZ2	
CB_011025	0.004552103	0.00103323	2.912082	up	2626	GATA4	
CB_024554	0.004545379	0.0010312	3.134904	up	118430	MUCL1	
CB_002132	0.004537803	0.00102854	3.822285	up			
CB_005975	0.004537528	0.00102834	2.396241	up	219981	OR5A2	
CB_007233	0.004520042	0.0010231	3.488699	up	6704	SPRR2E	
CB_006233	0.004482498	0.00101096	2.257136	up	138802	OR13C8	
CB_004178	0.004466159	0.00100579	2.038372	up			
CB_031280	0.004456652	0.00100266	2.016587	up	100133580	LOC100133580	
CB_026995	0.004456639	0.00100259	2.033337	up	148641	SLC35F3	
CB_030039	0.004439861	0.00099755	2.948961	up	648740	LOC648740	
CB_000667	0.004418779	0.00099106	2.099275	up			
CB_021215	0.004389771	0.00098239	4.265337	up	57834	CYP4F11	
CB_009114	0.004380727	0.00097988	3.08264	up	9378	NRXN1	
CB_001860	0.004359299	0.00097392	2.160632	up			
CB_007237	0.004342985	0.00096938	3.140213	up	388228	SBK1	
CB_031206	0.00434276	0.0009692	2.031157	up	649201	LOC649201	
CB_021860	0.00432828	0.00096472	2.112867	up	65108	MARCKSL1	
CB_026374	0.004325791	0.00096403	2.924022	up	221336	BEND6	
CB_031174	0.004305028	0.00095752	2.277402	up	442028	LOC442028	
CB_000146	0.004298623	0.00095532	2.259037	up	280665	LOC280665	
CB_021216	0.0042858	0.00095168	4.80117	up	57834	CYP4F11	
CB_004591	0.004270176	0.00094729	2.009096	up			
CB_004016	0.004256553	0.00094293	3.161271	up			
CB_009382	0.004255185	0.00094215	2.613847	up	341346	C12orf70	
CB_030142	0.004253465	0.00094115	2.840805	up	442459	LOC442459	
CB_029030	0.004253019	0.00094075	2.417519	up	92558	CCDC64	
CB_011820	0.004244354	0.00093803	7.152339	up	5803	PTPRZ1	
CB_023991	0.004239002	0.00093649	2.040847	up	81697	OR2B2	
CB_006551	0.00422586	0.00093253	3.36522	up	198437	C20orf201	
CB_021514	0.004224862	0.00093203	2.245767	up	63934	ZNF667	
CB_000179	0.004223154	0.00093158	2.037648	up			
CB_014077	0.004211675	0.00092752	2.003677	up	1448	CSN3	
CB_022579	0.004211486	0.00092741	2.44967	up	80139	ZNF703	
CB_031566	0.00420762	0.00092636	2.063652	up	100507610	LOC100507610	
CB_012085	0.004205951	0.00092579	2.227364	up	6659	SOX4	
CB_031281	0.004200399	0.0009241	2.077468	up	100130701	LOC100130701	
CB_006412	0.004183706	0.00091877	2.180222	up	26341	OR5H1	
CB_001236	0.004177678	0.0009168	2.103109	up	643623	LOC643623	
CB_002471	0.004174837	0.0009159	2.035872	up			
CB_020069	0.00416555	0.00091307	2.170052	up	55894	DEFB103B	
CB_010835	0.00416255	0.00091208	2.041436	up	1310	COL19A1	
CB_030969	0.004159088	0.00091112	2.052712	up	100129620	LOC100129620	
CB_005585	0.004141732	0.00090522	2.013159	up	1559	CYP2C9	
CB_006701	0.004141732	0.0009052	3.179782	up	64711	HS3ST6	
CB_009131	0.004099578	0.00089305	2.846671	up	221806	VWDE	

CB_027963	0.004073434	0.00088564	3.282345	up	164633	CABP7	
CB_019390	0.004060314	0.00088184	2.339502	up	54937	SOHLH2	
CB_024541	0.004050285	0.00087849	2.172504	up	7809	BSND	
CB_016258	0.004048076	0.00087756	2.783124	up	2299	FOXI1	
CB_005625	0.003995794	0.00086236	3.956737	up	2558	GABRA5	
CB_005499	0.003981297	0.00085841	8.15422	up	131	ADH7	
CB_018421	0.003973811	0.00085611	2.044092	up	51087	YBX2	
CB_010030	0.003957636	0.00085192	2.481938	up	100271846	HERV-V2	
CB_013028	0.00394129	0.0008473	3.904428	up	3174	HNF4G	
CB_013133	0.003940886	0.00084714	2.09978	up	9377	COX5A	
CB_009667	0.003933461	0.00084498	2.215801	up	347688	TUBB8	
CB_030421	0.00393048	0.00084422	2.121223	up	285456	LOC285456	
CB_003339	0.003927823	0.00084327	2.031866	up			
CB_007482	0.003924589	0.00084202	3.693021	up	7020	TFAP2A	
CB_019247	0.003914658	0.00083876	2.049401	up	54826	GIN1	
CB_025489	0.003902242	0.0008352	3.466974	up	144568	A2ML1	
CB_029086	0.003884706	0.00083047	4.260316	up	387695	C10orf99	
CB_025046	0.003879997	0.00082895	3.354885	up	112609	MRAP2	
CB_010192	0.003878942	0.00082864	3.720299	up	89766	UMODL1	
CB_011016	0.003863466	0.00082388	3.683379	up	2569	GABRR1	
CB_004751	0.003861671	0.00082338	2.594128	up			
CB_010629	0.003861671	0.00082335	5.920089	up	339	APOBEC1	
CB_009040	0.00385511	0.000821	2.177621	up	173	AFM	
CB_027576	0.003840642	0.00081688	2.103577	up	353133	LCE1C	
CB_009130	0.003823621	0.00081252	2.824642	up	221806	VWDE	
CB_025740	0.003818473	0.00081108	2.253468	up	122258	C13orf28	
CB_011389	0.003807647	0.00080764	3.623235	up	4283	CXCL9	
CB_000569	0.003806987	0.00080731	2.346649	up	645769	LOC645769	
CB_001941	0.003763454	0.00079396	2.376472	up			
CB_029528	0.003760264	0.00079287	2.086301	up	10230	NBR2	
CB_031728	0.003758764	0.0007925	2.069037	up	652147	LOC652147	
CB_010791	0.003747848	0.00078913	2.045018	up	1056	CEL	
CB_016685	0.00373744	0.00078587	2.137663	up	29802	VPREB3	
CB_006179	0.00373452	0.00078508	3.163124	up	440590	ZYG11A	
CB_021509	0.003719036	0.00078015	2.478628	up	63928	CHP2	
CB_025249	0.003713413	0.00077834	2.039525	up	124540	MSI2	
CB_031321	0.003712489	0.00077805	2.535794	up	647163	LOC647163	
CB_004667	0.003700691	0.00077448	2.13826	up			
CB_030409	0.003692288	0.00077203	2.401872	up	284440	LOC284440	
CB_006266	0.003687316	0.00077071	2.071915	up	390199	OR4D9	
CB_014114	0.003680947	0.00076875	2.571098	up	2290	FOXG1	
CB_008939	0.003650395	0.00076068	2.445619	up	100131137	BSPH1	
CB_018139	0.003649713	0.00076036	2.072295	up	25987	TSKU	
CB_011424	0.003635766	0.00075654	2.523443	up	4488	MSX2	
CB_002175	0.003634114	0.00075585	8.391342	up			
CB_003684	0.00361517	0.00075025	2.240203	up			
CB_010802	0.003587626	0.00074261	3.048027	up	1109	AKR1C4	
CB_023814	0.003565545	0.0007361	2.212867	up	84891	ZSCAN10	
CB_007187	0.003564044	0.00073568	3.464798	up	4915	NTRK2	
CB_004614	0.003562069	0.00073522	2.181412	up			
CB_022489	0.003546879	0.00073129	2.634642	up	79977	GRHL2	
CB_026548	0.003532817	0.00072726	2.032786	up	148113	CILP2	
CB_030168	0.003531064	0.00072663	2.223305	up	124149	ANKRD26P1	
CB_007152	0.003523405	0.00072433	2.353863	up	91862	MARVELD3	
CB_029858	0.003507103	0.00071976	2.195351	up	414777	HCG18	
CB_008740	0.003468582	0.00070973	2.014575	up	127255	LRRIQ3	

CB_006283	0.00346281	0.00070814	2.135273	up	401994	OR14I1	
CB_031239	0.00346281	0.00070814	2.869839	up	100130736	LOC100130736	
CB_017717	0.003456337	0.00070665	2.299533	up	6543	SLC8A2	
CB_016506	0.003447975	0.00070456	2.799424	up	26872	STEAP1	
CB_006236	0.003442191	0.00070261	2.152247	up	347468	OR13H1	
CB_011044	0.003438165	0.00070134	2.043281	up	2781	GNAZ	
CB_005498	0.003413802	0.00069374	13.94035	up	131	ADH7	
CB_010790	0.003411314	0.00069307	3.977708	up	1056	CEL	
CB_001782	0.00338732	0.00068647	2.012978	up	100130460	LOC100130460	
CB_023519	0.003384883	0.00068587	2.180672	up	84321	THOC3	
CB_018312	0.003366453	0.00068102	2.533516	up	50512	PODXL2	
CB_020195	0.003355997	0.00067832	2.920262	up	54346	UNC93A	
CB_004997	0.003352837	0.00067747	2.025769	up	2642	GCGR	
CB_007228	0.003352423	0.00067728	2.212956	up	10057	ABCC5	
CB_027511	0.003350438	0.00067677	2.056408	up	283748	PLA2G4D	
CB_008131	0.003340063	0.00067401	2.124673	up	2778	GNAS	
CB_010701	0.003331404	0.00067138	2.624677	up	656	BMP8B	
CB_013084	0.003317718	0.00066785	2.219542	up	8973	CHRNA6	
CB_003838	0.003299848	0.00066288	2.301271	up			
CB_004235	0.003299848	0.00066303	8.020301	up			
CB_030793	0.003290732	0.00066043	6.137615	up	400578	KRT16P2	
CB_030888	0.003285975	0.00065906	2.035473	up	645339	SNRPD2P2	
CB_019213	0.003281701	0.00065747	2.070074	up	54802	TRIT1	
CB_007138	0.003280579	0.00065714	5.152084	up	92196	DAPL1	
CB_001112	0.003268264	0.00065385	2.196377	up	440330	LOC440330	
CB_030359	0.003263067	0.00065271	2.522656	up	84740	AFAP1-AS1	
CB_016199	0.003255783	0.00065022	3.220309	up	23581	CASP14	
CB_024718	0.003255073	0.00065001	2.139011	up	129025	ZNF280A	
CB_025900	0.003254407	0.00064968	2.990318	up	127343	DMBX1	
CB_025191	0.003250403	0.00064847	2.091581	up	93081	C13orf27	
CB_004601	0.003248648	0.0006478	2.272915	up			
CB_002012	0.003239251	0.00064543	2.131452	up			
CB_024136	0.003237581	0.00064494	3.357519	up	94032	CAMK2N2	
CB_023067	0.003237441	0.00064486	3.830447	up	56169	GSDMC	
CB_031255	0.003236388	0.0006445	2.126499	up	653203	LOC653203	
CB_008303	0.003235462	0.00064421	5.923968	up	85452	KIAA1751	
CB_000154	0.00323531	0.00064412	2.348058	up			
CB_003549	0.00323531	0.00064413	2.47204	up			
CB_027550	0.003223368	0.00064119	2.00722	up	349565	NMNAT3	
CB_014376	0.00321565	0.00063899	2.954416	up	3110	MNX1	
CB_011269	0.00321565	0.00063898	3.701834	up	3889	KRT83	
CB_029667	0.003214385	0.00063854	2.55197	up	5369	PMCHL1	
CB_024499	0.003211027	0.00063746	2.058832	up	117159	DCD	
CB_000755	0.00320964	0.00063708	3.002497	up	399990	FLJ44874	
CB_003337	0.003206539	0.00063637	2.003726	up			
CB_014313	0.003192209	0.00063216	2.261387	up	9626	GUCA1C	
CB_001486	0.003167624	0.00062528	3.091406	up			
CB_002516	0.003160792	0.00062338	2.251095	up			
CB_000170	0.003151714	0.00062084	2.198377	up	379034	FLJ10489	
CB_009427	0.003150874	0.00062062	2.464719	up	440854	CAPN14	
CB_026512	0.003150596	0.00062052	2.031221	up	118738	ZNF488	
CB_017000	0.003135179	0.00061627	2.57851	up	23746	AIPL1	
CB_026269	0.003127939	0.00061447	2.021763	up	163081	ZNF567	
CB_003766	0.00312377	0.00061326	2.217279	up			
CB_004248	0.00311218	0.00061004	2.169019	up			
CB_009648	0.003111245	0.00060976	2.980215	up	5365	PLXNB3	

CB_018345	0.003098507	0.00060658	2.016834	up	51361	HOOK1	
CB_002560	0.003095073	0.0006053	2.243512	up			
CB_007071	0.003090402	0.00060406	3.610283	up	6705	SPRR2F	
CB_005567	0.003086889	0.00060313	3.011918	up	1143	CHRNB4	
CB_027897	0.003083974	0.0006023	2.031219	up	221527	ZBTB12	
CB_001608	0.00307701	0.00060037	2.234621	up			
CB_014453	0.003064053	0.00059701	2.856996	up	4617	MYF5	
CB_017489	0.003056407	0.00059516	2.300814	up	9900	SV2A	
CB_021945	0.003045793	0.00059249	2.005698	up	3219	HOXB9	
CB_014979	0.003024157	0.00058698	4.040314	up	2810	SFN	
CB_000019	0.003008505	0.00058233	2.073387	up	2064	ERBB2	
CB_025942	0.002995858	0.0005786	2.092223	up	149461	CLDN19	
CB_014195	0.002988668	0.00057655	3.01295	up	3050	HBZ	
CB_015821	0.002981341	0.00057457	2.119844	up	11045	UPK1A	
CB_007128	0.002974476	0.00057287	4.961963	up	6701	SPRR2B	
CB_024185	0.002969842	0.00057164	2.146716	up	114336	CGB2	
CB_006888	0.002963402	0.00056967	2.301173	up	4192	MDK	
CB_015849	0.002963402	0.00056975	2.521517	up	11077	HSF2BP	
CB_012101	0.002954473	0.00056737	14.22753	up	6699	SPRR1B	
CB_024954	0.002937183	0.00056298	2.58719	up	128817	CSTL1	
CB_024436	0.002935862	0.00056226	3.096006	up	116832	RPL39L	
CB_025991	0.002928264	0.00056053	2.860718	up	4589	MUC7	
CB_021706	0.002927557	0.00056035	3.420342	up	3224	HOXC8	
CB_019091	0.002913834	0.00055689	4.436995	up	26659	OR7A5	
CB_004053	0.002909237	0.00055573	3.682182	up			
CB_000224	0.002905471	0.00055488	2.155621	up			
CB_011417	0.002901255	0.00055357	2.247783	up	4440	MSI1	
CB_000388	0.002900628	0.0005534	2.063483	up	100128262	LOC100128262	
CB_028340	0.002887965	0.0005498	4.166827	up	29841	GRHL1	
CB_027292	0.002882386	0.00054842	2.304124	up	285955	SPDYE1	
CB_002512	0.002882386	0.00054846	2.508762	up			
CB_022744	0.00287886	0.00054748	2.02955	up	80759	KHDC1	
CB_023650	0.002868713	0.00054467	3.466951	up	84624	FNDC1	
CB_020510	0.002854551	0.00054116	2.004994	up	56963	RGMA	
CB_020726	0.002846853	0.00053935	2.069223	up	54165	DCUN1D1	
CB_002813	0.002846737	0.00053925	2.66344	up			
CB_002979	0.002840301	0.00053721	2.076612	up			
CB_006450	0.002834189	0.00053556	2.381921	up	282770	OR10AG1	
CB_031879	0.002816101	0.00053091	2.773145	up			
CB_026375	0.002813474	0.00053022	2.053635	up	221336	BEND6	
CB_015819	0.002811362	0.00052973	2.057725	up	10590	SCGN	
CB_018275	0.002805056	0.00052814	2.172177	up	27112	FAM155B	
CB_007861	0.002801132	0.00052696	3.648006	up	154215	NKAIN2	
CB_019947	0.002801066	0.0005269	2.645466	up	55811	ADCY10	
CB_027711	0.002799622	0.00052659	7.060399	up	204219	LASS3	
CB_003061	0.002798845	0.00052632	3.92616	up			
CB_010502	0.002783274	0.00052246	2.379144	up	2729	GCLC	
CB_013247	0.002779832	0.00052158	2.098086	up	1181	CLCN2	
CB_001721	0.002769812	0.00051869	2.220484	up			
CB_009870	0.002762853	0.00051643	2.282729	up	6530	SLC6A2	
CB_031314	0.002756105	0.00051486	2.078556	up	100130458	LOC100130458	
CB_021341	0.002754176	0.00051441	2.639169	up	10761	PLAC1	
CB_003679	0.002753755	0.00051421	2.31088	up			
CB_005596	0.00275132	0.00051353	3.2829	up	1591	CYP24A1	
CB_028795	0.002747658	0.00051263	2.485866	up	9333	TGM5	
CB_025699	0.002746522	0.00051233	2.199388	up	151354	FAM84A	

CB_030691	0.0027407	0.0005109	2.193591	up	283102	FLJ46111	
CB_020011	0.002734273	0.00050928	2.825007	up	55867	SLC22A11	
CB_008537	0.002734273	0.00050941	2.876961	up	445577	C9orf129	
CB_022952	0.002730323	0.00050814	2.614739	up	81792	ADAMTS12	
CB_012105	0.002730231	0.00050799	2.288559	up	6713	SQLE	
CB_004784	0.002713413	0.00050409	2.024469	up			
CB_012102	0.002711325	0.00050357	2.089206	up	6708	SPTA1	
CB_007747	0.002707283	0.00050231	3.491504	up	9633	MTL5	
CB_006265	0.00268626	0.00049713	2.146324	up	219983	OR4D6	
CB_026580	0.002672281	0.0004934	5.198704	up	255928	SYT14	
CB_025297	0.002670258	0.00049286	3.431546	up	140766	ADAMTS14	
CB_018784	0.002670147	0.00049275	2.026112	up	51213	LUZP4	
CB_027737	0.002664409	0.0004914	4.019875	up	116379	IL22RA2	
CB_023485	0.002664044	0.0004912	3.141049	up	84290	CAPNS2	
CB_010699	0.00265788	0.0004896	2.273366	up	655	BMP7	
CB_008123	0.002649148	0.00048749	2.11474	up	51611	DPH5	
CB_026979	0.00264743	0.00048709	2.417095	up	133482	SLCO6A1	
CB_002085	0.002640651	0.00048536	2.001639	up			
CB_013018	0.00263868	0.00048468	2.074417	up	2693	GHSR	
CB_023823	0.00263868	0.00048462	2.165934	up	84900	RNFT2	
CB_003776	0.002637593	0.00048431	2.593659	up			
CB_023716	0.002624178	0.00048082	2.144197	up	84700	MYO18B	
CB_004671	0.002620793	0.00048007	2.561028	up			
CB_002394	0.002616687	0.00047907	2.515171	up			
CB_001394	0.002616165	0.00047885	2.436599	up	283982	C17orf54	
CB_022668	0.002607625	0.00047671	2.054122	up	80263	TRIM45	
CB_019954	0.002606887	0.00047645	2.691598	up	55359	STYK1	
CB_013176	0.002606887	0.00047638	3.382242	up	9615	GDA	
CB_014505	0.002603057	0.00047496	2.068463	up	6875	TAF4B	
CB_019208	0.002600186	0.00047424	2.383857	up	55605	KIF21A	
CB_001967	0.002592004	0.00047258	2.274802	up	342850	ANKRD62	
CB_014334	0.002590291	0.00047194	2.10002	up	10024	TROAP	
CB_018762	0.002586588	0.0004711	3.179341	up	50805	IRX4	
CB_025414	0.002585706	0.00047079	2.320049	up	118932	ANKRD22	
CB_016926	0.002582709	0.00046996	2.263779	up	10331	B3GNT3	
CB_024953	0.002567369	0.00046601	2.197468	up	1748	DLX4	
CB_005376	0.002566132	0.0004655	5.392235	up	3239	HOXD13	
CB_002061	0.002557082	0.00046321	2.26984	up			
CB_011218	0.002555679	0.00046288	3.873788	up	3755	KCNG1	
CB_026969	0.00255565	0.00046283	2.377573	up	124590	USH1G	
CB_011270	0.002550508	0.00046149	2.032479	up	3891	KRT85	
CB_014487	0.002545527	0.00046016	2.471469	up	6370	CCL25	
CB_006319	0.002540397	0.00045903	2.803798	up	119678	OR52E2	
CB_002669	0.00251829	0.00045325	2.155302	up			
CB_015013	0.002507028	0.00045077	2.257416	up	4917	NTN3	
CB_007227	0.002504903	0.00045026	2.443673	up	10057	ABCC5	
CB_029591	0.002497416	0.00044852	2.106295	up	5820	PVT1	
CB_011069	0.002476834	0.00044359	7.197258	up	2979	GUCA1B	
CB_006486	0.00246899	0.00044169	2.35021	up	57055	DAZ2	
CB_026077	0.002457576	0.00043833	2.335489	up	130367	SGPP2	
CB_016995	0.002447459	0.00043586	3.970635	up	23657	SLC7A11	
CB_014422	0.002437208	0.00043364	3.866446	up	3898	LAD1	
CB_020732	0.002435747	0.00043332	2.240125	up	56675	NRIP3	
CB_024672	0.002435141	0.00043306	2.027066	up	90423	ATP6V1E2	
CB_000781	0.002434008	0.0004327	2.049126	up	100131129	LOC100131129	
CB_006478	0.00242552	0.00043065	2.341437	up	11200	CHEK2	

CB_026126	0.002391354	0.00042235	4.182163	up	143503	OR51E1	
CB_020371	0.002386316	0.00042102	4.342252	up	56300	IL1F9	
CB_003458	0.002384711	0.00042049	2.564564	up			
CB_015670	0.002380631	0.00041943	3.823075	up	10962	MLLT11	
CB_008531	0.002378717	0.00041887	9.3545	up	2786	GNG4	
CB_030554	0.002378592	0.00041877	2.037823	up	100128191	LOC100128191	
CB_015609	0.002375599	0.00041783	2.407181	up	7379	UPK2	
CB_024413	0.002373873	0.00041742	2.021178	up	115650	TNFRSF13C	
CB_023114	0.002373836	0.00041733	2.355951	up	83697	SLC4A9	
CB_013837	0.002371797	0.00041671	2.419375	up	2516	NR5A1	
CB_028737	0.002364297	0.00041483	2.047933	up	246181	AKR7L	
CB_009445	0.002362679	0.00041421	2.370387	up	387882	C12orf75	
CB_028005	0.002356161	0.00041284	2.002629	up	284451	ODF3L2	
CB_003069	0.002354388	0.00041235	2.15508	up			
CB_022694	0.002351794	0.00041174	2.972889	up	80328	ULBP2	
CB_008530	0.002351794	0.00041156	6.18736	up	2786	GNG4	
CB_022156	0.002348816	0.00041085	3.326761	up	79623	GALNT14	
CB_028616	0.002338341	0.0004088	2.079284	up	149998	LIPI	
CB_006063	0.002337363	0.00040851	2.975084	up	444882	IGFL4	
CB_019597	0.002335295	0.00040797	5.509296	up	55117	SLC6A15	
CB_004121	0.002332652	0.00040736	3.142379	up			
CB_009668	0.002312468	0.00040273	2.560366	up	347688	TUBB8	
CB_000626	0.002305544	0.00040098	2.450458	up			
CB_008965	0.002299381	0.00039943	3.019823	up	389903	CSAG3	
CB_011059	0.002284809	0.00039615	2.070611	up	2901	GRIK5	
CB_004091	0.002284809	0.00039617	2.38847	up			
CB_031659	0.002282611	0.00039551	2.34906	up	100129516	LOC100129516	
CB_020307	0.002274529	0.00039335	3.030094	up	54626	HES2	
CB_005097	0.002268767	0.00039217	2.308774	up	4625	MYH7	
CB_030440	0.002265748	0.00039136	2.830885	up	91149	LOC91149	
CB_032165	0.002262223	0.00039025	4.895297	up	100129297	LOC100129297	
CB_003928	0.002258132	0.00038927	2.052702	up			
CB_005205	0.002254909	0.00038842	3.613594	up	6770	STAR	
CB_031027	0.002246657	0.00038675	2.043966	up	100130452	LOC100130452	
CB_015378	0.002241993	0.0003858	2.322282	up	7697	ZNF138	
CB_012654	0.002235217	0.00038442	8.59536	up	8626	TP63	
CB_003114	0.002233339	0.00038387	2.667888	up			
CB_014826	0.002229111	0.00038295	3.535004	up	6698	SPRR1A	
CB_018311	0.002225304	0.00038176	2.762733	up	50512	PODXL2	
CB_008196	0.002224852	0.00038162	3.037559	up	645369	TMEM200C	
CB_026125	0.002224236	0.00038137	2.605007	up	143503	OR51E1	
CB_013670	0.002221686	0.00038083	4.669268	up	9355	LHX2	
CB_010907	0.002210439	0.0003781	11.54826	up	1825	DSC3	
CB_000131	0.002206497	0.00037718	3.231857	up			
CB_030352	0.002196721	0.00037498	3.762145	up	23574	PRG1	
CB_029478	0.002178299	0.00037103	2.188621	up	664618	HSP90AB4P	
CB_021344	0.002174589	0.00036983	2.025098	up	59067	IL21	
CB_023368	0.002172882	0.0003694	2.020494	up	84140	FAM161A	
CB_016756	0.002168219	0.00036843	2.373152	up	780	DDR1	
CB_000938	0.002168219	0.00036835	2.458517	up			
CB_020844	0.002159488	0.00036644	4.302209	up	57526	PCDH19	
CB_018925	0.00214724	0.00036378	2.370959	up	51286	CEND1	
CB_021288	0.00213828	0.00036175	9.185091	up	56033	BARX1	
CB_009353	0.00213667	0.00036136	2.005196	up	146779	EFCAB3	
CB_013419	0.002133324	0.00036064	2.602231	up	4703	NEB	
CB_029242	0.002118076	0.00035733	3.703898	up			

CB_011046	0.002117421	0.00035718	2.230483	up	2784	GNB3	
CB_017017	0.0021165	0.00035689	2.006583	up	25830	SULT4A1	
CB_028365	0.002114988	0.00035657	2.119597	up	4810	NHS	
CB_029606	0.00210776	0.00035512	2.300141	up	643486	LOC643486	
CB_002256	0.002087291	0.00035031	2.186922	up			
CB_019075	0.002080681	0.00034878	2.901588	up	4237	MFAP2	
CB_032156	0.002076616	0.00034793	2.391614	up	100127909	LOC100127909	
CB_011268	0.002076616	0.00034792	3.356015	up	3889	KRT83	
CB_021612	0.002076067	0.00034777	5.861704	up	64211	LHX5	
CB_010174	0.00206782	0.000346	3.388483	up	163589	TDRD5	
CB_022410	0.0020582	0.00034367	2.752254	up	79883	PODNL1	
CB_001932	0.002053197	0.00034261	2.245725	up			
CB_000060	0.002053197	0.00034256	3.113595	up			
CB_002115	0.002051016	0.00034201	2.063025	up			
CB_022212	0.00204198	0.00033988	4.741743	up	79679	VTCN1	
CB_003788	0.002040658	0.00033931	2.963167	up			
CB_012600	0.002035324	0.00033788	3.416874	up	8538	BARX2	
CB_011401	0.002025019	0.0003356	5.006406	up	4322	MMP13	
CB_021544	0.002023107	0.00033518	2.618518	up	64090	GAL3ST2	
CB_029516	0.002021217	0.00033474	3.487656	up	6702	SPRR2C	
CB_019276	0.002021084	0.00033466	2.10356	up	54857	GDPD2	
CB_013787	0.00201984	0.00033409	4.069883	up	728461	CSAG2	
CB_016150	0.002017909	0.00033367	3.624713	up	6493	SIM2	
CB_006614	0.002005759	0.00033081	2.161932	up	493829	TRIM72	
CB_019289	0.00199755	0.00032924	2.04397	up	54866	PPP1R14D	
CB_004246	0.001997072	0.00032913	4.602199	up			
CB_020617	0.001994703	0.00032861	2.986148	up	57116	ZNF695	
CB_029099	0.001986773	0.00032686	2.290224	up	388555	IGFL3	
CB_029165	0.001979068	0.000325	2.008168	up	8702	B4GALT4	
CB_001480	0.001974979	0.00032423	2.154093	up	552862	C8orf28	
CB_028017	0.001973418	0.00032394	17.20341	up	339967	TMPRSS11A	
CB_022291	0.001964164	0.0003219	5.155659	up	79755	ZNF750	
CB_011260	0.001963048	0.00032168	8.034234	up	3860	KRT13	
CB_002287	0.001949045	0.00031886	2.616069	up	27198	GPR81	
CB_020653	0.001947549	0.00031843	3.438088	up	57156	TMEM63C	
CB_017291	0.001945468	0.00031788	6.947606	up	9699	RIMS2	
CB_008764	0.001919689	0.00031254	2.142979	up	222183	SRRM3	
CB_026929	0.001919552	0.00031249	3.299448	up	27177	IL1F8	
CB_003225	0.001918354	0.0003122	2.02493	up			
CB_012188	0.001916502	0.00031151	2.273971	up	7022	TFAP2C	
CB_025537	0.001907706	0.00030944	4.383043	up	53833	IL20RB	
CB_019544	0.001907588	0.00030933	2.014841	up	55071	C9orf40	
CB_016158	0.001907045	0.00030912	2.190589	up	22809	ATF5	
CB_023014	0.00190649	0.000309	2.416973	up	56157	TEX13A	
CB_020062	0.001897753	0.00030725	2.677564	up	55891	LENEP	
CB_010658	0.001897044	0.00030708	2.144059	up	434	ASIP	
CB_025301	0.001896885	0.00030701	2.831592	up	91614	DEPDC7	
CB_029332	0.001893204	0.00030622	4.003584	up	84176	MYH16	
CB_006950	0.001886702	0.0003048	5.14923	up	5653	KLK6	
CB_026033	0.001863232	0.00029983	2.367793	up	124222	PAQR4	
CB_011366	0.001856111	0.00029812	2.119723	up	4185	ADAM11	
CB_013351	0.00185028	0.00029678	4.584738	up	2297	FOXD1	
CB_008693	0.001848297	0.00029617	2.227878	up	285888	CNPY1	
CB_000588	0.001848014	0.00029603	2.801565	up	100128950	LOC100128950	
CB_028079	0.001845115	0.00029534	2.724281	up	256764	WDR72	
CB_027188	0.001844606	0.00029521	2.167026	up	138046	RALYL	

CB_003040	0.001837928	0.00029387	11.66106	up			
CB_013318	0.00183679	0.00029363	2.747209	up	2047	EPHB1	
CB_031791	0.001836497	0.0002935	3.149458	up			
CB_026451	0.001832236	0.00029265	5.651073	up	169044	COL22A1	
CB_028339	0.001829121	0.00029193	4.289484	up	29841	GRHL1	
CB_001289	0.001822541	0.00029051	2.803582	up	201477	LOC201477	
CB_013825	0.001819042	0.00028975	5.65957	up	1823	DSC1	
CB_025500	0.001817734	0.00028946	2.134571	up	147015	DHRS13	
CB_022378	0.001800643	0.00028548	2.136742	up	79850	FAM57A	
CB_010570	0.001792225	0.00028338	3.180072	up	3627	CXCL10	
CB_000602	0.001788775	0.00028244	2.230953	up	100131000	LOC100131000	
CB_028445	0.001788775	0.00028246	2.533076	up	286077	FAM83H	
CB_022623	0.001786261	0.00028192	2.548965	up	80206	FHOD3	
CB_007976	0.001779058	0.00028046	2.027916	up	10592	SMC2	
CB_020286	0.001776111	0.00027973	2.973045	up	54551	MAGEL2	
CB_031126	0.001757731	0.00027587	2.505155	up	646903	LOC646903	
CB_019092	0.001757141	0.00027571	2.571464	up	26659	OR7A5	
CB_027929	0.001752993	0.00027481	2.467926	up	120935	CCDC38	
CB_005276	0.001731508	0.00027038	9.829088	up	3872	KRT17	
CB_014188	0.001730065	0.00027012	3.071203	up	3024	HIST1H1A	
CB_010571	0.001729146	0.00026992	3.948065	up	3627	CXCL10	
CB_009479	0.001725155	0.000269	2.685509	up	284418	FAM71E2	
CB_029680	0.001704232	0.00026465	9.184813	up	347689	SOX2OT	
CB_031938	0.00170267	0.00026438	2.119178	up	100131738	LOC100131738	
CB_010917	0.001701233	0.00026405	2.060848	up	1875	E2F5	
CB_021222	0.00170102	0.00026397	2.491134	up	3238	HOXD12	
CB_027496	0.001700182	0.00026378	2.061884	up	133060	OTOP1	
CB_018637	0.001699543	0.00026336	2.007636	up	24150	TP53TG3	
CB_014496	0.001691075	0.00026154	2.12847	up	6608	SMO	
CB_005623	0.001690378	0.00026124	2.021809	up	2556	GABRA3	
CB_016186	0.001690378	0.00026133	11.44595	up	23650	TRIM29	
CB_030349	0.001688054	0.00026056	4.859521	up	84789	MGC2889	
CB_003713	0.001687964	0.00026052	2.859993	up			
CB_008294	0.001680398	0.00025917	2.070934	up	389207	GRXCR1	
CB_006687	0.001676213	0.00025823	2.739677	up	149699	GTSF1L	
CB_017164	0.001671826	0.00025738	2.047214	up	7775	ZNF232	
CB_015632	0.001670473	0.00025705	14.02373	up	10804	GJB6	
CB_031822	0.001664032	0.0002558	3.957359	up			
CB_012491	0.001662169	0.00025541	7.976815	up	8369	HIST1H4G	
CB_027712	0.001654144	0.00025371	2.69239	up	204219	LASS3	
CB_020051	0.00165117	0.00025288	2.005843	up	55502	HES6	
CB_031591	0.001648687	0.00025224	2.080571	up	100506886	LOC100506886	
CB_024559	0.00164401	0.00025139	2.3956	up	54058	C21orf58	
CB_010961	0.001639733	0.00025066	2.215385	up	2165	F13B	
CB_004713	0.001639007	0.00025041	2.032599	up			
CB_004922	0.001635005	0.00024947	6.481494	up	1277	COL1A1	
CB_004600	0.001633983	0.00024921	2.193855	up			
CB_023755	0.001633393	0.00024907	2.015793	up	84790	TUBA1C	
CB_011126	0.00162221	0.00024676	2.242082	up	3208	HPCA	
CB_006114	0.001616093	0.00024559	2.202074	up	255220	TXNDC8	
CB_030460	0.001614379	0.00024522	2.386078	up	400891	LOC400891	
CB_022066	0.00161398	0.00024509	4.171135	up	79173	C19orf57	
CB_029319	0.001601365	0.00024266	2.468873	up	159125	RBMY2EP	
CB_022214	0.001596692	0.00024149	2.414681	up	84561	SLC12A8	
CB_016462	0.001587309	0.00023973	2.444996	up	23520	ANP32C	
CB_003048	0.001585271	0.0002394	2.155165	up			

CB_020297	0.001583714	0.00023911	12.44867	up	54576	UGT1A8	
CB_028087	0.001574892	0.00023745	5.900098	up	55117	SLC6A15	
CB_028053	0.00156935	0.00023623	2.117363	up	9088	PKMYT1	
CB_003805	0.001553893	0.00023305	2.317741	up			
CB_026372	0.001553049	0.00023288	2.02271	up	221322	C6orf170	
CB_026213	0.001547405	0.00023176	2.026945	up	152065	C3orf22	
CB_014039	0.001547254	0.00023169	2.741782	up	430	ASCL2	
CB_009759	0.001547254	0.00023166	5.007407	up	374897	SBSN	
CB_009956	0.001546827	0.00023155	5.079309	up	57526	PCDH19	
CB_010239	0.001544858	0.00023114	6.518695	up	768	CA9	
CB_014222	0.001540609	0.00023018	3.211451	up	4111	MAGEA12	
CB_031401	0.001540219	0.0002301	2.159611	up	100129786	LOC100129786	
CB_029592	0.00153962	0.00022987	2.434966	up	5820	PVT1	
CB_023581	0.001536897	0.00022914	3.547141	up	84466	MEGF10	
CB_017916	0.001536683	0.00022901	2.041464	up	23321	TRIM2	
CB_011113	0.00153268	0.00022829	2.095943	up	3148	HMGB2	
CB_001760	0.001532583	0.0002282	2.398532	up			
CB_015825	0.001527314	0.00022721	2.773429	up	9506	PAGE4	
CB_028656	0.00152068	0.00022595	2.521483	up	84063	KIRREL2	
CB_020572	0.001517828	0.00022534	3.566146	up	25769	SLC24A2	
CB_004238	0.001505873	0.00022287	2.017287	up			
CB_005651	0.001504327	0.00022259	3.765538	up	2906	GRIN2D	
CB_026197	0.001502754	0.00022231	2.781475	up	150350	ENTHD1	
CB_021484	0.001496438	0.00022107	2.447459	up	112399	EGLN3	
CB_030919	0.001490757	0.00022004	2.785897	up	400619	FLJ26484	
CB_004914	0.001490756	0.00021998	3.873563	up	1029	CDKN2A	
CB_008686	0.001489917	0.00021977	2.01	up	400745	SH2D5	
CB_001163	0.001489917	0.00021975	2.518783	up			
CB_016439	0.001488746	0.00021936	2.019499	up	26689	OR4D1	
CB_029112	0.001488526	0.00021929	2.097768	up	389383	C6orf126	
CB_015755	0.001488526	0.00021924	9.226378	up	6317	SERPINB3	
CB_004098	0.001483903	0.00021837	2.355934	up			
CB_014089	0.001479015	0.00021738	5.611977	up	1750	DLX6	
CB_004924	0.001473656	0.00021616	4.278125	up	1281	COL3A1	
CB_024358	0.001473561	0.00021613	7.160267	up	114771	PGLYRP3	
CB_020509	0.001471325	0.00021568	2.158381	up	10509	SEMA4B	
CB_016729	0.001471325	0.00021565	4.196047	up	2571	GAD1	
CB_002775	0.001469935	0.00021515	2.216752	up			
CB_030833	0.001467926	0.00021474	2.375747	up	63930	LOC63930	
CB_028422	0.001462019	0.00021371	3.259824	up	200844	C3orf67	
CB_013583	0.001462018	0.00021363	2.036868	up	9141	PDCD5	
CB_012821	0.001462018	0.00021366	2.169685	up	8871	SYNJ2	
CB_026981	0.001462018	0.00021361	2.470109	up	134285	TMEM171	
CB_015420	0.001461053	0.00021341	3.064271	up	10669	CGREF1	
CB_001257	0.001458398	0.00021279	4.116816	up	160824	SP3P	
CB_015055	0.001455592	0.0002122	2.984522	up	5270	SERPINE2	
CB_016539	0.0014514	0.00021132	3.101495	up	100133941	CD24	
CB_014828	0.001448047	0.00021059	11.56726	up	6700	SPRR2A	
CB_028609	0.001444365	0.00020986	2.37568	up	284252	KCTD1	
CB_014827	0.001443386	0.00020966	13.25213	up	6700	SPRR2A	
CB_025857	0.001441851	0.0002093	2.154016	up	64843	ISL2	
CB_002069	0.001440369	0.00020895	4.255045	up			
CB_019238	0.001436636	0.00020832	2.290784	up	54820	NDE1	
CB_006268	0.001432612	0.00020762	2.079589	up	126370	OR1I1	
CB_025058	0.001431684	0.0002073	2.18657	up	113115	FAM54A	
CB_022513	0.001431606	0.00020727	2.184707	up	140578	CHODL	

CB_025244	0.001429191	0.00020676	2.056343	up	5068	REG3A	
CB_007266	0.001426654	0.00020624	2.262353	up	100129271	C1orf68	
CB_000333	0.001422017	0.00020523	2.638715	up	123876	ACSM2A	
CB_001511	0.001418604	0.0002044	2.082607	up			
CB_026294	0.00141587	0.00020365	4.66464	up	169166	SNX31	
CB_024003	0.001409898	0.00020254	3.012981	up	58538	MPP4	
CB_016352	0.001409851	0.00020249	2.530516	up	26525	IL1F5	
CB_003481	0.001408864	0.00020224	3.677893	up			
CB_005859	0.001397334	0.00019998	2.957048	up	49861	CLDN20	
CB_029385	0.001389346	0.00019844	2.773373	up	245935	DEFB122	
CB_027952	0.001386984	0.00019805	2.024585	up	148206	ZNF714	
CB_028603	0.001382406	0.00019721	7.831069	up	5744	PTHLH	
CB_012007	0.001380225	0.0001967	3.258913	up	6447	SCG5	
CB_005586	0.001351122	0.00019127	2.978968	up	1562	CYP2C18	
CB_010121	0.001350032	0.00019095	2.032171	up	130574	LYPD6	
CB_003340	0.001339662	0.00018881	2.613567	up			
CB_023412	0.001334141	0.00018771	2.455221	up	84197	SGK196	
CB_000283	0.001334141	0.00018775	2.893038	up	148638	LOC148638	
CB_022459	0.001319484	0.00018512	2.04484	up	79944	L2HGDH	
CB_020026	0.001316477	0.00018459	2.384738	up	53353	LRP1B	
CB_018054	0.001316427	0.00018452	3.150572	up	25878	MXRA5	
CB_024754	0.001314609	0.00018418	3.320163	up	8840	WISP1	
CB_022868	0.001313818	0.00018401	2.237543	up	81569	ACTL8	
CB_028016	0.001310311	0.00018339	12.3585	up	339967	TMPRSS11A	
CB_013381	0.001306098	0.00018249	2.180978	up	3217	HOXB7	
CB_011230	0.001301152	0.00018162	2.042516	up	3780	KCNN1	
CB_005560	0.001294187	0.0001804	2.077542	up	1132	CHRM4	
CB_011395	0.001294187	0.00018038	6.547558	up	4312	MMP1	
CB_027980	0.00129239	0.00018001	2.842527	up	222008	VSTM2A	
CB_027277	0.001284733	0.00017849	6.124636	up	317754	POTED	
CB_017290	0.001283153	0.00017815	7.622722	up	9699	RIMS2	
CB_011732	0.001281347	0.00017768	3.977604	up	5644	PRSS1	
CB_006007	0.001276699	0.00017687	2.031197	up	51155	HN1	
CB_028086	0.001275813	0.00017671	4.982439	up	55117	SLC6A15	
CB_008328	0.001274427	0.00017645	2.465906	up	441161	OOEP	
CB_021929	0.00127323	0.00017613	2.204406	up	66004	LYNX1	
CB_018604	0.00127323	0.00017617	2.60127	up	3196	TLX2	
CB_028438	0.001271178	0.00017574	2.543943	up	284948	SH2D6	
CB_006660	0.001269028	0.00017528	2.052021	up	22859	LPHN1	
CB_029079	0.001264827	0.00017462	3.475877	up	346288	14-Sep	
CB_013043	0.001264797	0.0001746	2.223574	up	4998	ORC1	
CB_020428	0.001264018	0.0001744	2.03821	up	7286	TUFT1	
CB_026345	0.001261248	0.00017385	7.463574	up	204962	SLC44A5	
CB_026664	0.001252712	0.00017214	4.753656	up	266740	MAGEA2B	
CB_021405	0.001247829	0.00017135	2.626435	up	2792	GNGT1	
CB_005243	0.001240594	0.00017012	3.749668	up	1290	COL5A2	
CB_014421	0.001236145	0.00016937	7.112713	up	3868	KRT16	
CB_010909	0.001230601	0.0001683	2.788956	up	1829	DSG2	
CB_022462	0.001217936	0.00016564	2.506194	up	54457	TAF7L	
CB_022095	0.00121789	0.00016558	8.951427	up	1825	DSC3	
CB_024694	0.001217623	0.00016552	2.135647	up	140732	SUN5	
CB_008082	0.001216232	0.00016527	2.029783	up	440145	MZT1	
CB_004495	0.001209434	0.00016404	2.391627	up			
CB_023828	0.001208574	0.00016387	2.660082	up	84904	C9orf100	
CB_030412	0.001207921	0.00016376	2.336982	up	284836	C21orf125	
CB_023681	0.001203757	0.00016278	3.30419	up	84659	RNASE7	

CB_021404	0.001202864	0.00016258	4.23492	up	2700	GJA3	
CB_007137	0.001202644	0.00016253	6.06735	up	92196	DAPL1	
CB_013141	0.00120202	0.00016241	6.244227	up	9407	TMPRSS11D	
CB_013480	0.001197368	0.00016163	3.452068	up	6695	SPOCK1	
CB_010001	0.001189567	0.00016029	2.177452	up	2668	GDNF	
CB_032009	0.001188687	0.00016004	2.390629	up	100506930	LOC100506930	
CB_029098	0.001188015	0.00015993	6.088369	up	388533	KRTDAP	
CB_030003	0.001187797	0.00015988	2.06376	up	727677	LOC727677	
CB_027691	0.001185631	0.00015946	4.169807	up	126433	FBXO27	
CB_011345	0.001185248	0.00015935	5.572053	up	4103	MAGEA4	
CB_026013	0.001185112	0.0001593	2.786211	up	121273	C12orf54	
CB_015483	0.001183466	0.00015904	2.862462	up	26227	PHGDH	
CB_023012	0.001178506	0.00015805	2.979999	up	56154	TEX15	
CB_015398	0.00117654	0.00015766	2.553417	up	10642	IGF2BP1	
CB_011054	0.001174089	0.00015726	9.557288	up	2877	GPX2	
CB_028594	0.001173666	0.00015718	3.146581	up	26664	OR7C1	
CB_001028	0.001162716	0.00015512	2.769971	up	85455	DISP2	
CB_013846	0.00116162	0.00015492	6.648711	up	3381	IBSP	
CB_023156	0.001161517	0.00015489	2.35979	up	3743	KCNA7	
CB_004082	0.001159423	0.0001545	2.246201	up			
CB_021090	0.001158051	0.00015425	2.911811	up	4104	MAGEA5	
CB_012025	0.001151368	0.00015319	2.961938	up	6518	SLC2A5	
CB_010879	0.001149825	0.00015289	2.374808	up	1503	CTPS	
CB_000849	0.001142042	0.00015162	2.040224	up			
CB_009524	0.001141966	0.00015158	2.754628	up	728116	ZBTB8B	
CB_004046	0.001126899	0.00014913	2.029446	up			
CB_010745	0.001124215	0.00014863	2.263801	up	908	CCT6A	
CB_026534	0.001118071	0.00014747	2.468421	up	124602	KIF19	
CB_001749	0.001117267	0.00014733	4.360814	up			
CB_008181	0.001116035	0.000147	2.795845	up	2118	ETV4	
CB_026541	0.001113732	0.00014661	2.710459	up	128272	ARHGEF19	
CB_007737	0.001106842	0.00014532	2.221495	up	399473	SPRED3	
CB_010988	0.001104624	0.00014497	2.094797	up	2312	FLG	
CB_014994	0.001104624	0.00014498	2.408121	up	4761	NEUROD2	
CB_028419	0.001101764	0.00014437	9.782871	up	163351	GBP6	
CB_016642	0.001092807	0.00014296	2.795711	up	29923	C7orf68	
CB_000813	0.001082148	0.00014115	2.10756	up	100130372	LOC100130372	
CB_009495	0.001081859	0.00014108	2.051267	up	360030	NANOGNB	
CB_025131	0.001072208	0.0001395	3.791765	up	154150	HDGFL1	
CB_023265	0.001071924	0.00013944	2.111975	up	83959	SLC4A11	
CB_026323	0.00107172	0.00013936	2.535241	up	55103	RALGPS2	
CB_027016	0.001067162	0.00013847	7.17289	up	163589	TDRD5	
CB_014919	0.001064402	0.00013794	2.038415	up	10344	CCL26	
CB_015542	0.00105259	0.00013589	2.909599	up	10877	CFHR4	
CB_025389	0.001051506	0.00013566	2.199722	up	90861	HN1L	
CB_003644	0.001051068	0.00013556	2.686802	up			
CB_010028	0.001046519	0.00013486	3.850845	up	150946	FAM59B	
CB_003853	0.001045259	0.00013463	2.829943	up			
CB_013490	0.0010375	0.00013345	2.231754	up	6953	TCP10	
CB_031699	0.001034176	0.00013283	2.263846	up	100510377	LOC100510377	
CB_012084	0.001027894	0.00013183	11.77397	up	6657	SOX2	
CB_001854	0.001023958	0.00013101	2.071144	up			
CB_004023	0.001020333	0.00013035	2.348277	up			
CB_022772	0.001018566	0.00013	2.053392	up	89874	SLC25A21	
CB_008308	0.001016221	0.00012952	3.0066	up	390874	ONECUT3	
CB_030102	0.001013821	0.00012905	10.33348	up	646813	LOC646813	

CB_025812	0.001009333	0.00012828	3.642971	up	149708	WFDC5	
CB_029968	0.001008245	0.00012806	2.024537	up	339674	LOC339674	
CB_026920	0.001004978	0.00012743	19.43488	up	286887	KRT6C	
CB_025266	0.000996692	0.00012601	2.436214	up	133396	IL31RA	
CB_020781	0.000995626	0.00012582	2.16821	up	57464	FAM40B	
CB_005320	0.000995604	0.0001258	4.998002	up	7291	TWIST1	
CB_001447	0.000994214	0.00012552	2.367412	up	100170224	SNAR-B1	
CB_029820	0.000992902	0.00012534	2.65997	up	100132708	C2orf14	
CB_008363	0.000992299	0.00012517	8.080276	up	441282	AKR1B15	
CB_021554	0.000988614	0.00012458	2.16641	up	64100	ELSPBP1	
CB_006198	0.000986269	0.00012421	2.225487	up	22801	ITGA11	
CB_009554	0.000984477	0.00012387	8.423175	up	255928	SYT14	
CB_001843	0.000983664	0.00012371	3.070978	up			
CB_001029	0.000981221	0.00012309	2.078585	up	85455	DISP2	
CB_004776	0.000979824	0.00012284	2.58951	up			
CB_006433	0.000968833	0.00012112	2.108466	up	26533	OR10G3	
CB_008488	0.000968686	0.00012108	2.496643	up	202915	TMEM184A	
CB_015430	0.000968163	0.000121	3.001468	up	10686	CLDN16	
CB_019754	0.000964039	0.00012017	2.28499	up	55224	ETNK2	
CB_002749	0.000962783	0.00011989	2.116479	up			
CB_014986	0.000955415	0.00011876	2.148405	up	4007	PRICKLE3	
CB_029362	0.000954332	0.00011852	2.99233	up	348825	TPRXL	
CB_023843	0.000946933	0.00011739	2.350478	up	84915	C12orf34	
CB_015906	0.000943861	0.00011692	2.393857	up	6941	TCF19	
CB_007545	0.000941049	0.0001165	2.143891	up	4839	NOP2	
CB_009242	0.000939903	0.00011629	2.308775	up	26271	FBXO5	
CB_003000	0.000939622	0.00011624	3.118737	up			
CB_007730	0.000939395	0.00011619	5.261442	up	139728	PNCK	
CB_029067	0.000930591	0.00011463	4.20944	up	339010	POTEB	
CB_004069	0.00092885	0.00011437	2.090443	up			
CB_016357	0.000926902	0.00011404	2.072596	up	3751	KCND2	
CB_025909	0.000925031	0.00011375	2.707799	up	59341	TRPV4	
CB_028103	0.000922323	0.00011337	2.125509	up	9071	CLDN10	
CB_016211	0.000921229	0.00011317	4.837358	up	22802	CLCA4	
CB_022449	0.000918772	0.00011281	2.559386	up	79929	MAP6D1	
CB_003627	0.000918679	0.00011278	2.16952	up			
CB_001456	0.000918502	0.00011274	2.034991	up	267020	ATP5L2	
CB_012998	0.00091845	0.00011272	2.156521	up	2016	EMX1	
CB_023367	0.000913327	0.00011179	2.182698	up	84140	FAM161A	
CB_024415	0.000913327	0.00011178	5.46502	up	115701	ALPK2	
CB_003389	0.000913063	0.00011171	2.968153	up	728715	LOC728715	
CB_005268	0.000910044	0.00011119	2.202237	up	3375	IAPP	
CB_010854	0.000908093	0.00011082	3.764321	up	1382	CRABP2	
CB_020662	0.000903628	0.00011009	2.731493	up	5738	PTGFRN	
CB_004782	0.000902974	0.00010998	2.268142	up			
CB_014056	0.00090276	0.00010994	2.621223	up	84733	CBX2	
CB_019939	0.000900349	0.00010949	2.15746	up	55353	LAPTM4B	
CB_007965	0.000898592	0.00010907	2.491434	up	81624	DIAPH3	
CB_031825	0.000895565	0.00010862	2.652428	up			
CB_030378	0.000892573	0.0001081	2.05503	up	150197	LOC150197	
CB_007956	0.000889692	0.00010755	3.022852	up	122786	FRMD6	
CB_008021	0.00088804	0.0001073	2.163695	up	6531	SLC6A3	
CB_019265	0.00088372	0.00010662	2.575933	up	54845	ESRP1	
CB_014418	0.000874043	0.00010506	21.21078	up	3853	KRT6A	
CB_016870	0.00086865	0.00010406	3.704801	up	6334	SCN8A	
CB_027130	0.000868508	0.00010403	3.40991	up	285973	ATG9B	

CB_030892	0.000866733	0.00010376	3.133956	up	441355	FLJ39080	
CB_026211	0.000861643	0.00010291	2.285294	up	152002	C3orf21	
CB_011949	0.000857904	0.00010235	11.87879	up	6318	SERPINB4	
CB_000302	0.000857741	0.00010227	2.330125	up	6652	SORD	
CB_006416	0.000853826	0.00010164	3.260957	up	205	AK4	
CB_005274	0.000851155	0.00010115	2.416656	up	3792	KEL	
CB_010439	0.000851155	0.00010116	3.699829	up	2019	EN1	
CB_010966	0.000846183	0.00010039	6.006647	up	2201	FBN2	
CB_005362	0.000845736	0.00010029	2.57604	up	2524	FUT2	
CB_016832	0.00084558	0.00010025	7.983875	up	26047	CNTNAP2	
CB_001631	0.000844164	0.00010003	3.803429	up	122589	RPLP0P3	
CB_031858	0.000842699	9.9807E-05	3.915482	up			
CB_027483	0.000842449	9.9738E-05	4.668083	up	130576	LYPD6B	
CB_029780	0.000840633	9.944E-05	3.318677	up	285987	DLX6-AS1	
CB_007766	0.000840597	9.9423E-05	2.451059	up	400954	EML6	
CB_031296	0.000833298	9.8246E-05	4.017352	up	3755	KCNG1	
CB_005013	0.000831799	9.7974E-05	2.000328	up	2821	GPI	
CB_020618	0.000830371	9.7766E-05	4.211587	up	57116	ZNF695	
CB_027559	0.000825182	9.705E-05	3.065294	up	347741	OTOP3	
CB_002015	0.000817903	9.5922E-05	2.224675	up			
CB_032017	0.000813151	9.5184E-05	2.136478	up	79160	MGC4294	
CB_029579	0.0008123	9.5048E-05	3.978168	up	64396	GMCL1L	
CB_014588	0.000799224	9.3174E-05	2.641001	up	10105	PPIF	
CB_009301	0.000798511	9.3053E-05	2.511338	up	50507	NOX4	
CB_002250	0.000798383	9.3012E-05	4.665882	up	65268	WNK2	
CB_022141	0.000798015	9.2957E-05	2.604516	up	79605	PGBD5	
CB_015335	0.000795539	9.2604E-05	3.303857	up	10631	POSTN	
CB_006841	0.000795186	9.2527E-05	3.931972	up	4109	MAGEA10	
CB_021232	0.000789857	9.1744E-05	2.077362	up	58477	SRPRB	
CB_019555	0.000783474	9.0732E-05	2.85102	up	55083	KIF26B	
CB_027746	0.000783474	9.0766E-05	3.586522	up	10242	KCNMB2	
CB_006258	0.000780525	9.0326E-05	2.089757	up	219428	OR4C16	
CB_004034	0.00077625	8.9708E-05	2.235576	up	80174	DBF4B	
CB_020657	0.000775687	8.9594E-05	2.658751	up	57167	SALL4	
CB_001753	0.000771802	8.9023E-05	3.150244	up	285629	LOC285629	
CB_013952	0.000771456	8.8946E-05	2.395323	up	6508	SLC4A3	
CB_025451	0.000765099	8.8019E-05	2.017376	up	130827	TMEM182	
CB_025817	0.000764135	8.7823E-05	2.033644	up	80174	DBF4B	
CB_003008	0.000763958	8.7767E-05	2.470761	up			
CB_008084	0.000762925	8.76E-05	11.54609	up	54578	UGT1A6	
CB_003989	0.000762639	8.753E-05	2.524912	up			
CB_010418	0.000762639	8.7526E-05	3.032324	up	1952	CELSR2	
CB_011734	0.000757604	8.6736E-05	2.738497	up	5646	PRSS3	
CB_024906	0.000756572	8.657E-05	3.780254	up	170961	ANKRD24	
CB_000361	0.000756366	8.6511E-05	2.75985	up	283028	LOC283028	
CB_008606	0.000756181	8.6466E-05	2.644299	up	139604	MAGEB16	
CB_001839	0.0007553	8.6338E-05	2.102934	up	728361	OVOL3	
CB_002280	0.00074895	8.5354E-05	2.38733	up			
CB_015539	0.000743584	8.4589E-05	6.074028	up	10874	NMU	
CB_014061	0.000733493	8.3208E-05	2.263205	up	1046	CDX4	
CB_023095	0.000732166	8.3012E-05	2.426133	up	83639	TEX101	
CB_008083	0.000729415	8.2526E-05	16.97843	up	54578	UGT1A6	
CB_020543	0.000729018	8.2435E-05	18.9918	up	57016	AKR1B10	
CB_031819	0.000727627	8.2243E-05	2.62163	up			
CB_000250	0.000727494	8.2217E-05	2.520562	up			
CB_012633	0.000727007	8.2139E-05	14.37974	up	8581	LY6D	

CB_007089	0.0007265	8.2035E-05	2.215911	up	388581	FAM132A	
CB_000496	0.000725255	8.186E-05	2.080349	up	647662	FLJ40039	
CB_029367	0.000718684	8.0902E-05	2.05805	up	55545	MSX2P1	
CB_017064	0.000717988	8.0789E-05	2.201477	up	27076	LYPD3	
CB_014016	0.00071759	8.0732E-05	3.163094	up	55806	HR	
CB_012032	0.000716244	8.0514E-05	2.174732	up	6541	SLC7A1	
CB_005992	0.000714206	8.0239E-05	3.333408	up	513	ATP5D	
CB_006006	0.00071372	8.014E-05	2.075903	up	51155	HN1	
CB_021128	0.000711979	7.9865E-05	2.133604	up	8884	SLC5A6	
CB_026752	0.00071171	7.9801E-05	2.606405	up	221393	GPR115	
CB_019109	0.000711462	7.9751E-05	3.139544	up	54742	LY6K	
CB_001419	0.000710947	7.9637E-05	2.32547	up			
CB_030174	0.000710837	7.9602E-05	2.379314	up	54677	CROT	
CB_030757	0.000708317	7.9218E-05	2.101648	up	400624	FLJ45079	
CB_005729	0.000706727	7.8873E-05	2.283857	up	4985	OPRD1	
CB_031752	0.000703997	7.8512E-05	2.121018	up			
CB_032084	0.000703213	7.8369E-05	7.509705	up	728755	LOC728755	
CB_005218	0.000700515	7.7944E-05	2.162597	up	7167	TPI1	
CB_021185	0.000697059	7.7424E-05	2.241163	up	23426	GRIP1	
CB_001773	0.00069628	7.732E-05	7.327786	up			
CB_020377	0.00069445	7.7018E-05	2.534553	up	56479	KCNQ5	
CB_022571	0.000690568	7.6478E-05	2.149792	up	80128	TRIM46	
CB_021348	0.000686949	7.5936E-05	2.116847	up	50614	GALNT9	
CB_026775	0.000678576	7.4601E-05	2.626673	up	255061	TAC4	
CB_026018	0.000675239	7.4127E-05	2.124403	up	121793	C13orf16	
CB_011733	0.000668643	7.3022E-05	2.515416	up	5645	PRSS2	
CB_029278	0.000666727	7.2728E-05	2.066515	up	339240	LOC339240	
CB_010659	0.000661308	7.1906E-05	2.134602	up	440	ASNS	
CB_030073	0.000659572	7.1665E-05	2.829271	up	100131726	LOC100131726	
CB_012461	0.000658105	7.1422E-05	3.908282	up	8344	HIST1H2BE	
CB_010460	0.000655309	7.0994E-05	5.467364	up	2196	FAT2	
CB_014423	0.00064965	7.0165E-05	3.002736	up	284217	LAMA1	
CB_010317	0.000648412	6.9949E-05	2.143981	up	1325	CORT	
CB_014266	0.000645229	6.9503E-05	2.614152	up	6496	SIX3	
CB_030847	0.000640375	6.8828E-05	9.142289	up	284116	KRT42P	
CB_011388	0.000639527	6.8692E-05	2.110675	up	4282	MIF	
CB_019243	0.000637066	6.8331E-05	4.88559	up	55612	FERMT1	
CB_021265	0.000635315	6.8093E-05	2.137528	up	58516	FAM60A	
CB_030673	0.00062816	6.7087E-05	3.822618	up	440173	LOC440173	
CB_016952	0.000620515	6.5966E-05	2.821979	up	23413	NCS1	
CB_014572	0.000616875	6.5444E-05	2.26101	up	10083	USH1C	
CB_030786	0.000615065	6.5142E-05	2.142339	up	401898	ZNF833P	
CB_029169	0.000612696	6.4765E-05	2.26891	up	388962	BOLA3	
CB_001938	0.000612696	6.4758E-05	2.495214	up			
CB_008209	0.000605592	6.3766E-05	2.056109	up	729330	OC90	
CB_014017	0.000602147	6.3287E-05	2.768283	up	55806	HR	
CB_025071	0.000598881	6.2848E-05	3.380733	up	113730	KLHDC7B	
CB_019793	0.000597469	6.2606E-05	3.416473	up	55247	NEIL3	
CB_005266	0.00058438	6.0752E-05	2.084314	up	3292	HSD17B1	
CB_019938	0.00058002	6.0153E-05	2.632561	up	55353	LAPTM4B	
CB_002059	0.000579121	6.002E-05	10.88616	up			
CB_020616	0.000576796	5.9709E-05	7.038368	up	57115	PGLYRP4	
CB_011196	0.000573628	5.929E-05	3.923	up	3696	ITGB8	
CB_017642	0.000572077	5.903E-05	2.082392	up	22995	CEP152	
CB_011364	0.000570348	5.8805E-05	2.091538	up	4172	MCM3	
CB_010009	0.000566744	5.8309E-05	2.252309	up	256223	CDRT15L2	

CB_014983	0.000557775	5.7041E-05	4.238287	up	3664	IRF6	
CB_001719	0.000557755	5.7029E-05	2.047259	up			
CB_008433	0.000557722	5.6993E-05	2.010406	up	284403	WDR62	
CB_019846	0.000556623	5.68E-05	3.614275	up	55287	TMEM40	
CB_002022	0.000555727	5.664E-05	2.115183	up			
CB_030813	0.000555509	5.6592E-05	2.126829	up	440910	LOC440910	
CB_025437	0.000555105	5.6517E-05	2.30836	up	125965	COX6B2	
CB_017016	0.000555105	5.6528E-05	3.159961	up	25830	SULT4A1	
CB_009828	0.000550697	5.5933E-05	5.097984	up	285782	CAGE1	
CB_015677	0.000548749	5.5683E-05	2.195177	up	10970	CKAP4	
CB_003629	0.000545928	5.5345E-05	2.214596	up			
CB_030506	0.000542229	5.4824E-05	2.486708	up	100132215	LOC100132215	
CB_019242	0.000537982	5.4224E-05	6.393302	up	55612	FERMT1	
CB_022907	0.000536512	5.405E-05	3.245684	up	81607	PVRL4	
CB_023973	0.000535607	5.3899E-05	2.18553	up	66037	BOLL	
CB_007045	0.000531331	5.3296E-05	2.658847	up	55620	STAP2	
CB_012576	0.000529592	5.3093E-05	2.501154	up	8508	NIPSNAP1	
CB_000143	0.000526025	5.2635E-05	2.304689	up			
CB_032000	0.000524894	5.248E-05	2.384182	up	100506676	LOC100506676	
CB_018821	0.000523746	5.2332E-05	2.679779	up	51514	DTL	
CB_006287	0.000523261	5.2276E-05	2.555069	up	219437	OR5L1	
CB_013535	0.00052035	5.1894E-05	2.208024	up	8437	RASAL1	
CB_015889	0.000510756	5.0646E-05	7.744559	up	11166	SOX21	
CB_007829	0.00051	5.053E-05	7.797794	up	165679	C3orf57	
CB_010775	0.00050928	5.0435E-05	4.972289	up	1001	CDH3	
CB_006968	0.000508937	5.0385E-05	2.306094	up	388753	C1orf31	
CB_010071	0.000508764	5.0343E-05	2.080594	up	100506581	LOC100506581	
CB_007753	0.000507352	5.0131E-05	2.157118	up	55898	UNC45A	
CB_013339	0.000506784	5.0051E-05	3.070964	up	2191	FAP	
CB_012855	0.000505818	4.9895E-05	2.533111	up	8936	WASF1	
CB_003595	0.000501054	4.9302E-05	3.452936	up			
CB_022237	0.000494267	4.8407E-05	2.13525	up	79703	C11orf80	
CB_027111	0.000493901	4.8356E-05	2.600822	up	401024	FSIP2	
CB_002317	0.000491597	4.8084E-05	3.569404	up			
CB_015510	0.000491065	4.7991E-05	6.778997	up	65268	WNK2	
CB_025700	0.00049069	4.7917E-05	3.24614	up	151354	FAM84A	
CB_011132	0.000485573	4.7225E-05	2.555183	up	3236	HOXD10	
CB_003592	0.000485573	4.7209E-05	3.000222	up			
CB_009571	0.000480384	4.6553E-05	2.040011	up	100130932	LOC100130932	
CB_020018	0.000476436	4.6045E-05	2.521624	up	55366	LGR4	
CB_022955	0.000476436	4.6045E-05	2.657589	up	81796	SLCO5A1	
CB_006189	0.000476436	4.6049E-05	3.164249	up	441478	NRARP	
CB_002189	0.000475302	4.5879E-05	2.775044	up			
CB_020165	0.000472859	4.5593E-05	2.017991	up	54205	CYCS	
CB_026539	0.000471623	4.5449E-05	5.07814	up	127534	GJB4	
CB_006180	0.000471129	4.5365E-05	3.720187	up	440590	ZYG11A	
CB_003736	0.000467418	4.4903E-05	12.05611	up			
CB_016799	0.000467062	4.4854E-05	2.154733	up	28978	TMEM14A	
CB_013478	0.000465153	4.4612E-05	2.057889	up	6611	SMS	
CB_021769	0.000464094	4.4488E-05	2.191249	up	64785	GINS3	
CB_015768	0.000464094	4.4442E-05	2.484386	up	6613	SUMO2	
CB_002137	0.000464094	4.4467E-05	4.217497	up	171484	FAM9C	
CB_007601	0.000464094	4.4474E-05	4.869641	up	55061	SUSD4	
CB_025251	0.000451947	4.293E-05	5.579784	up	81832	NETO1	
CB_018053	0.000451083	4.2819E-05	4.035388	up	25878	MXRA5	
CB_016803	0.00045031	4.2689E-05	10.31716	up	28983	TMPRSS11E	

CB_007191	0.000446023	4.2099E-05	3.11147	up	121551	BTBD11	
CB_005144	0.000445598	4.2051E-05	13.63163	up	5317	PKP1	
CB_008039	0.00044559	4.2044E-05	2.39187	up	6715	SRD5A1	
CB_023200	0.000441736	4.1547E-05	2.893682	up	83873	GPR61	
CB_030708	0.000440864	4.1458E-05	3.109541	up	646300	COL6A4P2	
CB_027459	0.000440777	4.1443E-05	2.202991	up	165215	FAM171B	
CB_021531	0.000439714	4.1322E-05	4.441239	up	64065	PERP	
CB_005597	0.000438502	4.118E-05	8.408896	up	1591	CYP24A1	
CB_010404	0.0004382	4.1145E-05	2.026184	up	1852	DUSP9	
CB_004777	0.000437579	4.1064E-05	2.047516	up			
CB_005543	0.000435055	4.0746E-05	6.029836	up	774	CACNA1B	
CB_022916	0.000434716	4.0707E-05	2.019912	up	81616	ACSBG2	
CB_019702	0.000433358	4.0553E-05	2.088923	up	55726	C12orf11	
CB_024363	0.000431914	4.0397E-05	2.602809	up	114784	CSMD2	
CB_005262	0.000431216	4.0305E-05	2.915989	up	2978	GUCA1A	
CB_015279	0.000430043	4.0161E-05	17.56415	up	10563	CXCL13	
CB_019701	0.000429317	4.0066E-05	2.172619	up	55726	C12orf11	
CB_011616	0.000427256	3.9792E-05	17.15952	up	5268	SERPINB5	
CB_000369	0.000426621	3.9679E-05	2.652225	up			
CB_015948	0.000421087	3.8991E-05	3.282936	up	7784	ZP3	
CB_016455	0.000421008	3.8977E-05	5.647035	up	5275	SERPINB13	
CB_001418	0.00041434	3.8241E-05	2.19847	up	100129461	LOC100129461	
CB_020573	0.000414005	3.8204E-05	3.521322	up	25769	SLC24A2	
CB_011237	0.000413253	3.8062E-05	2.038331	up	3797	KIF3C	
CB_019246	0.000411914	3.7887E-05	2.431352	up	54825	CDHR2	
CB_008583	0.000409464	3.7603E-05	2.616238	up	200916	RPL22L1	
CB_022088	0.000407699	3.7383E-05	2.399613	up	4880	NPPC	
CB_016766	0.00040666	3.7229E-05	2.08444	up	50943	FOXP3	
CB_008676	0.000405534	3.7049E-05	2.146809	up	125931	CEACAM20	
CB_007823	0.000400118	3.6327E-05	8.875866	up	6696	SPP1	
CB_029902	0.000396153	3.584E-05	3.327138	up	79100	MGC4473	
CB_013482	0.000396101	3.5823E-05	2.602381	up	6804	STX1A	
CB_021445	0.000395486	3.5714E-05	2.34425	up	7552	ZNF711	
CB_009971	0.00039415	3.5547E-05	2.35165	up	2902	GRIN1	
CB_019048	0.000393078	3.5438E-05	2.093362	up	51805	COQ3	
CB_003601	0.000392339	3.5327E-05	2.51081	up			
CB_014566	0.000391766	3.5251E-05	2.128171	up	10078	TSSC4	
CB_004033	0.000388648	3.4841E-05	4.770027	up			
CB_020164	0.000387561	3.4707E-05	2.050752	up	54205	CYCS	
CB_007921	0.000387051	3.4609E-05	2.271971	up	723790	HIST2H2AA4	
CB_010485	0.00038594	3.4488E-05	3.012763	up	2583	B4GALNT1	
CB_000196	0.000385751	3.4465E-05	2.58572	up	80078	FLJ13744	
CB_008717	0.000385329	3.4415E-05	2.453878	up	282973	JAKMIP3	
CB_011782	0.000385246	3.4392E-05	2.002446	up	5756	TWF1	
CB_001847	0.00037969	3.3725E-05	2.475141	up			
CB_014818	0.000378306	3.359E-05	12.92912	up	6273	S100A2	
CB_022496	0.000377925	3.3538E-05	5.156399	up	79983	POF1B	
CB_005145	0.00037608	3.3299E-05	13.6731	up	5317	PKP1	
CB_010698	0.000375983	3.3276E-05	4.493168	up	655	BMP7	
CB_028088	0.000375319	3.3176E-05	2.057241	up	4176	MCM7	
CB_028974	0.000374855	3.3117E-05	8.53017	up	23532	PRAME	
CB_012856	0.000373826	3.2967E-05	2.962699	up	8936	WASF1	
CB_005379	0.000373688	3.2937E-05	14.95129	up	3861	KRT14	
CB_008028	0.000372451	3.2781E-05	2.300294	up	65062	ALS2CR4	
CB_005762	0.000371216	3.2631E-05	2.021015	up	5558	PRIM2	
CB_009812	0.000370333	3.253E-05	3.16103	up	401097	LOC401097	

CB_032158	0.000368978	3.2387E-05	2.402374	up	400706	LOC400706	
CB_021298	0.00036721	3.218E-05	4.396404	up	59336	PRDM13	
CB_022533	0.000361929	3.1614E-05	3.102665	up	80032	ZNF556	
CB_021466	0.000361591	3.1559E-05	2.097915	up	56660	KCNK12	
CB_010375	0.000359584	3.1335E-05	2.148324	up	1740	DLG2	
CB_010983	0.000359366	3.1309E-05	2.01512	up	2288	FKBP4	
CB_017858	0.000358737	3.1184E-05	2.662708	up	23251	KIAA1024	
CB_004868	0.000354553	3.0745E-05	2.167598	up	335	APOA1	
CB_000801	0.000351908	3.0409E-05	2.763635	up	100128851	LOC100128851	
CB_004493	0.000350151	3.0213E-05	2.271984	up			
CB_016103	0.000349366	3.0107E-05	2.888742	up	8989	TRPA1	
CB_010910	0.00034666	2.9788E-05	23.05961	up	1830	DSG3	
CB_014980	0.000346242	2.9733E-05	5.209648	up	2842	GPR19	
CB_011661	0.000344959	2.9596E-05	2.302394	up	5453	POU3F1	
CB_002559	0.000342833	2.9337E-05	3.688142	up			
CB_026136	0.000341001	2.911E-05	2.704028	up	1663	DDX11	
CB_023456	0.000340767	2.9078E-05	2.073402	up	84262	PSMG3	
CB_014295	0.000340207	2.8972E-05	3.130776	up	8208	CHAF1B	
CB_017737	0.000338694	2.8806E-05	2.199297	up	23109	DDN	
CB_023330	0.00033799	2.8724E-05	3.619413	up	84077	C3orf20	
CB_002599	0.000336444	2.8536E-05	3.060831	up			
CB_002456	0.000330426	2.7883E-05	2.248918	up			
CB_026418	0.000330316	2.7858E-05	7.787693	up	256714	MAP7D2	
CB_007519	0.000328988	2.7708E-05	3.498815	up	4610	MYCL1	
CB_019037	0.000328619	2.7668E-05	5.27918	up	3226	HOXC10	
CB_025967	0.000327675	2.7541E-05	2.167795	up	91442	C19orf40	
CB_012468	0.000324491	2.7171E-05	2.206699	up	8350	HIST1H3A	
CB_022912	0.000324051	2.7129E-05	2.013318	up	81611	ANP32E	
CB_016040	0.000320411	2.6728E-05	4.048485	up	11281	POU6F2	
CB_016876	0.000319024	2.6591E-05	2.136857	up	1834	DSPP	
CB_018343	0.000318645	2.654E-05	4.259769	up	51050	PI15	
CB_009223	0.000318606	2.652E-05	2.495628	up	100128327	BET3L	
CB_025929	0.000318606	2.6519E-05	3.167492	up	121214	SDR9C7	
CB_031352	0.000318108	2.646E-05	37.41779	up			
CB_022077	0.0003169	2.6334E-05	2.347001	up	79187	FSD1	
CB_017019	0.000316799	2.6321E-05	2.73217	up	25837	RAB26	
CB_006935	0.000314776	2.6108E-05	5.890128	up	494514	C18orf56	
CB_005227	0.000311382	2.5723E-05	2.072939	up	7372	UMPS	
CB_022342	0.000306949	2.5223E-05	2.531622	up	79814	AGMAT	
CB_013011	0.000306484	2.5158E-05	4.518994	up	2256	FGF11	
CB_028031	0.000304574	2.4938E-05	3.33465	up	348738	C2orf48	
CB_005089	0.000296722	2.4088E-05	2.301245	up	4436	MSH2	
CB_026196	0.000296479	2.405E-05	2.760316	up	150350	ENTHD1	
CB_023215	0.000296392	2.4033E-05	2.507488	up	83886	PRSS27	
CB_023649	0.000293374	2.3747E-05	4.11973	up	84624	FNDC1	
CB_029781	0.000293101	2.3715E-05	3.976685	up	285548	LOC285548	
CB_016201	0.000290897	2.3509E-05	3.548096	up	23624	CBLC	
CB_001470	0.000290599	2.348E-05	3.268952	up	552860	NCRNA00252	
CB_001152	0.000288436	2.3251E-05	4.591806	up	645158	LOC645158	
CB_021748	0.000286761	2.307E-05	3.90267	up	94081	SFXN1	
CB_027278	0.000284472	2.2814E-05	4.757071	up	317754	POTED	
CB_009601	0.000283031	2.2663E-05	2.463413	up	28969	BZW2	
CB_012195	0.000279275	2.2264E-05	2.111698	up	7036	TFR2	
CB_018351	0.000278156	2.2162E-05	2.591628	up	51053	GMNN	
CB_021444	0.000276952	2.2031E-05	2.449977	up	7552	ZNF711	
CB_008551	0.000276341	2.1978E-05	3.311131	up	729475	RAD51AP2	

CB_015073	0.000276182	2.1961E-05	2.050936	up	5437	POLR2H	
CB_026412	0.000275644	2.19E-05	2.837874	up	255758	TCTEX1D2	
CB_021882	0.000275197	2.1857E-05	2.280057	up	65243	ZNF643	
CB_021284	0.000274555	2.1764E-05	2.537315	up	58985	IL22RA1	
CB_029475	0.000273713	2.1665E-05	2.715146	up	26821	SNORA74A	
CB_016722	0.000272629	2.1548E-05	2.381391	up	29967	LRP12	
CB_017063	0.000269688	2.1244E-05	7.799833	up	27076	LYPD3	
CB_016267	0.000269	2.1138E-05	5.396579	up	25758	C11orf41	
CB_027516	0.000268408	2.1075E-05	5.726461	up	1745	DLX1	
CB_024543	0.000267876	2.1016E-05	2.939217	up	9134	CCNE2	
CB_023906	0.000262676	2.0483E-05	4.366167	up	84985	FAM83A	
CB_003830	0.000261394	2.035E-05	2.727085	up			
CB_000272	0.000261057	2.0317E-05	4.462153	up	150005	LOC150005	
CB_001877	0.000260828	2.0268E-05	2.282576	up			
CB_028331	0.000260828	2.0256E-05	4.196383	up	57822	GRHL3	
CB_006767	0.000259257	2.0114E-05	4.133415	up	222642	TSPO2	
CB_030010	0.000257069	1.9887E-05	2.653462	up	730101	LOC730101	
CB_012643	0.000256846	1.9841E-05	2.206929	up	8607	RUVBL1	
CB_028427	0.000255578	1.9718E-05	2.089835	up	253714	MMS22L	
CB_019755	0.000249681	1.9133E-05	3.250006	up	55224	ETNK2	
CB_020313	0.000249604	1.9123E-05	2.310496	up	54714	CNGB3	
CB_020614	0.000249519	1.9109E-05	3.112875	up	57113	TRPC7	
CB_014437	0.000249351	1.9092E-05	4.108602	up	4001	LMNB1	
CB_023263	0.000248795	1.9032E-05	3.253139	up	83942	TSSK1B	
CB_019020	0.000248264	1.8982E-05	6.878291	up	10736	SIX2	
CB_032087	0.000243116	1.8465E-05	2.875911	up	283553	LOC283553	
CB_014205	0.000243006	1.8452E-05	2.309007	up	3149	HMGB3	
CB_017177	0.000242957	1.8445E-05	4.113097	up	5013	OTX1	
CB_024102	0.00024001	1.8164E-05	2.82572	up	90226	UCN2	
CB_005630	0.000239204	1.8073E-05	4.522342	up	2571	GAD1	
CB_006156	0.00023912	1.8044E-05	4.070187	up	339184	CCDC144NL	
CB_012421	0.000236901	1.7805E-05	2.055317	up	8091	HMGA2	
CB_012481	0.000235036	1.7605E-05	2.114167	up	8360	HIST1H4D	
CB_006415	0.000233107	1.7427E-05	3.491208	up	205	AK4	
CB_003585	0.000231762	1.7286E-05	2.186412	up			
CB_002224	0.000230971	1.7216E-05	7.023584	up			
CB_004188	0.000229855	1.71E-05	2.893938	up			
CB_015313	0.000228506	1.6975E-05	2.036822	up	10609	LEPREL4	
CB_013352	0.000227825	1.6903E-05	9.694449	up	2304	FOXE1	
CB_012064	0.000227009	1.6803E-05	2.585967	up	6624	FSCN1	
CB_022265	0.000226037	1.6705E-05	4.184969	up	79733	E2F8	
CB_030983	0.000225941	1.6684E-05	2.162183	up	440117	LOC440117	
CB_029473	0.000223378	1.6441E-05	7.132667	up	641516	KC6	
CB_027609	0.000222895	1.6399E-05	13.0123	up	128876	FAM83C	
CB_029741	0.000220865	1.6223E-05	3.623684	up	440356	LOC440356	
CB_012484	0.0002172	1.5821E-05	2.702067	up	8362	HIST1H4K	
CB_010918	0.000216793	1.5782E-05	2.43347	up	780	DDR1	
CB_013937	0.000215124	1.5636E-05	2.470833	up	5881	RAC3	
CB_028988	0.00021494	1.5619E-05	16.87153	up	84985	FAM83A	
CB_008265	0.000211796	1.5334E-05	2.031625	up	57673	BEND3	
CB_008417	0.000209521	1.5136E-05	6.653856	up	445582	POTEE	
CB_009789	0.00020864	1.5052E-05	4.355093	up	1136	CHRNA3	
CB_013770	0.000208083	1.5006E-05	9.559887	up	9547	CXCL14	
CB_009144	0.000207329	1.4941E-05	2.416271	up	5349	FXYD3	
CB_027787	0.000206889	1.49E-05	2.083388	up	5983	RFC3	
CB_010871	0.000206165	1.4825E-05	13.67302	up	1469	CST1	

CB_023730	0.000205992	1.4809E-05	2.336957	up	6502	SKP2	
CB_008010	0.000204372	1.467E-05	2.908737	up	63979	FIGNL1	
CB_018411	0.00020325	1.4554E-05	2.570513	up	51083	GAL	
CB_023608	0.000202938	1.4512E-05	4.038159	up	84518	CNFN	
CB_004165	0.000201959	1.4417E-05	3.667495	up			
CB_001613	0.000201042	1.4329E-05	4.904689	up	644213	GAPDHP32	
CB_008296	0.000200738	1.4301E-05	3.387501	up	389257	LRRC14B	
CB_020804	0.000200237	1.4253E-05	2.097365	up	57486	NLN	
CB_027620	0.000199804	1.4215E-05	5.432015	up	159963	SLC5A12	
CB_027024	0.000198018	1.4054E-05	2.046177	up	170393	C10orf91	
CB_023827	0.000197098	1.3948E-05	4.338935	up	84904	C9orf100	
CB_012066	0.000196915	1.3926E-05	2.067217	up	6627	SNRPA1	
CB_002313	0.000196322	1.3868E-05	3.959336	up			
CB_012535	0.000195181	1.3763E-05	2.070078	up	8458	TTF2	
CB_024167	0.000195181	1.3757E-05	4.953746	up	4241	MFI2	
CB_005356	0.000195137	1.3741E-05	2.88747	up	2161	F12	
CB_015998	0.000195125	1.3734E-05	2.151069	up	11222	MRPL3	
CB_024368	0.0001943	1.3642E-05	2.352809	up	114787	GPRIN1	
CB_030498	0.000191942	1.3444E-05	3.959232	up	284593	FAM41C	
CB_013439	0.000191862	1.3425E-05	4.085477	up	5017	OVOL1	
CB_026918	0.000189592	1.3206E-05	2.237252	up	286826	LIN9	
CB_021938	0.000187078	1.2987E-05	10.52641	up	2707	GJB3	
CB_010984	0.000186462	1.2938E-05	2.334331	up	2288	FKBP4	
CB_020401	0.000180226	1.2397E-05	14.06825	up	56649	TMPRSS4	
CB_023778	0.000179119	1.2295E-05	3.215618	up	84842	HPDL	
CB_031515	0.000178797	1.2264E-05	2.87823	up	100288902	LOC100288902	
CB_008081	0.000178797	1.2261E-05	2.912851	up	7298	TYMS	
CB_008857	0.000178797	1.2264E-05	2.962287	up	729533	FAM72A	
CB_030704	0.000178294	1.2205E-05	2.111842	up	221718	C6orf218	
CB_022794	0.0001775	1.213E-05	2.945679	up	1663	DDX11	
CB_010559	0.000176977	1.2079E-05	3.156921	up	3547	IGSF1	
CB_020467	0.000175753	1.1945E-05	2.317709	up	56924	PAK6	
CB_014752	0.000175017	1.1883E-05	2.135267	up	4176	MCM7	
CB_009827	0.000174285	1.1814E-05	6.974135	up	115749	C12orf56	
CB_029900	0.000172012	1.1613E-05	2.429823	up	25845	LOC25845	
CB_003566	0.000170194	1.1418E-05	5.960909	up			
CB_021221	0.000165361	1.1028E-05	22.22575	up	3237	HOXD11	
CB_029794	0.000165009	1.099E-05	2.504583	up	100144603	LOC100144603	
CB_024881	0.0001648	1.097E-05	3.623907	up	84706	GPT2	
CB_026898	0.000164347	1.0932E-05	2.865109	up	259307	IL4I1	
CB_022795	0.000164052	1.0907E-05	2.604926	up	1663	DDX11	
CB_015307	0.00016393	1.0896E-05	2.141464	up	10605	PAIP1	
CB_015805	0.000162937	1.0807E-05	2.029249	up	8092	ALX1	
CB_006458	0.000162677	1.078E-05	2.093595	up	441670	OR4M1	
CB_015941	0.000160365	1.058E-05	2.139748	up	7705	ZNF146	
CB_012024	0.000159776	1.0529E-05	2.56175	up	6509	SLC1A4	
CB_019177	0.000159663	1.0515E-05	2.191309	up	29980	DONSON	
CB_024826	0.000159663	1.0519E-05	8.823309	up	131578	LRRC15	
CB_005563	0.000158449	1.0404E-05	3.323822	up	1138	CHRNA5	
CB_030889	0.000157989	1.0366E-05	4.915702	up	100132774	LOC100132774	
CB_027873	0.000156852	1.0277E-05	2.009636	up	160418	TMTC3	
CB_011863	0.000156795	1.0271E-05	2.447793	up	5932	RBBP8	
CB_007069	0.000156435	1.0245E-05	2.067389	up	132299	OCIAD2	
CB_018767	0.000153407	1.0007E-05	2.096416	up	51475	CABP2	
CB_025771	0.000153341	1.0001E-05	2.017832	up	153328	SLC25A48	
CB_027799	0.000152849	9.9636E-06	2.003287	up	337882	KRTAP19-1	

CB_021126	0.00015105	9.8224E-06	2.445897	up	8884	SLC5A6	
CB_015388	0.00015025	9.7466E-06	18.01953	up	9635	CLCA2	
CB_009011	0.00014991	9.7143E-06	2.84857	up	100170841	C17orf96	
CB_002511	0.000148964	9.6419E-06	2.422551	up			
CB_005278	0.000147363	9.5149E-06	25.15807	up	3852	KRT5	
CB_003765	0.000146676	9.4532E-06	2.954344	up			
CB_015463	0.000146555	9.4418E-06	2.140142	up	10744	PTTG2	
CB_018622	0.000146529	9.4378E-06	2.087973	up	9585	KIF20B	
CB_002084	0.00014627	9.4165E-06	3.845532	up			
CB_012208	0.000144772	9.2902E-06	3.353356	up	7058	THBS2	
CB_010328	0.000143073	9.1608E-06	2.01688	up	1434	CSE1L	
CB_016408	0.000142731	9.1276E-06	2.012875	up	23542	MAPK8IP2	
CB_014494	0.000142655	9.1141E-06	5.422735	up	6535	SLC6A8	
CB_010878	0.000141718	9.0381E-06	2.196521	up	1503	CTPS	
CB_008882	0.000139434	8.8615E-06	3.771546	up	7161	TP73	
CB_027891	0.000139081	8.8312E-06	2.365505	up	353500	BMP8A	
CB_014934	0.000139044	8.8125E-06	5.503199	up	10381	TUBB3	
CB_014768	0.000137365	8.6799E-06	3.791688	up	4241	MFI2	
CB_028075	0.000136903	8.642E-06	4.453892	up	55388	MCM10	
CB_016260	0.000136544	8.6064E-06	4.316323	up	26256	CABYR	
CB_015259	0.000135784	8.5396E-06	2.352844	up	10535	RNASEH2A	
CB_014419	0.000131247	8.1727E-06	20.45981	up	3854	KRT6B	
CB_016265	0.000129624	8.0446E-06	3.00243	up	25758	C11orf41	
CB_017821	0.000129624	8.0449E-06	5.083996	up	23213	SULF1	
CB_027492	0.000128382	7.9352E-06	2.373302	up	347688	TUBB8	
CB_008364	0.000128382	7.9351E-06	3.793023	up	284992	CCDC150	
CB_008492	0.000128265	7.9239E-06	2.023657	up	390259	BSX	
CB_027514	0.000128072	7.9079E-06	2.300476	up	86	ACTL6A	
CB_012053	0.000127364	7.8561E-06	2.045967	up	6598	SMARCB1	
CB_005632	0.000127287	7.8433E-06	2.027735	up	2618	GART	
CB_010686	0.000127287	7.8424E-06	2.494633	up	576	BAI2	
CB_000402	0.000126943	7.8145E-06	3.333633	up	284219	LOC284219	
CB_011201	0.000126938	7.8077E-06	2.226106	up	3706	ITPKA	
CB_001259	0.00012638	7.7645E-06	2.443863	up	284889	LOC284889	
CB_026105	0.000125948	7.7247E-06	2.026959	up	136051	ZNF786	
CB_008698	0.000125687	7.7009E-06	15.22245	up	642587	LOC642587	
CB_013224	0.000125025	7.6484E-06	2.078898	up	790	CAD	
CB_020561	0.000125025	7.6477E-06	3.624604	up	57216	VANGL2	
CB_030601	0.000124701	7.6187E-06	3.129507	up	399815	LOC399815	
CB_012450	0.000124671	7.6149E-06	2.259692	up	8334	HIST1H2AC	
CB_008766	0.000124571	7.6049E-06	2.271572	up	222183	SRRM3	
CB_027482	0.000124364	7.5883E-06	2.494321	up	91683	SYT12	
CB_006190	0.00012124	7.3477E-06	2.639339	up	441478	NRARP	
CB_025669	0.000120953	7.3265E-06	3.602668	up	221150	SKA3	
CB_017864	0.000120508	7.2881E-06	4.652849	up	23255	KIAA0802	
CB_021153	0.000119232	7.1982E-06	3.835836	up	5366	PMAIP1	
CB_007095	0.000119232	7.1996E-06	4.715367	up	548596	CKMT1A	
CB_031740	0.000118958	7.1731E-06	2.502965	up			
CB_030101	0.00011786	7.0778E-06	3.88749	up	646813	LOC646813	
CB_021317	0.000117681	7.0611E-06	4.53182	up	8038	ADAM12	
CB_005232	0.000117342	7.0389E-06	3.18746	up	7498	XDH	
CB_029971	0.000117102	7.0171E-06	3.616107	up	345630	FBLL1	
CB_012809	0.000115941	6.9273E-06	2.882103	up	8851	CDK5R1	
CB_009572	0.00011518	6.8672E-06	2.113304	up	100130932	LOC100130932	
CB_022564	0.000114927	6.8467E-06	3.541802	up	80119	PIF1	
CB_006267	0.000114243	6.8041E-06	2.63061	up	122740	OR4K14	

CB_004929	0.000114155	6.7917E-06	6.126221	up	1294	COL7A1	
CB_011828	0.000112116	6.6424E-06	2.044025	up	5832	ALDH18A1	
CB_001210	0.000111504	6.5927E-06	2.177693	up	100291323	LOC100291323	
CB_016092	0.000111504	6.5933E-06	2.529071	up	3835	KIF22	
CB_000797	0.000110648	6.5181E-06	3.20291	up	645566	FLJ45482	
CB_029527	0.000109597	6.4372E-06	2.390707	up	23642	SNHG1	
CB_007094	0.00010948	6.4233E-06	5.25852	up	548596	CKMT1A	
CB_015399	0.000108903	6.3825E-06	3.526414	up	10643	IGF2BP3	
CB_028822	0.000108526	6.3484E-06	2.18059	up	622	BDH1	
CB_031751	0.000108331	6.3352E-06	7.994315	up			
CB_014088	0.000107428	6.279E-06	9.026941	up	1749	DLX5	
CB_020459	0.000107361	6.27E-06	2.176838	up	56915	EXOSC5	
CB_015844	0.000106839	6.2302E-06	2.373953	up	11072	DUSP14	
CB_021780	0.000106751	6.2191E-06	2.031241	up	10198	MPHOSPH9	
CB_002937	0.000105942	6.1653E-06	2.719525	up			
CB_003001	0.000105333	6.1209E-06	3.320161	up			
CB_009459	0.000105294	6.1142E-06	10.0256	up	27299	ADAMDEC1	
CB_020868	0.000104825	6.0726E-06	3.436967	up	57549	IGSF9	
CB_004640	0.000103122	5.9538E-06	2.774861	up			
CB_019844	0.000100828	5.7718E-06	2.66167	up	55771	PRR11	
CB_005309	0.000100597	5.7552E-06	2.267843	up	6528	SLC5A5	
CB_027553	0.000100431	5.7368E-06	4.605669	up	128239	IQGAP3	
CB_017009	0.000100418	5.734E-06	2.957592	up	24147	FJX1	
CB_025584	0.000100143	5.7088E-06	3.641022	up	165055	CCDC138	
CB_029650	9.98051E-05	5.6784E-06	2.192305	up	100101490	PSIMCT-1	
CB_029793	9.85345E-05	5.5952E-06	2.104835	up	100144603	LOC100144603	
CB_029352	9.79174E-05	5.5462E-06	2.279204	up	286016	TPI1P2	
CB_019518	9.7219E-05	5.4764E-06	9.048135	up	55040	EPN3	
CB_002986	9.68666E-05	5.4391E-06	2.944052	up			
CB_019914	9.49994E-05	5.3041E-06	2.316622	up	55342	STRBP	
CB_001734	9.46206E-05	5.28E-06	3.453042	up			
CB_024369	9.41345E-05	5.2469E-06	2.896377	up	114787	GPRIN1	
CB_024674	9.38069E-05	5.2122E-06	3.580589	up	91057	CCDC34	
CB_027637	9.37674E-05	5.2073E-06	2.166438	up	284098	PIGW	
CB_012189	9.36732E-05	5.2004E-06	2.550648	up	7023	TFAP4	
CB_030850	9.26243E-05	5.1157E-06	2.534826	up	1720	LOC1720	
CB_020830	9.19388E-05	5.0599E-06	2.313923	up	57510	XPO5	
CB_030173	9.15585E-05	5.0293E-06	2.951101	up	83956	RACGAP1P	
CB_027558	9.08507E-05	4.9803E-06	2.60222	up	145864	HAPLN3	
CB_010700	9.06323E-05	4.964E-06	4.908361	up	655	BMP7	
CB_013288	9.05252E-05	4.951E-06	9.883808	up	1832	DSP	
CB_020643	9.04011E-05	4.9413E-06	4.771003	up	5522	PPP2R2C	
CB_026908	8.94934E-05	4.869E-06	7.781741	up	26154	ABCA12	
CB_021182	8.83153E-05	4.7825E-06	2.089366	up	10793	ZNF273	
CB_002897	8.79639E-05	4.7514E-06	5.912432	up	100132159	LOC100132159	
CB_030427	8.77294E-05	4.7327E-06	2.6489	up	286467	LOC286467	
CB_029733	8.74752E-05	4.7162E-06	2.292852	up	340357	LOC340357	
CB_007397	8.70158E-05	4.679E-06	5.038149	up	196051	PPAPDC1A	
CB_005605	8.69222E-05	4.6726E-06	2.56088	up	1719	DHFR	
CB_023208	8.43301E-05	4.4704E-06	4.75588	up	83879	CDCA7	
CB_028366	8.31782E-05	4.3863E-06	3.336773	up	4810	NHS	
CB_031091	8.28092E-05	4.3588E-06	3.488011	up	131909	FAM172B	
CB_025626	8.25884E-05	4.3386E-06	2.776057	up	220042	C11orf82	
CB_023613	8.24942E-05	4.3287E-06	2.834059	up	84524	ZC3H8	
CB_012209	8.2126E-05	4.3002E-06	3.799476	up	7058	THBS2	
CB_025601	8.19677E-05	4.2854E-06	2.051914	up	200539	ANKRD23	

CB_011294	8.14547E-05	4.2509E-06	2.168284	up	3948	LDHC	
CB_028721	8.03035E-05	4.1641E-06	3.22341	up	55734	ZFP64	
CB_028597	8.02398E-05	4.1569E-06	2.270649	up	4521	NUDT1	
CB_009019	8.00934E-05	4.1443E-06	2.133552	up	1786	DNMT1	
CB_012015	7.99847E-05	4.1361E-06	2.673858	up	6474	SHOX2	
CB_023881	7.91792E-05	4.0756E-06	3.29021	up	84951	TNS4	
CB_002046	7.88027E-05	4.0488E-06	2.074662	up			
CB_031716	7.87507E-05	4.0448E-06	2.55626	up	646993	HMGB3P24	
CB_016767	7.7222E-05	3.9504E-06	4.210343	up	50943	FOXP3	
CB_000527	7.69713E-05	3.9303E-06	2.108033	up	254128	LOC254128	
CB_028426	7.69468E-05	3.9238E-06	2.120905	up	253714	MMS22L	
CB_001370	7.67802E-05	3.912E-06	2.412499	up			
CB_031224	7.66941E-05	3.9003E-06	2.173784	up	729595	HMGB3P22	
CB_031945	7.64514E-05	3.8855E-06	3.404183	up	641384	TMEM75	
CB_007977	7.62616E-05	3.8674E-06	2.412862	up	10592	SMC2	
CB_015845	7.58901E-05	3.8414E-06	2.107771	up	11073	TOPBP1	
CB_025037	7.53766E-05	3.8058E-06	2.361596	up	92935	MARS2	
CB_005375	7.46952E-05	3.7652E-06	8.388339	up	3209	HOXA13	
CB_015803	7.46204E-05	3.7531E-06	2.054449	up	7978	MTERF	
CB_006583	7.39022E-05	3.6997E-06	3.606106	up	116729	DYSFIP1	
CB_020400	7.31898E-05	3.6525E-06	16.3049	up	56649	TMPRSS4	
CB_000605	7.29852E-05	3.6389E-06	2.270241	up	100131242	LOC100131242	
CB_013566	7.29084E-05	3.6263E-06	26.23999	up	9119	KRT75	
CB_012467	0.000071882	3.5553E-06	2.143417	up	8349	HIST2H2BE	
CB_023209	7.15465E-05	3.5319E-06	9.139255	up	83879	CDCA7	
CB_017902	7.13038E-05	3.5098E-06	2.297006	up	23306	TMEM194A	
CB_009029	7.13038E-05	3.5131E-06	3.088656	up	199953	TMEM201	
CB_016717	7.13038E-05	3.509E-06	3.325726	up	4796	TONSL	
CB_012424	7.1007E-05	3.4843E-06	4.482373	up	8140	SLC7A5	
CB_020059	7.09775E-05	3.4813E-06	4.476389	up	4139	MARK1	
CB_014658	6.95111E-05	3.3851E-06	2.191805	up	10212	DDX39	
CB_008542	6.89358E-05	3.3451E-06	4.439571	up	26011	ODZ4	
CB_006765	6.85879E-05	3.3228E-06	23.95928	up	222584	FAM83B	
CB_012441	6.85842E-05	3.3198E-06	2.898152	up	8317	CDC7	
CB_007145	6.84937E-05	3.3128E-06	2.386887	up	131076	CCDC58	
CB_028845	6.84937E-05	3.3112E-06	2.914774	up	144455	E2F7	
CB_020114	6.69894E-05	3.2167E-06	2.205767	up	55920	RCC2	
CB_006057	6.65579E-05	3.1834E-06	9.825754	up	147920	IGFL2	
CB_007539	6.55669E-05	3.112E-06	2.414795	up	80179	MYO19	
CB_023238	6.53537E-05	3.0998E-06	4.948485	up	83903	GSG2	
CB_021367	6.49472E-05	3.0719E-06	2.552322	up	56652	C10orf2	
CB_025807	6.47234E-05	3.0525E-06	2.08521	up	93594	WDR67	
CB_018056	6.35297E-05	2.976E-06	2.192428	up	25879	DCAF13	
CB_008627	6.25128E-05	2.9155E-06	2.230419	up	55839	CENPN	
CB_028852	6.17603E-05	2.8687E-06	2.75034	up	3925	STMN1	
CB_026419	6.14446E-05	2.8455E-06	4.390986	up	256714	MAP7D2	
CB_023121	6.10929E-05	2.8163E-06	6.206811	up	83715	ESPN	
CB_020828	6.10927E-05	2.8151E-06	2.296819	up	57510	XPO5	
CB_013434	6.10927E-05	2.8154E-06	2.602563	up	4902	NRTN	
CB_028248	6.09891E-05	2.8087E-06	2.039714	up	202051	SPATA24	
CB_013867	6.09396E-05	2.8045E-06	10.45034	up	4100	MAGEA1	
CB_025525	6.08288E-05	2.7945E-06	2.731674	up	150696	PROM2	
CB_027451	5.99777E-05	2.7431E-06	3.417887	up	147700	KLC3	
CB_013289	5.94733E-05	2.7059E-06	10.72654	up	1832	DSP	
CB_007597	5.92955E-05	2.695E-06	2.00255	up	84792	C7orf70	
CB_027805	5.92077E-05	2.6883E-06	2.055981	up	337975	KRTAP20-1	

CB_018717	5.85059E-05	2.6377E-06	2.065157	up	51726	DNAJB11	
CB_013913	5.82832E-05	2.6221E-06	3.34015	up	5347	PLK1	
CB_031967	5.81184E-05	2.6082E-06	2.989365	up	100507636	LOC100507636	
CB_015030	5.77837E-05	2.5859E-06	5.694562	up	5083	PAX9	
CB_016785	5.73424E-05	2.5607E-06	2.232469	up	28969	BZW2	
CB_019349	5.69568E-05	2.5329E-06	2.249631	up	54908	CCDC99	
CB_026802	5.64736E-05	2.504E-06	3.374169	up	221613	HIST1H2AA	
CB_016718	5.64707E-05	2.5021E-06	2.267814	up	4796	TONSL	
CB_011599	5.56123E-05	2.4435E-06	2.282806	up	5203	PFDN4	
CB_030426	5.54941E-05	2.4316E-06	2.016504	up	286103	C8orf77	
CB_012442	5.46716E-05	2.3834E-06	3.476395	up	8318	CDC45	
CB_016259	5.40564E-05	2.3464E-06	3.343622	up	26256	CABYR	
CB_024335	5.38974E-05	2.3326E-06	2.236723	up	92667	C20orf72	
CB_023501	5.35938E-05	2.3155E-06	2.852026	up	84306	PDCD2L	
CB_019845	5.3129E-05	2.2841E-06	4.016465	up	55287	TMEM40	
CB_012474	5.276E-05	2.2582E-06	6.732579	up	8356	HIST1H3J	
CB_021959	5.19026E-05	2.2018E-06	3.315525	up	79000	C1orf135	
CB_024716	5.17361E-05	2.1931E-06	2.718371	up	128178	EDARADD	
CB_017509	5.10924E-05	2.1488E-06	2.671495	up	9918	NCAPD2	
CB_012470	5.0897E-05	2.1341E-06	2.987672	up	8352	HIST1H3C	
CB_022065	4.98946E-05	2.0779E-06	2.97833	up	79172	CENPO	
CB_017963	4.98946E-05	2.0777E-06	17.70359	up	50649	ARHGEF4	
CB_023495	4.96556E-05	2.0655E-06	2.080648	up	84300	C6orf125	
CB_018621	4.94494E-05	2.0476E-06	2.149897	up	9585	KIF20B	
CB_016855	4.89274E-05	2.0089E-06	2.089771	up	29088	MRPL15	
CB_022357	4.89274E-05	2.0087E-06	2.460944	up	79828	METTL8	
CB_023897	4.86148E-05	1.9845E-06	2.12704	up	84968	PNMA6A	
CB_012075	4.86143E-05	1.9837E-06	2.067868	up	6636	SNRPF	
CB_028980	4.82606E-05	1.9662E-06	4.537227	up	389336	C5orf46	
CB_029522	4.79673E-05	1.9482E-06	5.857056	up	386757	SLC6A10P	
CB_010336	4.77719E-05	1.9325E-06	17.87066	up	1470	CST2	
CB_026660	4.77399E-05	1.9291E-06	2.011967	up	9631	NUP155	
CB_031569	4.72281E-05	1.8977E-06	3.019769	up	100506545	LOC100506545	
CB_008088	4.67807E-05	1.8733E-06	2.589749	up	7366	UGT2B15	
CB_000684	4.67807E-05	1.8728E-06	4.530121	up	100128338	LOC100128338	
CB_010345	4.67807E-05	1.874E-06	7.941172	up	1515	CTSL2	
CB_025111	4.61531E-05	1.8408E-06	2.18408	up	154467	C6orf129	
CB_012420	4.5826E-05	1.8105E-06	5.015555	up	8091	HMGA2	
CB_003960	4.5826E-05	1.8113E-06	7.093533	up			
CB_010085	4.57143E-05	1.803E-06	4.401907	up	54478	FAM64A	
CB_000180	4.53144E-05	1.7813E-06	2.335055	up			
CB_016800	4.4572E-05	1.7441E-06	2.305415	up	28982	FLVCR1	
CB_016015	4.42744E-05	1.7287E-06	7.380029	up	11247	NXPH4	
CB_029846	4.3079E-05	1.6725E-06	2.592434	up	57291	KIAA0114	
CB_031829	4.3079E-05	1.6735E-06	3.475899	up			
CB_024268	4.28471E-05	1.6594E-06	2.197506	up	91433	RCCD1	
CB_025622	4.23292E-05	1.6313E-06	2.624367	up	219844	HYLS1	
CB_022931	4.23292E-05	1.63E-06	2.817448	up	81624	DIAPH3	
CB_020261	4.20665E-05	1.6151E-06	2.45542	up	54517	PUS7	
CB_024216	4.18201E-05	1.6037E-06	2.039968	up	93323	HAUS8	
CB_005088	4.10369E-05	1.5567E-06	2.494469	up	4436	MSH2	
CB_024577	4.05696E-05	1.53E-06	2.396023	up	114907	FBXO32	
CB_018055	4.0032E-05	1.5015E-06	2.242689	up	25879	DCAF13	
CB_022854	3.96992E-05	1.4821E-06	2.013255	up	81557	MAGED4B	
CB_016755	3.96601E-05	1.48E-06	4.389601	up	1734	DIO2	
CB_026067	3.84004E-05	1.4074E-06	2.119566	up	128061	C1orf131	

CB_012071	3.83682E-05	1.405E-06	2.081313	up	6635	SNRPE	
CB_003633	3.8301E-05	1.3995E-06	3.134255	up			
CB_014493	3.82483E-05	1.3958E-06	5.811642	up	6535	SLC6A8	
CB_023542	3.78812E-05	1.3764E-06	2.112082	up	84365	MKI67IP	
CB_000936	3.72489E-05	1.3469E-06	3.142978	up			
CB_024460	3.72341E-05	1.3458E-06	4.065222	up	5217	PFN2	
CB_010200	3.66119E-05	1.3181E-06	16.75846	up	5013	OTX1	
CB_011629	3.63636E-05	1.3034E-06	24.97164	up	5307	PITX1	
CB_012072	3.62388E-05	1.296E-06	2.207713	up	6635	SNRPE	
CB_029855	3.61113E-05	1.2857E-06	3.769532	up	646024	RAET1K	
CB_015029	3.58678E-05	1.2731E-06	4.192195	up	5083	PAX9	
CB_004589	3.58299E-05	1.27E-06	4.263185	up			
CB_024139	3.48798E-05	1.2259E-06	6.298725	up	10595	ERN2	
CB_001434	3.47459E-05	1.2201E-06	3.06672	up			
CB_026175	3.36218E-05	1.1641E-06	2.421344	up	148304	C1orf74	
CB_005343	3.28389E-05	1.1281E-06	10.74474	up	1300	COL10A1	
CB_014184	3.25873E-05	1.1138E-06	3.751467	up	3009	HIST1H1B	
CB_032147	3.19897E-05	1.0845E-06	4.374562	up	100507949	LOC100507949	
CB_008625	3.17604E-05	1.0754E-06	5.604432	up	10024	TROAP	
CB_020170	3.14902E-05	1.0613E-06	8.437497	up	3206	HOXA10	
CB_011850	3.14135E-05	1.0572E-06	2.011545	up	5902	RANBP1	
CB_019422	3.14078E-05	1.0565E-06	2.336281	up	54962	TIPIN	
CB_016754	3.13827E-05	1.0531E-06	2.766303	up	1734	DIO2	
CB_012235	3.12046E-05	1.0443E-06	2.694381	up	7112	TMPO	
CB_011919	3.10504E-05	1.0374E-06	2.200725	up	6182	MRPL12	
CB_010772	3.10504E-05	1.0376E-06	4.383635	up	995	CDC25C	
CB_028513	3.07365E-05	1.0222E-06	3.382159	up	375444	C5orf34	
CB_004894	3.06152E-05	1.0148E-06	2.771989	up	675	BRCA2	
CB_026212	3.04999E-05	1.0105E-06	2.05176	up	152002	C3orf21	
CB_023520	3.03018E-05	1.003E-06	2.895828	up	84321	THOC3	
CB_004496	3.02898E-05	1.0016E-06	2.197198	up			
CB_013909	2.95532E-05	9.717E-07	2.002528	up	5296	PIK3R2	
CB_019640	2.95532E-05	9.712E-07	3.890207	up	55143	CDCA8	
CB_031629	2.93967E-05	9.6419E-07	20.73299	up	100509927	LOC100509927	
CB_029714	2.92393E-05	9.567E-07	2.813276	up	643432	TSG1	
CB_017334	2.91223E-05	9.5057E-07	2.752867	up	9735	KNTC1	
CB_015511	2.90197E-05	9.4676E-07	4.169212	up	65268	WNK2	
CB_016819	2.89747E-05	9.4437E-07	2.038822	up	28998	MRPL13	
CB_012927	2.86806E-05	9.3206E-07	24.75613	up	2706	GJB2	
CB_024931	2.79634E-05	9.0166E-07	6.26073	up	171177	RHOV	
CB_032150	2.78852E-05	8.9781E-07	6.805207	up	654780	LOC654780	
CB_023543	2.77648E-05	8.9262E-07	2.270814	up	84365	MKI67IP	
CB_019662	2.76076E-05	8.8675E-07	2.297816	up	55159	RFWD3	
CB_010771	2.74903E-05	8.7987E-07	5.179363	up	993	CDC25A	
CB_019677	2.74505E-05	8.7772E-07	8.607117	up	259266	ASPM	
CB_031117	2.71911E-05	8.6779E-07	2.886653	up	100306975	LOC100306975	
CB_019559	2.71617E-05	8.6634E-07	4.023965	up	53335	BCL11A	
CB_029948	2.66012E-05	8.454E-07	2.139512	up	151507	MSL3L2	
CB_020757	2.66012E-05	8.4531E-07	4.608538	up	57405	SPC25	
CB_013955	2.65609E-05	8.4254E-07	3.619396	up	6564	SLC15A1	
CB_017510	2.65477E-05	8.417E-07	2.49405	up	9918	NCAPD2	
CB_032151	2.63766E-05	8.3502E-07	5.149299	up	654780	LOC654780	
CB_018746	2.58538E-05	8.0986E-07	6.981763	up	51195	RAPGEFL1	
CB_012995	2.58059E-05	8.0714E-07	2.267966	up	1978	EIF4EBP1	
CB_021840	2.56942E-05	8.0324E-07	3.576848	up	64946	CENPH	
CB_021883	2.56877E-05	8.0247E-07	2.140788	up	65244	SPATS2	

CB_021502	2.55154E-05	7.9482E-07	2.865227	up	63922	CHTF18	
CB_025073	2.51337E-05	7.8072E-07	5.94409	up	113828	FAM83F	
CB_014110	2.50155E-05	7.7528E-07	3.361675	up	2139	EYA2	
CB_014836	2.46297E-05	7.6072E-07	2.016178	up	7203	CCT3	
CB_015025	2.4444E-05	7.5059E-07	2.004403	up	5036	PA2G4	
CB_012219	2.42471E-05	7.3878E-07	2.002453	up	7083	TK1	
CB_012472	2.40128E-05	7.3012E-07	3.194159	up	8354	HIST1H3I	
CB_005481	2.32081E-05	6.9572E-07	4.774446	up	3589	IL11	
CB_031282	2.31541E-05	6.9296E-07	2.510454	up			
CB_026206	2.28478E-05	6.7929E-07	3.770393	up	151246	SGOL2	
CB_019516	2.28478E-05	6.7908E-07	3.949524	up	55038	CDCA4	
CB_024326	2.24946E-05	6.6489E-07	2.412249	up	91862	MARVELD3	
CB_008805	2.24394E-05	6.62E-07	5.744041	up	55635	DEPDC1	
CB_011585	2.23449E-05	6.578E-07	3.523224	up	5163	PDK1	
CB_014659	2.21462E-05	6.4844E-07	2.36081	up	10213	PSMD14	
CB_005606	2.19515E-05	6.3999E-07	2.380036	up	1719	DHFR	
CB_022911	2.19136E-05	6.385E-07	6.800157	up	81610	FAM83D	
CB_026698	2.17287E-05	6.2932E-07	3.052826	up	195828	ZNF367	
CB_021902	2.17256E-05	6.2889E-07	6.690678	up	53836	GPR87	
CB_019986	2.16021E-05	6.2223E-07	3.686208	up	55839	CENPN	
CB_016676	2.15663E-05	6.2052E-07	5.779434	up	26585	GREM1	
CB_022356	2.14088E-05	6.1463E-07	2.495459	up	79828	METTL8	
CB_015496	2.13392E-05	6.1144E-07	2.613091	up	10797	MTHFD2	
CB_020058	2.08528E-05	5.9437E-07	3.516595	up	55506	H2AFY2	
CB_019527	1.99619E-05	5.5857E-07	7.027973	up	55061	SUSD4	
CB_020532	1.99128E-05	5.5653E-07	5.428022	up	56992	KIF15	
CB_031669	1.94882E-05	5.4219E-07	2.295451	up	100508181	LOC100508181	
CB_022492	1.92153E-05	5.3124E-07	2.670595	up	79980	DSN1	
CB_017593	1.91398E-05	5.2741E-07	2.085226	up	6683	SPAST	
CB_003537	1.89132E-05	5.1728E-07	3.399891	up			
CB_024158	1.8814E-05	5.1378E-07	3.855909	up	7804	LRP8	
CB_019694	1.87692E-05	5.1177E-07	3.007658	up	55723	ASF1B	
CB_025865	1.85402E-05	5.0406E-07	2.757281	up	11200	CHEK2	
CB_028720	1.83455E-05	4.9557E-07	2.295322	up	55734	ZFP64	
CB_025092	1.80853E-05	4.8538E-07	6.88293	up	115908	CTHRC1	
CB_022605	1.79196E-05	4.798E-07	2.311769	up	80178	C16orf59	
CB_025932	1.78165E-05	4.7619E-07	4.064807	up	8784	TNFRSF18	
CB_027283	1.75355E-05	4.6628E-07	2.834993	up	128312	HIST3H2BB	
CB_021953	1.72938E-05	4.5646E-07	4.089306	up	78995	C17orf53	
CB_017410	1.72367E-05	4.5396E-07	2.110681	up	9816	URB2	
CB_021305	1.72367E-05	4.5394E-07	3.729505	up	59344	ALOXE3	
CB_014783	1.69108E-05	4.4199E-07	6.583206	up	4320	MMP11	
CB_011835	1.69108E-05	4.4207E-07	19.19574	up	5865	RAB3B	
CB_009847	1.68914E-05	4.4102E-07	3.762183	up	78995	C17orf53	
CB_010202	1.68657E-05	4.3796E-07	2.630066	up	79172	CENPO	
CB_028610	1.68657E-05	4.3849E-07	3.229629	up	284252	KCTD1	
CB_000722	1.68224E-05	4.3602E-07	7.675454	up	100130899	LOC100130899	
CB_026742	1.67366E-05	4.33E-07	5.757341	up	5831	PYCR1	
CB_028095	1.65715E-05	4.2768E-07	2.471958	up	84515	MCM8	
CB_018074	1.64521E-05	4.2327E-07	2.343061	up	25902	MTHFD1L	
CB_007505	1.63346E-05	4.1768E-07	2.289331	up	6240	RRM1	
CB_031177	1.6276E-05	4.1515E-07	5.056365	up	145438	C14orf82	
CB_019898	1.61499E-05	4.0988E-07	6.956323	up	55789	DEPDC1B	
CB_022215	1.61359E-05	4.0927E-07	4.806212	up	79682	MLF1IP	
CB_010329	1.5932E-05	4.0243E-07	2.002664	up	1434	CSE1L	
CB_017381	1.57383E-05	3.9595E-07	4.34346	up	9787	DLGAP5	

CB_007130	1.57255E-05	3.9461E-07	9.223935	up	157570	ESCO2	
CB_006970	1.56528E-05	3.9079E-07	2.154793	up	400506	C16orf88	
CB_022522	1.56471E-05	3.8968E-07	2.05106	up	80018	NAA25	
CB_011630	1.55925E-05	3.8758E-07	22.49455	up	5307	PITX1	
CB_023120	1.52224E-05	3.7573E-07	6.722976	up	83715	ESPN	
CB_020620	1.51958E-05	3.7448E-07	2.216449	up	10017	BCL2L10	
CB_027098	1.51098E-05	3.7127E-07	4.498509	up	284403	WDR62	
CB_018944	1.50436E-05	3.6869E-07	2.188124	up	11335	CBX3	
CB_027647	1.50159E-05	3.6739E-07	6.465289	up	338707	B4GALNT4	
CB_014181	1.49672E-05	3.6563E-07	2.43189	up	3006	HIST1H1C	
CB_024544	1.49638E-05	3.6531E-07	3.314189	up	9134	CCNE2	
CB_030679	1.48941E-05	3.6337E-07	2.275534	up	100132724	LOC100132724	
CB_008475	1.47705E-05	3.5892E-07	6.177717	up	92312	MEX3A	
CB_014821	1.47447E-05	3.5786E-07	7.619451	up	6495	SIX1	
CB_012435	1.47121E-05	3.5683E-07	2.377335	up	8294	HIST1H4I	
CB_014338	1.46934E-05	3.5591E-07	2.36193	up	10036	CHAF1A	
CB_014265	1.46278E-05	3.5338E-07	2.15504	up	6472	SHMT2	
CB_016930	1.3582E-05	3.1629E-07	3.307597	up	10733	PLK4	
CB_016366	1.35199E-05	3.1442E-07	3.355905	up	9700	ESPL1	
CB_024060	1.34306E-05	3.1156E-07	9.669262	up	85416	ZIC5	
CB_011400	1.34306E-05	3.117E-07	15.82063	up	4321	MMP12	
CB_006726	1.34023E-05	3.0998E-07	2.181593	up	26147	PHF19	
CB_021508	1.33616E-05	3.0835E-07	2.272328	up	63926	ANKRD5	
CB_028846	1.33616E-05	3.084E-07	9.603248	up	144455	E2F7	
CB_014135	1.31096E-05	2.9989E-07	25.47843	up	2709	GJB5	
CB_010809	1.30848E-05	2.987E-07	2.539847	up	1163	CKS1B	
CB_029120	1.30273E-05	2.9697E-07	4.057632	up	728833	FAM72D	
CB_023432	1.29719E-05	2.9427E-07	2.054446	up	84231	TRAF7	
CB_022994	1.29145E-05	2.9276E-07	4.49094	up	81930	KIF18A	
CB_023517	1.2841E-05	2.8947E-07	2.410538	up	84319	C3orf26	
CB_012486	1.28334E-05	2.8876E-07	2.274339	up	8365	HIST1H4H	
CB_016944	1.27473E-05	2.8595E-07	3.877003	up	22824	HSPA4L	
CB_012076	1.27368E-05	2.855E-07	2.189718	up	6637	SNRPG	
CB_024558	1.2545E-05	2.8057E-07	4.088986	up	29968	PSAT1	
CB_003284	1.2446E-05	2.7653E-07	2.987442	up			
CB_007204	1.23184E-05	2.7339E-07	5.330518	up	2175	FANCA	
CB_021456	1.21174E-05	2.6739E-07	2.771146	up	27085	MTBP	
CB_024751	1.20885E-05	2.6599E-07	2.119122	up	140893	C20orf151	
CB_022828	1.18996E-05	2.6057E-07	3.433698	up	91057	CCDC34	
CB_029338	1.17614E-05	2.5618E-07	3.33086	up	128872	HMGB3P1	
CB_015370	1.17215E-05	2.5475E-07	9.346066	up	6513	SLC2A1	
CB_009781	1.15848E-05	2.5142E-07	2.720401	up	6566	SLC16A1	
CB_030456	1.15355E-05	2.4989E-07	3.556273	up	93429	DKFZp434J0226	
CB_014060	1.12636E-05	2.4266E-07	7.160536	up	1033	CDKN3	
CB_016530	1.10399E-05	2.3631E-07	3.535944	up	7371	UCK2	
CB_022329	1.09919E-05	2.3437E-07	4.399262	up	79801	SHCBP1	
CB_028811	1.06897E-05	2.2504E-07	7.541266	up	5818	PVRL1	
CB_015585	1.05287E-05	2.1932E-07	4.668278	up	2491	CENPI	
CB_014059	1.04908E-05	2.182E-07	6.144438	up	1033	CDKN3	
CB_012801	1.04611E-05	2.1691E-07	2.481523	up	8833	GMPS	
CB_004467	1.03127E-05	2.1138E-07	11.41218	up			
CB_024251	1.02995E-05	2.103E-07	3.214486	up	85465	EPT1	
CB_007557	1.02683E-05	2.0933E-07	4.44416	up	6241	RRM2	
CB_011654	1.01662E-05	2.0662E-07	2.620968	up	5427	POLE2	
CB_018007	9.77017E-06	1.9624E-07	2.016842	up	23518	R3HDM1	
CB_010810	9.63392E-06	1.9293E-07	3.359889	up	1163	CKS1B	

CB_027471	9.55727E-06	1.906E-07	4.428609	up	8612	PPAP2C	
CB_029719	9.39343E-06	1.8514E-07	2.233861	up	388796	LOC388796	
CB_005125	9.08438E-06	1.7569E-07	9.921488	up	5080	PAX6	
CB_014351	8.88011E-06	1.7076E-07	2.272396	up	10051	SMC4	
CB_012917	8.84384E-06	1.6992E-07	2.441238	up	8424	BBOX1	
CB_025147	8.56445E-06	1.6279E-07	2.50134	up	26472	PPP1R14B	
CB_011920	8.3864E-06	1.5754E-07	2.370337	up	6182	MRPL12	
CB_010792	8.27862E-06	1.5434E-07	11.8113	up	1058	CENPA	
CB_014352	8.26265E-06	1.5366E-07	2.818569	up	10051	SMC4	
CB_023240	8.26265E-06	1.5378E-07	4.40258	up	891	CCNB1	
CB_011883	8.24903E-06	1.53E-07	3.335131	up	5984	RFC4	
CB_011667	8.24088E-06	1.5272E-07	2.570835	up	5471	PPAT	
CB_021522	8.14303E-06	1.5E-07	6.626257	up	63967	CLSPN	
CB_016071	8.12185E-06	1.4935E-07	3.641139	up	11339	OIP5	
CB_031642	8.04135E-06	1.4736E-07	2.696209	up	100508670	LOC100508670	
CB_015891	7.93957E-06	1.4477E-07	3.018596	up	11169	WDHD1	
CB_011551	7.91655E-06	1.4395E-07	2.007595	up	5050	PAFAH1B3	
CB_011666	7.91655E-06	1.4372E-07	3.364963	up	5471	PPAT	
CB_000530	7.87499E-06	1.4269E-07	5.064137	up			
CB_020113	7.87117E-06	1.4219E-07	2.168357	up	55920	RCC2	
CB_019047	7.82293E-06	1.4038E-07	7.549952	up	51804	SIX4	
CB_028200	7.77468E-06	1.3939E-07	2.085212	up	5933	RBL1	
CB_021264	7.74834E-06	1.388E-07	2.718986	up	58516	FAM60A	
CB_003703	7.61727E-06	1.3524E-07	3.68378	up			
CB_010126	7.59306E-06	1.3437E-07	4.76912	up	638	BIK	
CB_024252	7.42678E-06	1.2986E-07	2.904585	up	85465	EPT1	
CB_005029	7.25838E-06	1.2565E-07	2.671419	up	3251	HPRT1	
CB_013119	7.18731E-06	1.2419E-07	4.050904	up	9319	TRIP13	
CB_026863	7.14772E-06	1.2236E-07	5.844082	up	79412	KREMEN2	
CB_020479	6.95244E-06	1.1732E-07	6.563676	up	56938	ARNTL2	
CB_008258	6.95105E-06	1.1713E-07	6.302294	up	146909	KIF18B	
CB_023039	6.89541E-06	1.1568E-07	4.620087	up	83461	CDCA3	
CB_012519	6.73141E-06	1.1141E-07	4.154563	up	8438	RAD54L	
CB_012490	6.56889E-06	1.0799E-07	3.749995	up	8368	HIST1H4L	
CB_019319	6.50484E-06	1.0642E-07	3.587252	up	54892	NCAPG2	
CB_003525	6.34918E-06	1.0341E-07	4.39138	up			
CB_015320	6.11342E-06	9.7301E-08	5.842794	up	10615	SPAG5	
CB_025122	6.10186E-06	9.7021E-08	6.68411	up	53335	BCL11A	
CB_017422	6.0987E-06	9.6874E-08	7.732533	up	9833	MELK	
CB_009272	5.75754E-06	8.9264E-08	2.722427	up	90417	C15orf23	
CB_021976	5.6467E-06	8.7009E-08	2.067742	up	79023	NUP37	
CB_020161	5.60986E-06	8.5996E-08	2.405964	up	54069	C21orf45	
CB_013574	5.56639E-06	8.5065E-08	5.338049	up	9133	CCNB2	
CB_023954	5.55342E-06	8.4533E-08	5.045561	up	11130	ZWINT	
CB_012469	5.54494E-06	8.3985E-08	2.926324	up	8351	HIST1H3D	
CB_015584	5.33211E-06	7.9625E-08	4.267442	up	2491	CENPI	
CB_010271	5.23447E-06	7.7757E-08	3.958596	up	991	CDC20	
CB_016537	5.23447E-06	7.7918E-08	5.378816	up	3161	HMMR	
CB_010171	5.15108E-06	7.6187E-08	9.227006	up	64220	STRA6	
CB_028883	5.13789E-06	7.5911E-08	2.821882	up	253782	LASS6	
CB_012480	5.01923E-06	7.3123E-08	2.314594	up	8360	HIST1H4D	
CB_014339	4.96933E-06	7.1989E-08	2.714127	up	10036	CHAF1A	
CB_028333	4.96839E-06	7.1831E-08	3.186395	up	4830	NME1	
CB_012331	4.96363E-06	7.1684E-08	2.683237	up	7443	VRK1	
CB_016403	4.94884E-06	7.1259E-08	2.165871	up	25800	SLC39A6	
CB_013138	4.78532E-06	6.7743E-08	2.845964	up	9401	RECQL4	

CB_002788	4.78293E-06	6.756E-08	2.568106	up			
CB_023492	4.76112E-06	6.7098E-08	4.77748	up	84296	GINS4	
CB_029409	4.75444E-06	6.6859E-08	4.523721	up	26255	PTTG3P	
CB_009775	4.6747E-06	6.5139E-08	2.598289	up	117178	SSX2IP	
CB_018080	4.6747E-06	6.508E-08	9.715413	up	25907	TMEM158	
CB_019747	4.65194E-06	6.4675E-08	3.040109	up	26586	CKAP2	
CB_015771	4.62104E-06	6.3952E-08	2.142726	up	6632	SNRPD1	
CB_012082	4.59704E-06	6.3401E-08	3.814452	up	6652	SORD	
CB_014751	4.55599E-06	6.2619E-08	2.681293	up	4175	MCM6	
CB_014592	4.49299E-06	6.1397E-08	7.301994	up	10112	KIF20A	
CB_012447	4.44786E-06	6.0357E-08	8.964268	up	8329	HIST1H2AI	
CB_025873	4.42781E-06	5.9944E-08	5.025422	up	3159	HMGA1	
CB_024929	4.42781E-06	5.9826E-08	8.295918	up	165545	DQX1	
CB_012627	4.26196E-06	5.6348E-08	5.203899	up	9156	EXO1	
CB_012074	4.20195E-06	5.5221E-08	2.516895	up	6635	SNRPE	
CB_023273	4.13173E-06	5.3971E-08	8.51858	up	83990	BRIP1	
CB_019748	4.09331E-06	5.321E-08	3.434641	up	26586	CKAP2	
CB_031375	4.09331E-06	5.3187E-08	4.406344	up	100130009	LOC100130009	
CB_012845	4.09328E-06	5.3079E-08	3.226373	up	8914	TIMELESS	
CB_019637	3.78727E-06	4.755E-08	5.383125	up	1894	ECT2	
CB_010795	3.75404E-06	4.6955E-08	5.135078	up	1062	CENPE	
CB_015574	3.67698E-06	4.5816E-08	3.23456	up	10926	DBF4	
CB_016597	3.61911E-06	4.4751E-08	8.661786	up	29128	UHRF1	
CB_016083	3.57351E-06	4.3986E-08	3.721623	up	672	BRCA1	
CB_010373	3.46104E-06	4.1699E-08	2.795596	up	1736	DKC1	
CB_025784	3.46104E-06	4.1643E-08	3.107757	up	199731	CADM4	
CB_020019	3.41014E-06	4.0481E-08	8.780683	up	55872	PBK	
CB_012804	3.35967E-06	3.946E-08	3.644258	up	8836	GGH	
CB_019627	3.31379E-06	3.8454E-08	2.21219	up	55706	TMEM48	
CB_004893	3.28121E-06	3.7867E-08	3.50403	up	641	BLM	
CB_017415	3.2805E-06	3.7807E-08	4.621106	up	9824	ARHGAP11A	
CB_006895	3.27846E-06	3.7732E-08	6.063315	up	151648	SGOL1	
CB_024229	3.27828E-06	3.7527E-08	3.317228	up	92815	HIST3H2A	
CB_014946	3.21022E-06	3.6183E-08	7.975562	up	10403	NDC80	
CB_011390	3.17492E-06	3.5685E-08	7.856418	up	4288	MKI67	
CB_027951	3.15461E-06	3.5306E-08	7.579261	up	147841	SPC24	
CB_021507	3.12767E-06	3.4906E-08	2.407157	up	63926	ANKRD5	
CB_019857	3.10965E-06	3.4409E-08	2.327043	up	55299	BRIX1	
CB_019605	3.03437E-06	3.3143E-08	4.819413	up	3070	HELLS	
CB_017852	3.02726E-06	3.2847E-08	2.700294	up	23246	BOP1	
CB_016825	2.98494E-06	3.2015E-08	2.962261	up	29028	ATAD2	
CB_016856	2.96649E-06	3.1618E-08	6.33751	up	29089	UBE2T	
CB_008167	2.89692E-06	3.0723E-08	3.216398	up	10606	PAICS	
CB_026199	2.86735E-06	3.0273E-08	6.568364	up	150468	CKAP2L	
CB_021970	2.85853E-06	3.0135E-08	2.740529	up	79017	GGCT	
CB_012475	2.85853E-06	3.005E-08	4.951139	up	8357	HIST1H3H	
CB_006432	2.83522E-06	2.9754E-08	4.386206	up	333932	HIST2H3A	
CB_016390	2.78669E-06	2.9024E-08	6.763268	up	24137	KIF4A	
CB_019526	2.70029E-06	2.7696E-08	3.220205	up	55055	ZWILCH	
CB_019678	2.66954E-06	2.7305E-08	12.63597	up	259266	ASPM	
CB_019732	2.65135E-06	2.7026E-08	3.323821	up	55215	FANCI	
CB_011845	2.6425E-06	2.6852E-08	2.009393	up	5888	RAD51	
CB_024689	2.57055E-06	2.5754E-08	9.819454	up	113130	CDCA5	
CB_011439	2.57037E-06	2.5711E-08	3.472909	up	4605	MYBL2	
CB_008048	2.52043E-06	2.4972E-08	3.08281	up	1104	RCC1	
CB_023304	2.51028E-06	2.4712E-08	7.878419	up	84057	MND1	

CB_021557	2.46825E-06	2.422E-08	3.639717	up	64105	CENPK	
CB_011473	2.46463E-06	2.4107E-08	8.249326	up	4751	NEK2	
CB_025828	2.41177E-06	2.3246E-08	7.229286	up	83540	NUF2	
CB_001884	2.40151E-06	2.3071E-08	4.629612	up			
CB_022258	2.34401E-06	2.2332E-08	2.488512	up	79723	SUV39H2	
CB_019481	2.30212E-06	2.1386E-08	5.477889	up	55010	C12orf48	
CB_012991	2.23263E-06	2.049E-08	5.98271	up	1870	E2F2	
CB_027293	2.19166E-06	1.9873E-08	2.14877	up	317772	HIST2H2AB	
CB_010254	2.10279E-06	1.8668E-08	6.674142	up	890	CCNA2	
CB_007205	2.08881E-06	1.8444E-08	5.129974	up	2187	FANCB	
CB_018763	2.08881E-06	1.8387E-08	6.716917	up	51203	NUSAP1	
CB_012452	2.0888E-06	1.8296E-08	4.460282	up	8335	HIST1H2AB	
CB_020248	2.08726E-06	1.8202E-08	2.411378	up	54503	ZDHHC13	
CB_013403	2.0838E-06	1.8102E-08	2.818788	up	4171	MCM2	
CB_019941	1.94973E-06	1.6474E-08	10.303	up	55355	HJURP	
CB_026757	1.94576E-06	1.641E-08	6.592221	up	57082	CASC5	
CB_001124	1.90338E-06	1.5962E-08	2.913111	up	84914	ZNF587	
CB_007559	1.86722E-06	1.5518E-08	2.893287	up	554313	HIST2H4B	
CB_013010	1.86722E-06	1.5508E-08	4.08397	up	2237	FEN1	
CB_012463	1.82248E-06	1.4994E-08	2.142844	up	8346	HIST1H2BI	
CB_006749	1.76631E-06	1.4196E-08	2.087303	up	135293	PM20D2	
CB_024634	1.76622E-06	1.4042E-08	2.238025	up	85235	HIST1H2AH	
CB_026001	1.76622E-06	1.4116E-08	4.13092	up	116028	C16orf75	
CB_008257	1.75095E-06	1.3795E-08	3.788766	up	146909	KIF18B	
CB_029317	1.71454E-06	1.3403E-08	4.467909	up	7012	TERC	
CB_012487	1.69392E-06	1.3185E-08	2.171165	up	8366	HIST1H4B	
CB_012489	1.69392E-06	1.3159E-08	5.263881	up	8368	HIST1H4L	
CB_031099	1.63793E-06	1.2334E-08	2.644712	up	100128881	LOC100128881	
CB_017149	1.63793E-06	1.2303E-08	3.258325	up	27338	UBE2S	
CB_011392	1.52888E-06	1.1294E-08	6.120199	up	4288	MKI67	
CB_019240	1.4887E-06	1.0835E-08	3.803886	up	54821	ERCC6L	
CB_007129	1.4887E-06	1.0776E-08	6.970703	up	157570	ESCO2	
CB_012913	1.47885E-06	1.0651E-08	5.993328	up	9055	PRC1	
CB_025119	1.4684E-06	1.0522E-08	5.97232	up	9493	KIF23	
CB_015779	1.45296E-06	1.0388E-08	4.176383	up	6712	SPTBN2	
CB_029056	1.43385E-06	1.0183E-08	3.257502	up	197021	LCTL	
CB_011250	1.40411E-06	9.9497E-09	3.114931	up	3838	KPNA2	
CB_022925	1.39438E-06	9.705E-09	6.522971	up	81620	CDT1	
CB_012449	1.39438E-06	9.7412E-09	7.078414	up	8332	HIST1H2AL	
CB_020086	1.39438E-06	9.8312E-09	9.744709	up	54443	ANLN	
CB_012482	1.37615E-06	9.428E-09	2.546804	up	8361	HIST1H4F	
CB_022009	1.37615E-06	9.3884E-09	4.821465	up	79075	DSCC1	
CB_021099	1.35461E-06	9.0836E-09	2.318683	up	8331	HIST1H2AJ	
CB_012448	1.33103E-06	8.8941E-09	2.298758	up	8330	HIST1H2AK	
CB_007207	1.32091E-06	8.7692E-09	2.896934	up	2177	FANCD2	
CB_023486	1.32091E-06	8.7738E-09	4.280398	up	9718	ECE2	
CB_021972	1.31786E-06	8.6925E-09	4.323378	up	79019	CENPM	
CB_025118	1.27685E-06	8.2788E-09	4.921252	up	9493	KIF23	
CB_021825	1.27685E-06	8.2761E-09	7.235778	up	53335	BCL11A	
CB_026005	1.27632E-06	8.2324E-09	4.60358	up	120071	GYLTL1B	
CB_009770	1.27397E-06	8.1402E-09	5.901169	up	9319	TRIP13	
CB_019671	1.25708E-06	7.8268E-09	10.79581	up	55165	CEP55	
CB_019672	1.24354E-06	7.6685E-09	8.735747	up	55165	CEP55	
CB_013401	1.22603E-06	7.5217E-09	5.177374	up	3832	KIF11	
CB_016596	1.22219E-06	7.3848E-09	5.068424	up	29128	UHRF1	
CB_008045	1.20721E-06	7.2531E-09	5.424614	up	6491	STIL	

CB_010234	1.2071E-06	7.2036E-09	4.350581	up	701	BUB1B	
CB_011646	1.19071E-06	6.9274E-09	4.955171	up	5367	PMCH	
CB_021100	1.17223E-06	6.727E-09	6.939571	up	9837	GINS1	
CB_016391	1.16412E-06	6.6445E-09	3.230609	up	24137	KIF4A	
CB_015698	1.16412E-06	6.662E-09	5.645041	up	11004	KIF2C	
CB_012273	1.15325E-06	6.4719E-09	7.397501	up	7272	TTK	
CB_012471	1.14869E-06	6.3734E-09	4.09409	up	8353	HIST1H3E	
CB_011246	1.1309E-06	6.1326E-09	5.277687	up	3833	KIFC1	
CB_011340	1.12408E-06	6.0409E-09	5.72598	up	4085	MAD2L1	
CB_018751	1.12408E-06	5.9829E-09	11.11619	up	1063	CENPF	
CB_028714	1.105E-06	5.8157E-09	2.994981	up	10721	POLQ	
CB_016985	1.07648E-06	5.5261E-09	6.435249	up	23594	ORC6	
CB_031841	1.07496E-06	5.4872E-09	2.0154	up			
CB_011569	1.04192E-06	5.2685E-09	3.06372	up	5111	PCNA	
CB_028030	1.04192E-06	5.2373E-09	4.430143	up	348654	GEN1	
CB_028805	1.02336E-06	5.1265E-09	3.628467	up	2305	FOXM1	
CB_004095	1.01083E-06	5.0317E-09	2.99572	up			
CB_011568	9.8191E-07	4.7943E-09	2.936312	up	5111	PCNA	
CB_015742	9.78297E-07	4.7611E-09	2.723111	up	5591	PRKDC	
CB_010255	9.77173E-07	4.7402E-09	3.510492	up	898	CCNE1	
CB_008044	9.67617E-07	4.6361E-09	7.885154	up	6491	STIL	
CB_026240	9.59086E-07	4.5308E-09	13.46312	up	157313	CDCA2	
CB_013220	9.27229E-07	4.3068E-09	7.047509	up	699	BUB1	
CB_013336	9.19037E-07	4.2396E-09	5.509985	up	2146	EZH2	
CB_021096	9.16111E-07	4.2116E-09	2.155042	up	3018	HIST1H2BB	
CB_028710	8.96618E-07	4.0083E-09	5.756786	up	9824	ARHGAP11A	
CB_017520	8.71678E-07	3.8691E-09	10.18413	up	9928	KIF14	
CB_027885	8.10052E-07	3.5314E-09	7.858919	up	11065	UBE2C	
CB_013100	8.03885E-07	3.4918E-09	5.689572	up	9212	AURKB	
CB_018534	7.43241E-07	3.0186E-09	3.096217	up	51659	GINS2	
CB_012458	7.31001E-07	2.9235E-09	2.911765	up	8341	HIST1H2BN	
CB_012473	7.00041E-07	2.7522E-09	3.492342	up	8355	HIST1H3G	
CB_013102	6.91926E-07	2.6664E-09	4.589055	up	9232	PTTG1	
CB_007716	6.91926E-07	2.6764E-09	8.036031	up	220134	SKA1	
CB_014750	6.77757E-07	2.5464E-09	5.064336	up	4173	MCM4	
CB_013912	6.73672E-07	2.5097E-09	5.370962	up	5347	PLK1	
CB_023239	6.54255E-07	2.3751E-09	7.123287	up	891	CCNB1	
CB_016473	6.41128E-07	2.2868E-09	4.067872	up	25788	RAD54B	
CB_011140	6.40493E-07	2.2542E-09	2.037849	up	3329	HSPD1	
CB_012456	6.23174E-07	2.1243E-09	2.378057	up	8339	HIST1H2BG	
CB_025960	6.23174E-07	2.1436E-09	6.014549	up	90381	C15orf42	
CB_024170	6.19382E-07	2.0515E-09	4.41462	up	91687	CENPL	
CB_027594	5.99178E-07	1.9121E-09	7.301668	up	89958	C9orf140	
CB_007784	5.96246E-07	1.8504E-09	4.451874	up	89839	ARHGAP11B	
CB_015930	5.96246E-07	1.8801E-09	11.30298	up	7546	ZIC2	
CB_031343	5.71289E-07	1.6573E-09	7.457562	up			
CB_028399	5.58251E-07	1.5576E-09	4.646009	up	6790	AURKA	
CB_016197	5.45606E-07	1.4814E-09	9.487779	up	22974	TPX2	
CB_019604	5.37405E-07	1.4398E-09	5.930583	up	3070	HELLS	
CB_015337	5.14483E-07	1.3376E-09	4.945448	up	10635	RAD51AP1	
CB_017366	4.55378E-07	1.0251E-09	7.857845	up	9768	KIAA0101	
CB_012453	4.31949E-07	9.3811E-10	2.552186	up	8336	HIST1H2AM	
CB_006880	4.22255E-07	8.8359E-10	8.106777	up	332	BIRC5	
CB_010768	3.9004E-07	7.6053E-10	8.59259	up	983	CDK1	
CB_015743	3.61268E-07	6.6305E-10	2.575408	up	5591	PRKDC	
CB_016591	3.61268E-07	6.5365E-10	4.385835	up	29127	RACGAP1	

CB_012479	3.48328E-07	6.1845E-10	2.575027	up	8359	HIST1H4A	
CB_012477	3.29034E-07	5.6412E-10	4.873899	up	8358	HIST1H3B	
CB_018805	3.27351E-07	5.5021E-10	7.26905	up	51512	GTSE1	
CB_011472	3.27351E-07	5.5526E-10	10.24831	up	4751	NEK2	
CB_012476	3.15644E-07	5.1539E-10	4.398416	up	8357	HIST1H3H	
CB_023768	2.99301E-07	4.6973E-10	4.32682	up	84823	LMNB2	
CB_021091	2.77E-07	4.2594E-10	2.86301	up	3012	HIST1H2AE	
CB_021824	2.75139E-07	4.1872E-10	9.62036	up	53335	BCL11A	
CB_006916	2.56603E-07	3.6204E-10	4.029962	up	387103	CENPW	
CB_008806	2.50105E-07	3.489E-10	6.364032	up	1111	CHEK1	
CB_021098	2.25616E-07	3.0759E-10	2.97991	up	3013	HIST1H2AD	
CB_011391	2.13163E-07	2.6836E-10	6.938131	up	4288	MKI67	
CB_026919	1.88217E-07	2.2112E-10	3.232804	up	286827	TRIM59	
CB_019482	1.88217E-07	2.2075E-10	5.241545	up	55010	C12orf48	
CB_018750	1.43372E-07	1.5228E-10	7.927329	up	1063	CENPF	
CB_008263	1.26199E-07	1.1803E-10	5.990538	up	1763	DNA2	
CB_006044	1.15741E-07	9.7245E-11	6.815811	up	79019	CENPM	
CB_019343	7.96303E-08	5.5134E-11	9.863329	up	55635	DEPDC1	
CB_012460	7.08828E-08	3.9276E-11	5.1909	up	8343	HIST1H2BF	
CB_008075	6.22146E-08	1.8222E-11	12.53551	up	7153	TOP2A	
CB_010744	5.28683E-08	1.1108E-11	6.233105	up	899	CCNF	
CB_021068	3.9125E-08	3.195E-12	4.883566	up	8968	HIST1H3F	
CB_012454	3.9125E-08	5.5821E-12	6.45755	up	8336	HIST1H2AM	
CB_004966	0.04949195	0.02522225	2.22961	down	2162	F13A1	
CB_023527	0.048954878	0.02486664	2.175393	down	84332	DYDC2	
CB_005460	0.04836875	0.02448227	2.029975	down	3479	IGF1	
CB_008439	0.048287634	0.02442706	2.679012	down	12	SERPINA3	
CB_008437	0.047614858	0.02400632	3.169859	down	12	SERPINA3	
CB_005558	0.047508575	0.02393994	2.09637	down	1128	CHRM1	
CB_027392	0.04690362	0.02356297	2.278992	down	2259	FGF14	
CB_014887	0.045955054	0.02297352	2.263831	down	9956	HS3ST2	
CB_020506	0.045903992	0.02294244	2.22433	down	54716	SLC6A20	
CB_024264	0.045618992	0.02276936	2.691372	down	64073	C19orf33	
CB_019040	0.045287143	0.02255419	2.200791	down	8862	APLN	
CB_027165	0.044590846	0.0221339	2.276549	down	283659	PRTG	
CB_017875	0.044574283	0.02212427	2.174215	down	158471	PRUNE2	
CB_010561	0.04414112	0.02183999	2.122815	down	3579	CXCR2	
CB_022680	0.043364402	0.0213505	2.224817	down	80303	EFHD1	
CB_018726	0.042630915	0.02090152	2.067048	down	51454	GULP1	
CB_005647	0.042628516	0.02089967	2.086622	down	2903	GRIN2A	
CB_006403	0.040980253	0.01986484	2.068674	down	390181	OR5AK2	
CB_005000	0.038051706	0.01809472	2.433208	down	2690	GHR	
CB_011084	0.037619844	0.01784848	2.128893	down	3034	HAL	
CB_025616	0.03745469	0.01774676	2.70592	down	219670	ENKUR	
CB_029119	0.036110535	0.01694827	2.007721	down	389799	C9orf171	
CB_005001	0.036006197	0.0168828	2.491184	down	2690	GHR	
CB_018743	0.03579775	0.01675737	2.1453	down	5625	PRODH	
CB_026880	0.032920357	0.01504512	3.844723	down	9576	SPAG6	
CB_028268	0.031911865	0.01443852	2.263398	down	285498	RNF212	
CB_027523	0.03177427	0.01435914	2.221178	down	253935	ANGPTL5	
CB_024501	0.03141405	0.01415463	3.214203	down	83659	TEKT1	
CB_009662	0.03078538	0.01378896	2.271682	down	340273	ABCB5	
CB_025232	0.030547388	0.0136541	2.277198	down	143241	DYDC1	
CB_011354	0.030495048	0.01362061	2.714312	down	4133	MAP2	
CB_029405	0.029553387	0.01309888	2.214103	down	11223	MST1P9	
CB_005511	0.028606044	0.0125468	3.319323	down	186	AGTR2	

CB_028802	0.028231552	0.01231978	2.066892	down	6296	ACSM3	
CB_006745	0.027883802	0.01212273	2.243129	down	10718	NRG3	
CB_010800	0.027662616	0.012002	2.019467	down	1089	CEACAM4	
CB_006824	0.02707086	0.01167402	2.335604	down	388633	LDLRAD1	
CB_010524	0.026492564	0.01134778	2.477446	down	3036	HAS1	
CB_008795	0.026434429	0.01131407	2.00683	down	154796	AMOT	
CB_005061	0.02627246	0.01122893	2.494785	down	3815	KIT	
CB_027727	0.02619407	0.01118545	2.505549	down	353189	SLCO4C1	
CB_011700	0.026009457	0.01108601	2.121411	down	5577	PRKAR2B	
CB_010708	0.025662996	0.01089562	2.149685	down	671	BPI	
CB_027529	0.025573697	0.01084878	2.578071	down	345275	HSD17B13	
CB_013848	0.025344452	0.01072381	2.150829	down	3382	ICA1	
CB_017115	0.025155464	0.0106275	2.774462	down	27284	SULT1B1	
CB_017505	0.02435598	0.01018085	2.233841	down	9914	ATP2C2	
CB_027017	0.024248844	0.01011877	2.216199	down	165530	CLEC4F	
CB_023833	0.024208354	0.01009612	2.012397	down	54768	HYDIN	
CB_012217	0.024156457	0.0100695	2.121073	down	7079	TIMP4	
CB_019009	0.023763614	0.00986317	2.110193	down	8863	PER3	
CB_011804	0.023699017	0.00982735	2.083865	down	5789	PTPRD	
CB_024290	0.02341244	0.00967511	2.504265	down	79937	CNTNAP3	
CB_020156	0.02327631	0.00960594	2.000257	down	56129	PCDHB7	
CB_022877	0.022932995	0.00941736	2.309398	down	81578	COL21A1	
CB_021914	0.022704698	0.00928314	2.179533	down	65987	KCTD14	
CB_009725	0.022517495	0.00918602	2.375377	down	29951	PDZRN4	
CB_003319	0.02243263	0.00913756	2.171177	down			
CB_013123	0.022416078	0.0091289	2.145002	down	9332	CD163	
CB_008313	0.022193916	0.00901077	2.276197	down	22996	TTC39A	
CB_008479	0.022070734	0.00894466	2.299746	down	3815	KIT	
CB_009972	0.022016399	0.00891496	2.046925	down	83543	AIF1L	
CB_010632	0.021910679	0.00886069	2.3145	down	347	APOD	
CB_001277	0.021587325	0.00868886	2.442208	down			
CB_025617	0.02124449	0.00850541	2.522623	down	219670	ENKUR	
CB_031105	0.021075549	0.00841338	2.019878	down	283481	LOC283481	
CB_013125	0.020739036	0.00823934	2.176096	down	9340	GLP2R	
CB_008033	0.020675702	0.00820787	2.868807	down	401551	WDR38	
CB_013860	0.020379527	0.00805454	2.12503	down	3752	KCND3	
CB_017622	0.020335756	0.00802682	2.017957	down	22979	EFR3B	
CB_017091	0.019461192	0.00757754	2.336707	down	53832	IL20RA	
CB_022842	0.019341165	0.00751099	2.831615	down	81501	TM7SF4	
CB_001526	0.019294418	0.0074861	2.75104	down			
CB_022081	0.019135473	0.00740259	2.046828	down	79192	IRX1	
CB_011815	0.01892666	0.00729271	2.792615	down	5799	PTPRN2	
CB_010512	0.018476155	0.00707109	2.308635	down	2919	CXCL1	
CB_016887	0.018207045	0.00693608	2.151529	down	3803	KIR2DL2	
CB_025409	0.01788944	0.00678132	2.75999	down	116372	LYPD1	
CB_006998	0.017851003	0.00676307	2.347421	down	257177	C1orf192	
CB_009674	0.01774191	0.00670711	2.065297	down	730112	FAM166B	
CB_011214	0.017728768	0.00669961	3.57852	down	3739	KCNA4	
CB_000450	0.017392144	0.00653436	2.214037	down	100131176	LOC100131176	
CB_001827	0.017354006	0.00651288	3.750387	down	80763	C12orf39	
CB_024697	0.017268589	0.00646741	2.37293	down	140733	MACROD2	
CB_016082	0.017216781	0.00644091	2.893144	down	4311	MME	
CB_023918	0.017000435	0.00633302	2.021805	down	85016	C11orf70	
CB_016670	0.016988125	0.00632709	2.532478	down	26577	PCOLCE2	
CB_027321	0.016855907	0.00626341	2.015994	down	1379	CR1L	
CB_009716	0.016522638	0.00610762	2.033688	down	401207	FLJ44606	

CB_027256	0.016284488	0.00598706	2.129178	down	257019	FRMD3	
CB_021572	0.01621201	0.00595168	2.384419	down	63951	DMRTA1	
CB_024602	0.015977884	0.00583508	2.282441	down	4632	MYL1	
CB_006740	0.015888238	0.00579323	2.04762	down	79822	ARHGAP28	
CB_024814	0.015678963	0.00569067	2.126823	down	64407	RGS18	
CB_005307	0.015672427	0.005687	2.433391	down	6401	SELE	
CB_026648	0.015560274	0.00563397	2.774576	down	120065	OR5P2	
CB_014101	0.015466652	0.00558596	2.231191	down	2066	ERBB4	
CB_023227	0.015453474	0.0055784	2.096525	down	83894	TTC29	
CB_024907	0.015395015	0.00555062	2.675733	down	171024	SYNPO2	
CB_016045	0.015362322	0.0055351	2.354279	down	10687	PNMA2	
CB_014099	0.015354891	0.0055315	2.020151	down	2042	EPHA3	
CB_005938	0.015211582	0.00545805	2.194801	down	80760	ITIH5	
CB_024524	0.014955016	0.00533849	2.134373	down	117245	HRASLS5	
CB_026225	0.014792044	0.00526202	2.336516	down	153643	FAM81B	
CB_008720	0.014724094	0.00523061	2.500385	down	374864	C18orf34	
CB_022686	0.014677555	0.00520989	2.249172	down	80310	PDGFD	
CB_002414	0.014586805	0.00516635	2.709123	down			
CB_030363	0.014492312	0.00512051	2.028972	down	200058	FLJ23867	
CB_010380	0.014390033	0.00507121	2.134584	down	1768	DNAH6	
CB_022272	0.014356858	0.00505532	3.135769	down	79739	TTLL7	
CB_022372	0.014281541	0.00502196	2.18808	down	79844	ZDHHC11	
CB_023226	0.014222748	0.00499389	2.460606	down	83894	TTC29	
CB_004857	0.014078015	0.00493303	2.441583	down	212	ALAS2	
CB_021881	0.01404642	0.00491973	2.251983	down	6614	SIGLEC1	
CB_002524	0.01402442	0.00490736	2.369713	down			
CB_025920	0.013934341	0.00486347	2.156941	down	56477	CCL28	
CB_017620	0.013655162	0.00473334	2.89517	down	22979	EFR3B	
CB_022080	0.013552464	0.00468657	2.095407	down	79191	IRX3	
CB_019818	0.013514601	0.00466598	2.019394	down	55259	CASC1	
CB_012720	0.013458153	0.00464045	2.003401	down	8727	CTNNAL1	
CB_027599	0.013381437	0.004606	2.976035	down	123872	LRRC50	
CB_020154	0.01335477	0.00459195	2.170304	down	56131	PCDHB4	
CB_027816	0.013307562	0.00457198	2.130254	down	148808	MFSD4	
CB_005574	0.013269386	0.00455241	3.085668	down	1440	CSF3	
CB_022771	0.013231502	0.004533	2.467392	down	283987	C17orf28	
CB_023832	0.01317231	0.00450457	2.681546	down	54768	HYDIN	
CB_023372	0.013120918	0.00447894	2.826757	down	84152	PPP1R1B	
CB_026119	0.01309121	0.00446397	2.026782	down	138311	FAM69B	
CB_013334	0.013037624	0.00443661	2.319738	down	2119	ETV5	
CB_022632	0.012996248	0.00441738	2.200193	down	80217	C10orf79	
CB_026081	0.0129441	0.00439225	2.687697	down	130733	TMEM178	
CB_022271	0.0129441	0.00439227	2.861902	down	79739	TTLL7	
CB_026028	0.012915973	0.00437904	2.301554	down	123624	AGBL1	
CB_021229	0.012820454	0.00433466	2.336635	down	58473	PLEKHB1	
CB_017067	0.012793154	0.00432279	3.077363	down	27092	CACNG4	
CB_020153	0.012766333	0.0043113	2.589991	down	56133	PCDHB2	
CB_025810	0.012753683	0.00430602	3.833849	down	143662	MUC15	
CB_004114	0.012735379	0.004296	2.038797	down			
CB_013287	0.012463344	0.00417223	2.034156	down	1827	RCAN1	
CB_010435	0.012357124	0.00412323	2.182584	down	2012	EMP1	
CB_006649	0.012212374	0.00405813	2.20691	down	6542	SLC7A2	
CB_028405	0.012178811	0.00404319	2.080279	down	127845	GOLT1A	
CB_002735	0.012138824	0.00402472	2.070916	down			
CB_010754	0.012132383	0.00402105	2.543979	down	933	CD22	
CB_022732	0.012130584	0.00402026	2.706585	down	80736	SLC44A4	

CB_027949	0.012062541	0.00399063	3.062409	down	144809	C13orf30	
CB_030564	0.012011662	0.00396733	3.900749	down	150147	C21orf128	
CB_018889	0.012006084	0.00396491	2.333458	down	51348	KLRF1	
CB_024494	0.011865182	0.00390038	3.299258	down	83648	FAM167A	
CB_019731	0.011791904	0.00386604	2.200262	down	55214	LEPREL1	
CB_014116	0.011603975	0.00378264	2.546592	down	2303	FOXC2	
CB_007846	0.01154381	0.00375691	2.146875	down	122060	SLAIN1	
CB_022542	0.011378623	0.00368537	2.683921	down	80059	LRRTM4	
CB_016396	0.011368713	0.00368126	2.017209	down	3809	KIR2DS4	
CB_018727	0.011320075	0.0036603	2.295528	down	51454	GULP1	
CB_018066	0.011233574	0.00362289	2.235821	down	25891	PAMR1	
CB_031670	0.011043111	0.00354221	2.20779	down	100510454	LOC100510454	
CB_013541	0.011003826	0.00352403	2.137897	down	8876	VNN1	
CB_005749	0.010940732	0.00349723	2.032481	down	5345	SERPINF2	
CB_012000	0.01087647	0.00346979	2.544138	down	6425	SFRP5	
CB_025049	0.010875396	0.00346911	2.885811	down	112703	FAM71E1	
CB_010141	0.010850597	0.00345793	2.202454	down	54768	HYDIN	
CB_016080	0.010731149	0.00340863	2.93068	down	4311	MME	
CB_021569	0.010649211	0.00337298	2.042632	down	64122	FN3K	
CB_027081	0.010646869	0.00337207	2.324938	down	284076	TTLL6	
CB_016935	0.010576501	0.00334308	2.540459	down	11086	ADAM29	
CB_009580	0.010485946	0.00329908	2.244447	down	290	ANPEP	
CB_026913	0.010410504	0.0032685	2.750714	down	219681	ARMC3	
CB_015809	0.010334798	0.00323588	2.133045	down	9501	RPH3AL	
CB_003308	0.01033317	0.00323472	2.067305	down			
CB_008438	0.010285281	0.00321403	2.838236	down	12	SERPINA3	
CB_020567	0.010044321	0.003115	2.693332	down	57221	KIAA1244	
CB_030740	0.010024974	0.00310661	3.290925	down	55350	VNN3	
CB_009807	0.009830713	0.00302257	2.128976	down	783	CACNB2	
CB_011096	0.009796732	0.00300777	2.179103	down	3109	HLA-DMB	
CB_011538	0.009756587	0.00299185	2.095832	down	5027	P2RX7	
CB_014792	0.009746679	0.00298669	2.03736	down	4496	MT1H	
CB_011965	0.009717361	0.00297432	2.791796	down	6366	CCL21	
CB_005573	0.009713638	0.00297271	3.074064	down	1437	CSF2	
CB_014922	0.009684822	0.00296186	2.107173	down	3268	AGFG2	
CB_010798	0.009674571	0.00295723	2.922033	down	1084	CEACAM3	
CB_013854	0.009672035	0.002956	3.429457	down	3745	KCNB1	
CB_020127	0.009662556	0.00295203	2.420052	down	55966	AJAP1	
CB_028434	0.009477936	0.0028772	4.946384	down	284340	CXCL17	
CB_013495	0.009444799	0.00286296	2.627483	down	7103	TSPAN8	
CB_004902	0.009382721	0.00283798	2.590904	down	718	C3	
CB_020144	0.009330854	0.00281416	2.19185	down	56171	DNAH7	
CB_012955	0.009280994	0.00279411	2.019261	down	828	CAPS	
CB_005618	0.009253932	0.00278098	2.133117	down	2350	FOLR2	
CB_006004	0.009169473	0.00274775	2.143166	down	721	C4B	
CB_015898	0.009139384	0.00273453	3.38028	down	2981	GUCA2B	
CB_028309	0.009120153	0.00272747	2.239521	down	256380	SCML4	
CB_014463	0.009077296	0.00270703	2.160639	down	4818	NKG7	
CB_027373	0.009038929	0.00269222	2.510938	down	161753	ODF3L1	
CB_017572	0.008692465	0.00255201	3.936353	down	22866	CNKSR2	
CB_030392	0.008643095	0.00253271	2.495766	down	255082	CASC2	
CB_024372	0.008558383	0.00249812	2.555017	down	114792	KLHL32	
CB_011832	0.008489955	0.00247077	2.893471	down	5858	PZP	
CB_009921	0.008488765	0.00247002	2.400452	down	387638	C10orf113	
CB_026481	0.008462958	0.00245872	2.394199	down	6660	SOX5	
CB_016099	0.008421046	0.00244321	6.586608	down	1755	DMBT1	

CB_027118	0.008415589	0.00243992	2.114659	down	285489	DOK7	
CB_016081	0.008359419	0.00241706	3.102894	down	4311	MME	
CB_017883	0.008334271	0.00240715	2.320365	down	23284	LPHN3	
CB_024000	0.008334262	0.00240643	2.604307	down	4135	MAP6	
CB_002024	0.008181481	0.00234549	2.318942	down			
CB_005306	0.008076725	0.00230467	6.246205	down	6401	SELE	
CB_027872	0.008001034	0.00227508	2.284342	down	135112	NCOA7	
CB_015756	0.007958806	0.00225967	2.531318	down	6323	SCN1A	
CB_004411	0.007953214	0.00225758	2.226147	down			
CB_030252	0.007893939	0.00223487	3.246494	down	26238	C6orf123	
CB_022915	0.007802847	0.00219721	2.042092	down	81615	TMEM163	
CB_030318	0.00779902	0.002196	3.470676	down	283392	LOC283392	
CB_011711	0.007786796	0.00219096	2.938838	down	5596	MAPK4	
CB_002692	0.00776022	0.00218056	3.353988	down			
CB_025381	0.007745275	0.00217326	2.126628	down	90527	DUOXA1	
CB_029097	0.007733463	0.00216786	3.344246	down	388531	RGS9BP	
CB_022311	0.007714238	0.0021599	2.101018	down	79782	LRRC31	
CB_012887	0.007666076	0.00214055	3.290218	down	9023	CH25H	
CB_025394	0.007590116	0.00211059	2.789929	down	92291	CAPN13	
CB_017882	0.007561036	0.00209961	2.257815	down	23284	LPHN3	
CB_006329	0.007503917	0.00207806	2.139249	down	390072	OR52N4	
CB_000354	0.007429822	0.00204721	2.091295	down	100131581	LOC100131581	
CB_020304	0.007429003	0.00204659	2.145637	down	54621	VSIG10	
CB_024857	0.007403655	0.00203715	2.339817	down	2204	FCAR	
CB_005214	0.007330464	0.00200852	2.644546	down	7056	THBD	
CB_022633	0.007272047	0.00198722	2.990968	down	80217	C10orf79	
CB_007900	0.007246989	0.00197669	2.116843	down	83988	NCALD	
CB_026339	0.007225723	0.00196952	2.308027	down	202309	GAPT	
CB_021432	0.007202727	0.00196131	2.371242	down	6581	SLC22A3	
CB_020296	0.007198444	0.00195946	2.429405	down	54567	DLL4	
CB_016105	0.007181745	0.00195236	2.279821	down	9940	DLEC1	
CB_024878	0.007123327	0.00193007	2.396446	down	7273	TTN	
CB_012031	0.007067355	0.00190921	2.171452	down	6539	SLC6A12	
CB_007801	0.007066547	0.00190877	2.344434	down	129049	SGSM1	
CB_017856	0.007023736	0.00189331	2.220642	down	221981	THSD7A	
CB_027625	0.006995707	0.00188242	3.500541	down	201625	DNAH12	
CB_020362	0.006964501	0.00187136	2.347276	down	56253	CRTAM	
CB_009497	0.006939573	0.00186226	2.185756	down	125336	LOXHD1	
CB_027910	0.006917279	0.00185299	2.544633	down	339403	RXFP4	
CB_003258	0.006896747	0.00184508	2.21966	down	4916	NTRK3	
CB_011149	0.00688203	0.00183874	2.417469	down	3397	ID1	
CB_013090	0.006856313	0.00182837	2.416424	down	9099	USP2	
CB_001047	0.00678839	0.00180415	2.237598	down	286058	LOC286058	
CB_005380	0.006778942	0.00180056	2.34272	down	3949	LDLR	
CB_023179	0.006699016	0.00177052	2.137361	down	114898	C1QTNF2	
CB_016084	0.006697718	0.00176932	2.319213	down	6622	SNCA	
CB_020859	0.006683297	0.00176371	2.548284	down	57538	ALPK3	
CB_004861	0.006596097	0.00173405	2.17575	down	229	ALDOB	
CB_006741	0.006563091	0.0017218	2.127893	down	79822	ARHGAP28	
CB_014133	0.00646347	0.00168511	2.38601	down	2702	GJA5	
CB_010401	0.006460893	0.00168413	2.54812	down	1837	DTNA	
CB_011939	0.006442647	0.00167774	2.195103	down	6281	S100A10	
CB_028156	0.006428276	0.00167277	3.009962	down	3249	HPN	
CB_007009	0.006383174	0.00165709	2.448211	down	388610	TRNP1	
CB_005781	0.006325361	0.00163807	2.957884	down	5743	PTGS2	
CB_022314	0.006207401	0.00159424	4.152594	down	79785	RERGL	

CB_026661	0.006163975	0.0015782	2.642629	down	197257	LDHD	
CB_026230	0.006150795	0.00157356	2.095546	down	154075	SAMD3	
CB_030807	0.006142305	0.00157031	2.486402	down	267010	RNU12	
CB_019860	0.00612237	0.00156376	2.000907	down	55303	GIMAP4	
CB_010471	0.00604615	0.00153653	2.607276	down	2318	FLNC	
CB_010797	0.006045459	0.00153616	3.194908	down	1084	CEACAM3	
CB_026796	0.00602771	0.00152879	2.85965	down	3772	KCNJ15	
CB_005709	0.005917443	0.00148975	2.360464	down	3762	KCNJ5	
CB_006587	0.005895696	0.00148119	3.35005	down	389118	CDHR4	
CB_004903	0.005886148	0.00147837	2.375798	down	718	C3	
CB_021323	0.005874845	0.00147456	2.300791	down	9848	MFAP3L	
CB_011170	0.005861724	0.00146932	2.477651	down	3575	IL7R	
CB_020463	0.005815666	0.00145362	2.444163	down	56920	SEMA3G	
CB_030654	0.005814516	0.00145324	2.092779	down	440925	LOC440925	
CB_005249	0.005808487	0.00145099	2.25646	down	1536	CYBB	
CB_016021	0.005795672	0.00144623	3.202688	down	11254	SLC6A14	
CB_010377	0.005739904	0.00142561	2.944169	down	1767	DNAH5	
CB_024732	0.005685393	0.00140636	3.066339	down	1602	DACH1	
CB_010267	0.005667255	0.00139927	2.152633	down	968	CD68	
CB_004412	0.005667158	0.00139916	2.065783	down			
CB_001296	0.005651668	0.00139426	2.083509	down	399875	LOC399875	
CB_026582	0.005600254	0.00137865	3.097861	down	256076	COL6A5	
CB_023099	0.005567205	0.00136619	2.189672	down	83641	FAM107B	
CB_010975	0.005556864	0.00136241	2.621684	down	2247	FGF2	
CB_017352	0.005555332	0.00136186	2.411913	down	9750	FAM65B	
CB_012903	0.005540473	0.00135681	2.594113	down	9172	MYOM2	
CB_017777	0.005527665	0.00135254	2.285516	down	23150	FRMD4B	
CB_020566	0.005522436	0.00135084	2.875016	down	57221	KIAA1244	
CB_015255	0.005521067	0.00135031	2.442909	down	10529	NEBL	
CB_024493	0.00551847	0.00134906	3.434769	down	83648	FAM167A	
CB_024966	0.005464063	0.00133204	2.489158	down	168507	PKD1L1	
CB_011102	0.005452536	0.00132819	2.116181	down	3115	HLA-DPB1	
CB_018710	0.005449283	0.00132705	2.164156	down	51725	FBXO40	
CB_005889	0.005448325	0.00132673	2.038024	down	6876	TAGLN	
CB_005472	0.005437273	0.00132232	2.06872	down	3684	ITGAM	
CB_019189	0.005420638	0.00131681	10.42473	down	55600	ITLN1	
CB_007899	0.005405386	0.00131109	2.614756	down	467	ATF3	
CB_003030	0.005344556	0.0012929	2.224385	down			
CB_026405	0.005290212	0.00127495	2.077117	down	254268	AKNAD1	
CB_017651	0.005221617	0.00125328	2.172204	down	23007	PLCH1	
CB_022413	0.005170838	0.00123694	2.326242	down	79887	PLBD1	
CB_023098	0.00510795	0.00121462	2.083551	down	83641	FAM107B	
CB_016100	0.005040665	0.00119159	6.800356	down	1755	DMBT1	
CB_003997	0.004977459	0.00117096	2.028256	down			
CB_016131	0.004970007	0.00116756	2.44075	down	22914	KLRK1	
CB_005501	0.004835579	0.00112356	2.172969	down	135	ADORA2A	
CB_012200	0.004826349	0.00112067	2.736583	down	7042	TGFB2	
CB_021369	0.004816049	0.00111767	2.847632	down	2243	FGA	
CB_025610	0.004815784	0.00111728	2.296857	down	203102	ADAM32	
CB_019216	0.004798341	0.00111163	2.608832	down	55607	PPP1R9A	
CB_014788	0.004783921	0.00110716	2.040883	down	4490	MT1B	
CB_023772	0.004763888	0.00110078	3.451235	down	84830	C6orf105	
CB_029065	0.004727527	0.00108848	2.14138	down	285440	CYP4V2	
CB_018168	0.004715527	0.00108505	2.109677	down	26032	SUSD5	
CB_024525	0.004713162	0.0010842	2.519016	down	117248	GALNTL2	
CB_010862	0.004691677	0.00107711	3.057998	down	1410	CRYAB	

CB_007467	0.004674894	0.00107236	2.189677	down	224	ALDH3A2	
CB_000106	0.004667949	0.0010701	2.842215	down			
CB_026145	0.004616546	0.00105334	2.480543	down	145581	LRFN5	
CB_015848	0.004609868	0.00105124	2.27588	down	11076	TPPP	
CB_012590	0.004604297	0.00104931	2.050605	down	8530	CST7	
CB_022282	0.004600422	0.00104815	2.124839	down	79745	CLIP4	
CB_009487	0.004574884	0.00104028	2.206786	down	400713	ZNF880	
CB_023348	0.004558875	0.0010357	2.821291	down	92126	DSEL	
CB_004267	0.004541177	0.00102967	2.377295	down			
CB_019912	0.004530896	0.00102633	2.032449	down	55340	GIMAP5	
CB_010594	0.004530608	0.00102619	2.036381	down	72	ACTG2	
CB_028922	0.004525422	0.00102452	2.578399	down	2494	NR5A2	
CB_028992	0.004470917	0.00100743	2.243872	down	79819	WDR78	
CB_011649	0.00446338	0.00100481	3.213783	down	5409	PNMT	
CB_020435	0.004463151	0.00100469	2.048188	down	56895	AGPAT4	
CB_010755	0.004433592	0.00099579	2.076878	down	945	CD33	
CB_005726	0.004431365	0.00099493	3.79677	down	4886	NPY1R	
CB_006783	0.004391721	0.00098311	2.041898	down	340554	ZC3H12B	
CB_010722	0.00434276	0.00096926	2.808845	down	759	CA1	
CB_019072	0.004340519	0.0009683	2.219058	down	10458	BAIAP2	
CB_030817	0.004314073	0.00096032	2.216202	down	100130000	LOC100130000	
CB_019832	0.004305259	0.00095768	2.111186	down	55277	FGGY	
CB_030326	0.0042858	0.0009516	2.165099	down	339483	MTMR9LP	
CB_011420	0.004242581	0.00093748	3.393331	down	4481	MSR1	
CB_026027	0.004217371	0.00092957	2.478403	down	123624	AGBL1	
CB_009848	0.004200399	0.0009241	2.545865	down	11155	LDB3	
CB_017621	0.004190053	0.00092076	3.295448	down	22979	EFR3B	
CB_009695	0.004177131	0.0009165	5.328809	down	389177	TMEM212	
CB_001977	0.004158393	0.00091083	2.028066	down			
CB_004710	0.00415389	0.00090886	2.203316	down			
CB_012792	0.004104439	0.00089492	2.073799	down	8821	INPP4B	
CB_014938	0.00409096	0.00089055	3.007669	down	10391	CORO2B	
CB_029187	0.004056996	0.00088071	2.237777	down	203859	ANO5	
CB_018095	0.004000188	0.00086344	2.144393	down	25925	ZNF521	
CB_029764	0.003985976	0.0008598	2.748684	down	572558	LOC572558	
CB_003270	0.003978262	0.00085753	2.077418	down			
CB_015751	0.003942438	0.00084791	2.730849	down	6096	RORB	
CB_014921	0.003938373	0.00084635	2.051987	down	10346	TRIM22	
CB_016889	0.003937536	0.00084609	2.204504	down	4071	TM4SF1	
CB_022371	0.003907018	0.00083633	2.173633	down	79844	ZDHHC11	
CB_031746	0.003901392	0.00083482	2.030479	down			
CB_021553	0.00388503	0.00083076	2.078562	down	64098	PARVG	
CB_011423	0.003819336	0.00081138	2.179991	down	4487	MSX1	
CB_013114	0.003798381	0.00080507	2.183052	down	9308	CD83	
CB_025625	0.003789609	0.00080234	2.225353	down	220004	C11orf66	
CB_014621	0.003786328	0.00080119	2.680864	down	10157	AASS	
CB_010903	0.003778229	0.00079876	2.726651	down	1803	DPP4	
CB_021290	0.003772084	0.00079668	2.021603	down	59084	ENPP5	
CB_013833	0.003767193	0.00079517	2.676566	down	2115	ETV1	
CB_030870	0.003763454	0.00079394	5.272387	down	390732	CES1P2	
CB_009577	0.00376155	0.00079326	3.194486	down	287	ANK2	
CB_023656	0.003750578	0.00078994	3.467247	down	84631	SLITRK2	
CB_021261	0.003750535	0.00078981	5.612428	down	58511	DNASE2B	
CB_020568	0.00374611	0.00078858	3.232446	down	57221	KIAA1244	
CB_013002	0.003725284	0.00078195	3.580418	down	2170	FABP3	
CB_017850	0.003717928	0.00077959	3.63982	down	23242	COBL	

CB_023705	0.003705498	0.00077595	2.297805	down	84689	MS4A14	
CB_005416	0.003677792	0.00076767	2.228447	down	3047	HBG1	
CB_010230	0.003660642	0.00076363	2.638466	down	652	BMP4	
CB_011188	0.003659274	0.00076299	2.388547	down	3675	ITGA3	
CB_019499	0.00364114	0.00075823	2.715902	down	55026	FAM70A	
CB_016574	0.003625475	0.00075359	3.528734	down	10891	PPARGC1A	
CB_026552	0.00361517	0.00075037	2.516103	down	162282	ANKFN1	
CB_007805	0.003598595	0.00074555	2.240853	down	2852	GPER	
CB_007850	0.003580753	0.00074021	2.252317	down	3360	HTR4	
CB_024975	0.003578102	0.00073951	2.271079	down	171389	NLRP6	
CB_028310	0.003538826	0.0007289	2.213795	down	256380	SCML4	
CB_001960	0.003529036	0.00072594	3.780686	down			
CB_017849	0.003528772	0.00072571	3.822138	down	23242	COBL	
CB_026684	0.003516519	0.00072213	2.277914	down	123041	SLC24A4	
CB_025606	0.003509167	0.0007204	3.073079	down	202374	STK32A	
CB_008828	0.003503663	0.00071894	2.286112	down	117	ADCYAP1R1	
CB_023188	0.003493502	0.00071652	2.676858	down	83853	ROPN1L	
CB_028281	0.00348886	0.00071521	2.220079	down	64581	CLEC7A	
CB_024696	0.00348886	0.00071523	2.633038	down	140733	MACROD2	
CB_004256	0.003484388	0.00071404	3.22281	down			
CB_028871	0.003434259	0.00069986	2.035913	down	81704	DOCK8	
CB_001894	0.003430813	0.00069882	2.190424	down	168667	BMPER	
CB_014106	0.003418722	0.00069522	2.480688	down	2114	ETS2	
CB_021876	0.003418483	0.00069512	2.373529	down	10129	FRY	
CB_004730	0.003418221	0.00069496	2.703112	down			
CB_019267	0.003396116	0.00068885	3.476413	down	54848	ARHGEF38	
CB_011109	0.003388349	0.00068679	2.034458	down	3127	HLA-DRB5	
CB_016888	0.003374048	0.00068307	2.08411	down	4071	TM4SF1	
CB_014083	0.003374048	0.0006832	3.170643	down	1668	DEFA3	
CB_008527	0.003362825	0.00068012	19.13995	down	729238	SFTPA2	
CB_012897	0.003354259	0.00067781	2.388314	down	9034	CCRL2	
CB_015254	0.003336429	0.0006729	3.473877	down	10529	NEBL	
CB_028727	0.003321001	0.00066861	2.102603	down	22891	ZNF365	
CB_011991	0.003314947	0.00066715	2.12113	down	6415	SEPW1	
CB_026748	0.003308496	0.00066541	2.377007	down	139378	GPR112	
CB_021225	0.003293626	0.00066122	2.042276	down	57835	SLC4A5	
CB_024535	0.003287572	0.00065954	2.049455	down	56062	KLHL4	
CB_030500	0.003272147	0.00065504	2.716695	down	285759	FLJ34503	
CB_010196	0.00325233	0.00064917	2.124914	down	60401	EDA2R	
CB_007395	0.003219544	0.00064002	2.110347	down	394	ARHGAP5	
CB_007550	0.00321367	0.00063834	2.726594	down	6387	CXCL12	
CB_013232	0.003196486	0.00063392	2.881913	down	862	RUNX1T1	
CB_005512	0.00319506	0.00063348	6.613552	down	186	AGTR2	
CB_016619	0.003185145	0.0006301	2.316896	down	29799	YPEL1	
CB_026649	0.003170142	0.00062608	3.158382	down	120066	OR5P3	
CB_028293	0.003159561	0.00062287	2.271387	down	11162	NUDT6	
CB_030703	0.003159561	0.00062299	2.656868	down	388815	C21orf34	
CB_017817	0.003134435	0.00061604	2.731523	down	23209	MLC1	
CB_005706	0.003130803	0.00061513	2.264802	down	3687	ITGAX	
CB_011313	0.003124305	0.00061341	2.029069	down	4035	LRP1	
CB_027443	0.003117224	0.00061118	2.336932	down	53829	P2RY13	
CB_026152	0.003090889	0.0006042	2.073331	down	146434	ZNF597	
CB_002689	0.003077238	0.00060051	2.86073	down			
CB_019599	0.003064053	0.000597	2.552419	down	55698	RADIL	
CB_010067	0.003053062	0.00059429	2.099015	down	85016	C11orf70	
CB_030191	0.00303864	0.00059086	2.029438	down	84850	C9orf70	

CB_009893	0.00302776	0.00058826	2.365145	down	79649	MAP7D3	
CB_030545	0.003026937	0.00058799	2.023488	down	200197	C1orf126	
CB_011966	0.003025882	0.00058756	2.877542	down	6366	CCL21	
CB_026815	0.003020893	0.00058602	2.387031	down	9099	USP2	
CB_008387	0.003010409	0.00058298	2.257837	down	728621	CCDC30	
CB_017652	0.003009095	0.00058251	2.647878	down	23007	PLCH1	
CB_005931	0.00300555	0.00058128	2.300023	down	414899	BLID	
CB_023704	0.002987023	0.00057613	2.205964	down	84689	MS4A14	
CB_023794	0.002978158	0.00057387	2.390063	down	84870	RSPO3	
CB_016860	0.00296508	0.00057016	2.048134	down	29094	HSPC159	
CB_009391	0.002957165	0.00056817	2.42343	down	374462	PTPRQ	
CB_026776	0.002941069	0.00056408	2.005329	down	9462	RASAL2	
CB_004913	0.002909269	0.00055579	2.81656	down	1028	CDKN1C	
CB_009368	0.002907387	0.00055534	2.336616	down	23327	NEDD4L	
CB_006481	0.0029005	0.00055329	3.130405	down	785	CACNB4	
CB_021821	0.0029005	0.00055333	3.229087	down	64881	PCDH20	
CB_023152	0.002873651	0.00054598	2.650677	down	8325	FZD8	
CB_001538	0.002851383	0.00054043	2.271161	down	5265	SERPINA1	
CB_002659	0.002833952	0.00053538	2.2637	down			
CB_003639	0.00283351	0.00053521	2.140114	down			
CB_011275	0.002822072	0.0005326	2.122701	down	3903	LAIR1	
CB_024733	0.002798845	0.00052635	2.67862	down	1602	DACH1	
CB_005038	0.00278863	0.0005239	2.58914	down	3383	ICAM1	
CB_001921	0.002787132	0.00052344	2.750999	down	257019	FRMD3	
CB_006523	0.00278604	0.00052315	2.144717	down	114789	SLC25A25	
CB_015917	0.002771646	0.00051939	2.129809	down	7376	NR1H2	
CB_029434	0.002767674	0.00051825	2.74953	down	163720	CYP4Z2P	
CB_002555	0.002761674	0.00051616	2.13585	down			
CB_004904	0.002753515	0.00051412	3.148567	down	729	C6	
CB_020486	0.002742325	0.00051129	2.155413	down	56944	OLFML3	
CB_012930	0.00271086	0.00050336	2.571205	down	1756	DMD	
CB_021258	0.002710129	0.00050305	3.813089	down	59277	NTN4	
CB_011458	0.002690855	0.00049853	6.615272	down	4680	CEACAM6	
CB_018130	0.00268976	0.00049827	2.219502	down	25976	TIPARP	
CB_019077	0.002681995	0.00049608	2.480692	down	50613	UBQLN3	
CB_027843	0.002674671	0.00049414	3.992593	down	163782	KANK4	
CB_028358	0.002670147	0.00049279	2.232446	down	2934	GSN	
CB_013156	0.002646636	0.00048686	2.63902	down	9481	SLC25A27	
CB_019232	0.002645479	0.0004866	2.499614	down	140803	TRPM6	
CB_008617	0.002642715	0.00048593	2.098436	down	129852	C2orf73	
CB_029343	0.00261292	0.00047801	2.185081	down	8418	CMAH	
CB_024276	0.002605808	0.0004757	2.094867	down	3113	HLA-DPA1	
CB_003530	0.002593641	0.00047296	3.234488	down			
CB_007081	0.002585706	0.00047081	2.300489	down	10873	ME3	
CB_017579	0.002582288	0.00046982	2.143056	down	22874	PLEKHA6	
CB_010703	0.002576836	0.00046842	2.739791	down	660	BMX	
CB_019784	0.002574144	0.00046773	2.363691	down	55244	SLC47A1	
CB_026795	0.002551692	0.00046191	4.123378	down	3772	KCNJ15	
CB_005399	0.002551169	0.00046177	14.4483	down	6439	SFTPB	
CB_023153	0.002545619	0.00046029	2.844156	down	56135	PCDHAC1	
CB_003290	0.002534133	0.00045745	2.163903	down	514	ATP5E	
CB_010873	0.002533752	0.00045734	2.681747	down	1490	CTGF	
CB_015737	0.002476834	0.00044369	2.196848	down	3204	HOXA7	
CB_022370	0.002475409	0.0004432	2.068856	down	79844	ZDHHC11	
CB_008893	0.002472646	0.00044258	2.335501	down	4093	SMAD9	
CB_020873	0.0024701	0.00044199	2.664333	down	57556	SEMA6A	

CB_025468	0.00246881	0.00044162	2.123161	down	137835	TMEM71	
CB_026324	0.002464464	0.00044059	2.048612	down	200132	TCTEX1D1	
CB_025081	0.002462693	0.00043984	2.410312	down	115207	KCTD12	
CB_008678	0.002444916	0.0004353	2.097045	down	133874	C5orf58	
CB_028529	0.002443076	0.00043489	2.016615	down	377677	CA13	
CB_017397	0.00243491	0.00043293	2.552977	down	1718	DHCR24	
CB_012252	0.002423859	0.00043019	4.050888	down	7177	TPSAB1	
CB_009608	0.002418271	0.00042867	2.222712	down	1806	DPYD	
CB_020047	0.002418271	0.00042883	7.557438	down	55885	LMO3	
CB_029345	0.002409869	0.00042663	2.156177	down	8418	CMAH	
CB_024750	0.002409869	0.00042669	2.566516	down	140886	PABPC5	
CB_013676	0.00240635	0.0004258	3.176594	down	9365	KL	
CB_024865	0.00237359	0.00041722	2.224724	down	26509	MYOF	
CB_029871	0.002357539	0.00041312	2.016584	down	326342	EMR4P	
CB_023199	0.002327554	0.0004061	2.24345	down	83872	HMCN1	
CB_024140	0.002321314	0.00040474	2.194065	down	153572	IRX2	
CB_025467	0.002318363	0.00040405	2.015358	down	137835	TMEM71	
CB_004695	0.00228501	0.00039625	2.799103	down			
CB_016219	0.002284429	0.00039597	2.577345	down	23576	DDAH1	
CB_000515	0.002282611	0.00039547	2.350749	down	647190	RPS16P5	
CB_011161	0.002279035	0.00039467	3.330236	down	3489	IGFBP6	
CB_025676	0.002269485	0.00039233	2.694388	down	1154	CISH	
CB_025571	0.002245305	0.00038648	2.023187	down	158326	FREM1	
CB_028793	0.002236306	0.00038468	2.224079	down	9414	TJP2	
CB_005360	0.002229111	0.00038294	4.481758	down	2266	FGG	
CB_011909	0.002205471	0.00037693	2.533769	down	6092	ROBO2	
CB_027844	0.002205301	0.00037687	3.072344	down	163782	KANK4	
CB_004955	0.002188499	0.00037316	2.137018	down	2052	EPHX1	
CB_029565	0.002183632	0.00037213	2.005797	down	196968	C15orf51	
CB_009576	0.00216217	0.00036706	3.644653	down	287	ANK2	
CB_006978	0.002148176	0.00036401	2.155485	down	4059	BCAM	
CB_020145	0.002148054	0.00036395	3.716419	down	56138	PCDHA11	
CB_013539	0.00213879	0.0003619	2.932221	down	8825	LIN7A	
CB_010850	0.00213636	0.00036126	3.411998	down	1359	CPA3	
CB_029001	0.002129843	0.00035992	2.239879	down	388325	C17orf87	
CB_013345	0.002118267	0.00035743	2.548627	down	2255	FGF10	
CB_005342	0.002096569	0.00035287	6.092153	down	1080	CFTR	
CB_000693	0.002096497	0.00035272	3.357297	down	401147	FLJ43963	
CB_006164	0.002087388	0.00035043	2.014817	down	388335	TMEM220	
CB_014740	0.00208494	0.00034972	2.784345	down	4093	SMAD9	
CB_023369	0.002079397	0.00034853	3.237214	down	84141	FAM176A	
CB_024815	0.002070054	0.00034647	2.555518	down	90139	TSPAN18	
CB_011990	0.002067057	0.00034584	2.161065	down	6415	SEPW1	
CB_011911	0.002063581	0.00034516	2.867992	down	6098	ROS1	
CB_009558	0.002049319	0.00034163	2.229869	down	199990	C1orf86	
CB_024044	0.002034336	0.00033762	2.437689	down	85409	NKD2	
CB_013000	0.002028809	0.00033638	2.936929	down	2070	EYA4	
CB_022830	0.002022857	0.00033507	2.937898	down	81027	TUBB1	
CB_029281	0.002021084	0.00033468	2.142123	down	4500	MT1L	
CB_012224	0.002019967	0.00033415	2.343979	down	7097	TLR2	
CB_011150	0.002018799	0.00033389	2.013444	down	3398	ID2	
CB_016446	0.002016517	0.0003332	4.575239	down	23569	PADI4	
CB_008172	0.002009432	0.00033151	2.496226	down	368	ABCC6	
CB_002887	0.002007088	0.00033109	3.069322	down			
CB_022824	0.001995445	0.00032876	2.023112	down	80709	AKNA	
CB_029131	0.00197962	0.00032516	2.006721	down	400223	C14orf181	

CB_024702	0.00197776	0.00032472	2.285282	down	5783	PTPN13	
CB_021131	0.001972991	0.00032384	2.346	down	6546	SLC8A1	
CB_010022	0.001971865	0.00032353	2.120383	down	3572	IL6ST	
CB_005058	0.001966511	0.00032244	2.311007	down	3784	KCNQ1	
CB_014469	0.001957949	0.00032075	2.182631	down	5837	PYGM	
CB_017126	0.001947617	0.00031854	2.390333	down	27295	PDLIM3	
CB_029000	0.001928546	0.0003145	2.169195	down	388325	C17orf87	
CB_017183	0.001926944	0.00031412	2.802173	down	11227	GALNT5	
CB_029135	0.001926944	0.00031412	2.963429	down	400451	FAM174B	
CB_007080	0.001926901	0.00031402	2.824521	down	10873	ME3	
CB_023071	0.001917623	0.00031191	2.989891	down	64332	NFKBIZ	
CB_007807	0.00191114	0.00031024	2.322675	down	2852	GPER	
CB_026021	0.00191114	0.00031022	2.588431	down	122481	AK7	
CB_015721	0.001898684	0.00030746	2.008843	down	11035	RIPK3	
CB_018202	0.0018943	0.00030648	2.357385	down	26084	ARHGEF26	
CB_022319	0.001891798	0.00030578	2.062362	down	79789	CLMN	
CB_009945	0.00188437	0.00030425	2.095166	down	25976	TIPARP	
CB_027777	0.001878517	0.00030295	2.049657	down	145447	ABHD12B	
CB_009556	0.00187507	0.00030221	2.053561	down	199990	C1orf86	
CB_012721	0.001869424	0.00030098	2.162045	down	8727	CTNNAL1	
CB_028825	0.001862994	0.00029973	2.909561	down	1191	CLU	
CB_001820	0.001862498	0.0002996	2.04722	down	56255	TMX4	
CB_022363	0.001862443	0.00029953	4.515446	down	79838	TMC5	
CB_005040	0.001852163	0.00029718	2.845138	down	3426	CFI	
CB_020162	0.001831122	0.00029236	2.083409	down	27115	PDE7B	
CB_017580	0.001827362	0.00029157	2.615369	down	22875	ENPP4	
CB_012898	0.00182047	0.00029015	2.074351	down	9034	CCRL2	
CB_029312	0.001819452	0.00028995	6.244924	down	140828	NCRNA00261	
CB_019900	0.00181641	0.00028919	2.041067	down	55790	CSGALNACT1	
CB_030773	0.001810967	0.00028809	2.537884	down	100131755	ARMCX4	
CB_007841	0.001807648	0.00028733	2.354734	down	153218	SPINK13	
CB_027865	0.001800643	0.0002855	2.095817	down	3290	HSD11B1	
CB_007449	0.001800643	0.00028557	2.206912	down	91746	YTHDC1	
CB_021142	0.001794996	0.00028422	3.112324	down	7373	COL14A1	
CB_005725	0.001791901	0.00028324	3.810394	down	4883	NPR3	
CB_011062	0.00179139	0.00028305	3.158404	down	2921	CXCL3	
CB_022196	0.00178958	0.00028265	2.010263	down	79661	NEIL1	
CB_004780	0.00178628	0.00028195	2.016094	down	51312	SLC25A37	
CB_001381	0.001780311	0.00028079	2.063786	down			
CB_009751	0.001769419	0.00027854	3.235517	down	6323	SCN1A	
CB_026340	0.001725535	0.00026908	3.300643	down	202559	KHDRBS2	
CB_008937	0.001725091	0.00026896	2.142638	down	100131827	ZNF717	
CB_021269	0.001720579	0.00026809	2.299096	down	58526	MID1IP1	
CB_027855	0.001719875	0.00026793	2.178256	down	338773	TMEM119	
CB_022318	0.001704891	0.00026484	2.059006	down	79789	CLMN	
CB_012928	0.001702561	0.00026434	3.132085	down	1756	DMD	
CB_030508	0.00169989	0.00026352	2.197034	down	193629	NCRNA00189	
CB_005778	0.001699543	0.00026339	2.794995	down	5740	PTGIS	
CB_026410	0.001690438	0.0002614	2.643621	down	255119	C4orf22	
CB_007239	0.001690378	0.00026135	2.331804	down	340526	RGAG4	
CB_010974	0.001690378	0.00026134	2.501272	down	2247	FGF2	
CB_004901	0.001651802	0.00025303	2.008118	down	717	C2	
CB_009727	0.001649714	0.00025253	3.708845	down	2719	GPC3	
CB_023402	0.0016394	0.00025053	2.252908	down	84187	TMEM164	
CB_023914	0.001639007	0.00025042	3.639547	down	85004	RERG	
CB_002081	0.001638444	0.00025026	2.117446	down			

CB_021437	0.001630681	0.00024858	2.270198	down	3126	HLA-DRB4	
CB_026882	0.001627986	0.00024809	2.181727	down	1438	CSF2RA	
CB_018895	0.001612979	0.00024488	2.027309	down	51761	ATP8A2	
CB_023122	0.001610497	0.00024443	2.633294	down	83716	CRISPLD2	
CB_002415	0.001609754	0.00024422	2.00055	down			
CB_018306	0.001591214	0.00024047	2.343522	down	50486	G0S2	
CB_022149	0.001589373	0.00024017	3.42573	down	79614	C5orf23	
CB_007336	0.001588907	0.00024006	2.206042	down	83478	ARHGAP24	
CB_014381	0.001572366	0.000237	3.335777	down	3158	HMGCS2	
CB_004946	0.001570287	0.00023658	2.123365	down	1806	DPYD	
CB_004649	0.001564916	0.00023528	2.788015	down			
CB_010438	0.001558381	0.00023395	2.37119	down	2014	EMP3	
CB_008932	0.001542355	0.00023063	3.265558	down	7068	THRB	
CB_006787	0.001540219	0.00023006	2.766742	down	345895	RSPH4A	
CB_029568	0.001539058	0.00022969	2.045455	down	196968	C15orf51	
CB_026407	0.001537273	0.00022927	2.239905	down	254778	C8orf46	
CB_001133	0.001534953	0.00022871	2.487605	down	151878	LOC151878	
CB_005774	0.001525119	0.00022673	2.970313	down	5732	PTGER2	
CB_014102	0.001523824	0.00022647	5.287482	down	2066	ERBB4	
CB_018816	0.001515467	0.00022491	2.165366	down	51232	CRIM1	
CB_005619	0.001511674	0.00022414	2.375487	down	2350	FOLR2	
CB_010967	0.001508785	0.00022352	2.032132	down	2204	FCAR	
CB_010283	0.001508288	0.00022339	7.317299	down	1066	CES1	
CB_021937	0.001506592	0.00022305	2.441583	down	1879	EBF1	
CB_001393	0.001502	0.00022218	2.449952	down	6919	TCEA2	
CB_013830	0.001500962	0.00022197	3.073313	down	2115	ETV1	
CB_018105	0.001490189	0.00021984	2.278962	down	25937	WWTR1	
CB_002039	0.001488746	0.0002194	3.382203	down			
CB_026475	0.001478987	0.00021732	4.090697	down	222865	TMEM130	
CB_017678	0.001471325	0.0002157	2.312355	down	23043	TNIK	
CB_013552	0.001471325	0.00021565	3.873436	down	9077	DIRAS3	
CB_009539	0.001469825	0.00021511	3.539931	down	6563	SLC14A1	
CB_026627	0.001468075	0.00021479	2.841764	down	167681	PRSS35	
CB_005592	0.001464362	0.00021415	2.284514	down	1579	CYP4A11	
CB_018352	0.001446211	0.00021023	2.516923	down	51364	ZMYND10	
CB_008272	0.00144222	0.00020945	2.150174	down	94030	LRRC4B	
CB_022638	0.001430804	0.00020713	2.221518	down	80221	ACSF2	
CB_008394	0.00143052	0.00020702	2.12145	down	139716	GAB3	
CB_021168	0.001414535	0.00020334	2.157734	down	6252	RTN1	
CB_000130	0.001412562	0.00020304	2.327585	down			
CB_021524	0.001409898	0.00020258	2.087257	down	63976	PRDM16	
CB_012047	0.001409574	0.00020237	5.264833	down	6590	SLPI	
CB_015842	0.001403499	0.00020101	2.255297	down	11069	RAPGEF4	
CB_011668	0.001400084	0.00020042	4.556183	down	5473	PPBP	
CB_017867	0.001392779	0.00019904	2.282842	down	23261	CAMTA1	
CB_022276	0.001382042	0.00019707	2.338065	down	79742	CXorf36	
CB_020698	0.001379138	0.00019653	2.052604	down	57326	PBXIP1	
CB_014464	0.001375599	0.00019593	2.052527	down	5010	CLDN11	
CB_029676	0.001370973	0.00019508	2.099927	down	387751	GVINP1	
CB_008455	0.001363115	0.00019366	3.341793	down	155006	TMEM213	
CB_029644	0.001355766	0.00019229	2.348235	down	644314	MT1IP	
CB_023793	0.001351122	0.00019124	2.636154	down	84870	RSPO3	
CB_018897	0.001351122	0.00019126	3.223837	down	51761	ATP8A2	
CB_002821	0.001350716	0.00019113	2.087483	down			
CB_018890	0.001345531	0.00019002	2.687913	down	51348	KLRF1	
CB_014405	0.001343337	0.00018949	2.134579	down	3632	INPP5A	

CB_022382	0.001338771	0.00018859	2.031788	down	79856	SNX22	
CB_027326	0.00133269	0.00018735	2.268204	down	3568	IL5RA	
CB_022063	0.001332562	0.00018731	3.030999	down	79170	PRR15L	
CB_014239	0.001319703	0.00018517	2.101032	down	4783	NFIL3	
CB_015763	0.001308428	0.00018306	2.123251	down	6515	SLC2A3	
CB_004993	0.001307585	0.00018291	2.083454	down	2581	GALC	
CB_004627	0.001306526	0.00018266	2.059712	down			
CB_008964	0.001306526	0.00018264	2.17503	down	775	CACNA1C	
CB_008699	0.001300157	0.00018137	3.12902	down	54852	PAQR5	
CB_018492	0.001292215	0.00017996	2.162499	down	51643	TMBIM4	
CB_014187	0.001287802	0.00017904	2.219572	down	3021	H3F3B	
CB_006082	0.001285579	0.00017871	2.097673	down	5136	PDE1A	
CB_007666	0.001284629	0.00017846	2.877247	down	6340	SCNN1G	
CB_010764	0.001283153	0.00017814	2.524181	down	969	CD69	
CB_031285	0.001278941	0.00017728	2.089024	down	100129744	LOC100129744	
CB_025547	0.001278803	0.00017722	2.096357	down	153830	RNF145	
CB_005989	0.001274427	0.00017644	2.926335	down	220965	FAM13C	
CB_001955	0.001268771	0.00017523	2.761338	down			
CB_020678	0.00125565	0.00017282	2.041572	down	57213	C13orf1	
CB_015806	0.001254515	0.00017262	2.122043	down	8510	MMP23B	
CB_021634	0.001254284	0.00017251	2.006862	down	27010	TPK1	
CB_010525	0.001253591	0.00017234	5.45219	down	3036	HAS1	
CB_022302	0.001252801	0.00017217	2.034915	down	79772	MCTP1	
CB_004445	0.001252139	0.000172	2.006041	down			
CB_008059	0.001242528	0.00017046	5.524799	down	6751	SSTR1	
CB_004704	0.001237985	0.00016968	3.940576	down			
CB_028619	0.001234237	0.00016905	4.056676	down	375612	LHFPL3	
CB_022285	0.001233704	0.00016885	2.958319	down	79750	ZNF385D	
CB_016292	0.001233704	0.00016885	4.077371	down	23430	TPSD1	
CB_009209	0.001229212	0.00016805	2.053774	down	9208	LRRFIP1	
CB_025333	0.001227076	0.00016766	2.278726	down	84807	NFKBID	
CB_026986	0.001224659	0.00016708	2.349384	down	143098	MPP7	
CB_011100	0.001221208	0.00016643	2.29861	down	3115	HLA-DPB1	
CB_008293	0.001220006	0.00016618	2.164005	down	389072	PLEKHM3	
CB_014264	0.001217128	0.00016543	19.5877	down	653509	SFTPA1	
CB_022859	0.001212394	0.0001646	2.517035	down	81563	C1orf21	
CB_007983	0.001207696	0.00016371	2.310858	down	913	CD1E	
CB_028125	0.001207168	0.00016355	2.437384	down	56999	ADAMTS9	
CB_009626	0.001204827	0.00016311	3.076824	down	57139	RGL3	
CB_005041	0.001203757	0.00016281	3.059325	down	3426	CFI	
CB_022441	0.001202264	0.00016246	3.346058	down	79919	C2orf54	
CB_021421	0.001185953	0.00015956	2.885555	down	8431	NR0B2	
CB_017235	0.001183878	0.00015911	2.565283	down	6277	S100A6	
CB_016527	0.00117929	0.0001582	2.25383	down	7224	TRPC5	
CB_023810	0.001175525	0.00015749	2.224477	down	83700	JAM3	
CB_003524	0.001171144	0.00015673	6.408389	down			
CB_021370	0.001169435	0.00015644	6.840188	down	2243	FGA	
CB_017080	0.001168499	0.00015625	2.500654	down	27123	DKK2	
CB_024105	0.001166064	0.0001559	2.02664	down	89927	C16orf45	
CB_030031	0.001133856	0.00015031	2.07343	down	64098	PARVG	
CB_031157	0.001126899	0.00014913	2.475904	down	730102	LOC730102	
CB_009204	0.001120222	0.00014789	2.712632	down	100129583	FAM47E	
CB_018575	0.001117793	0.00014741	2.080829	down	10404	PGCP	
CB_015494	0.001115564	0.00014691	2.056362	down	10791	VAMP5	
CB_007875	0.001104896	0.00014505	2.569029	down	27253	PCDH17	
CB_011871	0.001104858	0.00014502	2.330578	down	5957	RCVRN	

CB_020431	0.00110448	0.0001449	4.095452	down	56892	C8orf4	
CB_028108	0.001104227	0.00014484	2.653295	down	9586	CREB5	
CB_011910	0.001103855	0.00014476	4.201265	down	6092	ROBO2	
CB_028569	0.001103572	0.00014469	2.511027	down	375449	MAST4	
CB_009313	0.001103297	0.00014463	5.797789	down	388743	CAPN8	
CB_012212	0.001102095	0.00014443	2.157223	down	7067	THRA	
CB_011857	0.001088656	0.00014217	2.104386	down	5919	RARRES2	
CB_010816	0.001068828	0.00013879	2.302888	down	1215	CMA1	
CB_013923	0.001068665	0.00013872	2.217876	down	5551	PRF1	
CB_012405	0.001057709	0.00013671	2.754176	down	7855	FZD5	
CB_008548	0.001054763	0.00013629	2.355422	down	653567	FAM23A	
CB_010284	0.001050467	0.00013545	6.791874	down	1066	CES1	
CB_002066	0.001045888	0.00013475	2.163631	down			
CB_016388	0.001045888	0.00013473	2.847447	down	22941	SHANK2	
CB_005163	0.001042075	0.00013412	2.235947	down	5627	PROS1	
CB_012756	0.001041418	0.00013401	2.505211	down	8778	SIGLEC5	
CB_021239	0.001031921	0.00013249	2.100329	down	58488	PCTP	
CB_012202	0.001026153	0.00013146	4.649083	down	7044	LEFTY2	
CB_017153	0.001022833	0.00013077	2.707219	down	27345	KCNMB4	
CB_009585	0.001021476	0.00013056	2.056907	down	309	ANXA6	
CB_024526	0.001017614	0.0001298	3.534912	down	117283	IP6K3	
CB_026760	0.001014136	0.00012918	2.083297	down	54414	SIAE	
CB_024529	0.00101076	0.00012847	2.371403	down	117289	TAGAP	
CB_006139	0.001008245	0.00012806	2.016735	down	5768	QSOX1	
CB_018034	0.001003853	0.00012721	3.527697	down	25849	PARM1	
CB_020931	0.00100079	0.00012668	2.846926	down	57619	SHROOM3	
CB_012499	0.000997247	0.00012614	3.896357	down	8395	PIP5K1B	
CB_011245	0.000995433	0.00012574	2.25069	down	3824	KLRD1	
CB_023349	0.000985883	0.00012412	3.045296	down	92126	DSEL	
CB_006707	0.000985752	0.00012405	2.841206	down	400831	C20orf202	
CB_004736	0.000983664	0.00012374	2.29421	down			
CB_013460	0.000983263	0.00012351	2.05156	down	5873	RAB27A	
CB_005109	0.000982752	0.00012343	2.020453	down	653361	NCF1	
CB_012875	0.000981622	0.00012325	2.545177	down	8987	STBD1	
CB_007806	0.000977069	0.00012235	2.814166	down	2852	GPER	
CB_016290	0.000975349	0.00012212	2.122541	down	11043	MID2	
CB_021036	0.00096889	0.00012114	3.598288	down	366	AQP9	
CB_025755	0.000965757	0.00012056	2.028747	down	132671	SPATA18	
CB_022255	0.000964661	0.00012037	2.075559	down	79720	VPS37B	
CB_031467	0.000964661	0.00012036	2.961837	down	256483	LOC256483	
CB_010719	0.000964111	0.00012025	2.533406	down	727	C5	
CB_001641	0.000963838	0.00012007	2.601537	down	730066	VN1R103P	
CB_028872	0.000955533	0.00011879	2.216658	down	81704	DOCK8	
CB_017694	0.000952803	0.00011828	2.054881	down	23062	GGA2	
CB_021373	0.000942772	0.00011675	2.139229	down	558	AXL	
CB_021448	0.000939132	0.00011613	2.763933	down	53826	FXYD6	
CB_018199	0.000937724	0.00011588	2.241775	down	23034	SAMD4A	
CB_014795	0.000934996	0.00011546	2.41444	down	4502	MT2A	
CB_005398	0.000932492	0.00011495	19.5318	down	6439	SFTPB	
CB_026781	0.000925031	0.00011376	2.347152	down	4000	LMNA	
CB_010991	0.000922224	0.00011335	2.081382	down	2321	FLT1	
CB_024183	0.000922222	0.00011333	3.36141	down	89876	C3orf15	
CB_015294	0.000920722	0.00011307	2.352602	down	10577	NPC2	
CB_011282	0.000916393	0.00011238	2.256248	down	3920	LAMP2	
CB_002652	0.000916393	0.00011237	2.567374	down			
CB_025318	0.000914234	0.000112	2.403189	down	168433	RNF133	

CB_012221	0.000913925	0.00011192	2.861948	down	7089	TLE2	
CB_008029	0.000912066	0.00011155	3.480228	down	4582	MUC1	
CB_000213	0.000911505	0.00011144	2.026226	down	441108	C5orf56	
CB_025374	0.000907275	0.00011066	2.823832	down	9590	AKAP12	
CB_012561	0.000904993	0.00011031	2.064548	down	8496	PPFIBP1	
CB_014951	0.000904691	0.00011024	2.676656	down	10418	SPON1	
CB_025375	0.00090275	0.00010992	3.024181	down	9590	AKAP12	
CB_024938	0.000902175	0.00010979	2.036813	down	6525	SMTN	
CB_023064	0.000900349	0.00010948	2.00987	down	23710	GABARAPL1	
CB_025642	0.000898605	0.00010912	2.499752	down	201134	CCDC46	
CB_032005	0.000898605	0.00010911	2.845466	down	100128893	LOC100128893	
CB_020767	0.000898592	0.00010907	2.216933	down	57419	SLC24A3	
CB_024277	0.000897944	0.00010897	2.239242	down	3113	HLA-DPA1	
CB_017513	0.000895565	0.00010862	2.515983	down	9920	KBTBD11	
CB_022228	0.000895417	0.00010858	2.104389	down	79695	GALNT12	
CB_017830	0.000891784	0.00010799	2.038887	down	23220	DTX4	
CB_002476	0.000891784	0.00010798	2.546756	down			
CB_006682	0.000891784	0.00010799	4.011721	down	120114	FAT3	
CB_029270	0.000883699	0.00010661	2.54142	down	727708	SNORD116-19	
CB_020151	0.000881764	0.00010622	2.462389	down	56121	PCDHB15	
CB_013465	0.000879973	0.00010595	2.355048	down	5920	RARRES3	
CB_005250	0.000877542	0.00010559	2.263131	down	1959	EGR2	
CB_032052	0.000875825	0.00010535	2.460902	down	100507073	LOC100507073	
CB_009764	0.000871587	0.00010466	4.5683	down	55607	PPP1R9A	
CB_000110	0.000871261	0.00010458	3.242921	down			
CB_014288	0.000868729	0.00010418	3.742084	down	7851	MALL	
CB_021571	0.000868362	0.000104	2.388774	down	64123	ELTD1	
CB_022963	0.000866528	0.0001037	3.570929	down	81849	ST6GALNAC5	
CB_000931	0.000865241	0.00010348	2.424924	down	100131831	LOC100131831	
CB_023446	0.000864324	0.00010333	2.372112	down	84251	SGIP1	
CB_019899	0.000862126	0.00010299	2.689747	down	55332	DRAM1	
CB_004296	0.000858439	0.00010243	2.064854	down			
CB_004915	0.000856044	0.00010193	2.179225	down	1071	CETP	
CB_010347	0.000851636	0.00010127	2.761377	down	1521	CTSW	
CB_015473	0.000850203	0.00010102	2.522535	down	10753	CAPN9	
CB_030196	0.000848309	0.00010074	2.856687	down	969	CD69	
CB_017367	0.000848161	0.00010069	2.409404	down	9770	RASSF2	
CB_010232	0.000845752	0.00010031	2.047508	down	659	BMPR2	
CB_012317	0.000840681	9.9459E-05	2.108644	down	7408	VASP	
CB_028231	0.000836838	9.8859E-05	2.513059	down	348093	RBPMS2	
CB_027723	0.000835401	9.8663E-05	2.170461	down	152189	CMTM8	
CB_018200	0.000824448	9.6938E-05	2.497639	down	23034	SAMD4A	
CB_016407	0.00082384	9.684E-05	2.60829	down	23764	MAFF	
CB_019785	0.000820942	9.6434E-05	2.293484	down	55244	SLC47A1	
CB_021143	0.000818458	9.6039E-05	3.451605	down	7373	COL14A1	
CB_015345	0.000813159	9.5211E-05	2.161592	down	2124	EVI2B	
CB_008186	0.0008123	9.5059E-05	2.471519	down	114548	NLRP3	
CB_028854	0.00080909	9.4606E-05	2.416632	down	286343	C9orf150	
CB_014126	0.000801295	9.3479E-05	2.018434	down	2669	GEM	
CB_014523	0.000800711	9.3375E-05	2.09411	down	7071	KLF10	
CB_015284	0.000795364	9.256E-05	17.85808	down	10568	SLC34A2	
CB_020805	0.000792101	9.2097E-05	2.083729	down	49854	ZNF295	
CB_006602	0.000786591	9.1277E-05	2.962144	down	278	AMY1C	
CB_007378	0.000786352	9.1237E-05	5.979641	down	200373	PCDP1	
CB_017551	0.000784367	9.0969E-05	2.107876	down	2744	GLS	
CB_003453	0.000783567	9.0845E-05	3.32422	down			

CB_022323	0.000783485	9.0817E-05	2.565449	down	79794	C12orf49	
CB_011098	0.000779287	9.0158E-05	2.388027	down	3111	HLA-DOA	
CB_007474	0.000779074	9.0121E-05	4.591641	down	5648	MASP1	
CB_008831	0.000777477	8.9895E-05	2.127986	down	123	PLIN2	
CB_018113	0.000777477	8.9904E-05	2.309025	down	25945	PVRL3	
CB_008465	0.000771645	0.00008898	2.304901	down	284417	TMEM150B	
CB_010349	0.000770336	8.8792E-05	3.786575	down	1524	CX3CR1	
CB_004905	0.000765305	8.8091E-05	3.164545	down	732	C8B	
CB_031733	0.000765234	8.8071E-05	3.430262	down	645722	LOC645722	
CB_009915	0.000764984	8.7981E-05	8.266024	down	142680	SLC34A3	
CB_026221	0.000762925	8.7588E-05	2.414583	down	153020	RASGEF1B	
CB_005744	0.000758719	8.6936E-05	3.265087	down	5243	ABCB1	
CB_018331	0.000757896	8.6782E-05	2.644321	down	9750	FAM65B	
CB_028459	0.000751048	8.5736E-05	3.717061	down	342527	SMTNL2	
CB_005540	0.000750906	8.5708E-05	2.021283	down	725	C4BPB	
CB_008326	0.000750829	8.5665E-05	2.147566	down	152573	SHISA3	
CB_019015	0.000750829	8.5675E-05	2.635284	down	53358	SHC3	
CB_009494	0.000747073	8.5104E-05	2.604009	down	84525	HOPX	
CB_006636	0.000746196	8.4969E-05	2.102508	down	169200	TMEM64	
CB_002675	0.000742169	8.4322E-05	3.124308	down			
CB_022016	0.000741338	8.4192E-05	2.577369	down	79083	MLPH	
CB_012914	0.000741133	8.4157E-05	2.421793	down	9056	SLC7A7	
CB_015048	0.00073265	8.309E-05	2.905399	down	5168	ENPP2	
CB_010382	0.000730968	8.2818E-05	3.286317	down	1768	DNAH6	
CB_009911	0.000729438	8.254E-05	2.148421	down	397	ARHGDIB	
CB_025646	0.000723857	8.1658E-05	2.187238	down	112464	PRKCDBP	
CB_027255	0.00072154	8.1338E-05	2.859588	down	257019	FRMD3	
CB_021119	0.000720021	8.1087E-05	3.288668	down	6565	SLC15A2	
CB_018697	0.000716313	8.0544E-05	2.009441	down	51347	TAOK3	
CB_012046	0.000714206	8.0235E-05	3.781264	down	6588	SLN	
CB_028595	0.00071171	7.979E-05	4.171546	down	158062	LCN6	
CB_014459	0.000709911	7.9464E-05	2.476269	down	4781	NFIB	
CB_010760	0.000709609	7.9408E-05	2.158747	down	953	ENTPD1	
CB_018972	0.000708102	7.9183E-05	2.933513	down	51316	PLAC8	
CB_024836	0.000707079	7.8923E-05	3.208226	down	83657	DYNLRB2	
CB_017528	0.000706063	7.8787E-05	2.026875	down	10144	FAM13A	
CB_012910	0.000703807	7.8469E-05	7.77248	down	9052	GPRC5A	
CB_026363	0.000700515	7.7979E-05	2.398824	down	220164	DOK6	
CB_021936	0.000700515	7.792E-05	2.510459	down	1879	EBF1	
CB_027254	0.000698472	7.7652E-05	4.6539	down	257019	FRMD3	
CB_025636	0.000698381	7.7631E-05	2.882431	down	221458	KIF6	
CB_020845	0.000696111	7.7291E-05	2.101369	down	57528	KCTD16	
CB_015693	0.000692959	7.6831E-05	3.178389	down	10990	LILRB5	
CB_012434	0.000692412	7.6715E-05	2.33568	down	8291	DYSF	
CB_024434	0.000690568	7.6465E-05	3.454456	down	116511	MAS1L	
CB_026725	0.000687554	7.6046E-05	2.136057	down	79683	ZDHHC14	
CB_030991	0.000687532	7.6033E-05	3.162129	down	401022	LOC401022	
CB_017679	0.000687335	7.5989E-05	2.129386	down	23043	TNIK	
CB_018707	0.000686765	7.5904E-05	2.141761	down	51411	BIN2	
CB_024230	0.000679834	7.488E-05	2.021593	down	89853	FAM125B	
CB_022476	0.000679649	7.4784E-05	2.198512	down	79960	PHF17	
CB_016016	0.000679008	7.467E-05	2.425569	down	11248	NXPH3	
CB_013776	0.000678413	7.4561E-05	2.086197	down	114548	NLRP3	
CB_016044	0.000678413	7.4562E-05	2.451745	down	11309	SLCO2B1	
CB_020080	0.000675388	7.4155E-05	2.039939	down	55901	THSD1	
CB_008190	0.000672985	7.3773E-05	2.302613	down	10149	GPR64	

CB_020571	0.000671384	7.3523E-05	2.248707	down	57186	RALGAPA2	
CB_028848	0.000669525	7.3192E-05	3.802958	down	389434	IYD	
CB_005471	0.000667471	7.2851E-05	2.006513	down	4689	NCF4	
CB_016796	0.000667471	7.2831E-05	2.139822	down	57496	MKL2	
CB_003295	0.00066451	7.2328E-05	2.099112	down			
CB_029344	0.000662465	7.2069E-05	2.347939	down	8418	CMAH	
CB_019059	0.00066064	7.1813E-05	4.578533	down	53905	DUOX1	
CB_017885	0.000657607	7.1347E-05	2.370287	down	23286	WWC1	
CB_016039	0.000655024	7.0953E-05	2.578291	down	11279	KLF8	
CB_024333	0.00065293	7.0654E-05	3.192415	down	92304	SCGB3A1	
CB_020709	0.000652236	7.0537E-05	2.120626	down	57192	MCOLN1	
CB_004896	0.000651897	7.0483E-05	2.173908	down	695	BTK	
CB_021188	0.000651243	7.0399E-05	2.183395	down	28513	CDH19	
CB_010734	0.000651103	7.0373E-05	2.122483	down	824	CAPN2	
CB_007304	0.000645752	0.00006958	6.940836	down	1066	CES1	
CB_025304	0.000644579	6.9402E-05	2.193623	down	130540	ALS2CR12	
CB_010757	0.000641881	6.9057E-05	2.440823	down	951	CD37	
CB_011244	0.000641337	6.8972E-05	2.796519	down	3824	KLRD1	
CB_009013	0.000639527	6.8692E-05	2.229488	down	2982	GUCY1A3	
CB_031164	0.000635093	6.8059E-05	3.085593	down	646324	LOC646324	
CB_024964	0.000631065	6.7507E-05	2.413799	down	154661	RUNDC3B	
CB_015717	0.000630097	6.7364E-05	2.321778	down	11027	LILRA2	
CB_010634	0.000629091	6.7216E-05	19.08529	down	361	AQP4	
CB_029593	0.000627692	6.7017E-05	2.179485	down	653390	RRN3P2	
CB_025014	0.000627315	6.6957E-05	2.66041	down	91461	PKDCC	
CB_021172	0.000626604	6.6831E-05	3.421809	down	7363	UGT2B4	
CB_010402	0.000626593	6.682E-05	2.361601	down	1842	ECM2	
CB_019687	0.000625538	6.6648E-05	2.018578	down	55177	FAM82A2	
CB_018176	0.000624723	6.6532E-05	2.063817	down	26040	SETBP1	
CB_011517	0.000624212	6.645E-05	3.484794	down	4950	OCLN	
CB_005575	0.000622937	6.6272E-05	7.861612	down	1440	CSF3	
CB_017845	0.000620218	6.592E-05	4.756366	down	23237	ARC	
CB_012990	0.000616205	6.5332E-05	2.043477	down	1845	DUSP3	
CB_014255	0.000616205	6.5327E-05	2.906896	down	5581	PRKCE	
CB_026932	0.000615098	6.5156E-05	5.452389	down	8013	NR4A3	
CB_020825	0.000614975	6.5118E-05	2.552683	down	57507	ZNF608	
CB_007398	0.000614066	6.4988E-05	2.180378	down	389432	SAMD5	
CB_027427	0.00061334	6.4882E-05	4.583347	down	4499	MT1M	
CB_014781	0.000611742	6.4616E-05	2.052503	down	4303	FOXO4	
CB_010785	0.000607738	6.4106E-05	3.472179	down	1043	CD52	
CB_021689	0.000599807	6.2975E-05	2.089199	down	3125	HLA-DRB3	
CB_024433	0.000597426	6.2592E-05	2.151583	down	116496	FAM129A	
CB_016859	0.000595876	6.2402E-05	4.282075	down	29091	STXBP6	
CB_011518	0.000592511	6.1927E-05	3.391584	down	4950	OCLN	
CB_028562	0.000590291	6.1596E-05	2.037728	down	961	CD47	
CB_006101	0.000590291	6.162E-05	2.910301	down	445347	TARP	
CB_017363	0.000589983	6.1551E-05	2.48865	down	9764	KIAA0513	
CB_010964	0.000589823	6.1525E-05	2.243893	down	2192	FBLN1	
CB_012531	0.000588698	6.1389E-05	2.202423	down	8452	CUL3	
CB_030702	0.000585747	6.0989E-05	3.234896	down	388815	C21orf34	
CB_023437	0.000581548	6.0394E-05	8.202106	down	84239	ATP13A4	
CB_006069	0.000580279	6.0189E-05	2.743193	down	3983	ABLIM1	
CB_024566	0.000579564	6.0087E-05	2.625227	down	59271	C21orf63	
CB_023097	0.000579	5.9992E-05	2.216493	down	83641	FAM107B	
CB_001838	0.000575147	5.9511E-05	2.818178	down	9340	GLP2R	
CB_021469	0.000570964	5.8897E-05	3.027262	down	58191	CXCL16	

CB_018071	0.000568223	5.8506E-05	2.03825	down	25898	RCHY1	
CB_001358	0.000567651	5.8429E-05	2.634557	down	23034	SAMD4A	
CB_005189	0.000565902	5.815E-05	2.097199	down	6444	SGCD	
CB_027124	0.000565304	5.8074E-05	3.56132	down	285755	PPIL6	
CB_004076	0.000564396	5.7913E-05	2.409762	down			
CB_013188	0.000557722	5.6988E-05	2.230834	down	323	APBB2	
CB_006071	0.000557722	5.7006E-05	2.61011	down	3983	ABLIM1	
CB_025168	0.000557443	5.6939E-05	3.043833	down	4481	MSR1	
CB_003808	0.000552192	5.6111E-05	2.498173	down			
CB_020673	0.000551456	5.6028E-05	2.051786	down	57185	NIPAL3	
CB_019951	0.000551353	5.6008E-05	2.013528	down	55356	SLC22A15	
CB_023635	0.000550455	5.59E-05	2.614791	down	84612	PARD6B	
CB_019688	0.000548355	5.5634E-05	2.075288	down	55177	FAM82A2	
CB_025662	0.000545911	5.5335E-05	2.859417	down	143630	UBQLNL	
CB_004871	0.000545798	5.5314E-05	3.661668	down	350	APOH	
CB_007947	0.000545507	5.5276E-05	2.181589	down	831	CAST	
CB_026589	0.000545036	5.5203E-05	2.035777	down	257364	SNX33	
CB_007133	0.00054388	5.5008E-05	2.126497	down	114769	CARD16	
CB_025344	0.000542161	5.4797E-05	3.864485	down	200879	LIPH	
CB_005107	0.000540965	5.46E-05	2.370888	down	653361	NCF1	
CB_007565	0.000540242	5.4494E-05	2.570388	down	30820	KCNIP1	
CB_008473	0.000537351	5.4152E-05	2.463435	down	32	ACACB	
CB_027311	0.000536112	5.3982E-05	2.855862	down	4493	MT1E	
CB_005598	0.00053559	5.3889E-05	4.224595	down	1593	CYP27A1	
CB_029334	0.000531331	5.3309E-05	2.031004	down	401428	OR2A20P	
CB_011165	0.000527085	5.2791E-05	2.139854	down	3563	IL3RA	
CB_007547	0.000525512	5.2559E-05	3.347707	down	619279	ZNF704	
CB_007302	0.000520968	5.2005E-05	2.280404	down	972	CD74	
CB_020099	0.000515898	5.1376E-05	2.667257	down	11107	PRDM5	
CB_025557	0.000514969	5.1243E-05	5.066392	down	8796	SCEL	
CB_012746	0.000514667	5.1205E-05	2.024075	down	8764	TNFRSF14	
CB_014661	0.000511399	5.0736E-05	6.089485	down	10216	PRG4	
CB_023136	0.00050914	5.0413E-05	2.254991	down	56145	PCDHA3	
CB_011213	0.000507789	5.0205E-05	3.133549	down	3738	KCNA3	
CB_026065	0.000503757	4.9634E-05	4.398772	down	127733	UBXN10	
CB_018432	0.00049745	4.8861E-05	2.477516	down	712	C1QA	
CB_015548	0.000496095	4.8652E-05	2.918915	down	10893	MMP24	
CB_009590	0.000495318	4.8542E-05	2.572185	down	11279	KLF8	
CB_025013	0.000491597	4.8102E-05	2.265071	down	91461	PKDCC	
CB_009805	0.000491312	4.804E-05	2.13329	down	93664	CADPS2	
CB_026635	0.000491065	4.7992E-05	5.2469	down	221476	PI16	
CB_016168	0.000490527	4.7893E-05	2.024098	down	23414	ZFPM2	
CB_030225	0.000487902	4.7498E-05	2.123758	down	391037	SKINTL	
CB_014833	0.000487165	4.7403E-05	3.009381	down	6909	TBX2	
CB_027382	0.000485405	4.7178E-05	2.398359	down	283897	C16orf54	
CB_014438	0.000485363	4.7158E-05	2.438157	down	4005	LMO2	
CB_007515	0.000483158	4.689E-05	3.025525	down	255743	NPNT	
CB_005280	0.000483112	4.6878E-05	2.457511	down	3908	LAMA2	
CB_020049	0.000482839	4.6844E-05	6.675845	down	54210	TREM1	
CB_029427	0.00048193	4.6733E-05	2.284647	down	119369	NUDT9P1	
CB_015358	0.000479957	4.6466E-05	2.101553	down	5817	PVR	
CB_024438	0.000477558	4.618E-05	2.138755	down	116842	LEAP2	
CB_018779	0.000469911	4.5232E-05	2.877948	down	9465	AKAP7	
CB_014457	0.000468329	4.5035E-05	2.632839	down	4781	NFIB	
CB_018939	0.000467528	4.4936E-05	2.87287	down	51296	SLC15A3	
CB_021442	0.000464094	4.4465E-05	2.017266	down	26301	GBGT1	

CB_017061	0.000461691	4.4163E-05	5.35787	down	27074	LAMP3	
CB_030123	0.000460052	4.3933E-05	2.145796	down	554203	NCRNA00183	
CB_018096	0.000459203	4.3816E-05	2.443075	down	25925	ZNF521	
CB_006637	0.000459203	4.3806E-05	2.453751	down	169200	TMEM64	
CB_014732	0.000457647	4.3624E-05	6.07865	down	922	CD5L	
CB_002003	0.000457339	4.358E-05	2.221552	down			
CB_018163	0.000453675	4.3137E-05	2.231065	down	667	DST	
CB_020878	0.000453263	4.3084E-05	3.431562	down	57562	KIAA1377	
CB_011407	0.000452694	4.3015E-05	2.915844	down	4332	MNDA	
CB_022060	0.000451534	4.2884E-05	2.809967	down	79168	LILRA6	
CB_012841	0.000451377	4.2862E-05	2.120685	down	8905	AP1S2	
CB_016167	0.000451192	4.2837E-05	2.514937	down	22936	ELL2	
CB_020833	0.000450785	4.277E-05	2.567975	down	57513	CASKIN2	
CB_014011	0.000450785	4.2763E-05	5.887806	down	306	ANXA3	
CB_009540	0.000450132	4.2655E-05	3.861933	down	6563	SLC14A1	
CB_021272	0.000447435	4.2296E-05	4.433033	down	58529	MYOZ1	
CB_014996	0.000447415	4.2287E-05	3.671006	down	4778	NFE2	
CB_008477	0.000442618	4.1686E-05	2.473043	down	79839	CCDC102B	
CB_023403	0.000441875	4.1595E-05	2.33242	down	84187	TMEM164	
CB_022981	0.000441777	4.1576E-05	2.13618	down	6916	TBXAS1	
CB_026550	0.00043887	4.1229E-05	2.869762	down	157869	C8orf84	
CB_028381	0.000437505	4.1038E-05	2.849929	down	4013	VWA5A	
CB_029445	0.000437325	4.1007E-05	2.834638	down	284367	SIGLECP3	
CB_017217	0.000433678	4.059E-05	2.020154	down	8916	HERC3	
CB_014671	0.000431573	4.0345E-05	13.9752	down	10232	MSLN	
CB_025267	0.000430655	4.0245E-05	2.076781	down	146722	CD300LF	
CB_024402	0.000430655	4.0245E-05	3.087214	down	115019	SLC26A9	
CB_011097	0.00042944	4.0084E-05	2.214499	down	3111	HLA-DOA	
CB_005103	0.000429258	4.0048E-05	4.038725	down	4653	MYOC	
CB_025891	0.000426944	3.975E-05	2.193208	down	259230	SGMS1	
CB_020848	0.00042549	3.9544E-05	3.754093	down	57530	CGN	
CB_006138	0.000425361	3.9515E-05	2.063443	down	5768	QSOX1	
CB_009229	0.000422892	3.9231E-05	2.564853	down	247	ALOX15B	
CB_005108	0.000422024	3.9118E-05	2.577965	down	653361	NCF1	
CB_016689	0.000421499	3.9056E-05	3.822804	down	29953	TRHDE	
CB_024650	0.000420523	3.8925E-05	2.09268	down	139716	GAB3	
CB_008511	0.000420017	3.8865E-05	3.991261	down	23705	CADM1	
CB_012658	0.000419486	3.8809E-05	2.192851	down	8633	UNC5C	
CB_013810	0.00041819	3.8676E-05	2.989936	down	1004	CDH6	
CB_010227	0.000416504	3.8487E-05	8.604135	down	651	BMP3	
CB_012760	0.000413948	3.8192E-05	2.141926	down	8787	RGS9	
CB_010720	0.000413449	3.8106E-05	3.203472	down	728	C5AR1	
CB_029461	0.000412817	3.8009E-05	2.228166	down	389538	MGC72080	
CB_003436	0.000412731	3.7991E-05	2.636563	down			
CB_012645	0.000412484	3.7952E-05	2.15253	down	8609	KLF7	
CB_025167	0.00040854	3.7499E-05	4.124187	down	4481	MSR1	
CB_000133	0.000408389	3.7459E-05	2.026609	down	2869	GRK5	
CB_030032	0.000408186	3.7434E-05	2.229464	down	399959	LOC399959	
CB_025082	0.000406562	3.7214E-05	2.462257	down	115330	GPR146	
CB_010813	0.00040655	3.7206E-05	2.873783	down	1183	CLCN4	
CB_025475	0.000405724	3.7086E-05	2.228808	down	139818	DOCK11	
CB_009746	0.000405688	3.707E-05	2.673681	down	5144	PDE4D	
CB_015351	0.000404731	3.6963E-05	2.308927	down	4162	MCAM	
CB_016030	0.000402426	3.6663E-05	2.510481	down	11264	PXMP4	
CB_001044	0.00040221	3.6624E-05	5.19651	down	285025	CCDC141	
CB_024553	0.00040037	3.6406E-05	2.048697	down	118429	ANTXR2	

CB_017301	0.000399325	3.6241E-05	2.592114	down	9706	ULK2	
CB_027125	0.000398471	3.6145E-05	3.574724	down	285755	PPIL6	
CB_021072	0.000396861	3.593E-05	2.643513	down	6641	SNTB1	
CB_010163	0.000395563	3.5743E-05	2.338227	down	3120	HLA-DQB2	
CB_014960	0.000395009	3.5643E-05	2.228267	down	3108	HLA-DMA	
CB_017704	0.000392777	3.5392E-05	2.150874	down	23071	ERP44	
CB_025667	0.000392231	3.5311E-05	2.15274	down	196383	RILPL2	
CB_025117	0.000391896	3.5275E-05	2.620384	down	7099	TLR4	
CB_021338	0.000390717	3.5138E-05	5.706653	down	3170	FOXA2	
CB_014557	0.000390201	3.5061E-05	2.816152	down	10060	ABCC9	
CB_009919	0.000389628	3.4978E-05	2.103589	down	3958	LGALS3	
CB_030717	0.00038936	3.4923E-05	2.124518	down	284232	LOC284232	
CB_013822	0.000387441	3.469E-05	2.907254	down	1794	DOCK2	
CB_026016	0.000387437	3.4683E-05	3.182787	down	121506	ERP27	
CB_024783	0.000387437	3.4683E-05	4.518931	down	81035	COLEC12	
CB_010814	0.000384991	3.4348E-05	2.418104	down	1183	CLCN4	
CB_000440	0.00038496	3.434E-05	2.023628	down	158402	LOC158402	
CB_015818	0.000384757	3.431E-05	3.231023	down	10560	SLC19A2	
CB_026433	0.000384709	3.428E-05	2.679785	down	222166	C7orf41	
CB_020167	0.000384331	3.4241E-05	2.572897	down	54206	ERRFI1	
CB_020932	0.000379426	3.3696E-05	3.8859	down	57619	SHROOM3	
CB_015115	0.000376961	3.3414E-05	5.931463	down	6101	RP1	
CB_011298	0.00037608	3.3309E-05	2.266253	down	3958	LGALS3	
CB_031324	0.000375924	3.3259E-05	2.379922	down			
CB_019626	0.000375924	3.3259E-05	3.638586	down	55137	FIGN	
CB_023436	0.000375924	3.3255E-05	8.145519	down	84239	ATP13A4	
CB_014651	0.000375539	3.3207E-05	3.581699	down	10203	CALCRL	
CB_023554	0.000375494	3.3197E-05	12.36495	down	84417	C2orf40	
CB_009182	0.000374874	3.3125E-05	3.673436	down	84879	MFSD2A	
CB_016683	0.000374846	3.3099E-05	3.377237	down	29950	SERTAD1	
CB_006972	0.000374178	3.301E-05	2.299585	down	401548	SNX30	
CB_020007	0.000372861	3.2852E-05	2.077915	down	55862	ECHDC1	
CB_031538	0.000372488	3.279E-05	4.162681	down	100505695	LOC100505695	
CB_013697	0.000371216	3.2629E-05	7.720913	down	9413	FAM189A2	
CB_018021	0.000370459	3.2547E-05	2.196123	down	55187	VPS13D	
CB_014214	0.000370104	3.2493E-05	2.24628	down	3727	JUND	
CB_004647	0.000368623	3.2345E-05	2.179166	down			
CB_010660	0.000368153	3.2293E-05	2.918217	down	467	ATF3	
CB_026301	0.000366067	3.2068E-05	2.355863	down	196074	METT5D1	
CB_006428	0.000363645	3.1804E-05	2.770407	down	23114	NFASC	
CB_005474	0.000363274	3.1766E-05	3.965363	down	3577	CXCR1	
CB_017254	0.000362922	3.1724E-05	2.088581	down	9663	LPIN2	
CB_014762	0.000361986	3.1625E-05	4.50437	down	4223	MEOX2	
CB_015715	0.00036188	3.1598E-05	2.502412	down	11025	LILRB3	
CB_012286	0.000360312	3.1415E-05	2.364017	down	7305	TYROBP	
CB_010371	0.000359234	3.127E-05	2.302793	down	1735	DIO3	
CB_029675	0.000359102	3.1245E-05	3.071285	down	387751	GVINP1	
CB_011378	0.000359008	3.1222E-05	2.042907	down	4242	MFNG	
CB_010759	0.000357413	3.1044E-05	2.12611	down	953	ENTPD1	
CB_007194	0.00035608	3.0917E-05	2.006915	down	2908	NR3C1	
CB_007898	0.000352832	3.0556E-05	4.492465	down	467	ATF3	
CB_004468	0.000351966	3.0453E-05	2.143084	down			
CB_020016	0.000351966	3.0427E-05	5.842787	down	9496	TBX4	
CB_019014	0.000351249	3.0345E-05	5.63844	down	53358	SHC3	
CB_004935	0.000351184	3.0325E-05	2.216858	down	1471	CST3	
CB_027518	0.000351119	3.0313E-05	3.331355	down	91404	SESTD1	

CB_012899	0.000349552	3.0145E-05	3.100021	down	9037	SEMA5A	
CB_017310	0.000349515	3.0128E-05	2.060134	down	9719	ADAMTSL2	
CB_012234	0.000347987	2.9938E-05	2.488294	down	7111	TMOD1	
CB_023575	0.00034666	2.9791E-05	3.153428	down	84457	PHYHIPL	
CB_023594	0.000344111	2.9512E-05	2.003377	down	7462	LAT2	
CB_026489	0.000343742	2.9461E-05	3.437797	down	256435	ST6GALNAC3	
CB_024182	0.000343719	2.9451E-05	3.64214	down	89876	C3orf15	
CB_006971	0.000342576	2.9299E-05	7.130062	down	401546	C9orf152	
CB_008974	0.000342517	2.9277E-05	2.893375	down	729085	FAM198A	
CB_024507	0.000341509	2.9164E-05	8.666584	down	117156	SCGB3A2	
CB_029867	0.000340756	2.9051E-05	2.953913	down	29931	LOH3CR2A	
CB_004186	0.000338432	2.8778E-05	2.405843	down			
CB_009009	0.000337503	2.8678E-05	2.187948	down	55790	CSGALNACT1	
CB_012398	0.000335895	2.8451E-05	2.12835	down	7802	DNALI1	
CB_011641	0.000334317	2.828E-05	4.214149	down	5350	PLN	
CB_013547	0.000330386	2.7874E-05	2.191413	down	9064	MAP3K6	
CB_012304	0.000329213	2.7733E-05	9.057343	down	7356	SCGB1A1	
CB_027519	0.000327858	2.758E-05	2.505387	down	91404	SESTD1	
CB_015471	0.000327051	2.7476E-05	2.118813	down	10750	GRAP	
CB_023680	0.00032545	2.7298E-05	3.410275	down	84658	EMR3	
CB_018203	0.000325433	2.7286E-05	3.468919	down	26084	ARHGEF26	
CB_013153	0.000323199	2.7047E-05	4.001488	down	9472	AKAP6	
CB_011707	0.000322435	2.6958E-05	2.021455	down	5587	PRKD1	
CB_005269	0.000320946	2.678E-05	2.163885	down	3459	IFNGR1	
CB_014954	0.000320292	2.6713E-05	2.103252	down	10449	ACAA2	
CB_015482	0.000319024	2.6588E-05	2.527154	down	10769	PLK2	
CB_006036	0.00031692	2.6341E-05	3.142389	down	80223	RAB11FIP1	
CB_023445	0.00031636	2.6274E-05	2.563359	down	84251	SGIP1	
CB_010353	0.000316238	2.6249E-05	2.440629	down	1601	DAB2	
CB_024567	0.000314639	2.6086E-05	2.230784	down	59271	C21orf63	
CB_000767	0.000310507	2.5625E-05	2.877287	down	100131657	LOC100131657	
CB_005188	0.000310507	2.5628E-05	2.910291	down	6444	SGCD	
CB_007594	0.000310507	2.5631E-05	4.366012	down	192668	CYS1	
CB_013870	0.000309396	2.5495E-05	2.377988	down	2122	MECOM	
CB_004287	0.000309152	2.5465E-05	2.369238	down			
CB_010327	0.000308715	2.5414E-05	2.824067	down	1397	CRIP2	
CB_008509	0.000306899	2.5204E-05	2.143248	down	116535	MRGPRF	
CB_014372	0.000304618	2.4947E-05	2.444406	down	2615	LRRC32	
CB_011382	0.000304129	2.4873E-05	4.341232	down	4248	MGAT3	
CB_014489	0.000303218	2.4781E-05	2.020598	down	6386	SDCBP	
CB_004539	0.000301958	2.4652E-05	2.374068	down			
CB_021935	0.00030073	2.4528E-05	2.884481	down	1879	EBF1	
CB_009462	0.000300305	2.4474E-05	3.42669	down	80726	KIAA1683	
CB_016421	0.000297978	2.4233E-05	3.762451	down	23554	TSPAN12	
CB_012500	0.000296661	2.4069E-05	4.188244	down	8395	PIP5K1B	
CB_006427	0.000293956	2.3798E-05	3.189143	down	23114	NFASC	
CB_007695	0.000293112	2.3721E-05	2.540304	down	55064	C9orf68	
CB_019861	0.000289739	2.3384E-05	4.377812	down	55304	SPTLC3	
CB_029153	0.000288603	2.3269E-05	2.058843	down	9619	ABCG1	
CB_014941	0.000287867	2.3182E-05	4.107905	down	10396	ATP8A1	
CB_016781	0.000284668	2.2839E-05	3.162948	down	25840	METTL7A	
CB_023107	0.0002837	2.2739E-05	2.044804	down	89822	KCNK17	
CB_029739	0.000283175	2.268E-05	3.707027	down	285205	LOC285205	
CB_015065	0.000282448	2.2612E-05	3.433791	down	5334	PLCL1	
CB_025470	0.000280776	2.2456E-05	2.745697	down	137872	ADHFE1	
CB_003688	0.000280769	2.245E-05	2.235136	down			

CB_014450	0.000280272	2.2389E-05	2.122665	down	4329	ALDH6A1	
CB_002071	0.000280215	2.238E-05	3.772716	down			
CB_025805	0.000280002	2.2354E-05	2.163804	down	80833	APOL3	
CB_008292	0.000279996	2.234E-05	2.065158	down	389072	PLEKHM3	
CB_005775	0.000275197	2.1855E-05	2.574951	down	5734	PTGER4	
CB_012886	0.000271989	2.1485E-05	3.756025	down	9021	SOCS3	
CB_025575	0.000269642	2.1227E-05	4.72905	down	158866	ZDHHC15	
CB_004842	0.000269095	2.115E-05	2.075324	down	38	ACAT1	
CB_015475	0.000268753	2.1113E-05	2.188067	down	10763	NES	
CB_012272	0.000267621	2.0992E-05	18.10842	down	7080	NKX2-1	
CB_009961	0.000266083	2.0842E-05	3.64232	down	57669	EPB41L5	
CB_013494	0.000265217	2.0728E-05	4.196791	down	7102	TSPAN7	
CB_008174	0.000264476	2.0666E-05	2.472454	down	368	ABCC6	
CB_013568	0.000263594	2.0579E-05	2.804071	down	9121	SLC16A5	
CB_000235	0.000263001	2.0517E-05	4.808321	down	150378	FLJ30901	
CB_017200	0.000261057	2.0313E-05	2.595851	down	30061	SLC40A1	
CB_029075	0.000261057	2.0319E-05	3.319777	down	343990	C2orf55	
CB_007897	0.000261057	2.032E-05	4.704104	down	467	ATF3	
CB_013832	0.000260828	2.0269E-05	2.304632	down	2115	ETV1	
CB_004964	0.000259138	2.0101E-05	5.685593	down	2160	F11	
CB_005743	0.000258381	2.0021E-05	4.127358	down	5243	ABCB1	
CB_000324	0.00025782	1.9971E-05	2.808102	down	440479	FLJ34223	
CB_023106	0.000255578	1.9719E-05	3.613199	down	89822	KCNK17	
CB_016416	0.000254214	1.9586E-05	2.364609	down	4482	MSRA	
CB_015994	0.0002534	1.9511E-05	2.564098	down	11213	IRAK3	
CB_018094	0.00025335	1.9503E-05	4.661831	down	25924	MYRIP	
CB_025752	0.00025335	1.9499E-05	2.440571	down	130497	OSR1	
CB_008700	0.000253245	1.9475E-05	2.681615	down	91607	SLFN11	
CB_012225	0.000251286	1.9284E-05	2.899183	down	7098	TLR3	
CB_030655	0.000247363	1.8882E-05	2.832679	down	440925	LOC440925	
CB_010962	0.000246377	1.879E-05	2.789115	down	2180	ACSL1	
CB_002536	0.000245865	1.8732E-05	3.092708	down			
CB_026862	0.000245854	1.8723E-05	2.093828	down	3603	IL16	
CB_005162	0.000243846	1.8543E-05	2.832385	down	5627	PROS1	
CB_006014	0.000243231	1.8484E-05	4.18016	down	5265	SERPINA1	
CB_023292	0.000242447	1.8399E-05	2.745993	down	56100	PCDHGB6	
CB_002598	0.000242219	1.8377E-05	2.285766	down			
CB_030259	0.000240876	1.8245E-05	2.113733	down	79686	C14orf139	
CB_005147	0.000240708	1.8225E-05	4.745345	down	5340	PLG	
CB_007769	0.000239808	1.8145E-05	3.468837	down	642987	TMEM232	
CB_022167	0.000239493	1.8114E-05	3.169836	down	79632	FAM184A	
CB_009728	0.000239167	1.8055E-05	5.434846	down	2719	GPC3	
CB_013453	0.000238838	1.8007E-05	2.209366	down	5330	PLCB2	
CB_022444	0.000238481	1.7969E-05	2.467135	down	79923	NANOG	
CB_027335	0.000236901	1.7798E-05	3.981829	down	152831	KLB	
CB_016858	0.000233958	1.7505E-05	3.862049	down	29091	STXBP6	
CB_019194	0.000233357	1.745E-05	2.18928	down	55603	FAM46A	
CB_000824	0.000233107	1.7426E-05	6.113417	down	120892	LRRK2	
CB_008926	0.000232593	1.7359E-05	2.652	down	6935	ZEB1	
CB_014455	0.00023156	1.7268E-05	2.871058	down	4774	NFIA	
CB_026377	0.000230969	1.7213E-05	2.226815	down	114781	BTBD9	
CB_011521	0.000230068	1.7134E-05	5.481533	down	4973	OLR1	
CB_019503	0.000229443	1.7055E-05	2.297663	down	55031	USP47	
CB_021942	0.000229232	1.7036E-05	2.335417	down	3203	HOXA6	
CB_010219	0.000229028	1.7017E-05	2.066064	down	4853	NOTCH2	
CB_008709	0.000227132	1.6826E-05	2.311268	down	3910	LAMA4	

CB_011620	0.000227132	1.6819E-05	20.80368	down	5284	PIGR	
CB_005427	0.000226928	1.6789E-05	4.414617	down	6556	SLC11A1	
CB_014062	0.000226915	1.6785E-05	2.475075	down	1051	CEBPB	
CB_021552	0.000225951	1.6692E-05	2.733319	down	64097	EPB41L4A	
CB_010165	0.000225951	1.6689E-05	3.992108	down	23026	MYO16	
CB_002552	0.000225533	1.6636E-05	2.084422	down			
CB_027173	0.000224263	1.6514E-05	2.212298	down	285521	COX18	
CB_002403	0.000220602	1.6185E-05	2.263112	down			
CB_023342	0.000220245	1.6151E-05	3.410897	down	84101	USP44	
CB_013493	0.00021997	1.6121E-05	2.087429	down	7084	TK2	
CB_006791	0.00021997	1.612E-05	2.321541	down	387923	SERP2	
CB_016972	0.00021997	1.6112E-05	2.547963	down	23533	PIK3R5	
CB_029756	0.000219804	1.6088E-05	3.412063	down	254312	LOC254312	
CB_021608	0.000218795	1.599E-05	2.149881	down	9659	PDE4DIP	
CB_029890	0.000218422	1.5945E-05	2.269173	down	84852	ATP1A1OS	
CB_002791	0.00021652	1.5755E-05	2.025787	down			
CB_009177	0.000216067	1.5718E-05	2.781629	down	255809	C19orf38	
CB_020268	0.000215913	1.5704E-05	2.42823	down	54532	USP53	
CB_012216	0.000215277	1.5651E-05	2.129275	down	7077	TIMP2	
CB_005470	0.000213534	1.5488E-05	2.049253	down	3454	IFNAR1	
CB_002633	0.000212371	1.5386E-05	2.351496	down			
CB_015222	0.000211478	1.5304E-05	2.206241	down	10486	CAP2	
CB_018141	0.000209818	1.5164E-05	2.52139	down	9223	MAGI1	
CB_022303	0.000209536	1.514E-05	2.11759	down	79772	MCTP1	
CB_010911	0.000208661	1.5057E-05	3.983847	down	1839	HBEGF	
CB_022699	0.000208083	1.5003E-05	3.497279	down	80332	ADAM33	
CB_005187	0.000203232	1.455E-05	3.421918	down	6338	SCNN1B	
CB_002916	0.000202654	1.4482E-05	3.177459	down			
CB_021480	0.000201047	1.4332E-05	2.425462	down	63895	FAM38B	
CB_002194	0.000199804	1.421E-05	2.916681	down			
CB_029140	0.000199804	1.4214E-05	6.353263	down	344148	NCKAP5	
CB_026066	0.000199773	1.4204E-05	2.588361	down	127795	C1orf87	
CB_015049	0.000198087	1.4071E-05	2.383635	down	5178	PEG3	
CB_020889	0.000198057	1.4063E-05	3.266324	down	57573	ZNF471	
CB_020238	0.000197037	1.394E-05	2.885331	down	54491	FAM105A	
CB_001336	0.0001969	1.3921E-05	2.109793	down			
CB_006827	0.00019625	1.386E-05	3.060278	down	400746	C1orf130	
CB_026218	0.000196226	1.3853E-05	2.063896	down	152926	PPM1K	
CB_015840	0.000196226	1.3854E-05	4.152515	down	11067	C10orf10	
CB_016934	0.000195305	1.3775E-05	2.37793	down	10982	MAPRE2	
CB_007694	0.000195181	1.3762E-05	2.46941	down	220979	C10orf25	
CB_020100	0.000195181	1.3755E-05	2.773583	down	11107	PRDM5	
CB_026670	0.000195071	1.3723E-05	2.208905	down	153222	C5orf41	
CB_012768	0.000195071	1.3717E-05	4.619525	down	8794	TNFRSF10C	
CB_030688	0.000194497	1.3662E-05	2.074988	down	54508	FLJ11235	
CB_024736	0.000194225	1.3634E-05	2.721204	down	1305	COL13A1	
CB_013755	0.000192271	1.3472E-05	3.047842	down	9452	ITM2A	
CB_005288	0.000191942	1.3446E-05	3.193554	down	4688	NCF2	
CB_015508	0.000191942	1.344E-05	3.758268	down	10810	WASF3	
CB_026390	0.000191862	1.3422E-05	2.966039	down	222223	KIAA1324L	
CB_009654	0.000191819	1.3413E-05	2.276674	down	644538	LOC644538	
CB_012038	0.000191647	1.339E-05	3.058294	down	6571	SLC18A2	
CB_014907	0.000191647	1.3392E-05	3.167716	down	10319	LAMC3	
CB_019638	0.000191	1.3335E-05	2.887915	down	55711	FAR2	
CB_013531	0.000190912	1.3326E-05	2.34809	down	8313	AXIN2	
CB_009170	0.000190184	1.3254E-05	2.01193	down	93166	PRDM6	

CB_002469	0.000188915	1.3153E-05	3.158724	down			
CB_016886	0.000187356	1.3015E-05	2.123352	down	3705	ITPK1	
CB_022369	0.000186462	1.2936E-05	2.592668	down	79843	FAM124B	
CB_014585	0.000186462	1.2938E-05	3.26291	down	10100	TSPAN2	
CB_004397	0.000186416	1.292E-05	4.473073	down			
CB_003457	0.000185466	1.2842E-05	3.242331	down			
CB_023962	0.000185123	1.2813E-05	11.6112	down	4969	OGN	
CB_012906	0.000183919	1.2706E-05	2.602648	down	9046	DOK2	
CB_001146	0.000183469	1.2669E-05	2.14031	down	731424	LOC731424	
CB_004798	0.000183053	1.2638E-05	2.238757	down			
CB_013445	0.000182694	1.2604E-05	2.204944	down	5209	PFKFB3	
CB_017684	0.000182634	1.2597E-05	2.062777	down	23051	ZHX3	
CB_012006	0.000182122	1.255E-05	11.88666	down	6441	SFTPD	
CB_018433	0.000180338	1.2407E-05	5.544837	down	51090	PLLP	
CB_012755	0.000179586	1.2337E-05	2.172774	down	8777	MPDZ	
CB_002251	0.000178911	1.2276E-05	2.03405	down			
CB_014805	0.000178911	1.2278E-05	2.735073	down	4601	MXI1	
CB_024934	0.000178634	1.2239E-05	2.155923	down	51776	ZAK	
CB_008597	0.000177795	1.2162E-05	3.625342	down	55638	SYBU	
CB_020303	0.000177563	1.214E-05	3.216448	down	54621	VSIG10	
CB_023150	0.0001775	1.2133E-05	6.338257	down	56137	PCDHA12	
CB_023085	0.000177479	1.2123E-05	3.220134	down	83604	TMEM47	
CB_015764	0.000177048	1.2088E-05	2.544533	down	6515	SLC2A3	
CB_025080	0.000176977	1.2081E-05	2.570339	down	115207	KCTD12	
CB_026252	0.000175753	1.1952E-05	2.214198	down	159195	USP54	
CB_012782	0.000175537	1.1927E-05	2.6399	down	8809	IL18R1	
CB_012129	0.000174669	1.185E-05	2.151211	down	6776	STAT5A	
CB_028354	0.000174669	1.1846E-05	3.473926	down	6035	RNASE1	
CB_016594	0.000174409	1.1825E-05	5.841151	down	745	C11orf9	
CB_028074	0.000173162	1.1708E-05	2.387653	down	4681	NBL1	
CB_009830	0.00017227	1.1637E-05	3.41555	down	401124	DTHD1	
CB_019886	0.000171181	1.1544E-05	3.230889	down	55321	C20orf46	
CB_000495	0.000170982	1.1509E-05	4.691434	down			
CB_008346	0.000170533	1.146E-05	2.124245	down	57545	CC2D2A	
CB_020605	0.000170371	1.1435E-05	2.061857	down	57134	MAN1C1	
CB_014663	0.00017008	1.1405E-05	2.944235	down	10219	KLRG1	
CB_022962	0.000169585	1.1364E-05	2.800575	down	81848	SPRY4	
CB_014564	0.000169057	1.1326E-05	2.32638	down	10076	PTPRU	
CB_020081	0.000168242	1.126E-05	2.732242	down	55902	ACSS2	
CB_031156	0.000167918	1.1233E-05	2.359538	down	730102	LOC730102	
CB_011932	0.000167918	1.1233E-05	3.060769	down	6272	SORT1	
CB_015155	0.000166836	1.115E-05	5.044708	down	8406	SRPX	
CB_010548	0.00016669	1.1133E-05	3.73625	down	3400	ID4	
CB_031042	0.00016669	1.1135E-05	4.247068	down	400456	LOC400456	
CB_020769	0.00016669	1.1135E-05	4.934003	down	57452	GALNTL1	
CB_022269	0.000166183	1.1094E-05	2.056252	down	28971	C11orf67	
CB_017106	0.000165009	1.0992E-05	2.44271	down	27244	SESN1	
CB_010892	0.000164885	1.0978E-05	2.523632	down	1634	DCN	
CB_029400	0.0001648	1.0968E-05	2.937172	down	7754	ZNF204P	
CB_008528	0.000163709	1.0877E-05	5.513251	down	729238	SFTPA2	
CB_005719	0.000163525	1.0859E-05	3.480587	down	4256	MGP	
CB_004026	0.000163394	1.0848E-05	3.001451	down			
CB_020758	0.00016237	1.0754E-05	2.433742	down	57406	ABHD6	
CB_027215	0.000161638	1.0685E-05	3.24155	down	128346	C1orf162	
CB_023137	0.000161586	1.0679E-05	2.364374	down	2793	GNGT2	
CB_023116	0.000161525	1.0673E-05	2.871531	down	83699	SH3BGRL2	

CB_007231	0.000161401	1.066E-05	2.06054	down	3176	HNMT	
CB_024231	0.000161401	1.0662E-05	2.518826	down	89853	FAM125B	
CB_003632	0.000160181	1.0558E-05	2.644392	down			
CB_013786	0.000159102	1.0459E-05	2.706446	down	9592	IER2	
CB_004977	0.000156296	1.0221E-05	3.387142	down	2206	MS4A2	
CB_007004	0.000152042	9.9038E-06	2.628671	down	343521	TCTEX1D4	
CB_008547	0.000151812	9.8864E-06	2.307218	down	653567	FAM23A	
CB_019152	0.000151458	9.8585E-06	2.286194	down	54764	ZRANB1	
CB_005680	0.000150741	9.7856E-06	8.236585	down	3248	HPGD	
CB_024043	0.000150197	9.7384E-06	3.275442	down	85407	NKD1	
CB_024300	0.000148942	9.6381E-06	2.035366	down	90634	N4BP2L1	
CB_027908	0.000148776	9.625E-06	2.536655	down	353514	LILRA5	
CB_029833	0.000147953	9.5624E-06	4.231149	down	56967	C14orf132	
CB_023961	0.000147885	9.5533E-06	7.616409	down	4969	OGN	
CB_024920	0.000147289	9.5031E-06	2.351843	down	1634	DCN	
CB_016697	0.000146148	9.4064E-06	2.045056	down	23670	TMEM2	
CB_009084	0.000146056	9.3933E-06	2.148998	down	84141	FAM176A	
CB_020826	0.000144966	9.3119E-06	2.938772	down	57507	ZNF608	
CB_027540	0.000144192	9.2484E-06	2.028593	down	283349	RASSF3	
CB_016422	0.000143351	9.1866E-06	2.205653	down	23555	TSPAN15	
CB_001614	0.000142821	9.1379E-06	4.573451	down			
CB_014132	0.000141865	9.0558E-06	2.168103	down	2678	GGT1	
CB_023695	0.000141776	9.0463E-06	2.309861	down	84674	CARD6	
CB_028640	0.000141607	9.0267E-06	2.652943	down	91227	GGTLC2	
CB_009538	0.00014111	8.9881E-06	5.850081	down	6563	SLC14A1	
CB_018929	0.000140889	8.9718E-06	2.781375	down	6926	TBX3	
CB_017552	0.00014061	8.9496E-06	2.174552	down	2744	GLS	
CB_012938	0.000139081	8.8287E-06	2.871426	down	388	RHOB	
CB_023294	0.000139026	8.8069E-06	2.981665	down	56099	PCDHGB7	
CB_022906	0.000138196	8.7478E-06	2.246986	down	81606	LBH	
CB_011419	0.000137795	8.7202E-06	2.079043	down	4478	MSN	
CB_015105	0.000137694	8.7116E-06	3.182453	down	5592	PRKG1	
CB_028652	0.000137258	8.671E-06	3.955336	down	6387	CXCL12	
CB_020680	0.000136662	8.6246E-06	2.441463	down	57217	TTC7A	
CB_011429	0.000136662	8.6236E-06	5.055399	down	4582	MUC1	
CB_028460	0.000136146	8.5726E-06	3.533619	down	343450	KCNT2	
CB_022660	0.000135899	8.5506E-06	2.47959	down	5362	PLXNA2	
CB_026719	0.000135784	8.5395E-06	2.112397	down	128077	LIX1L	
CB_015499	0.000135784	8.5413E-06	2.587082	down	10800	CYSLTR1	
CB_023139	0.000135784	8.5398E-06	2.734911	down	56143	PCDHA5	
CB_007732	0.000135784	8.5388E-06	3.029405	down	5737	PTGFR	
CB_015724	0.000135178	8.4812E-06	2.234152	down	11041	B3GNT1	
CB_002653	0.000134813	8.4481E-06	2.662059	down			
CB_014697	0.000134419	8.4171E-06	2.016705	down	10268	RAMP3	
CB_003183	0.00013394	8.3828E-06	2.018282	down			
CB_010226	0.000133497	8.3466E-06	6.336138	down	651	BMP3	
CB_023883	0.000131655	8.2085E-06	3.683841	down	84953	MICALCL	
CB_002468	0.000131492	8.1962E-06	3.292958	down			
CB_022110	0.000130696	8.1321E-06	3.100685	down	79442	LRRC2	
CB_026836	0.000130263	8.0969E-06	2.853474	down	8741	TNFSF13	
CB_012019	0.000130212	8.0917E-06	2.028635	down	6489	ST8SIA1	
CB_004926	0.000129821	8.0612E-06	3.714734	down	1286	COL4A4	
CB_027903	0.000129385	8.0239E-06	2.553237	down	347902	AMIGO2	
CB_005055	0.000128903	7.9858E-06	4.887351	down	3730	KAL1	
CB_008647	0.00012872	7.9704E-06	23.66072	down	253970	SFTA3	
CB_027407	0.000128587	7.9601E-06	7.25159	down	155465	AGR3	

CB_021332	0.000128554	7.956E-06	2.800168	down	60370	AVPI1	
CB_005419	0.00012844	7.9429E-06	2.801486	down	3570	IL6R	
CB_024943	0.000128112	7.9124E-06	2.09119	down	5998	RGS3	
CB_028348	0.000127336	7.8489E-06	2.237082	down	2040	STOM	
CB_019222	0.000126943	7.815E-06	2.777549	down	54810	GIPC2	
CB_028778	0.000126137	7.7405E-06	2.625458	down	57447	NDRG2	
CB_023545	0.000125218	7.6662E-06	2.493902	down	5098	PCDHGC3	
CB_025329	0.000125115	7.6559E-06	6.184786	down	84525	HOPX	
CB_016593	0.000124402	7.5926E-06	3.016987	down	745	C11orf9	
CB_010132	0.000123887	7.5552E-06	3.899778	down	4660	PPP1R12B	
CB_028524	0.000123382	7.5127E-06	9.122138	down	120892	LRRK2	
CB_026559	0.000123118	7.4947E-06	3.166935	down	168537	GIMAP7	
CB_009602	0.000121849	7.4007E-06	3.01911	down	256435	ST6GALNAC3	
CB_009065	0.000121849	7.4039E-06	3.396232	down	4774	NFIA	
CB_006440	0.000120901	7.3195E-06	3.316396	down	345557	PLCXD3	
CB_013821	0.000120232	7.2676E-06	6.943422	down	1776	DNASE1L3	
CB_018108	0.00011896	7.1769E-06	2.22424	down	25939	SAMHD1	
CB_020836	0.000118958	7.1719E-06	2.940396	down	57519	STARD9	
CB_028526	0.000118754	7.1538E-06	2.127726	down	376497	SLC27A1	
CB_016528	0.000118355	7.1166E-06	2.137043	down	23639	LRRC6	
CB_016560	0.00011786	7.077E-06	2.542532	down	7761	ZNF214	
CB_016419	0.000117781	7.069E-06	2.32155	down	4542	MYO1F	
CB_005282	0.000117253	7.0294E-06	2.765186	down	4053	LTBP2	
CB_028318	0.000117102	7.0156E-06	2.106304	down	2595	GANC	
CB_023512	0.000117102	7.0165E-06	4.544442	down	84316	LSMD1	
CB_008246	0.00011648	6.9688E-06	4.901718	down	114800	CCDC85A	
CB_019862	0.000115887	6.9204E-06	3.603557	down	55304	SPTLC3	
CB_011455	0.000115288	6.8791E-06	2.819096	down	4660	PPP1R12B	
CB_020704	0.000115272	6.8764E-06	2.309049	down	4792	NFKBIA	
CB_013557	0.000115266	6.8742E-06	4.368545	down	8404	SPARCL1	
CB_020885	0.000115129	6.8624E-06	3.060521	down	57569	ARHGAP20	
CB_015725	0.000114145	6.7892E-06	2.405379	down	2766	GMPR	
CB_022811	0.0001139	6.7711E-06	2.103725	down	6934	TCF7L2	
CB_003779	0.000113199	6.7204E-06	8.290422	down			
CB_022346	0.000113087	6.712E-06	2.317448	down	79817	MOBKL2B	
CB_008824	0.000112937	6.7013E-06	2.896204	down	149483	CCDC17	
CB_018898	0.000112116	6.6437E-06	2.165145	down	51762	RAB8B	
CB_014739	0.000112085	6.6383E-06	2.12547	down	4092	SMAD7	
CB_021342	0.000111948	6.6266E-06	5.135373	down	27159	CHIA	
CB_028116	0.000111778	6.613E-06	2.365626	down	11259	FILIP1L	
CB_006024	0.000110871	6.5418E-06	2.154857	down	79971	WLS	
CB_017605	0.000110751	6.5314E-06	2.465154	down	22898	DENND3	
CB_010023	0.000110751	6.5321E-06	2.557816	down	3572	IL6ST	
CB_021600	0.000110743	6.5272E-06	8.699699	down	64168	NECAB1	
CB_005443	0.000110477	6.5028E-06	7.416318	down	3569	IL6	
CB_010021	0.000110221	6.4824E-06	2.682111	down	3572	IL6ST	
CB_011985	0.000109597	6.4349E-06	3.205	down	6403	SELP	
CB_016768	0.000109304	6.4112E-06	2.261254	down	28954	REM1	
CB_016076	0.000109304	6.4107E-06	4.043804	down	11343	MGLL	
CB_008245	0.000108868	6.3787E-06	2.539022	down	114800	CCDC85A	
CB_013227	0.000107328	6.2664E-06	6.741926	down	820	CAMP	
CB_015804	0.000106866	6.236E-06	3.981707	down	8013	NR4A3	
CB_029076	0.00010627	6.1895E-06	3.505124	down	344148	NCKAP5	
CB_006805	0.000105942	6.1648E-06	2.551484	down	401494	PTPLAD2	
CB_023285	0.000105333	6.122E-06	2.743248	down	56107	PCDHGA9	
CB_022905	0.000105118	6.0957E-06	2.149465	down	81606	LBH	

CB_004758	0.000104825	6.0705E-06	2.08818	down			
CB_028475	0.000104788	6.0666E-06	3.162148	down	374378	GALNTL4	
CB_005142	0.000104706	6.0601E-06	2.193003	down	5311	PKD2	
CB_013266	0.000104053	6.0207E-06	4.057541	down	1512	CTSH	
CB_005715	0.000103956	6.0122E-06	2.461826	down	4048	LTA4H	
CB_005517	0.000103956	6.0134E-06	4.03112	down	217	ALDH2	
CB_017851	0.000103847	6.0039E-06	2.194238	down	23243	ANKRD28	
CB_012817	0.000103612	5.9886E-06	3.064575	down	8869	ST3GAL5	
CB_010971	0.000103287	5.9649E-06	3.404689	down	2219	FCN1	
CB_028769	0.000103106	5.9496E-06	2.304695	down	8522	GAS7	
CB_013734	0.000103095	5.9473E-06	19.8756	down	9476	NAPSA	
CB_021565	0.000103038	5.9424E-06	6.014967	down	64116	SLC39A8	
CB_024191	0.000103037	5.9407E-06	2.117769	down	85464	SSH2	
CB_014070	0.000102833	5.9257E-06	2.763607	down	1326	MAP3K8	
CB_023083	0.000102118	5.8714E-06	2.176137	down	83595	SOX7	
CB_005207	0.000102007	5.8587E-06	2.648172	down	412	STS	
CB_016305	0.000101957	5.8542E-06	2.329092	down	22921	MSRB2	
CB_009664	0.00010157	5.8287E-06	2.279211	down	322	APBB1	
CB_022414	0.00010157	5.8277E-06	3.097801	down	79888	LPCAT1	
CB_020972	0.000101281	5.8042E-06	3.392107	down	57669	EPB41L5	
CB_008202	0.000100597	5.7554E-06	3.042011	down	23514	KIAA0146	
CB_011933	0.000100431	5.7394E-06	2.495922	down	6274	S100A3	
CB_017120	0.000100431	5.7395E-06	7.380785	down	27289	RND1	
CB_015340	0.000100205	5.7193E-06	2.859538	down	2192	FBLN1	
CB_007536	0.000100205	5.7194E-06	3.019292	down	619208	C6orf225	
CB_000452	0.000100196	5.7134E-06	2.625713	down	100129572	LOC100129572	
CB_005116	9.99866E-05	5.6967E-06	2.055357	down	4867	NPHP1	
CB_021652	9.99464E-05	5.688E-06	6.659863	down	64344	HIF3A	
CB_025469	9.92441E-05	5.6418E-06	4.249919	down	137872	ADHFE1	
CB_022721	9.92048E-05	5.638E-06	4.46829	down	80704	SLC19A3	
CB_031009	9.81831E-05	5.5706E-06	2.363136	down	100132741	LOC100132741	
CB_003247	9.80191E-05	5.5567E-06	2.05465	down	11346	SYNPO	
CB_014048	9.80191E-05	5.5597E-06	5.713368	down	761	CA3	
CB_005069	9.79181E-05	5.5478E-06	4.7644	down	6445	SGCG	
CB_027774	9.79174E-05	5.5448E-06	2.22131	down	10398	MYL9	
CB_010249	9.77574E-05	5.5294E-06	4.166212	down	858	CAV2	
CB_015441	9.76672E-05	5.5212E-06	4.765036	down	27233	SULT1C4	
CB_007946	9.73151E-05	5.4874E-06	2.480484	down	831	CAST	
CB_006732	9.7219E-05	5.4773E-06	2.213217	down	25943	C20orf194	
CB_022640	9.71261E-05	5.465E-06	2.612596	down	80223	RAB11FIP1	
CB_009156	9.68804E-05	5.4414E-06	3.164484	down	26052	DNM3	
CB_022679	9.67629E-05	5.4317E-06	2.16126	down	80301	PLEKHO2	
CB_013780	9.65879E-05	5.4188E-06	2.103372	down	9575	CLOCK	
CB_015232	9.65879E-05	5.4184E-06	2.193471	down	10493	VAT1	
CB_021479	9.65879E-05	5.4161E-06	2.346018	down	63895	FAM38B	
CB_016382	9.658E-05	5.4138E-06	2.383932	down	23194	FBXL7	
CB_007551	9.64034E-05	5.4024E-06	2.806483	down	6387	CXCL12	
CB_017186	9.58092E-05	5.3675E-06	3.110876	down	26508	HEYL	
CB_004970	9.57296E-05	5.36E-06	3.15674	down	2157	F8	
CB_015440	9.53892E-05	5.3349E-06	3.37939	down	27233	SULT1C4	
CB_012806	9.51313E-05	5.3175E-06	2.510681	down	8839	WISP2	
CB_027545	9.38722E-05	5.2253E-06	2.235559	down	339834	CCDC36	
CB_016029	9.38722E-05	5.2278E-06	2.310648	down	11264	PXMP4	
CB_027250	9.38722E-05	5.2266E-06	3.652154	down	254295	PHYHD1	
CB_010644	9.38722E-05	5.2207E-06	4.963214	down	374	AREG	
CB_024660	9.33902E-05	5.1758E-06	4.228322	down	140706	C20orf160	

CB_020601	9.31727E-05	5.1556E-06	2.88514	down	57104	PNPLA2	
CB_009399	0.000093166	5.152E-06	5.58922	down	387763	C11orf96	
CB_015233	9.29151E-05	5.1354E-06	2.366312	down	10493	VAT1	
CB_026506	9.2443E-05	5.1013E-06	2.329182	down	144165	PRICKLE1	
CB_005539	9.23595E-05	5.0928E-06	8.585255	down	722	C4BPA	
CB_024940	9.20494E-05	5.0708E-06	2.889519	down	3241	HPCAL1	
CB_027476	9.14993E-05	5.0245E-06	3.017709	down	427	ASAH1	
CB_008470	9.14325E-05	5.0194E-06	9.863714	down	21	ABCA3	
CB_008614	9.13899E-05	5.0142E-06	2.2624	down	9749	PHACTR2	
CB_022352	9.07792E-05	4.9749E-06	2.621181	down	10395	DLC1	
CB_008474	9.06323E-05	4.9654E-06	5.234159	down	32	ACACB	
CB_018040	9.05334E-05	4.9543E-06	3.80042	down	25854	FAM149A	
CB_007877	9.00156E-05	4.916E-06	2.136532	down	152098	ZCWPW2	
CB_022746	8.98805E-05	4.9015E-06	12.43185	down	80761	UPK3B	
CB_013390	8.8774E-05	4.82E-06	2.722767	down	3603	IL16	
CB_028842	8.8707E-05	4.815E-06	4.02731	down	51703	ACSL5	
CB_011524	8.86804E-05	4.8093E-06	2.58291	down	4983	OPHN1	
CB_024877	8.84758E-05	4.7954E-06	4.521145	down	7273	TTN	
CB_014756	8.83159E-05	4.7839E-06	2.330813	down	4209	MEF2D	
CB_029791	8.82328E-05	4.7752E-06	2.813213	down	441094	FLJ42709	
CB_011351	8.81356E-05	4.7672E-06	3.49186	down	4118	MAL	
CB_020651	8.80922E-05	4.7633E-06	2.076381	down	57153	SLC44A2	
CB_021226	8.80892E-05	4.7605E-06	5.260418	down	58189	WFDC1	
CB_006852	8.74752E-05	4.7154E-06	3.253138	down	84460	ZMAT1	
CB_004661	8.73858E-05	4.7073E-06	2.302998	down			
CB_026154	8.64729E-05	4.6444E-06	2.650363	down	146556	C16orf89	
CB_012767	8.60573E-05	4.6101E-06	4.269652	down	8793	TNFRSF10D	
CB_013698	8.58523E-05	4.592E-06	2.923843	down	9413	FAM189A2	
CB_029009	8.57269E-05	4.5839E-06	2.525762	down	2549	GAB1	
CB_025198	8.5503E-05	4.5659E-06	3.981981	down	116441	TM4SF18	
CB_019969	8.5503E-05	4.5633E-06	6.07643	down	55363	HEMGN	
CB_021409	8.50988E-05	4.5315E-06	2.11907	down	3142	HLX	
CB_015129	8.48799E-05	4.5154E-06	2.352222	down	6867	TACC1	
CB_026700	8.48799E-05	4.5166E-06	2.366448	down	91526	ANKRD44	
CB_013117	8.48799E-05	4.5171E-06	5.283647	down	9314	KLF4	
CB_017266	8.48319E-05	4.5091E-06	2.326864	down	9674	KIAA0040	
CB_010305	8.38295E-05	4.4408E-06	2.922969	down	1193	CLIC2	
CB_018279	8.38295E-05	4.4412E-06	2.964887	down	27147	DENND2A	
CB_006758	8.37257E-05	4.4291E-06	2.076299	down	170371	C10orf128	
CB_023966	8.25884E-05	4.3422E-06	2.32481	down	85360	SYDE1	
CB_018225	8.25841E-05	4.336E-06	2.328053	down	26112	CCDC69	
CB_023555	8.23659E-05	4.3193E-06	2.754185	down	84418	C5orf32	
CB_018229	8.20065E-05	4.2888E-06	2.021043	down	26118	WSB1	
CB_014131	8.15407E-05	4.2592E-06	2.533441	down	2678	GGT1	
CB_007712	8.11363E-05	4.2304E-06	2.366886	down	55423	SIRPG	
CB_017880	8.11174E-05	4.2281E-06	3.146335	down	440073	IQSEC3	
CB_010743	8.10947E-05	4.2244E-06	2.488576	down	896	CCND3	
CB_010989	8.07585E-05	4.1992E-06	2.833099	down	2313	FLI1	
CB_013607	8.06616E-05	4.1916E-06	2.092053	down	9185	REPS2	
CB_026721	8.04482E-05	4.1728E-06	10.83956	down	256815	C10orf67	
CB_029572	8.01179E-05	4.1481E-06	2.446016	down	2679	GGT3P	
CB_015920	7.96765E-05	4.1176E-06	2.494803	down	7402	UTRN	
CB_021635	7.95905E-05	4.1074E-06	6.410173	down	64284	RAB17	
CB_008569	7.95647E-05	4.1043E-06	2.601091	down	8910	SGCE	
CB_023183	7.94874E-05	4.0991E-06	7.735326	down	83850	ESYT3	
CB_028180	7.93483E-05	4.0884E-06	2.672776	down	140738	TMEM37	

CB_016389	7.93483E-05	4.0894E-06	3.877256	down	22941	SHANK2	
CB_016059	7.93483E-05	4.0891E-06	4.239116	down	11326	VSIG4	
CB_008418	7.91424E-05	4.0717E-06	2.959957	down	4919	ROR1	
CB_013954	7.88208E-05	4.0509E-06	2.076908	down	6560	SLC12A4	
CB_007837	7.70871E-05	3.9386E-06	6.726773	down	4629	MYH11	
CB_013184	7.69468E-05	3.9254E-06	3.659252	down	9630	GNA14	
CB_015597	7.67303E-05	3.9082E-06	6.831242	down	5950	RBP4	
CB_002389	7.64284E-05	3.8819E-06	11.65939	down			
CB_028078	7.63457E-05	3.8765E-06	2.767161	down	255488	RNF144B	
CB_031544	7.63457E-05	3.8762E-06	2.79257	down	100506601	LOC100506601	
CB_030635	7.63378E-05	3.8737E-06	10.23663	down	723809	LOC723809	
CB_016938	7.62662E-05	3.8689E-06	3.179462	down	11173	ADAMTS7	
CB_022145	7.61072E-05	3.8572E-06	4.553008	down	79611	ACSS3	
CB_007838	7.58033E-05	3.8322E-06	6.432536	down	4629	MYH11	
CB_020123	7.5674E-05	3.8244E-06	3.503716	down	55930	MYO5C	
CB_014961	7.48504E-05	3.7757E-06	3.372842	down	3108	HLA-DMA	
CB_028915	7.46632E-05	3.7584E-06	2.38473	down	389125	MUSTN1	
CB_023182	7.46632E-05	3.7591E-06	4.776586	down	114905	C1QTNF7	
CB_006605	7.43279E-05	3.7269E-06	2.77491	down	55075	UACA	
CB_005084	7.41257E-05	3.7156E-06	2.199011	down	100507436	MICA	
CB_004875	7.36577E-05	3.6824E-06	3.499363	down	367	AR	
CB_003310	7.29826E-05	3.6364E-06	3.445943	down	5137	PDE1C	
CB_001240	7.29232E-05	3.6311E-06	3.343932	down	283624	LOC283624	
CB_010627	7.19221E-05	3.5584E-06	2.193736	down	334	APLP2	
CB_010912	7.18156E-05	3.5509E-06	2.78571	down	1848	DUSP6	
CB_031983	7.17452E-05	3.5451E-06	3.096266	down	644192	LOC644192	
CB_026632	7.13038E-05	3.5048E-06	2.343761	down	219654	ZCCHC24	
CB_008251	7.13038E-05	3.5139E-06	2.48325	down	126374	WTIP	
CB_025393	7.13038E-05	3.5165E-06	2.52791	down	91624	NEXN	
CB_029770	7.13038E-05	3.51E-06	2.533278	down	378805	FLJ43663	
CB_008612	7.13038E-05	3.5163E-06	2.58676	down	10335	MRVI1	
CB_025349	7.13038E-05	3.5164E-06	2.912723	down	203190	LGI3	
CB_017326	7.11196E-05	3.4939E-06	3.03701	down	9728	SECISBP2L	
CB_003717	7.04901E-05	3.4485E-06	2.634099	down			
CB_028313	7.04719E-05	3.4465E-06	6.864181	down	358	AQP1	
CB_008032	6.9935E-05	3.4158E-06	8.278296	down	6532	SLC6A4	
CB_006025	6.97142E-05	3.4038E-06	3.082049	down	2328	FMO3	
CB_018553	6.966E-05	3.3957E-06	2.513505	down	51393	TRPV2	
CB_026709	6.966E-05	3.3956E-06	2.795476	down	127435	PODN	
CB_017053	6.95794E-05	3.3896E-06	16.28759	down	27063	ANKRD1	
CB_011169	6.90406E-05	3.3546E-06	2.487011	down	3572	IL6ST	
CB_004908	6.90406E-05	3.3541E-06	3.528974	down	825	CAPN3	
CB_011375	6.8876E-05	3.3408E-06	2.519481	down	4211	MEIS1	
CB_013980	6.85879E-05	3.3228E-06	4.607545	down	9590	AKAP12	
CB_009484	6.85842E-05	3.3204E-06	2.805607	down	23331	TTC28	
CB_003267	6.84937E-05	3.3122E-06	8.028634	down	6532	SLC6A4	
CB_009367	6.84612E-05	3.3069E-06	4.342859	down	23327	NEDD4L	
CB_016680	6.78629E-05	3.2726E-06	2.22466	down	10015	PDCD6IP	
CB_005720	6.77754E-05	3.2662E-06	5.497245	down	4306	NR3C2	
CB_005358	6.77667E-05	3.2647E-06	5.351567	down	2203	FBP1	
CB_026701	6.72081E-05	3.2284E-06	2.278426	down	195827	C9orf21	
CB_017549	6.69552E-05	3.2139E-06	2.871333	down	89795	NAV3	
CB_014678	6.66123E-05	3.1891E-06	2.659892	down	10241	CALCOCO2	
CB_020915	6.65579E-05	3.1843E-06	2.605594	down	57600	FNIP2	
CB_024317	6.62995E-05	3.1667E-06	3.110512	down	2788	GNG7	
CB_002884	6.62677E-05	3.1631E-06	3.601737	down			

CB_012783	6.61347E-05	3.1557E-06	3.038183	down	8809	IL18R1	
CB_011986	6.61112E-05	3.1535E-06	2.988193	down	6404	SELPLG	
CB_010018	6.58228E-05	3.1325E-06	5.091297	down	100127983	LOC100127983	
CB_007676	6.56323E-05	3.1188E-06	3.274279	down	11078	TRIOBP	
CB_010920	6.56041E-05	3.1148E-06	5.202741	down	1906	EDN1	
CB_008599	6.53115E-05	3.0968E-06	3.46912	down	152078	C3orf55	
CB_019868	6.52421E-05	3.0916E-06	2.557445	down	54674	LRRN3	
CB_013625	6.52421E-05	3.0924E-06	3.138216	down	9223	MAGI1	
CB_013004	6.52421E-05	3.0917E-06	4.451708	down	2202	EFEMP1	
CB_025193	6.51704E-05	3.0859E-06	2.308427	down	115548	FCHO2	
CB_017843	6.51704E-05	3.0849E-06	2.473167	down	23235	SIK2	
CB_021705	6.48016E-05	3.0586E-06	2.37949	down	7145	TNS1	
CB_014539	6.44531E-05	3.0366E-06	2.343117	down	7905	REEP5	
CB_022416	6.43707E-05	3.0303E-06	2.200117	down	79890	RIN3	
CB_031837	6.41963E-05	3.0184E-06	2.906075	down	388692	LOC388692	
CB_016484	6.4084E-05	3.0099E-06	2.092974	down	6251	RSU1	
CB_008850	6.37263E-05	2.9893E-06	2.763543	down	284759	SIRPB2	
CB_009321	6.37263E-05	2.9888E-06	10.13298	down	91851	CHRDL1	
CB_020035	6.34664E-05	2.971E-06	3.39552	down	55450	CAMK2N1	
CB_017237	6.28522E-05	2.9393E-06	2.878403	down	9639	ARHGEF10	
CB_018241	6.27301E-05	2.9286E-06	2.326818	down	26137	ZBTB20	
CB_029525	6.24822E-05	2.9131E-06	5.764471	down	150000	ABCC13	
CB_018281	6.20828E-05	2.8895E-06	2.135634	down	55841	WWC3	
CB_007653	6.16665E-05	2.8623E-06	2.587098	down	85458	DIXDC1	
CB_007839	6.14446E-05	2.8462E-06	2.014339	down	116985	ARAP1	
CB_013067	6.14446E-05	2.8435E-06	2.055578	down	6809	STX3	
CB_025018	6.14446E-05	2.8471E-06	3.517082	down	91768	CABLES1	
CB_013945	6.14331E-05	2.8408E-06	3.644891	down	6368	CCL23	
CB_020810	6.13781E-05	2.8363E-06	2.605263	down	57493	HEG1	
CB_026944	6.06305E-05	2.7816E-06	2.629186	down	150094	SIK1	
CB_003376	6.01435E-05	2.7526E-06	2.268868	down			
CB_011874	5.92077E-05	2.6891E-06	2.022755	down	5962	RDX	
CB_016997	5.87996E-05	2.6622E-06	5.174988	down	23705	CADM1	
CB_003071	5.86088E-05	2.647E-06	2.413127	down			
CB_008986	5.86088E-05	2.6459E-06	3.398176	down	23348	DOCK9	
CB_017971	5.86088E-05	2.6448E-06	4.072144	down	23382	AHCYL2	
CB_027453	5.85093E-05	2.6388E-06	2.01542	down	23673	STX12	
CB_024982	5.84256E-05	2.6323E-06	3.753047	down	160364	CLEC12A	
CB_007895	5.82832E-05	2.6197E-06	5.304286	down	79908	BTNL8	
CB_020534	5.81773E-05	2.6119E-06	2.033179	down	56994	CHPT1	
CB_026203	5.77547E-05	2.5837E-06	9.386371	down	151126	ZNF385B	
CB_004925	5.72844E-05	2.5545E-06	3.282445	down	1285	COL4A3	
CB_026948	5.71254E-05	2.5454E-06	2.997246	down	317649	EIF4E3	
CB_002843	5.70741E-05	2.5415E-06	3.31335	down			
CB_022221	5.69845E-05	2.5366E-06	7.190983	down	79689	STEAP4	
CB_025449	5.69049E-05	2.5295E-06	2.90085	down	130132	RFTN2	
CB_008630	5.57802E-05	2.4583E-06	2.673264	down	58191	CXCL16	
CB_029891	5.57546E-05	2.4547E-06	2.027021	down	84852	ATP1A1OS	
CB_020888	5.57546E-05	2.4554E-06	2.137025	down	57572	DOCK6	
CB_026463	5.55171E-05	2.4335E-06	2.734912	down	260425	MAGI3	
CB_015040	5.54897E-05	2.4296E-06	2.813032	down	5141	PDE4A	
CB_007733	5.53049E-05	2.4198E-06	2.103054	down	8239	USP9X	
CB_019287	5.48685E-05	2.3946E-06	2.112981	down	54863	C9orf167	
CB_028934	5.48087E-05	2.3911E-06	3.712377	down	387496	RASL11A	
CB_011889	5.45801E-05	2.3768E-06	3.532113	down	5997	RGS2	
CB_021417	5.45801E-05	2.3745E-06	11.42384	down	5239	PGM5	

CB_022017	5.39485E-05	2.3373E-06	9.010716	down	79083	MLPH	
CB_015265	5.37303E-05	2.3245E-06	4.262284	down	4739	NEDD9	
CB_013942	5.35995E-05	2.318E-06	4.648439	down	6097	RORC	
CB_007107	5.35828E-05	2.3138E-06	3.550643	down	9060	PAPSS2	
CB_008370	5.3524E-05	2.3071E-06	3.155374	down	6688	SPI1	
CB_019466	5.32621E-05	2.2941E-06	2.071956	down	53373	TPCN1	
CB_015754	5.30719E-05	2.2796E-06	5.826254	down	6258	RXRG	
CB_014913	5.29693E-05	2.2731E-06	2.349925	down	10326	SIRPB1	
CB_028380	5.29637E-05	2.272E-06	2.584826	down	4013	VWA5A	
CB_012238	5.26978E-05	2.2546E-06	3.56527	down	7134	TNNC1	
CB_017923	5.25091E-05	2.2433E-06	3.143241	down	23328	SASH1	
CB_004848	5.24719E-05	2.2401E-06	2.711662	down	6442	SGCA	
CB_031554	5.23922E-05	2.235E-06	3.627435	down	100506393	LOC100506393	
CB_011168	5.22263E-05	2.2213E-06	2.281077	down	3572	IL6ST	
CB_026259	5.19267E-05	2.2045E-06	3.397867	down	161176	C14orf49	
CB_012621	5.17746E-05	2.1955E-06	2.18014	down	8566	PDXK	
CB_015474	5.17361E-05	2.1903E-06	3.104899	down	10763	NES	
CB_026338	5.11448E-05	2.1551E-06	2.062436	down	202052	DNAJC18	
CB_007659	5.10775E-05	2.1474E-06	4.67541	down	284654	RSPO1	
CB_006925	5.10775E-05	2.1462E-06	6.561944	down	196996	GRAMD2	
CB_006599	5.09309E-05	2.1363E-06	2.230779	down	10133	OPTN	
CB_000016	5.08464E-05	2.1296E-06	2.864688	down	643616	MOP-1	
CB_026208	5.05613E-05	2.1136E-06	3.384542	down	117583	PARD3B	
CB_027075	5.00147E-05	2.0844E-06	3.130261	down	146177	VWA3A	
CB_027338	4.95849E-05	2.0585E-06	3.117247	down	326624	RAB37	
CB_027528	4.95849E-05	2.0592E-06	3.855033	down	199974	CYP4Z1	
CB_020237	4.95464E-05	2.0534E-06	2.71573	down	54491	FAM105A	
CB_005494	4.95464E-05	2.0539E-06	20.60552	down	126	ADH1C	
CB_010045	4.93912E-05	2.0428E-06	3.289262	down	253827	MSRB3	
CB_010643	4.92881E-05	2.037E-06	5.345304	down	374	AREG	
CB_010948	4.91693E-05	2.0297E-06	2.12368	down	2054	STX2	
CB_012715	4.89996E-05	2.0194E-06	2.542649	down	8722	CTSF	
CB_017327	4.89274E-05	2.0078E-06	6.045583	down	9729	KIAA0408	
CB_021527	4.87576E-05	1.9973E-06	2.545282	down	64061	TSPYL2	
CB_013014	4.86551E-05	1.99E-06	2.387821	down	2289	FKBP5	
CB_015736	4.86373E-05	1.9885E-06	2.069816	down	3176	HNMT	
CB_022520	4.8627E-05	1.9865E-06	3.988267	down	80014	WWC2	
CB_007034	4.83359E-05	1.9708E-06	3.528059	down	440503	PLIN5	
CB_006184	4.83304E-05	1.9698E-06	2.649782	down	440738	MAP1LC3C	
CB_002191	4.81051E-05	1.9571E-06	2.727865	down			
CB_017857	4.81051E-05	1.9576E-06	3.50148	down	23250	ATP11A	
CB_013492	4.80521E-05	1.9539E-06	3.79322	down	7052	TGM2	
CB_019184	4.78884E-05	1.9434E-06	2.023166	down	55599	RNPC3	
CB_012798	4.78232E-05	1.9398E-06	2.16214	down	8829	NRP1	
CB_029189	4.77864E-05	1.9348E-06	3.871281	down	255189	PLA2G4F	
CB_001690	4.75343E-05	1.9185E-06	2.092551	down			
CB_012937	4.74339E-05	1.9115E-06	3.97327	down	388	RHOB	
CB_027674	4.73371E-05	1.9053E-06	2.861911	down	340419	RSPO2	
CB_005528	4.72689E-05	1.9018E-06	8.606607	down	477	ATP1A2	
CB_010337	4.72658E-05	1.9009E-06	5.994802	down	1474	CST6	
CB_014129	4.72281E-05	1.8979E-06	3.122663	down	2674	GFRA1	
CB_003649	4.72098E-05	1.8957E-06	3.930089	down			
CB_024461	4.7151E-05	1.8926E-06	2.669362	down	4638	MYLK	
CB_010256	4.71048E-05	1.89E-06	2.135866	down	902	CCNH	
CB_020837	4.67428E-05	1.8695E-06	3.876317	down	57519	STARD9	
CB_012579	4.66038E-05	1.8632E-06	2.115305	down	8515	ITGA10	

CB_001207	4.65224E-05	1.8592E-06	3.194379	down	11107	PRDM5	
CB_014906	4.64411E-05	1.8553E-06	2.565377	down	10319	LAMC3	
CB_031852	4.62727E-05	1.8463E-06	2.318865	down	574406	C1orf138	
CB_004953	4.6144E-05	1.8397E-06	2.871755	down	2022	ENG	
CB_020207	4.61177E-05	1.8359E-06	3.65711	down	54438	GFOD1	
CB_007118	4.61171E-05	1.8301E-06	2.395281	down	445329	SULT1A4	
CB_015229	4.61171E-05	1.835E-06	2.458819	down	10491	CRTAP	
CB_016483	4.61171E-05	1.8348E-06	2.515766	down	6251	RSU1	
CB_024803	4.61171E-05	1.8329E-06	3.106109	down	170575	GIMAP1	
CB_005620	4.61171E-05	1.8334E-06	6.412283	down	2352	FOLR3	
CB_027275	4.584E-05	1.8142E-06	3.557903	down	284904	SEC14L4	
CB_017431	4.5826E-05	1.8118E-06	2.766646	down	9844	ELMO1	
CB_022296	4.5826E-05	1.8112E-06	3.261966	down	79762	C1orf115	
CB_017550	4.57859E-05	1.8073E-06	2.068487	down	22841	RAB11FIP2	
CB_013733	4.56899E-05	1.7984E-06	10.45282	down	9476	NAPSA	
CB_008837	4.50575E-05	1.7686E-06	2.07529	down	8653	DDX3Y	
CB_013532	4.48976E-05	1.7601E-06	2.910002	down	8313	AXIN2	
CB_005768	4.45962E-05	1.7462E-06	2.154063	down	5638	PRRG1	
CB_013762	4.44025E-05	1.7351E-06	2.627733	down	9535	GMFG	
CB_015017	4.43741E-05	1.7333E-06	5.135121	down	4929	NR4A2	
CB_021102	4.40914E-05	1.7209E-06	4.342888	down	8470	SORBS2	
CB_026293	4.34914E-05	1.6954E-06	2.605642	down	167465	ZNF366	
CB_010437	4.32663E-05	1.6838E-06	4.895696	down	2013	EMP2	
CB_011688	4.31628E-05	1.6791E-06	6.973903	down	5549	PRELP	
CB_016918	4.3079E-05	1.674E-06	2.227514	down	9693	RAPGEF2	
CB_019940	4.28991E-05	1.6628E-06	4.810152	down	55805	LRP2BP	
CB_016611	4.28429E-05	1.6585E-06	2.01393	down	29904	EEF2K	
CB_004890	4.26818E-05	1.6496E-06	5.393926	down	590	BCHE	
CB_012504	4.25271E-05	1.6416E-06	5.411422	down	8399	PLA2G10	
CB_027605	4.23253E-05	1.6291E-06	2.993077	down	128646	SIRPD	
CB_026614	4.2259E-05	1.6259E-06	3.826057	down	135932	TMEM139	
CB_011210	4.21808E-05	1.6215E-06	2.671094	down	3726	JUNB	
CB_016026	4.1683E-05	1.5958E-06	2.033759	down	11261	CHP	
CB_009778	4.15696E-05	1.5901E-06	14.25653	down	5225	PGC	
CB_005077	4.15115E-05	1.585E-06	4.420212	down	4128	MAOA	
CB_011456	4.1423E-05	1.5806E-06	3.216311	down	4660	PPP1R12B	
CB_025253	4.13456E-05	1.5763E-06	4.669233	down	195814	SDR16C5	
CB_012684	4.13456E-05	1.5753E-06	5.559326	down	8671	SLC4A4	
CB_014700	4.12882E-05	1.571E-06	2.05319	down	5813	PURA	
CB_025630	4.10866E-05	1.5593E-06	2.245902	down	221016	CCDC7	
CB_024620	0.000040948	1.5508E-06	3.524221	down	140628	GATA5	
CB_003774	4.09153E-05	1.5485E-06	3.932734	down			
CB_014625	4.08129E-05	1.5418E-06	2.17265	down	10160	FARP1	
CB_021234	4.05132E-05	1.5272E-06	3.088918	down	58480	RHOU	
CB_029607	4.03765E-05	1.5196E-06	2.662437	down	643036	SLED1	
CB_016990	4.03765E-05	1.5202E-06	3.599701	down	23604	DAPK2	
CB_015006	4.00579E-05	1.5037E-06	5.691443	down	4900	NRGN	
CB_021551	4.005E-05	1.5028E-06	2.698855	down	64097	EPB41L4A	
CB_011166	3.99905E-05	1.498E-06	2.74672	down	3572	IL6ST	
CB_017606	3.97078E-05	1.4843E-06	2.555044	down	22898	DENND3	
CB_014456	3.9579E-05	1.4732E-06	2.840869	down	4781	NFIB	
CB_002447	3.9579E-05	1.4749E-06	3.036019	down			
CB_023154	3.9579E-05	1.4744E-06	3.481689	down	56134	PCDHAC2	
CB_015018	3.95686E-05	1.4703E-06	5.645175	down	4929	NR4A2	
CB_009081	3.94061E-05	1.4624E-06	2.584935	down	10979	FERMT2	
CB_000969	3.93973E-05	1.4615E-06	6.200775	down	54210	TREM1	

CB_024227	3.90654E-05	1.4429E-06	5.498372	down	90865	IL33	
CB_020856	3.88836E-05	1.4344E-06	2.50308	down	57536	KIAA1328	
CB_005714	3.87104E-05	1.4249E-06	2.947312	down	4048	LTA4H	
CB_026092	3.85993E-05	1.4184E-06	3.249143	down	132720	C4orf32	
CB_015988	3.85993E-05	1.4186E-06	15.88527	down	11197	WIF1	
CB_022507	3.83717E-05	1.4058E-06	2.220345	down	80005	DOCK5	
CB_023255	3.83579E-05	1.4037E-06	2.18339	down	83935	TMEM133	
CB_000373	3.83579E-05	1.404E-06	2.596545	down	283501	LOC283501	
CB_013709	3.83579E-05	1.404E-06	4.5356	down	9429	ABCG2	
CB_010218	3.8301E-05	1.3991E-06	5.661848	down	650	BMP2	
CB_011203	3.82483E-05	1.395E-06	2.473724	down	3708	ITPR1	
CB_019838	3.75941E-05	1.3624E-06	2.53035	down	55282	LRRC36	
CB_032062	3.7413E-05	1.354E-06	3.960917	down	389102	YPLR6490	
CB_003278	3.72716E-05	1.3483E-06	5.001003	down			
CB_000634	3.70102E-05	1.3348E-06	2.917996	down	100129846	LOC100129846	
CB_021651	3.67223E-05	1.3226E-06	3.877873	down	64344	HIF3A	
CB_016910	3.65884E-05	1.3166E-06	2.990396	down	8994	LIMD1	
CB_008004	3.64726E-05	1.309E-06	2.717775	down	2268	FGR	
CB_018626	3.6372E-05	1.3048E-06	2.928117	down	51421	AMOTL2	
CB_001079	3.632E-05	1.3012E-06	4.209692	down	10865	ARID5A	
CB_020541	3.62534E-05	1.2977E-06	2.908372	down	10060	ABCC9	
CB_023063	3.61453E-05	1.2904E-06	2.083267	down	23710	GABARAPL1	
CB_000089	3.60679E-05	1.2825E-06	2.422839	down			
CB_024015	3.59428E-05	1.2769E-06	2.46258	down	89846	FGD3	
CB_013061	3.58678E-05	1.2726E-06	6.769502	down	6505	SLC1A1	
CB_006160	3.55413E-05	1.2559E-06	2.138415	down	343413	FCRL6	
CB_008810	3.55156E-05	1.2544E-06	2.358882	down	65065	NBEAL1	
CB_006154	0.000035416	1.2498E-06	4.714768	down	199920	C1orf168	
CB_007000	3.53219E-05	1.2453E-06	2.534104	down	340527	NHSL2	
CB_004722	3.48798E-05	1.2268E-06	4.631704	down			
CB_028916	3.4792E-05	1.2222E-06	31.21256	down	389376	SFTA2	
CB_016380	3.47459E-05	1.22E-06	3.026973	down	23266	LPHN2	
CB_027105	3.46402E-05	1.2147E-06	5.137002	down	285025	CCDC141	
CB_005075	3.43472E-05	1.2033E-06	7.201427	down	4023	LPL	
CB_004978	3.43319E-05	1.2013E-06	3.308608	down	2206	MS4A2	
CB_012803	3.42842E-05	1.1973E-06	5.011815	down	8835	SOCS2	
CB_020122	3.41832E-05	1.1922E-06	3.936313	down	55930	MYO5C	
CB_011984	3.4171E-05	1.1901E-06	4.772559	down	6403	SELP	
CB_024462	3.41411E-05	1.1885E-06	2.935086	down	4638	MYLK	
CB_013218	3.40221E-05	1.1833E-06	2.517974	down	683	BST1	
CB_007671	3.39968E-05	1.1815E-06	5.14454	down	131405	TRIM71	
CB_013550	3.39353E-05	1.1781E-06	3.276084	down	9068	ANGPTL1	
CB_023986	3.38694E-05	1.1742E-06	12.78873	down	57864	SLC46A2	
CB_008613	3.36618E-05	1.1665E-06	2.852851	down	9749	PHACTR2	
CB_010635	3.34398E-05	1.1572E-06	6.688432	down	361	AQP4	
CB_006062	3.30002E-05	1.1368E-06	6.541246	down	285016	FAM150B	
CB_017548	3.29953E-05	1.1361E-06	3.205643	down	89795	NAV3	
CB_028389	3.29839E-05	1.1352E-06	6.633818	down	23767	FLRT3	
CB_021593	3.28389E-05	1.1271E-06	3.333502	down	152007	GLIPR2	
CB_023940	3.27981E-05	1.1251E-06	2.208456	down	9117	SEC22C	
CB_007891	3.26623E-05	1.1195E-06	2.490408	down	51092	SIDT2	
CB_024267	3.26623E-05	1.119E-06	3.133649	down	90273	CEACAM21	
CB_027900	3.26152E-05	1.1158E-06	2.188888	down	255877	BCL6B	
CB_009678	3.26076E-05	1.115E-06	2.461795	down	26137	ZBTB20	
CB_013861	3.24438E-05	1.1078E-06	2.940532	down	3764	KCNJ8	
CB_016370	3.2421E-05	1.1057E-06	2.647866	down	9771	RAPGEF5	

CB_014524	3.2421E-05	1.106E-06	4.343736	down	7113	TMPRSS2	
CB_017029	3.21931E-05	1.0972E-06	3.059704	down	26353	HSPB8	
CB_005177	3.21442E-05	1.0942E-06	2.579429	down	6103	RPGR	
CB_007294	3.21442E-05	1.0951E-06	3.050821	down	10659	CELF2	
CB_010935	3.21427E-05	1.0926E-06	2.836412	down	1983	EIF5	
CB_001886	3.21427E-05	1.093E-06	5.55666	down			
CB_028312	3.2141E-05	1.0919E-06	2.491521	down	378925	RNF148	
CB_025838	3.20575E-05	1.0875E-06	2.76783	down	23336	SYNM	
CB_024362	3.18402E-05	1.0787E-06	2.069946	down	114781	BTBD9	
CB_010012	3.17589E-05	1.0749E-06	5.274399	down	4256	MGP	
CB_002434	3.16863E-05	1.0709E-06	2.135432	down	3841	KPNA5	
CB_009559	3.14951E-05	1.062E-06	2.862508	down	93649	MYOCD	
CB_029366	3.13827E-05	1.0535E-06	4.595689	down	56969	RPL23AP32	
CB_012005	3.13827E-05	1.0535E-06	31.38555	down	6440	SFTPC	
CB_028862	3.13419E-05	1.0503E-06	4.708727	down	221091	LRRN4CL	
CB_006132	3.08667E-05	1.0281E-06	4.194138	down	11217	AKAP2	
CB_022373	3.07365E-05	1.0223E-06	2.328553	down	79845	RNF122	
CB_017592	3.07365E-05	1.0211E-06	2.469187	down	22885	ABLIM3	
CB_019123	3.04803E-05	1.0094E-06	3.663036	down	54749	EPDR1	
CB_000380	3.0297E-05	1.0024E-06	2.379433	down	286437	LOC286437	
CB_002763	3.00719E-05	9.9158E-07	6.08489	down			
CB_010601	2.9539E-05	9.6979E-07	2.830637	down	79026	AHNAK	
CB_010326	2.95114E-05	9.6842E-07	5.607285	down	1396	CRIP1	
CB_028837	2.92494E-05	9.5759E-07	4.872724	down	387758	FIBIN	
CB_017686	2.92149E-05	9.5452E-07	2.15037	down	23051	ZHX3	
CB_013309	2.90081E-05	9.4592E-07	3.93104	down	1960	EGR3	
CB_014650	2.88387E-05	9.3903E-07	3.402475	down	10203	CALCRL	
CB_011272	2.88109E-05	9.3766E-07	7.187226	down	3899	AFF3	
CB_005753	2.87643E-05	9.3569E-07	3.87525	down	5446	PON3	
CB_016520	2.86566E-05	9.3034E-07	3.291359	down	7092	TLL1	
CB_028686	2.86566E-05	9.3037E-07	3.738241	down	79960	PHF17	
CB_018224	2.83957E-05	9.2044E-07	3.696647	down	26112	CCDC69	
CB_017922	2.83853E-05	9.1886E-07	4.579581	down	23327	NEDD4L	
CB_012495	2.83337E-05	9.1674E-07	3.617334	down	8382	NME5	
CB_020269	2.82935E-05	9.1455E-07	2.757336	down	54532	USP53	
CB_007295	2.80765E-05	9.0575E-07	4.358378	down	10659	CELF2	
CB_017416	2.79634E-05	9.0159E-07	2.130096	down	9828	ARHGEF17	
CB_007972	2.78605E-05	8.9658E-07	3.030083	down	8425	LTBP4	
CB_011403	2.76076E-05	8.8694E-07	2.151345	down	4327	MMP19	
CB_006061	2.76076E-05	8.8712E-07	5.754982	down	285016	FAM150B	
CB_000590	2.76019E-05	8.8508E-07	2.196204	down	100130248	LOC100130248	
CB_026286	2.71911E-05	8.6813E-07	4.458059	down	166929	SGMS2	
CB_013549	2.70461E-05	8.6179E-07	5.308662	down	9068	ANGPTL1	
CB_013454	2.69278E-05	8.5759E-07	3.471384	down	5414	4-Sep	
CB_010898	2.69183E-05	8.5687E-07	7.366758	down	1674	DES	
CB_023903	2.67401E-05	8.5035E-07	3.150901	down	84976	DISP1	
CB_006903	2.632E-05	8.3198E-07	3.540919	down	441430	ANKRD20A2	
CB_006588	2.63159E-05	8.3116E-07	4.952629	down	440712	C1orf186	
CB_003551	2.62018E-05	8.2658E-07	3.34277	down			
CB_016379	2.60682E-05	8.2195E-07	2.804362	down	23266	LPHN2	
CB_024663	2.60634E-05	8.2015E-07	2.633298	down	140711	C20orf118	
CB_028176	2.59726E-05	8.14E-07	2.373224	down	113612	CYP2U1	
CB_014495	2.58538E-05	8.0947E-07	4.219657	down	6578	SLCO2A1	
CB_005525	2.58538E-05	8.0904E-07	3.646925	down	240	ALOX5	
CB_017408	2.56877E-05	8.0203E-07	2.008372	down	9812	KIAA0141	
CB_014851	2.56877E-05	8.0263E-07	2.1237	down	7763	ZFAND5	

CB_027466	2.56081E-05	7.9892E-07	5.810962	down	93035	PKHD1L1	
CB_014263	2.55915E-05	7.98E-07	2.806256	down	6414	SEPP1	
CB_023284	2.53563E-05	7.8946E-07	2.704002	down	9708	PCDHGA8	
CB_032168	2.51657E-05	7.8233E-07	2.601847	down	642781	LOC642781	
CB_005409	2.51337E-05	7.8093E-07	4.172857	down	7306	TYRP1	
CB_008710	2.51011E-05	7.7912E-07	2.471475	down	3910	LAMA4	
CB_017685	2.49809E-05	7.7381E-07	2.326052	down	23051	ZHX3	
CB_013569	2.47779E-05	7.6634E-07	2.839133	down	9122	SLC16A4	
CB_023457	2.46297E-05	7.6066E-07	2.070273	down	84263	HSDL2	
CB_012656	2.46297E-05	7.6026E-07	8.085919	down	8630	HSD17B6	
CB_021144	2.45197E-05	7.5444E-07	3.313628	down	8434	RECK	
CB_011012	2.45197E-05	7.5491E-07	9.295558	down	2532	DARC	
CB_027815	2.44892E-05	7.5275E-07	2.920929	down	128344	C1orf88	
CB_030434	2.44865E-05	7.5228E-07	5.049114	down	339524	LOC339524	
CB_028568	2.4339E-05	7.4443E-07	10.33688	down	283383	GPR133	
CB_013108	2.42922E-05	7.4208E-07	2.061293	down	9265	CYTH3	
CB_012349	2.42922E-05	7.4165E-07	3.999048	down	7538	ZFP36	
CB_010250	2.42922E-05	7.4137E-07	4.703632	down	858	CAV2	
CB_012218	2.4183E-05	7.3606E-07	2.482885	down	7082	TJP1	
CB_027337	2.37663E-05	7.2187E-07	3.419777	down	326624	RAB37	
CB_010325	2.36933E-05	7.1928E-07	3.878515	down	1396	CRIP1	
CB_005593	2.36933E-05	7.1908E-07	23.90216	down	1580	CYP4B1	
CB_012781	2.36671E-05	7.1663E-07	2.965216	down	8809	IL18R1	
CB_005492	2.35393E-05	7.1124E-07	30.62061	down	124	ADH1A	
CB_011607	2.34722E-05	7.085E-07	19.8432	down	5225	PGC	
CB_014253	2.33292E-05	7.0293E-07	4.312275	down	5507	PPP1R3C	
CB_006883	2.32843E-05	7.0062E-07	8.112622	down	387104	C6orf174	
CB_021628	2.32204E-05	6.9645E-07	4.92709	down	3386	ICAM4	
CB_031101	2.31541E-05	6.93E-07	3.026787	down	100132526	LOC100132526	
CB_021570	2.31541E-05	6.9273E-07	3.878734	down	64123	ELTD1	
CB_010463	2.28991E-05	6.821E-07	2.698347	down	2295	FOXF2	
CB_027180	2.28478E-05	6.7948E-07	14.80696	down	286133	SCARA5	
CB_023222	2.27856E-05	6.7583E-07	3.139593	down	83891	SNX25	
CB_015618	2.26028E-05	6.6981E-07	8.80286	down	8685	MARCO	
CB_002515	2.24946E-05	6.6506E-07	7.403319	down			
CB_009347	2.22796E-05	6.5507E-07	2.991234	down	51562	MBIP	
CB_013816	2.22266E-05	6.522E-07	2.290057	down	1612	DAPK1	
CB_022298	2.22266E-05	6.522E-07	2.872832	down	474344	GIMAP6	
CB_016899	2.21592E-05	6.4952E-07	2.24164	down	6844	VAMP2	
CB_005165	2.21478E-05	6.4884E-07	2.043082	down	5728	PTEN	
CB_002590	2.2102E-05	6.4631E-07	4.974999	down			
CB_005222	2.19737E-05	6.4176E-07	8.287756	down	7274	TTPA	
CB_002530	2.19136E-05	6.381E-07	6.115908	down			
CB_023584	2.18262E-05	6.3353E-07	2.49928	down	200014	CC2D1B	
CB_006070	2.17494E-05	6.3061E-07	3.241087	down	3983	ABLIM1	
CB_005027	2.17494E-05	6.3056E-07	3.322625	down	6910	TBX5	
CB_006844	2.17206E-05	6.2811E-07	6.126056	down	200010	SLC5A9	
CB_011575	2.16007E-05	6.2185E-07	3.316071	down	5138	PDE2A	
CB_017351	2.14088E-05	6.1447E-07	2.863397	down	9749	PHACTR2	
CB_021310	2.14088E-05	6.1544E-07	5.539055	down	59350	RXFP1	
CB_020650	2.13392E-05	6.1158E-07	2.627128	down	57153	SLC44A2	
CB_004728	2.13392E-05	6.1161E-07	5.015241	down			
CB_007254	2.11114E-05	6.0341E-07	2.424686	down	8829	NRP1	
CB_015682	2.09091E-05	5.9664E-07	9.676144	down	10974	C10orf116	
CB_019972	2.08628E-05	5.9498E-07	2.5029	down	55824	PAG1	
CB_017187	2.08528E-05	5.9405E-07	2.509401	down	26524	LATS2	

CB_018666	2.08528E-05	5.9395E-07	2.863402	down	51170	HSD17B11	
CB_011209	2.08528E-05	5.9344E-07	3.186913	down	3725	JUN	
CB_027251	2.06964E-05	5.8663E-07	3.618103	down	6330	SCN4B	
CB_025459	2.06298E-05	5.8441E-07	2.021475	down	132160	PPM1M	
CB_015659	2.05664E-05	5.8229E-07	2.048558	down	10955	SERINC3	
CB_022523	2.03888E-05	5.7606E-07	2.544933	down	80019	UBTD1	
CB_005545	2.03888E-05	5.763E-07	4.320699	down	776	CACNA1D	
CB_017425	2.03713E-05	5.7516E-07	2.617556	down	9839	ZEB2	
CB_001845	2.03573E-05	5.7411E-07	2.586975	down			
CB_006699	2.0349E-05	5.7291E-07	5.514698	down	414308	MRC1L1	
CB_021145	2.01759E-05	5.6625E-07	3.809746	down	8434	RECK	
CB_017136	2.01759E-05	5.6644E-07	4.872522	down	27306	HPGDS	
CB_003218	2.01637E-05	5.6546E-07	5.810661	down			
CB_009819	1.98926E-05	5.5533E-07	2.006439	down	253725	FAM21C	
CB_006889	1.98898E-05	5.5494E-07	3.040287	down	4916	NTRK3	
CB_017090	1.93276E-05	5.3649E-07	2.395627	down	27143	KIAA1274	
CB_029298	1.93219E-05	5.3603E-07	2.383044	down	246119	TTTY10	
CB_011827	1.93219E-05	5.3561E-07	2.016312	down	5829	PXN	
CB_010436	1.91398E-05	5.2746E-07	4.214088	down	2013	EMP2	
CB_017924	1.90561E-05	5.2413E-07	2.66973	down	23328	SASH1	
CB_019553	1.90305E-05	5.2282E-07	3.684778	down	55081	IFT57	
CB_015236	1.90305E-05	5.2258E-07	3.885817	down	10497	UNC13B	
CB_007483	1.90305E-05	5.2277E-07	4.337666	down	7035	TFPI	
CB_028598	1.89692E-05	5.1963E-07	2.978209	down	7052	TGM2	
CB_010919	1.89137E-05	5.1781E-07	5.172289	down	1906	EDN1	
CB_016820	1.88576E-05	5.1538E-07	8.297302	down	28999	KLF15	
CB_010929	1.85327E-05	5.0317E-07	6.518963	down	1958	EGR1	
CB_011167	1.85154E-05	5.0192E-07	3.967956	down	3572	IL6ST	
CB_018322	1.84211E-05	4.9854E-07	4.186296	down	4008	LMO7	
CB_016075	1.84211E-05	4.9852E-07	5.05457	down	11343	MGLL	
CB_023184	1.84211E-05	4.9907E-07	5.22097	down	83850	ESYT3	
CB_029147	1.84064E-05	4.975E-07	2.400074	down	57188	ADAMTSL3	
CB_005892	1.83455E-05	4.9496E-07	2.023319	down	414328	C9orf103	
CB_009689	1.83455E-05	4.9527E-07	2.172857	down	727910	TLCD2	
CB_014785	1.83455E-05	4.9539E-07	3.255255	down	4337	MOCS1	
CB_021594	1.83204E-05	4.9321E-07	2.252345	down	152007	GLIPR2	
CB_017525	1.83204E-05	4.9343E-07	3.360527	down	9936	CD302	
CB_011110	1.83204E-05	4.9267E-07	6.319729	down	3131	HLF	
CB_015590	1.81536E-05	4.8779E-07	2.584333	down	11214	AKAP13	
CB_016492	1.81146E-05	4.8646E-07	4.999774	down	9723	SEMA3E	
CB_021664	1.77184E-05	4.7301E-07	9.860935	down	64399	HHIP	
CB_025280	1.75556E-05	4.6783E-07	4.659202	down	170689	ADAMTS15	
CB_009281	1.75355E-05	4.6646E-07	3.389528	down	55289	ACOXL	
CB_020092	1.74884E-05	4.6465E-07	3.245721	down	55911	APOB48R	
CB_030288	1.7308E-05	4.5794E-07	5.532245	down	79940	C6orf155	
CB_008803	1.73072E-05	4.5709E-07	3.484258	down	53405	CLIC5	
CB_010093	1.72724E-05	4.5562E-07	2.554108	down	5269	SERPINB6	
CB_011280	1.72367E-05	4.5421E-07	2.401167	down	3913	LAMB2	
CB_020846	1.70737E-05	4.4849E-07	5.125157	down	57528	KCTD16	
CB_007077	1.70659E-05	4.4801E-07	2.327457	down	4921	DDR2	
CB_023398	1.69851E-05	4.4527E-07	3.056779	down	84182	FAM188B	
CB_009390	1.69851E-05	4.4533E-07	3.687756	down	374462	PTPRQ	
CB_029181	1.68792E-05	4.4024E-07	2.323593	down	65059	RAPH1	
CB_014458	1.68657E-05	4.3875E-07	3.431232	down	4781	NFIB	
CB_027304	1.68657E-05	4.3844E-07	3.805428	down	155038	GIMAP8	
CB_015924	1.67471E-05	4.3354E-07	2.293251	down	7402	UTRN	

CB_026278	1.67269E-05	4.3248E-07	4.4426	down	164312	LRRN4	
CB_007301	1.66431E-05	4.3005E-07	3.446562	down	947	CD34	
CB_008927	1.66425E-05	4.2978E-07	2.554898	down	6935	ZEB1	
CB_007572	1.64521E-05	4.2351E-07	3.253547	down	6103	RPGR	
CB_023941	1.64468E-05	4.2263E-07	2.489109	down	9117	SEC22C	
CB_016961	1.64273E-05	4.2187E-07	2.078462	down	23473	CAPN7	
CB_014849	1.63733E-05	4.1971E-07	2.137173	down	7763	ZFAND5	
CB_019598	1.63491E-05	4.1857E-07	4.991955	down	55118	CRTAC1	
CB_024421	1.63012E-05	4.1605E-07	4.405716	down	116159	CYYR1	
CB_021106	1.62703E-05	4.1475E-07	4.420418	down	420	ART4	
CB_010613	1.62627E-05	4.143E-07	3.375159	down	241	ALOX5AP	
CB_020579	1.62513E-05	4.1375E-07	4.111193	down	57088	PLSCR4	
CB_016549	1.62475E-05	4.1339E-07	2.820096	down	29103	DNAJC15	
CB_007335	1.61977E-05	4.1161E-07	2.29206	down	27106	ARRDC2	
CB_016976	1.61294E-05	4.0885E-07	11.43372	down	23584	VSIG2	
CB_014638	1.61292E-05	4.0859E-07	3.306325	down	10186	LHFP	
CB_021236	1.59772E-05	4.0398E-07	4.113178	down	58484	NLRC4	
CB_002548	1.58736E-05	4.0036E-07	4.703182	down	4919	ROR1	
CB_012973	1.58146E-05	3.9862E-07	2.144941	down	1263	PLK3	
CB_007460	1.57255E-05	3.9538E-07	3.179302	down	196740	C10orf72	
CB_027862	1.57255E-05	3.9448E-07	5.723752	down	338557	GPR120	
CB_005529	1.57255E-05	3.9502E-07	8.084674	down	477	ATP1A2	
CB_011860	1.57255E-05	3.95E-07	13.83773	down	5923	RASGRF1	
CB_010922	1.56573E-05	3.9143E-07	2.420379	down	1909	EDNRA	
CB_012204	1.56573E-05	3.914E-07	4.576302	down	7049	TGFBR3	
CB_023399	1.56224E-05	3.8857E-07	3.443508	down	84182	FAM188B	
CB_012183	1.5539E-05	3.8601E-07	2.674638	down	7006	TEC	
CB_009840	1.55303E-05	3.8554E-07	5.410509	down	368	ABCC6	
CB_028329	1.53566E-05	3.8026E-07	3.043864	down	4286	MITF	
CB_016900	1.52034E-05	3.7502E-07	2.200869	down	6844	VAMP2	
CB_021103	1.51958E-05	3.7456E-07	5.229655	down	8470	SORBS2	
CB_005298	1.50643E-05	3.6944E-07	2.885808	down	5175	PECAM1	
CB_020877	1.50425E-05	3.6843E-07	2.986992	down	57561	ARRDC3	
CB_007575	1.48417E-05	3.6186E-07	4.812591	down	10580	SORBS1	
CB_018800	1.48046E-05	3.6025E-07	17.48281	down	1361	CPB2	
CB_014786	1.47121E-05	3.5665E-07	3.139424	down	4337	MOCS1	
CB_018064	1.46902E-05	3.556E-07	5.23356	down	25890	ABI3BP	
CB_029621	1.46624E-05	3.547E-07	3.2361	down	80022	MYO15B	
CB_019064	1.45995E-05	3.5202E-07	2.455466	down	54103	PION	
CB_008985	1.45426E-05	3.5019E-07	2.785656	down	23348	DOCK9	
CB_013284	1.44855E-05	3.4858E-07	2.361994	down	1787	TRDMT1	
CB_026904	1.44823E-05	3.4828E-07	5.045694	down	80274	SCUBE1	
CB_024115	1.44706E-05	3.4748E-07	3.058325	down	9830	TRIM14	
CB_007193	1.44706E-05	3.4754E-07	3.069102	down	2908	NR3C1	
CB_028139	1.44706E-05	3.4732E-07	3.090191	down	23345	SYNE1	
CB_012978	1.44292E-05	3.4561E-07	2.294664	down	1465	CSRP1	
CB_013433	1.4367E-05	3.4344E-07	3.543178	down	4855	NOTCH4	
CB_009339	1.4367E-05	3.4345E-07	8.366487	down	2330	FMO5	
CB_011859	1.42788E-05	3.4067E-07	13.19244	down	5923	RASGRF1	
CB_001871	1.42521E-05	3.398E-07	4.993597	down			
CB_014687	1.42135E-05	3.3821E-07	2.739658	down	10253	SPRY2	
CB_019394	1.41594E-05	3.3625E-07	3.593842	down	54941	RNF125	
CB_012078	1.41129E-05	3.3425E-07	2.615653	down	6642	SNX1	
CB_002665	1.41129E-05	3.3414E-07	3.476193	down			
CB_015333	1.41129E-05	3.339E-07	3.835047	down	10628	TXNIP	
CB_021874	1.40933E-05	3.3311E-07	5.120837	down	10129	FRY	

CB_003472	1.40272E-05	3.3066E-07	3.651388	down	4685	NCAM2	
CB_006462	1.39774E-05	3.2912E-07	14.22651	down	9254	CACNA2D2	
CB_024876	1.38264E-05	3.2483E-07	4.613564	down	7273	TTN	
CB_027696	1.37777E-05	3.2288E-07	3.304106	down	154865	IQUB	
CB_008787	1.37777E-05	3.2303E-07	6.460742	down	22854	NTNG1	
CB_017083	1.36933E-05	3.2019E-07	5.474274	down	27129	HSPB7	
CB_005408	1.36576E-05	3.1913E-07	3.02796	down	7306	TYRP1	
CB_022364	1.35896E-05	3.1706E-07	3.023463	down	79839	CCDC102B	
CB_026432	1.35896E-05	3.1711E-07	4.180219	down	222166	C7orf41	
CB_007677	1.34306E-05	3.1158E-07	2.172249	down	11078	TRIOBP	
CB_022502	1.34306E-05	3.115E-07	2.81926	down	79991	OBFC1	
CB_022918	1.34306E-05	3.1138E-07	3.739912	down	81617	CAB39L	
CB_027465	1.33728E-05	3.0909E-07	2.705868	down	6799	SULT1A2	
CB_023980	1.33117E-05	3.0683E-07	2.548069	down	23499	MACF1	
CB_010027	1.33117E-05	3.0675E-07	3.251973	down	120329	CASP12	
CB_003012	1.32296E-05	3.041E-07	3.057995	down			
CB_027415	1.32236E-05	3.0354E-07	2.837387	down	79742	CXorf36	
CB_012554	1.32096E-05	3.0301E-07	3.341722	down	8490	RGS5	
CB_019863	1.31877E-05	3.023E-07	5.31527	down	55304	SPTLC3	
CB_022947	1.31435E-05	3.0108E-07	4.875724	down	221692	PHACTR1	
CB_006813	1.31011E-05	2.9948E-07	4.014994	down	3082	HGF	
CB_011061	1.30273E-05	2.9685E-07	5.783937	down	2920	CXCL2	
CB_022361	1.30144E-05	2.9627E-07	2.623857	down	79834	PEAK1	
CB_007433	1.30116E-05	2.9591E-07	2.477646	down	57222	ERGIC1	
CB_005424	1.30116E-05	2.9594E-07	3.009189	down	1604	CD55	
CB_025164	1.30116E-05	2.9568E-07	7.5485	down	5468	PPARG	
CB_005371	1.28434E-05	2.8973E-07	10.29833	down	3043	HBB	
CB_008885	1.28334E-05	2.8889E-07	2.132064	down	8837	CFLAR	
CB_003550	1.25359E-05	2.8001E-07	6.777994	down			
CB_023257	1.25123E-05	2.7877E-07	2.177741	down	83938	C10orf11	
CB_012989	1.25123E-05	2.7903E-07	2.53949	down	1831	TSC22D3	
CB_015810	1.25123E-05	2.7908E-07	6.257114	down	9510	ADAMTS1	
CB_020838	1.2446E-05	2.7692E-07	3.653561	down	57520	HECW2	
CB_015811	1.23184E-05	2.7321E-07	4.983809	down	9510	ADAMTS1	
CB_011075	1.23017E-05	2.7263E-07	3.385005	down	2995	GYPC	
CB_022851	1.22994E-05	2.7238E-07	2.449746	down	81553	FAM49A	
CB_013503	1.22894E-05	2.7197E-07	10.63156	down	7433	VIPR1	
CB_023245	1.22789E-05	2.7154E-07	2.095249	down	23731	C9orf5	
CB_028576	1.22448E-05	2.7059E-07	2.409093	down	166336	PRICKLE2	
CB_012607	1.19433E-05	2.6223E-07	6.25497	down	8547	FCN3	
CB_012880	1.19424E-05	2.6202E-07	5.258379	down	8999	CDKL2	
CB_006973	1.19388E-05	2.6175E-07	2.974185	down	401548	SNX30	
CB_011806	1.18808E-05	2.5975E-07	3.937572	down	5793	PTPRG	
CB_025165	1.18808E-05	2.5991E-07	8.039366	down	5468	PPARG	
CB_022191	1.17043E-05	2.542E-07	3.771976	down	79652	TMEM204	
CB_005059	1.15558E-05	2.506E-07	2.440948	down	3753	KCNE1	
CB_018942	1.14007E-05	2.4652E-07	3.471942	down	51562	MBIP	
CB_028673	1.13083E-05	2.4403E-07	3.750467	down	55901	THSD1	
CB_005179	1.13083E-05	2.4416E-07	6.833663	down	6247	RS1	
CB_007355	1.11015E-05	2.3844E-07	5.309283	down	343637	RSPO4	
CB_023809	1.10614E-05	2.3725E-07	3.317177	down	84886	C1orf198	
CB_006078	1.10614E-05	2.372E-07	6.777638	down	3953	LEPR	
CB_024260	1.10399E-05	2.3627E-07	3.044493	down	93663	ARHGAP18	
CB_019016	1.10399E-05	2.3638E-07	19.96954	down	53405	CLIC5	
CB_014666	1.08793E-05	2.311E-07	11.7811	down	10223	GPA33	
CB_026101	1.0876E-05	2.3086E-07	3.100151	down	134265	AFAP1L1	

CB_015953	1.07967E-05	2.2815E-07	2.516064	down	7940	LST1	
CB_014939	1.07384E-05	2.2658E-07	2.110504	down	10392	NOD1	
CB_023882	1.07366E-05	2.2635E-07	6.420013	down	84952	CGNL1	
CB_013082	1.06897E-05	2.2486E-07	2.548678	down	8814	CDKL1	
CB_026993	1.06897E-05	2.2492E-07	6.099917	down	147463	ANKRD29	
CB_022874	1.06004E-05	2.2215E-07	4.552894	down	81575	APOLD1	
CB_026849	1.05896E-05	2.2173E-07	2.853066	down	122773	KLHDC1	
CB_028330	1.05896E-05	2.2165E-07	3.193092	down	4286	MITF	
CB_031821	1.05575E-05	2.2042E-07	3.584052	down			
CB_018709	1.05564E-05	2.2023E-07	2.380482	down	51449	PCYOX1	
CB_025729	1.05287E-05	2.1931E-07	3.198958	down	93010	B3GNT7	
CB_003965	1.04443E-05	2.1624E-07	2.120015	down			
CB_023117	1.04443E-05	2.1637E-07	3.537474	down	83699	SH3BGRL2	
CB_008287	1.04443E-05	2.1635E-07	4.252529	down	375033	PEAR1	
CB_010462	1.04397E-05	2.1564E-07	4.395596	down	2294	FOXF1	
CB_014850	1.0414E-05	2.1487E-07	2.025845	down	7763	ZFAND5	
CB_005673	1.0414E-05	2.1466E-07	3.750303	down	2977	GUCY1A2	
CB_012081	1.0414E-05	2.1459E-07	4.365323	down	6649	SOD3	
CB_013534	1.0414E-05	2.1495E-07	11.58552	down	8436	SDPR	
CB_028921	1.02995E-05	2.1023E-07	4.664141	down	2494	NR5A2	
CB_010543	1.01662E-05	2.0652E-07	3.11515	down	3316	HSPB2	
CB_018879	9.96818E-06	2.0145E-07	3.850834	down	51267	CLEC1A	
CB_002758	9.92289E-06	2.0015E-07	2.951148	down			
CB_017941	9.56527E-06	1.9099E-07	2.92553	down	23348	DOCK9	
CB_008149	9.54112E-06	1.9012E-07	4.676346	down	641700	ECSCR	
CB_017317	9.53308E-06	1.8981E-07	3.773284	down	9721	GPRIN2	
CB_014218	9.51063E-06	1.8891E-07	4.637774	down	4008	LMO7	
CB_013054	9.46421E-06	1.8769E-07	4.259906	down	6236	RRAD	
CB_013448	9.42929E-06	1.8625E-07	2.970342	down	5269	SERPINB6	
CB_011587	9.42629E-06	1.8604E-07	7.75562	down	5166	PDK4	
CB_015921	9.39343E-06	1.8516E-07	2.346201	down	7402	UTRN	
CB_012659	9.39343E-06	1.8524E-07	3.154443	down	8633	UNC5C	
CB_005151	9.39343E-06	1.8508E-07	3.518816	down	5376	PMP22	
CB_027934	9.39343E-06	1.8515E-07	3.604955	down	126668	TDRD10	
CB_010968	9.39343E-06	1.8518E-07	7.402225	down	2205	FCER1A	
CB_015922	9.28488E-06	1.8207E-07	2.891296	down	7402	UTRN	
CB_031820	9.27678E-06	1.8162E-07	2.979178	down			
CB_009298	9.24804E-06	1.8091E-07	2.167993	down	53373	TPCN1	
CB_021339	9.20318E-06	1.7989E-07	2.466955	down	10742	RAI2	
CB_016086	9.16257E-06	1.7895E-07	2.968539	down	3373	HYAL1	
CB_021602	9.15109E-06	1.7814E-07	4.47529	down	64174	DPEP2	
CB_007313	9.11169E-06	1.7695E-07	2.677473	down	7106	TSPAN4	
CB_017889	9.10282E-06	1.7644E-07	2.432584	down	23294	ANKS1A	
CB_028566	9.10282E-06	1.7648E-07	7.623678	down	283383	GPR133	
CB_027765	9.08438E-06	1.7568E-07	3.212952	down	3672	ITGA1	
CB_015963	8.9883E-06	1.7341E-07	8.095231	down	10351	ABCA8	
CB_016095	8.81358E-06	1.692E-07	2.119937	down	1727	CYB5R3	
CB_014122	8.66551E-06	1.6567E-07	5.018823	down	2627	GATA6	
CB_014333	8.66473E-06	1.6551E-07	2.50893	down	10023	FRAT1	
CB_009001	8.61834E-06	1.6436E-07	2.166193	down	6709	SPTAN1	
CB_013328	8.61243E-06	1.6397E-07	3.986402	down	2078	ERG	
CB_024774	8.5552E-06	1.622E-07	6.524446	down	142683	ITLN2	
CB_005891	8.48143E-06	1.6027E-07	7.216861	down	948	CD36	
CB_010474	8.46695E-06	1.5986E-07	8.678073	down	2330	FMO5	
CB_012126	8.42774E-06	1.5872E-07	11.43109	down	6769	STAC	
CB_022550	8.39071E-06	1.5785E-07	2.410118	down	54507	ADAMTSL4	

CB_030716	8.39071E-06	1.5789E-07	3.572752	down	284232	LOC284232	
CB_013900	8.34279E-06	1.5619E-07	5.795983	down	5137	PDE1C	
CB_010396	8.32468E-06	1.5572E-07	3.908981	down	1808	DPYSL2	
CB_010031	8.27276E-06	1.541E-07	6.489475	down	5137	PDE1C	
CB_018098	8.25982E-06	1.5334E-07	2.727211	down	25927	CNRIP1	
CB_016176	8.21697E-06	1.5191E-07	2.902828	down	23499	MACF1	
CB_022961	8.21697E-06	1.5201E-07	4.157251	down	81848	SPRY4	
CB_007971	8.20495E-06	1.514E-07	4.962459	down	8425	LTBP4	
CB_015964	8.11857E-06	1.4916E-07	17.00125	down	10351	ABCA8	
CB_021248	8.08851E-06	1.4848E-07	4.499306	down	58494	JAM2	
CB_024069	8.04889E-06	1.4763E-07	2.66427	down	8692	HYAL2	
CB_018065	8.04135E-06	1.4726E-07	6.228472	down	25890	ABI3BP	
CB_019365	8.01083E-06	1.4655E-07	3.968054	down	54922	RASIP1	
CB_008876	7.99591E-06	1.4615E-07	2.767216	down	141	ADPRH	
CB_022098	7.9614E-06	1.4539E-07	4.005769	down	57194	ATP10A	
CB_005745	7.93957E-06	1.4487E-07	13.46234	down	5319	PLA2G1B	
CB_009617	7.93714E-06	1.4445E-07	4.639401	down	55679	LIMS2	
CB_026968	7.91655E-06	1.439E-07	2.173748	down	123879	DCUN1D3	
CB_021472	7.87117E-06	1.42E-07	6.422402	down	63876	PKNOX2	
CB_002098	7.87117E-06	1.4235E-07	10.23195	down			
CB_005369	7.87117E-06	1.4248E-07	10.83506	down	3040	HBA2	
CB_028873	7.84118E-06	1.4121E-07	4.552762	down	400120	C13orf36	
CB_018277	7.83501E-06	1.4085E-07	4.426269	down	27145	FILIP1	
CB_026449	7.65838E-06	1.3695E-07	6.569825	down	9104	RGN	
CB_017787	7.65688E-06	1.3679E-07	2.043606	down	23164	MPRIP	
CB_001298	7.65688E-06	1.368E-07	3.313521	down	3603	IL16	
CB_015469	7.64602E-06	1.3624E-07	2.326516	down	10749	KIF1C	
CB_009000	7.64602E-06	1.3621E-07	4.559723	down	81575	APOLD1	
CB_010586	7.64602E-06	1.3608E-07	12.97822	down	33	ACADL	
CB_024801	7.63602E-06	1.357E-07	3.684055	down	122953	JDP2	
CB_011369	7.61727E-06	1.352E-07	2.789485	down	4193	MDM2	
CB_002503	7.59306E-06	1.3402E-07	3.41021	down			
CB_024596	7.59306E-06	1.3396E-07	3.416256	down	976	CD97	
CB_026740	7.57799E-06	1.3335E-07	3.764988	down	10235	RASGRP2	
CB_021592	7.51958E-06	1.3208E-07	2.772834	down	64147	KIF9	
CB_012555	7.51958E-06	1.32E-07	4.208389	down	8490	RGS5	
CB_029945	7.45823E-06	1.3077E-07	4.969448	down	158376	LOC158376	
CB_005639	7.45823E-06	1.3067E-07	6.486393	down	2891	GRIA2	
CB_010600	7.45656E-06	1.305E-07	2.728895	down	79026	AHNAK	
CB_004394	7.42072E-06	1.2964E-07	4.459714	down			
CB_017899	7.40597E-06	1.2926E-07	2.523935	down	23303	KIF13B	
CB_011303	7.40597E-06	1.2923E-07	9.431776	down	3977	LIFR	
CB_015559	7.30259E-06	1.2711E-07	2.918178	down	10908	PNPLA6	
CB_012651	7.29303E-06	1.268E-07	3.735945	down	8622	PDE8B	
CB_016217	7.29303E-06	1.2683E-07	4.103554	down	25802	LMOD1	
CB_005368	7.29303E-06	1.2661E-07	11.29952	down	3040	HBA2	
CB_016216	7.2425E-06	1.2526E-07	5.335971	down	25802	LMOD1	
CB_008545	7.17811E-06	1.2381E-07	14.07671	down	219995	MS4A15	
CB_020834	7.16237E-06	1.2319E-07	3.771334	down	57514	ARHGAP31	
CB_023143	7.14772E-06	1.226E-07	5.499646	down	185	AGTR1	
CB_018657	7.14772E-06	1.2242E-07	9.007642	down	9173	IL1RL1	
CB_028328	7.12796E-06	1.2168E-07	2.919847	down	4286	MITF	
CB_027971	7.12796E-06	1.217E-07	31.22953	down	200504	GKN2	
CB_018748	7.09871E-06	1.2097E-07	3.461964	down	51196	PLCE1	
CB_005322	7.05074E-06	1.2004E-07	3.139169	down	203	AK1	
CB_020966	6.98751E-06	1.1841E-07	2.635565	down	57658	CALCOCO1	

CB_009666	6.97589E-06	1.18E-07	2.300729	down	57091	CASS4	
CB_012734	6.95244E-06	1.1739E-07	3.306848	down	8742	TNFSF12	
CB_022220	6.95244E-06	1.1749E-07	8.475355	down	79689	STEAP4	
CB_004899	6.94299E-06	1.1689E-07	2.603531	down	710	SERPING1	
CB_024198	6.89541E-06	1.156E-07	2.640384	down	85450	ITPRIP	
CB_003719	6.89541E-06	1.1545E-07	3.155075	down			
CB_014332	6.88613E-06	1.1495E-07	2.334364	down	10023	FRAT1	
CB_027842	6.79923E-06	1.1285E-07	2.663441	down	160622	GRASP	
CB_022159	6.72152E-06	1.1114E-07	9.181296	down	79625	C4orf31	
CB_001769	6.56916E-06	1.081E-07	13.09883	down			
CB_022604	6.56593E-06	1.0783E-07	5.353863	down	80177	MYCT1	
CB_014447	6.44377E-06	1.0522E-07	4.357892	down	4091	SMAD6	
CB_028144	6.37575E-06	1.04E-07	4.3512	down	4739	NEDD9	
CB_028140	6.34234E-06	1.0284E-07	3.916693	down	23345	SYNE1	
CB_013072	6.29309E-06	1.0156E-07	2.514993	down	7439	BEST1	
CB_021875	6.25003E-06	1.0061E-07	6.862459	down	10129	FRY	
CB_014158	6.22601E-06	9.9883E-08	2.210428	down	2843	GPR20	
CB_020255	6.20602E-06	9.9464E-08	3.950143	down	54511	HMGCLL1	
CB_018900	6.17585E-06	9.8837E-08	3.228977	down	4815	NINJ2	
CB_014912	6.17585E-06	9.8823E-08	4.216779	down	10326	SIRPB1	
CB_008677	6.16152E-06	9.836E-08	5.347271	down	131873	COL6A6	
CB_005928	6.04679E-06	9.5858E-08	5.593634	down	414332	LCN10	
CB_022691	6.04531E-06	9.5738E-08	7.669291	down	80323	CCDC68	
CB_010558	6.02999E-06	9.5304E-08	3.737615	down	3491	CYR61	
CB_005633	6.01933E-06	9.5041E-08	2.512212	down	2621	GAS6	
CB_001181	6.00858E-06	9.4724E-08	2.41024	down			
CB_007199	6.00299E-06	9.4402E-08	14.37044	down	145781	GCOM1	
CB_010737	5.98129E-06	9.3871E-08	5.742099	down	857	CAV1	
CB_014649	5.90275E-06	9.2177E-08	4.224497	down	10203	CALCRL	
CB_016002	5.88363E-06	9.1779E-08	2.653148	down	11228	RASSF8	
CB_020295	5.87428E-06	9.154E-08	2.542489	down	54558	SPATA6	
CB_005510	5.87428E-06	9.1509E-08	9.594139	down	153	ADRB1	
CB_013892	5.77156E-06	8.9573E-08	5.902089	down	4919	ROR1	
CB_010887	5.75224E-06	8.9091E-08	3.886971	down	1528	CYB5A	
CB_017831	5.71513E-06	8.8335E-08	4.06608	down	23221	RHOBTB2	
CB_010010	5.68852E-06	8.7833E-08	3.530428	down	1528	CYB5A	
CB_020835	5.66005E-06	8.7304E-08	2.368364	down	57515	SERINC1	
CB_011060	5.64316E-06	8.6865E-08	9.717057	down	2920	CXCL2	
CB_002587	5.54494E-06	8.4066E-08	3.080322	down	311	ANXA11	
CB_028431	5.54494E-06	8.4283E-08	3.390743	down	283298	OLFML1	
CB_008512	5.54494E-06	8.3992E-08	7.792451	down	221395	GPR116	
CB_022720	5.54494E-06	8.4298E-08	10.6911	down	80704	SLC19A3	
CB_017424	5.53967E-06	8.3603E-08	2.617095	down	9839	ZEB2	
CB_009619	5.53472E-06	8.3441E-08	4.715469	down	202151	RANBP3L	
CB_010454	5.49589E-06	8.2768E-08	26.13088	down	2167	FABP4	
CB_012646	5.42078E-06	8.1207E-08	2.513053	down	8613	PPAP2B	
CB_010145	5.38247E-06	8.0548E-08	6.580183	down	3953	LEPR	
CB_013840	5.2355E-06	7.8017E-08	6.27899	down	2662	GDF10	
CB_013664	5.23447E-06	7.7902E-08	2.083701	down	9344	TAOK2	
CB_021922	5.12578E-06	7.5569E-08	6.066366	down	79098	C1orf116	
CB_010949	5.07754E-06	7.4616E-08	2.003697	down	2060	EPS15	
CB_018579	5.0595E-06	7.4191E-08	4.430299	down	51673	TPPP3	
CB_019203	5.05852E-06	7.4047E-08	3.790342	down	54796	BNC2	
CB_011477	5.05318E-06	7.3858E-08	2.468942	down	4784	NFIX	
CB_011597	5.05123E-06	7.3749E-08	2.69864	down	5199	CFP	
CB_010316	5.0215E-06	7.3236E-08	3.472964	down	1316	KLF6	

CB_025336	5.01034E-06	7.2835E-08	3.552766	down	121512	FGD4	
CB_022745	4.96933E-06	7.2002E-08	13.72014	down	80760	ITIH5	
CB_020922	4.95643E-06	7.1501E-08	4.07557	down	57608	KIAA1462	
CB_020352	4.94884E-06	7.1313E-08	3.067832	down	56261	GPCPD1	
CB_027498	4.91815E-06	7.0325E-08	2.856654	down	90627	STARD13	
CB_020294	4.90036E-06	6.9993E-08	2.903578	down	54558	SPATA6	
CB_025335	4.89383E-06	6.9822E-08	2.950563	down	121512	FGD4	
CB_016079	4.88678E-06	6.9644E-08	2.010936	down	11346	SYNPO	
CB_004592	4.80689E-06	6.8244E-08	14.89136	down			
CB_005741	4.78293E-06	6.7633E-08	4.132828	down	5241	PGR	
CB_024133	4.75444E-06	6.6929E-08	2.327858	down	94274	PPP1R14A	
CB_005372	4.75444E-06	6.6835E-08	9.039619	down	3045	HBD	
CB_019464	4.71043E-06	6.5712E-08	2.434712	down	54996	MOSC2	
CB_017404	4.65194E-06	6.4628E-08	6.229549	down	9806	SPOCK2	
CB_018283	4.63854E-06	6.4341E-08	5.025279	down	27151	CPAMD8	
CB_009639	4.62769E-06	6.4118E-08	2.385697	down	9674	KIAA0040	
CB_007218	4.61161E-06	6.3749E-08	3.446955	down	91663	MYADM	
CB_028015	4.60216E-06	6.3545E-08	2.315884	down	338699	ANKRD42	
CB_019283	4.54009E-06	6.2256E-08	2.038106	down	54861	SNRK	
CB_004917	4.54009E-06	6.2197E-08	2.133502	down	1130	LYST	
CB_013083	4.50023E-06	6.1567E-08	4.339145	down	8814	CDKL1	
CB_018749	4.49299E-06	6.1365E-08	3.294994	down	51196	PLCE1	
CB_013668	4.48987E-06	6.1212E-08	4.975304	down	9353	SLIT2	
CB_015612	4.48311E-06	6.098E-08	3.987357	down	7832	BTG2	
CB_010783	4.36958E-06	5.8463E-08	7.685852	down	1036	CDO1	
CB_016804	4.3325E-06	5.7692E-08	5.711881	down	28984	C13orf15	
CB_011055	4.3325E-06	5.7684E-08	5.757623	down	2878	GPX3	
CB_015549	4.32857E-06	5.744E-08	10.58594	down	10894	LYVE1	
CB_006219	4.32857E-06	5.7503E-08	12.71675	down	338596	ST8SIA6	
CB_029146	4.30338E-06	5.7032E-08	7.83681	down	57188	ADAMTSL3	
CB_005370	4.27878E-06	5.6638E-08	13.12	down	3040	HBA2	
CB_021166	4.24472E-06	5.5985E-08	3.598068	down	6196	RPS6KA2	
CB_013006	4.23288E-06	5.5695E-08	2.842146	down	2217	FCGRT	
CB_008827	4.20195E-06	5.5221E-08	2.763297	down	115	ADCY9	
CB_001340	4.16962E-06	5.4664E-08	6.328799	down	439950	LOC439950	
CB_011043	4.16747E-06	5.457E-08	2.345459	down	2776	GNAQ	
CB_004898	4.09328E-06	5.3048E-08	2.341179	down	710	SERPING1	
CB_002682	4.03958E-06	5.2063E-08	10.21885	down			
CB_025655	3.99919E-06	5.1352E-08	2.097388	down	127700	OSCP1	
CB_010756	3.99588E-06	5.1246E-08	4.324341	down	947	CD34	
CB_013283	3.97619E-06	5.0931E-08	2.063966	down	1787	TRDMT1	
CB_006853	3.93443E-06	5.0271E-08	4.084489	down	84460	ZMAT1	
CB_025654	3.92965E-06	5.0148E-08	2.091541	down	127700	OSCP1	
CB_008782	3.92862E-06	5.0072E-08	4.335761	down	104	ADARB1	
CB_007573	3.87745E-06	4.9122E-08	4.086913	down	10580	SORBS1	
CB_026531	3.87745E-06	4.9065E-08	4.322291	down	120425	AMICA1	
CB_013720	3.81673E-06	4.8041E-08	2.911771	down	9459	ARHGEF6	
CB_020319	3.80925E-06	4.7887E-08	3.834491	down	3202	HOXA5	
CB_018924	3.69651E-06	4.6176E-08	4.68411	down	51285	RASL12	
CB_000142	3.65369E-06	4.5236E-08	2.859658	down	64150	DIO3OS	
CB_023717	3.61806E-06	4.468E-08	2.508556	down	84701	COX4I2	
CB_017591	3.57351E-06	4.4017E-08	6.035264	down	22885	ABLIM3	
CB_025791	3.51642E-06	4.3091E-08	7.200689	down	219790	RTKN2	
CB_004530	3.5009E-06	4.2734E-08	4.787441	down			
CB_028567	3.49289E-06	4.2581E-08	3.035418	down	283383	GPR133	
CB_012691	3.48325E-06	4.2408E-08	4.696623	down	8676	STX11	

CB_012876	3.47178E-06	4.2213E-08	9.815984	down	8991	SELENBP1	
CB_019017	3.46919E-06	4.2017E-08	23.27494	down	53405	CLIC5	
CB_004837	3.465E-06	4.1856E-08	5.811726	down	2	A2M	
CB_021668	3.45746E-06	4.1381E-08	2.375297	down	64411	ARAP3	
CB_021366	3.44838E-06	4.1163E-08	5.681288	down	60495	HPSE2	
CB_014522	3.43987E-06	4.0953E-08	5.79221	down	7025	NR2F1	
CB_008347	3.43431E-06	4.0832E-08	2.207433	down	57545	CC2D2A	
CB_004189	3.41014E-06	4.0491E-08	4.623933	down			
CB_005176	3.39563E-06	4.0163E-08	2.602526	down	6103	RPGR	
CB_017794	3.39563E-06	4.0211E-08	3.845428	down	23171	GPD1L	
CB_010443	3.38283E-06	3.9898E-08	4.730678	down	2034	EPAS1	
CB_006620	3.38047E-06	3.9817E-08	2.979873	down	10217	CTDSPL	
CB_021227	3.35578E-06	3.9239E-08	2.274564	down	58190	CTDSP1	
CB_018631	3.35578E-06	3.9313E-08	3.742226	down	389136	VGLL3	
CB_022657	3.3535E-06	3.9115E-08	5.98349	down	80243	PREX2	
CB_026780	3.34501E-06	3.8892E-08	5.92724	down	8854	ALDH1A2	
CB_027693	3.34501E-06	3.8922E-08	7.52458	down	285313	IGSF10	
CB_003280	3.31282E-06	3.839E-08	7.900637	down			
CB_027319	3.2975E-06	3.8108E-08	3.357248	down	23492	CBX7	
CB_011032	3.27828E-06	3.7568E-08	2.34828	down	2701	GJA4	
CB_010923	3.27828E-06	3.7644E-08	2.962158	down	1909	EDNRA	
CB_013291	3.27828E-06	3.7678E-08	5.528466	down	1843	DUSP1	
CB_015583	3.27828E-06	3.7377E-08	6.611006	down	2354	FOSB	
CB_024729	3.2693E-06	3.7056E-08	5.456585	down	117532	TMC2	
CB_015132	3.26904E-06	3.695E-08	4.805563	down	7035	TFPI	
CB_021104	3.23069E-06	3.6465E-08	3.038422	down	8470	SORBS2	
CB_022917	3.21022E-06	3.6134E-08	6.135456	down	81617	CAB39L	
CB_009536	3.15759E-06	3.544E-08	6.141143	down	284	ANGPT1	
CB_030681	3.15541E-06	3.5365E-08	3.272238	down	104	ADARB1	
CB_022487	3.12148E-06	3.4787E-08	3.84815	down	79974	C7orf58	
CB_005717	3.11209E-06	3.4503E-08	6.383288	down	4129	MAOB	
CB_022946	3.08462E-06	3.4034E-08	5.319486	down	221692	PHACTR1	
CB_011296	3.04408E-06	3.3394E-08	4.110697	down	3953	LEPR	
CB_004849	3.04408E-06	3.3362E-08	5.481324	down	154	ADRB2	
CB_018419	3.02726E-06	3.2913E-08	2.089224	down	815	CAMK2A	
CB_022074	3.02726E-06	3.3001E-08	2.935874	down	11000	SLC27A3	
CB_010904	3.02504E-06	3.2705E-08	3.4888	down	1805	DPT	
CB_009620	3.02504E-06	3.2692E-08	4.862503	down	221	ALDH3B1	
CB_027881	3.02504E-06	3.2696E-08	6.088508	down	342035	GLDN	
CB_021247	3.00807E-06	3.2379E-08	4.52674	down	58494	JAM2	
CB_008476	2.98494E-06	3.2035E-08	3.356176	down	6414	SEPP1	
CB_002360	2.98494E-06	3.203E-08	6.509631	down	79836	LONRF3	
CB_003393	2.89036E-06	3.0608E-08	7.378904	down			
CB_020581	2.85853E-06	3.0124E-08	4.608927	down	57091	CASS4	
CB_012939	2.83522E-06	2.974E-08	4.918778	down	408	ARRB1	
CB_009079	2.82834E-06	2.9592E-08	3.79961	down	10979	FERMT2	
CB_015740	2.80189E-06	2.9271E-08	2.147875	down	4649	MYO9A	
CB_022112	2.75413E-06	2.8597E-08	2.49897	down	79443	FYCO1	
CB_021149	2.75413E-06	2.8542E-08	2.732379	down	1408	CRY2	
CB_003304	2.73615E-06	2.8174E-08	3.060744	down			
CB_027773	2.73376E-06	2.8083E-08	2.619693	down	5295	PIK3R1	
CB_018708	2.66954E-06	2.7338E-08	2.157826	down	51449	PCYOX1	
CB_028688	2.66954E-06	2.7331E-08	2.70507	down	10252	SPRY1	
CB_012106	2.63395E-06	2.6681E-08	2.343051	down	6717	SRI	
CB_027541	2.63109E-06	2.6611E-08	2.188878	down	283349	RASSF3	
CB_018266	2.59823E-06	2.6196E-08	4.982506	down	4616	GADD45B	

CB_006743	2.58723E-06	2.5962E-08	6.695981	down	126669	SHE	
CB_012104	2.55856E-06	2.5553E-08	6.039861	down	6711	SPTBN1	
CB_005215	2.55331E-06	2.5419E-08	4.941078	down	7078	TIMP3	
CB_020695	2.5368E-06	2.5215E-08	11.367	down	9457	FHL5	
CB_007892	2.53016E-06	2.5109E-08	2.823395	down	51092	SIDT2	
CB_005350	2.51263E-06	2.4815E-08	4.791009	down	2006	ELN	
CB_028314	2.50681E-06	2.4639E-08	6.528098	down	358	AQP1	
CB_013022	2.46182E-06	2.4033E-08	6.056549	down	2791	GNG11	
CB_014777	2.43955E-06	2.3745E-08	2.468373	down	4299	AFF1	
CB_004900	2.43056E-06	2.3605E-08	2.202952	down	710	SERPING1	
CB_017786	2.33441E-06	2.2155E-08	2.097947	down	23164	MPRIP	
CB_028835	2.31158E-06	2.1802E-08	2.223295	down	65059	RAPH1	
CB_008203	2.31158E-06	2.1598E-08	3.583483	down	9625	AATK	
CB_014927	2.31158E-06	2.1877E-08	4.137645	down	10370	CITED2	
CB_027531	2.31158E-06	2.1841E-08	4.491983	down	23037	PDZD2	
CB_004845	2.31158E-06	2.1859E-08	4.900964	down	94	ACVRL1	
CB_008204	2.31158E-06	2.1716E-08	9.693422	down	9625	AATK	
CB_025563	2.31158E-06	2.154E-08	15.443	down	157310	PEBP4	
CB_028226	2.28968E-06	2.1234E-08	2.220871	down	158135	TTLL11	
CB_004485	2.24295E-06	2.0694E-08	3.318897	down	58494	JAM2	
CB_025343	2.24003E-06	2.0632E-08	2.190454	down	196883	ADCY4	
CB_004879	2.22158E-06	2.0196E-08	9.173018	down	443	ASPA	
CB_023538	2.18557E-06	1.9749E-08	4.579329	down	10826	C5orf4	
CB_025248	2.18279E-06	1.9689E-08	3.065068	down	90952	ESAM	
CB_007274	2.18055E-06	1.9565E-08	4.96558	down	7048	TGFBR2	
CB_009587	2.1773E-06	1.9502E-08	5.101269	down	316	AOX1	
CB_022486	2.13683E-06	1.9071E-08	4.158324	down	79974	C7orf58	
CB_014694	2.13342E-06	1.8973E-08	5.182317	down	10266	RAMP2	
CB_019495	2.09025E-06	1.849E-08	4.490022	down	55022	PID1	
CB_017501	2.08881E-06	1.8424E-08	5.070392	down	9912	ARHGAP44	
CB_006220	2.0888E-06	1.8345E-08	13.86547	down	338596	ST8SIA6	
CB_016118	2.0838E-06	1.81E-08	9.689855	down	22915	MMRN1	
CB_011235	2.07215E-06	1.7903E-08	3.373568	down	3791	KDR	
CB_027887	2.03364E-06	1.7506E-08	2.548747	down	11142	PKIG	
CB_028824	1.98365E-06	1.6981E-08	3.264174	down	966	CD59	
CB_009088	1.96505E-06	1.6772E-08	15.84723	down	64116	SLC39A8	
CB_009616	1.96292E-06	1.671E-08	4.244596	down	55679	LIMS2	
CB_017900	1.95062E-06	1.654E-08	2.074567	down	23303	KIF13B	
CB_022656	1.95062E-06	1.6571E-08	8.522244	down	80243	PREX2	
CB_027076	1.94445E-06	1.6368E-08	7.574616	down	146177	VWA3A	
CB_018632	1.86722E-06	1.557E-08	5.827085	down	389136	VGLL3	
CB_005493	1.83696E-06	1.5143E-08	20.36599	down	125	ADH1B	
CB_026214	1.82038E-06	1.4915E-08	2.231548	down	152273	FGD5	
CB_007481	1.82038E-06	1.4919E-08	2.50533	down	79148	MMP28	
CB_026668	1.795E-06	1.4569E-08	8.08963	down	93649	MYOCD	
CB_012805	1.79017E-06	1.4473E-08	3.208596	down	8837	CFLAR	
CB_024707	1.77318E-06	1.4308E-08	6.80522	down	6910	TBX5	
CB_003631	1.76631E-06	1.4173E-08	19.70096	down	6332	SCN7A	
CB_004778	1.76622E-06	1.4134E-08	3.760667	down			
CB_022353	1.76622E-06	1.414E-08	4.52785	down	79825	CCDC48	
CB_021640	1.72154E-06	1.3509E-08	5.923054	down	64321	SOX17	
CB_028208	1.71454E-06	1.3427E-08	2.652585	down	8848	TSC22D1	
CB_026741	1.66776E-06	1.2796E-08	2.356136	down	10235	RASGRP2	
CB_024132	1.66776E-06	1.2772E-08	5.923923	down	94274	PPP1R14A	
CB_007198	1.65383E-06	1.2621E-08	8.099756	down	145781	GCOM1	
CB_027886	1.64574E-06	1.2471E-08	2.627798	down	11142	PKIG	

CB_012161	1.64574E-06	1.2447E-08	6.112341	down	6886	TAL1	
CB_011951	1.64574E-06	1.242E-08	13.00151	down	6332	SCN7A	
CB_015625	1.63793E-06	1.2279E-08	2.199458	down	9444	QKI	
CB_025392	1.63793E-06	1.2333E-08	4.037217	down	91624	NEXN	
CB_015235	1.62586E-06	1.2114E-08	2.333169	down	10497	UNC13B	
CB_019295	1.62586E-06	1.2109E-08	5.23592	down	54873	PALMD	
CB_017502	1.57435E-06	1.1655E-08	4.259134	down	9912	ARHGAP44	
CB_005939	1.52717E-06	1.1257E-08	9.460617	down	80760	ITIH5	
CB_005299	1.50766E-06	1.1042E-08	3.624463	down	5175	PECAM1	
CB_022389	1.4887E-06	1.0856E-08	2.858347	down	79864	C11orf63	
CB_017325	1.4887E-06	1.0804E-08	2.886072	down	9728	SECISBP2L	
CB_010346	1.47885E-06	1.0667E-08	3.260622	down	1519	CTSO	
CB_016377	1.47823E-06	1.0616E-08	3.749295	down	23266	LPHN2	
CB_012045	1.45227E-06	1.036E-08	5.640926	down	6586	SLIT3	
CB_027909	1.44963E-06	1.0318E-08	10.28654	down	57716	PRX	
CB_028190	1.39438E-06	9.8027E-09	2.419627	down	221749	C6orf145	
CB_023223	1.39438E-06	9.7874E-09	3.472601	down	83891	SNX25	
CB_011866	1.39438E-06	9.8151E-09	3.890243	down	5939	RBMS2	
CB_017567	1.39438E-06	9.7624E-09	8.598358	down	22854	NTNG1	
CB_023936	1.38334E-06	9.5256E-09	11.24598	down	6358	CCL14	
CB_017644	1.38334E-06	9.5394E-09	11.62119	down	22998	LIMCH1	
CB_005433	1.37615E-06	9.4462E-09	7.571316	down	730	C7	
CB_015116	1.36957E-06	9.2973E-09	3.080484	down	6237	RRAS	
CB_027771	1.36957E-06	9.3142E-09	3.909781	down	83849	SYT15	
CB_027038	1.36957E-06	9.2659E-09	4.836863	down	219621	C10orf107	
CB_013546	1.35902E-06	9.1562E-09	2.99165	down	9022	CLIC3	
CB_021374	1.35902E-06	9.1424E-09	4.357505	down	1073	CFL2	
CB_008513	1.31786E-06	8.7118E-09	6.163442	down	221395	GPR116	
CB_008233	1.3079E-06	8.6045E-09	3.348086	down	85358	SHANK3	
CB_000181	1.2987E-06	8.5233E-09	3.690266	down			
CB_031310	1.2827E-06	8.3823E-09	6.303009	down	100129101	LOC100129101	
CB_020472	1.27632E-06	8.2348E-09	3.0076	down	56929	FEM1C	
CB_023077	1.27632E-06	8.2148E-09	3.441241	down	83547	RILP	
CB_014807	1.27397E-06	8.1391E-09	3.623543	down	4628	MYH10	
CB_020923	1.27397E-06	8.1131E-09	3.967058	down	57608	KIAA1462	
CB_009136	1.26809E-06	8.041E-09	25.18664	down	177	AGER	
CB_007197	1.26291E-06	7.9882E-09	9.041981	down	145781	GCOM1	
CB_017127	1.25888E-06	7.9418E-09	3.908993	down	27295	PDLIM3	
CB_016994	1.25888E-06	7.9427E-09	4.657599	down	23645	PPP1R15A	
CB_005411	1.25708E-06	7.8915E-09	5.707852	down	7450	VWF	
CB_014117	1.25123E-06	7.7358E-09	8.111445	down	2353	FOS	
CB_005685	1.22536E-06	7.4905E-09	2.20707	down	3355	HTR1F	
CB_018999	1.22536E-06	7.4981E-09	8.808499	down	2348	FOLR1	
CB_020677	1.22219E-06	7.4216E-09	5.637226	down	57211	GPR126	
CB_026631	1.22219E-06	7.4322E-09	5.732232	down	79987	SVEP1	
CB_021877	1.22219E-06	7.44E-09	6.361274	down	10129	FRY	
CB_006893	1.2071E-06	7.1496E-09	2.088144	down	64400	AKTIP	
CB_020323	1.2071E-06	7.2333E-09	3.601474	down	7148	TNXB	
CB_021167	1.2071E-06	7.2327E-09	4.511064	down	6196	RPS6KA2	
CB_021704	1.2071E-06	7.1585E-09	7.561323	down	7145	TNS1	
CB_021165	1.20384E-06	7.0993E-09	4.932023	down	6196	RPS6KA2	
CB_015312	1.20228E-06	7.0523E-09	2.3441	down	10608	MXD4	
CB_010042	1.20228E-06	7.071E-09	4.735818	down	730005	LOC730005	
CB_009588	1.18373E-06	6.8493E-09	5.621477	down	316	AOX1	
CB_013349	1.18373E-06	6.8413E-09	32.47904	down	2277	FIGF	
CB_027935	1.18248E-06	6.8046E-09	5.214189	down	126668	TDRD10	

CB_014171	1.16168E-06	6.6113E-09	4.655263	down	2869	GRK5	
CB_006558	1.15956E-06	6.5413E-09	2.183452	down	492311	C5orf53	
CB_017988	1.15956E-06	6.544E-09	3.448579	down	23500	DAAM2	
CB_005724	1.15239E-06	6.4263E-09	3.422404	down	4881	NPR1	
CB_028859	1.15239E-06	6.4461E-09	6.583248	down	92162	TMEM88	
CB_011117	1.14869E-06	6.3603E-09	10.10976	down	3164	NR4A1	
CB_016161	1.14228E-06	6.2953E-09	4.506548	down	22918	CD93	
CB_005355	1.13599E-06	6.2309E-09	6.52217	down	2159	F10	
CB_024414	1.13166E-06	6.1893E-09	4.378386	down	115677	NOSTRIN	
CB_015923	1.1309E-06	6.1556E-09	2.664349	down	7402	UTRN	
CB_006694	1.09776E-06	5.7325E-09	3.397636	down	152503	SH3D19	
CB_021703	1.09776E-06	5.7428E-09	5.098504	down	7145	TNS1	
CB_017645	1.09776E-06	5.7274E-09	9.419062	down	22998	LIMCH1	
CB_021108	1.07648E-06	5.5369E-09	12.06535	down	653	BMP5	
CB_027236	1.07648E-06	5.5461E-09	16.40779	down	199675	C19orf59	
CB_013696	1.07496E-06	5.4793E-09	4.966921	down	9411	ARHGAP29	
CB_005772	1.05217E-06	5.3375E-09	5.358245	down	5730	PTGDS	
CB_020276	1.00208E-06	4.9722E-09	7.399662	down	54538	ROBO4	
CB_017198	9.93075E-07	4.9118E-09	3.754067	down	29995	LMCD1	
CB_023244	9.87835E-07	4.8545E-09	2.37526	down	23731	C9orf5	
CB_009621	9.86135E-07	4.8305E-09	3.930568	down	221	ALDH3B1	
CB_007217	9.77173E-07	4.7273E-09	3.604918	down	91663	MYADM	
CB_028294	9.67617E-07	4.6404E-09	2.370802	down	64236	PDLIM2	
CB_005582	9.67617E-07	4.6478E-09	5.308975	down	1555	CYP2B6	
CB_008463	9.66368E-07	4.5958E-09	8.358195	down	221303	FAM162B	
CB_006665	9.59086E-07	4.5156E-09	2.973644	down	11030	RBPMS	
CB_024901	9.49472E-07	4.4553E-09	6.126986	down	168667	BMPER	
CB_008788	9.49472E-07	4.4505E-09	9.551544	down	22854	NTNG1	
CB_022429	9.21567E-07	4.2659E-09	4.006277	down	79901	CYBRD1	
CB_029199	9.14124E-07	4.188E-09	7.275643	down	387700	SLC16A12	
CB_010844	9.06316E-07	4.1274E-09	4.823332	down	1346	COX7A1	
CB_022206	9.06316E-07	4.1378E-09	8.508825	down	79674	VEPH1	
CB_011934	9.05478E-07	4.0959E-09	3.363643	down	6275	S100A4	
CB_010546	9.04863E-07	4.0738E-09	3.066039	down	3340	NDST1	
CB_017212	9.04863E-07	4.0692E-09	3.322599	down	30846	EHD2	
CB_010408	8.56779E-07	3.7894E-09	5.739498	down	1901	S1PR1	
CB_020359	8.48147E-07	3.7378E-09	12.99715	down	56241	SUSD2	
CB_012733	7.93312E-07	3.4333E-09	3.298955	down	8742	TNFSF12	
CB_026852	7.92524E-07	3.4173E-09	33.703	down	177	AGER	
CB_027417	7.88152E-07	3.386E-09	3.665749	down	79742	CXorf36	
CB_024608	7.59143E-07	3.2252E-09	4.021616	down	23460	ABCA6	
CB_024160	7.57525E-07	3.206E-09	8.540078	down	148	ADRA1A	
CB_012580	7.57525E-07	3.1895E-09	10.29657	down	8516	ITGA8	
CB_014847	7.54556E-07	3.1497E-09	3.375029	down	7466	WFS1	
CB_005690	7.54556E-07	3.0993E-09	3.465288	down	3384	ICAM2	
CB_013239	7.52193E-07	3.0765E-09	2.422852	down	977	CD151	
CB_005167	7.42989E-07	2.9946E-09	6.307633	down	5745	PTH1R	
CB_010899	6.94641E-07	2.7199E-09	8.984638	down	1675	CFD	
CB_018123	6.78473E-07	2.5921E-09	4.822264	down	25959	KANK2	
CB_009025	6.77146E-07	2.5334E-09	4.908627	down	7122	CLDN5	
CB_012920	6.65089E-07	2.4638E-09	7.270796	down	1910	EDNRB	
CB_026223	6.65089E-07	2.4672E-09	8.116093	down	153579	BTNL9	
CB_018937	6.63491E-07	2.4297E-09	3.43324	down	51294	PCDH12	
CB_021365	6.46915E-07	2.3382E-09	8.023247	down	60495	HPSE2	
CB_005297	6.40493E-07	2.2639E-09	3.961692	down	5175	PECAM1	
CB_018664	6.37329E-07	2.2114E-09	7.479603	down	51705	EMCN	

CB_016378	6.23174E-07	2.1428E-09	4.450276	down	23266	LPHN2	
CB_007477	6.19382E-07	2.0524E-09	2.151375	down	79836	LONRF3	
CB_026224	6.06692E-07	1.9812E-09	11.65285	down	153579	BTNL9	
CB_023207	5.99178E-07	1.9377E-09	4.049558	down	83878	USHBP1	
CB_009860	5.99178E-07	1.9346E-09	4.25408	down	3603	IL16	
CB_011279	5.96989E-07	1.8928E-09	2.869422	down	3913	LAMB2	
CB_022508	5.96246E-07	1.8691E-09	2.504569	down	80005	DOCK5	
CB_016264	5.96246E-07	1.8394E-09	5.627266	down	8322	FZD4	
CB_010180	5.93718E-07	1.7977E-09	16.93007	down	11185	INMT	
CB_004763	5.91826E-07	1.7732E-09	24.02518	down			
CB_013722	5.89252E-07	1.7537E-09	2.691865	down	9467	SH3BP5	
CB_012665	5.85987E-07	1.7185E-09	13.02982	down	8639	AOC3	
CB_014452	5.76957E-07	1.6829E-09	3.45453	down	4593	MUSK	
CB_014201	5.67111E-07	1.6242E-09	2.388244	down	3092	HIP1	
CB_001735	5.60777E-07	1.5824E-09	4.431148	down			
CB_031093	5.60777E-07	1.5749E-09	18.76191	down	400550	LOC400550	
CB_016727	5.49593E-07	1.5237E-09	2.823747	down	30815	ST6GALNAC6	
CB_010736	5.49593E-07	1.5247E-09	4.125537	down	847	CAT	
CB_025441	5.45606E-07	1.4941E-09	9.648387	down	126393	HSPB6	
CB_011191	5.33916E-07	1.4219E-09	4.153154	down	3680	ITGA9	
CB_004895	5.30533E-07	1.4045E-09	2.621667	down	686	BTD	
CB_003237	5.19621E-07	1.3674E-09	6.37283	down			
CB_010233	5.09111E-07	1.3075E-09	4.285867	down	687	KLF9	
CB_011056	4.93283E-07	1.259E-09	3.262044	down	2878	GPX3	
CB_022428	4.76149E-07	1.2077E-09	3.993197	down	79901	CYBRD1	
CB_017961	4.74176E-07	1.1877E-09	3.298957	down	23371	TENC1	
CB_022339	4.7388E-07	1.1794E-09	4.092517	down	79812	MMRN2	
CB_031965	4.71277E-07	1.1655E-09	5.987141	down	255480	LOC255480	
CB_020360	4.71277E-07	1.161E-09	10.92001	down	56241	SUSD2	
CB_023841	4.70791E-07	1.1419E-09	10.35737	down	84913	ATOH8	
CB_009049	4.69869E-07	1.1239E-09	2.427351	down	9922	IQSEC1	
CB_009745	4.69869E-07	1.1277E-09	3.370557	down	5144	PDE4D	
CB_026759	4.69869E-07	1.0985E-09	4.903791	down	10044	SH2D3C	
CB_022168	4.60922E-07	1.0522E-09	5.078008	down	79633	FAT4	
CB_010738	4.55539E-07	1.0327E-09	6.566709	down	857	CAV1	
CB_018276	4.53179E-07	1.013E-09	4.968011	down	27145	FILIP1	
CB_025803	4.40732E-07	9.7815E-10	2.578784	down	8578	SCARF1	
CB_019827	4.34578E-07	9.5071E-10	16.24182	down	55273	TMEM100	
CB_009208	4.27212E-07	9.1433E-10	3.007915	down	9208	LRRFIP1	
CB_001325	4.27212E-07	9.2105E-10	10.35805	down	400568	LOC400568	
CB_005742	4.22363E-07	8.9721E-10	4.364817	down	5241	PGR	
CB_027288	4.19684E-07	8.7156E-10	5.853621	down	161198	CLEC14A	
CB_027936	4.19684E-07	8.6755E-10	6.202119	down	126668	TDRD10	
CB_008538	4.06269E-07	8.2438E-10	3.574796	down	23176	8-Sep	
CB_015856	4.06269E-07	8.1851E-10	24.83615	down	11095	ADAMTS8	
CB_010664	4.02345E-07	8.0366E-10	4.468179	down	482	ATP1B2	
CB_017354	4.02216E-07	7.9702E-10	3.190133	down	9754	STARD8	
CB_018814	3.96861E-07	7.8012E-10	5.199345	down	51751	HIGD1B	
CB_011592	3.83333E-07	7.4137E-10	3.552465	down	5187	PER1	
CB_005349	3.69788E-07	7.0345E-10	5.23512	down	1589	CYP21A2	
CB_011904	3.69772E-07	6.9756E-10	4.355655	down	6038	RNASE4	
CB_005541	3.29034E-07	5.7477E-10	8.46483	down	762	CA4	
CB_015311	3.27351E-07	5.5387E-10	2.774949	down	10608	MXD4	
CB_005641	3.15644E-07	5.1336E-10	16.50236	down	2890	GRIA1	
CB_015184	3.0288E-07	4.8014E-10	5.964	down	10516	FBLN5	
CB_017526	2.78811E-07	4.3315E-10	3.519285	down	9936	CD302	

CB_027566	2.75139E-07	4.1225E-10	6.196846	down	6711	SPTBN1	
CB_026585	2.75139E-07	4.1683E-10	18.02492	down	256691	MAMDC2	
CB_010181	2.71854E-07	3.8786E-10	7.041021	down	11185	INMT	
CB_003950	2.28746E-07	3.1548E-10	5.657918	down			
CB_014926	2.17913E-07	2.9227E-10	3.97558	down	10370	CITED2	
CB_016309	2.1328E-07	2.8063E-10	3.40725	down	284119	PTRF	
CB_005314	2.05751E-07	2.5441E-10	6.224293	down	7010	TEK	
CB_012177	1.88217E-07	2.2378E-10	18.25979	down	6943	TCF21	
CB_027499	1.72457E-07	1.9411E-10	4.154817	down	90627	STARD13	
CB_020750	1.69645E-07	1.8825E-10	4.666706	down	57381	RHOJ	
CB_020322	1.43372E-07	1.5038E-10	15.27534	down	7148	TNXB	
CB_028429	1.39086E-07	1.4111E-10	4.177772	down	256949	KANK3	
CB_017134	1.35633E-07	1.3075E-10	3.864007	down	27303	RBMS3	
CB_020251	1.35633E-07	1.3116E-10	5.303517	down	54507	ADAMTSL4	
CB_005313	1.25683E-07	1.1556E-10	6.773453	down	6928	HNF1B	
CB_000273	1.23352E-07	1.0976E-10	4.21531	down	11030	RBPMS	
CB_009597	9.74099E-08	8.0071E-11	15.78845	down	2273	FHL1	
CB_017331	9.59356E-08	7.5383E-11	2.576534	down	9732	DOCK4	
CB_006957	9.59356E-08	7.6042E-11	7.258698	down	219348	PLAC9	
CB_016715	9.59356E-08	7.466E-11	8.388946	down	395	ARHGAP6	
CB_014949	8.85176E-08	6.5575E-11	6.210699	down	10411	RAPGEF3	
CB_015858	7.96303E-08	5.6806E-11	6.08879	down	11099	PTPN21	
CB_011377	7.96303E-08	5.3345E-11	13.81932	down	4239	MFAP4	
CB_014278	7.88723E-08	5.1137E-11	5.418807	down	7075	TIE1	
CB_012236	7.88723E-08	5.1264E-11	24.25688	down	7123	CLEC3B	
CB_010992	7.40792E-08	4.4625E-11	4.302376	down	2324	FLT4	
CB_010777	7.40792E-08	4.3962E-11	4.819443	down	1003	CDH5	
CB_026830	7.08828E-08	4.0452E-11	7.395188	down	130271	PLEKHH2	
CB_020325	7.08828E-08	3.8977E-11	12.21431	down	7148	TNXB	
CB_020324	7.08828E-08	3.9497E-11	13.00802	down	7148	TNXB	
CB_009378	6.83393E-08	3.3086E-11	3.661793	down	283	ANG	
CB_010307	6.83393E-08	3.365E-11	4.00155	down	9079	LDB2	
CB_028041	6.83393E-08	3.4667E-11	7.223291	down	10395	DLC1	
CB_011799	6.76338E-08	3.1093E-11	9.523437	down	5787	PTPRB	
CB_022293	6.39133E-08	2.7356E-11	2.296748	down	79758	DHRS12	
CB_020944	6.39133E-08	2.7265E-11	3.75161	down	57631	LRCH2	
CB_024009	6.29792E-08	2.496E-11	8.661529	down	23345	SYNE1	
CB_027770	6.22146E-08	2.2868E-11	5.130161	down	83849	SYT15	
CB_010473	6.22146E-08	2.367E-11	10.0653	down	2327	FMO2	
CB_024893	6.22146E-08	2.0797E-11	11.31436	down	147372	CCBE1	
CB_015975	6.22146E-08	1.9203E-11	15.61282	down	11170	FAM107A	
CB_018770	6.22146E-08	2.0199E-11	16.03716	down	51208	CLDN18	
CB_013914	5.45611E-08	1.3839E-11	8.291745	down	5348	FXYD1	
CB_010306	5.28683E-08	1.239E-11	6.086524	down	9079	LDB2	
CB_026831	4.64041E-08	7.3563E-12	6.275165	down	130271	PLEKHH2	
CB_011813	3.9125E-08	5.5422E-12	5.273172	down	5797	PTPRM	
CB_018685	3.9125E-08	4.1624E-12	6.31802	down	10365	KLF2	
CB_020256	3.88791E-08	2.4653E-12	4.69193	down	54511	HMGCLL1	
CB_023970	2.509E-08	1.1932E-12	8.580241	down	64651	CSRNP1	
CB_023969	2.509E-08	6.9342E-13	8.790145	down	64651	CSRNP1	
CB_014966	2.509E-08	1.1233E-12	10.06403	down	395	ARHGAP6	

Table S3A Differentially expressed lncRNA between smokers and non-smokers in the tumor tissue

ProbeName	p (Corr)	FC (abs)	Regulation	Transcripts Ensemble_68	Biotype-Transcripts Ensemble_68	Name Ensemble_68
RNA49700|UCSC_8831_1084	0.004043973	2.40162	Up	ENST00000508936	antisense	RP11-301H24.3
RNA147283|nc-HOXB9-192	0.029772477	2.51484	Up	ENST00000572218	intronic	KANSL1
RNA176963|ENST00000422008	0.025944404	2.05086	Up	ENST00000422008	intronic	GAS5
RNA178219|ENST00000523995	0.00128834	2.9063	Up	ENST00000523995	intronic	RP11-37B2.1
RNA58121|UCRs_436_205	0.021102734	2.19614	Up	ENST00000507721	intronic	HNRPDL
RNA61153|RNAz_1867_274	0.011021987	2.44971	Up	ENST00000513732	intronic	GPBP1
RNA61627|RNAz_2341_260	0.021102734	2.76601	Up	ENST00000515481	intronic	HNRNPH1
RNA174244|ENST00000414938	0.023841633	3.17142	Up	ENST00000414938	lincRNA	EGOT
RNA33832|lncRNAdb_39_1529	0.027396227	3.26838	Up	ENST00000414938	lincRNA	EGOT
RNA35519|ENCODE_383_1588	0.025944404	2.34779	Up	ENST00000420981	lincRNA	RP11-420G6.4
RNA176282|ENST00000511876	0.014070939	2.19207	Up	ENST00000511876	processed_transcript	CTC-454M9.1
RNA178218|ENST00000521996	0.003666628	3.00616	Up	ENST00000521996	processed_transcript	RP11-37B2.1
RNA33830|lncRNAdb_37_1554	0.035059078	2.55355	Up	ENST00000487314	processed_transcript	FAS
RNA40510|RefSeq_2569_1210	0.014070939	2.02335	Up	ENST00000561287	processed_transcript	UBE2Q2P3
RNA40511|RefSeq_2570_1210	0.013925476	2.13871	Up	ENST00000561287	processed_transcript	UBE2Q2P3
RNA50672|UCSC_10027_801	0.00270696	3.69984	Up	ENST00000476135	processed_transcript	HDAC9
RNA51557|UCSC_11124_473	0.02196707	2.14361	Up	ENST00000500559	processed_transcript	RP11-33B1.1
RNA60529|RNAz_1241_293	0.00270696	2.68832	Up	ENST00000460432	processed_transcript	DCAF6
RNA63789|RNAz_4509_219	0.009684487	2.01589	Up	ENST00000494632	processed_transcript	CBWD6
RNA50987|UCSC_10402_692	0.03027634	2.13221	Up	ENST00000462600	processed_transcript	HLA-J
RNA144077|snRNA_30_328	0.021102734	2.1229	Up	ENST00000362605	unknown	7SK
RNA50126|UCSC_9340_976	0.011021987	2.51826	Up	ENST00000437814	unknown	METTL15
RNA51509|UCSC_11070_492	0.02034267	2.16097	Up	ENST00000314501	unknown	AC009951.1
RNA175363|ENST00000537961	0.038786301	2.65253	Down	ENST00000537961	lincRNA	RP11-598F7.6
RNA37014|ENCODE_1884_485	0.035059078	5.47501	Down	ENST00000452852	lincRNA	LINC00393
RNA178460|ENST00000439725	0.021102734	8.6255	Down	ENST00000535745	processed_transcript	H19
RNA178461|ENST00000411861	0.021102734	8.65622	Down	ENST00000535745	processed_transcript	H19
RNA178462|ENST00000417089	0.021102734	8.43756	Down	ENST00000535745	processed_transcript	H19
RNA178463|ENST00000446406	0.02196707	8.0195	Down	ENST00000535745	processed_transcript	H19

RNA178464|ENST00000431095	0.021102734	8.19828	Down	ENST00000535745	processed_transcript	H19
RNA178465|ENST00000411754	0.02196707	8.08122	Down	ENST00000535745	processed_transcript	H19
RNA178466|ENST00000422826	0.02196707	8.46618	Down	ENST00000535745	processed_transcript	H19
RNA178467|ENST00000428066	0.021016364	7.82526	Down	ENST00000535745	processed_transcript	H19
RNA178468|ENST00000436715	0.036307048	8.32599	Down	ENST00000535745	processed_transcript	H19
RNA178469|ENST00000447298	0.024432166	7.47442	Down	ENST00000535745	processed_transcript	H19
RNA178470|ENST00000442037	0.021102734	8.26445	Down	ENST00000535745	processed_transcript	H19
RNA178471|ENST00000535745	0.02604372	7.9301	Down	ENST00000535745	processed_transcript	H19
RNA33817|lncRNAdb_24_2322	0.02359837	7.98041	Down	ENST00000535745	processed_transcript	H19
RNA46974|UCSC_5536_1867	0.021563914	8.13938	Down	ENST00000535745	processed_transcript	H19
RNA47740|UCSC_6480_1652	0.02196707	8.03363	Down	ENST00000535745	processed_transcript	H19
RNA48150|UCSC_6979_1542	0.021102734	8.70724	Down	ENST00000535745	processed_transcript	H19
RNA48555|UCSC_7465_1417	0.02196707	8.36596	Down	ENST00000535745	processed_transcript	H19
RNA50503|UCSC_9805_863	0.023831301	7.36635	Down	ENST00000535745	processed_transcript	H19
RNA53698|H-InvDB_974_445	0.023831301	7.70622	Down	ENST00000535745	processed_transcript	H19
RNA54074|H-InvDB_1367_377	0.025944404	7.88597	Down	ENST00000535745	processed_transcript	H19
RNA58194|CombinedLit_52_225	0.021102734	8.54929	Down	ENST00000535745	processed_transcript	H19

Table S3B Differentially expressed lncRNA between poor and moderate differentiation patients in the tumor tissue

ProbeName	p (Corr)	FC (abs)	Regulatior	Transcripts Ensemble_68	Biotype-Transcripts Ensemble_68	Name Ensemble_68
RNA176087|ENST00000539666	0.042676502	2.1737	Down	ENST00000539666	processed_transcript	ZNF518A
RNA63690|RNAz_4410_221	0.013545528	2.04691	Down	Unkown	Unkown	Unkown
RNA63765|RNAz_4485_219	0.033415105	2.17446	Down	Unkown	Unkown	Unkown
RNA34706|NRED_838_1591	0.021263157	2.22058	Down	Unkown	Unkown	Unkown
RNA45999|UCSC_4347_2146	0.018766701	2.29934	Down	Unkown	Unkown	Unkown
RNA96657|EvoFold_500_211	0.03205033	2.3387	Down	Unkown	Unkown	Unkown
RNA35111|NRED_1252_426	0.022288028	2.3614	Down	Unkown	Unkown	Unkown
RNA96642|EvoFold_483_213	0.03205033	2.4155	Down	Unkown	Unkown	Unkown
RNA96208|EvoFold_18_363	0.03033404	2.42385	Down	Unkown	Unkown	Unkown

RNA57856|UCRs_171_263	0.03205033	2.46561	Down	Unkown	Unkown	Unkown
RNA59509|RNAz_220_402	0.027349381	2.51227	Down	Unkown	Unkown	Unkown
RNA60947|RNAz_1661_277	0.045196943	2.64847	Down	Unkown	Unkown	Unkown
RNA62309|RNAz_3027_239	0.022288028	2.65308	Down	Unkown	Unkown	Unkown
RNA163746|XLOC_004366	0.013545528	2.70618	Down	Unkown	Unkown	Unkown
RNA55283|H-InvDB_2657_229	0.03033404	3.13858	Down	Unkown	Unkown	Unkown
RNA64855|RNAz_5576_200	0.03205033	3.60921	Down	Unkown	Unkown	Unkown
RNA62153|RNAz_2871_240	0.01993647	4.45984	Down	Unkown	Unkown	Unkown
RNA47169|UCSC_5771_1814	0.042676502	6.59296	Down	Unkown	Unkown	Unkown

Table S4A The lncRNA probes expressed with same direction between our study and GSE56850 dataset									
ProbeName	p (Corr)	p	FC (abs)	Regulatio	GSE56850 regulation	Transcripts Ensemble_68	Biotype-Transcripts Ensemble_68	Name Ensemble_68	
ENCODE_360_1711	0.00066	7.17E-05	2.05471	down	down	ENST00000366278	antisense	AC105053.3	
UCSC_6895_1560	6.92E-05	3.36E-06	6.44117	down	down	ENST00000377178	antisense	RP11-195E11.3	
NRED_1227_577	9.46E-06	1.88E-07	4.23917	down	down	ENST00000411904	antisense	RP11-251M1.1	
ENST00000415561	0.00035	3.04E-05	2.26685	down	down	ENST00000415561	antisense	AC009948.3	
NRED_623_1917	1.70E-05	4.46E-07	3.07111	down	down	ENST00000502125	antisense	RP11-327J17.3	
NRED_622_1917	3.57E-05	1.26E-06	3.07126	down	down	ENST00000502125	antisense	RP11-327J17.3	
XLOC_004435	0.00036	3.12E-05	2.04794	down	down	ENST00000512650	antisense	CTC-564N23.2	
asoverlaps_762_993	2.44E-05	7.48E-07	2.36672	down	down	ENST00000512856	antisense	CTD-2260A17.1	
NRED_400_2352	1.34E-05	3.09E-07	7.65951	down	down	ENST00000529215	antisense	CTD-2562J17.7	
NRED_665_1838	2.09E-05	5.97E-07	3.90056	down	down	ENST00000560778	antisense	RP11-2E17.1	
NRED_980_1313	2.46E-05	7.61E-07	4.10755	down	down	ENST00000560778	antisense	RP11-2E17.1	
asoverlaps_444_1823	4.06E-06	5.23E-08	4.52867	down	down	ENST00000581080	antisense	CTD-2267D19.2	
ENCODE_149_2503	1.94E-05	5.39E-07	4.46772	down	down	ENST00000412204	lincRNA	BX571672.2	
ENCODE_404_1497	0.01292	0.00438	3.02078	down	down	ENST00000412485	lincRNA	GS1-600G8.5	
ENCODE_514_1109	0.00013	8.02E-06	2.90682	down	down	ENST00000413897	lincRNA	BX571672.2	
ENCODE_1685_527	3.97E-05	1.49E-06	2.66216	down	down	ENST00000415575	lincRNA	RP11-203H2.2	
ENCODE_1546_554	0.00902	0.00268	2.34051	down	down	ENST00000417650	lincRNA	RP11-439L18.1	
ENCODE_2224_432	6.42E-05	3.02E-06	3.72359	down	down	ENST00000419428	lincRNA	RP11-356N1.2	
ENCODE_1641_537	4.62E-07	1.06E-09	5.04469	down	down	ENST00000420825	lincRNA	RP1-251M9.2	
ENCODE_1090_686	0.00233	0.00041	2.72957	down	down	ENST00000421019	lincRNA	RP11-290F20.3	
UCSC_7090_1512	0.00068	7.49E-05	2.67229	down	down	ENST00000421976	lincRNA	AC109642.1	
UCSC_10438_679	2.23E-06	2.05E-08	5.85273	down	down	ENST00000421976	lincRNA	AC109642.1	
ENCODE_259_2010	5.65E-05	2.50E-06	4.42771	down	down	ENST00000422459	lincRNA	RP11-290F20.3	
ENCODE_1630_539	4.10E-05	1.55E-06	4.9904	down	down	ENST00000422459	lincRNA	RP11-290F20.3	
RefSeq_1234_2469	0.00035	3.01E-05	2.57356	down	down	ENST00000427111	lincRNA	RP11-118B22.2	
UCSC_8328_1198	0.00024	1.81E-05	3.03492	down	down	ENST00000428624	lincRNA	BX004987.5	
ENCODE_150_2495	1.04E-05	2.13E-07	4.60712	down	down	ENST00000428624	lincRNA	BX004987.5	
ENCODE_1803_501	0.00018	1.20E-05	2.5328	down	down	ENST00000431700	lincRNA	BX004987.5	

ENCODE_1846_493	0.0007	7.76E-05	3.00209	down	down	ENST00000433724	lincRNA	AC116609.1		
ENCODE_513_1109	0.0001	5.87E-06	2.34573	down	down	ENST00000433980	lincRNA	BX004987.5		
XLOC_001808	1.69E-05	4.40E-07	2.58136	down	down	ENST00000434645	lincRNA	AC007283.5		
RefSeq_1599_2113	1.85E-05	5.04E-07	3.30951	down	down	ENST00000437047	lincRNA	AC034187.2		
ENCODE_2830_279	0.0004	3.64E-05	2.51467	down	down	ENST00000442663	lincRNA	BX004987.3		
ENCODE_2673_338	0.0053	0.00128	2.02246	down	down	ENST00000446290	lincRNA	RP11-274B18.2		
RefSeq_3109_693	4.70E-07	1.11E-09	11.0439	down	down	ENST00000446372	lincRNA	SFTA1P		
ENCODE_2411_399	1.36E-07	1.34E-10	11.8381	down	down	ENST00000446372	lincRNA	SFTA1P		
ENCODE_675_873	1.39E-07	1.39E-10	12.2044	down	down	ENST00000446372	lincRNA	SFTA1P		
ENCODE_2871_254	2.13E-07	2.74E-10	12.6751	down	down	ENST00000446372	lincRNA	SFTA1P		
ENCODE_2256_428	0.00017	1.11E-05	3.72944	down	down	ENST00000451023	lincRNA	RP11-356N1.2		
ENCODE_1192_645	0.00027	2.18E-05	2.80782	down	down	ENST00000452503	lincRNA	SKINTL		
ENCODE_1353_594	0.00606	0.00154	3.00472	down	down	ENST00000453787	lincRNA	RP11-151D14.1		
ENST00000522771	0.00997	0.00308	2.57679	down	down	ENST00000455531	lincRNA	MEG3		
UCSC_2881_2670	0.00578	0.00144	2.62733	down	down	ENST00000455531	lincRNA	MEG3		
ENCODE_2462_388	0.00086	0.0001	3.91837	down	down	ENST00000456460	lincRNA	BX004987.5		
ENCODE_2620_352	5.81E-05	2.60E-06	4.13814	down	down	ENST00000456795	lincRNA	RP3-331H24.4		
UCSC_308_5871	7.69E-05	3.93E-06	2.06293	down	down	ENST00000480258	lincRNA	DCP1A		
XLOC_003239	6.34E-06	1.03E-07	7.60941	down	down	ENST00000488425	lincRNA	RP11-88I21.2		
RefSeq_2411_1378	0.00013	8.31E-06	4.10172	down	down	ENST00000508732	lincRNA	CTD-2536I1.1		
ENST00000536729	0.00724	0.00197	2.24947	down	down	ENST00000536729	lincRNA	RP11-444D3.1		
ENST00000446891	0.00581	0.00145	2.27079	down	down	ENST00000536729	lincRNA	RP11-444D3.1		
ENST00000537961	0.00013	8.18E-06	2.21644	down	down	ENST00000537961	lincRNA	RP11-598F7.6		
UCSC_8997_1047	0.00068	7.46E-05	5.63286	down	down	ENST00000548886	lincRNA	RMST		
H-InvDB_1801_324	2.75E-05	8.81E-07	2.06507	down	down	ENST00000555882	lincRNA	DIO3OS		
ENST00000556899	5.73E-05	2.55E-06	4.35647	down	down	ENST00000556899	lincRNA	CTD-2536I1.1		
RefSeq_2345_1434	9.97E-05	5.67E-06	2.07905	down	down	ENST00000571091	lincRNA	MIR22HG		
RefSeq_1083_2654	0.00013	8.16E-06	2.08943	down	down	ENST00000571091	lincRNA	MIR22HG		
RefSeq_2441_1351	0.00011	6.12E-06	2.11227	down	down	ENST00000571091	lincRNA	MIR22HG		
ENST00000334146	0.00011	6.44E-06	2.13819	down	down	ENST00000571091	lincRNA	MIR22HG		
ENST00000435523	1.53E-06	1.12E-08	3.06424	down	down	ENST00000579072	lincRNA	AC058791.2		
ENST00000416999	1.87E-06	1.56E-08	3.53724	down	down	ENST00000580227	lincRNA	AC058791.2		
NRED_992_1278	4.82E-05	1.96E-06	2.54478	down	down	ENST00000581174	lincRNA	AC058791.2		
ENST00000423414	1.22E-05	2.69E-07	2.65102	down	down	ENST00000584405	lincRNA	AC058791.2		

XLOC_000264	3.88E-05	1.43E-06	7.77133	down	down	ENST00000413519	processed_transcript	RP11-183M13.1	
ENST00000418813	0.00051	5.02E-05	2.89768	down	down	ENST00000418813	processed_transcript	LINC00478	
ENCODE_2881_244	4.97E-08	8.72E-12	13.2377	down	down	ENST00000419627	processed_transcript	RP3-323P13.2	
UCSC_1084_3948	0.00031	2.54E-05	2.26916	down	down	ENST00000420823	processed_transcript	AC090044.1	
ENCODE_2086_451	1.04E-06	5.27E-09	6.55226	down	down	ENST00000421704	processed_transcript	LINC00472	
ENCODE_91_2957	4.61E-05	1.83E-06	4.04602	down	down	ENST00000426635	processed_transcript	LINC00472	
ENST00000419952	0.00012	7.45E-06	3.23477	down	down	ENST00000428669	processed_transcript	LINC00478	
ENST00000436211	0.00645	0.00168	2.05676	down	down	ENST00000436211	processed_transcript	BX248398.1	
ENST00000438676	1.13E-06	6.16E-09	3.89146	down	down	ENST00000438676	processed_transcript	RP1-234P15.4	
UCSC_2792_2713	0.00442	0.00099	2.07682	down	down	ENST00000441044	processed_transcript	MTMR9LP	
UCSC_7487_1410	0.00416	0.00091	2.1834	down	down	ENST00000441044	processed_transcript	MTMR9LP	
RefSeq_1363_2324	0.003	0.00058	2.25155	down	down	ENST00000441044	processed_transcript	MTMR9LP	
ENCODE_1954_475	3.76E-06	4.71E-08	5.44542	down	down	ENST00000441570	processed_transcript	LINC00472	
ENST00000418974	0.00423	0.00093	2.19589	down	down	ENST00000445243	processed_transcript	RP11-495P10.1	
ENST00000445461	0.0005	4.87E-05	2.91877	down	down	ENST00000445461	processed_transcript	LINC00478	
ENST00000453910	0.00061	6.37E-05	2.57534	down	down	ENST00000453910	processed_transcript	LINC00478	
RefSeq_735_3278	0.00197	0.00032	3.14006	down	down	ENST00000458468	processed_transcript	LINC00478	
RefSeq_645_3475	0.00157	0.00024	3.23812	down	down	ENST00000458468	processed_transcript	LINC00478	
XLOC_013869	0.00118	0.00016	3.34569	down	down	ENST00000458468	processed_transcript	LINC00478	
H-InvDB_2592_237	9.57E-06	1.91E-07	2.67514	down	down	ENST00000464796	processed_transcript	TAL1	
RefSeq_865_3008	1.00E-04	5.69E-06	2.42887	down	down	ENST00000465070	processed_transcript	ZEB2	
ENST00000474768	1.27E-05	2.85E-07	5.13556	down	down	ENST00000474768	processed_transcript	ADAMTS9-AS2	
UCSC_8569_1143	3.78E-05	1.37E-06	3.41919	down	down	ENST00000482258	processed_transcript	PAPSS2	
RefSeq_1291_2410	0.00756	0.0021	2.12106	down	down	ENST00000483658	processed_transcript	KALRN	
UCSC_2168_3068	0.00418	0.00092	2.14006	down	down	ENST00000483658	processed_transcript	KALRN	
UCSC_2471_2871	0.00258	0.00047	2.7412	down	down	ENST00000485055	processed_transcript	CCDC39	
UCSC_6252_1706	9.10E-05	4.99E-06	3.34206	down	down	ENST00000485493	processed_transcript	ERG	
UCSC_11583_309	0.00031	2.51E-05	22.6708	down	down	ENST00000491208	processed_transcript	SFTPA2	
H-InvDB_3014_207	0.00032	2.66E-05	24.5639	down	down	ENST00000491208	processed_transcript	SFTPA2	
NRED_1180_814	0.00028	2.21E-05	2.62528	down	down	ENST00000491442	processed_transcript	HIVEP3	
NRED_712_1775	0.00051	4.99E-05	2.64316	down	down	ENST00000491442	processed_transcript	HIVEP3	
UCSC_7628_1373	4.61E-05	1.84E-06	2.22721	down	down	ENST00000504086	processed_transcript	ITGA1	
RefSeq_1020_2755	4.96E-05	2.06E-06	2.89877	down	down	ENST00000504474	processed_transcript	RP11-65F13.2	
ENST00000504932	4.31E-05	1.67E-06	2.61525	down	down	ENST00000504932	processed_transcript	RP11-677M14.3	

NRED_1049_1145	4.75E-06	6.66E-08	6.60145	down	down	ENST00000508269	processed_transcript	RP11-291L15.2		
ENST00000509179	0.00029	2.32E-05	2.23886	down	down	ENST00000508521	processed_transcript	CTC-454M9.1		
ENST00000510284	0.00034	2.92E-05	2.28086	down	down	ENST00000510284	processed_transcript	RP11-701P16.5		
XLOC_005755	1.52E-05	3.75E-07	8.43289	down	down	ENST00000511849	processed_transcript	RP1-240B8.3		
ENST00000511876	0.00016	1.08E-05	2.05913	down	down	ENST00000511876	processed_transcript	CTC-454M9.1		
ENST00000512359	6.33E-05	2.96E-06	8.33538	down	down	ENST00000512359	processed_transcript	RP11-291L15.2		
ENST00000512519	0.00156	0.00023	2.42714	down	down	ENST00000512519	processed_transcript	CTD-2127H9.1		
ENST00000532353	0.00043	3.99E-05	2.22087	down	down	ENST00000532353	processed_transcript	TRAF3IP2-AS1		
ENST00000532579	0.00012	7.30E-06	4.62017	down	down	ENST00000532579	processed_transcript	RP11-677M14.3		
UCSC_6618_1623	0.00148	0.00022	2.36022	down	down	ENST00000539983	processed_transcript	PZP		
UCSC_3870_2294	1.91E-05	5.29E-07	3.65901	down	down	ENST00000556961	processed_transcript	FBLN5		
RefSeq_1646_2073	1.13E-06	6.17E-09	9.53017	down	down	ENST00000563852	processed_transcript	RP11-506G7.1		
UCSC_9337_977	1.46E-05	3.53E-07	4.77959	down	down	ENST00000564564	processed_transcript	MT1JP		
UCSC_10849_559	0.00028	2.21E-05	5.9903	down	down	ENST00000564974	processed_transcript	MT1X		
ENCODE_208_2228	5.22E-06	7.74E-08	3.9599	down	down	ENST00000579235	processed_transcript	AC058791.1		
NRED_172_3224	7.71E-05	3.94E-06	2.45454	down	down	ENST00000580522	processed_transcript	AC058791.2		
ENST00000443623	9.36E-05	5.19E-06	2.5833	down	down	ENST00000580522	processed_transcript	AC058791.2		
ENST00000451786	4.81E-05	1.95E-06	2.60441	down	down	ENST00000580522	processed_transcript	AC058791.2		
NRED_447_2251	4.89E-05	2.01E-06	2.65069	down	down	ENST00000580522	processed_transcript	AC058791.2		
ENST00000444003	2.09E-06	1.82E-08	4.6971	down	down	ENST00000582369	processed_transcript	AC058791.1		
ENST00000447430	2.23E-05	6.55E-07	2.97619	down	down	ENST00000584325	processed_transcript	AC016831.7		
UCSC_1064_3978	5.13E-06	7.57E-08	3.39169	down	down	ENST00000452320	intronic	MAGI2-AS3		
RNAz_2931_240	0.00388	0.00083	2.92	down	down	ENST00000470802	intronic	ROBO2		
H-InvDB_862_482	3.10E-05	1.04E-06	2.13253	down	down	ENST00000506361	intronic	MYLK		
UCSC_7413_1427	0.00014	8.80E-06	2.67872	down	down	ENST00000524231	intronic	ABCA10		
ENST00000549723	0.01619	0.00594	2.21326	down	down	ENST00000549723	intronic	RP11-887P2.1		
UCSC_876_4275	4.89E-05	2.01E-06	3.42853	down	down	ENST00000555379	intronic	CTD-2552B11.4		
ENST00000418546	7.43E-07	3.00E-09	4.45192	down	down	ENST00000577311	intronic	AC058791.1		
NRED_915_1440	0.00075	8.6E-05	2.48369	down	down	ENST00000584798	intronic	EIF4A1		
XLOC_003222	6.01E-06	9.46E-08	8.28471	down	down	ENST00000493545	intronic	RP11-768G7.2		
UCSC_10738_593	0.00048	4.60E-05	2.83017	down	down	ENST00000515882	intronic	SLIT2-IT1		
XLOC_005763	0.00823	0.00237	2.02038	down	down					
UCSC_3622_2372	0.00158	0.00024	2.02674	down	down					
NRED_382_2401	0.00169	0.00026	2.03147	down	down					

RNAz_4497_219	9.3E-05	5.15E-06	2.03164	down	down					
RNAz_4287_224	0.00091	0.00011	2.03963	down	down					
RefSeq_250_4981	0.00348	0.00071	2.04353	down	down					
RefSeq_337_4427	7.03E-05	3.44E-06	2.05609	down	down					
XLOC_010574	8.89E-05	4.83E-06	2.06623	down	down					
XLOC_005275	2.17E-05	6.28E-07	2.07123	down	down					
XLOC_002005	0.02855	0.01251	2.08598	down	down					
UCSC_968_4134	0.00048	4.59E-05	2.09136	down	down					
RNAz_5618_200	0.00703	0.00189	2.0996	down	down					
UCRs_364_215	0.00012	7.08E-06	2.10341	down	down					
RNAz_884_315	6.81E-06	1.13E-07	2.11703	down	down					
RefSeq_165_5771	0.00032	2.72E-05	2.11819	down	down					
XLOC_002461	0.00756	0.0021	2.13813	down	down					
UCSC_4669_2069	0.00805	0.00229	2.13897	down	down					
XLOC_003821	0.00243	0.00043	2.14807	down	down					
XLOC_006299	0.00167	0.00026	2.15292	down	down					
UCSC_8057_1266	0.00024	1.83E-05	2.15694	down	down					
p0209_imsncRNA319	0.00019	1.31E-05	2.16255	down	down					
RNAz_4881_208	0.00021	1.49E-05	2.17289	down	down					
XLOC_005449	0.001	0.00013	2.17595	down	down					
EvoFold_183_267	0.00233	0.00041	2.18114	down	down					
XLOC_007412	0.00071	7.96E-05	2.19441	down	down					
UCSC_7527_1401	0.00011	6.73E-06	2.20486	down	down					
RNAz_2685_241	0.00476	0.0011	2.20811	down	down					
RNAz_729_320	2.20E-05	6.41E-07	2.22673	down	down					
RNAz_4511_219	0.00028	2.26E-05	2.23226	down	down					
UCRs_471_201	0.00097	0.00012	2.27708	down	down					
UCSC_4731_2053	0.00482	0.00112	2.28456	down	down					
RNAz_3643_234	7.97E-05	4.12E-06	2.29719	down	down					
UCSC_786_4462	1.05E-05	2.18E-07	2.3071	down	down					
EvoFold_492_212	0.00027	2.10E-05	2.31332	down	down					
asoverlaps_80_3270	3.71E-05	1.34E-06	2.33086	down	down					
XLOC_001181	1.03E-05	2.11E-07	2.34507	down	down					
RNAz_4027_229	0.00196	0.00032	2.34635	down	down					

RNAz_4629_216	0.00884	0.00261	2.35562	down	down					
NRED_968_1341	0.00144	0.00021	2.35816	down	down					
RefSeq_2723_1072	0.00023	1.68E-05	2.3791	down	down					
UCSC_3836_2305	1.91E-05	5.25E-07	2.39485	down	down					
UCSC_9759_873	0.00071	7.90E-05	2.41648	down	down					
UCSC_6027_1755	0.01963	0.00766	2.42976	down	down					
UCSC_4721_2054	2.14E-05	6.12E-07	2.43347	down	down					
NRED_787_1672	0.00169	0.00026	2.47403	down	down					
XLOC_014361	0.00014	8.47E-06	2.51149	down	down					
UCSC_7180_1489	0.00324	0.00064	2.52287	down	down					
NRED_231_2922	0.00305	0.00059	2.5247	down	down					
H-InvDB_1548_350	0.00813	0.00233	2.54536	down	down					
RNAz_1231_294	4.09E-06	5.30E-08	2.55801	down	down					
RNAz_323_381	9.72E-05	5.48E-06	2.59151	down	down					
XLOC_007827	0.00029	2.29E-05	2.60424	down	down					
RNAz_2084_269	0.00094	0.00012	2.60828	down	down					
RNAz_2522_250	0.00033	2.82E-05	2.61921	down	down					
XLOC_011171	0.00262	0.00048	2.62377	down	down					
asoverlaps_92_3152	2.37E-05	7.16E-07	2.62892	down	down					
UCSC_4102_2222	8.26E-05	4.34E-06	2.63056	down	down					
RNAz_2652_243	4.54E-05	1.79E-06	2.68383	down	down					
p0384_imsncRNA801	0.00392	0.00084	2.69832	down	down					
XLOC_012542	0.00169	0.00026	2.73408	down	down					
RNAz_1_1128	0.0001	5.87E-06	2.7383	down	down					
UCSC_7612_1379	0.00028	2.21E-05	2.74665	down	down					
UCSC_2676_2772	0.00117	0.00016	2.74983	down	down					
XLOC_011287	7.62E-05	3.86E-06	2.76048	down	down					
RefSeq_2738_1064	3.57E-05	1.27E-06	2.84576	down	down					
XLOC_011880	0.005	0.00118	2.87258	down	down					
UCSC_2675_2772	0.00033	2.73E-05	2.93837	down	down					
UCSC_113_7541	1.41E-05	3.33E-07	3.13454	down	down					
XLOC_001475	0.00065	7.07E-05	3.20527	down	down					
XLOC_012707	5.59E-05	2.47E-06	3.24599	down	down					
H-InvDB_191_799	6.35E-06	1.03E-07	3.25327	down	down					

p0340_imsncRNA761	2.08E-06	1.80E-08	3.28417	down	down					
UCSC_11731_220	4.13E-06	5.39E-08	3.36231	down	down					
RNAz_5393_200	0.00269	0.0005	3.37518	down	down					
p0263_imsncRNA449	0.00051	5.09E-05	3.41149	down	down					
NRED_624_1917	0.00095	0.00012	3.54678	down	down					
XLOC_010719	0.0018	0.00028	3.55683	down	down					
XLOC_014378	0.00221	0.00038	3.60467	down	down					
XLOC_001412	2.94E-05	9.63E-07	3.64486	down	down					
XLOC_011341	0.00154	0.00023	3.64857	down	down					
UCSC_1173_3851	1.87E-06	1.56E-08	3.80811	down	down					
XLOC_007456	0.00047	4.57E-05	3.87054	down	down					
XLOC_002825	5.93E-06	9.29E-08	3.88698	down	down					
RNAz_2035_270	3.55E-05	1.25E-06	3.98994	down	down					
RNAz_5170_200	1.48E-05	3.62E-07	4.00895	down	down					
UCSC_9132_1018	0.0004	3.57E-05	4.06469	down	down					
p0127_imsncRNA205	0.00529	0.00127	4.17835	down	down					
XLOC_003852	2.04E-07	2.49E-10	4.2099	down	down					
NRED_1222_606	2.49E-05	7.71E-07	4.22027	down	down					
RNAz_1612_278	0.00012	7.49E-06	4.41432	down	down					
XLOC_003595	0.00084	9.86E-05	4.41697	down	down					
RNAz_2640_244	5.20E-07	1.36E-09	4.52561	down	down					
RNAz_1978_271	6.35E-05	2.97E-06	4.55622	down	down					
UCSC_4028_2247	8.80E-06	1.69E-07	4.56508	down	down					
XLOC_008450	0.00044	4.14E-05	4.57596	down	down					
XLOC_002772	3.71E-05	1.34E-06	4.60764	down	down					
UCSC_4696_2062	6.50E-06	1.06E-07	4.65323	down	down					
UCSC_11438_359	0.00051	5.04E-05	4.90318	down	down					
asoverlaps_90_3155	9.26E-05	5.11E-06	5.09984	down	down					
RefSeq_3194_620	2.83E-05	9.14E-07	5.11554	down	down					
XLOC_001242	5.67E-07	1.62E-09	5.21768	down	down					
RNAz_4005_229	3.55E-06	4.37E-08	5.30306	down	down					
RNAz_368_366	0.00024	1.80E-05	5.56738	down	down					
NRED_933_1416	1.76E-05	4.68E-07	5.6397	down	down					
H-InvDB_607_577	1.16E-06	6.59E-09	6.09419	down	down					

XLOC_011172	0.00047	4.48E-05	6.25251	down	down					
UCSC_11018_508	1.72E-05	4.54E-07	6.29449	down	down					
H-InvDB_808_501	2.38E-06	2.28E-08	6.78242	down	down					
asoverlaps_56_3602	0.00016	1.05E-05	8.05654	down	down					
asoverlaps_609_1447	1.75E-06	1.39E-08	8.57328	down	down					
UCSC_4960_2001	4.70E-07	1.13E-09	8.6538	down	down					
XLOC_001474	6.15E-07	2.02E-09	9.52264	down	down					
ENST00000499071	3.83E-07	7.37E-10	9.59983	down	down					
ENST00000499142	4.76E-07	1.20E-09	10.0756	down	down					
NRED_755_1719	1.09E-05	2.31E-07	11.3049	down	down					
RefSeq_1070_2676	5.94E-07	1.79E-09	11.7986	down	down					
XLOC_004926	6.22E-08	1.71E-11	13.0128	down	down					
ENST00000501311	3.03E-06	3.28E-08	16.1293	down	down					
RefSeq_813_3099	2.02E-06	1.74E-08	16.7115	down	down					
XLOC_012046	1.81E-06	1.48E-08	16.9874	down	down					
H-InvDB_1005_436	1.75E-06	1.38E-08	28.2072	down	down					
ENST00000423121	0.01144	0.00371	2.1073	up	up	ENST00000423121	antisense	RP11-466F5.8		
ENST00000425981	0.03185	0.0144	3.61647	up	up	ENST00000425981	antisense	AC009264.1		
ENST00000490916	0.00219	0.00037	2.20567	up	up	ENST00000490916	antisense	RP11-204J18.3		
ENST00000536474	0.0112	0.00361	2.76842	up	up	ENST00000536474	antisense	FAM222A-AS1		
ENST00000541723	0.00235	0.00041	2.86595	up	up	ENST00000536474	antisense	FAM222A-AS1		
XLOC_006883	0.00907	0.0027	2.27803	up	up	ENST00000523831	antisense	KB-1615E4.2		
XLOC_006154	0.01466	0.0052	2.55037	up	up	ENST00000438324	antisense	RP5-1185I7.1		
XLOC_004238	0.00026	1.97E-05	3.2593	up	up	ENST00000515085	antisense	RP11-310P5.2		
XLOC_009398	4.9E-05	2.02E-06	11.0536	up	up	ENST00000531363	antisense	RP11-1L12.3		
ENCODE_2663_340	0.00531	0.00128	2.04992	up	up	ENST00000582801	antisense	RP11-561O23.8		
RefSeq_1312_2380	0.00276	0.00052	2.30312	up	up	ENST00000566699	antisense	RP4-659J6.2		
UCSC_2313_2962	0.0001	5.97E-06	2.89103	up	up	ENST00000565254	antisense	RP11-50D9.3		
ENCODE_1219_634	0.00105	0.00014	2.93959	up	up	ENST00000425295	antisense	KDM5B-AS1		
UCSC_7259_1470	0.00762	0.00212	2.99401	up	up	ENST00000430027	antisense	DLX6-AS1		
asoverlaps_186_2547	2.19E-05	6.36E-07	3.01774	up	up	ENST00000565254	antisense	RP11-50D9.3		
ENCODE_925_748	0.00173	0.00027	3.13799	up	up	ENST00000425295	antisense	KDM5B-AS1		
asoverlaps_775_970	0.00029	2.36E-05	3.26294	up	up	ENST00000414354	antisense	MFI2-AS1		
RefSeq_1246_2455	0.00464	0.00106	3.67012	up	up	ENST00000539163	antisense	HNF1A-AS1		

RefSeq_2223_1560	0.00015	9.40E-06	5.22318	up	up	ENST00000434346	antisense	RP11-184I16.2		
RefSeq_1085_2653	0.00336	0.00068	5.4234	up	up	ENST00000428449	antisense	RP11-560I19.4		
UCSC_9100_1027	0.00173	0.00027	6.14039	up	up	ENST00000428449	antisense	RP11-560I19.4		
UCSC_5633_1845	9.44E-06	1.87E-07	8.76097	up	up	ENST00000380330	antisense	RP11-116G8.4		
RefSeq_2601_1178	0.0083	0.00239	2.99515	up	up	ENST00000536474	antisense	FAM222A-AS1		
ENST00000507152	0.00336	0.00068	2.09422	up	up	ENST00000507152	lincRNA	RP11-366M4.3		
ENST00000550019	0.00186	0.0003	2.28064	up	up	ENST00000550019	lincRNA	RP11-493L12.5		
ENST00000547285	0.00198	0.00033	2.40342	up	up	ENST00000547285	lincRNA	CTD-2314B22.3		
ENST00000546117	0.01298	0.00441	2.80853	up	up	ENST00000546117	lincRNA	RP11-173C20.2		
ENST00000418499	0.00061	6.39E-05	2.88154	up	up	ENST00000418499	lincRNA	AL589743.1		
ENST00000551726	0.01625	0.00597	2.89048	up	up	ENST00000551726	lincRNA	RP11-81H3.2		
ENST00000515416	0.00742	0.00204	3.15367	up	up	ENST00000551726	lincRNA	RP11-81H3.2		
ENST00000551881	3.10E-05	1.03E-06	5.60612	up	up	ENST00000551881	lincRNA	AL589743.1		
ENST00000548475	4.38E-06	5.86E-08	8.25804	up	up	ENST00000549813	lincRNA	CTD-2314B22.3		
ENST00000457958	3.96E-05	1.47E-06	8.80807	up	up	ENST00000457958	lincRNA	LINC00511		
ENST00000431060	0.00595	0.0015	2.1215	up	up	ENST00000431060	lincRNA	RP11-66B24.2		
XLOC_004062	0.00655	0.00172	2.17039	up	up	ENST00000507299	lincRNA	CTD-2325B11.1		
XLOC_002523	0.01918	0.00743	2.72376	up	up	ENST00000435896	lincRNA	AC010148.1		
XLOC_000364	0.00786	0.00222	2.75516	up	up	ENST00000442182	lincRNA	RP11-39H13.1		
XLOC_001763	0.01357	0.0047	2.7758	up	up	ENST00000426615	lincRNA	AC009336.24		
XLOC_003218	0.01399	0.00489	3.61477	up	up	ENST00000481624	lincRNA	RP11-572C15.5		
XLOC_001872	5.59E-05	2.47E-06	3.69554	up	up	ENST00000414512	lincRNA	AC067956.1		
XLOC_011856	0.00018	1.20E-05	3.76043	up	up	ENST00000575693	lincRNA	LA16c-325D7.2		
XLOC_006422	0.00027	2.14E-05	4.28328	up	up	ENST00000456114	lincRNA	AC005537.2		
XLOC_004031	0.02109	0.00842	4.75349	up	up	ENST00000509399	lincRNA	RP11-297P16.4		
ENCODE_2077_453	0.01036	0.00325	2.00854	up	up	ENST00000455010	lincRNA	RP11-90C4.3		
RefSeq_2431_1362	0.00253	0.00046	2.01029	up	up	ENST00000440089	lincRNA	AC105339.1		
ENCODE_57_3328	0.0387	0.01849	2.01782	up	up	ENST00000380888	lincRNA	RP4-610C12.4		
RefSeq_1365_2322	0.0098	0.00301	2.02642	up	up	ENST00000518580	lincRNA	RP11-109J4.1		
RefSeq_752_3221	0.00473	0.00109	2.02946	up	up	ENST00000571152	lincRNA	LINC00514		
ENCODE_2846_271	0.00017	1.10E-05	2.04912	up	up	ENST00000416563	lincRNA	RP13-329D4.3		
RefSeq_2826_982	0.00889	0.00263	2.05566	up	up	ENST00000520457	lincRNA	HPYR1		
ENCODE_1592_546	0.01409	0.00494	2.17139	up	up	ENST00000414401	lincRNA	CECR7		
ENCODE_2310_418	0.00076	8.70E-05	2.21782	up	up	ENST00000414126	lincRNA	AC073316.2		

ENCODE_942_743	0.0004	3.64E-05	2.2316	up	up	ENST00000454128	lincRNA	AF127936.3	
ENCODE_1488_566	0.03961	0.01903	2.37418	up	up	ENST00000412563	lincRNA	AC007091.1	
ENCODE_2452_389	0.00264	0.00048	2.40011	up	up	ENST00000422548	lincRNA	RP5-968D22.1	
UCSC_7506_1406	0.00161	0.00025	2.4217	up	up	ENST00000547285	lincRNA	CTD-2314B22.3	
UCSC_7509_1405	0.00262	0.00048	2.42568	up	up	ENST00000547285	lincRNA	CTD-2314B22.3	
lncRNAdb_41_1441	0.00917	0.00275	2.49065	up	up	ENST00000397381	lincRNA	UCA1	
ENCODE_545_1017	0.00182	0.00029	2.50827	up	up	ENST00000437648	lincRNA	RP11-146I2.1	
ENCODE_1726_517	0.0029	0.00055	2.59693	up	up	ENST00000438499	lincRNA	RP11-1M18.1	
ENCODE_273_1977	0.00615	0.00157	2.60701	up	up	ENST00000445865	lincRNA	AC007880.1	
UCSC_10810_570	0.00019	1.34E-05	2.69617	up	up	ENST00000549065	lincRNA	CTD-2314B22.3	
ENCODE_1068_694	0.00079	9.21E-05	2.97923	up	up	ENST00000427017	lincRNA	RP1-182D15.2	
UCSC_3536_2395	0.00038	3.33E-05	3.05766	up	up	ENST00000455088	lincRNA	CTD-2314B22.3	
NRED_815_1629	0.00847	0.00246	3.07166	up	up	ENST00000545400	lincRNA	RP11-153K16.1	
NRED_1111_987	0.00658	0.00173	3.12592	up	up	ENST00000551726	lincRNA	RP11-81H3.2	
UCSC_5122_1962	0.00558	0.00137	3.12882	up	up	ENST00000500092	lincRNA	RP11-519M16.1	
NRED_828_1611	0.00025	1.89E-05	3.18113	up	up	ENST00000546186	lincRNA	CTD-2335A18.1	
ENCODE_1661_532	0.00278	0.00052	3.31708	up	up	ENST00000453395	lincRNA	LA16c-83F12.6	
ENCODE_1187_647	0.00018	1.20E-05	3.37163	up	up	ENST00000439703	lincRNA	RP11-417E7.1	
ENCODE_1110_678	0.00693	0.00186	3.43152	up	up	ENST00000432481	lincRNA	AC009410.1	
ENCODE_1948_475	1.37E-05	3.21E-07	3.57523	up	up	ENST00000415205	lincRNA	Z83851.3	
ENCODE_637_905	0.00096	0.00012	3.83957	up	up	ENST00000449673	lincRNA	AC092669.6	
ENCODE_870_774	0.00181	0.00029	4.52631	up	up	ENST00000437790	lincRNA	AC092669.2	
ENCODE_516_1103	0.0003	2.44E-05	4.54432	up	up	ENST00000439259	lincRNA	AC012494.1	
ENCODE_2013_465	9.77E-05	5.52E-06	4.67676	up	up	ENST00000452675	lincRNA	RP11-367G18.1	
UCSC_7583_1386	3.87E-05	1.43E-06	5.18079	up	up	ENST00000551881	lincRNA	AL589743.1	
UCSC_7588_1385	3.14E-05	1.05E-06	5.39882	up	up	ENST00000551881	lincRNA	AL589743.1	
UCSC_962_4142	2.96E-05	9.71E-07	5.40589	up	up	ENST00000552602	lincRNA	CTD-2314B22.3	
H-InvDB_1894_318	0.00017	1.18E-05	5.6941	up	up	ENST00000445613	lincRNA	AC104088.1	
ENCODE_2706_327	0.00015	9.56E-06	7.64244	up	up	ENST00000427157	lincRNA	RP11-367G18.1	
NRED_446_2251	9.34E-05	5.17E-06	7.80277	up	up	ENST00000457958	lincRNA	LINC00511	
NRED_779_1681	4.13E-05	1.57E-06	9.07579	up	up	ENST00000457958	lincRNA	LINC00511	
ENCODE_1762_510	0.00037	3.31E-05	10.1267	up	up	ENST00000446484	lincRNA	AC009262.2	
UCSC_8570_1142	0.00039	3.54E-05	13.7697	up	up	ENST00000523313	lincRNA	RP11-697M17.1	
ENST00000504833	7.52E-05	3.79E-06	2.0825	up	up	ENST00000504833	processed_transcript	CTD-2001C12.1	

ENST00000509037	0.04177	0.02036	2.08827	up	up	ENST00000509037	processed_transcript	RP11-19O2.2		
ENST00000510576	0.03714	0.01756	2.16459	up	up	ENST00000510576	processed_transcript	CTB-113P19.1		
ENST00000512617	0.0171	0.00638	2.1876	up	up	ENST00000521600	processed_transcript	PVT1		
ENST00000521600	0.0029	0.00055	2.20547	up	up	ENST00000521600	processed_transcript	PVT1		
ENST00000519104	0.0148	0.00526	2.23398	up	up	ENST00000519104	processed_transcript	RP3-399L15.3		
ENST00000517864	0.0003	2.40E-05	2.26485	up	up	ENST00000517864	processed_transcript	RP11-320N21.1		
ENST00000430666	0.00316	0.00062	2.39282	up	up	ENST00000430666	processed_transcript	AC092937.2		
ENST00000524165	0.00039	3.46E-05	2.52766	up	up	ENST00000522963	processed_transcript	PVT1		
ENST00000458666	0.01171	0.00383	2.5602	up	up	ENST00000458666	processed_transcript	AC087859.1		
ENST00000458151	0.0004	3.64E-05	2.68575	up	up	ENST00000458151	processed_transcript	RP5-1024G6.5		
ENST00000473776	0.00231	0.0004	2.73858	up	up	ENST00000473776	processed_transcript	RP5-894A10.5		
ENST00000521951	0.00038	3.31E-05	2.83275	up	up	ENST00000521951	processed_transcript	PVT1		
ENST00000424735	0.00048	4.62E-05	2.92845	up	up	ENST00000436905	processed_transcript	RP11-547I7.2		
ENST00000517790	0.00213	0.00036	2.95517	up	up	ENST00000517790	processed_transcript	PVT1		
ENST00000427794	0.00042	3.92E-05	3.04933	up	up	ENST00000427794	processed_transcript	RP5-907D15.2		
ENST00000520913	0.00112	0.00015	3.13553	up	up	ENST00000517838	processed_transcript	PVT1		
ENST00000414438	0.0061	0.00156	3.47235	up	up	ENST00000414438	processed_transcript	AC069277.2		
ENST00000510016	0.00203	0.00034	3.8233	up	up	ENST00000510016	processed_transcript	RP11-8L2.1		
ENST00000519481	0.00028	2.24E-05	3.95447	up	up	ENST00000519481	processed_transcript	PVT1		
ENST00000523190	0.00033	2.77E-05	3.99355	up	up	ENST00000523190	processed_transcript	PVT1		
ENST00000522875	9.71E-05	5.47E-06	4.30271	up	up	ENST00000522875	processed_transcript	PVT1		
ENST00000414603	0.00271	0.0005	4.37647	up	up	ENST00000414603	processed_transcript	AC069277.2		
ENST00000508406	9.04E-05	4.94E-06	5.51791	up	up	ENST00000508406	processed_transcript	RP11-8L2.1		
ENST00000432385	0.00032	2.65E-05	6.05881	up	up	ENST00000432385	processed_transcript	RP11-385J1.2		
ENST00000492337	0.0012	0.00016	8.10203	up	up	ENST00000492337	processed_transcript	SOX2-OT		
ENST00000458250	1.65E-05	4.26E-07	8.9731	up	up	ENST00000458250	processed_transcript	MIR205HG		
ENST00000526388	3.86E-05	1.42E-06	9.22232	up	up	ENST00000526388	processed_transcript	CTC-497E21.4		
ENST00000529328	3.31E-05	1.14E-06	10.2439	up	up	ENST00000526388	processed_transcript	CTC-497E21.4		
ENST00000366437	0.00025	1.87E-05	14.6308	up	up	ENST00000366437	processed_transcript	MIR205HG		
ENST00000431096	0.00015	9.82E-06	14.7474	up	up	ENST00000366437	processed_transcript	MIR205HG		
ENST00000440276	0.00014	8.70E-06	14.8979	up	up	ENST00000366437	processed_transcript	MIR205HG		
ENST00000433108	0.00054	5.39E-05	22.9758	up	up	ENST00000433108	processed_transcript	MIR205HG		
XLOC_004204	0.00631	0.00163	2.18204	up	up	ENST00000317596	processed_transcript	RP11-290F5.2		
XLOC_008583	0.00035	3.05E-05	2.41833	up	up	ENST00000454935	processed_transcript	LINC00263		

XLOC_012236	0.0021	0.00035	2.44893	up	up	ENST00000515692	processed_transcript	RP11-304F15.4		
XLOC_001856	9.36E-05	5.19E-06	2.00833	up	up	ENST00000427280	processed_transcript	AC073321.5		
XLOC_002916	0.00266	0.00049	2.16508	up	up	ENST00000469301	processed_transcript	RP11-379K17.4		
XLOC_012093	0.00459	0.00104	2.09759	up	up	ENST00000563763	processed_transcript	WSCD1		
ENCODE_560_1008	0.00061	6.40E-05	2.07652	up	up	ENST00000422414	processed_transcript	RP4-781K5.7		
asoverlaps_369_1983	0.03173	0.01433	2.12441	up	up	ENST00000510576	processed_transcript	CTB-113P19.1		
ENCODE_141_2551	8.06E-05	4.19E-06	2.40154	up	up	ENST00000454935	processed_transcript	LINC00263		
UCSC_11096_482	0.00065	7.01E-05	2.61351	up	up	ENST00000522963	processed_transcript	PVT1		
NRED_1022_1208	0.00031	2.58E-05	2.87409	up	up	ENST00000458151	processed_transcript	RP5-1024G6.5		
NRED_872_1536	0.00049	4.73E-05	2.97828	up	up	ENST00000521951	processed_transcript	PVT1		
ENCODE_1120_674	0.00381	0.00081	3.28333	up	up	ENST00000437892	processed_transcript	RP11-124L5.7		
UCSC_2544_2838	0.00012	7.25E-06	5.44903	up	up	ENST00000508406	processed_transcript	RP11-8L2.1		
RefSeq_1214_2495	0.00129	0.00018	7.23221	up	up	ENST00000492337	processed_transcript	SOX2-OT		
UCSC_4403_2131	0.00337	0.00068	8.53175	up	up	ENST00000500112	processed_transcript	RP11-255B23.3		
UCSC_11599_304	0.00574	0.00142	2.08252	up	up	ENST00000527329	processed_transcript	RP11-652L8.2		
UCSC_10811_570	0.00465	0.00106	2.68055	up	up	ENST00000447037	processed_transcript	AP001469.9		
UCSC_10510_656	0.00022	1.58E-05	3.15863	up	up	ENST00000416066	processed_transcript	RP11-498P14.3		
UCSC_1502_3551	0.00046	4.41E-05	3.51678	up	up	ENST00000444312	processed_transcript	ATG9B		
UCSC_10960_529	0.02091	0.00833	2.02906	up	up	ENST00000548086	processed_transcript	SCN8A		
NRED_977_1318	0.00056	5.67E-05	2.07943	up	up	ENST00000545509	processed_transcript	ITFG2		
UCSC_10777_583	0.00365	0.00076	2.08727	up	up	ENST00000483169	processed_transcript	DLEU1		
UCSC_5554_1862	0.00092	0.00011	2.10425	up	up	ENST00000461681	processed_transcript	ITGB3BP		
NRED_941_1403	0.003	0.00058	2.18437	up	up	ENST00000479850	processed_transcript	CCDC169		
UCSC_8383_1186	0.00036	3.13E-05	2.36823	up	up	ENST00000462427	processed_transcript	DLEU1		
UCSC_11251_431	0.00045	4.23E-05	2.39428	up	up	ENST00000462427	processed_transcript	DLEU1		
CombinedLit_465_209	0.00239	0.00042	2.56445	up	up	ENST00000479817	processed_transcript	MNX1		
UCSC_8683_1119	0.00022	1.62E-05	2.57785	up	up	ENST00000462427	processed_transcript	DLEU1		
UCSC_3871_2293	0.00879	0.00259	2.60102	up	up	ENST00000455723	processed_transcript	TMSB15B		
UCSC_9753_875	0.00061	6.40E-05	2.98383	up	up	ENST00000462427	processed_transcript	DLEU1		
UCSC_10940_534	0.00046	4.39E-05	3.00504	up	up	ENST00000484606	processed_transcript	DLEU1		
CombinedLit_267_667	0.00096	0.00012	3.02131	up	up	ENST00000470593	processed_transcript	DLEU1		
UCSC_7187_1487	0.00059	6.16E-05	3.04509	up	up	ENST00000470593	processed_transcript	DLEU1		
CombinedLit_279_638	3.44E-05	1.20E-06	3.14983	up	up	ENST00000498557	processed_transcript	DLEU1		
UCSC_8188_1233	0.00032	2.65E-05	3.18703	up	up	ENST00000470593	processed_transcript	DLEU1		

CombinedLit_440_285	0.00092	0.00011	3.21645	up	up	ENST00000470726	processed_transcript	DLEU1		
UCSC_7735_1344	0.00024	1.79E-05	3.21873	up	up	ENST00000470593	processed_transcript	DLEU1		
UCSC_7722_1348	0.00027	2.08E-05	3.23965	up	up	ENST00000470593	processed_transcript	DLEU1		
UCSC_9065_1034	0.00049	4.72E-05	3.26381	up	up	ENST00000470593	processed_transcript	DLEU1		
UCSC_7240_1473	2.09E-05	5.94E-07	3.28554	up	up	ENST00000476901	processed_transcript	TRMU		
CombinedLit_400_355	0.00028	2.22E-05	3.32416	up	up	ENST00000470593	processed_transcript	DLEU1		
UCSC_8985_1051	0.00026	2.06E-05	3.44248	up	up	ENST00000468522	processed_transcript	DLEU1		
CombinedLit_387_387	0.00122	0.00017	3.51776	up	up	ENST00000498557	processed_transcript	DLEU1		
UCSC_7891_1304	0.00116	0.00015	3.77108	up	up	ENST00000483169	processed_transcript	DLEU1		
UCSC_6472_1652	0.00137	0.00019	3.83141	up	up	ENST00000484869	processed_transcript	DLEU1		
UCSC_9498_940	0.00595	0.0015	3.92769	up	up	ENST00000463474	processed_transcript	DLEU1		
UCSC_9080_1031	0.00607	0.00155	4.526	up	up	ENST00000462427	processed_transcript	DLEU1		
UCSC_11419_366	0.00076	8.67E-05	6.58505	up	up	ENST00000515325	processed_transcript	NMU		
RefSeq_1014_2768	0.02782	0.01209	2.53129	up	up	ENST00000575787	processed_transcript	ALOX12P2		
UCSC_4127_2215	0.02107	0.00841	2.67586	up	up	ENST00000575787	processed_transcript	ALOX12P2		
RefSeq_1746_1992	6.12E-05	2.82E-06	3.51771	up	up	ENST00000438684	processed_transcript	MSL3P1		
UCSC_8900_1070	0.00102	0.00013	3.7196	up	up	ENST00000570835	processed_transcript	ALOX12P2		
ENST00000439208	0.00088	0.00011	2.39986	up	up	ENST00000424351	retained_intron	AC017076.4		
ENST00000426452	0.00092	0.00011	4.30063	up	up	ENST00000426452	retained_intron	RP11-191L9.4		
ENST00000435967	0.00115	0.00015	4.66134	up	up	ENST00000435967	retained_intron	AC007128.1		
ENST00000425330	0.00043	4.00E-05	5.46614	up	up	ENST00000425330	retained_intron	RP11-385J1.2		
ENST00000476964	0.00514	0.00123	6.31482	up	up	ENST00000466034	retained_intron	SOX2-OT		
ENST00000466034	0.00321	0.00064	6.86801	up	up	ENST00000466034	retained_intron	SOX2-OT		
ENST00000493521	0.00189	0.00031	7.08534	up	up	ENST00000477928	retained_intron	SOX2-OT		
ENST00000477928	0.002	0.00033	7.41794	up	up	ENST00000477928	retained_intron	SOX2-OT		
H-InvDB_1573_346	0.00011	6.23E-06	5.86087	up	up	ENST00000421498	retained_intron	RP11-385J1.2		
UCSC_2985_2611	0.00501	0.00118	2.41481	up	up	ENST00000584132	retained_intron	SOCS7		
RefSeq_707_3337	2.26E-05	6.67E-07	3.21696	up	up	ENST00000488743	retained_intron	SLC5A6		
ENST00000509547	0.04352	0.02144	2.25082	up	up					
ENST00000530512	0.01562	0.00566	2.29962	up	up					
ENST00000502335	0.01338	0.00461	2.48389	up	up					
XLOC_012014	0.01163	0.00379	2.02726	up	up					
XLOC_007516	0.03719	0.01759	2.03624	up	up					
XLOC_000757	0.00016	1.03E-05	2.0536	up	up					

XLOC_009981	0.01766	0.00667	2.05551	up	up					
XLOC_013273	0.04556	0.02272	2.06486	up	up					
XLOC_001836	0.00863	0.00253	2.06876	up	up					
XLOC_005939	0.01568	0.00569	2.07024	up	up					
XLOC_005220	0.00076	8.71E-05	2.20028	up	up					
XLOC_014255	0.00252	0.00045	2.26224	up	up					
XLOC_014296	0.01372	0.00476	2.28607	up	up					
XLOC_011570	0.00054	5.38E-05	2.35205	up	up					
XLOC_012333	0.00282	0.00053	2.39352	up	up					
XLOC_006922	0.00198	0.00033	2.48312	up	up					
XLOC_001877	0.00935	0.00282	2.4872	up	up					
XLOC_002360	0.00911	0.00272	2.53729	up	up					
XLOC_006924	0.0033	0.00066	2.60019	up	up					
XLOC_010709	7.59E-05	3.84E-06	2.63668	up	up					
XLOC_005989	0.0152	0.00545	2.79874	up	up					
XLOC_001716	0.00786	0.00222	2.95555	up	up					
XLOC_006923	0.00169	0.00026	3.03711	up	up					
XLOC_000456	0.00242	0.00043	3.20485	up	up					
XLOC_001983	0.00261	0.00048	3.25895	up	up					
XLOC_005511	0.00022	1.58E-05	3.29917	up	up					
XLOC_008109	0.00156	0.00023	3.32357	up	up					
XLOC_002204	0.00091	0.00011	3.37948	up	up					
XLOC_007116	0.01368	0.00474	3.38008	up	up					
XLOC_010390	0.00227	0.00039	3.95075	up	up					
XLOC_012684	0.00079	9.21E-05	4.28286	up	up					
XLOC_005433	0.00719	0.00196	4.58176	up	up					
XLOC_009782	0.00121	0.00016	4.60843	up	up					
XLOC_005810	6.34E-06	1.03E-07	4.75665	up	up					
XLOC_012683	8.85E-05	4.80E-06	5.10563	up	up					
RNAz_1000_310	0.00571	0.00141	2.01774	up	up					
RNAz_2992_239	0.0043	0.00096	2.02304	up	up					
asoverlaps_620_1422	0.04894	0.02486	2.03665	up	up					
RNAz_3211_238	0.0066	0.00173	2.06333	up	up					
H-InvDB_836_492	0.01807	0.00687	2.07806	up	up					

RNAz_2126_267	0.00607	0.00155	2.10433	up	up					
RNAz_1926_273	0.00047	4.54E-05	2.11834	up	up					
UCSC_4066_2233	0.00101	0.00013	2.12106	up	up					
RefSeq_1285_2415	0.01154	0.00376	2.12708	up	up					
RefSeq_2267_1518	0.00169	0.00026	2.13446	up	up					
UCSC_4778_2043	0.00915	0.00274	2.16728	up	up					
hox-HOXD9-38	0.00029	2.30E-05	2.19329	up	up					
RNAz_5388_200	0.00321	0.00064	2.21783	up	up					
RefSeq_2275_1512	0.0004	3.62E-05	2.23837	up	up					
RNAz_3730_233	0.00173	0.00027	2.25072	up	up					
RefSeq_2396_1389	0.02009	0.0079	2.25754	up	up					
RefSeq_1181_2526	0.00391	0.00084	2.27962	up	up					
RefSeq_2973_835	1.62E-05	4.12E-07	2.28151	up	up					
UCRs_414_207	0.00045	4.24E-05	2.28352	up	up					
hox-HOXC8-64	0.02431	0.01015	2.30271	up	up					
EvoFold_8_420	0.02447	0.01025	2.34749	up	up					
RNAz_3963_230	0.00173	0.00027	2.51971	up	up					
asoverlaps_592_1486	0.00154	0.00023	2.56602	up	up					
RNAz_153_431	0.00694	0.00186	2.5685	up	up					
RNAz_2719_240	0.00155	0.00023	2.59063	up	up					
RNAz_5122_200	0.00485	0.00113	2.61316	up	up					
RNAz_5101_200	0.02288	0.00939	2.63249	up	up					
RNAz_5083_201	0.0041	0.00089	2.6463	up	up					
RNAz_1289_288	0.00899	0.00267	2.67143	up	up					
H-InvDB_440_641	0.00253	0.00046	2.7627	up	up					
RefSeq_2830_978	0.00154	0.00023	2.76868	up	up					
RNAz_1441_280	0.00816	0.00234	2.7896	up	up					
RNAz_4580_217	0.00053	5.30E-05	2.80744	up	up					
H-InvDB_2505_248	0.00424	0.00094	2.84745	up	up					
UCSC_4950_2004	0.00144	0.00021	2.87103	up	up					
RefSeq_854_3016	0.00799	0.00227	2.88474	up	up					
RNAz_1792_275	0.00096	0.00012	2.93524	up	up					
RNAz_5259_200	0.00829	0.00239	2.98056	up	up					
RNAz_3732_233	0.00209	0.00035	2.98303	up	up					

RNAz_264_393	0.00928	0.00279	3.02327	up	up					
UCSC_10376_699	0.00132	0.00018	3.04601	up	up					
H-InvDB_1278_388	0.01964	0.00767	3.14567	up	up					
RNAz_2978_239	0.00104	0.00013	3.1639	up	up					
UCSC_7568_1390	0.00501	0.00118	3.28441	up	up					
RNAz_5646_200	0.00118	0.00016	3.29436	up	up					
UCSC_3717_2342	0.00936	0.00283	3.30447	up	up					
NRED_126_3551	4.33E-05	1.69E-06	3.31195	up	up					
EvoFold_206_261	0.00014	8.83E-06	3.465	up	up					
RNAz_3748_233	0.00308	0.0006	3.5083	up	up					
RNAz_5311_200	0.00805	0.00229	3.51187	up	up					
RNAz_1379_280	0.00014	8.61E-06	3.57143	up	up					
UCSC_2122_3100	0.00082	9.60E-05	3.77583	up	up					
RNAz_3199_238	0.01503	0.00537	3.82783	up	up					
UCSC_11604_300	0.00031	2.53E-05	3.84716	up	up					
RNAz_834_317	0.0005	4.96E-05	3.86941	up	up					
asoverlaps_141_2736	0.00115	0.00015	3.97106	up	up					
UCSC_11364_387	6.82E-05	3.29E-06	4.3368	up	up					
UCRs_124_279	0.00649	0.0017	4.66965	up	up					
RNAz_261_393	0.00645	0.00168	4.67669	up	up					
UCSC_812_4399	1.08E-05	2.29E-07	4.7306	up	up					
RNAz_4918_207	0.00038	3.36E-05	5.70558	up	up					
UCSC_8693_1117	3.46E-06	4.16E-08	8.70908	up	up					
RNAz_4515_219	3.97E-05	1.48E-06	8.79889	up	up					
p0194_imsncRNA293	0.00113	0.00015	2.01172	up	up					
p0216_imsncRNA329	0.00237	0.00042	2.01851	up	up					
p0768_imsncRNA553	0.0181	0.00689	2.02024	up	up					
p0565_imsncRNA229	0.03926	0.01883	2.06441	up	up					
p0211_imsncRNA320	0.01296	0.0044	2.15126	up	up					
p0197_imsncRNA298	5.81E-05	2.61E-06	2.23193	up	up					
p0408_imsncRNA823	0.00845	0.00246	2.53642	up	up					
p0575_imsncRNA253	0.0001	5.80E-06	5.91105	up	up					

Table S4B The lncRNA probes expressed with different direction between our study and GSE56850 dataset

ProbeName	p (Corr)	p	FC (abs)	Regulatio	GSE56850 regulation	Transcripts Ensemble_68	Biotype-Transcripts Ensemble_68	Name Ensemble_68
UCSC_10060_788	0.00083	9.83E-05	3.38	up	down	ENST00000439173	antisense	CSAG2
XLOC_004127	0.00305	0.00059	2.00358	up	down	ENST00000504957	lincRNA	RP11-18H21.3
XLOC_003402	0.01452	0.00513	2.34178	up	down	ENST00000438408	lincRNA	AC128709.3
ENCODE_937_745	0.03049	0.01362	2.07329	up	down	ENST00000580244	lincRNA	RP11-561O23.5
ENCODE_2209_435	0.014	0.00489	2.15152	up	down	ENST00000444130	lincRNA	AP000705.7
RefSeq_771_3182	0.02698	0.01162	2.17903	up	down	ENST00000580244	lincRNA	RP11-561O23.5
UCSC_6531_1641	0.02547	0.01079	2.20148	up	down	ENST00000580244	lincRNA	RP11-561O23.5
ENST00000483650	0.00856	0.0025	2.08836	up	down	ENST00000483650	processed_transcript	RP11-548O1.3
ENST00000453023	0.00103	0.00013	2.38858	up	down	ENST00000453023	processed_transcript	MIAT
ENST00000429250	0.00275	0.00051	2.49078	up	down	ENST00000429250	processed_transcript	RP11-90J7.3
UCSC_5320_1918	0.0111	0.00357	2.27722	up	down	ENST00000420224	processed_transcript	RP11-3B12.3
ENCODE_315_1832	0.01202	0.00397	2.24236	up	down	ENST00000573162	processed_transcript	AC145141.1
UCSC_437_5360	0.0085	0.00247	2.28436	up	down	ENST00000393525	processed_transcript	ANKRD36BP2
XLOC_006329	0.00937	0.00283	2.10512	up	down			
XLOC_008674	0.00584	0.00146	2.10762	up	down			
XLOC_013099	0.00495	0.00116	2.16243	up	down			
XLOC_001411	0.01574	0.00572	2.16721	up	down			
XLOC_002473	0.02097	0.00836	2.23577	up	down			
XLOC_007125	0.00952	0.00289	2.41143	up	down			
XLOC_007614	0.00849	0.00247	2.41922	up	down			
XLOC_014016	0.01035	0.00324	2.6013	up	down			
XLOC_000091	0.01244	0.00416	2.60929	up	down			
XLOC_011222	0.01238	0.00413	2.61752	up	down			
XLOC_008829	0.00524	0.00126	2.69743	up	down			
XLOC_002173	0.00052	5.15E-05	2.78506	up	down			
XLOC_012796	0.01204	0.00398	3.24052	up	down			
XLOC_000702	0.00118	0.00016	4.87006	up	down			

ENST00000483424	0.00704	0.0019	4.60421	up	down					
UCSC_9875_843	0.00707	0.00191	2.01571	up	down					
RNAz_1358_280	0.00737	0.00202	2.04722	up	down					
H-InvDB_2043_301	0.00926	0.00278	2.07236	up	down					
nc-HOXD4-36	0.00812	0.00232	2.08021	up	down					
UCSC_6189_1720	0.00319	0.00063	2.11782	up	down					
nc-HOXC6-252	0.02357	0.00976	2.14535	up	down					
RNAz_4884_208	0.00231	0.0004	2.18664	up	down					
RNAz_2290_262	0.00467	0.00107	2.20854	up	down					
nc-HOXC6-246	0.00627	0.00162	2.21044	up	down					
UCSC_7385_1433	0.01071	0.0034	2.2323	up	down					
UCSC_1935_3233	0.01262	0.00424	2.43269	up	down					
RNAz_3361_237	0.00103	0.00013	2.49073	up	down					
NRED_966_1346	0.0117	0.00382	2.65152	up	down					
H-InvDB_1797_324	0.02175	0.00878	2.76708	up	down					
RefSeq_1983_1758	0.03197	0.01447	2.90987	up	down					
UCSC_5130_1961	0.0018	0.00029	3.18185	up	down					
snRNA_57_318	0.00694	0.00186	2.02045	up	down					
p0889_imsncRNA704	0.01354	0.00468	2.00262	up	down					
p0598_imsncRNA307	0.0157	0.0057	2.00908	up	down					
p0545_imsncRNA172	0.01023	0.00319	2.09955	up	down					
p0824_imsncRNA624	0.00028	2.27E-05	2.13747	up	down					
p0874_imsncRNA684	0.01375	0.00478	2.36019	up	down			HOXD-AS1		
ENST00000425005	0.00358	0.00074	2.71105	down	up	ENST00000425005	antisense	RP4-755D9.1		
UCSC_4513_2102	0.00826	0.00238	3.20758	down	up	ENST00000553843	antisense	CASC2		
ENST00000439517	0.00065	7.01E-05	2.48517	down	up	ENST00000439517	lincRNA	AC019117.2		
ENCODE_2459_388	0.00464	0.00106	3.37536	down	up	ENST00000454003	lincRNA	RP11-76C10.5		
ENST00000542980	0.01934	0.00751	2.01994	down	up	ENST00000536673	lincRNA	RP5-1022P6.6		
ENCODE_1987_470	0.00224	0.00039	2.21908	down	up	ENST00000430333	processed_transcript	AP001046.5		
ENST00000442815	0.00319	0.00063	2.03134	down	up	ENST00000442815	processed_transcript	C1orf168		
UCSC_4189_2198	0.00028	2.24E-05	3.37604	down	up	ENST00000484327	processed_transcript	RP11-93L9.1		
ENST00000508111	0.00989	0.00305	2.36013	down	up	ENST00000508111	processed_transcript	NAPSB		
RefSeq_2471_1330	0.00182	0.00029	3.09191	down	up	ENST00000527780	processed_transcript			
XLOC_010798	0.01204	0.00398	2.02248	down	up					

UCSC_5273_1930	0.01727	0.00647	2.49522	down	up						
NRED_566_2018	0.00045	4.25E-05	3.16383	down	up						
XLOC_011889	0.0088	0.0026	3.40534	down	up						
XLOC_003910	0.00067	7.34E-05	3.99154	down	up						
UCSC_6948_1549	1.77E-05	4.72E-07	4.86546	down	up						

Table S5A The mRNA probes expressed with same direction between our study and TCGA dataset

ProbeName	p (Corr)	p	FC (abs)	Regulation	TCGA regulation	Entrez GeneID	Gene Symbol	
CB_018205	0.047907	0.024202	3.921805	up	up	26085	KLK13	
CB_023984	0.043876	0.021668	2.118228	up	up	56667	MUC13	
CB_006121	0.043073	0.021185	2.079051	up	up	312	ANXA13	
CB_015786	0.039858	0.019172	4.983169	up	up	7348	UPK1B	
CB_014153	0.039031	0.018686	2.058078	up	up	2831	NPBWR1	
CB_027501	0.037202	0.017596	4.119896	up	up	347733	TUBB2B	
CB_020904	0.037095	0.017535	2.576063	up	up	57586	SYT13	
CB_012986	0.036802	0.017359	2.014503	up	up	1690	COCH	
CB_011262	0.036681	0.017284	2.328313	up	up	3880	KRT19	
CB_009151	0.033845	0.015586	2.363683	up	up	120376	C11orf93	
CB_023321	0.033287	0.015263	2.102731	up	up	84070	FAM186B	
CB_012385	0.031958	0.014466	2.356782	up	up	7712	ZNF157	
CB_027547	0.031945	0.014458	2.072942	up	up	340596	LHFPL1	
CB_009426	0.031754	0.014346	2.033344	up	up	440854	CAPN14	
CB_024076	0.031752	0.014344	3.358024	up	up	10317	B3GALT5	
CB_021718	0.029077	0.012827	2.215856	up	up	6785	ELOVL4	
CB_024581	0.028408	0.012425	2.648712	up	up	7477	WNT7B	
CB_016613	0.027377	0.011848	2.00722	up	up	29906	ST8SIA5	
CB_012513	0.026941	0.0116	2.135823	up	up	8419	BFSP2	
CB_025258	0.02551	0.010813	3.106711	up	up	140469	MYO3B	
CB_011998	0.025276	0.010689	2.298021	up	up	6423	SFRP2	
CB_006594	0.025053	0.01057	2.164295	up	up	347252	IGFBPL1	
CB_013933	0.02489	0.010476	3.285437	up	up	5746	PTH2R	
CB_013978	0.0248	0.010424	2.094578	up	up	9242	MSC	
CB_011018	0.024701	0.010371	2.249524	up	up	2596	GAP43	
CB_004979	0.023946	0.009956	2.431278	up	up	2261	FGFR3	
CB_029176	0.022785	0.009331	2.396148	up	up	401138	AMTN	
CB_025486	0.022306	0.009069	2.765954	up	up	144406	WDR66	
CB_025743	0.021921	0.008866	2.258445	up	up	124220	ZG16B	
CB_015095	0.021147	0.008452	2.762603	up	up	653247	PRB2	
CB_016823	0.019283	0.007481	2.25305	up	up	54989	ZNF770	
CB_013826	0.019103	0.007386	2.439557	up	up	1833	EPYC	
CB_008619	0.018453	0.007059	2.401131	up	up	23244	PDS5A	
CB_010702	0.018418	0.007041	2.650365	up	up	656	BMP8B	
CB_020542	0.017757	0.006714	5.313235	up	up	57016	AKR1B10	
CB_020181	0.017687	0.006678	2.642816	up	up	53820	DSCR6	
CB_022763	0.017581	0.006625	2.943609	up	up	29785	CYP2S1	
CB_022817	0.017542	0.006607	2.675715	up	up	53637	S1PR5	
CB_010604	0.017339	0.006504	2.475599	up	up	197	AHSG	
CB_021181	0.017325	0.006497	2.286595	up	up	10309	CCNO	
CB_022693	0.017044	0.006353	2.302655	up	up	80326	WNT10A	
CB_008056	0.017043	0.006353	2.136912	up	up	613212	CTXN3	
CB_005893	0.01658	0.006134	2.53318	up	up	93273	LEMD1	
CB_019921	0.016468	0.006077	2.054599	up	up	55347	ABHD10	
CB_029039	0.016308	0.005999	4.222098	up	up	145741	C2CD4A	
CB_017815	0.016285	0.005988	2.860162	up	up	114088	TRIM9	
CB_031131	0.015965	0.005829	2.665074	up	up	283422	C12orf36	
CB_011207	0.015964	0.005829	2.005423	up	up	3714	JAG2	
CB_014501	0.015743	0.00572	2.098562	up	up	6756	SSX1	
CB_011615	0.015679	0.005691	4.504324	up	up	5266	PI3	

CB_013360	0.015576	0.005642	2.331873	up	up	2591	GALNT3	
CB_021879	0.01545	0.005576	2.760814	up	up	668	FOXL2	
CB_014262	0.015395	0.005551	2.677768	up	up	6373	CXCL11	
CB_015552	0.014484	0.005117	2.4321	up	up	10900	RUNDC3A	
CB_007057	0.014258	0.005012	3.741684	up	up	6706	SPRR2G	
CB_014078	0.014239	0.005002	3.331127	up	up	1475	CSTA	
CB_022642	0.014124	0.004954	2.378875	up	up	23120	ATP10B	
CB_013317	0.013955	0.004874	3.619193	up	up	2045	EPHA7	
CB_022995	0.013735	0.004772	2.013096	up	up	81931	ZNF93	
CB_028458	0.013308	0.004572	2.421711	up	up	341032	C11orf53	
CB_026912	0.013272	0.004555	3.429204	up	up	163778	SPRR4	
CB_027987	0.013169	0.004503	2.345808	up	up	254263	CNIH2	
CB_011211	0.013058	0.004447	2.224092	up	up	3728	JUP	
CB_022562	0.012789	0.004321	2.201171	up	up	80117	ARL14	
CB_006807	0.012732	0.004293	2.350509	up	up	441168	FAM26F	
CB_009857	0.012664	0.004263	4.338561	up	up	92211	CDHR1	
CB_025378	0.012646	0.004252	2.339213	up	up	11202	KLK8	
CB_009361	0.012608	0.004235	2.361419	up	up	54549	SDK2	
CB_014555	0.01239	0.004139	2.606496	up	up	10057	ABCC5	
CB_022479	0.012389	0.004138	2.267769	up	up	79966	SCD5	
CB_028888	0.012014	0.003969	3.889365	up	up	10683	DLL3	
CB_014269	0.01194	0.003936	4.963901	up	up	6707	SPRR3	
CB_014206	0.011818	0.003877	2.181718	up	up	3149	HMGB3	
CB_014825	0.011638	0.003798	4.323119	up	up	6698	SPRR1A	
CB_012961	0.011526	0.003748	2.663359	up	up	1015	CDH17	
CB_020731	0.010941	0.003497	2.062107	up	up	56675	NRIP3	
CB_007177	0.010854	0.003459	3.282262	up	up	7253	TSHR	
CB_013086	0.010695	0.003394	2.049456	up	up	9066	SYT7	
CB_011347	0.010694	0.003393	2.00155	up	up	4113	MAGEB2	
CB_025070	0.01056	0.003335	2.722806	up	up	113730	KLHDC7B	
CB_007844	0.010511	0.003312	2.023214	up	up	8710	SERPINB7	
CB_023161	0.01044	0.003281	3.627453	up	up	53940	FTHL17	
CB_018193	0.010124	0.003149	2.243159	up	up	26059	ERC2	
CB_010803	0.010118	0.003147	3.398958	up	up	1114	CHGB	
CB_019038	0.010024	0.003105	3.098864	up	up	3229	HOXC13	
CB_015387	0.009991	0.003092	2.441504	up	up	8190	MIA	
CB_016816	0.009699	0.002967	2.60458	up	up	29113	C6orf15	
CB_004927	0.009499	0.002887	2.467279	up	up	1289	COL5A1	
CB_010696	0.009498	0.002887	4.700477	up	up	646	BNC1	
CB_028768	0.009374	0.002834	2.697085	up	up	166824	RASSF6	
CB_026348	0.009215	0.002766	4.537425	up	up	154664	ABCA13	
CB_017998	0.009196	0.002758	2.037728	up	up	23509	POFUT1	
CB_015218	0.009063	0.002701	3.525498	up	up	10481	HOXB13	
CB_014286	0.009009	0.00268	2.522035	up	up	7516	XRCC2	
CB_014221	0.008947	0.002655	3.501537	up	up	4108	MAGEA9	
CB_025452	0.008868	0.002623	2.14356	up	up	131096	KCNH8	
CB_013104	0.008574	0.002506	3.718411	up	up	9248	GPR50	
CB_009982	0.008485	0.002469	2.299498	up	up	579	NKX3-2	
CB_010241	0.008473	0.002463	4.235367	up	up	771	CA12	
CB_015067	0.008425	0.002445	2.244543	up	up	5368	PNOC	
CB_011936	0.007937	0.002252	5.388558	up	up	6278	S100A7	
CB_009143	0.007861	0.002221	2.095565	up	up	5349	FXYD3	
CB_017500	0.0077	0.002155	5.906891	up	up	9911	TMCC2	
CB_025903	0.007446	0.002054	2.130375	up	up	253650	ANKRD18A	
CB_012154	0.007429	0.002046	3.424916	up	up	6861	SYT5	
CB_014533	0.007419	0.002042	2.780941	up	up	7681	MKRN3	

CB_023673	0.007404	0.002037	2.484256	up	up	84648	LCE3D	
CB_014993	0.007391	0.002031	2.492691	up	up	4753	NELL2	
CB_005202	0.007378	0.002026	2.437075	up	up	6662	SOX9	
CB_005060	0.007288	0.001993	2.146524	up	up	3795	KHK	
CB_005688	0.006986	0.001879	3.932215	up	up	3358	HTR2C	
CB_005052	0.006829	0.001818	2.252908	up	up	182	JAG1	
CB_021714	0.006776	0.001799	2.033493	up	up	8220	DGCR14	
CB_014384	0.006773	0.001798	2.077555	up	up	3195	TLX1	
CB_027958	0.00671	0.001775	3.228171	up	up	151647	FAM19A4	
CB_018154	0.006565	0.001723	2.0534	up	up	26007	DAK	
CB_022752	0.006467	0.001687	2.313903	up	up	80775	TMEM177	
CB_011310	0.006255	0.001612	2.239736	up	up	4017	LOXL2	
CB_004930	0.00625	0.001609	2.466883	up	up	1311	COMP	
CB_010924	0.006238	0.001606	3.196095	up	up	1917	EEF1A2	
CB_029047	0.006214	0.001597	2.184476	up	up	152519	NIPAL1	
CB_019039	0.00615	0.001573	2.07514	up	up	7976	FZD3	
CB_011399	0.005967	0.001509	5.460282	up	up	4319	MMP10	
CB_006663	0.005937	0.001497	2.186156	up	up	494143	CHAC2	
CB_013356	0.005837	0.001461	2.05355	up	up	2346	FOLH1	
CB_009439	0.00583	0.001458	2.794436	up	up	134111	UBE2QL1	
CB_015979	0.005531	0.001354	2.391295	up	up	11187	PKP3	
CB_011033	0.005461	0.001331	2.017238	up	up	2730	GCLM	
CB_024117	0.005426	0.001319	2.096326	up	up	2567	GABRG3	
CB_010387	0.00542	0.001316	2.486913	up	up	1775	DNASE1L2	
CB_028596	0.005339	0.00129	2.083857	up	up	374393	FAM111B	
CB_021283	0.005243	0.001261	2.366971	up	up	58157	NGB	
CB_011004	0.00524	0.001259	2.140915	up	up	2444	FRK	
CB_014067	0.005213	0.00125	2.382671	up	up	1237	CCR8	
CB_018818	0.005208	0.001249	2.225025	up	up	26499	PLEK2	
CB_005599	0.005119	0.001219	3.289973	up	up	1594	CYP27B1	
CB_002777	0.005096	0.001211	2.200968	up	up	6518	SLC2A5	
CB_007131	0.005073	0.001202	2.921303	up	up	3776	KCNK2	
CB_015774	0.005027	0.001187	7.422284	up	up	6665	SOX15	
CB_010706	0.004871	0.001136	4.016424	up	up	667	DST	
CB_030684	0.004812	0.001116	2.011946	up	up	84808	C1orf170	
CB_023210	0.00477	0.001102	2.251303	up	up	83881	MIXL1	
CB_016869	0.004659	0.001067	3.172012	up	up	6334	SCN8A	
CB_026750	0.004657	0.001066	2.432757	up	up	200407	CREG2	
CB_021136	0.004601	0.001048	5.593141	up	up	9076	CLDN1	
CB_026663	0.004598	0.001047	2.983283	up	up	266740	MAGEA2B	
CB_018718	0.004562	0.001037	3.056089	up	up	51450	PRRX2	
CB_027032	0.004554	0.001034	2.249494	up	up	203111	C8orf47	
CB_024554	0.004545	0.001031	3.134904	up	up	118430	MUCL1	
CB_026995	0.004457	0.001003	2.033337	up	up	148641	SLC35F3	
CB_021215	0.00439	0.000982	4.265337	up	up	57834	CYP4F11	
CB_009114	0.004381	0.00098	3.08264	up	up	9378	NRXN1	
CB_007237	0.004343	0.000969	3.140213	up	up	388228	SBK1	
CB_021860	0.004328	0.000965	2.112867	up	up	65108	MARCKSL1	
CB_026374	0.004326	0.000964	2.924022	up	up	221336	BEND6	
CB_009382	0.004255	0.000942	2.613847	up	up	341346	C12orf70	
CB_022579	0.004211	0.000927	2.44967	up	up	80139	ZNF703	
CB_012085	0.004206	0.000926	2.227364	up	up	6659	SOX4	
CB_020069	0.004166	0.000913	2.170052	up	up	55894	DEFB103B	
CB_005585	0.004142	0.000905	2.013159	up	up	1559	CYP2C9	
CB_009131	0.0041	0.000893	2.846671	up	up	221806	VWDE	
CB_024541	0.00405	0.000878	2.172504	up	up	7809	BSND	

CB_005625	0.003996	0.000862	3.956737	up	up	2558	GABRA5	
CB_018421	0.003974	0.000856	2.044092	up	up	51087	YBX2	
CB_013133	0.003941	0.000847	2.09978	up	up	9377	COX5A	
CB_007482	0.003925	0.000842	3.693021	up	up	7020	TFAP2A	
CB_025046	0.00388	0.000829	3.354885	up	up	112609	MRAP2	
CB_027576	0.003841	0.000817	2.103577	up	up	353133	LCE1C	
CB_011389	0.003808	0.000808	3.623235	up	up	4283	CXCL9	
CB_021509	0.003719	0.00078	2.478628	up	up	63928	CHP2	
CB_025249	0.003713	0.000778	2.039525	up	up	124540	MSI2	
CB_011424	0.003636	0.000757	2.523443	up	up	4488	MSX2	
CB_022489	0.003547	0.000731	2.634642	up	up	79977	GRHL2	
CB_026548	0.003533	0.000727	2.032786	up	up	148113	CILP2	
CB_007152	0.003523	0.000724	2.353863	up	up	91862	MARVELD3	
CB_016506	0.003448	0.000705	2.799424	up	up	26872	STEAP1	
CB_004997	0.003353	0.000677	2.025769	up	up	2642	GCGR	
CB_027511	0.00335	0.000677	2.056408	up	up	283748	PLA2G4D	
CB_019213	0.003282	0.000657	2.070074	up	up	54802	TRIT1	
CB_007138	0.003281	0.000657	5.152084	up	up	92196	DAPL1	
CB_024718	0.003255	0.00065	2.139011	up	up	129025	ZNF280A	
CB_024136	0.003238	0.000645	3.357519	up	up	94032	CAMK2N2	
CB_023067	0.003237	0.000645	3.830447	up	up	56169	GSDMC	
CB_018345	0.003099	0.000607	2.016834	up	up	51361	HOOK1	
CB_007071	0.00309	0.000604	3.610283	up	up	6705	SPRR2F	
CB_027897	0.003084	0.000602	2.031219	up	up	221527	ZBTB12	
CB_014979	0.003024	0.000587	4.040314	up	up	2810	SFN	
CB_007128	0.002974	0.000573	4.961963	up	up	6701	SPRR2B	
CB_015849	0.002963	0.00057	2.521517	up	up	11077	HSF2BP	
CB_012101	0.002954	0.000567	14.22753	up	up	6699	SPRR1B	
CB_024954	0.002937	0.000563	2.58719	up	up	128817	CSTL1	
CB_024436	0.002936	0.000562	3.096006	up	up	116832	RPL39L	
CB_021706	0.002928	0.00056	3.420342	up	up	3224	HOXC8	
CB_018275	0.002805	0.000528	2.172177	up	up	27112	FAM155B	
CB_007861	0.002801	0.000527	3.648006	up	up	154215	NKAIN2	
CB_010502	0.002783	0.000522	2.379144	up	up	2729	GCLC	
CB_028795	0.002748	0.000513	2.485866	up	up	9333	TGM5	
CB_012105	0.00273	0.000508	2.288559	up	up	6713	SQLE	
CB_007747	0.002707	0.000502	3.491504	up	up	9633	MTL5	
CB_018784	0.00267	0.000493	2.026112	up	up	51213	LUZP4	
CB_010699	0.002658	0.00049	2.273366	up	up	655	BMP7	
CB_022668	0.002608	0.000477	2.054122	up	up	80263	TRIM45	
CB_019954	0.002607	0.000476	2.691598	up	up	55359	STYK1	
CB_014334	0.00259	0.000472	2.10002	up	up	10024	TROAP	
CB_016926	0.002583	0.00047	2.263779	up	up	10331	B3GNT3	
CB_005376	0.002566	0.000466	5.392235	up	up	3239	HOXD13	
CB_026969	0.002556	0.000463	2.377573	up	up	124590	USH1G	
CB_026077	0.002458	0.000438	2.335489	up	up	130367	SGPP2	
CB_016995	0.002447	0.000436	3.970635	up	up	23657	SLC7A11	
CB_015670	0.002381	0.000419	3.823075	up	up	10962	MLLT11	
CB_024413	0.002374	0.000417	2.021178	up	up	115650	TNFRSF13C	
CB_009445	0.002363	0.000414	2.370387	up	up	387882	C12orf75	
CB_022694	0.002352	0.000412	2.972889	up	up	80328	ULBP2	
CB_019597	0.002335	0.000408	5.509296	up	up	55117	SLC6A15	
CB_020307	0.002275	0.000393	3.030094	up	up	54626	HES2	
CB_015378	0.002242	0.000386	2.322282	up	up	7697	ZNF138	
CB_018311	0.002225	0.000382	2.762733	up	up	50512	PODXL2	
CB_013670	0.002222	0.000381	4.669268	up	up	9355	LHX2	

CB_011046	0.002117	0.000357	2.230483	up	up	2784	GNB3	
CB_022212	0.002042	0.00034	4.741743	up	up	79679	VTCN1	
CB_012600	0.002035	0.000338	3.416874	up	up	8538	BARX2	
CB_019289	0.001998	0.000329	2.04397	up	up	54866	PPP1R14D	
CB_020617	0.001995	0.000329	2.986148	up	up	57116	ZNF695	
CB_029099	0.001987	0.000327	2.290224	up	up	388555	IGFL3	
CB_028017	0.001973	0.000324	17.20341	up	up	339967	TMPRSS11A	
CB_022291	0.001964	0.000322	5.155659	up	up	79755	ZNF750	
CB_020653	0.001948	0.000318	3.438088	up	up	57156	TMEM63C	
CB_012188	0.001917	0.000312	2.273971	up	up	7022	TFAP2C	
CB_019544	0.001908	0.000309	2.014841	up	up	55071	C9orf40	
CB_011366	0.001856	0.000298	2.119723	up	up	4185	ADAM11	
CB_028339	0.001829	0.000292	4.289484	up	up	29841	GRHL1	
CB_013825	0.001819	0.00029	5.65957	up	up	1823	DSC1	
CB_022378	0.001801	0.000285	2.136742	up	up	79850	FAM57A	
CB_028445	0.001789	0.000282	2.533076	up	up	286077	FAM83H	
CB_007976	0.001779	0.00028	2.027916	up	up	10592	SMC2	
CB_010571	0.001729	0.00027	3.948065	up	up	3627	CXCL10	
CB_018637	0.0017	0.000263	2.007636	up	up	24150	TP53TG3	
CB_014496	0.001691	0.000262	2.12847	up	up	6608	SMO	
CB_016186	0.00169	0.000261	11.44595	up	up	23650	TRIM29	
CB_015632	0.00167	0.000257	14.02373	up	up	10804	GJB6	
CB_020051	0.001651	0.000253	2.005843	up	up	55502	HES6	
CB_024559	0.001644	0.000251	2.3956	up	up	54058	C21orf58	
CB_004922	0.001635	0.000249	6.481494	up	up	1277	COL1A1	
CB_023755	0.001633	0.000249	2.015793	up	up	84790	TUBA1C	
CB_011126	0.001622	0.000247	2.242082	up	up	3208	HPCA	
CB_014039	0.001547	0.000232	2.741782	up	up	430	ASCL2	
CB_009759	0.001547	0.000232	5.007407	up	up	374897	SBSN	
CB_017916	0.001537	0.000229	2.041464	up	up	23321	TRIM2	
CB_028656	0.001521	0.000226	2.521483	up	up	84063	KIRREL2	
CB_005651	0.001504	0.000223	3.765538	up	up	2906	GRIN2D	
CB_015755	0.001489	0.000219	9.226378	up	up	6317	SERPINB3	
CB_004924	0.001474	0.000216	4.278125	up	up	1281	COL3A1	
CB_024358	0.001474	0.000216	7.160267	up	up	114771	PGLYRP3	
CB_028422	0.001462	0.000214	3.259824	up	up	200844	C3orf67	
CB_014828	0.001448	0.000211	11.56726	up	up	6700	SPRR2A	
CB_028609	0.001444	0.00021	2.37568	up	up	284252	KCTD1	
CB_025857	0.001442	0.000209	2.154016	up	up	64843	ISL2	
CB_019238	0.001437	0.000208	2.290784	up	up	54820	NDE1	
CB_025058	0.001432	0.000207	2.18657	up	up	113115	FAM54A	
CB_027952	0.001387	0.000198	2.024585	up	up	148206	ZNF714	
CB_023412	0.001334	0.000188	2.455221	up	up	84197	SGK196	
CB_022459	0.001319	0.000185	2.04484	up	up	79944	L2HGDH	
CB_018054	0.001316	0.000185	3.150572	up	up	25878	MXRA5	
CB_024754	0.001315	0.000184	3.320163	up	up	8840	WISP1	
CB_013381	0.001306	0.000182	2.180978	up	up	3217	HOXB7	
CB_011395	0.001294	0.00018	6.547558	up	up	4312	MMP1	
CB_006660	0.001269	0.000175	2.052021	up	up	22859	LPHN1	
CB_026345	0.001261	0.000174	7.463574	up	up	204962	SLC44A5	
CB_014421	0.001236	0.000169	7.112713	up	up	3868	KRT16	
CB_010909	0.001231	0.000168	2.788956	up	up	1829	DSG2	
CB_022095	0.001218	0.000166	8.951427	up	up	1825	DSC3	
CB_008082	0.001216	0.000165	2.029783	up	up	440145	MZT1	
CB_021404	0.001203	0.000163	4.23492	up	up	2700	GJA3	
CB_013141	0.001202	0.000162	6.244227	up	up	9407	TMPRSS11D	

CB_013480	0.001197	0.000162	3.452068	up	up	6695	SPOCK1	
CB_029098	0.001188	0.00016	6.088369	up	up	388533	KRTDAP	
CB_027691	0.001186	0.000159	4.169807	up	up	126433	FBXO27	
CB_026013	0.001185	0.000159	2.786211	up	up	121273	C12orf54	
CB_023012	0.001179	0.000158	2.979999	up	up	56154	TEX15	
CB_011054	0.001174	0.000157	9.557288	up	up	2877	GPX2	
CB_013846	0.001162	0.000155	6.648711	up	up	3381	IBSP	
CB_026541	0.001114	0.000147	2.710459	up	up	128272	ARHGEF19	
CB_014919	0.001064	0.000138	2.038415	up	up	10344	CCL26	
CB_012084	0.001028	0.000132	11.77397	up	up	6657	SOX2	
CB_022772	0.001019	0.00013	2.053392	up	up	89874	SLC25A21	
CB_025812	0.001009	0.000128	3.642971	up	up	149708	WFDC5	
CB_026920	0.001005	0.000127	19.43488	up	up	286887	KRT6C	
CB_005320	0.000996	0.000126	4.998002	up	up	7291	TWIST1	
CB_008488	0.000969	0.000121	2.496643	up	up	202915	TMEM184A	
CB_015430	0.000968	0.000121	3.001468	up	up	10686	CLDN16	
CB_019754	0.000964	0.00012	2.28499	up	up	55224	ETNK2	
CB_029362	0.000954	0.000119	2.99233	up	up	348825	TPRXL	
CB_007545	0.000941	0.000116	2.143891	up	up	4839	NOP2	
CB_016357	0.000927	0.000114	2.072596	up	up	3751	KCND2	
CB_025909	0.000925	0.000114	2.707799	up	up	59341	TRPV4	
CB_028103	0.000922	0.000113	2.125509	up	up	9071	CLDN10	
CB_022449	0.000919	0.000113	2.559386	up	up	79929	MAP6D1	
CB_012998	0.000918	0.000113	2.156521	up	up	2016	EMX1	
CB_024415	0.000913	0.000112	5.46502	up	up	115701	ALPK2	
CB_014056	0.000903	0.00011	2.621223	up	up	84733	CBX2	
CB_007956	0.00089	0.000108	3.022852	up	up	122786	FRMD6	
CB_014418	0.000874	0.000105	21.21078	up	up	3853	KRT6A	
CB_006416	0.000854	0.000102	3.260957	up	up	205	AK4	
CB_010439	0.000851	0.000101	3.699829	up	up	2019	EN1	
CB_005013	0.000832	9.8E-05	2.000328	up	up	2821	GPI	
CB_027559	0.000825	9.71E-05	3.065294	up	up	347741	OTOP3	
CB_022141	0.000798	9.3E-05	2.604516	up	up	79605	PGBD5	
CB_015539	0.000744	8.46E-05	6.074028	up	up	10874	NMU	
CB_007089	0.000726	8.2E-05	2.215911	up	up	388581	FAM132A	
CB_012032	0.000716	8.05E-05	2.174732	up	up	6541	SLC7A1	
CB_026752	0.000712	7.98E-05	2.606405	up	up	221393	GPR115	
CB_019109	0.000711	7.98E-05	3.139544	up	up	54742	LY6K	
CB_030174	0.000711	7.96E-05	2.379314	up	up	54677	CROT	
CB_021185	0.000697	7.74E-05	2.241163	up	up	23426	GRIP1	
CB_010659	0.000661	7.19E-05	2.134602	up	up	440	ASNS	
CB_010460	0.000655	7.1E-05	5.467364	up	up	2196	FAT2	
CB_014423	0.00065	7.02E-05	3.002736	up	up	284217	LAMA1	
CB_010317	0.000648	6.99E-05	2.143981	up	up	1325	CORT	
CB_011388	0.00064	6.87E-05	2.110675	up	up	4282	MIF	
CB_019243	0.000637	6.83E-05	4.88559	up	up	55612	FERMT1	
CB_016952	0.000621	6.6E-05	2.821979	up	up	23413	NCS1	
CB_014572	0.000617	6.54E-05	2.26101	up	up	10083	USH1C	
CB_019793	0.000597	6.26E-05	3.416473	up	up	55247	NEIL3	
CB_005266	0.000584	6.08E-05	2.084314	up	up	3292	HSD17B1	
CB_020616	0.000577	5.97E-05	7.038368	up	up	57115	PGLYRP4	
CB_017642	0.000572	5.9E-05	2.082392	up	up	22995	CEP152	
CB_014983	0.000558	5.7E-05	4.238287	up	up	3664	IRF6	
CB_008433	0.000558	5.7E-05	2.010406	up	up	284403	WDR62	
CB_025437	0.000555	5.65E-05	2.30836	up	up	125965	COX6B2	
CB_017016	0.000555	5.65E-05	3.159961	up	up	25830	SULT4A1	

CB_015677	0.000549	5.57E-05	2.195177	up	up	10970	CKAP4	
CB_022907	0.000537	5.41E-05	3.245684	up	up	81607	PVRL4	
CB_007045	0.000531	5.33E-05	2.658847	up	up	55620	STAP2	
CB_010775	0.000509	5.04E-05	4.972289	up	up	1001	CDH3	
CB_013339	0.000507	5.01E-05	3.070964	up	up	2191	FAP	
CB_022237	0.000494	4.84E-05	2.13525	up	up	79703	C11orf80	
CB_011132	0.000486	4.72E-05	2.555183	up	up	3236	HOXD10	
CB_006189	0.000476	4.6E-05	3.164249	up	up	441478	NRARP	
CB_016799	0.000467	4.49E-05	2.154733	up	up	28978	TMEM14A	
CB_007191	0.000446	4.21E-05	3.11147	up	up	121551	BTBD11	
CB_027459	0.000441	4.14E-05	2.202991	up	up	165215	FAM171B	
CB_005597	0.000439	4.12E-05	8.408896	up	up	1591	CYP24A1	
CB_015279	0.00043	4.02E-05	17.56415	up	up	10563	CXCL13	
CB_011616	0.000427	3.98E-05	17.15952	up	up	5268	SERPINB5	
CB_015948	0.000421	3.9E-05	3.282936	up	up	7784	ZP3	
CB_019048	0.000393	3.54E-05	2.093362	up	up	51805	COQ3	
CB_022496	0.000378	3.35E-05	5.156399	up	up	79983	POF1B	
CB_028088	0.000375	3.32E-05	2.057241	up	up	4176	MCM7	
CB_012856	0.000374	3.3E-05	2.962699	up	up	8936	WASF1	
CB_021298	0.000367	3.22E-05	4.396404	up	up	59336	PRDM13	
CB_022533	0.000362	3.16E-05	3.102665	up	up	80032	ZNF556	
CB_010983	0.000359	3.13E-05	2.01512	up	up	2288	FKBP4	
CB_017858	0.000359	3.12E-05	2.662708	up	up	23251	KIAA1024	
CB_010910	0.000347	2.98E-05	23.05961	up	up	1830	DSG3	
CB_014980	0.000346	2.97E-05	5.209648	up	up	2842	GPR19	
CB_011661	0.000345	2.96E-05	2.302394	up	up	5453	POU3F1	
CB_026136	0.000341	2.91E-05	2.704028	up	up	1663	DDX11	
CB_014295	0.00034	2.9E-05	3.130776	up	up	8208	CHAF1B	
CB_019037	0.000329	2.77E-05	5.27918	up	up	3226	HOXC10	
CB_025967	0.000328	2.75E-05	2.167795	up	up	91442	C19orf40	
CB_018343	0.000319	2.65E-05	4.259769	up	up	51050	PI15	
CB_025929	0.000319	2.65E-05	3.167492	up	up	121214	SDR9C7	
CB_017019	0.000317	2.63E-05	2.73217	up	up	25837	RAB26	
CB_006935	0.000315	2.61E-05	5.890128	up	up	494514	C18orf56	
CB_022342	0.000307	2.52E-05	2.531622	up	up	79814	AGMAT	
CB_013011	0.000306	2.52E-05	4.518994	up	up	2256	FGF11	
CB_026196	0.000296	2.4E-05	2.760316	up	up	150350	ENTHD1	
CB_016201	0.000291	2.35E-05	3.548096	up	up	23624	CBLC	
CB_021748	0.000287	2.31E-05	3.90267	up	up	94081	SFXN1	
CB_018351	0.000278	2.22E-05	2.591628	up	up	51053	GMNN	
CB_008551	0.000276	2.2E-05	3.311131	up	up	729475	RAD51AP2	
CB_026412	0.000276	2.19E-05	2.837874	up	up	255758	TCTEX1D2	
CB_021284	0.000275	2.18E-05	2.537315	up	up	58985	IL22RA1	
CB_016722	0.000273	2.15E-05	2.381391	up	up	29967	LRP12	
CB_017063	0.00027	2.12E-05	7.799833	up	up	27076	LYPD3	
CB_024543	0.000268	2.1E-05	2.939217	up	up	9134	CCNE2	
CB_028427	0.000256	1.97E-05	2.089835	up	up	253714	MMS22L	
CB_020313	0.00025	1.91E-05	2.310496	up	up	54714	CNGB3	
CB_014437	0.000249	1.91E-05	4.108602	up	up	4001	LMNB1	
CB_019020	0.000248	1.9E-05	6.878291	up	up	10736	SIX2	
CB_017177	0.000243	1.84E-05	4.113097	up	up	5013	OTX1	
CB_024102	0.00024	1.82E-05	2.82572	up	up	90226	UCN2	
CB_006156	0.000239	1.8E-05	4.070187	up	up	339184	CCDC144NL	
CB_015313	0.000229	1.7E-05	2.036822	up	up	10609	LEPREL4	
CB_013352	0.000228	1.69E-05	9.694449	up	up	2304	FOXE1	
CB_013937	0.000215	1.56E-05	2.470833	up	up	5881	RAC3	

CB_023730	0.000206	1.48E-05	2.336957	up	up	6502	SKP2	
CB_018411	0.000203	1.46E-05	2.570513	up	up	51083	GAL	
CB_023608	0.000203	1.45E-05	4.038159	up	up	84518	CNFN	
CB_027620	0.0002	1.42E-05	5.432015	up	up	159963	SLC5A12	
CB_027024	0.000198	1.41E-05	2.046177	up	up	170393	C10orf91	
CB_012066	0.000197	1.39E-05	2.067217	up	up	6627	SNRPA1	
CB_012535	0.000195	1.38E-05	2.070078	up	up	8458	TTF2	
CB_024167	0.000195	1.38E-05	4.953746	up	up	4241	MFI2	
CB_005356	0.000195	1.37E-05	2.88747	up	up	2161	F12	
CB_015998	0.000195	1.37E-05	2.151069	up	up	11222	MRPL3	
CB_024368	0.000194	1.36E-05	2.352809	up	up	114787	GPRIN1	
CB_013439	0.000192	1.34E-05	4.085477	up	up	5017	OVOL1	
CB_021938	0.000187	1.3E-05	10.52641	up	up	2707	GJB3	
CB_023778	0.000179	1.23E-05	3.215618	up	up	84842	HPDL	
CB_010559	0.000177	1.21E-05	3.156921	up	up	3547	IGSF1	
CB_009827	0.000174	1.18E-05	6.974135	up	up	115749	C12orf56	
CB_024881	0.000165	1.1E-05	3.623907	up	up	84706	GPT2	
CB_015307	0.000164	1.09E-05	2.141464	up	up	10605	PAIP1	
CB_015941	0.00016	1.06E-05	2.139748	up	up	7705	ZNF146	
CB_012024	0.00016	1.05E-05	2.56175	up	up	6509	SLC1A4	
CB_027873	0.000157	1.03E-05	2.009636	up	up	160418	TMTC3	
CB_025771	0.000153	1E-05	2.017832	up	up	153328	SLC25A48	
CB_015388	0.00015	9.75E-06	18.01953	up	up	9635	CLCA2	
CB_009011	0.00015	9.71E-06	2.84857	up	up	1E+08	C17orf96	
CB_005278	0.000147	9.51E-06	25.15807	up	up	3852	KRT5	
CB_012208	0.000145	9.29E-06	3.353356	up	up	7058	THBS2	
CB_014494	0.000143	9.11E-06	5.422735	up	up	6535	SLC6A8	
CB_015259	0.000136	8.54E-06	2.352844	up	up	10535	RNASEH2A	
CB_014419	0.000131	8.17E-06	20.45981	up	up	3854	KRT6B	
CB_027492	0.000128	7.94E-06	2.373302	up	up	347688	TUBB8	
CB_012053	0.000127	7.86E-06	2.045967	up	up	6598	SMARCB1	
CB_005632	0.000127	7.84E-06	2.027735	up	up	2618	GART	
CB_011201	0.000127	7.81E-06	2.226106	up	up	3706	ITPKA	
CB_013224	0.000125	7.65E-06	2.078898	up	up	790	CAD	
CB_020561	0.000125	7.65E-06	3.624604	up	up	57216	VANGL2	
CB_027482	0.000124	7.59E-06	2.494321	up	up	91683	SYT12	
CB_025669	0.000121	7.33E-06	3.602668	up	up	221150	SKA3	
CB_005232	0.000117	7.04E-06	3.18746	up	up	7498	XDH	
CB_012809	0.000116	6.93E-06	2.882103	up	up	8851	CDK5R1	
CB_022564	0.000115	6.85E-06	3.541802	up	up	80119	PIF1	
CB_016092	0.000112	6.59E-06	2.529071	up	up	3835	KIF22	
CB_028822	0.000109	6.35E-06	2.18059	up	up	622	BDH1	
CB_014088	0.000107	6.28E-06	9.026941	up	up	1749	DLX5	
CB_020459	0.000107	6.27E-06	2.176838	up	up	56915	EXOSC5	
CB_005309	0.000101	5.76E-06	2.267843	up	up	6528	SLC5A5	
CB_027553	0.0001	5.74E-06	4.605669	up	up	128239	IQGAP3	
CB_017009	0.0001	5.73E-06	2.957592	up	up	24147	FJX1	
CB_024674	9.38E-05	5.21E-06	3.580589	up	up	91057	CCDC34	
CB_027637	9.38E-05	5.21E-06	2.166438	up	up	284098	PIGW	
CB_027558	9.09E-05	4.98E-06	2.60222	up	up	145864	HAPLN3	
CB_007397	8.7E-05	4.68E-06	5.038149	up	up	196051	PPAPDC1A	
CB_005605	8.69E-05	4.67E-06	2.56088	up	up	1719	DHFR	
CB_028366	8.32E-05	4.39E-06	3.336773	up	up	4810	NHS	
CB_023881	7.92E-05	4.08E-06	3.29021	up	up	84951	TNS4	
CB_015845	7.59E-05	3.84E-06	2.107771	up	up	11073	TOPBP1	
CB_025037	7.54E-05	3.81E-06	2.361596	up	up	92935	MARS2	

CB_005375	7.47E-05	3.77E-06	8.388339	up	up	3209	HOXA13	
CB_013566	7.29E-05	3.63E-06	26.23999	up	up	9119	KRT75	
CB_012467	7.19E-05	3.56E-06	2.143417	up	up	8349	HIST2H2BE	
CB_017902	7.13E-05	3.51E-06	2.297006	up	up	23306	TMEM194A	
CB_009029	7.13E-05	3.51E-06	3.088656	up	up	199953	TMEM201	
CB_016717	7.13E-05	3.51E-06	3.325726	up	up	4796	TONSL	
CB_012424	7.1E-05	3.48E-06	4.482373	up	up	8140	SLC7A5	
CB_020059	7.1E-05	3.48E-06	4.476389	up	up	4139	MARK1	
CB_006765	6.86E-05	3.32E-06	23.95928	up	up	222584	FAM83B	
CB_012441	6.86E-05	3.32E-06	2.898152	up	up	8317	CDC7	
CB_007145	6.85E-05	3.31E-06	2.386887	up	up	131076	CCDC58	
CB_028845	6.85E-05	3.31E-06	2.914774	up	up	144455	E2F7	
CB_020114	6.7E-05	3.22E-06	2.205767	up	up	55920	RCC2	
CB_006057	6.66E-05	3.18E-06	9.825754	up	up	147920	IGFL2	
CB_023238	6.54E-05	3.1E-06	4.948485	up	up	83903	GSG2	
CB_021367	6.49E-05	3.07E-06	2.552322	up	up	56652	C10orf2	
CB_025807	6.47E-05	3.05E-06	2.08521	up	up	93594	WDR67	
CB_013434	6.11E-05	2.82E-06	2.602563	up	up	4902	NRTN	
CB_013867	6.09E-05	2.8E-06	10.45034	up	up	4100	MAGEA1	
CB_025525	6.08E-05	2.79E-06	2.731674	up	up	150696	PROM2	
CB_013913	5.83E-05	2.62E-06	3.34015	up	up	5347	PLK1	
CB_015030	5.78E-05	2.59E-06	5.694562	up	up	5083	PAX9	
CB_023501	5.36E-05	2.32E-06	2.852026	up	up	84306	PDCD2L	
CB_012474	5.28E-05	2.26E-06	6.732579	up	up	8356	HIST1H3J	
CB_024716	5.17E-05	2.19E-06	2.718371	up	up	128178	EDARADD	
CB_017509	5.11E-05	2.15E-06	2.671495	up	up	9918	NCAPD2	
CB_012075	4.86E-05	1.98E-06	2.067868	up	up	6636	SNRPF	
CB_028980	4.83E-05	1.97E-06	4.537227	up	up	389336	C5orf46	
CB_010336	4.78E-05	1.93E-06	17.87066	up	up	1470	CST2	
CB_008088	4.68E-05	1.87E-06	2.589749	up	up	7366	UGT2B15	
CB_010345	4.68E-05	1.87E-06	7.941172	up	up	1515	CTSL2	
CB_012420	4.58E-05	1.81E-06	5.015555	up	up	8091	HMGA2	
CB_016015	4.43E-05	1.73E-06	7.380029	up	up	11247	NXPH4	
CB_024268	4.28E-05	1.66E-06	2.197506	up	up	91433	RCCD1	
CB_025622	4.23E-05	1.63E-06	2.624367	up	up	219844	HYLS1	
CB_020261	4.21E-05	1.62E-06	2.45542	up	up	54517	PUS7	
CB_024460	3.72E-05	1.35E-06	4.065222	up	up	5217	PFN2	
CB_011629	3.64E-05	1.3E-06	24.97164	up	up	5307	PITX1	
CB_026175	3.36E-05	1.16E-06	2.421344	up	up	148304	C1orf74	
CB_019422	3.14E-05	1.06E-06	2.336281	up	up	54962	TIPIN	
CB_016754	3.14E-05	1.05E-06	2.766303	up	up	1734	DIO2	
CB_012235	3.12E-05	1.04E-06	2.694381	up	up	7112	TMPO	
CB_011919	3.11E-05	1.04E-06	2.200725	up	up	6182	MRPL12	
CB_013909	2.96E-05	9.72E-07	2.002528	up	up	5296	PIK3R2	
CB_017334	2.91E-05	9.51E-07	2.752867	up	up	9735	KNTC1	
CB_024931	2.8E-05	9.02E-07	6.26073	up	up	171177	RHOV	
CB_019662	2.76E-05	8.87E-07	2.297816	up	up	55159	RFWD3	
CB_020757	2.66E-05	8.45E-07	4.608538	up	up	57405	SPC25	
CB_012995	2.58E-05	8.07E-07	2.267966	up	up	1978	EIF4EBP1	
CB_021840	2.57E-05	8.03E-07	3.576848	up	up	64946	CENPH	
CB_021502	2.55E-05	7.95E-07	2.865227	up	up	63922	CHTF18	
CB_005481	2.32E-05	6.96E-07	4.774446	up	up	3589	IL11	
CB_019516	2.28E-05	6.79E-07	3.949524	up	up	55038	CDCA4	
CB_008805	2.24E-05	6.62E-07	5.744041	up	up	55635	DEPDC1	
CB_011585	2.23E-05	6.58E-07	3.523224	up	up	5163	PDK1	
CB_026698	2.17E-05	6.29E-07	3.052826	up	up	195828	ZNF367	

CB_021902	2.17E-05	6.29E-07	6.690678	up	up	53836	GPR87	
CB_019986	2.16E-05	6.22E-07	3.686208	up	up	55839	CENPN	
CB_016676	2.16E-05	6.21E-07	5.779434	up	up	26585	GREM1	
CB_020058	2.09E-05	5.94E-07	3.516595	up	up	55506	H2AFY2	
CB_022492	1.92E-05	5.31E-07	2.670595	up	up	79980	DSN1	
CB_024158	1.88E-05	5.14E-07	3.855909	up	up	7804	LRP8	
CB_019694	1.88E-05	5.12E-07	3.007658	up	up	55723	ASF1B	
CB_022605	1.79E-05	4.8E-07	2.311769	up	up	80178	C16orf59	
CB_027283	1.75E-05	4.66E-07	2.834993	up	up	128312	HIST3H2BB	
CB_017410	1.72E-05	4.54E-07	2.110681	up	up	9816	URB2	
CB_021305	1.72E-05	4.54E-07	3.729505	up	up	59344	ALOXE3	
CB_010202	1.69E-05	4.38E-07	2.630066	up	up	79172	CENPO	
CB_019898	1.61E-05	4.1E-07	6.956323	up	up	55789	DEPDC1B	
CB_017381	1.57E-05	3.96E-07	4.34346	up	up	9787	DLGAP5	
CB_007130	1.57E-05	3.95E-07	9.223935	up	up	157570	ESCO2	
CB_022522	1.56E-05	3.9E-07	2.05106	up	up	80018	NAA25	
CB_023120	1.52E-05	3.76E-07	6.722976	up	up	83715	ESPN	
CB_020620	1.52E-05	3.74E-07	2.216449	up	up	10017	BCL2L10	
CB_027647	1.5E-05	3.67E-07	6.465289	up	up	338707	B4GALNT4	
CB_014181	1.5E-05	3.66E-07	2.43189	up	up	3006	HIST1H1C	
CB_014821	1.47E-05	3.58E-07	7.619451	up	up	6495	SIX1	
CB_014338	1.47E-05	3.56E-07	2.36193	up	up	10036	CHAF1A	
CB_016366	1.35E-05	3.14E-07	3.355905	up	up	9700	ESPL1	
CB_006726	1.34E-05	3.1E-07	2.181593	up	up	26147	PHF19	
CB_014135	1.31E-05	3E-07	25.47843	up	up	2709	GJB5	
CB_023432	1.3E-05	2.94E-07	2.054446	up	up	84231	TRAF7	
CB_022994	1.29E-05	2.93E-07	4.49094	up	up	81930	KIF18A	
CB_012486	1.28E-05	2.89E-07	2.274339	up	up	8365	HIST1H4H	
CB_012076	1.27E-05	2.86E-07	2.189718	up	up	6637	SNRPG	
CB_021456	1.21E-05	2.67E-07	2.771146	up	up	27085	MTBP	
CB_009781	1.16E-05	2.51E-07	2.720401	up	up	6566	SLC16A1	
CB_022329	1.1E-05	2.34E-07	4.399262	up	up	79801	SHCBP1	
CB_012801	1.05E-05	2.17E-07	2.481523	up	up	8833	GMPS	
CB_007557	1.03E-05	2.09E-07	4.44416	up	up	6241	RRM2	
CB_011654	1.02E-05	2.07E-07	2.620968	up	up	5427	POLE2	
CB_027471	9.56E-06	1.91E-07	4.428609	up	up	8612	PPAP2C	
CB_012917	8.84E-06	1.7E-07	2.441238	up	up	8424	BBOX1	
CB_025147	8.56E-06	1.63E-07	2.50134	up	up	26472	PPP1R14B	
CB_011883	8.25E-06	1.53E-07	3.335131	up	up	5984	RFC4	
CB_021522	8.14E-06	1.5E-07	6.626257	up	up	63967	CLSPN	
CB_016071	8.12E-06	1.49E-07	3.641139	up	up	11339	OIP5	
CB_011551	7.92E-06	1.44E-07	2.007595	up	up	5050	PAFAH1B3	
CB_019047	7.82E-06	1.4E-07	7.549952	up	up	51804	SIX4	
CB_021264	7.75E-06	1.39E-07	2.718986	up	up	58516	FAM60A	
CB_010126	7.59E-06	1.34E-07	4.76912	up	up	638	BIK	
CB_013119	7.19E-06	1.24E-07	4.050904	up	up	9319	TRIP13	
CB_015320	6.11E-06	9.73E-08	5.842794	up	up	10615	SPAG5	
CB_009272	5.76E-06	8.93E-08	2.722427	up	up	90417	C15orf23	
CB_013574	5.57E-06	8.51E-08	5.338049	up	up	9133	CCNB2	
CB_015584	5.33E-06	7.96E-08	4.267442	up	up	2491	CENPI	
CB_016537	5.23E-06	7.79E-08	5.378816	up	up	3161	HMMR	
CB_012331	4.96E-06	7.17E-08	2.683237	up	up	7443	VRK1	
CB_016403	4.95E-06	7.13E-08	2.165871	up	up	25800	SLC39A6	
CB_023492	4.76E-06	6.71E-08	4.77748	up	up	84296	GINS4	
CB_018080	4.67E-06	6.51E-08	9.715413	up	up	25907	TMEM158	
CB_015771	4.62E-06	6.4E-08	2.142726	up	up	6632	SNRPD1	

CB_012082	4.6E-06	6.34E-08	3.814452	up	up	6652	SORD	
CB_014751	4.56E-06	6.26E-08	2.681293	up	up	4175	MCM6	
CB_025873	4.43E-06	5.99E-08	5.025422	up	up	3159	HMGA1	
CB_023273	4.13E-06	5.4E-08	8.51858	up	up	83990	BRIP1	
CB_019748	4.09E-06	5.32E-08	3.434641	up	up	26586	CKAP2	
CB_012845	4.09E-06	5.31E-08	3.226373	up	up	8914	TIMELESS	
CB_019637	3.79E-06	4.76E-08	5.383125	up	up	1894	ECT2	
CB_025784	3.46E-06	4.16E-08	3.107757	up	up	199731	CADM4	
CB_020019	3.41E-06	4.05E-08	8.780683	up	up	55872	PBK	
CB_012804	3.36E-06	3.95E-08	3.644258	up	up	8836	GGH	
CB_004893	3.28E-06	3.79E-08	3.50403	up	up	641	BLM	
CB_017415	3.28E-06	3.78E-08	4.621106	up	up	9824	ARHGAP11A	
CB_024229	3.28E-06	3.75E-08	3.317228	up	up	92815	HIST3H2A	
CB_014946	3.21E-06	3.62E-08	7.975562	up	up	10403	NDC80	
CB_011390	3.17E-06	3.57E-08	7.856418	up	up	4288	MKI67	
CB_027951	3.15E-06	3.53E-08	7.579261	up	up	147841	SPC24	
CB_017852	3.03E-06	3.28E-08	2.700294	up	up	23246	BOP1	
CB_016825	2.98E-06	3.2E-08	2.962261	up	up	29028	ATAD2	
CB_016856	2.97E-06	3.16E-08	6.33751	up	up	29089	UBE2T	
CB_008167	2.9E-06	3.07E-08	3.216398	up	up	10606	PAICS	
CB_012475	2.86E-06	3.01E-08	4.951139	up	up	8357	HIST1H3H	
CB_019678	2.67E-06	2.73E-08	12.63597	up	up	259266	ASPM	
CB_019732	2.65E-06	2.7E-08	3.323821	up	up	55215	FANCI	
CB_011845	2.64E-06	2.69E-08	2.009393	up	up	5888	RAD51	
CB_024689	2.57E-06	2.58E-08	9.819454	up	up	113130	CDCA5	
CB_023304	2.51E-06	2.47E-08	7.878419	up	up	84057	MND1	
CB_025828	2.41E-06	2.32E-08	7.229286	up	up	83540	NUF2	
CB_012991	2.23E-06	2.05E-08	5.98271	up	up	1870	E2F2	
CB_010254	2.1E-06	1.87E-08	6.674142	up	up	890	CCNA2	
CB_018763	2.09E-06	1.84E-08	6.716917	up	up	51203	NUSAP1	
CB_012452	2.09E-06	1.83E-08	4.460282	up	up	8335	HIST1H2AB	
CB_013403	2.08E-06	1.81E-08	2.818788	up	up	4171	MCM2	
CB_013010	1.87E-06	1.55E-08	4.08397	up	up	2237	FEN1	
CB_006749	1.77E-06	1.42E-08	2.087303	up	up	135293	PM20D2	
CB_017149	1.64E-06	1.23E-08	3.258325	up	up	27338	UBE2S	
CB_019240	1.49E-06	1.08E-08	3.803886	up	up	54821	ERCC6L	
CB_022925	1.39E-06	9.7E-09	6.522971	up	up	81620	CDT1	
CB_022009	1.38E-06	9.39E-09	4.821465	up	up	79075	DSCC1	
CB_023486	1.32E-06	8.77E-09	4.280398	up	up	9718	ECE2	
CB_025118	1.28E-06	8.28E-09	4.921252	up	up	9493	KIF23	
CB_013401	1.23E-06	7.52E-09	5.177374	up	up	3832	KIF11	
CB_011646	1.19E-06	6.93E-09	4.955171	up	up	5367	PMCH	
CB_016391	1.16E-06	6.64E-09	3.230609	up	up	24137	KIF4A	
CB_028714	1.11E-06	5.82E-09	2.994981	up	up	10721	POLQ	
CB_016985	1.08E-06	5.53E-09	6.435249	up	up	23594	ORC6	
CB_011569	1.04E-06	5.27E-09	3.06372	up	up	5111	PCNA	
CB_010255	9.77E-07	4.74E-09	3.510492	up	up	898	CCNE1	
CB_008044	9.68E-07	4.64E-09	7.885154	up	up	6491	STIL	
CB_013220	9.27E-07	4.31E-09	7.047509	up	up	699	BUB1	
CB_013336	9.19E-07	4.24E-09	5.509985	up	up	2146	EZH2	
CB_017520	8.72E-07	3.87E-09	10.18413	up	up	9928	KIF14	
CB_013100	8.04E-07	3.49E-09	5.689572	up	up	9212	AURKB	
CB_018534	7.43E-07	3.02E-09	3.096217	up	up	51659	GINS2	
CB_007716	6.92E-07	2.68E-09	8.036031	up	up	220134	SKA1	
CB_023239	6.54E-07	2.38E-09	7.123287	up	up	891	CCNB1	
CB_016473	6.41E-07	2.29E-09	4.067872	up	up	25788	RAD54B	

CB_012456	6.23E-07	2.12E-09	2.378057	up	up	8339	HIST1H2BG	
CB_015930	5.96E-07	1.88E-09	11.30298	up	up	7546	ZIC2	
CB_012453	4.32E-07	9.38E-10	2.552186	up	up	8336	HIST1H2AM	
CB_006880	4.22E-07	8.84E-10	8.106777	up	up	332	BIRC5	
CB_012477	3.29E-07	5.64E-10	4.873899	up	up	8358	HIST1H3B	
CB_023768	2.99E-07	4.7E-10	4.32682	up	up	84823	LMNB2	
CB_021091	2.77E-07	4.26E-10	2.86301	up	up	3012	HIST1H2AE	
CB_021824	2.75E-07	4.19E-10	9.62036	up	up	53335	BCL11A	
CB_006916	2.57E-07	3.62E-10	4.029962	up	up	387103	CENPW	
CB_026919	1.88E-07	2.21E-10	3.232804	up	up	286827	TRIM59	
CB_018750	1.43E-07	1.52E-10	7.927329	up	up	1063	CENPF	
CB_012460	7.09E-08	3.93E-11	5.1909	up	up	8343	HIST1H2BF	
CB_010744	5.29E-08	1.11E-11	6.233105	up	up	899	CCNF	
CB_007907	0.048649	0.024677	2.189062	up	up	222962	SLC29A4	
CB_019034	0.048515	0.024584	3.008995	up	up	50940	PDE11A	
CB_020631	0.048508	0.024576	2.494927	up	up	57126	CD177	
CB_020091	0.048328	0.024458	2.15827	up	up	57214	KIAA1199	
CB_021407	0.046948	0.02359	2.125306	up	up	2898	GRIK2	
CB_010101	0.04637	0.023229	2.575517	up	up	83416	FCRL5	
CB_028467	0.045619	0.022769	2.423965	up	up	346689	KLRG2	
CB_017733	0.045121	0.022456	2.247964	up	up	23105	FSTL4	
CB_018138	0.044849	0.022288	3.289143	up	up	25984	KRT23	
CB_012738	0.043926	0.021705	2.49329	up	up	8745	ADAM23	
CB_027884	0.041774	0.020358	2.194746	up	up	5570	PKIB	
CB_025225	0.041607	0.020258	2.081123	up	up	130951	C2orf65	
CB_015097	0.041474	0.020173	2.05924	up	up	5544	PRB3	
CB_009767	0.040205	0.01939	2.738561	up	up	57057	TBX20	
CB_014087	0.039361	0.018882	2.00953	up	up	1747	DLX3	
CB_021403	0.034331	0.01588	2.444201	up	up	1996	ELAVL4	
CB_014518	0.03381	0.015565	3.242602	up	up	6999	TDO2	
CB_017481	0.033333	0.015289	3.059576	up	up	9892	SNAP91	
CB_022818	0.032094	0.014545	2.227674	up	up	54361	WNT4	
CB_013763	0.031639	0.014281	2.350817	up	up	9536	PTGES	
CB_023323	0.029119	0.012849	2.324958	up	up	84072	HORMAD1	
CB_010053	0.028634	0.012565	2.083916	up	up	4897	NRCAM	
CB_024666	0.028298	0.012358	2.339525	up	up	6899	TBX1	
CB_010596	0.026914	0.011585	2.106412	up	up	119	ADD2	
CB_008978	0.026781	0.011512	2.082914	up	up	5673	PSG5	
CB_025750	0.025786	0.010966	2.087431	up	up	130399	ACVR1C	
CB_021934	0.025494	0.010804	2.429665	up	up	3897	L1CAM	
CB_013243	0.023259	0.009597	2.246988	up	up	1047	CLGN	
CB_017910	0.023089	0.009508	2.265633	up	up	23314	SATB2	
CB_011225	0.02308	0.009502	2.005742	up	up	3775	KCNK1	
CB_009329	0.022417	0.00913	2.007589	up	up	56474	CTPS2	
CB_010662	0.021298	0.008536	2.846229	up	up	479	ATP12A	
CB_022119	0.020636	0.008187	2.301428	up	up	79570	NKAIN1	
CB_029614	0.019915	0.007807	2.253797	up	up	650655	ABCA17P	
CB_010831	0.01932	0.007499	2.051107	up	up	1299	COL9A3	
CB_002088	0.019243	0.007459	2.04718	up	up	338324	S100A7A	
CB_011263	0.018512	0.00709	3.383653	up	up	3881	KRT31	
CB_021289	0.018373	0.007019	3.120939	up	up	59082	CARD18	
CB_014006	0.018336	0.007	3.618035	up	up	9982	FGFBP1	
CB_005518	0.017803	0.006737	4.734831	up	up	218	ALDH3A1	
CB_013012	0.017318	0.006493	2.571115	up	up	2257	FGF12	
CB_020577	0.017304	0.006486	2.122859	up	up	26507	CNNM1	
CB_013805	0.017041	0.006351	5.513941	up	up	793	CALB1	

CB_000153	0.016971	0.006317	2.482276	up	up	594839	SNORA33	
CB_010291	0.016243	0.005966	2.389568	up	up	1116	CHI3L1	
CB_014163	0.01619	0.005941	2.026926	up	up	2847	MCHR1	
CB_006899	0.015894	0.005797	2.110515	up	up	402381	SOHLH1	
CB_020983	0.015568	0.005638	2.120745	up	up	65267	WNK3	
CB_001000	0.015558	0.005633	2.384037	up	up	873	CBR1	
CB_001121	0.015062	0.005388	2.399225	up	up	5827	PXMP2	
CB_010787	0.014899	0.005312	2.303672	up	up	1044	CDX1	
CB_026215	0.01478	0.005257	3.188732	up	up	152404	IGSF11	
CB_028709	0.014325	0.005041	2.99387	up	up	170692	ADAMTS18	
CB_029990	0.01365	0.004731	2.988081	up	up	80307	FER1L4	
CB_010908	0.013646	0.004729	2.208585	up	up	1828	DSG1	
CB_012339	0.013543	0.004682	2.244521	up	up	7474	WNT5A	
CB_019746	0.012813	0.004331	2.551413	up	up	55220	KLHDC8A	
CB_015400	0.012391	0.00414	2.024895	up	up	10644	IGF2BP2	
CB_021457	0.012287	0.004091	2.647505	up	up	43847	KLK14	
CB_016815	0.011891	0.003911	2.763648	up	up	170680	PSORS1C2	
CB_004923	0.01185	0.003891	2.172032	up	up	1278	COL1A2	
CB_011738	0.01185	0.003891	2.250075	up	up	5655	KLK10	
CB_010002	0.011034	0.003538	2.425603	up	up	2668	GDNF	
CB_009398	0.01091	0.003483	2.849844	up	up	5328	PLAU	
CB_027361	0.010851	0.003458	3.200162	up	up	4105	MAGEA6	
CB_021928	0.010651	0.003375	2.87661	up	up	66004	LYNX1	
CB_011261	0.010607	0.003355	4.602764	up	up	3866	KRT15	
CB_005542	0.010564	0.003337	2.746892	up	up	774	CACNA1B	
CB_015409	0.010401	0.003264	3.940327	up	up	10655	DMRT2	
CB_029110	0.009745	0.002986	3.18797	up	up	389208	TMPRSS11F	
CB_021056	0.00964	0.002943	2.56814	up	up	6326	SCN2A	
CB_021871	0.009368	0.002832	2.196154	up	up	773	CACNA1A	
CB_009140	0.009254	0.002781	2.086646	up	up	57553	MICAL3	
CB_011376	0.009151	0.00274	2.15219	up	up	4232	MEST	
CB_008106	0.009093	0.002713	3.123975	up	up	90161	HS6ST2	
CB_027183	0.009021	0.002686	2.043196	up	up	3557	IL1RN	
CB_003623	0.009016	0.002683	2.247044	up	up	285966	FAM115C	
CB_003594	0.008997	0.002675	2.215912	up	up	2274	FHL2	
CB_020379	0.008996	0.002674	6.165541	up	up	28234	SLCO1B3	
CB_025260	0.008663	0.00254	3.228136	up	up	81832	NETO1	
CB_025319	0.008552	0.002496	2.297479	up	up	199713	NLRP7	
CB_012341	0.008333	0.002406	2.172607	up	up	7480	WNT10B	
CB_025134	0.008159	0.002336	2.050321	up	up	64919	BCL11B	
CB_012034	0.007875	0.002228	3.243694	up	up	6549	SLC9A2	
CB_014504	0.007717	0.002161	2.01393	up	up	6875	TAF4B	
CB_005441	0.007691	0.002151	2.856591	up	up	3485	IGFBP2	
CB_015732	0.007648	0.002132	2.663362	up	up	1087	CEACAM7	
CB_027303	0.007489	0.002073	3.194621	up	up	7499	XG	
CB_013070	0.007457	0.002059	3.717036	up	up	7345	UCHL1	
CB_021045	0.007418	0.002042	2.075002	up	up	30848	CTAG2	
CB_014937	0.00734	0.002012	3.346585	up	up	10389	SCML2	
CB_023301	0.007207	0.001963	4.462192	up	up	63950	DMRTA2	
CB_022483	0.007066	0.001908	2.435577	up	up	79968	WDR76	
CB_012982	0.007027	0.001895	2.605761	up	up	1607	DGKB	
CB_016759	0.006998	0.001883	2.110508	up	up	9641	IKBKE	
CB_021336	0.006998	0.001883	2.581644	up	up	10863	ADAM28	
CB_015120	0.006932	0.001859	3.650441	up	up	6354	CCL7	
CB_028515	0.006909	0.00185	2.536332	up	up	375519	GJB7	
CB_028786	0.006898	0.001846	2.198941	up	up	26470	SEZ6L2	

CB_013177	0.006882	0.001839	2.62509	up	up	9615	GDA	
CB_028356	0.006711	0.001776	2.239838	up	up	8838	WISP3	
CB_003019	0.006653	0.001754	2.088638	up	up	644815	FAM83G	
CB_006842	0.006434	0.001674	2.52926	up	up	4110	MAGEA11	
CB_024572	0.006279	0.001621	3.953885	up	up	1029	CDKN2A	
CB_024246	0.006259	0.001614	2.448337	up	up	90293	KLHL13	
CB_024621	0.006209	0.001595	5.087011	up	up	89777	SERPINB12	
CB_013632	0.006117	0.001562	5.066552	up	up	9245	GCNT3	
CB_010281	0.0061	0.001556	2.238762	up	up	1041	CDSN	
CB_016880	0.006094	0.001554	2.447659	up	up	3227	HOXC11	
CB_014386	0.006049	0.001537	2.091722	up	up	3207	HOXA11	
CB_016882	0.005873	0.001474	2.088849	up	up	3235	HOXD9	
CB_012307	0.00579	0.001443	2.990443	up	up	7368	UGT8	
CB_020281	0.005666	0.001399	3.315866	up	up	54544	CRCT1	
CB_015498	0.005522	0.001351	2.780389	up	up	10799	RPP40	
CB_012332	0.00536	0.001298	3.897112	up	up	7447	VSNL1	
CB_018732	0.005338	0.00129	2.911244	up	up	51458	RHCG	
CB_002861	0.005298	0.001278	2.119977	up	up	54991	C1orf159	
CB_009721	0.005169	0.001236	2.790864	up	up	4703	NEB	
CB_008718	0.005155	0.001231	7.51574	up	up	282973	JAKMIP3	
CB_015777	0.005154	0.001231	4.336811	up	up	6703	SPRR2D	
CB_014233	0.004922	0.001152	2.190045	up	up	4610	MYCL1	
CB_013131	0.004885	0.00114	2.144401	up	up	9368	SLC9A3R1	
CB_027192	0.004883	0.00114	2.289418	up	up	200634	KRTCAP3	
CB_023556	0.004833	0.001123	2.314494	up	up	84419	C15orf48	
CB_013320	0.004821	0.001119	2.732217	up	up	2048	EPHB2	
CB_027948	0.004813	0.001116	4.011859	up	up	144501	KRT80	
CB_028135	0.004774	0.001104	2.315536	up	up	5352	PLOD2	
CB_019468	0.004772	0.001103	2.049411	up	up	55001	TTC22	
CB_015260	0.004756	0.001098	2.809859	up	up	10537	UBD	
CB_020508	0.00458	0.001042	2.279856	up	up	10509	SEMA4B	
CB_011025	0.004552	0.001033	2.912082	up	up	2626	GATA4	
CB_007233	0.00452	0.001023	3.488699	up	up	6704	SPRR2E	
CB_029030	0.004253	0.000941	2.417519	up	up	92558	CCDC64	
CB_011820	0.004244	0.000938	7.152339	up	up	5803	PTPRZ1	
CB_019390	0.00406	0.000882	2.339502	up	up	54937	SOHLH2	
CB_005499	0.003981	0.000858	8.15422	up	up	131	ADH7	
CB_013028	0.003941	0.000847	3.904428	up	up	3174	HNF4G	
CB_025489	0.003902	0.000835	3.466974	up	up	144568	A2ML1	
CB_029086	0.003885	0.00083	4.260316	up	up	387695	C10orf99	
CB_011016	0.003863	0.000824	3.683379	up	up	2569	GABRR1	
CB_010791	0.003748	0.000789	2.045018	up	up	1056	CEL	
CB_018139	0.00365	0.00076	2.072295	up	up	25987	TSKU	
CB_007187	0.003564	0.000736	3.464798	up	up	4915	NTRK2	
CB_008740	0.003469	0.00071	2.014575	up	up	127255	LRRIQ3	
CB_023519	0.003385	0.000686	2.180672	up	up	84321	THOC3	
CB_020195	0.003356	0.000678	2.920262	up	up	54346	UNC93A	
CB_008131	0.00334	0.000674	2.124673	up	up	2778	GNAS	
CB_030793	0.003291	0.00066	6.137615	up	up	400578	KRT16P2	
CB_025900	0.003254	0.00065	2.990318	up	up	127343	DMBX1	
CB_027550	0.003223	0.000641	2.00722	up	up	349565	NMNAT3	
CB_026269	0.003128	0.000614	2.021763	up	up	163081	ZNF567	
CB_009648	0.003111	0.00061	2.980215	up	up	5365	PLXNB3	
CB_005567	0.003087	0.000603	3.011918	up	up	1143	CHRNB4	
CB_017489	0.003056	0.000595	2.300814	up	up	9900	SV2A	
CB_021945	0.003046	0.000592	2.005698	up	up	3219	HOXB9	

CB_015821	0.002981	0.000575	2.119844	up	up	11045	UPK1A		
CB_006888	0.002963	0.00057	2.301173	up	up	4192	MDK		
CB_022744	0.002879	0.000547	2.02955	up	up	80759	KHDC1		
CB_023650	0.002869	0.000545	3.466951	up	up	84624	FNDC1		
CB_020510	0.002855	0.000541	2.004994	up	up	56963	RGMA		
CB_020726	0.002847	0.000539	2.069223	up	up	54165	DCUN1D1		
CB_013247	0.00278	0.000522	2.098086	up	up	1181	CLCN2		
CB_009870	0.002763	0.000516	2.282729	up	up	6530	SLC6A2		
CB_021341	0.002754	0.000514	2.639169	up	up	10761	PLAC1		
CB_025699	0.002747	0.000512	2.199388	up	up	151354	FAM84A		
CB_022952	0.00273	0.000508	2.614739	up	up	81792	ADAMTS12		
CB_026580	0.002672	0.000493	5.198704	up	up	255928	SYT14		
CB_025297	0.00267	0.000493	3.431546	up	up	140766	ADAMTS14		
CB_027737	0.002664	0.000491	4.019875	up	up	116379	IL22RA2		
CB_023485	0.002664	0.000491	3.141049	up	up	84290	CAPNS2		
CB_008123	0.002649	0.000487	2.11474	up	up	51611	DPH5		
CB_023823	0.002639	0.000485	2.165934	up	up	84900	RNFT2		
CB_019208	0.0026	0.000474	2.383857	up	up	55605	KIF21A		
CB_018762	0.002587	0.000471	3.179341	up	up	50805	IRX4		
CB_025414	0.002586	0.000471	2.320049	up	up	118932	ANKRD22		
CB_024953	0.002567	0.000466	2.197468	up	up	1748	DLX4		
CB_011218	0.002556	0.000463	3.873788	up	up	3755	KCNG1		
CB_014422	0.002437	0.000434	3.866446	up	up	3898	LAD1		
CB_024672	0.002435	0.000433	2.027066	up	up	90423	ATP6V1E2		
CB_006478	0.002426	0.000431	2.341437	up	up	11200	CHEK2		
CB_026126	0.002391	0.000422	4.182163	up	up	143503	OR51E1		
CB_008531	0.002379	0.000419	9.3545	up	up	2786	GNG4		
CB_022156	0.002349	0.000411	3.326761	up	up	79623	GALNT14		
CB_005205	0.002255	0.000388	3.613594	up	up	6770	STAR		
CB_012654	0.002235	0.000384	8.59536	up	up	8626	TP63		
CB_023368	0.002173	0.000369	2.020494	up	up	84140	FAM161A		
CB_016756	0.002168	0.000368	2.373152	up	up	780	DDR1		
CB_021288	0.002138	0.000362	9.185091	up	up	56033	BARX1		
CB_019075	0.002081	0.000349	2.901588	up	up	4237	MFAP2		
CB_010174	0.002068	0.000346	3.388483	up	up	163589	TDRD5		
CB_022410	0.002058	0.000344	2.752254	up	up	79883	PODNL1		
CB_011401	0.002025	0.000336	5.006406	up	up	4322	MMP13		
CB_019276	0.002021	0.000335	2.10356	up	up	54857	GDPD2		
CB_016150	0.002018	0.000334	3.624713	up	up	6493	SIM2		
CB_029165	0.001979	0.000325	2.008168	up	up	8702	B4GALT4		
CB_011260	0.001963	0.000322	8.034234	up	up	3860	KRT13		
CB_017291	0.001945	0.000318	6.947606	up	up	9699	RIMS2		
CB_008764	0.00192	0.000313	2.142979	up	up	222183	SRRM3		
CB_025537	0.001908	0.000309	4.383043	up	up	53833	IL20RB		
CB_016158	0.001907	0.000309	2.190589	up	up	22809	ATF5		
CB_025301	0.001897	0.000307	2.831592	up	up	91614	DEPDC7		
CB_006950	0.001887	0.000305	5.14923	up	up	5653	KLK6		
CB_026033	0.001863	0.0003	2.367793	up	up	124222	PAQR4		
CB_028079	0.001845	0.000295	2.724281	up	up	256764	WDR72		
CB_013318	0.001837	0.000294	2.747209	up	up	2047	EPHB1		
CB_026451	0.001832	0.000293	5.651073	up	up	169044	COL22A1		
CB_025500	0.001818	0.000289	2.134571	up	up	147015	DHRS13		
CB_022623	0.001786	0.000282	2.548965	up	up	80206	FHOD3		
CB_005276	0.001732	0.00027	9.829088	up	up	3872	KRT17		
CB_010917	0.001701	0.000264	2.060848	up	up	1875	E2F5		
CB_005623	0.00169	0.000261	2.021809	up	up	2556	GABRA3		

CB_022066	0.001614	0.000245	4.171135	up	up	79173	C19orf57	
CB_022214	0.001597	0.000241	2.414681	up	up	84561	SLC12A8	
CB_020297	0.001584	0.000239	12.44867	up	up	54576	UGT1A8	
CB_028053	0.001569	0.000236	2.117363	up	up	9088	PKMYT1	
CB_026372	0.001553	0.000233	2.02271	up	up	221322	C6orf170	
CB_010239	0.001545	0.000231	6.518695	up	up	768	CA9	
CB_023581	0.001537	0.000229	3.547141	up	up	84466	MEGF10	
CB_011113	0.001533	0.000228	2.095943	up	up	3148	HMGB2	
CB_021484	0.001496	0.000221	2.447459	up	up	112399	EGLN3	
CB_008686	0.00149	0.00022	2.01	up	up	400745	SH2D5	
CB_014089	0.001479	0.000217	5.611977	up	up	1750	DLX6	
CB_013583	0.001462	0.000214	2.036868	up	up	9141	PDCD5	
CB_012821	0.001462	0.000214	2.169685	up	up	8871	SYNJ2	
CB_026981	0.001462	0.000214	2.470109	up	up	134285	TMEM171	
CB_015420	0.001461	0.000213	3.064271	up	up	10669	CGREF1	
CB_015055	0.001456	0.000212	2.984522	up	up	5270	SERPINE2	
CB_022513	0.001432	0.000207	2.184707	up	up	140578	CHODL	
CB_026294	0.001416	0.000204	4.66464	up	up	169166	SNX31	
CB_028603	0.001382	0.000197	7.831069	up	up	5744	PTHLH	
CB_012007	0.00138	0.000197	3.258913	up	up	6447	SCG5	
CB_005586	0.001351	0.000191	2.978968	up	up	1562	CYP2C18	
CB_010121	0.00135	0.000191	2.032171	up	up	130574	LYPD6	
CB_011732	0.001281	0.000178	3.977604	up	up	5644	PRSS1	
CB_006007	0.001277	0.000177	2.031197	up	up	51155	HN1	
CB_013043	0.001265	0.000175	2.223574	up	up	4998	ORC1	
CB_020428	0.001264	0.000174	2.03821	up	up	7286	TUFT1	
CB_021405	0.001248	0.000171	2.626435	up	up	2792	GNGT1	
CB_005243	0.001241	0.00017	3.749668	up	up	1290	COL5A2	
CB_023681	0.001204	0.000163	3.30419	up	up	84659	RNASE7	
CB_011345	0.001185	0.000159	5.572053	up	up	4103	MAGEA4	
CB_015483	0.001183	0.000159	2.862462	up	up	26227	PHGDH	
CB_021090	0.001158	0.000154	2.911811	up	up	4104	MAGEA5	
CB_010745	0.001124	0.000149	2.263801	up	up	908	CCT6A	
CB_008181	0.001116	0.000147	2.795845	up	up	2118	ETV4	
CB_028419	0.001102	0.000144	9.782871	up	up	163351	GBP6	
CB_023265	0.001072	0.000139	2.111975	up	up	83959	SLC4A11	
CB_026323	0.001072	0.000139	2.535241	up	up	55103	RALGPS2	
CB_025389	0.001052	0.000136	2.199722	up	up	90861	HN1L	
CB_010028	0.001047	0.000135	3.850845	up	up	150946	FAM59B	
CB_008363	0.000992	0.000125	8.080276	up	up	441282	AKR1B15	
CB_006198	0.000986	0.000124	2.225487	up	up	22801	ITGA11	
CB_014986	0.000955	0.000119	2.148405	up	up	4007	PRICKLE3	
CB_015906	0.000944	0.000117	2.393857	up	up	6941	TCF19	
CB_009242	0.00094	0.000116	2.308775	up	up	26271	FBXO5	
CB_007730	0.000939	0.000116	5.261442	up	up	139728	PNCK	
CB_016211	0.000921	0.000113	4.837358	up	up	22802	CLCA4	
CB_010854	0.000908	0.000111	3.764321	up	up	1382	CRABP2	
CB_020662	0.000904	0.00011	2.731493	up	up	5738	PTGFRN	
CB_019939	0.0009	0.000109	2.15746	up	up	55353	LAPTM4B	
CB_007965	0.000899	0.000109	2.491434	up	up	81624	DIAPH3	
CB_019265	0.000884	0.000107	2.575933	up	up	54845	ESRP1	
CB_011949	0.000858	0.000102	11.87879	up	up	6318	SERPINB4	
CB_010966	0.000846	0.0001	6.006647	up	up	2201	FBN2	
CB_005362	0.000846	0.0001	2.57604	up	up	2524	FUT2	
CB_016832	0.000846	0.0001	7.983875	up	up	26047	CNTNAP2	
CB_027483	0.000842	9.97E-05	4.668083	up	up	130576	LYPD6B	

CB_007766	0.000841	9.94E-05	2.451059	up	up	400954	EML6	
CB_014588	0.000799	9.32E-05	2.641001	up	up	10105	PPIF	
CB_009301	0.000799	9.31E-05	2.511338	up	up	50507	NOX4	
CB_002250	0.000798	9.3E-05	4.665882	up	up	65268	WNK2	
CB_015335	0.000796	9.26E-05	3.303857	up	up	10631	POSTN	
CB_006841	0.000795	9.25E-05	3.931972	up	up	4109	MAGEA10	
CB_021232	0.00079	9.17E-05	2.077362	up	up	58477	SRPRB	
CB_019555	0.000783	9.07E-05	2.85102	up	up	55083	KIF26B	
CB_004034	0.000776	8.97E-05	2.235576	up	up	80174	DBF4B	
CB_020657	0.000776	8.96E-05	2.658751	up	up	57167	SALL4	
CB_013952	0.000771	8.89E-05	2.395323	up	up	6508	SLC4A3	
CB_025451	0.000765	8.8E-05	2.017376	up	up	130827	TMEM182	
CB_008084	0.000763	8.76E-05	11.54609	up	up	54578	UGT1A6	
CB_010418	0.000763	8.75E-05	3.032324	up	up	1952	CELSR2	
CB_011734	0.000758	8.67E-05	2.738497	up	up	5646	PRSS3	
CB_023095	0.000732	8.3E-05	2.426133	up	up	83639	TEX101	
CB_012633	0.000727	8.21E-05	14.37974	up	up	8581	LY6D	
CB_014016	0.000718	8.07E-05	3.163094	up	up	55806	HR	
CB_005992	0.000714	8.02E-05	3.333408	up	up	513	ATP5D	
CB_021128	0.000712	7.99E-05	2.133604	up	up	8884	SLC5A6	
CB_005218	0.000701	7.79E-05	2.162597	up	up	7167	TPI1	
CB_020377	0.000694	7.7E-05	2.534553	up	up	56479	KCNQ5	
CB_012461	0.000658	7.14E-05	3.908282	up	up	8344	HIST1H2BE	
CB_029169	0.000613	6.48E-05	2.26891	up	up	388962	BOLA3	
CB_011196	0.000574	5.93E-05	3.923	up	up	3696	ITGB8	
CB_011364	0.00057	5.88E-05	2.091538	up	up	4172	MCM3	
CB_019846	0.000557	5.68E-05	3.614275	up	up	55287	TMEM40	
CB_012576	0.00053	5.31E-05	2.501154	up	up	8508	NIPSNAP1	
CB_018821	0.000524	5.23E-05	2.679779	up	up	51514	DTL	
CB_013535	0.00052	5.19E-05	2.208024	up	up	8437	RASAL1	
CB_007753	0.000507	5.01E-05	2.157118	up	up	55898	UNC45A	
CB_020018	0.000476	4.6E-05	2.521624	up	up	55366	LGR4	
CB_020165	0.000473	4.56E-05	2.017991	up	up	54205	CYCS	
CB_026539	0.000472	4.54E-05	5.07814	up	up	127534	GJB4	
CB_013478	0.000465	4.46E-05	2.057889	up	up	6611	SMS	
CB_021769	0.000464	4.45E-05	2.191249	up	up	64785	GINS3	
CB_015768	0.000464	4.44E-05	2.484386	up	up	6613	SUMO2	
CB_007601	0.000464	4.45E-05	4.869641	up	up	55061	SUSD4	
CB_016803	0.00045	4.27E-05	10.31716	up	up	28983	TMPRSS11E	
CB_005144	0.000446	4.21E-05	13.63163	up	up	5317	PKP1	
CB_008039	0.000446	4.2E-05	2.39187	up	up	6715	SRD5A1	
CB_021531	0.00044	4.13E-05	4.441239	up	up	64065	PERP	
CB_010404	0.000438	4.11E-05	2.026184	up	up	1852	DUSP9	
CB_016455	0.000421	3.9E-05	5.647035	up	up	5275	SERPINB13	
CB_011237	0.000413	3.81E-05	2.038331	up	up	3797	KIF3C	
CB_008583	0.000409	3.76E-05	2.616238	up	up	200916	RPL22L1	
CB_016766	0.000407	3.72E-05	2.08444	up	up	50943	FOXP3	
CB_007823	0.0004	3.63E-05	8.875866	up	up	6696	SPP1	
CB_013482	0.000396	3.58E-05	2.602381	up	up	6804	STX1A	
CB_021445	0.000395	3.57E-05	2.34425	up	up	7552	ZNF711	
CB_011782	0.000385	3.44E-05	2.002446	up	up	5756	TWF1	
CB_014818	0.000378	3.36E-05	12.92912	up	up	6273	S100A2	
CB_028974	0.000375	3.31E-05	8.53017	up	up	23532	PRAME	
CB_005379	0.000374	3.29E-05	14.95129	up	up	3861	KRT14	
CB_023456	0.000341	2.91E-05	2.073402	up	up	84262	PSMG3	
CB_022912	0.000324	2.71E-05	2.013318	up	up	81611	ANP32E	

CB_016040	0.00032	2.67E-05	4.048485	up	up	11281	POU6F2	
CB_005227	0.000311	2.57E-05	2.072939	up	up	7372	UMPS	
CB_005089	0.000297	2.41E-05	2.301245	up	up	4436	MSH2	
CB_023215	0.000296	2.4E-05	2.507488	up	up	83886	PRSS27	
CB_009601	0.000283	2.27E-05	2.463413	up	up	28969	BZW2	
CB_015073	0.000276	2.2E-05	2.050936	up	up	5437	POLR2H	
CB_021882	0.000275	2.19E-05	2.280057	up	up	65243	ZNF643	
CB_023906	0.000263	2.05E-05	4.366167	up	up	84985	FAM83A	
CB_028331	0.000261	2.03E-05	4.196383	up	up	57822	GRHL3	
CB_012643	0.000257	1.98E-05	2.206929	up	up	8607	RUVBL1	
CB_012064	0.000227	1.68E-05	2.585967	up	up	6624	FSCN1	
CB_022265	0.000226	1.67E-05	4.184969	up	up	79733	E2F8	
CB_027609	0.000223	1.64E-05	13.0123	up	up	128876	FAM83C	
CB_008265	0.000212	1.53E-05	2.031625	up	up	57673	BEND3	
CB_013770	0.000208	1.5E-05	9.559887	up	up	9547	CXCL14	
CB_027787	0.000207	1.49E-05	2.083388	up	up	5983	RFC3	
CB_010871	0.000206	1.48E-05	13.67302	up	up	1469	CST1	
CB_008010	0.000204	1.47E-05	2.908737	up	up	63979	FIGNL1	
CB_020804	0.0002	1.43E-05	2.097365	up	up	57486	NLN	
CB_026918	0.00019	1.32E-05	2.237252	up	up	286826	LIN9	
CB_020401	0.00018	1.24E-05	14.06825	up	up	56649	TMPRSS4	
CB_008081	0.000179	1.23E-05	2.912851	up	up	7298	TYMS	
CB_008857	0.000179	1.23E-05	2.962287	up	up	729533	FAM72A	
CB_020467	0.000176	1.19E-05	2.317709	up	up	56924	PAK6	
CB_021221	0.000165	1.1E-05	22.22575	up	up	3237	HOXD11	
CB_026898	0.000164	1.09E-05	2.865109	up	up	259307	IL4I1	
CB_019177	0.00016	1.05E-05	2.191309	up	up	29980	DONSON	
CB_024826	0.00016	1.05E-05	8.823309	up	up	131578	LRRC15	
CB_005563	0.000158	1.04E-05	3.323822	up	up	1138	CHRNA5	
CB_011863	0.000157	1.03E-05	2.447793	up	up	5932	RBBP8	
CB_007069	0.000156	1.02E-05	2.067389	up	up	132299	OCIAD2	
CB_018622	0.000147	9.44E-06	2.087973	up	up	9585	KIF20B	
CB_010328	0.000143	9.16E-06	2.01688	up	up	1434	CSE1L	
CB_016408	0.000143	9.13E-06	2.012875	up	up	23542	MAPK8IP2	
CB_008882	0.000139	8.86E-06	3.771546	up	up	7161	TP73	
CB_027891	0.000139	8.83E-06	2.365505	up	up	353500	BMP8A	
CB_014934	0.000139	8.81E-06	5.503199	up	up	10381	TUBB3	
CB_028075	0.000137	8.64E-06	4.453892	up	up	55388	MCM10	
CB_016260	0.000137	8.61E-06	4.316323	up	up	26256	CABYR	
CB_017821	0.00013	8.04E-06	5.083996	up	up	23213	SULF1	
CB_008364	0.000128	7.94E-06	3.793023	up	up	284992	CCDC150	
CB_027514	0.000128	7.91E-06	2.300476	up	up	86	ACTL6A	
CB_010686	0.000127	7.84E-06	2.494633	up	up	576	BAI2	
CB_026105	0.000126	7.72E-06	2.026959	up	up	136051	ZNF786	
CB_012450	0.000125	7.61E-06	2.259692	up	up	8334	HIST1H2AC	
CB_021153	0.000119	7.2E-06	3.835836	up	up	5366	PMAIP1	
CB_007095	0.000119	7.2E-06	4.715367	up	up	548596	CKMT1A	
CB_021317	0.000118	7.06E-06	4.53182	up	up	8038	ADAM12	
CB_004929	0.000114	6.79E-06	6.126221	up	up	1294	COL7A1	
CB_011828	0.000112	6.64E-06	2.044025	up	up	5832	ALDH18A1	
CB_015399	0.000109	6.38E-06	3.526414	up	up	10643	IGF2BP3	
CB_015844	0.000107	6.23E-06	2.373953	up	up	11072	DUSP14	
CB_021780	0.000107	6.22E-06	2.031241	up	up	10198	MPHOSPH9	
CB_009459	0.000105	6.11E-06	10.0256	up	up	27299	ADAMDEC1	
CB_020868	0.000105	6.07E-06	3.436967	up	up	57549	IGSF9	
CB_019844	0.000101	5.77E-06	2.66167	up	up	55771	PRR11	

CB_025584	0.0001	5.71E-06	3.641022	up	up	165055	CCDC138	
CB_029352	9.79E-05	5.55E-06	2.279204	up	up	286016	TPI1P2	
CB_019518	9.72E-05	5.48E-06	9.048135	up	up	55040	EPN3	
CB_019914	9.5E-05	5.3E-06	2.316622	up	up	55342	STRBP	
CB_012189	9.37E-05	5.2E-06	2.550648	up	up	7023	TFAP4	
CB_020830	9.19E-05	5.06E-06	2.313923	up	up	57510	XPO5	
CB_013288	9.05E-05	4.95E-06	9.883808	up	up	1832	DSP	
CB_020643	9.04E-05	4.94E-06	4.771003	up	up	5522	PPP2R2C	
CB_026908	8.95E-05	4.87E-06	7.781741	up	up	26154	ABCA12	
CB_021182	8.83E-05	4.78E-06	2.089366	up	up	10793	ZNF273	
CB_023208	8.43E-05	4.47E-06	4.75588	up	up	83879	CDCA7	
CB_025626	8.26E-05	4.34E-06	2.776057	up	up	220042	C11orf82	
CB_023613	8.25E-05	4.33E-06	2.834059	up	up	84524	ZC3H8	
CB_025601	8.2E-05	4.29E-06	2.051914	up	up	200539	ANKRD23	
CB_011294	8.15E-05	4.25E-06	2.168284	up	up	3948	LDHC	
CB_028721	8.03E-05	4.16E-06	3.22341	up	up	55734	ZFP64	
CB_028597	8.02E-05	4.16E-06	2.270649	up	up	4521	NUDT1	
CB_009019	8.01E-05	4.14E-06	2.133552	up	up	1786	DNMT1	
CB_012015	8E-05	4.14E-06	2.673858	up	up	6474	SHOX2	
CB_015803	7.46E-05	3.75E-06	2.054449	up	up	7978	MTERF	
CB_007539	6.56E-05	3.11E-06	2.414795	up	up	80179	MYO19	
CB_018056	6.35E-05	2.98E-06	2.192428	up	up	25879	DCAF13	
CB_028852	6.18E-05	2.87E-06	2.75034	up	up	3925	STMN1	
CB_028248	6.1E-05	2.81E-06	2.039714	up	up	202051	SPATA24	
CB_027451	6E-05	2.74E-06	3.417887	up	up	147700	KLC3	
CB_018717	5.85E-05	2.64E-06	2.065157	up	up	51726	DNAJB11	
CB_011599	5.56E-05	2.44E-06	2.282806	up	up	5203	PFDN4	
CB_012442	5.47E-05	2.38E-06	3.476395	up	up	8318	CDC45	
CB_024335	5.39E-05	2.33E-06	2.236723	up	up	92667	C20orf72	
CB_017963	4.99E-05	2.08E-06	17.70359	up	up	50649	ARHGEF4	
CB_016855	4.89E-05	2.01E-06	2.089771	up	up	29088	MRPL15	
CB_022357	4.89E-05	2.01E-06	2.460944	up	up	79828	METTL8	
CB_026660	4.77E-05	1.93E-06	2.011967	up	up	9631	NUP155	
CB_010085	4.57E-05	1.8E-06	4.401907	up	up	54478	FAM64A	
CB_016800	4.46E-05	1.74E-06	2.305415	up	up	28982	FLVCR1	
CB_024216	4.18E-05	1.6E-06	2.039968	up	up	93323	HAUS8	
CB_024577	4.06E-05	1.53E-06	2.396023	up	up	114907	FBXO32	
CB_022854	3.97E-05	1.48E-06	2.013255	up	up	81557	MAGED4B	
CB_026067	3.84E-05	1.41E-06	2.119566	up	up	128061	C1orf131	
CB_012071	3.84E-05	1.41E-06	2.081313	up	up	6635	SNRPE	
CB_023542	3.79E-05	1.38E-06	2.112082	up	up	84365	MKI67IP	
CB_005343	3.28E-05	1.13E-06	10.74474	up	up	1300	COL10A1	
CB_020170	3.15E-05	1.06E-06	8.437497	up	up	3206	HOXA10	
CB_011850	3.14E-05	1.06E-06	2.011545	up	up	5902	RANBP1	
CB_010772	3.11E-05	1.04E-06	4.383635	up	up	995	CDC25C	
CB_028513	3.07E-05	1.02E-06	3.382159	up	up	375444	C5orf34	
CB_004894	3.06E-05	1.01E-06	2.771989	up	up	675	BRCA2	
CB_019640	2.96E-05	9.71E-07	3.890207	up	up	55143	CDCA8	
CB_016819	2.9E-05	9.44E-07	2.038822	up	up	28998	MRPL13	
CB_012927	2.87E-05	9.32E-07	24.75613	up	up	2706	GJB2	
CB_010771	2.75E-05	8.8E-07	5.179363	up	up	993	CDC25A	
CB_013955	2.66E-05	8.43E-07	3.619396	up	up	6564	SLC15A1	
CB_018746	2.59E-05	8.1E-07	6.981763	up	up	51195	RAPGEFL1	
CB_021883	2.57E-05	8.02E-07	2.140788	up	up	65244	SPATS2	
CB_025073	2.51E-05	7.81E-07	5.94409	up	up	113828	FAM83F	
CB_014110	2.5E-05	7.75E-07	3.361675	up	up	2139	EYA2	

CB_014836	2.46E-05	7.61E-07	2.016178	up	up	7203	CCT3	
CB_015025	2.44E-05	7.51E-07	2.004403	up	up	5036	PA2G4	
CB_012219	2.42E-05	7.39E-07	2.002453	up	up	7083	TK1	
CB_026206	2.28E-05	6.79E-07	3.770393	up	up	151246	SGOL2	
CB_014659	2.21E-05	6.48E-07	2.36081	up	up	10213	PSMD14	
CB_022911	2.19E-05	6.39E-07	6.800157	up	up	81610	FAM83D	
CB_015496	2.13E-05	6.11E-07	2.613091	up	up	10797	MTHFD2	
CB_020532	1.99E-05	5.57E-07	5.428022	up	up	56992	KIF15	
CB_017593	1.91E-05	5.27E-07	2.085226	up	up	6683	SPAST	
CB_025092	1.81E-05	4.85E-07	6.88293	up	up	115908	CTHRC1	
CB_025932	1.78E-05	4.76E-07	4.064807	up	up	8784	TNFRSF18	
CB_021953	1.73E-05	4.56E-07	4.089306	up	up	78995	C17orf53	
CB_014783	1.69E-05	4.42E-07	6.583206	up	up	4320	MMP11	
CB_026742	1.67E-05	4.33E-07	5.757341	up	up	5831	PYCR1	
CB_028095	1.66E-05	4.28E-07	2.471958	up	up	84515	MCM8	
CB_018074	1.65E-05	4.23E-07	2.343061	up	up	25902	MTHFD1L	
CB_007505	1.63E-05	4.18E-07	2.289331	up	up	6240	RRM1	
CB_022215	1.61E-05	4.09E-07	4.806212	up	up	79682	MLF1IP	
CB_006970	1.57E-05	3.91E-07	2.154793	up	up	400506	C16orf88	
CB_018944	1.5E-05	3.69E-07	2.188124	up	up	11335	CBX3	
CB_008475	1.48E-05	3.59E-07	6.177717	up	up	92312	MEX3A	
CB_014265	1.46E-05	3.53E-07	2.15504	up	up	6472	SHMT2	
CB_016930	1.36E-05	3.16E-07	3.307597	up	up	10733	PLK4	
CB_024060	1.34E-05	3.12E-07	9.669262	up	up	85416	ZIC5	
CB_021508	1.34E-05	3.08E-07	2.272328	up	up	63926	ANKRD5	
CB_010809	1.31E-05	2.99E-07	2.539847	up	up	1163	CKS1B	
CB_029120	1.3E-05	2.97E-07	4.057632	up	up	728833	FAM72D	
CB_016944	1.27E-05	2.86E-07	3.877003	up	up	22824	HSPA4L	
CB_024558	1.25E-05	2.81E-07	4.088986	up	up	29968	PSAT1	
CB_007204	1.23E-05	2.73E-07	5.330518	up	up	2175	FANCA	
CB_015370	1.17E-05	2.55E-07	9.346066	up	up	6513	SLC2A1	
CB_014060	1.13E-05	2.43E-07	7.160536	up	up	1033	CDKN3	
CB_016530	1.1E-05	2.36E-07	3.535944	up	up	7371	UCK2	
CB_028811	1.07E-05	2.25E-07	7.541266	up	up	5818	PVRL1	
CB_024251	1.03E-05	2.1E-07	3.214486	up	up	85465	EPT1	
CB_018007	9.77E-06	1.96E-07	2.016842	up	up	23518	R3HDM1	
CB_005125	9.08E-06	1.76E-07	9.921488	up	up	5080	PAX6	
CB_014351	8.88E-06	1.71E-07	2.272396	up	up	10051	SMC4	
CB_010792	8.28E-06	1.54E-07	11.8113	up	up	1058	CENPA	
CB_011667	8.24E-06	1.53E-07	2.570835	up	up	5471	PPAT	
CB_015891	7.94E-06	1.45E-07	3.018596	up	up	11169	WDHD1	
CB_028200	7.77E-06	1.39E-07	2.085212	up	up	5933	RBL1	
CB_005029	7.26E-06	1.26E-07	2.671419	up	up	3251	HPRT1	
CB_020479	6.95E-06	1.17E-07	6.563676	up	up	56938	ARNTL2	
CB_008258	6.95E-06	1.17E-07	6.302294	up	up	146909	KIF18B	
CB_023039	6.9E-06	1.16E-07	4.620087	up	up	83461	CDCA3	
CB_012519	6.73E-06	1.11E-07	4.154563	up	up	8438	RAD54L	
CB_019319	6.5E-06	1.06E-07	3.587252	up	up	54892	NCAPG2	
CB_017422	6.1E-06	9.69E-08	7.732533	up	up	9833	MELK	
CB_021976	5.65E-06	8.7E-08	2.067742	up	up	79023	NUP37	
CB_023954	5.55E-06	8.45E-08	5.045561	up	up	11130	ZWINT	
CB_012469	5.54E-06	8.4E-08	2.926324	up	up	8351	HIST1H3D	
CB_010271	5.23E-06	7.78E-08	3.958596	up	up	991	CDC20	
CB_010171	5.15E-06	7.62E-08	9.227006	up	up	64220	STRA6	
CB_028333	4.97E-06	7.18E-08	3.186395	up	up	4830	NME1	
CB_029409	4.75E-06	6.69E-08	4.523721	up	up	26255	PTTG3P	

CB_009775	4.67E-06	6.51E-08	2.598289	up	up	117178	SSX2IP	
CB_014592	4.49E-06	6.14E-08	7.301994	up	up	10112	KIF20A	
CB_024929	4.43E-06	5.98E-08	8.295918	up	up	165545	DQX1	
CB_012627	4.26E-06	5.63E-08	5.203899	up	up	9156	EXO1	
CB_010795	3.75E-06	4.7E-08	5.135078	up	up	1062	CENPE	
CB_015574	3.68E-06	4.58E-08	3.23456	up	up	10926	DBF4	
CB_016083	3.57E-06	4.4E-08	3.721623	up	up	672	BRCA1	
CB_010373	3.46E-06	4.17E-08	2.795596	up	up	1736	DKC1	
CB_019627	3.31E-06	3.85E-08	2.21219	up	up	55706	TMEM48	
CB_006895	3.28E-06	3.77E-08	6.063315	up	up	151648	SGOL1	
CB_019857	3.11E-06	3.44E-08	2.327043	up	up	55299	BRIX1	
CB_019605	3.03E-06	3.31E-08	4.819413	up	up	3070	HELLS	
CB_026199	2.87E-06	3.03E-08	6.568364	up	up	150468	CKAP2L	
CB_021970	2.86E-06	3.01E-08	2.740529	up	up	79017	GGCT	
CB_019526	2.7E-06	2.77E-08	3.220205	up	up	55055	ZWILCH	
CB_011439	2.57E-06	2.57E-08	3.472909	up	up	4605	MYBL2	
CB_008048	2.52E-06	2.5E-08	3.08281	up	up	1104	RCC1	
CB_021557	2.47E-06	2.42E-08	3.639717	up	up	64105	CENPK	
CB_011473	2.46E-06	2.41E-08	8.249326	up	up	4751	NEK2	
CB_022258	2.34E-06	2.23E-08	2.488512	up	up	79723	SUV39H2	
CB_007205	2.09E-06	1.84E-08	5.129974	up	up	2187	FANCB	
CB_020248	2.09E-06	1.82E-08	2.411378	up	up	54503	ZDHHC13	
CB_019941	1.95E-06	1.65E-08	10.303	up	up	55355	HJURP	
CB_026757	1.95E-06	1.64E-08	6.592221	up	up	57082	CASC5	
CB_001124	1.9E-06	1.6E-08	2.913111	up	up	84914	ZNF587	
CB_012913	1.48E-06	1.07E-08	5.993328	up	up	9055	PRC1	
CB_015779	1.45E-06	1.04E-08	4.176383	up	up	6712	SPTBN2	
CB_029056	1.43E-06	1.02E-08	3.257502	up	up	197021	LCTL	
CB_011250	1.4E-06	9.95E-09	3.114931	up	up	3838	KPNA2	
CB_020086	1.39E-06	9.83E-09	9.744709	up	up	54443	ANLN	
CB_007207	1.32E-06	8.77E-09	2.896934	up	up	2177	FANCD2	
CB_021972	1.32E-06	8.69E-09	4.323378	up	up	79019	CENPM	
CB_026005	1.28E-06	8.23E-09	4.60358	up	up	120071	GYLTL1B	
CB_019671	1.26E-06	7.83E-09	10.79581	up	up	55165	CEP55	
CB_010234	1.21E-06	7.2E-09	4.350581	up	up	701	BUB1B	
CB_021100	1.17E-06	6.73E-09	6.939571	up	up	9837	GINS1	
CB_015698	1.16E-06	6.66E-09	5.645041	up	up	11004	KIF2C	
CB_012273	1.15E-06	6.47E-09	7.397501	up	up	7272	TTK	
CB_011246	1.13E-06	6.13E-09	5.277687	up	up	3833	KIFC1	
CB_011340	1.12E-06	6.04E-09	5.72598	up	up	4085	MAD2L1	
CB_028030	1.04E-06	5.24E-09	4.430143	up	up	348654	GEN1	
CB_028805	1.02E-06	5.13E-09	3.628467	up	up	2305	FOXM1	
CB_015742	9.78E-07	4.76E-09	2.723111	up	up	5591	PRKDC	
CB_026240	9.59E-07	4.53E-09	13.46312	up	up	157313	CDCA2	
CB_027885	8.1E-07	3.53E-09	7.858919	up	up	11065	UBE2C	
CB_012458	7.31E-07	2.92E-09	2.911765	up	up	8341	HIST1H2BN	
CB_012473	7E-07	2.75E-09	3.492342	up	up	8355	HIST1H3G	
CB_013102	6.92E-07	2.67E-09	4.589055	up	up	9232	PTTG1	
CB_014750	6.78E-07	2.55E-09	5.064336	up	up	4173	MCM4	
CB_011140	6.4E-07	2.25E-09	2.037849	up	up	3329	HSPD1	
CB_024170	6.19E-07	2.05E-09	4.41462	up	up	91687	CENPL	
CB_007784	5.96E-07	1.85E-09	4.451874	up	up	89839	ARHGAP11B	
CB_028399	5.58E-07	1.56E-09	4.646009	up	up	6790	AURKA	
CB_016197	5.46E-07	1.48E-09	9.487779	up	up	22974	TPX2	
CB_015337	5.14E-07	1.34E-09	4.945448	up	up	10635	RAD51AP1	
CB_017366	4.55E-07	1.03E-09	7.857845	up	up	9768	KIAA0101	

CB_010768	3.9E-07	7.61E-10	8.59259	up	up	983	CDK1	
CB_016591	3.61E-07	6.54E-10	4.385835	up	up	29127	RACGAP1	
CB_018805	3.27E-07	5.5E-10	7.26905	up	up	51512	GTSE1	
CB_008806	2.5E-07	3.49E-10	6.364032	up	up	1111	CHEK1	
CB_008263	1.26E-07	1.18E-10	5.990538	up	up	1763	DNA2	
CB_008075	6.22E-08	1.82E-11	12.53551	up	up	7153	TOP2A	
CB_004233	0.04964	0.025316	2.185746	up	up			
CB_026490	0.024612	0.010321	4.384974	up	up	260436	C4orf7	
CB_000658	0.02165	0.008721	2.069732	up	up	730338	LOC730338	
CB_009519	0.0167	0.006191	2.313173	up	up	389493	LOC389493	
CB_022467	0.009368	0.002832	2.613577	up	up	79953	TMEM90B	
CB_016340	0.007293	0.001995	2.481461	up	up	24141	C20orf103	
CB_022670	0.005012	0.001182	2.065398	up	up	80264	ZNF430	
CB_027711	0.0028	0.000527	7.060399	up	up	204219	LASS3	
CB_020371	0.002386	0.000421	4.342252	up	up	56300	IL1F9	
CB_026929	0.00192	0.000312	3.299448	up	up	27177	IL1F8	
CB_016352	0.00141	0.000202	2.530516	up	up	26525	IL1F5	
CB_010879	0.00115	0.000153	2.374808	up	up	1503	CTPS	
CB_026018	0.000675	7.41E-05	2.124403	up	up	121793	C13orf16	
CB_006968	0.000509	5.04E-05	2.306094	up	up	388753	C1orf31	
CB_010071	0.000509	5.03E-05	2.080594	up	up	1.01E+08	LOC100506581	
CB_009571	0.00048	4.66E-05	2.040011	up	up	1E+08	LOC100130932	
CB_019702	0.000433	4.06E-05	2.088923	up	up	55726	C12orf11	
CB_007921	0.000387	3.46E-05	2.271971	up	up	723790	HIST2H2AA4	
CB_016265	0.00013	8.04E-06	3.00243	up	up	25758	C11orf41	
CB_001259	0.000126	7.76E-06	2.443863	up	up	284889	LOC284889	
CB_008542	6.89E-05	3.35E-06	4.439571	up	up	26011	ODZ4	
CB_007597	5.93E-05	2.69E-06	2.00255	up	up	84792	C7orf70	
CB_021959	5.19E-05	2.2E-06	3.315525	up	up	79000	C1orf135	
CB_023495	4.97E-05	2.07E-06	2.080648	up	up	84300	C6orf125	
CB_025111	4.62E-05	1.84E-06	2.18408	up	up	154467	C6orf129	
CB_026212	3.05E-05	1.01E-06	2.05176	up	up	152002	C3orf21	
CB_024751	1.21E-05	2.66E-07	2.119122	up	up	140893	C20orf151	
CB_020161	5.61E-06	8.6E-08	2.405964	up	up	54069	C21orf45	
CB_028883	5.14E-06	7.59E-08	2.821882	up	up	253782	LASS6	
CB_019481	2.3E-06	2.14E-08	5.477889	up	up	55010	C12orf48	
CB_025960	6.23E-07	2.14E-09	6.014549	up	up	90381	C15orf42	
CB_027594	5.99E-07	1.91E-09	7.301668	up	up	89958	C9orf140	
CB_022441	0.001202	0.000162	3.346058	down	down	79919	C2orf54	
CB_007118	4.61E-05	1.83E-06	2.395281	down	down	445329	SULT1A4	
CB_024462	3.41E-05	1.19E-06	2.935086	down	down	4638	MYLK	
CB_019040	0.045287	0.022554	2.200791	down	down	8862	APLN	
CB_010561	0.044141	0.02184	2.122815	down	down	3579	CXCR2	
CB_005511	0.028606	0.012547	3.319323	down	down	186	AGTR2	
CB_028802	0.028232	0.01232	2.066892	down	down	6296	ACSM3	
CB_010800	0.027663	0.012002	2.019467	down	down	1089	CEACAM4	
CB_005061	0.026272	0.011229	2.494785	down	down	3815	KIT	
CB_027727	0.026194	0.011185	2.505549	down	down	353189	SLCO4C1	
CB_011700	0.026009	0.011086	2.121411	down	down	5577	PRKAR2B	
CB_027529	0.025574	0.010849	2.578071	down	down	345275	HSD17B13	
CB_017505	0.024356	0.010181	2.233841	down	down	9914	ATP2C2	
CB_020156	0.023276	0.009606	2.000257	down	down	56129	PCDHB7	
CB_021914	0.022705	0.009283	2.179533	down	down	65987	KCTD14	
CB_008033	0.020676	0.008208	2.868807	down	down	401551	WDR38	
CB_006998	0.017851	0.006763	2.347421	down	down	257177	C1orf192	
CB_016082	0.017217	0.006441	2.893144	down	down	4311	MME	

CB_024814	0.015679	0.005691	2.126823	down	down	64407	RGS18	
CB_005307	0.015672	0.005687	2.433391	down	down	6401	SELE	
CB_016045	0.015362	0.005535	2.354279	down	down	10687	PNMA2	
CB_014099	0.015355	0.005531	2.020151	down	down	2042	EPHA3	
CB_021881	0.014046	0.00492	2.251983	down	down	6614	SIGLEC1	
CB_022080	0.013552	0.004687	2.095407	down	down	79191	IRX3	
CB_020154	0.013355	0.004592	2.170304	down	down	56131	PCDHB4	
CB_027816	0.013308	0.004572	2.130254	down	down	148808	MFSD4	
CB_026119	0.013091	0.004464	2.026782	down	down	138311	FAM69B	
CB_013334	0.013038	0.004437	2.319738	down	down	2119	ETV5	
CB_010435	0.012357	0.004123	2.182584	down	down	2012	EMP1	
CB_030564	0.012012	0.003967	3.900749	down	down	150147	C21orf128	
CB_018889	0.012006	0.003965	2.333458	down	down	51348	KLRF1	
CB_024494	0.011865	0.0039	3.299258	down	down	83648	FAM167A	
CB_005749	0.010941	0.003497	2.032481	down	down	5345	SERPINF2	
CB_012000	0.010876	0.00347	2.544138	down	down	6425	SFRP5	
CB_021569	0.010649	0.003373	2.042632	down	down	64122	FN3K	
CB_009580	0.010486	0.003299	2.244447	down	down	290	ANPEP	
CB_015809	0.010335	0.003236	2.133045	down	down	9501	RPH3AL	
CB_011965	0.009717	0.002974	2.791796	down	down	6366	CCL21	
CB_005573	0.009714	0.002973	3.074064	down	down	1437	CSF2	
CB_028434	0.009478	0.002877	4.946384	down	down	284340	CXCL17	
CB_013495	0.009445	0.002863	2.627483	down	down	7103	TSPAN8	
CB_012955	0.009281	0.002794	2.019261	down	down	828	CAPS	
CB_014463	0.009077	0.002707	2.160639	down	down	4818	NKG7	
CB_027373	0.009039	0.002692	2.510938	down	down	161753	ODF3L1	
CB_024000	0.008334	0.002406	2.604307	down	down	4135	MAP6	
CB_012887	0.007666	0.002141	3.290218	down	down	9023	CH25H	
CB_020304	0.007429	0.002047	2.145637	down	down	54621	VSIG10	
CB_005214	0.00733	0.002009	2.644546	down	down	7056	THBD	
CB_021432	0.007203	0.001961	2.371242	down	down	6581	SLC22A3	
CB_020296	0.007198	0.001959	2.429405	down	down	54567	DLL4	
CB_011149	0.006882	0.001839	2.417469	down	down	3397	ID1	
CB_013090	0.006856	0.001828	2.416424	down	down	9099	USP2	
CB_005380	0.006779	0.001801	2.34272	down	down	3949	LDLR	
CB_023179	0.006699	0.001771	2.137361	down	down	114898	C1QTNF2	
CB_020859	0.006683	0.001764	2.548284	down	down	57538	ALPK3	
CB_028156	0.006428	0.001673	3.009962	down	down	3249	HPN	
CB_007009	0.006383	0.001657	2.448211	down	down	388610	TRNP1	
CB_022314	0.006207	0.001594	4.152594	down	down	79785	RERGL	
CB_026661	0.006164	0.001578	2.642629	down	down	197257	LDHD	
CB_020463	0.005816	0.001454	2.444163	down	down	56920	SEMA3G	
CB_005249	0.005808	0.001451	2.25646	down	down	1536	CYBB	
CB_016021	0.005796	0.001446	3.202688	down	down	11254	SLC6A14	
CB_024732	0.005685	0.001406	3.066339	down	down	1602	DACH1	
CB_017352	0.005555	0.001362	2.411913	down	down	9750	FAM65B	
CB_017777	0.005528	0.001353	2.285516	down	down	23150	FRMD4B	
CB_019189	0.005421	0.001317	10.42473	down	down	55600	ITLN1	
CB_022413	0.005171	0.001237	2.326242	down	down	79887	PLBD1	
CB_029065	0.004728	0.001088	2.14138	down	down	285440	CYP4V2	
CB_018168	0.004716	0.001085	2.109677	down	down	26032	SUSD5	
CB_024525	0.004713	0.001084	2.519016	down	down	117248	GALNTL2	
CB_009487	0.004575	0.00104	2.206786	down	down	400713	ZNF880	
CB_010594	0.004531	0.001026	2.036381	down	down	72	ACTG2	
CB_028922	0.004525	0.001025	2.578399	down	down	2494	NR5A2	
CB_028992	0.004471	0.001007	2.243872	down	down	79819	WDR78	

CB_010755	0.004434	0.000996	2.076878	down	down	945	CD33	
CB_005726	0.004431	0.000995	3.79677	down	down	4886	NPY1R	
CB_014938	0.004091	0.000891	3.007669	down	down	10391	CORO2B	
CB_029187	0.004057	0.000881	2.237777	down	down	203859	ANO5	
CB_013114	0.003798	0.000805	2.183052	down	down	9308	CD83	
CB_010903	0.003778	0.000799	2.726651	down	down	1803	DPP4	
CB_021290	0.003772	0.000797	2.021603	down	down	59084	ENPP5	
CB_020568	0.003746	0.000789	3.232446	down	down	57221	KIAA1244	
CB_011188	0.003659	0.000763	2.388547	down	down	3675	ITGA3	
CB_016574	0.003625	0.000754	3.528734	down	down	10891	PPARGC1A	
CB_028310	0.003539	0.000729	2.213795	down	down	256380	SCML4	
CB_001894	0.003431	0.000699	2.190424	down	down	168667	BMPER	
CB_019267	0.003396	0.000689	3.476413	down	down	54848	ARHGEF38	
CB_011109	0.003388	0.000687	2.034458	down	down	3127	HLA-DRB5	
CB_014083	0.003374	0.000683	3.170643	down	down	1668	DEFA3	
CB_008527	0.003363	0.00068	19.13995	down	down	729238	SFTPA2	
CB_007395	0.00322	0.00064	2.110347	down	down	394	ARHGAP5	
CB_005706	0.003131	0.000615	2.264802	down	down	3687	ITGAX	
CB_027443	0.003117	0.000611	2.336932	down	down	53829	P2RY13	
CB_026152	0.003091	0.000604	2.073331	down	down	146434	ZNF597	
CB_004913	0.002909	0.000556	2.81656	down	down	1028	CDKN1C	
CB_023152	0.002874	0.000546	2.650677	down	down	8325	FZD8	
CB_001538	0.002851	0.00054	2.271161	down	down	5265	SERPINA1	
CB_005038	0.002789	0.000524	2.58914	down	down	3383	ICAM1	
CB_001921	0.002787	0.000523	2.750999	down	down	257019	FRMD3	
CB_021258	0.00271	0.000503	3.813089	down	down	59277	NTN4	
CB_011458	0.002691	0.000499	6.615272	down	down	4680	CEACAM6	
CB_028358	0.00267	0.000493	2.232446	down	down	2934	GSN	
CB_010873	0.002534	0.000457	2.681747	down	down	1490	CTGF	
CB_020873	0.00247	0.000442	2.664333	down	down	57556	SEMA6A	
CB_026324	0.002464	0.000441	2.048612	down	down	200132	TCTEX1D1	
CB_025081	0.002463	0.00044	2.410312	down	down	115207	KCTD12	
CB_028529	0.002443	0.000435	2.016615	down	down	377677	CA13	
CB_017397	0.002435	0.000433	2.552977	down	down	1718	DHCR24	
CB_020047	0.002418	0.000429	7.557438	down	down	55885	LMO3	
CB_024750	0.00241	0.000427	2.566516	down	down	140886	PABPC5	
CB_013676	0.002406	0.000426	3.176594	down	down	9365	KL	
CB_023199	0.002328	0.000406	2.24345	down	down	83872	HMCN1	
CB_025676	0.002269	0.000392	2.694388	down	down	1154	CISH	
CB_028793	0.002236	0.000385	2.224079	down	down	9414	TJP2	
CB_006978	0.002148	0.000364	2.155485	down	down	4059	BCAM	
CB_020145	0.002148	0.000364	3.716419	down	down	56138	PCDHA11	
CB_010850	0.002136	0.000361	3.411998	down	down	1359	CPA3	
CB_013345	0.002118	0.000357	2.548627	down	down	2255	FGF10	
CB_005342	0.002097	0.000353	6.092153	down	down	1080	CFTR	
CB_006164	0.002087	0.00035	2.014817	down	down	388335	TMEM220	
CB_014740	0.002085	0.00035	2.784345	down	down	4093	SMAD9	
CB_024815	0.00207	0.000346	2.555518	down	down	90139	TSPAN18	
CB_011911	0.002064	0.000345	2.867992	down	down	6098	ROS1	
CB_024044	0.002034	0.000338	2.437689	down	down	85409	NKD2	
CB_022830	0.002023	0.000335	2.937898	down	down	81027	TUBB1	
CB_005058	0.001967	0.000322	2.311007	down	down	3784	KCNQ1	
CB_017183	0.001927	0.000314	2.802173	down	down	11227	GALNT5	
CB_029135	0.001927	0.000314	2.963429	down	down	400451	FAM174B	
CB_023071	0.001918	0.000312	2.989891	down	down	64332	NFKBIZ	
CB_026021	0.001911	0.00031	2.588431	down	down	122481	AK7	

CB_022319	0.001892	0.000306	2.062362	down	down	79789	CLMN	
CB_001820	0.001862	0.0003	2.04722	down	down	56255	TMX4	
CB_022363	0.001862	0.0003	4.515446	down	down	79838	TMC5	
CB_007449	0.001801	0.000286	2.206912	down	down	91746	YTHDC1	
CB_011062	0.001791	0.000283	3.158404	down	down	2921	CXCL3	
CB_004780	0.001786	0.000282	2.016094	down	down	51312	SLC25A37	
CB_027855	0.00172	0.000268	2.178256	down	down	338773	TMEM119	
CB_010974	0.00169	0.000261	2.501272	down	down	2247	FGF2	
CB_023914	0.001639	0.00025	3.639547	down	down	85004	RERG	
CB_023122	0.00161	0.000244	2.633294	down	down	83716	CRISPLD2	
CB_018306	0.001591	0.00024	2.343522	down	down	50486	G0S2	
CB_010438	0.001558	0.000234	2.37119	down	down	2014	EMP3	
CB_005774	0.001525	0.000227	2.970313	down	down	5732	PTGER2	
CB_018816	0.001515	0.000225	2.165366	down	down	51232	CRIM1	
CB_005619	0.001512	0.000224	2.375487	down	down	2350	FOLR2	
CB_010967	0.001509	0.000224	2.032132	down	down	2204	FCAR	
CB_026475	0.001479	0.000217	4.090697	down	down	222865	TMEM130	
CB_017678	0.001471	0.000216	2.312355	down	down	23043	TNIK	
CB_008272	0.001442	0.000209	2.150174	down	down	94030	LRRC4B	
CB_012047	0.00141	0.000202	5.264833	down	down	6590	SLPI	
CB_011668	0.0014	0.0002	4.556183	down	down	5473	PPBP	
CB_022276	0.001382	0.000197	2.338065	down	down	79742	CXorf36	
CB_023793	0.001351	0.000191	2.636154	down	down	84870	RSPO3	
CB_018897	0.001351	0.000191	3.223837	down	down	51761	ATP8A2	
CB_014405	0.001343	0.000189	2.134579	down	down	3632	INPP5A	
CB_022382	0.001339	0.000189	2.031788	down	down	79856	SNX22	
CB_022063	0.001333	0.000187	3.030999	down	down	79170	PRR15L	
CB_008699	0.0013	0.000181	3.12902	down	down	54852	PAQR5	
CB_018492	0.001292	0.00018	2.162499	down	down	51643	TMBIM4	
CB_007666	0.001285	0.000178	2.877247	down	down	6340	SCNN1G	
CB_010764	0.001283	0.000178	2.524181	down	down	969	CD69	
CB_008059	0.001243	0.00017	5.524799	down	down	6751	SSTR1	
CB_025333	0.001227	0.000168	2.278726	down	down	84807	NFKBID	
CB_022859	0.001212	0.000165	2.517035	down	down	81563	C1orf21	
CB_028125	0.001207	0.000164	2.437384	down	down	56999	ADAMTS9	
CB_009626	0.001205	0.000163	3.076824	down	down	57139	RGL3	
CB_021421	0.001186	0.00016	2.885555	down	down	8431	NR0B2	
CB_023810	0.001176	0.000157	2.224477	down	down	83700	JAM3	
CB_017080	0.001168	0.000156	2.500654	down	down	27123	DKK2	
CB_024105	0.001166	0.000156	2.02664	down	down	89927	C16orf45	
CB_009204	0.00112	0.000148	2.712632	down	down	1E+08	FAM47E	
CB_015494	0.001116	0.000147	2.056362	down	down	10791	VAMP5	
CB_007875	0.001105	0.000145	2.569029	down	down	27253	PCDH17	
CB_020431	0.001104	0.000145	4.095452	down	down	56892	C8orf4	
CB_012212	0.001102	0.000144	2.157223	down	down	7067	THRA	
CB_012405	0.001058	0.000137	2.754176	down	down	7855	FZD5	
CB_005163	0.001042	0.000134	2.235947	down	down	5627	PROS1	
CB_021239	0.001032	0.000132	2.100329	down	down	58488	PCTP	
CB_017153	0.001023	0.000131	2.707219	down	down	27345	KCNMB4	
CB_009585	0.001021	0.000131	2.056907	down	down	309	ANXA6	
CB_026760	0.001014	0.000129	2.083297	down	down	54414	SIAE	
CB_018034	0.001004	0.000127	3.527697	down	down	25849	PARM1	
CB_012499	0.000997	0.000126	3.896357	down	down	8395	PIP5K1B	
CB_006707	0.000986	0.000124	2.841206	down	down	400831	C20orf202	
CB_005109	0.000983	0.000123	2.020453	down	down	653361	NCF1	
CB_016290	0.000975	0.000122	2.122541	down	down	11043	MID2	

CB_021036	0.000969	0.000121	3.598288	down	down	366	AQP9	
CB_025755	0.000966	0.000121	2.028747	down	down	132671	SPATA18	
CB_022255	0.000965	0.00012	2.075559	down	down	79720	VPS37B	
CB_010719	0.000964	0.00012	2.533406	down	down	727	C5	
CB_017694	0.000953	0.000118	2.054881	down	down	23062	GGA2	
CB_021373	0.000943	0.000117	2.139229	down	down	558	AXL	
CB_014795	0.000935	0.000115	2.41444	down	down	4502	MT2A	
CB_010991	0.000922	0.000113	2.081382	down	down	2321	FLT1	
CB_012221	0.000914	0.000112	2.861948	down	down	7089	TLE2	
CB_023064	0.0009	0.000109	2.00987	down	down	23710	GABARAPL1	
CB_020767	0.000899	0.000109	2.216933	down	down	57419	SLC24A3	
CB_017513	0.000896	0.000109	2.515983	down	down	9920	KBTBD11	
CB_022228	0.000895	0.000109	2.104389	down	down	79695	GALNT12	
CB_017830	0.000892	0.000108	2.038887	down	down	23220	DTX4	
CB_020151	0.000882	0.000106	2.462389	down	down	56121	PCDHB15	
CB_013465	0.00088	0.000106	2.355048	down	down	5920	RARRES3	
CB_014288	0.000869	0.000104	3.742084	down	down	7851	MALL	
CB_019899	0.000862	0.000103	2.689747	down	down	55332	DRAM1	
CB_010347	0.000852	0.000101	2.761377	down	down	1521	CTSW	
CB_017367	0.000848	0.000101	2.409404	down	down	9770	RASSF2	
CB_010232	0.000846	0.0001	2.047508	down	down	659	BMPR2	
CB_012317	0.000841	9.95E-05	2.108644	down	down	7408	VASP	
CB_028231	0.000837	9.89E-05	2.513059	down	down	348093	RBPMS2	
CB_027723	0.000835	9.87E-05	2.170461	down	down	152189	CMTM8	
CB_018200	0.000824	9.69E-05	2.497639	down	down	23034	SAMD4A	
CB_016407	0.000824	9.68E-05	2.60829	down	down	23764	MAFF	
CB_021143	0.000818	9.6E-05	3.451605	down	down	7373	COL14A1	
CB_015345	0.000813	9.52E-05	2.161592	down	down	2124	EVI2B	
CB_014126	0.000801	9.35E-05	2.018434	down	down	2669	GEM	
CB_014523	0.000801	9.34E-05	2.09411	down	down	7071	KLF10	
CB_015284	0.000795	9.26E-05	17.85808	down	down	10568	SLC34A2	
CB_020805	0.000792	9.21E-05	2.083729	down	down	49854	ZNF295	
CB_017551	0.000784	9.1E-05	2.107876	down	down	2744	GLS	
CB_022323	0.000783	9.08E-05	2.565449	down	down	79794	C12orf49	
CB_026221	0.000763	8.76E-05	2.414583	down	down	153020	RASGEF1B	
CB_028459	0.000751	8.57E-05	3.717061	down	down	342527	SMTNL2	
CB_008326	0.000751	8.57E-05	2.147566	down	down	152573	SHISA3	
CB_022016	0.000741	8.42E-05	2.577369	down	down	79083	MLPH	
CB_012914	0.000741	8.42E-05	2.421793	down	down	9056	SLC7A7	
CB_009911	0.000729	8.25E-05	2.148421	down	down	397	ARHGDIB	
CB_025646	0.000724	8.17E-05	2.187238	down	down	112464	PRKCDBP	
CB_024836	0.000707	7.89E-05	3.208226	down	down	83657	DYNLRB2	
CB_017528	0.000706	7.88E-05	2.026875	down	down	10144	FAM13A	
CB_012910	0.000704	7.85E-05	7.77248	down	down	9052	GPRC5A	
CB_026363	0.000701	7.8E-05	2.398824	down	down	220164	DOK6	
CB_020845	0.000696	7.73E-05	2.101369	down	down	57528	KCTD16	
CB_018707	0.000687	7.59E-05	2.141761	down	down	51411	BIN2	
CB_024230	0.00068	7.49E-05	2.021593	down	down	89853	FAM125B	
CB_016044	0.000678	7.46E-05	2.451745	down	down	11309	SLCO2B1	
CB_019059	0.000661	7.18E-05	4.578533	down	down	53905	DUOX1	
CB_020709	0.000652	7.05E-05	2.120626	down	down	57192	MCOLN1	
CB_010757	0.000642	6.91E-05	2.440823	down	down	951	CD37	
CB_010402	0.000627	6.68E-05	2.361601	down	down	1842	ECM2	
CB_019687	0.000626	6.66E-05	2.018578	down	down	55177	FAM82A2	
CB_018176	0.000625	6.65E-05	2.063817	down	down	26040	SETBP1	
CB_017845	0.00062	6.59E-05	4.756366	down	down	23237	ARC	

CB_012990	0.000616	6.53E-05	2.043477	down	down	1845	DUSP3	
CB_014255	0.000616	6.53E-05	2.906896	down	down	5581	PRKCE	
CB_007398	0.000614	6.5E-05	2.180378	down	down	389432	SAMD5	
CB_027427	0.000613	6.49E-05	4.583347	down	down	4499	MT1M	
CB_014781	0.000612	6.46E-05	2.052503	down	down	4303	FOXO4	
CB_010785	0.000608	6.41E-05	3.472179	down	down	1043	CD52	
CB_017363	0.00059	6.16E-05	2.48865	down	down	9764	KIAA0513	
CB_021469	0.000571	5.89E-05	3.027262	down	down	58191	CXCL16	
CB_005189	0.000566	5.82E-05	2.097199	down	down	6444	SGCD	
CB_013188	0.000558	5.7E-05	2.230834	down	down	323	APBB2	
CB_020673	0.000551	5.6E-05	2.051786	down	down	57185	NIPAL3	
CB_023635	0.00055	5.59E-05	2.614791	down	down	84612	PARD6B	
CB_007133	0.000544	5.5E-05	2.126497	down	down	114769	CARD16	
CB_025344	0.000542	5.48E-05	3.864485	down	down	200879	LIPH	
CB_027311	0.000536	5.4E-05	2.855862	down	down	4493	MT1E	
CB_005598	0.000536	5.39E-05	4.224595	down	down	1593	CYP27A1	
CB_011165	0.000527	5.28E-05	2.139854	down	down	3563	IL3RA	
CB_007547	0.000526	5.26E-05	3.347707	down	down	619279	ZNF704	
CB_011213	0.000508	5.02E-05	3.133549	down	down	3738	KCNA3	
CB_026065	0.000504	4.96E-05	4.398772	down	down	127733	UBXN10	
CB_015548	0.000496	4.87E-05	2.918915	down	down	10893	MMP24	
CB_009590	0.000495	4.85E-05	2.572185	down	down	11279	KLF8	
CB_016168	0.000491	4.79E-05	2.024098	down	down	23414	ZFPM2	
CB_027382	0.000485	4.72E-05	2.398359	down	down	283897	C16orf54	
CB_020049	0.000483	4.68E-05	6.675845	down	down	54210	TREM1	
CB_015358	0.00048	4.65E-05	2.101553	down	down	5817	PVR	
CB_018779	0.00047	4.52E-05	2.877948	down	down	9465	AKAP7	
CB_018939	0.000468	4.49E-05	2.87287	down	down	51296	SLC15A3	
CB_017061	0.000462	4.42E-05	5.35787	down	down	27074	LAMP3	
CB_018096	0.000459	4.38E-05	2.443075	down	down	25925	ZNF521	
CB_020878	0.000453	4.31E-05	3.431562	down	down	57562	KIAA1377	
CB_011407	0.000453	4.3E-05	2.915844	down	down	4332	MNDA	
CB_022060	0.000452	4.29E-05	2.809967	down	down	79168	LILRA6	
CB_012841	0.000451	4.29E-05	2.120685	down	down	8905	AP1S2	
CB_020833	0.000451	4.28E-05	2.567975	down	down	57513	CASKIN2	
CB_009540	0.00045	4.27E-05	3.861933	down	down	6563	SLC14A1	
CB_021272	0.000447	4.23E-05	4.433033	down	down	58529	MYOZ1	
CB_022981	0.000442	4.16E-05	2.13618	down	down	6916	TBXAS1	
CB_017217	0.000434	4.06E-05	2.020154	down	down	8916	HERC3	
CB_025891	0.000427	3.97E-05	2.193208	down	down	259230	SGMS1	
CB_016689	0.000421	3.91E-05	3.822804	down	down	29953	TRHDE	
CB_010720	0.000413	3.81E-05	3.203472	down	down	728	C5AR1	
CB_025082	0.000407	3.72E-05	2.462257	down	down	115330	GPR146	
CB_021072	0.000397	3.59E-05	2.643513	down	down	6641	SNTB1	
CB_025667	0.000392	3.53E-05	2.15274	down	down	196383	RILPL2	
CB_026016	0.000387	3.47E-05	3.182787	down	down	121506	ERP27	
CB_024783	0.000387	3.47E-05	4.518931	down	down	81035	COLEC12	
CB_010814	0.000385	3.43E-05	2.418104	down	down	1183	CLCN4	
CB_015818	0.000385	3.43E-05	3.231023	down	down	10560	SLC19A2	
CB_020167	0.000384	3.42E-05	2.572897	down	down	54206	ERRFI1	
CB_019626	0.000376	3.33E-05	3.638586	down	down	55137	FIGN	
CB_023436	0.000376	3.33E-05	8.145519	down	down	84239	ATP13A4	
CB_016683	0.000375	3.31E-05	3.377237	down	down	29950	SERTAD1	
CB_006972	0.000374	3.3E-05	2.299585	down	down	401548	SNX30	
CB_018021	0.00037	3.25E-05	2.196123	down	down	55187	VPS13D	
CB_014214	0.00037	3.25E-05	2.24628	down	down	3727	JUND	

CB_005474	0.000363	3.18E-05	3.965363	down	down	3577	CXCR1	
CB_017254	0.000363	3.17E-05	2.088581	down	down	9663	LPIN2	
CB_014762	0.000362	3.16E-05	4.50437	down	down	4223	MEOX2	
CB_012286	0.00036	3.14E-05	2.364017	down	down	7305	TYROBP	
CB_019014	0.000351	3.03E-05	5.63844	down	down	53358	SHC3	
CB_012899	0.00035	3.01E-05	3.100021	down	down	9037	SEMA5A	
CB_012234	0.000348	2.99E-05	2.488294	down	down	7111	TMOD1	
CB_023594	0.000344	2.95E-05	2.003377	down	down	7462	LAT2	
CB_026489	0.000344	2.95E-05	3.437797	down	down	256435	ST6GALNAC3	
CB_008974	0.000343	2.93E-05	2.893375	down	down	729085	FAM198A	
CB_011641	0.000334	2.83E-05	4.214149	down	down	5350	PLN	
CB_012304	0.000329	2.77E-05	9.057343	down	down	7356	SCGB1A1	
CB_027519	0.000328	2.76E-05	2.505387	down	down	91404	SESTD1	
CB_015471	0.000327	2.75E-05	2.118813	down	down	10750	GRAP	
CB_023680	0.000325	2.73E-05	3.410275	down	down	84658	EMR3	
CB_011707	0.000322	2.7E-05	2.021455	down	down	5587	PRKD1	
CB_005269	0.000321	2.68E-05	2.163885	down	down	3459	IFNGR1	
CB_023445	0.000316	2.63E-05	2.563359	down	down	84251	SGIP1	
CB_007594	0.000311	2.56E-05	4.366012	down	down	192668	CYS1	
CB_010327	0.000309	2.54E-05	2.824067	down	down	1397	CRIP2	
CB_021935	0.000301	2.45E-05	2.884481	down	down	1879	EBF1	
CB_016421	0.000298	2.42E-05	3.762451	down	down	23554	TSPAN12	
CB_014941	0.000288	2.32E-05	4.107905	down	down	10396	ATP8A1	
CB_016781	0.000285	2.28E-05	3.162948	down	down	25840	METTL7A	
CB_008292	0.00028	2.23E-05	2.065158	down	down	389072	PLEKHM3	
CB_005775	0.000275	2.19E-05	2.574951	down	down	5734	PTGER4	
CB_012886	0.000272	2.15E-05	3.756025	down	down	9021	SOCS3	
CB_004842	0.000269	2.12E-05	2.075324	down	down	38	ACAT1	
CB_013568	0.000264	2.06E-05	2.804071	down	down	9121	SLC16A5	
CB_017200	0.000261	2.03E-05	2.595851	down	down	30061	SLC40A1	
CB_007897	0.000261	2.03E-05	4.704104	down	down	467	ATF3	
CB_015994	0.000253	1.95E-05	2.564098	down	down	11213	IRAK3	
CB_025752	0.000253	1.95E-05	2.440571	down	down	130497	OSR1	
CB_012225	0.000251	1.93E-05	2.899183	down	down	7098	TLR3	
CB_010962	0.000246	1.88E-05	2.789115	down	down	2180	ACSL1	
CB_023292	0.000242	1.84E-05	2.745993	down	down	56100	PCDHGB6	
CB_013453	0.000239	1.8E-05	2.209366	down	down	5330	PLCB2	
CB_016858	0.000234	1.75E-05	3.862049	down	down	29091	STXBP6	
CB_000824	0.000233	1.74E-05	6.113417	down	down	120892	LRRK2	
CB_011521	0.00023	1.71E-05	5.481533	down	down	4973	OLR1	
CB_011620	0.000227	1.68E-05	20.80368	down	down	5284	PIGR	
CB_014062	0.000227	1.68E-05	2.475075	down	down	1051	CEBPB	
CB_023342	0.00022	1.62E-05	3.410897	down	down	84101	USP44	
CB_016972	0.00022	1.61E-05	2.547963	down	down	23533	PIK3R5	
CB_029890	0.000218	1.59E-05	2.269173	down	down	84852	ATP1A1OS	
CB_009177	0.000216	1.57E-05	2.781629	down	down	255809	C19orf38	
CB_012216	0.000215	1.57E-05	2.129275	down	down	7077	TIMP2	
CB_005187	0.000203	1.45E-05	3.421918	down	down	6338	SCNN1B	
CB_020889	0.000198	1.41E-05	3.266324	down	down	57573	ZNF471	
CB_020238	0.000197	1.39E-05	2.885331	down	down	54491	FAM105A	
CB_015840	0.000196	1.39E-05	4.152515	down	down	11067	C10orf10	
CB_007694	0.000195	1.38E-05	2.46941	down	down	220979	C10orf25	
CB_020100	0.000195	1.38E-05	2.773583	down	down	11107	PRDM5	
CB_019638	0.000191	1.33E-05	2.887915	down	down	55711	FAR2	
CB_014585	0.000186	1.29E-05	3.26291	down	down	10100	TSPAN2	
CB_023962	0.000185	1.28E-05	11.6112	down	down	4969	OGN	

CB_018433	0.00018	1.24E-05	5.544837	down	down	51090	PLLP	
CB_023085	0.000177	1.21E-05	3.220134	down	down	83604	TMEM47	
CB_014663	0.00017	1.14E-05	2.944235	down	down	10219	KLRG1	
CB_011932	0.000168	1.12E-05	3.060769	down	down	6272	SORT1	
CB_010548	0.000167	1.11E-05	3.73625	down	down	3400	ID4	
CB_020769	0.000167	1.11E-05	4.934003	down	down	57452	GALNTL1	
CB_010892	0.000165	1.1E-05	2.523632	down	down	1634	DCN	
CB_007231	0.000161	1.07E-05	2.06054	down	down	3176	HNMT	
CB_013786	0.000159	1.05E-05	2.706446	down	down	9592	IER2	
CB_019152	0.000151	9.86E-06	2.286194	down	down	54764	ZRANB1	
CB_005680	0.000151	9.79E-06	8.236585	down	down	3248	HPGD	
CB_016697	0.000146	9.41E-06	2.045056	down	down	23670	TMEM2	
CB_016422	0.000143	9.19E-06	2.205653	down	down	23555	TSPAN15	
CB_023695	0.000142	9.05E-06	2.309861	down	down	84674	CARD6	
CB_012938	0.000139	8.83E-06	2.871426	down	down	388	RHOB	
CB_023294	0.000139	8.81E-06	2.981665	down	down	56099	PCDHGB7	
CB_011419	0.000138	8.72E-06	2.079043	down	down	4478	MSN	
CB_028652	0.000137	8.67E-06	3.955336	down	down	6387	CXCL12	
CB_022660	0.000136	8.55E-06	2.47959	down	down	5362	PLXNA2	
CB_026719	0.000136	8.54E-06	2.112397	down	down	128077	LIX1L	
CB_015499	0.000136	8.54E-06	2.587082	down	down	10800	CYSLTR1	
CB_015724	0.000135	8.48E-06	2.234152	down	down	11041	B3GNT1	
CB_014697	0.000134	8.42E-06	2.016705	down	down	10268	RAMP3	
CB_023883	0.000132	8.21E-06	3.683841	down	down	84953	MICALCL	
CB_005055	0.000129	7.99E-06	4.887351	down	down	3730	KAL1	
CB_021332	0.000129	7.96E-06	2.800168	down	down	60370	AVPI1	
CB_005419	0.000128	7.94E-06	2.801486	down	down	3570	IL6R	
CB_028348	0.000127	7.85E-06	2.237082	down	down	2040	STOM	
CB_019222	0.000127	7.81E-06	2.777549	down	down	54810	GIPC2	
CB_023545	0.000125	7.67E-06	2.493902	down	down	5098	PCDHGC3	
CB_016593	0.000124	7.59E-06	3.016987	down	down	745	C11orf9	
CB_026559	0.000123	7.49E-06	3.166935	down	down	168537	GIMAP7	
CB_018108	0.000119	7.18E-06	2.22424	down	down	25939	SAMHD1	
CB_020836	0.000119	7.17E-06	2.940396	down	down	57519	STARD9	
CB_016560	0.000118	7.08E-06	2.542532	down	down	7761	ZNF214	
CB_016419	0.000118	7.07E-06	2.32155	down	down	4542	MYO1F	
CB_005282	0.000117	7.03E-06	2.765186	down	down	4053	LTBP2	
CB_028318	0.000117	7.02E-06	2.106304	down	down	2595	GANC	
CB_023512	0.000117	7.02E-06	4.544442	down	down	84316	LSMD1	
CB_008246	0.000116	6.97E-06	4.901718	down	down	114800	CCDC85A	
CB_020704	0.000115	6.88E-06	2.309049	down	down	4792	NFKBIA	
CB_013557	0.000115	6.87E-06	4.368545	down	down	8404	SPARCL1	
CB_020885	0.000115	6.86E-06	3.060521	down	down	57569	ARHGAP20	
CB_015725	0.000114	6.79E-06	2.405379	down	down	2766	GMPR	
CB_018898	0.000112	6.64E-06	2.165145	down	down	51762	RAB8B	
CB_014739	0.000112	6.64E-06	2.12547	down	down	4092	SMAD7	
CB_016768	0.000109	6.41E-06	2.261254	down	down	28954	REM1	
CB_013227	0.000107	6.27E-06	6.741926	down	down	820	CAMP	
CB_028475	0.000105	6.07E-06	3.162148	down	down	374378	GALNTL4	
CB_005142	0.000105	6.06E-06	2.193003	down	down	5311	PKD2	
CB_005517	0.000104	6.01E-06	4.03112	down	down	217	ALDH2	
CB_017851	0.000104	6E-06	2.194238	down	down	23243	ANKRD28	
CB_013734	0.000103	5.95E-06	19.8756	down	down	9476	NAPSA	
CB_021565	0.000103	5.94E-06	6.014967	down	down	64116	SLC39A8	
CB_024191	0.000103	5.94E-06	2.117769	down	down	85464	SSH2	
CB_014070	0.000103	5.93E-06	2.763607	down	down	1326	MAP3K8	

CB_023083	0.000102	5.87E-06	2.176137	down	down	83595	SOX7		
CB_005207	0.000102	5.86E-06	2.648172	down	down	412	STS		
CB_016305	0.000102	5.85E-06	2.329092	down	down	22921	MSRB2		
CB_022414	0.000102	5.83E-06	3.097801	down	down	79888	LPCAT1		
CB_017120	0.0001	5.74E-06	7.380785	down	down	27289	RND1		
CB_007536	0.0001	5.72E-06	3.019292	down	down	619208	C6orf225		
CB_003247	9.8E-05	5.56E-06	2.05465	down	down	11346	SYNPO		
CB_014048	9.8E-05	5.56E-06	5.713368	down	down	761	CA3		
CB_005069	9.79E-05	5.55E-06	4.7644	down	down	6445	SGCG		
CB_027774	9.79E-05	5.54E-06	2.22131	down	down	10398	MYL9		
CB_022679	9.68E-05	5.43E-06	2.16126	down	down	80301	PLEKHO2		
CB_016382	9.66E-05	5.41E-06	2.383932	down	down	23194	FBXL7		
CB_017186	9.58E-05	5.37E-06	3.110876	down	down	26508	HEYL		
CB_020601	9.32E-05	5.16E-06	2.88514	down	down	57104	PNPLA2		
CB_015233	9.29E-05	5.14E-06	2.366312	down	down	10493	VAT1		
CB_027476	9.15E-05	5.02E-06	3.017709	down	down	427	ASAH1		
CB_008474	9.06E-05	4.97E-06	5.234159	down	down	32	ACACB		
CB_022746	8.99E-05	4.9E-06	12.43185	down	down	80761	UPK3B		
CB_011524	8.87E-05	4.81E-06	2.58291	down	down	4983	OPHN1		
CB_011351	8.81E-05	4.77E-06	3.49186	down	down	4118	MAL		
CB_021226	8.81E-05	4.76E-06	5.260418	down	down	58189	WFDC1		
CB_012767	8.61E-05	4.61E-06	4.269652	down	down	8793	TNFRSF10D		
CB_017266	8.48E-05	4.51E-06	2.326864	down	down	9674	KIAA0040		
CB_023966	8.26E-05	4.34E-06	2.32481	down	down	85360	SYDE1		
CB_010989	8.08E-05	4.2E-06	2.833099	down	down	2313	FLI1		
CB_015920	7.97E-05	4.12E-06	2.494803	down	down	7402	UTRN		
CB_028180	7.93E-05	4.09E-06	2.672776	down	down	140738	TMEM37		
CB_013184	7.69E-05	3.93E-06	3.659252	down	down	9630	GNA14		
CB_016938	7.63E-05	3.87E-06	3.179462	down	down	11173	ADAMTS7		
CB_020123	7.57E-05	3.82E-06	3.503716	down	down	55930	MYO5C		
CB_006605	7.43E-05	3.73E-06	2.77491	down	down	55075	UACA		
CB_010627	7.19E-05	3.56E-06	2.193736	down	down	334	APLP2		
CB_010912	7.18E-05	3.55E-06	2.78571	down	down	1848	DUSP6		
CB_026632	7.13E-05	3.5E-06	2.343761	down	down	219654	ZCCHC24		
CB_008251	7.13E-05	3.51E-06	2.48325	down	down	126374	WTIP		
CB_008612	7.13E-05	3.52E-06	2.58676	down	down	10335	MRVI1		
CB_028313	7.05E-05	3.45E-06	6.864181	down	down	358	AQP1		
CB_018553	6.97E-05	3.4E-06	2.513505	down	down	51393	TRPV2		
CB_017053	6.96E-05	3.39E-06	16.28759	down	down	27063	ANKRD1		
CB_004908	6.9E-05	3.35E-06	3.528974	down	down	825	CAPN3		
CB_011375	6.89E-05	3.34E-06	2.519481	down	down	4211	MEIS1		
CB_013980	6.86E-05	3.32E-06	4.607545	down	down	9590	AKAP12		
CB_009484	6.86E-05	3.32E-06	2.805607	down	down	23331	TTC28		
CB_016680	6.79E-05	3.27E-06	2.22466	down	down	10015	PDCD6IP		
CB_005358	6.78E-05	3.26E-06	5.351567	down	down	2203	FBP1		
CB_014678	6.66E-05	3.19E-06	2.659892	down	down	10241	CALCOCO2		
CB_024317	6.63E-05	3.17E-06	3.110512	down	down	2788	GNG7		
CB_011986	6.61E-05	3.15E-06	2.988193	down	down	6404	SELPLG		
CB_010920	6.56E-05	3.11E-06	5.202741	down	down	1906	EDN1		
CB_017843	6.52E-05	3.08E-06	2.473167	down	down	23235	SIK2		
CB_021705	6.48E-05	3.06E-06	2.37949	down	down	7145	TNS1		
CB_014539	6.45E-05	3.04E-06	2.343117	down	down	7905	REEP5		
CB_022416	6.44E-05	3.03E-06	2.200117	down	down	79890	RIN3		
CB_016484	6.41E-05	3.01E-06	2.092974	down	down	6251	RSU1		
CB_009321	6.37E-05	2.99E-06	10.13298	down	down	91851	CHRDL1		
CB_020035	6.35E-05	2.97E-06	3.39552	down	down	55450	CAMK2N1		

CB_017237	6.29E-05	2.94E-06	2.878403	down	down	9639	ARHGEF10	
CB_018281	6.21E-05	2.89E-06	2.135634	down	down	55841	WWC3	
CB_007653	6.17E-05	2.86E-06	2.587098	down	down	85458	DIXDC1	
CB_007839	6.14E-05	2.85E-06	2.014339	down	down	116985	ARAP1	
CB_013945	6.14E-05	2.84E-06	3.644891	down	down	6368	CCL23	
CB_020810	6.14E-05	2.84E-06	2.605263	down	down	57493	HEG1	
CB_026944	6.06E-05	2.78E-06	2.629186	down	down	150094	SIK1	
CB_011874	5.92E-05	2.69E-06	2.022755	down	down	5962	RDX	
CB_020534	5.82E-05	2.61E-06	2.033179	down	down	56994	CHPT1	
CB_025449	5.69E-05	2.53E-06	2.90085	down	down	130132	RFTN2	
CB_020888	5.58E-05	2.46E-06	2.137025	down	down	57572	DOCK6	
CB_026463	5.55E-05	2.43E-06	2.734912	down	down	260425	MAGI3	
CB_028934	5.48E-05	2.39E-06	3.712377	down	down	387496	RASL11A	
CB_011889	5.46E-05	2.38E-06	3.532113	down	down	5997	RGS2	
CB_008370	5.35E-05	2.31E-06	3.155374	down	down	6688	SPI1	
CB_017923	5.25E-05	2.24E-06	3.143241	down	down	23328	SASH1	
CB_012621	5.18E-05	2.2E-06	2.18014	down	down	8566	PDXK	
CB_015474	5.17E-05	2.19E-06	3.104899	down	down	10763	NES	
CB_026338	5.11E-05	2.16E-06	2.062436	down	down	202052	DNAJC18	
CB_006925	5.11E-05	2.15E-06	6.561944	down	down	196996	GRAMD2	
CB_010045	4.94E-05	2.04E-06	3.289262	down	down	253827	MSRB3	
CB_012715	4.9E-05	2.02E-06	2.542649	down	down	8722	CTSF	
CB_021527	4.88E-05	2E-06	2.545282	down	down	64061	TSPYL2	
CB_007034	4.83E-05	1.97E-06	3.528059	down	down	440503	PLIN5	
CB_006184	4.83E-05	1.97E-06	2.649782	down	down	440738	MAP1LC3C	
CB_013492	4.81E-05	1.95E-06	3.79322	down	down	7052	TGM2	
CB_019184	4.79E-05	1.94E-06	2.023166	down	down	55599	RNPC3	
CB_012798	4.78E-05	1.94E-06	2.16214	down	down	8829	NRP1	
CB_010337	4.73E-05	1.9E-06	5.994802	down	down	1474	CST6	
CB_004953	4.61E-05	1.84E-06	2.871755	down	down	2022	ENG	
CB_024803	4.61E-05	1.83E-06	3.106109	down	down	170575	GIMAP1	
CB_005620	4.61E-05	1.83E-06	6.412283	down	down	2352	FOLR3	
CB_017431	4.58E-05	1.81E-06	2.766646	down	down	9844	ELMO1	
CB_022296	4.58E-05	1.81E-06	3.261966	down	down	79762	C1orf115	
CB_008837	4.51E-05	1.77E-06	2.07529	down	down	8653	DDX3Y	
CB_013532	4.49E-05	1.76E-06	2.910002	down	down	8313	AXIN2	
CB_013762	4.44E-05	1.74E-06	2.627733	down	down	9535	GMFG	
CB_016918	4.31E-05	1.67E-06	2.227514	down	down	9693	RAPGEF2	
CB_011210	4.22E-05	1.62E-06	2.671094	down	down	3726	JUNB	
CB_005077	4.15E-05	1.58E-06	4.420212	down	down	4128	MAOA	
CB_025253	4.13E-05	1.58E-06	4.669233	down	down	195814	SDR16C5	
CB_012684	4.13E-05	1.58E-06	5.559326	down	down	8671	SLC4A4	
CB_014700	4.13E-05	1.57E-06	2.05319	down	down	5813	PURA	
CB_024620	4.09E-05	1.55E-06	3.524221	down	down	140628	GATA5	
CB_014625	4.08E-05	1.54E-06	2.17265	down	down	10160	FARP1	
CB_021234	4.05E-05	1.53E-06	3.088918	down	down	58480	RHOU	
CB_021551	4E-05	1.5E-06	2.698855	down	down	64097	EPB41L4A	
CB_017606	3.97E-05	1.48E-06	2.555044	down	down	22898	DENND3	
CB_005714	3.87E-05	1.42E-06	2.947312	down	down	4048	LTA4H	
CB_026092	3.86E-05	1.42E-06	3.249143	down	down	132720	C4orf32	
CB_022507	3.84E-05	1.41E-06	2.220345	down	down	80005	DOCK5	
CB_023255	3.84E-05	1.4E-06	2.18339	down	down	83935	TMEM133	
CB_013709	3.84E-05	1.4E-06	4.5356	down	down	9429	ABCG2	
CB_010218	3.83E-05	1.4E-06	5.661848	down	down	650	BMP2	
CB_021651	3.67E-05	1.32E-06	3.877873	down	down	64344	HIF3A	
CB_016910	3.66E-05	1.32E-06	2.990396	down	down	8994	LIMD1	

CB_008004	3.65E-05	1.31E-06	2.717775	down	down	2268	FGR	
CB_020541	3.63E-05	1.3E-06	2.908372	down	down	10060	ABCC9	
CB_013061	3.59E-05	1.27E-06	6.769502	down	down	6505	SLC1A1	
CB_005075	3.43E-05	1.2E-06	7.201427	down	down	4023	LPL	
CB_004978	3.43E-05	1.2E-06	3.308608	down	down	2206	MS4A2	
CB_012803	3.43E-05	1.2E-06	5.011815	down	down	8835	SOCS2	
CB_013218	3.4E-05	1.18E-06	2.517974	down	down	683	BST1	
CB_023986	3.39E-05	1.17E-06	12.78873	down	down	57864	SLC46A2	
CB_028389	3.3E-05	1.14E-06	6.633818	down	down	23767	FLRT3	
CB_027900	3.26E-05	1.12E-06	2.188888	down	down	255877	BCL6B	
CB_013861	3.24E-05	1.11E-06	2.940532	down	down	3764	KCNJ8	
CB_016370	3.24E-05	1.11E-06	2.647866	down	down	9771	RAPGEF5	
CB_017029	3.22E-05	1.1E-06	3.059704	down	down	26353	HSPB8	
CB_024362	3.18E-05	1.08E-06	2.069946	down	down	114781	BTBD9	
CB_012005	3.14E-05	1.05E-06	31.38555	down	down	6440	SFTPC	
CB_028862	3.13E-05	1.05E-06	4.708727	down	down	221091	LRRN4CL	
CB_022373	3.07E-05	1.02E-06	2.328553	down	down	79845	RNF122	
CB_017592	3.07E-05	1.02E-06	2.469187	down	down	22885	ABLIM3	
CB_019123	3.05E-05	1.01E-06	3.663036	down	down	54749	EPDR1	
CB_010326	2.95E-05	9.68E-07	5.607285	down	down	1396	CRIP1	
CB_028837	2.92E-05	9.58E-07	4.872724	down	down	387758	FIBIN	
CB_013309	2.9E-05	9.46E-07	3.93104	down	down	1960	EGR3	
CB_016520	2.87E-05	9.3E-07	3.291359	down	down	7092	TLL1	
CB_028686	2.87E-05	9.3E-07	3.738241	down	down	79960	PHF17	
CB_020269	2.83E-05	9.15E-07	2.757336	down	down	54532	USP53	
CB_017416	2.8E-05	9.02E-07	2.130096	down	down	9828	ARHGEF17	
CB_007972	2.79E-05	8.97E-07	3.030083	down	down	8425	LTBP4	
CB_010898	2.69E-05	8.57E-07	7.366758	down	down	1674	DES	
CB_023903	2.67E-05	8.5E-07	3.150901	down	down	84976	DISP1	
CB_028176	2.6E-05	8.14E-07	2.373224	down	down	113612	CYP2U1	
CB_014495	2.59E-05	8.09E-07	4.219657	down	down	6578	SLCO2A1	
CB_005525	2.59E-05	8.09E-07	3.646925	down	down	240	ALOX5	
CB_021144	2.45E-05	7.54E-07	3.313628	down	down	8434	RECK	
CB_012349	2.43E-05	7.42E-07	3.999048	down	down	7538	ZFP36	
CB_010250	2.43E-05	7.41E-07	4.703632	down	down	858	CAV2	
CB_012781	2.37E-05	7.17E-07	2.965216	down	down	8809	IL18R1	
CB_014253	2.33E-05	7.03E-07	4.312275	down	down	5507	PPP1R3C	
CB_021628	2.32E-05	6.96E-07	4.92709	down	down	3386	ICAM4	
CB_021570	2.32E-05	6.93E-07	3.878734	down	down	64123	ELTD1	
CB_010463	2.29E-05	6.82E-07	2.698347	down	down	2295	FOXF2	
CB_005165	2.21E-05	6.49E-07	2.043082	down	down	5728	PTEN	
CB_005222	2.2E-05	6.42E-07	8.287756	down	down	7274	TTPA	
CB_023584	2.18E-05	6.34E-07	2.49928	down	down	200014	CC2D1B	
CB_005027	2.17E-05	6.31E-07	3.322625	down	down	6910	TBX5	
CB_020650	2.13E-05	6.12E-07	2.627128	down	down	57153	SLC44A2	
CB_017187	2.09E-05	5.94E-07	2.509401	down	down	26524	LATS2	
CB_011209	2.09E-05	5.93E-07	3.186913	down	down	3725	JUN	
CB_027251	2.07E-05	5.87E-07	3.618103	down	down	6330	SCN4B	
CB_015659	2.06E-05	5.82E-07	2.048558	down	down	10955	SERINC3	
CB_022523	2.04E-05	5.76E-07	2.544933	down	down	80019	UBTD1	
CB_006699	2.03E-05	5.73E-07	5.514698	down	down	414308	MRC1L1	
CB_017136	2.02E-05	5.66E-07	4.872522	down	down	27306	HPGDS	
CB_010436	1.91E-05	5.27E-07	4.214088	down	down	2013	EMP2	
CB_019553	1.9E-05	5.23E-07	3.684778	down	down	55081	IFT57	
CB_016820	1.89E-05	5.15E-07	8.297302	down	down	28999	KLF15	
CB_010929	1.85E-05	5.03E-07	6.518963	down	down	1958	EGR1	

CB_011167	1.85E-05	5.02E-07	3.967956	down	down	3572	IL6ST	
CB_018322	1.84E-05	4.99E-07	4.186296	down	down	4008	LMO7	
CB_023184	1.84E-05	4.99E-07	5.22097	down	down	83850	ESYT3	
CB_029147	1.84E-05	4.98E-07	2.400074	down	down	57188	ADAMTSL3	
CB_011110	1.83E-05	4.93E-07	6.319729	down	down	3131	HLF	
CB_016492	1.81E-05	4.86E-07	4.999774	down	down	9723	SEMA3E	
CB_025280	1.76E-05	4.68E-07	4.659202	down	down	170689	ADAMTS15	
CB_009281	1.75E-05	4.66E-07	3.389528	down	down	55289	ACOXL	
CB_029181	1.69E-05	4.4E-07	2.323593	down	down	65059	RAPH1	
CB_014458	1.69E-05	4.39E-07	3.431232	down	down	4781	NFIB	
CB_027304	1.69E-05	4.38E-07	3.805428	down	down	155038	GIMAP8	
CB_026278	1.67E-05	4.32E-07	4.4426	down	down	164312	LRRN4	
CB_016961	1.64E-05	4.22E-07	2.078462	down	down	23473	CAPN7	
CB_024421	1.63E-05	4.16E-07	4.405716	down	down	116159	CYYR1	
CB_010613	1.63E-05	4.14E-07	3.375159	down	down	241	ALOX5AP	
CB_020579	1.63E-05	4.14E-07	4.111193	down	down	57088	PLSCR4	
CB_007335	1.62E-05	4.12E-07	2.29206	down	down	27106	ARRDC2	
CB_016976	1.61E-05	4.09E-07	11.43372	down	down	23584	VSIG2	
CB_014638	1.61E-05	4.09E-07	3.306325	down	down	10186	LHFP	
CB_002548	1.59E-05	4E-07	4.703182	down	down	4919	ROR1	
CB_012973	1.58E-05	3.99E-07	2.144941	down	down	1263	PLK3	
CB_012204	1.57E-05	3.91E-07	4.576302	down	down	7049	TGFBR3	
CB_012183	1.55E-05	3.86E-07	2.674638	down	down	7006	TEC	
CB_020877	1.5E-05	3.68E-07	2.986992	down	down	57561	ARRDC3	
CB_008985	1.45E-05	3.5E-07	2.785656	down	down	23348	DOCK9	
CB_028139	1.45E-05	3.47E-07	3.090191	down	down	23345	SYNE1	
CB_009339	1.44E-05	3.43E-07	8.366487	down	down	2330	FMO5	
CB_019394	1.42E-05	3.36E-07	3.593842	down	down	54941	RNF125	
CB_012078	1.41E-05	3.34E-07	2.615653	down	down	6642	SNX1	
CB_003472	1.4E-05	3.31E-07	3.651388	down	down	4685	NCAM2	
CB_012554	1.32E-05	3.03E-07	3.341722	down	down	8490	RGS5	
CB_019863	1.32E-05	3.02E-07	5.31527	down	down	55304	SPTLC3	
CB_007433	1.3E-05	2.96E-07	2.477646	down	down	57222	ERGIC1	
CB_005371	1.28E-05	2.9E-07	10.29833	down	down	3043	HBB	
CB_020838	1.24E-05	2.77E-07	3.653561	down	down	57520	HECW2	
CB_028576	1.22E-05	2.71E-07	2.409093	down	down	166336	PRICKLE2	
CB_012607	1.19E-05	2.62E-07	6.25497	down	down	8547	FCN3	
CB_022191	1.17E-05	2.54E-07	3.771976	down	down	79652	TMEM204	
CB_007355	1.11E-05	2.38E-07	5.309283	down	down	343637	RSPO4	
CB_023809	1.11E-05	2.37E-07	3.317177	down	down	84886	C1orf198	
CB_024260	1.1E-05	2.36E-07	3.044493	down	down	93663	ARHGAP18	
CB_026101	1.09E-05	2.31E-07	3.100151	down	down	134265	AFAP1L1	
CB_023882	1.07E-05	2.26E-07	6.420013	down	down	84952	CGNL1	
CB_026849	1.06E-05	2.22E-07	2.853066	down	down	122773	KLHDC1	
CB_028330	1.06E-05	2.22E-07	3.193092	down	down	4286	MITF	
CB_018709	1.06E-05	2.2E-07	2.380482	down	down	51449	PCYOX1	
CB_025729	1.05E-05	2.19E-07	3.198958	down	down	93010	B3GNT7	
CB_023117	1.04E-05	2.16E-07	3.537474	down	down	83699	SH3BGRL2	
CB_010462	1.04E-05	2.16E-07	4.395596	down	down	2294	FOXF1	
CB_012081	1.04E-05	2.15E-07	4.365323	down	down	6649	SOD3	
CB_013534	1.04E-05	2.15E-07	11.58552	down	down	8436	SDPR	
CB_010543	1.02E-05	2.07E-07	3.11515	down	down	3316	HSPB2	
CB_018879	9.97E-06	2.01E-07	3.850834	down	down	51267	CLEC1A	
CB_008149	9.54E-06	1.9E-07	4.676346	down	down	641700	ECSCR	
CB_011587	9.43E-06	1.86E-07	7.75562	down	down	5166	PDK4	
CB_009298	9.25E-06	1.81E-07	2.167993	down	down	53373	TPCN1	

CB_021339	9.2E-06	1.8E-07	2.466955	down	down	10742	RAI2	
CB_016086	9.16E-06	1.79E-07	2.968539	down	down	3373	HYAL1	
CB_021602	9.15E-06	1.78E-07	4.47529	down	down	64174	DPEP2	
CB_017889	9.1E-06	1.76E-07	2.432584	down	down	23294	ANKS1A	
CB_028566	9.1E-06	1.76E-07	7.623678	down	down	283383	GPR133	
CB_014122	8.67E-06	1.66E-07	5.018823	down	down	2627	GATA6	
CB_012126	8.43E-06	1.59E-07	11.43109	down	down	6769	STAC	
CB_022961	8.22E-06	1.52E-07	4.157251	down	down	81848	SPRY4	
CB_019365	8.01E-06	1.47E-07	3.968054	down	down	54922	RASIP1	
CB_022098	7.96E-06	1.45E-07	4.005769	down	down	57194	ATP10A	
CB_005745	7.94E-06	1.45E-07	13.46234	down	down	5319	PLA2G1B	
CB_026968	7.92E-06	1.44E-07	2.173748	down	down	123879	DCUN1D3	
CB_021472	7.87E-06	1.42E-07	6.422402	down	down	63876	PKNOX2	
CB_018277	7.84E-06	1.41E-07	4.426269	down	down	27145	FILIP1	
CB_017787	7.66E-06	1.37E-07	2.043606	down	down	23164	MPRIP	
CB_015469	7.65E-06	1.36E-07	2.326516	down	down	10749	KIF1C	
CB_010586	7.65E-06	1.36E-07	12.97822	down	down	33	ACADL	
CB_024801	7.64E-06	1.36E-07	3.684055	down	down	122953	JDP2	
CB_024596	7.59E-06	1.34E-07	3.416256	down	down	976	CD97	
CB_010600	7.46E-06	1.3E-07	2.728895	down	down	79026	AHNAK	
CB_011303	7.41E-06	1.29E-07	9.431776	down	down	3977	LIFR	
CB_015559	7.3E-06	1.27E-07	2.918178	down	down	10908	PNPLA6	
CB_020834	7.16E-06	1.23E-07	3.771334	down	down	57514	ARHGAP31	
CB_023143	7.15E-06	1.23E-07	5.499646	down	down	185	AGTR1	
CB_027971	7.13E-06	1.22E-07	31.22953	down	down	200504	GKN2	
CB_018748	7.1E-06	1.21E-07	3.461964	down	down	51196	PLCE1	
CB_012734	6.95E-06	1.17E-07	3.306848	down	down	8742	TNFSF12	
CB_004899	6.94E-06	1.17E-07	2.603531	down	down	710	SERPING1	
CB_014332	6.89E-06	1.15E-07	2.334364	down	down	10023	FRAT1	
CB_027842	6.8E-06	1.13E-07	2.663441	down	down	160622	GRASP	
CB_022604	6.57E-06	1.08E-07	5.353863	down	down	80177	MYCT1	
CB_014447	6.44E-06	1.05E-07	4.357892	down	down	4091	SMAD6	
CB_028144	6.38E-06	1.04E-07	4.3512	down	down	4739	NEDD9	
CB_008677	6.16E-06	9.84E-08	5.347271	down	down	131873	COL6A6	
CB_022691	6.05E-06	9.57E-08	7.669291	down	down	80323	CCDC68	
CB_005633	6.02E-06	9.5E-08	2.512212	down	down	2621	GAS6	
CB_005510	5.87E-06	9.15E-08	9.594139	down	down	153	ADRB1	
CB_017831	5.72E-06	8.83E-08	4.06608	down	down	23221	RHOBTB2	
CB_011060	5.64E-06	8.69E-08	9.717057	down	down	2920	CXCL2	
CB_002587	5.54E-06	8.41E-08	3.080322	down	down	311	ANXA11	
CB_028431	5.54E-06	8.43E-08	3.390743	down	down	283298	OLFML1	
CB_022720	5.54E-06	8.43E-08	10.6911	down	down	80704	SLC19A3	
CB_009619	5.53E-06	8.34E-08	4.715469	down	down	202151	RANBP3L	
CB_013840	5.24E-06	7.8E-08	6.27899	down	down	2662	GDF10	
CB_013664	5.23E-06	7.79E-08	2.083701	down	down	9344	TAOK2	
CB_021922	5.13E-06	7.56E-08	6.066366	down	down	79098	C1orf116	
CB_010949	5.08E-06	7.46E-08	2.003697	down	down	2060	EPS15	
CB_018579	5.06E-06	7.42E-08	4.430299	down	down	51673	TPPP3	
CB_022745	4.97E-06	7.2E-08	13.72014	down	down	80760	ITIH5	
CB_020352	4.95E-06	7.13E-08	3.067832	down	down	56261	GPCPD1	
CB_027498	4.92E-06	7.03E-08	2.856654	down	down	90627	STARD13	
CB_005741	4.78E-06	6.76E-08	4.132828	down	down	5241	PGR	
CB_007218	4.61E-06	6.37E-08	3.446955	down	down	91663	MYADM	
CB_004917	4.54E-06	6.22E-08	2.133502	down	down	1130	LYST	
CB_010783	4.37E-06	5.85E-08	7.685852	down	down	1036	CDO1	
CB_011055	4.33E-06	5.77E-08	5.757623	down	down	2878	GPX3	

CB_015549	4.33E-06	5.74E-08	10.58594	down	down	10894	LYVE1	
CB_006219	4.33E-06	5.75E-08	12.71675	down	down	338596	ST8SIA6	
CB_005370	4.28E-06	5.66E-08	13.12	down	down	3040	HBA2	
CB_008827	4.2E-06	5.52E-08	2.763297	down	down	115	ADCY9	
CB_011043	4.17E-06	5.46E-08	2.345459	down	down	2776	GNAQ	
CB_025655	4E-06	5.14E-08	2.097388	down	down	127700	OSCP1	
CB_010756	4E-06	5.12E-08	4.324341	down	down	947	CD34	
CB_013720	3.82E-06	4.8E-08	2.911771	down	down	9459	ARHGEF6	
CB_020319	3.81E-06	4.79E-08	3.834491	down	down	3202	HOXA5	
CB_023717	3.62E-06	4.47E-08	2.508556	down	down	84701	COX4I2	
CB_025791	3.52E-06	4.31E-08	7.200689	down	down	219790	RTKN2	
CB_012691	3.48E-06	4.24E-08	4.696623	down	down	8676	STX11	
CB_004837	3.47E-06	4.19E-08	5.811726	down	down	2	A2M	
CB_021668	3.46E-06	4.14E-08	2.375297	down	down	64411	ARAP3	
CB_014522	3.44E-06	4.1E-08	5.79221	down	down	7025	NR2F1	
CB_006620	3.38E-06	3.98E-08	2.979873	down	down	10217	CTDSPL	
CB_021227	3.36E-06	3.92E-08	2.274564	down	down	58190	CTDSP1	
CB_027319	3.3E-06	3.81E-08	3.357248	down	down	23492	CBX7	
CB_011032	3.28E-06	3.76E-08	2.34828	down	down	2701	GJA4	
CB_015583	3.28E-06	3.74E-08	6.611006	down	down	2354	FOSB	
CB_015132	3.27E-06	3.69E-08	4.805563	down	down	7035	TFPI	
CB_005717	3.11E-06	3.45E-08	6.383288	down	down	4129	MAOB	
CB_004849	3.04E-06	3.34E-08	5.481324	down	down	154	ADRB2	
CB_010904	3.03E-06	3.27E-08	3.4888	down	down	1805	DPT	
CB_027881	3.03E-06	3.27E-08	6.088508	down	down	342035	GLDN	
CB_022112	2.75E-06	2.86E-08	2.49897	down	down	79443	FYCO1	
CB_028688	2.67E-06	2.73E-08	2.70507	down	down	10252	SPRY1	
CB_018266	2.6E-06	2.62E-08	4.982506	down	down	4616	GADD45B	
CB_006743	2.59E-06	2.6E-08	6.695981	down	down	126669	SHE	
CB_005215	2.55E-06	2.54E-08	4.941078	down	down	7078	TIMP3	
CB_020695	2.54E-06	2.52E-08	11.367	down	down	9457	FHL5	
CB_007892	2.53E-06	2.51E-08	2.823395	down	down	51092	SIDT2	
CB_013022	2.46E-06	2.4E-08	6.056549	down	down	2791	GNG11	
CB_014927	2.31E-06	2.19E-08	4.137645	down	down	10370	CITED2	
CB_025563	2.31E-06	2.15E-08	15.443	down	down	157310	PEBP4	
CB_004485	2.24E-06	2.07E-08	3.318897	down	down	58494	JAM2	
CB_007274	2.18E-06	1.96E-08	4.96558	down	down	7048	TGFBR2	
CB_011235	2.07E-06	1.79E-08	3.373568	down	down	3791	KDR	
CB_028824	1.98E-06	1.7E-08	3.264174	down	down	966	CD59	
CB_009616	1.96E-06	1.67E-08	4.244596	down	down	55679	LIMS2	
CB_018632	1.87E-06	1.56E-08	5.827085	down	down	389136	VGLL3	
CB_005493	1.84E-06	1.51E-08	20.36599	down	down	125	ADH1B	
CB_026214	1.82E-06	1.49E-08	2.231548	down	down	152273	FGD5	
CB_007481	1.82E-06	1.49E-08	2.50533	down	down	79148	MMP28	
CB_003631	1.77E-06	1.42E-08	19.70096	down	down	6332	SCN7A	
CB_021640	1.72E-06	1.35E-08	5.923054	down	down	64321	SOX17	
CB_028208	1.71E-06	1.34E-08	2.652585	down	down	8848	TSC22D1	
CB_024132	1.67E-06	1.28E-08	5.923923	down	down	94274	PPP1R14A	
CB_022389	1.49E-06	1.09E-08	2.858347	down	down	79864	C11orf63	
CB_010346	1.48E-06	1.07E-08	3.260622	down	down	1519	CTSO	
CB_011866	1.39E-06	9.82E-09	3.890243	down	down	5939	RBMS2	
CB_005433	1.38E-06	9.45E-09	7.571316	down	down	730	C7	
CB_015116	1.37E-06	9.3E-09	3.080484	down	down	6237	RRAS	
CB_021374	1.36E-06	9.14E-09	4.357505	down	down	1073	CFL2	
CB_020472	1.28E-06	8.23E-09	3.0076	down	down	56929	FEM1C	
CB_023077	1.28E-06	8.21E-09	3.441241	down	down	83547	RILP	

CB_020923	1.27E-06	8.11E-09	3.967058	down	down	57608	KIAA1462	
CB_016994	1.26E-06	7.94E-09	4.657599	down	down	23645	PPP1R15A	
CB_014117	1.25E-06	7.74E-09	8.111445	down	down	2353	FOS	
CB_020677	1.22E-06	7.42E-09	5.637226	down	down	57211	GPR126	
CB_015312	1.2E-06	7.05E-09	2.3441	down	down	10608	MXD4	
CB_013349	1.18E-06	6.84E-09	32.47904	down	down	2277	FIGF	
CB_014171	1.16E-06	6.61E-09	4.655263	down	down	2869	GRK5	
CB_028859	1.15E-06	6.45E-09	6.583248	down	down	92162	TMEM88	
CB_011117	1.15E-06	6.36E-09	10.10976	down	down	3164	NR4A1	
CB_016161	1.14E-06	6.3E-09	4.506548	down	down	22918	CD93	
CB_006694	1.1E-06	5.73E-09	3.397636	down	down	152503	SH3D19	
CB_027236	1.08E-06	5.55E-09	16.40779	down	down	199675	C19orf59	
CB_005772	1.05E-06	5.34E-09	5.358245	down	down	5730	PTGDS	
CB_020276	1E-06	4.97E-09	7.399662	down	down	54538	ROBO4	
CB_017198	9.93E-07	4.91E-09	3.754067	down	down	29995	LMCD1	
CB_008463	9.66E-07	4.6E-09	8.358195	down	down	221303	FAM162B	
CB_010844	9.06E-07	4.13E-09	4.823332	down	down	1346	COX7A1	
CB_010546	9.05E-07	4.07E-09	3.066039	down	down	3340	NDST1	
CB_017212	9.05E-07	4.07E-09	3.322599	down	down	30846	EHD2	
CB_024608	7.59E-07	3.23E-09	4.021616	down	down	23460	ABCA6	
CB_012580	7.58E-07	3.19E-09	10.29657	down	down	8516	ITGA8	
CB_005690	7.55E-07	3.1E-09	3.465288	down	down	3384	ICAM2	
CB_010899	6.95E-07	2.72E-09	8.984638	down	down	1675	CFD	
CB_009025	6.77E-07	2.53E-09	4.908627	down	down	7122	CLDN5	
CB_012920	6.65E-07	2.46E-09	7.270796	down	down	1910	EDNRB	
CB_018937	6.63E-07	2.43E-09	3.43324	down	down	51294	PCDH12	
CB_012665	5.86E-07	1.72E-09	13.02982	down	down	8639	AOC3	
CB_014201	5.67E-07	1.62E-09	2.388244	down	down	3092	HIP1	
CB_010736	5.5E-07	1.52E-09	4.125537	down	down	847	CAT	
CB_025441	5.46E-07	1.49E-09	9.648387	down	down	126393	HSPB6	
CB_011191	5.34E-07	1.42E-09	4.153154	down	down	3680	ITGA9	
CB_010233	5.09E-07	1.31E-09	4.285867	down	down	687	KLF9	
CB_020360	4.71E-07	1.16E-09	10.92001	down	down	56241	SUSD2	
CB_023841	4.71E-07	1.14E-09	10.35737	down	down	84913	ATOH8	
CB_010738	4.56E-07	1.03E-09	6.566709	down	down	857	CAV1	
CB_027288	4.2E-07	8.72E-10	5.853621	down	down	161198	CLEC14A	
CB_010664	4.02E-07	8.04E-10	4.468179	down	down	482	ATP1B2	
CB_017354	4.02E-07	7.97E-10	3.190133	down	down	9754	STARD8	
CB_018814	3.97E-07	7.8E-10	5.199345	down	down	51751	HIGD1B	
CB_011592	3.83E-07	7.41E-10	3.552465	down	down	5187	PER1	
CB_005541	3.29E-07	5.75E-10	8.46483	down	down	762	CA4	
CB_015184	3.03E-07	4.8E-10	5.964	down	down	10516	FBLN5	
CB_027566	2.75E-07	4.12E-10	6.196846	down	down	6711	SPTBN1	
CB_026585	2.75E-07	4.17E-10	18.02492	down	down	256691	MAMDC2	
CB_010181	2.72E-07	3.88E-10	7.041021	down	down	11185	INMT	
CB_016309	2.13E-07	2.81E-10	3.40725	down	down	284119	PTRF	
CB_028429	1.39E-07	1.41E-10	4.177772	down	down	256949	KANK3	
CB_005313	1.26E-07	1.16E-10	6.773453	down	down	6928	HNF1B	
CB_000273	1.23E-07	1.1E-10	4.21531	down	down	11030	RBPMS	
CB_009597	9.74E-08	8.01E-11	15.78845	down	down	2273	FHL1	
CB_006957	9.59E-08	7.6E-11	7.258698	down	down	219348	PLAC9	
CB_011377	7.96E-08	5.33E-11	13.81932	down	down	4239	MFAP4	
CB_012236	7.89E-08	5.13E-11	24.25688	down	down	7123	CLEC3B	
CB_009378	6.83E-08	3.31E-11	3.661793	down	down	283	ANG	
CB_028041	6.83E-08	3.47E-11	7.223291	down	down	10395	DLC1	
CB_011799	6.76E-08	3.11E-11	9.523437	down	down	5787	PTPRB	

CB_022293	6.39E-08	2.74E-11	2.296748	down	down	79758	DHRS12	
CB_020944	6.39E-08	2.73E-11	3.75161	down	down	57631	LRCH2	
CB_010473	6.22E-08	2.37E-11	10.0653	down	down	2327	FMO2	
CB_018770	6.22E-08	2.02E-11	16.03716	down	down	51208	CLDN18	
CB_026831	4.64E-08	7.36E-12	6.275165	down	down	130271	PLEKHH2	
CB_018685	3.91E-08	4.16E-12	6.31802	down	down	10365	KLF2	
CB_004966	0.049492	0.025222	2.22961	down	down	2162	F13A1	
CB_023527	0.048955	0.024867	2.175393	down	down	84332	DYDC2	
CB_005460	0.048369	0.024482	2.029975	down	down	3479	IGF1	
CB_027392	0.046904	0.023563	2.278992	down	down	2259	FGF14	
CB_014887	0.045955	0.022974	2.263831	down	down	9956	HS3ST2	
CB_017875	0.044574	0.022124	2.174215	down	down	158471	PRUNE2	
CB_025616	0.037455	0.017747	2.70592	down	down	219670	ENKUR	
CB_018743	0.035798	0.016757	2.1453	down	down	5625	PRODH	
CB_026880	0.03292	0.015045	3.844723	down	down	9576	SPAG6	
CB_024501	0.031414	0.014155	3.214203	down	down	83659	TEKT1	
CB_011354	0.030495	0.013621	2.714312	down	down	4133	MAP2	
CB_010524	0.026493	0.011348	2.477446	down	down	3036	HAS1	
CB_013848	0.025344	0.010724	2.150829	down	down	3382	ICA1	
CB_019009	0.023764	0.009863	2.110193	down	down	8863	PER3	
CB_013123	0.022416	0.009129	2.145002	down	down	9332	CD163	
CB_008313	0.022194	0.009011	2.276197	down	down	22996	TTC39A	
CB_009972	0.022016	0.008915	2.046925	down	down	83543	AIF1L	
CB_017091	0.019461	0.007578	2.336707	down	down	53832	IL20RA	
CB_011815	0.018927	0.007293	2.792615	down	down	5799	PTPRN2	
CB_009674	0.017742	0.006707	2.065297	down	down	730112	FAM166B	
CB_024697	0.017269	0.006467	2.37293	down	down	140733	MACROD2	
CB_023918	0.017	0.006333	2.021805	down	down	85016	C11orf70	
CB_016670	0.016988	0.006327	2.532478	down	down	26577	PCOLCE2	
CB_024907	0.015395	0.005551	2.675733	down	down	171024	SYNPO2	
CB_026225	0.014792	0.005262	2.336516	down	down	153643	FAM81B	
CB_022686	0.014678	0.00521	2.249172	down	down	80310	PDGFD	
CB_019818	0.013515	0.004666	2.019394	down	down	55259	CASC1	
CB_012720	0.013458	0.00464	2.003401	down	down	8727	CTNNAL1	
CB_005574	0.013269	0.004552	3.085668	down	down	1440	CSF3	
CB_021229	0.01282	0.004335	2.336635	down	down	58473	PLEKHB1	
CB_017067	0.012793	0.004323	3.077363	down	down	27092	CACNG4	
CB_025810	0.012754	0.004306	3.833849	down	down	143662	MUC15	
CB_013287	0.012463	0.004172	2.034156	down	down	1827	RCAN1	
CB_006649	0.012212	0.004058	2.20691	down	down	6542	SLC7A2	
CB_028405	0.012179	0.004043	2.080279	down	down	127845	GOLT1A	
CB_010754	0.012132	0.004021	2.543979	down	down	933	CD22	
CB_022732	0.012131	0.00402	2.706585	down	down	80736	SLC44A4	
CB_007846	0.011544	0.003757	2.146875	down	down	122060	SLAIN1	
CB_022542	0.011379	0.003685	2.683921	down	down	80059	LRRTM4	
CB_030740	0.010025	0.003107	3.290925	down	down	55350	VNN3	
CB_009807	0.009831	0.003023	2.128976	down	down	783	CACNB2	
CB_011096	0.009797	0.003008	2.179103	down	down	3109	HLA-DMB	
CB_011538	0.009757	0.002992	2.095832	down	down	5027	P2RX7	
CB_014922	0.009685	0.002962	2.107173	down	down	3268	AGFG2	
CB_010798	0.009675	0.002957	2.922033	down	down	1084	CEACAM3	
CB_004902	0.009383	0.002838	2.590904	down	down	718	C3	
CB_006004	0.009169	0.002748	2.143166	down	down	721	C4B	
CB_017572	0.008692	0.002552	3.936353	down	down	22866	CNKSR2	
CB_016099	0.008421	0.002443	6.586608	down	down	1755	DMBT1	
CB_027872	0.008001	0.002275	2.284342	down	down	135112	NCOA7	

CB_022915	0.007803	0.002197	2.042092	down	down	81615	TMEM163	
CB_025381	0.007745	0.002173	2.126628	down	down	90527	DUOXA1	
CB_007900	0.007247	0.001977	2.116843	down	down	83988	NCALD	
CB_026339	0.007226	0.00197	2.308027	down	down	202309	GAPT	
CB_012031	0.007067	0.001909	2.171452	down	down	6539	SLC6A12	
CB_007801	0.007067	0.001909	2.344434	down	down	129049	SGSM1	
CB_020362	0.006965	0.001871	2.347276	down	down	56253	CRTAM	
CB_003258	0.006897	0.001845	2.21966	down	down	4916	NTRK3	
CB_014133	0.006463	0.001685	2.38601	down	down	2702	GJA5	
CB_011939	0.006443	0.001678	2.195103	down	down	6281	S100A10	
CB_005781	0.006325	0.001638	2.957884	down	down	5743	PTGS2	
CB_026230	0.006151	0.001574	2.095546	down	down	154075	SAMD3	
CB_019860	0.006122	0.001564	2.000907	down	down	55303	GIMAP4	
CB_026796	0.006028	0.001529	2.85965	down	down	3772	KCNJ15	
CB_005709	0.005917	0.00149	2.360464	down	down	3762	KCNJ5	
CB_021323	0.005875	0.001475	2.300791	down	down	9848	MFAP3L	
CB_011170	0.005862	0.001469	2.477651	down	down	3575	IL7R	
CB_010267	0.005667	0.001399	2.152633	down	down	968	CD68	
CB_026582	0.0056	0.001379	3.097861	down	down	256076	COL6A5	
CB_023099	0.005567	0.001366	2.189672	down	down	83641	FAM107B	
CB_012903	0.00554	0.001357	2.594113	down	down	9172	MYOM2	
CB_015255	0.005521	0.00135	2.442909	down	down	10529	NEBL	
CB_011102	0.005453	0.001328	2.116181	down	down	3115	HLA-DPB1	
CB_005889	0.005448	0.001327	2.038024	down	down	6876	TAGLN	
CB_005472	0.005437	0.001322	2.06872	down	down	3684	ITGAM	
CB_017651	0.005222	0.001253	2.172204	down	down	23007	PLCH1	
CB_016131	0.00497	0.001168	2.44075	down	down	22914	KLRK1	
CB_005501	0.004836	0.001124	2.172969	down	down	135	ADORA2A	
CB_012200	0.004826	0.001121	2.736583	down	down	7042	TGFB2	
CB_019216	0.004798	0.001112	2.608832	down	down	55607	PPP1R9A	
CB_007467	0.004675	0.001072	2.189677	down	down	224	ALDH3A2	
CB_015848	0.00461	0.001051	2.27588	down	down	11076	TPPP	
CB_012590	0.004604	0.001049	2.050605	down	down	8530	CST7	
CB_023348	0.004559	0.001036	2.821291	down	down	92126	DSEL	
CB_019912	0.004531	0.001026	2.032449	down	down	55340	GIMAP5	
CB_011649	0.004463	0.001005	3.213783	down	down	5409	PNMT	
CB_020435	0.004463	0.001005	2.048188	down	down	56895	AGPAT4	
CB_019072	0.004341	0.000968	2.219058	down	down	10458	BAIAP2	
CB_014921	0.003938	0.000846	2.051987	down	down	10346	TRIM22	
CB_016889	0.003938	0.000846	2.204504	down	down	4071	TM4SF1	
CB_021553	0.003885	0.000831	2.078562	down	down	64098	PARVG	
CB_014621	0.003786	0.000801	2.680864	down	down	10157	AASS	
CB_013833	0.003767	0.000795	2.676566	down	down	2115	ETV1	
CB_009577	0.003762	0.000793	3.194486	down	down	287	ANK2	
CB_021261	0.003751	0.00079	5.612428	down	down	58511	DNASE2B	
CB_013002	0.003725	0.000782	3.580418	down	down	2170	FABP3	
CB_017850	0.003718	0.00078	3.63982	down	down	23242	COBL	
CB_023705	0.003705	0.000776	2.297805	down	down	84689	MS4A14	
CB_005416	0.003678	0.000768	2.228447	down	down	3047	HBG1	
CB_010230	0.003661	0.000764	2.638466	down	down	652	BMP4	
CB_007805	0.003599	0.000746	2.240853	down	down	2852	GPER	
CB_008828	0.003504	0.000719	2.286112	down	down	117	ADCYAP1R1	
CB_023188	0.003494	0.000717	2.676858	down	down	83853	ROPN1L	
CB_028281	0.003489	0.000715	2.220079	down	down	64581	CLEC7A	
CB_028871	0.003434	0.0007	2.035913	down	down	81704	DOCK8	
CB_014106	0.003419	0.000695	2.480688	down	down	2114	ETS2	

CB_021876	0.003418	0.000695	2.373529	down	down	10129	FRY	
CB_012897	0.003354	0.000678	2.388314	down	down	9034	CCRL2	
CB_011991	0.003315	0.000667	2.12113	down	down	6415	SEPW1	
CB_024535	0.003288	0.00066	2.049455	down	down	56062	KLHL4	
CB_010196	0.003252	0.000649	2.124914	down	down	60401	EDA2R	
CB_013232	0.003196	0.000634	2.881913	down	down	862	RUNX1T1	
CB_016619	0.003185	0.00063	2.316896	down	down	29799	YPEL1	
CB_017817	0.003134	0.000616	2.731523	down	down	23209	MLC1	
CB_011313	0.003124	0.000613	2.029069	down	down	4035	LRP1	
CB_008387	0.00301	0.000583	2.257837	down	down	728621	CCDC30	
CB_026776	0.002941	0.000564	2.005329	down	down	9462	RASAL2	
CB_009368	0.002907	0.000555	2.336616	down	down	23327	NEDD4L	
CB_006481	0.002901	0.000553	3.130405	down	down	785	CACNB4	
CB_021821	0.002901	0.000553	3.229087	down	down	64881	PCDH20	
CB_011275	0.002822	0.000533	2.122701	down	down	3903	LAIR1	
CB_006523	0.002786	0.000523	2.144717	down	down	114789	SLC25A25	
CB_015917	0.002772	0.000519	2.129809	down	down	7376	NR1H2	
CB_004904	0.002754	0.000514	3.148567	down	down	729	C6	
CB_020486	0.002742	0.000511	2.155413	down	down	56944	OLFML3	
CB_012930	0.002711	0.000503	2.571205	down	down	1756	DMD	
CB_018130	0.00269	0.000498	2.219502	down	down	25976	TIPARP	
CB_013156	0.002647	0.000487	2.63902	down	down	9481	SLC25A27	
CB_008617	0.002643	0.000486	2.098436	down	down	129852	C2orf73	
CB_024276	0.002606	0.000476	2.094867	down	down	3113	HLA-DPA1	
CB_007081	0.002586	0.000471	2.300489	down	down	10873	ME3	
CB_017579	0.002582	0.00047	2.143056	down	down	22874	PLEKHA6	
CB_019784	0.002574	0.000468	2.363691	down	down	55244	SLC47A1	
CB_005399	0.002551	0.000462	14.4483	down	down	6439	SFTPB	
CB_023153	0.002546	0.00046	2.844156	down	down	56135	PCDHAC1	
CB_025468	0.002469	0.000442	2.123161	down	down	137835	TMEM71	
CB_012252	0.002424	0.00043	4.050888	down	down	7177	TPSAB1	
CB_009608	0.002418	0.000429	2.222712	down	down	1806	DPYD	
CB_024140	0.002321	0.000405	2.194065	down	down	153572	IRX2	
CB_016219	0.002284	0.000396	2.577345	down	down	23576	DDAH1	
CB_025571	0.002245	0.000386	2.023187	down	down	158326	FREM1	
CB_005360	0.002229	0.000383	4.481758	down	down	2266	FGG	
CB_011909	0.002205	0.000377	2.533769	down	down	6092	ROBO2	
CB_004955	0.002188	0.000373	2.137018	down	down	2052	EPHX1	
CB_013539	0.002139	0.000362	2.932221	down	down	8825	LIN7A	
CB_013000	0.002029	0.000336	2.936929	down	down	2070	EYA4	
CB_011150	0.002019	0.000334	2.013444	down	down	3398	ID2	
CB_008172	0.002009	0.000332	2.496226	down	down	368	ABCC6	
CB_022824	0.001995	0.000329	2.023112	down	down	80709	AKNA	
CB_024702	0.001978	0.000325	2.285282	down	down	5783	PTPN13	
CB_021131	0.001973	0.000324	2.346	down	down	6546	SLC8A1	
CB_017126	0.001948	0.000319	2.390333	down	down	27295	PDLIM3	
CB_015721	0.001899	0.000307	2.008843	down	down	11035	RIPK3	
CB_018202	0.001894	0.000306	2.357385	down	down	26084	ARHGEF26	
CB_028825	0.001863	0.0003	2.909561	down	down	1191	CLU	
CB_005040	0.001852	0.000297	2.845138	down	down	3426	CFI	
CB_020162	0.001831	0.000292	2.083409	down	down	27115	PDE7B	
CB_017580	0.001827	0.000292	2.615369	down	down	22875	ENPP4	
CB_019900	0.001816	0.000289	2.041067	down	down	55790	CSGALNACT1	
CB_030773	0.001811	0.000288	2.537884	down	down	1E+08	ARMCX4	
CB_027865	0.001801	0.000286	2.095817	down	down	3290	HSD11B1	
CB_005725	0.001792	0.000283	3.810394	down	down	4883	NPR3	

CB_022196	0.00179	0.000283	2.010263	down	down	79661	NEIL1	
CB_008937	0.001725	0.000269	2.142638	down	down	1E+08	ZNF717	
CB_021269	0.001721	0.000268	2.299096	down	down	58526	MID1IP1	
CB_005778	0.0017	0.000263	2.794995	down	down	5740	PTGIS	
CB_004901	0.001652	0.000253	2.008118	down	down	717	C2	
CB_023402	0.001639	0.000251	2.252908	down	down	84187	TMEM164	
CB_026882	0.001628	0.000248	2.181727	down	down	1438	CSF2RA	
CB_007336	0.001589	0.00024	2.206042	down	down	83478	ARHGAP24	
CB_008932	0.001542	0.000231	3.265558	down	down	7068	THRB	
CB_006787	0.00154	0.00023	2.766742	down	down	345895	RSPH4A	
CB_001393	0.001502	0.000222	2.449952	down	down	6919	TCEA2	
CB_018105	0.00149	0.00022	2.278962	down	down	25937	WWTR1	
CB_026627	0.001468	0.000215	2.841764	down	down	167681	PRSS35	
CB_018352	0.001446	0.00021	2.516923	down	down	51364	ZMYND10	
CB_022638	0.001431	0.000207	2.221518	down	down	80221	ACSF2	
CB_008394	0.001431	0.000207	2.12145	down	down	139716	GAB3	
CB_021168	0.001415	0.000203	2.157734	down	down	6252	RTN1	
CB_015842	0.001403	0.000201	2.255297	down	down	11069	RAPGEF4	
CB_020698	0.001379	0.000197	2.052604	down	down	57326	PBXIP1	
CB_014464	0.001376	0.000196	2.052527	down	down	5010	CLDN11	
CB_029676	0.001371	0.000195	2.099927	down	down	387751	GVINP1	
CB_027326	0.001333	0.000187	2.268204	down	down	3568	IL5RA	
CB_014239	0.00132	0.000185	2.101032	down	down	4783	NFIL3	
CB_015763	0.001308	0.000183	2.123251	down	down	6515	SLC2A3	
CB_004993	0.001308	0.000183	2.083454	down	down	2581	GALC	
CB_008964	0.001307	0.000183	2.17503	down	down	775	CACNA1C	
CB_006082	0.001286	0.000179	2.097673	down	down	5136	PDE1A	
CB_025547	0.001279	0.000177	2.096357	down	down	153830	RNF145	
CB_005989	0.001274	0.000176	2.926335	down	down	220965	FAM13C	
CB_015806	0.001255	0.000173	2.122043	down	down	8510	MMP23B	
CB_021634	0.001254	0.000173	2.006862	down	down	27010	TPK1	
CB_022302	0.001253	0.000172	2.034915	down	down	79772	MCTP1	
CB_009209	0.001229	0.000168	2.053774	down	down	9208	LRRFIP1	
CB_026986	0.001225	0.000167	2.349384	down	down	143098	MPP7	
CB_014264	0.001217	0.000165	19.5877	down	down	653509	SFTPA1	
CB_007983	0.001208	0.000164	2.310858	down	down	913	CD1E	
CB_017235	0.001184	0.000159	2.565283	down	down	6277	S100A6	
CB_028569	0.001104	0.000145	2.511027	down	down	375449	MAST4	
CB_009313	0.001103	0.000145	5.797789	down	down	388743	CAPN8	
CB_011857	0.001089	0.000142	2.104386	down	down	5919	RARRES2	
CB_013923	0.001069	0.000139	2.217876	down	down	5551	PRF1	
CB_016388	0.001046	0.000135	2.847447	down	down	22941	SHANK2	
CB_012756	0.001041	0.000134	2.505211	down	down	8778	SIGLEC5	
CB_012202	0.001026	0.000131	4.649083	down	down	7044	LEFTY2	
CB_024529	0.001011	0.000128	2.371403	down	down	117289	TAGAP	
CB_006139	0.001008	0.000128	2.016735	down	down	5768	QSOX1	
CB_020931	0.001001	0.000127	2.846926	down	down	57619	SHROOM3	
CB_011245	0.000995	0.000126	2.25069	down	down	3824	KLRD1	
CB_013460	0.000983	0.000124	2.05156	down	down	5873	RAB27A	
CB_012875	0.000982	0.000123	2.545177	down	down	8987	STBD1	
CB_021448	0.000939	0.000116	2.763933	down	down	53826	FXYD6	
CB_026781	0.000925	0.000114	2.347152	down	down	4000	LMNA	
CB_015294	0.000921	0.000113	2.352602	down	down	10577	NPC2	
CB_008029	0.000912	0.000112	3.480228	down	down	4582	MUC1	
CB_000213	0.000912	0.000111	2.026226	down	down	441108	C5orf56	
CB_005250	0.000878	0.000106	2.263131	down	down	1959	EGR2	

CB_022963	0.000867	0.000104	3.570929	down	down	81849	ST6GALNAC5	
CB_008186	0.000812	9.51E-05	2.471519	down	down	114548	NLRP3	
CB_007378	0.000786	9.12E-05	5.979641	down	down	200373	PCDP1	
CB_011098	0.000779	9.02E-05	2.388027	down	down	3111	HLA-DOA	
CB_007474	0.000779	9.01E-05	4.591641	down	down	5648	MASP1	
CB_008831	0.000777	8.99E-05	2.127986	down	down	123	PLIN2	
CB_018113	0.000777	8.99E-05	2.309025	down	down	25945	PVRL3	
CB_008465	0.000772	8.9E-05	2.304901	down	down	284417	TMEM150B	
CB_010349	0.00077	8.88E-05	3.786575	down	down	1524	CX3CR1	
CB_004905	0.000765	8.81E-05	3.164545	down	down	732	C8B	
CB_005744	0.000759	8.69E-05	3.265087	down	down	5243	ABCB1	
CB_009494	0.000747	8.51E-05	2.604009	down	down	84525	HOPX	
CB_015048	0.000733	8.31E-05	2.905399	down	down	5168	ENPP2	
CB_021119	0.00072	8.11E-05	3.288668	down	down	6565	SLC15A2	
CB_018697	0.000716	8.05E-05	2.009441	down	down	51347	TAOK3	
CB_012046	0.000714	8.02E-05	3.781264	down	down	6588	SLN	
CB_010760	0.00071	7.94E-05	2.158747	down	down	953	ENTPD1	
CB_018972	0.000708	7.92E-05	2.933513	down	down	51316	PLAC8	
CB_015693	0.000693	7.68E-05	3.178389	down	down	10990	LILRB5	
CB_012434	0.000692	7.67E-05	2.33568	down	down	8291	DYSF	
CB_026725	0.000688	7.6E-05	2.136057	down	down	79683	ZDHHC14	
CB_020080	0.000675	7.42E-05	2.039939	down	down	55901	THSD1	
CB_008190	0.000673	7.38E-05	2.302613	down	down	10149	GPR64	
CB_020571	0.000671	7.35E-05	2.248707	down	down	57186	RALGAPA2	
CB_005471	0.000667	7.29E-05	2.006513	down	down	4689	NCF4	
CB_016796	0.000667	7.28E-05	2.139822	down	down	57496	MKL2	
CB_017885	0.000658	7.13E-05	2.370287	down	down	23286	WWC1	
CB_024333	0.000653	7.07E-05	3.192415	down	down	92304	SCGB3A1	
CB_004896	0.000652	7.05E-05	2.173908	down	down	695	BTK	
CB_010734	0.000651	7.04E-05	2.122483	down	down	824	CAPN2	
CB_009013	0.00064	6.87E-05	2.229488	down	down	2982	GUCY1A3	
CB_024964	0.000631	6.75E-05	2.413799	down	down	154661	RUNDC3B	
CB_015717	0.00063	6.74E-05	2.321778	down	down	11027	LILRA2	
CB_010634	0.000629	6.72E-05	19.08529	down	down	361	AQP4	
CB_025014	0.000627	6.7E-05	2.66041	down	down	91461	PKDCC	
CB_011517	0.000624	6.65E-05	3.484794	down	down	4950	OCLN	
CB_026932	0.000615	6.52E-05	5.452389	down	down	8013	NR4A3	
CB_020825	0.000615	6.51E-05	2.552683	down	down	57507	ZNF608	
CB_024433	0.000597	6.26E-05	2.151583	down	down	116496	FAM129A	
CB_028562	0.00059	6.16E-05	2.037728	down	down	961	CD47	
CB_010964	0.00059	6.15E-05	2.243893	down	down	2192	FBLN1	
CB_006069	0.00058	6.02E-05	2.743193	down	down	3983	ABLIM1	
CB_018071	0.000568	5.85E-05	2.03825	down	down	25898	RCHY1	
CB_027124	0.000565	5.81E-05	3.56132	down	down	285755	PPIL6	
CB_019951	0.000551	5.6E-05	2.013528	down	down	55356	SLC22A15	
CB_026589	0.000545	5.52E-05	2.035777	down	down	257364	SNX33	
CB_007565	0.00054	5.45E-05	2.570388	down	down	30820	KCNIP1	
CB_007302	0.000521	5.2E-05	2.280404	down	down	972	CD74	
CB_025557	0.000515	5.12E-05	5.066392	down	down	8796	SCEL	
CB_012746	0.000515	5.12E-05	2.024075	down	down	8764	TNFRSF14	
CB_014661	0.000511	5.07E-05	6.089485	down	down	10216	PRG4	
CB_023136	0.000509	5.04E-05	2.254991	down	down	56145	PCDHA3	
CB_018432	0.000497	4.89E-05	2.477516	down	down	712	C1QA	
CB_009805	0.000491	4.8E-05	2.13329	down	down	93664	CADPS2	
CB_014833	0.000487	4.74E-05	3.009381	down	down	6909	TBX2	
CB_014438	0.000485	4.72E-05	2.438157	down	down	4005	LMO2	

CB_007515	0.000483	4.69E-05	3.025525	down	down	255743	NPNT	
CB_005280	0.000483	4.69E-05	2.457511	down	down	3908	LAMA2	
CB_021442	0.000464	4.45E-05	2.017266	down	down	26301	GBGT1	
CB_014011	0.000451	4.28E-05	5.887806	down	down	306	ANXA3	
CB_014996	0.000447	4.23E-05	3.671006	down	down	4778	NFE2	
CB_008477	0.000443	4.17E-05	2.473043	down	down	79839	CCDC102B	
CB_028381	0.000438	4.1E-05	2.849929	down	down	4013	VWA5A	
CB_014671	0.000432	4.03E-05	13.9752	down	down	10232	MSLN	
CB_025267	0.000431	4.02E-05	2.076781	down	down	146722	CD300LF	
CB_024402	0.000431	4.02E-05	3.087214	down	down	115019	SLC26A9	
CB_020848	0.000425	3.95E-05	3.754093	down	down	57530	CGN	
CB_009229	0.000423	3.92E-05	2.564853	down	down	247	ALOX15B	
CB_008511	0.00042	3.89E-05	3.991261	down	down	23705	CADM1	
CB_012658	0.000419	3.88E-05	2.192851	down	down	8633	UNC5C	
CB_012760	0.000414	3.82E-05	2.141926	down	down	8787	RGS9	
CB_025475	0.000406	3.71E-05	2.228808	down	down	139818	DOCK11	
CB_009746	0.000406	3.71E-05	2.673681	down	down	5144	PDE4D	
CB_015351	0.000405	3.7E-05	2.308927	down	down	4162	MCAM	
CB_016030	0.000402	3.67E-05	2.510481	down	down	11264	PXMP4	
CB_024553	0.0004	3.64E-05	2.048697	down	down	118429	ANTXR2	
CB_017301	0.000399	3.62E-05	2.592114	down	down	9706	ULK2	
CB_010163	0.000396	3.57E-05	2.338227	down	down	3120	HLA-DQB2	
CB_014960	0.000395	3.56E-05	2.228267	down	down	3108	HLA-DMA	
CB_025117	0.000392	3.53E-05	2.620384	down	down	7099	TLR4	
CB_021338	0.000391	3.51E-05	5.706653	down	down	3170	FOXA2	
CB_009919	0.00039	3.5E-05	2.103589	down	down	3958	LGALS3	
CB_013822	0.000387	3.47E-05	2.907254	down	down	1794	DOCK2	
CB_026433	0.000385	3.43E-05	2.679785	down	down	222166	C7orf41	
CB_014651	0.000376	3.32E-05	3.581699	down	down	10203	CALCRL	
CB_023554	0.000375	3.32E-05	12.36495	down	down	84417	C2orf40	
CB_009182	0.000375	3.31E-05	3.673436	down	down	84879	MFSD2A	
CB_020007	0.000373	3.29E-05	2.077915	down	down	55862	ECHDC1	
CB_013697	0.000371	3.26E-05	7.720913	down	down	9413	FAM189A2	
CB_015715	0.000362	3.16E-05	2.502412	down	down	11025	LILRB3	
CB_011378	0.000359	3.12E-05	2.042907	down	down	4242	MFNG	
CB_007194	0.000356	3.09E-05	2.006915	down	down	2908	NR3C1	
CB_020016	0.000352	3.04E-05	5.842787	down	down	9496	TBX4	
CB_004935	0.000351	3.03E-05	2.216858	down	down	1471	CST3	
CB_017310	0.00035	3.01E-05	2.060134	down	down	9719	ADAMTSL2	
CB_006971	0.000343	2.93E-05	7.130062	down	down	401546	C9orf152	
CB_024507	0.000342	2.92E-05	8.666584	down	down	117156	SCGB3A2	
CB_012398	0.000336	2.85E-05	2.12835	down	down	7802	DNALI1	
CB_013547	0.00033	2.79E-05	2.191413	down	down	9064	MAP3K6	
CB_013153	0.000323	2.7E-05	4.001488	down	down	9472	AKAP6	
CB_014954	0.00032	2.67E-05	2.103252	down	down	10449	ACAA2	
CB_006036	0.000317	2.63E-05	3.142389	down	down	80223	RAB11FIP1	
CB_010353	0.000316	2.62E-05	2.440629	down	down	1601	DAB2	
CB_013870	0.000309	2.55E-05	2.377988	down	down	2122	MECOM	
CB_008509	0.000307	2.52E-05	2.143248	down	down	116535	MRGPRF	
CB_014372	0.000305	2.49E-05	2.444406	down	down	2615	LRRC32	
CB_014489	0.000303	2.48E-05	2.020598	down	down	6386	SDCBP	
CB_009462	0.0003	2.45E-05	3.42669	down	down	80726	KIAA1683	
CB_029153	0.000289	2.33E-05	2.058843	down	down	9619	ABCG1	
CB_023107	0.000284	2.27E-05	2.044804	down	down	89822	KCNK17	
CB_015065	0.000282	2.26E-05	3.433791	down	down	5334	PLCL1	
CB_025470	0.000281	2.25E-05	2.745697	down	down	137872	ADHFE1	

CB_014450	0.00028	2.24E-05	2.122665	down	down	4329	ALDH6A1	
CB_025805	0.00028	2.24E-05	2.163804	down	down	80833	APOL3	
CB_025575	0.00027	2.12E-05	4.72905	down	down	158866	ZDHHC15	
CB_012272	0.000268	2.1E-05	18.10842	down	down	7080	NKX2-1	
CB_009961	0.000266	2.08E-05	3.64232	down	down	57669	EPB41L5	
CB_013494	0.000265	2.07E-05	4.196791	down	down	7102	TSPAN7	
CB_016416	0.000254	1.96E-05	2.364609	down	down	4482	MSRA	
CB_008700	0.000253	1.95E-05	2.681615	down	down	91607	SLFN11	
CB_026862	0.000246	1.87E-05	2.093828	down	down	3603	IL16	
CB_007769	0.00024	1.81E-05	3.468837	down	down	642987	TMEM232	
CB_022167	0.000239	1.81E-05	3.169836	down	down	79632	FAM184A	
CB_019194	0.000233	1.74E-05	2.18928	down	down	55603	FAM46A	
CB_008926	0.000233	1.74E-05	2.652	down	down	6935	ZEB1	
CB_014455	0.000232	1.73E-05	2.871058	down	down	4774	NFIA	
CB_019503	0.000229	1.71E-05	2.297663	down	down	55031	USP47	
CB_010219	0.000229	1.7E-05	2.066064	down	down	4853	NOTCH2	
CB_008709	0.000227	1.68E-05	2.311268	down	down	3910	LAMA4	
CB_005427	0.000227	1.68E-05	4.414617	down	down	6556	SLC11A1	
CB_013493	0.00022	1.61E-05	2.087429	down	down	7084	TK2	
CB_006791	0.00022	1.61E-05	2.321541	down	down	387923	SERP2	
CB_021608	0.000219	1.6E-05	2.149881	down	down	9659	PDE4DIP	
CB_005470	0.000214	1.55E-05	2.049253	down	down	3454	IFNAR1	
CB_015222	0.000211	1.53E-05	2.206241	down	down	10486	CAP2	
CB_018141	0.00021	1.52E-05	2.52139	down	down	9223	MAGI1	
CB_010911	0.000209	1.51E-05	3.983847	down	down	1839	HBEGF	
CB_022699	0.000208	1.5E-05	3.497279	down	down	80332	ADAM33	
CB_029140	0.0002	1.42E-05	6.353263	down	down	344148	NCKAP5	
CB_026066	0.0002	1.42E-05	2.588361	down	down	127795	C1orf87	
CB_015049	0.000198	1.41E-05	2.383635	down	down	5178	PEG3	
CB_026218	0.000196	1.39E-05	2.063896	down	down	152926	PPM1K	
CB_016934	0.000195	1.38E-05	2.37793	down	down	10982	MAPRE2	
CB_012768	0.000195	1.37E-05	4.619525	down	down	8794	TNFRSF10C	
CB_024736	0.000194	1.36E-05	2.721204	down	down	1305	COL13A1	
CB_013755	0.000192	1.35E-05	3.047842	down	down	9452	ITM2A	
CB_005288	0.000192	1.34E-05	3.193554	down	down	4688	NCF2	
CB_015508	0.000192	1.34E-05	3.758268	down	down	10810	WASF3	
CB_026390	0.000192	1.34E-05	2.966039	down	down	222223	KIAA1324L	
CB_012038	0.000192	1.34E-05	3.058294	down	down	6571	SLC18A2	
CB_014907	0.000192	1.34E-05	3.167716	down	down	10319	LAMC3	
CB_009170	0.00019	1.33E-05	2.01193	down	down	93166	PRDM6	
CB_016886	0.000187	1.3E-05	2.123352	down	down	3705	ITPK1	
CB_022369	0.000186	1.29E-05	2.592668	down	down	79843	FAM124B	
CB_012906	0.000184	1.27E-05	2.602648	down	down	9046	DOK2	
CB_013445	0.000183	1.26E-05	2.204944	down	down	5209	PFKFB3	
CB_017684	0.000183	1.26E-05	2.062777	down	down	23051	ZHX3	
CB_012006	0.000182	1.26E-05	11.88666	down	down	6441	SFTPD	
CB_012755	0.00018	1.23E-05	2.172774	down	down	8777	MPDZ	
CB_008597	0.000178	1.22E-05	3.625342	down	down	55638	SYBU	
CB_023150	0.000177	1.21E-05	6.338257	down	down	56137	PCDHA12	
CB_026252	0.000176	1.2E-05	2.214198	down	down	159195	USP54	
CB_012129	0.000175	1.18E-05	2.151211	down	down	6776	STAT5A	
CB_028354	0.000175	1.18E-05	3.473926	down	down	6035	RNASE1	
CB_028074	0.000173	1.17E-05	2.387653	down	down	4681	NBL1	
CB_009830	0.000172	1.16E-05	3.41555	down	down	401124	DTHD1	
CB_008346	0.000171	1.15E-05	2.124245	down	down	57545	CC2D2A	
CB_020605	0.00017	1.14E-05	2.061857	down	down	57134	MAN1C1	

CB_014564	0.000169	1.13E-05	2.32638	down	down	10076	PTPRU	
CB_020081	0.000168	1.13E-05	2.732242	down	down	55902	ACSS2	
CB_017106	0.000165	1.1E-05	2.44271	down	down	27244	SESN1	
CB_029400	0.000165	1.1E-05	2.937172	down	down	7754	ZNF204P	
CB_005719	0.000164	1.09E-05	3.480587	down	down	4256	MGP	
CB_020758	0.000162	1.08E-05	2.433742	down	down	57406	ABHD6	
CB_027215	0.000162	1.07E-05	3.24155	down	down	128346	C1orf162	
CB_023137	0.000162	1.07E-05	2.364374	down	down	2793	GNGT2	
CB_024300	0.000149	9.64E-06	2.035366	down	down	90634	N4BP2L1	
CB_027908	0.000149	9.63E-06	2.536655	down	down	353514	LILRA5	
CB_029833	0.000148	9.56E-06	4.231149	down	down	56967	C14orf132	
CB_027540	0.000144	9.25E-06	2.028593	down	down	283349	RASSF3	
CB_014132	0.000142	9.06E-06	2.168103	down	down	2678	GGT1	
CB_028640	0.000142	9.03E-06	2.652943	down	down	91227	GGTLC2	
CB_018929	0.000141	8.97E-06	2.781375	down	down	6926	TBX3	
CB_022906	0.000138	8.75E-06	2.246986	down	down	81606	LBH	
CB_015105	0.000138	8.71E-06	3.182453	down	down	5592	PRKG1	
CB_020680	0.000137	8.62E-06	2.441463	down	down	57217	TTC7A	
CB_028460	0.000136	8.57E-06	3.533619	down	down	343450	KCNT2	
CB_026836	0.00013	8.1E-06	2.853474	down	down	8741	TNFSF13	
CB_012019	0.00013	8.09E-06	2.028635	down	down	6489	ST8SIA1	
CB_004926	0.00013	8.06E-06	3.714734	down	down	1286	COL4A4	
CB_008647	0.000129	7.97E-06	23.66072	down	down	253970	SFTA3	
CB_027407	0.000129	7.96E-06	7.25159	down	down	155465	AGR3	
CB_024943	0.000128	7.91E-06	2.09119	down	down	5998	RGS3	
CB_028778	0.000126	7.74E-06	2.625458	down	down	57447	NDRG2	
CB_010132	0.000124	7.56E-06	3.899778	down	down	4660	PPP1R12B	
CB_006440	0.000121	7.32E-06	3.316396	down	down	345557	PLCXD3	
CB_013821	0.00012	7.27E-06	6.943422	down	down	1776	DNASE1L3	
CB_028526	0.000119	7.15E-06	2.127726	down	down	376497	SLC27A1	
CB_016528	0.000118	7.12E-06	2.137043	down	down	23639	LRRC6	
CB_022811	0.000114	6.77E-06	2.103725	down	down	6934	TCF7L2	
CB_008824	0.000113	6.7E-06	2.896204	down	down	149483	CCDC17	
CB_028116	0.000112	6.61E-06	2.365626	down	down	11259	FILIP1L	
CB_006024	0.000111	6.54E-06	2.154857	down	down	79971	WLS	
CB_021600	0.000111	6.53E-06	8.699699	down	down	64168	NECAB1	
CB_005443	0.00011	6.5E-06	7.416318	down	down	3569	IL6	
CB_011985	0.00011	6.43E-06	3.205	down	down	6403	SELP	
CB_016076	0.000109	6.41E-06	4.043804	down	down	11343	MGLL	
CB_006805	0.000106	6.16E-06	2.551484	down	down	401494	PTPLAD2	
CB_013266	0.000104	6.02E-06	4.057541	down	down	1512	CTSH	
CB_012817	0.000104	5.99E-06	3.064575	down	down	8869	ST3GAL5	
CB_010971	0.000103	5.96E-06	3.404689	down	down	2219	FCN1	
CB_028769	0.000103	5.95E-06	2.304695	down	down	8522	GAS7	
CB_009664	0.000102	5.83E-06	2.279211	down	down	322	APBB1	
CB_011933	0.0001	5.74E-06	2.495922	down	down	6274	S100A3	
CB_005116	1E-04	5.7E-06	2.055357	down	down	4867	NPHP1	
CB_015441	9.77E-05	5.52E-06	4.765036	down	down	27233	SULT1C4	
CB_006732	9.72E-05	5.48E-06	2.213217	down	down	25943	C20orf194	
CB_009156	9.69E-05	5.44E-06	3.164484	down	down	26052	DNM3	
CB_004970	9.57E-05	5.36E-06	3.15674	down	down	2157	F8	
CB_012806	9.51E-05	5.32E-06	2.510681	down	down	8839	WISP2	
CB_027250	9.39E-05	5.23E-06	3.652154	down	down	254295	PHYHD1	
CB_009399	9.32E-05	5.15E-06	5.58922	down	down	387763	C11orf96	
CB_026506	9.24E-05	5.1E-06	2.329182	down	down	144165	PRICKLE1	
CB_005539	9.24E-05	5.09E-06	8.585255	down	down	722	C4BPA	

CB_024940	9.2E-05	5.07E-06	2.889519	down	down	3241	HPCAL1	
CB_008470	9.14E-05	5.02E-06	9.863714	down	down	21	ABCA3	
CB_008614	9.14E-05	5.01E-06	2.2624	down	down	9749	PHACTR2	
CB_018040	9.05E-05	4.95E-06	3.80042	down	down	25854	FAM149A	
CB_007877	9E-05	4.92E-06	2.136532	down	down	152098	ZCWPW2	
CB_028842	8.87E-05	4.81E-06	4.02731	down	down	51703	ACSL5	
CB_014756	8.83E-05	4.78E-06	2.330813	down	down	4209	MEF2D	
CB_006852	8.75E-05	4.72E-06	3.253138	down	down	84460	ZMAT1	
CB_026154	8.65E-05	4.64E-06	2.650363	down	down	146556	C16orf89	
CB_029009	8.57E-05	4.58E-06	2.525762	down	down	2549	GAB1	
CB_025198	8.55E-05	4.57E-06	3.981981	down	down	116441	TM4SF18	
CB_021409	8.51E-05	4.53E-06	2.11907	down	down	3142	HLX	
CB_015129	8.49E-05	4.52E-06	2.352222	down	down	6867	TACC1	
CB_026700	8.49E-05	4.52E-06	2.366448	down	down	91526	ANKRD44	
CB_013117	8.49E-05	4.52E-06	5.283647	down	down	9314	KLF4	
CB_010305	8.38E-05	4.44E-06	2.922969	down	down	1193	CLIC2	
CB_018279	8.38E-05	4.44E-06	2.964887	down	down	27147	DENND2A	
CB_006758	8.37E-05	4.43E-06	2.076299	down	down	170371	C10orf128	
CB_018225	8.26E-05	4.34E-06	2.328053	down	down	26112	CCDC69	
CB_018229	8.2E-05	4.29E-06	2.021043	down	down	26118	WSB1	
CB_010743	8.11E-05	4.22E-06	2.488576	down	down	896	CCND3	
CB_013607	8.07E-05	4.19E-06	2.092053	down	down	9185	REPS2	
CB_021635	7.96E-05	4.11E-06	6.410173	down	down	64284	RAB17	
CB_008569	7.96E-05	4.1E-06	2.601091	down	down	8910	SGCE	
CB_016059	7.93E-05	4.09E-06	4.239116	down	down	11326	VSIG4	
CB_013954	7.88E-05	4.05E-06	2.076908	down	down	6560	SLC12A4	
CB_007837	7.71E-05	3.94E-06	6.726773	down	down	4629	MYH11	
CB_015597	7.67E-05	3.91E-06	6.831242	down	down	5950	RBP4	
CB_028078	7.63E-05	3.88E-06	2.767161	down	down	255488	RNF144B	
CB_022145	7.61E-05	3.86E-06	4.553008	down	down	79611	ACSS3	
CB_028915	7.47E-05	3.76E-06	2.38473	down	down	389125	MUSTN1	
CB_023182	7.47E-05	3.76E-06	4.776586	down	down	114905	C1QTNF7	
CB_005084	7.41E-05	3.72E-06	2.199011	down	down	1.01E+08	MICA	
CB_004875	7.37E-05	3.68E-06	3.499363	down	down	367	AR	
CB_003310	7.3E-05	3.64E-06	3.445943	down	down	5137	PDE1C	
CB_025393	7.13E-05	3.52E-06	2.52791	down	down	91624	NEXN	
CB_025349	7.13E-05	3.52E-06	2.912723	down	down	203190	LGI3	
CB_017326	7.11E-05	3.49E-06	3.03701	down	down	9728	SECISBP2L	
CB_026709	6.97E-05	3.4E-06	2.795476	down	down	127435	PODN	
CB_005720	6.78E-05	3.27E-06	5.497245	down	down	4306	NR3C2	
CB_017549	6.7E-05	3.21E-06	2.871333	down	down	89795	NAV3	
CB_020915	6.66E-05	3.18E-06	2.605594	down	down	57600	FNIP2	
CB_007676	6.56E-05	3.12E-06	3.274279	down	down	11078	TRIOBP	
CB_019868	6.52E-05	3.09E-06	2.557445	down	down	54674	LRRN3	
CB_013004	6.52E-05	3.09E-06	4.451708	down	down	2202	EFEMP1	
CB_025193	6.52E-05	3.09E-06	2.308427	down	down	115548	FCHO2	
CB_008850	6.37E-05	2.99E-06	2.763543	down	down	284759	SIRPB2	
CB_013067	6.14E-05	2.84E-06	2.055578	down	down	6809	STX3	
CB_025018	6.14E-05	2.85E-06	3.517082	down	down	91768	CABLES1	
CB_027453	5.85E-05	2.64E-06	2.01542	down	down	23673	STX12	
CB_024982	5.84E-05	2.63E-06	3.753047	down	down	160364	CLEC12A	
CB_026203	5.78E-05	2.58E-06	9.386371	down	down	151126	ZNF385B	
CB_004925	5.73E-05	2.55E-06	3.282445	down	down	1285	COL4A3	
CB_026948	5.71E-05	2.55E-06	2.997246	down	down	317649	EIF4E3	
CB_022221	5.7E-05	2.54E-06	7.190983	down	down	79689	STEAP4	
CB_015040	5.55E-05	2.43E-06	2.813032	down	down	5141	PDE4A	

CB_021417	5.46E-05	2.37E-06	11.42384	down	down	5239	PGM5	
CB_013942	5.36E-05	2.32E-06	4.648439	down	down	6097	RORC	
CB_007107	5.36E-05	2.31E-06	3.550643	down	down	9060	PAPSS2	
CB_014913	5.3E-05	2.27E-06	2.349925	down	down	10326	SIRPB1	
CB_012238	5.27E-05	2.25E-06	3.56527	down	down	7134	TNNC1	
CB_004848	5.25E-05	2.24E-06	2.711662	down	down	6442	SGCA	
CB_006599	5.09E-05	2.14E-06	2.230779	down	down	10133	OPTN	
CB_026208	5.06E-05	2.11E-06	3.384542	down	down	117583	PARD3B	
CB_027338	4.96E-05	2.06E-06	3.117247	down	down	326624	RAB37	
CB_010948	4.92E-05	2.03E-06	2.12368	down	down	2054	STX2	
CB_013014	4.87E-05	1.99E-06	2.387821	down	down	2289	FKBP5	
CB_022520	4.86E-05	1.99E-06	3.988267	down	down	80014	WWC2	
CB_017857	4.81E-05	1.96E-06	3.50148	down	down	23250	ATP11A	
CB_029189	4.78E-05	1.93E-06	3.871281	down	down	255189	PLA2G4F	
CB_027674	4.73E-05	1.91E-06	2.861911	down	down	340419	RSPO2	
CB_005528	4.73E-05	1.9E-06	8.606607	down	down	477	ATP1A2	
CB_014129	4.72E-05	1.9E-06	3.122663	down	down	2674	GFRA1	
CB_010256	4.71E-05	1.89E-06	2.135866	down	down	902	CCNH	
CB_020207	4.61E-05	1.84E-06	3.65711	down	down	54438	GFOD1	
CB_015229	4.61E-05	1.84E-06	2.458819	down	down	10491	CRTAP	
CB_027275	4.58E-05	1.81E-06	3.557903	down	down	284904	SEC14L4	
CB_017550	4.58E-05	1.81E-06	2.068487	down	down	22841	RAB11FIP2	
CB_005768	4.46E-05	1.75E-06	2.154063	down	down	5638	PRRG1	
CB_015017	4.44E-05	1.73E-06	5.135121	down	down	4929	NR4A2	
CB_021102	4.41E-05	1.72E-06	4.342888	down	down	8470	SORBS2	
CB_011688	4.32E-05	1.68E-06	6.973903	down	down	5549	PRELP	
CB_019940	4.29E-05	1.66E-06	4.810152	down	down	55805	LRP2BP	
CB_016611	4.28E-05	1.66E-06	2.01393	down	down	29904	EEF2K	
CB_004890	4.27E-05	1.65E-06	5.393926	down	down	590	BCHE	
CB_012504	4.25E-05	1.64E-06	5.411422	down	down	8399	PLA2G10	
CB_026614	4.23E-05	1.63E-06	3.826057	down	down	135932	TMEM139	
CB_009778	4.16E-05	1.59E-06	14.25653	down	down	5225	PGC	
CB_016990	4.04E-05	1.52E-06	3.599701	down	down	23604	DAPK2	
CB_015006	4.01E-05	1.5E-06	5.691443	down	down	4900	NRGN	
CB_023154	3.96E-05	1.47E-06	3.481689	down	down	56134	PCDHAC2	
CB_009081	3.94E-05	1.46E-06	2.584935	down	down	10979	FERMT2	
CB_024227	3.91E-05	1.44E-06	5.498372	down	down	90865	IL33	
CB_020856	3.89E-05	1.43E-06	2.50308	down	down	57536	KIAA1328	
CB_011203	3.82E-05	1.39E-06	2.473724	down	down	3708	ITPR1	
CB_019838	3.76E-05	1.36E-06	2.53035	down	down	55282	LRRC36	
CB_018626	3.64E-05	1.3E-06	2.928117	down	down	51421	AMOTL2	
CB_001079	3.63E-05	1.3E-06	4.209692	down	down	10865	ARID5A	
CB_024015	3.59E-05	1.28E-06	2.46258	down	down	89846	FGD3	
CB_006160	3.55E-05	1.26E-06	2.138415	down	down	343413	FCRL6	
CB_008810	3.55E-05	1.25E-06	2.358882	down	down	65065	NBEAL1	
CB_028916	3.48E-05	1.22E-06	31.21256	down	down	389376	SFTA2	
CB_016380	3.47E-05	1.22E-06	3.026973	down	down	23266	LPHN2	
CB_013550	3.39E-05	1.18E-06	3.276084	down	down	9068	ANGPTL1	
CB_006062	3.3E-05	1.14E-06	6.541246	down	down	285016	FAM150B	
CB_021593	3.28E-05	1.13E-06	3.333502	down	down	152007	GLIPR2	
CB_023940	3.28E-05	1.13E-06	2.208456	down	down	9117	SEC22C	
CB_024267	3.27E-05	1.12E-06	3.133649	down	down	90273	CEACAM21	
CB_014524	3.24E-05	1.11E-06	4.343736	down	down	7113	TMPRSS2	
CB_005177	3.21E-05	1.09E-06	2.579429	down	down	6103	RPGR	
CB_007294	3.21E-05	1.1E-06	3.050821	down	down	10659	CELF2	
CB_025838	3.21E-05	1.09E-06	2.76783	down	down	23336	SYNM	

CB_002434	3.17E-05	1.07E-06	2.135432	down	down	3841	KPNA5	
CB_006132	3.09E-05	1.03E-06	4.194138	down	down	11217	AKAP2	
CB_005753	2.88E-05	9.36E-07	3.87525	down	down	5446	PON3	
CB_012495	2.83E-05	9.17E-07	3.617334	down	down	8382	NME5	
CB_011403	2.76E-05	8.87E-07	2.151345	down	down	4327	MMP19	
CB_026286	2.72E-05	8.68E-07	4.458059	down	down	166929	SGMS2	
CB_017408	2.57E-05	8.02E-07	2.008372	down	down	9812	KIAA0141	
CB_014851	2.57E-05	8.03E-07	2.1237	down	down	7763	ZFAND5	
CB_027466	2.56E-05	7.99E-07	5.810962	down	down	93035	PKHD1L1	
CB_014263	2.56E-05	7.98E-07	2.806256	down	down	6414	SEPP1	
CB_005409	2.51E-05	7.81E-07	4.172857	down	down	7306	TYRP1	
CB_013569	2.48E-05	7.66E-07	2.839133	down	down	9122	SLC16A4	
CB_023457	2.46E-05	7.61E-07	2.070273	down	down	84263	HSDL2	
CB_012656	2.46E-05	7.6E-07	8.085919	down	down	8630	HSD17B6	
CB_011012	2.45E-05	7.55E-07	9.295558	down	down	2532	DARC	
CB_013108	2.43E-05	7.42E-07	2.061293	down	down	9265	CYTH3	
CB_012218	2.42E-05	7.36E-07	2.482885	down	down	7082	TJP1	
CB_005593	2.37E-05	7.19E-07	23.90216	down	down	1580	CYP4B1	
CB_005492	2.35E-05	7.11E-07	30.62061	down	down	124	ADH1A	
CB_027180	2.28E-05	6.79E-07	14.80696	down	down	286133	SCARA5	
CB_023222	2.28E-05	6.76E-07	3.139593	down	down	83891	SNX25	
CB_015618	2.26E-05	6.7E-07	8.80286	down	down	8685	MARCO	
CB_009347	2.23E-05	6.55E-07	2.991234	down	down	51562	MBIP	
CB_013816	2.22E-05	6.52E-07	2.290057	down	down	1612	DAPK1	
CB_022298	2.22E-05	6.52E-07	2.872832	down	down	474344	GIMAP6	
CB_016899	2.22E-05	6.5E-07	2.24164	down	down	6844	VAMP2	
CB_011575	2.16E-05	6.22E-07	3.316071	down	down	5138	PDE2A	
CB_021310	2.14E-05	6.15E-07	5.539055	down	down	59350	RXFP1	
CB_015682	2.09E-05	5.97E-07	9.676144	down	down	10974	C10orf116	
CB_019972	2.09E-05	5.95E-07	2.5029	down	down	55824	PAG1	
CB_018666	2.09E-05	5.94E-07	2.863402	down	down	51170	HSD17B11	
CB_025459	2.06E-05	5.84E-07	2.021475	down	down	132160	PPM1M	
CB_005545	2.04E-05	5.76E-07	4.320699	down	down	776	CACNA1D	
CB_017425	2.04E-05	5.75E-07	2.617556	down	down	9839	ZEB2	
CB_009819	1.99E-05	5.55E-07	2.006439	down	down	253725	FAM21C	
CB_011827	1.93E-05	5.36E-07	2.016312	down	down	5829	PXN	
CB_015236	1.9E-05	5.23E-07	3.885817	down	down	10497	UNC13B	
CB_014785	1.83E-05	4.95E-07	3.255255	down	down	4337	MOCS1	
CB_017525	1.83E-05	4.93E-07	3.360527	down	down	9936	CD302	
CB_015590	1.82E-05	4.88E-07	2.584333	down	down	11214	AKAP13	
CB_008803	1.73E-05	4.57E-07	3.484258	down	down	53405	CLIC5	
CB_010093	1.73E-05	4.56E-07	2.554108	down	down	5269	SERPINB6	
CB_011280	1.72E-05	4.54E-07	2.401167	down	down	3913	LAMB2	
CB_007077	1.71E-05	4.48E-07	2.327457	down	down	4921	DDR2	
CB_023398	1.7E-05	4.45E-07	3.056779	down	down	84182	FAM188B	
CB_019598	1.63E-05	4.19E-07	4.991955	down	down	55118	CRTAC1	
CB_021106	1.63E-05	4.15E-07	4.420418	down	down	420	ART4	
CB_016549	1.62E-05	4.13E-07	2.820096	down	down	29103	DNAJC15	
CB_021236	1.6E-05	4.04E-07	4.113178	down	down	58484	NLRC4	
CB_011860	1.57E-05	3.95E-07	13.83773	down	down	5923	RASGRF1	
CB_010922	1.57E-05	3.91E-07	2.420379	down	down	1909	EDNRA	
CB_007575	1.48E-05	3.62E-07	4.812591	down	down	10580	SORBS1	
CB_018800	1.48E-05	3.6E-07	17.48281	down	down	1361	CPB2	
CB_018064	1.47E-05	3.56E-07	5.23356	down	down	25890	ABI3BP	
CB_029621	1.47E-05	3.55E-07	3.2361	down	down	80022	MYO15B	
CB_019064	1.46E-05	3.52E-07	2.455466	down	down	54103	PION	

CB_013284	1.45E-05	3.49E-07	2.361994	down	down	1787	TRDMT1	
CB_024115	1.45E-05	3.47E-07	3.058325	down	down	9830	TRIM14	
CB_012978	1.44E-05	3.46E-07	2.294664	down	down	1465	CSRP1	
CB_013433	1.44E-05	3.43E-07	3.543178	down	down	4855	NOTCH4	
CB_014687	1.42E-05	3.38E-07	2.739658	down	down	10253	SPRY2	
CB_015333	1.41E-05	3.34E-07	3.835047	down	down	10628	TXNIP	
CB_006462	1.4E-05	3.29E-07	14.22651	down	down	9254	CACNA2D2	
CB_027696	1.38E-05	3.23E-07	3.304106	down	down	154865	IQUB	
CB_017083	1.37E-05	3.2E-07	5.474274	down	down	27129	HSPB7	
CB_022502	1.34E-05	3.12E-07	2.81926	down	down	79991	OBFC1	
CB_022918	1.34E-05	3.11E-07	3.739912	down	down	81617	CAB39L	
CB_027465	1.34E-05	3.09E-07	2.705868	down	down	6799	SULT1A2	
CB_023980	1.33E-05	3.07E-07	2.548069	down	down	23499	MACF1	
CB_022947	1.31E-05	3.01E-07	4.875724	down	down	221692	PHACTR1	
CB_006813	1.31E-05	2.99E-07	4.014994	down	down	3082	HGF	
CB_022361	1.3E-05	2.96E-07	2.623857	down	down	79834	PEAK1	
CB_005424	1.3E-05	2.96E-07	3.009189	down	down	1604	CD55	
CB_025164	1.3E-05	2.96E-07	7.5485	down	down	5468	PPARG	
CB_008885	1.28E-05	2.89E-07	2.132064	down	down	8837	CFLAR	
CB_023257	1.25E-05	2.79E-07	2.177741	down	down	83938	C10orf11	
CB_012989	1.25E-05	2.79E-07	2.53949	down	down	1831	TSC22D3	
CB_015810	1.25E-05	2.79E-07	6.257114	down	down	9510	ADAMTS1	
CB_011075	1.23E-05	2.73E-07	3.385005	down	down	2995	GYPC	
CB_022851	1.23E-05	2.72E-07	2.449746	down	down	81553	FAM49A	
CB_013503	1.23E-05	2.72E-07	10.63156	down	down	7433	VIPR1	
CB_012880	1.19E-05	2.62E-07	5.258379	down	down	8999	CDKL2	
CB_011806	1.19E-05	2.6E-07	3.937572	down	down	5793	PTPRG	
CB_006078	1.11E-05	2.37E-07	6.777638	down	down	3953	LEPR	
CB_015953	1.08E-05	2.28E-07	2.516064	down	down	7940	LST1	
CB_014939	1.07E-05	2.27E-07	2.110504	down	down	10392	NOD1	
CB_013082	1.07E-05	2.25E-07	2.548678	down	down	8814	CDKL1	
CB_026993	1.07E-05	2.25E-07	6.099917	down	down	147463	ANKRD29	
CB_022874	1.06E-05	2.22E-07	4.552894	down	down	81575	APOLD1	
CB_008287	1.04E-05	2.16E-07	4.252529	down	down	375033	PEAR1	
CB_005673	1.04E-05	2.15E-07	3.750303	down	down	2977	GUCY1A2	
CB_017317	9.53E-06	1.9E-07	3.773284	down	down	9721	GPRIN2	
CB_013054	9.46E-06	1.88E-07	4.259906	down	down	6236	RRAD	
CB_005151	9.39E-06	1.85E-07	3.518816	down	down	5376	PMP22	
CB_027934	9.39E-06	1.85E-07	3.604955	down	down	126668	TDRD10	
CB_010968	9.39E-06	1.85E-07	7.402225	down	down	2205	FCER1A	
CB_007313	9.11E-06	1.77E-07	2.677473	down	down	7106	TSPAN4	
CB_027765	9.08E-06	1.76E-07	3.212952	down	down	3672	ITGA1	
CB_015963	8.99E-06	1.73E-07	8.095231	down	down	10351	ABCA8	
CB_016095	8.81E-06	1.69E-07	2.119937	down	down	1727	CYB5R3	
CB_009001	8.62E-06	1.64E-07	2.166193	down	down	6709	SPTAN1	
CB_013328	8.61E-06	1.64E-07	3.986402	down	down	2078	ERG	
CB_024774	8.56E-06	1.62E-07	6.524446	down	down	142683	ITLN2	
CB_005891	8.48E-06	1.6E-07	7.216861	down	down	948	CD36	
CB_022550	8.39E-06	1.58E-07	2.410118	down	down	54507	ADAMTSL4	
CB_010396	8.32E-06	1.56E-07	3.908981	down	down	1808	DPYSL2	
CB_018098	8.26E-06	1.53E-07	2.727211	down	down	25927	CNRIP1	
CB_024069	8.05E-06	1.48E-07	2.66427	down	down	8692	HYAL2	
CB_008876	8E-06	1.46E-07	2.767216	down	down	141	ADPRH	
CB_026449	7.66E-06	1.37E-07	6.569825	down	down	9104	RGN	
CB_026740	7.58E-06	1.33E-07	3.764988	down	down	10235	RASGRP2	
CB_021592	7.52E-06	1.32E-07	2.772834	down	down	64147	KIF9	

CB_017899	7.41E-06	1.29E-07	2.523935	down	down	23303	KIF13B	
CB_012651	7.29E-06	1.27E-07	3.735945	down	down	8622	PDE8B	
CB_016217	7.29E-06	1.27E-07	4.103554	down	down	25802	LMOD1	
CB_008545	7.18E-06	1.24E-07	14.07671	down	down	219995	MS4A15	
CB_018657	7.15E-06	1.22E-07	9.007642	down	down	9173	IL1RL1	
CB_005322	7.05E-06	1.2E-07	3.139169	down	down	203	AK1	
CB_020966	6.99E-06	1.18E-07	2.635565	down	down	57658	CALCOCO1	
CB_009666	6.98E-06	1.18E-07	2.300729	down	down	57091	CASS4	
CB_024198	6.9E-06	1.16E-07	2.640384	down	down	85450	ITPRIP	
CB_013072	6.29E-06	1.02E-07	2.514993	down	down	7439	BEST1	
CB_020255	6.21E-06	9.95E-08	3.950143	down	down	54511	HMGCLL1	
CB_018900	6.18E-06	9.88E-08	3.228977	down	down	4815	NINJ2	
CB_010558	6.03E-06	9.53E-08	3.737615	down	down	3491	CYR61	
CB_007199	6E-06	9.44E-08	14.37044	down	down	145781	GCOM1	
CB_016002	5.88E-06	9.18E-08	2.653148	down	down	11228	RASSF8	
CB_020295	5.87E-06	9.15E-08	2.542489	down	down	54558	SPATA6	
CB_010887	5.75E-06	8.91E-08	3.886971	down	down	1528	CYB5A	
CB_020835	5.66E-06	8.73E-08	2.368364	down	down	57515	SERINC1	
CB_008512	5.54E-06	8.4E-08	7.792451	down	down	221395	GPR116	
CB_010454	5.5E-06	8.28E-08	26.13088	down	down	2167	FABP4	
CB_012646	5.42E-06	8.12E-08	2.513053	down	down	8613	PPAP2B	
CB_019203	5.06E-06	7.4E-08	3.790342	down	down	54796	BNC2	
CB_011477	5.05E-06	7.39E-08	2.468942	down	down	4784	NFIX	
CB_011597	5.05E-06	7.37E-08	2.69864	down	down	5199	CFP	
CB_010316	5.02E-06	7.32E-08	3.472964	down	down	1316	KLF6	
CB_025336	5.01E-06	7.28E-08	3.552766	down	down	121512	FGD4	
CB_005372	4.75E-06	6.68E-08	9.039619	down	down	3045	HBD	
CB_017404	4.65E-06	6.46E-08	6.229549	down	down	9806	SPOCK2	
CB_018283	4.64E-06	6.43E-08	5.025279	down	down	27151	CPAMD8	
CB_028015	4.6E-06	6.35E-08	2.315884	down	down	338699	ANKRD42	
CB_019283	4.54E-06	6.23E-08	2.038106	down	down	54861	SNRK	
CB_013668	4.49E-06	6.12E-08	4.975304	down	down	9353	SLIT2	
CB_015612	4.48E-06	6.1E-08	3.987357	down	down	7832	BTG2	
CB_021166	4.24E-06	5.6E-08	3.598068	down	down	6196	RPS6KA2	
CB_013006	4.23E-06	5.57E-08	2.842146	down	down	2217	FCGRT	
CB_008782	3.93E-06	5.01E-08	4.335761	down	down	104	ADARB1	
CB_026531	3.88E-06	4.91E-08	4.322291	down	down	120425	AMICA1	
CB_018924	3.7E-06	4.62E-08	4.68411	down	down	51285	RASL12	
CB_012876	3.47E-06	4.22E-08	9.815984	down	down	8991	SELENBP1	
CB_017794	3.4E-06	4.02E-08	3.845428	down	down	23171	GPD1L	
CB_010443	3.38E-06	3.99E-08	4.730678	down	down	2034	EPAS1	
CB_026780	3.35E-06	3.89E-08	5.92724	down	down	8854	ALDH1A2	
CB_027693	3.35E-06	3.89E-08	7.52458	down	down	285313	IGSF10	
CB_013291	3.28E-06	3.77E-08	5.528466	down	down	1843	DUSP1	
CB_009536	3.16E-06	3.54E-08	6.141143	down	down	284	ANGPT1	
CB_022074	3.03E-06	3.3E-08	2.935874	down	down	11000	SLC27A3	
CB_002360	2.98E-06	3.2E-08	6.509631	down	down	79836	LONRF3	
CB_012939	2.84E-06	2.97E-08	4.918778	down	down	408	ARRB1	
CB_015740	2.8E-06	2.93E-08	2.147875	down	down	4649	MYO9A	
CB_021149	2.75E-06	2.85E-08	2.732379	down	down	1408	CRY2	
CB_027773	2.73E-06	2.81E-08	2.619693	down	down	5295	PIK3R1	
CB_005350	2.51E-06	2.48E-08	4.791009	down	down	2006	ELN	
CB_014777	2.44E-06	2.37E-08	2.468373	down	down	4299	AFF1	
CB_008203	2.31E-06	2.16E-08	3.583483	down	down	9625	AATK	
CB_027531	2.31E-06	2.18E-08	4.491983	down	down	23037	PDZD2	
CB_004845	2.31E-06	2.19E-08	4.900964	down	down	94	ACVRL1	

CB_028226	2.29E-06	2.12E-08	2.220871	down	down	158135	TTLL11	
CB_025343	2.24E-06	2.06E-08	2.190454	down	down	196883	ADCY4	
CB_004879	2.22E-06	2.02E-08	9.173018	down	down	443	ASPA	
CB_023538	2.19E-06	1.97E-08	4.579329	down	down	10826	C5orf4	
CB_025248	2.18E-06	1.97E-08	3.065068	down	down	90952	ESAM	
CB_009587	2.18E-06	1.95E-08	5.101269	down	down	316	AOX1	
CB_014694	2.13E-06	1.9E-08	5.182317	down	down	10266	RAMP2	
CB_019495	2.09E-06	1.85E-08	4.490022	down	down	55022	PID1	
CB_017501	2.09E-06	1.84E-08	5.070392	down	down	9912	ARHGAP44	
CB_016118	2.08E-06	1.81E-08	9.689855	down	down	22915	MMRN1	
CB_027887	2.03E-06	1.75E-08	2.548747	down	down	11142	PKIG	
CB_022353	1.77E-06	1.41E-08	4.52785	down	down	79825	CCDC48	
CB_012161	1.65E-06	1.24E-08	6.112341	down	down	6886	TAL1	
CB_015625	1.64E-06	1.23E-08	2.199458	down	down	9444	QKI	
CB_019295	1.63E-06	1.21E-08	5.23592	down	down	54873	PALMD	
CB_012045	1.45E-06	1.04E-08	5.640926	down	down	6586	SLIT3	
CB_027909	1.45E-06	1.03E-08	10.28654	down	down	57716	PRX	
CB_023936	1.38E-06	9.53E-09	11.24598	down	down	6358	CCL14	
CB_017644	1.38E-06	9.54E-09	11.62119	down	down	22998	LIMCH1	
CB_027771	1.37E-06	9.31E-09	3.909781	down	down	83849	SYT15	
CB_027038	1.37E-06	9.27E-09	4.836863	down	down	219621	C10orf107	
CB_013546	1.36E-06	9.16E-09	2.99165	down	down	9022	CLIC3	
CB_008233	1.31E-06	8.6E-09	3.348086	down	down	85358	SHANK3	
CB_014807	1.27E-06	8.14E-09	3.623543	down	down	4628	MYH10	
CB_009136	1.27E-06	8.04E-09	25.18664	down	down	177	AGER	
CB_005411	1.26E-06	7.89E-09	5.707852	down	down	7450	VWF	
CB_018999	1.23E-06	7.5E-09	8.808499	down	down	2348	FOLR1	
CB_026631	1.22E-06	7.43E-09	5.732232	down	down	79987	SVEP1	
CB_006893	1.21E-06	7.15E-09	2.088144	down	down	64400	AKTIP	
CB_020323	1.21E-06	7.23E-09	3.601474	down	down	7148	TNXB	
CB_017988	1.16E-06	6.54E-09	3.448579	down	down	23500	DAAM2	
CB_005724	1.15E-06	6.43E-09	3.422404	down	down	4881	NPR1	
CB_024414	1.13E-06	6.19E-09	4.378386	down	down	115677	NOSTRIN	
CB_021108	1.08E-06	5.54E-09	12.06535	down	down	653	BMP5	
CB_013696	1.07E-06	5.48E-09	4.966921	down	down	9411	ARHGAP29	
CB_028294	9.68E-07	4.64E-09	2.370802	down	down	64236	PDLIM2	
CB_022429	9.22E-07	4.27E-09	4.006277	down	down	79901	CYBRD1	
CB_029199	9.14E-07	4.19E-09	7.275643	down	down	387700	SLC16A12	
CB_022206	9.06E-07	4.14E-09	8.508825	down	down	79674	VEPH1	
CB_011934	9.05E-07	4.1E-09	3.363643	down	down	6275	S100A4	
CB_010408	8.57E-07	3.79E-09	5.739498	down	down	1901	S1PR1	
CB_014847	7.55E-07	3.15E-09	3.375029	down	down	7466	WFS1	
CB_013239	7.52E-07	3.08E-09	2.422852	down	down	977	CD151	
CB_005167	7.43E-07	2.99E-09	6.307633	down	down	5745	PTH1R	
CB_018123	6.78E-07	2.59E-09	4.822264	down	down	25959	KANK2	
CB_026223	6.65E-07	2.47E-09	8.116093	down	down	153579	BTNL9	
CB_018664	6.37E-07	2.21E-09	7.479603	down	down	51705	EMCN	
CB_023207	5.99E-07	1.94E-09	4.049558	down	down	83878	USHBP1	
CB_016264	5.96E-07	1.84E-09	5.627266	down	down	8322	FZD4	
CB_013722	5.89E-07	1.75E-09	2.691865	down	down	9467	SH3BP5	
CB_016727	5.5E-07	1.52E-09	2.823747	down	down	30815	ST6GALNAC6	
CB_004895	5.31E-07	1.4E-09	2.621667	down	down	686	BTD	
CB_017961	4.74E-07	1.19E-09	3.298957	down	down	23371	TENC1	
CB_022339	4.74E-07	1.18E-09	4.092517	down	down	79812	MMRN2	
CB_009049	4.7E-07	1.12E-09	2.427351	down	down	9922	IQSEC1	
CB_026759	4.7E-07	1.1E-09	4.903791	down	down	10044	SH2D3C	

CB_022168	4.61E-07	1.05E-09	5.078008	down	down	79633	FAT4	
CB_025803	4.41E-07	9.78E-10	2.578784	down	down	8578	SCARF1	
CB_019827	4.35E-07	9.51E-10	16.24182	down	down	55273	TMEM100	
CB_015856	4.06E-07	8.19E-10	24.83615	down	down	11095	ADAMTS8	
CB_011904	3.7E-07	6.98E-10	4.355655	down	down	6038	RNASE4	
CB_005314	2.06E-07	2.54E-10	6.224293	down	down	7010	TEK	
CB_012177	1.88E-07	2.24E-10	18.25979	down	down	6943	TCF21	
CB_020750	1.7E-07	1.88E-10	4.666706	down	down	57381	RHOJ	
CB_017134	1.36E-07	1.31E-10	3.864007	down	down	27303	RBMS3	
CB_017331	9.59E-08	7.54E-11	2.576534	down	down	9732	DOCK4	
CB_016715	9.59E-08	7.47E-11	8.388946	down	down	395	ARHGAP6	
CB_014949	8.85E-08	6.56E-11	6.210699	down	down	10411	RAPGEF3	
CB_015858	7.96E-08	5.68E-11	6.08879	down	down	11099	PTPN21	
CB_014278	7.89E-08	5.11E-11	5.418807	down	down	7075	TIE1	
CB_010992	7.41E-08	4.46E-11	4.302376	down	down	2324	FLT4	
CB_010777	7.41E-08	4.4E-11	4.819443	down	down	1003	CDH5	
CB_010307	6.83E-08	3.36E-11	4.00155	down	down	9079	LDB2	
CB_024893	6.22E-08	2.08E-11	11.31436	down	down	147372	CCBE1	
CB_015975	6.22E-08	1.92E-11	15.61282	down	down	11170	FAM107A	
CB_013914	5.46E-08	1.38E-11	8.291745	down	down	5348	FXYD1	
CB_011813	3.91E-08	5.54E-12	5.273172	down	down	5797	PTPRM	
CB_023970	2.51E-08	1.19E-12	8.580241	down	down	64651	CSRNP1	
CB_022842	0.019341	0.007511	2.831615	down	down	81501	TM7SF4	
CB_030363	0.014492	0.005121	2.028972	down	down	200058	FLJ23867	
CB_022771	0.013232	0.004533	2.467392	down	down	283987	C17orf28	
CB_016860	0.002965	0.00057	2.048134	down	down	29094	HSPC159	
CB_029345	0.00241	0.000427	2.156177	down	down	8418	CMAH	
CB_000693	0.002096	0.000353	3.357297	down	down	401147	FLJ43963	
CB_025642	0.000899	0.000109	2.499752	down	down	201134	CCDC46	
CB_006101	0.00059	6.16E-05	2.910301	down	down	445347	TARP	
CB_029075	0.000261	2.03E-05	3.319777	down	down	343990	C2orf55	
CB_006827	0.000196	1.39E-05	3.060278	down	down	400746	C1orf130	
CB_024934	0.000179	1.22E-05	2.155923	down	down	51776	ZAK	
CB_019886	0.000171	1.15E-05	3.230889	down	down	55321	C20orf46	
CB_022269	0.000166	1.11E-05	2.056252	down	down	28971	C11orf67	
CB_022346	0.000113	6.71E-06	2.317448	down	down	79817	MOBKL2B	
CB_023555	8.24E-05	4.32E-06	2.754185	down	down	84418	C5orf32	
CB_019287	5.49E-05	2.39E-06	2.112981	down	down	54863	C9orf167	
CB_016026	4.17E-05	1.6E-06	2.033759	down	down	11261	CHP	
CB_013454	2.69E-05	8.58E-07	3.471384	down	down	5414	4-Sep	
CB_023284	2.54E-05	7.89E-07	2.704002	down	down	9708	PCDHGA8	
CB_030434	2.45E-05	7.52E-07	5.049114	down	down	339524	LOC339524	
CB_017090	1.93E-05	5.36E-07	2.395627	down	down	27143	KIAA1274	
CB_007460	1.57E-05	3.95E-07	3.179302	down	down	196740	C10orf72	
CB_022159	6.72E-06	1.11E-07	9.181296	down	down	79625	C4orf31	
CB_016804	4.33E-06	5.77E-08	5.711881	down	down	28984	C13orf15	
CB_022487	3.12E-06	3.48E-08	3.84815	down	down	79974	C7orf58	
CB_028190	1.39E-06	9.8E-09	2.419627	down	down	221749	C6orf145	
CB_006558	1.16E-06	6.54E-09	2.183452	down	down	492311	C5orf53	
CB_023244	9.88E-07	4.85E-09	2.37526	down	down	23731	C9orf5	
CB_008538	4.06E-07	8.24E-10	3.574796	down	down	23176	8-Sep	

Table S5B The mRNA probes expressed with different direction between our study and TCGA dataset								
ProbeName	p (Corr)	p	FC (abs)	Regulation	TCGA regulation	Entrez GeneID	Gene Symbol	
CB_008838	0.00827	0.002381	2.433272	up	down	5825	ABCD3	
CB_017021	0.036604	0.017235	2.066424	up	down	26253	CLEC4E	
CB_009292	0.008205	0.002356	2.964326	up	down	80313	LRRC27	
CB_022175	0.006666	0.001758	2.815598	up	down	79639	TMEM53	
CB_019247	0.003915	0.000839	2.049401	up	down	54826	GIN1	
CB_008568	0.046648	0.023401	2.621854	up	down	728118	FAM22A	
CB_022737	0.031855	0.014408	2.772724	up	down	80741	LY6G5C	
CB_017764	0.018118	0.006894	2.219839	up	down	23132	RAD54L2	
CB_000635	0.014896	0.00531	2.590187	up	down	9905	SGSM2	
CB_000890	0.011713	0.003832	2.236412	up	down	2185	PTK2B	
CB_001159	0.007817	0.002202	2.747563	up	down	11158	RABL2B	
CB_006822	0.005693	0.001409	2.003814	up	down	55653	BCAS4	
CB_026987	0.004986	0.001174	2.613292	up	down	143279	HECTD2	
CB_030039	0.00444	0.000998	2.948961	up	down	648740	LOC648740	
CB_005647	0.042629	0.0209	2.086622	down	up	2903	GRIN2A	
CB_025920	0.013934	0.004863	2.156941	down	up	56477	CCL28	
CB_018727	0.01132	0.00366	2.295528	down	up	51454	GULP1	
CB_013541	0.011004	0.003524	2.137897	down	up	8876	VNN1	
CB_025049	0.010875	0.003469	2.885811	down	up	112703	FAM71E1	
CB_010283	0.001508	0.000223	7.317299	down	up	1066	CES1	
CB_012531	0.000589	6.14E-05	2.202423	down	up	8452	CUL3	
CB_006428	0.000364	3.18E-05	2.770407	down	up	23114	NFASC	
CB_008202	0.000101	5.76E-06	3.042011	down	up	23514	KIAA0146	
CB_005494	4.95E-05	2.05E-06	20.60552	down	up	126	ADH1C	
CB_010935	3.21E-05	1.09E-06	2.836412	down	up	1983	EIF5	
CB_010377	0.00574	0.001426	2.944169	down	up	1767	DNAH5	
CB_012561	0.000905	0.00011	2.064548	down	up	8496	PPFIBP1	
CB_017971	5.86E-05	2.64E-06	4.072144	down	up	23382	AHCYL2	
CB_012106	2.63E-06	2.67E-08	2.343051	down	up	6717	SRI	
CB_024290	0.023412	0.009675	2.504265	down	up	79937	CNTNAP3	
CB_025409	0.017889	0.006781	2.75999	down	up	116372	LYPD1	
CB_014792	0.009747	0.002987	2.03736	down	up	4496	MT1H	
CB_016084	0.006698	0.001769	2.319213	down	up	6622	SNCA	
CB_010401	0.006461	0.001684	2.54812	down	up	1837	DTNA	
CB_025610	0.004816	0.001117	2.296857	down	up	203102	ADAM32	
CB_010862	0.004692	0.001077	3.057998	down	up	1410	CRYAB	
CB_022282	0.0046	0.001048	2.124839	down	up	79745	CLIP4	
CB_021225	0.003294	0.000661	2.042276	down	up	57835	SLC4A5	
CB_028293	0.00316	0.000623	2.271387	down	up	11162	NUDT6	
CB_009558	0.002049	0.000342	2.229869	down	up	199990	C1orf86	
CB_017867	0.001393	0.000199	2.282842	down	up	23261	CAMTA1	
CB_028108	0.001104	0.000145	2.653295	down	up	9586	CREB5	
CB_011282	0.000916	0.000112	2.256248	down	up	3920	LAMP2	
CB_007947	0.000546	5.53E-05	2.181589	down	up	831	CAST	
CB_018163	0.000454	4.31E-05	2.231065	down	up	667	DST	
CB_027173	0.000224	1.65E-05	2.212298	down	up	285521	COX18	
CB_007712	8.11E-05	4.23E-06	2.366886	down	up	55423	SIRPG	
CB_029131	0.00198	0.000325	2.006721	down	up	400223	C14orf181	

Table S6A The lncRNA probes expressed with same direction between our study and TCGA dataset								
ProbeName	p (Corr)	p	FC (abs)	Regulati	TCGA regulation	Transcripts Ensemble_68	Biotype-Transcripts Ensemble_68	Name Ensemble_68
RNA176927|ENST00000423121	0.011445	0.00371	2.1073	up	up	ENST00000423121	antisense	RP11-466F5.8
RNA175466|ENST00000436286	0.01239	0.00414	2.27238	up	up	ENST00000436286	antisense	RP4-669H2.1
RNA177939|ENST00000420243	0.00012	7.2E-06	5.61033	up	up	ENST00000420243	antisense	POU6F2-AS2
RNA39964|RefSeq_1987_1753	0.005684	0.00141	2.07479	up	up	ENST00000562866	antisense	RP11-368I7.2
RNA46604|UCSC_5107_1965	0.007901	0.00224	2.26414	up	up	ENST00000454234	antisense	RP5-1185I7.1
RNA39340|RefSeq_1312_2380	0.002759	0.00052	2.30312	up	up	ENST00000566699	antisense	RP4-659J6.2
RNA50625|UCSC_9958_819	0.000885	0.00011	2.30836	up	up	ENST00000523330	antisense	RP11-539E17.4
RNA35154|ENCODE_8_5492	0.025144	0.01062	2.35346	up	up	ENST00000438934	antisense	DGCR5
RNA33827|lncRNAdb_34_1628	0.000633	6.8E-05	2.46992	up	up	ENST00000522674	antisense	HOXA11-AS
RNA41061|RefSeq_3170_639	0.000402	3.7E-05	2.62242	up	up	ENST00000565014	antisense	CTD-2574D22.5
RNA55560|H-InvDB_2942_213	0.001204	0.00016	2.68067	up	up	ENST00000439173	antisense	CSAG2
RNA48386|UCSC_7259_1470	0.007621	0.00212	2.99401	up	up	ENST00000430027	antisense	DLX6-AS1
RNA50362|UCSC_9635_907	0.002473	0.00044	3.75973	up	up	ENST00000525867	antisense	AF131216.5
RNA50260|UCSC_9510_937	0.000751	8.6E-05	4.09083	up	up	ENST00000480284	antisense	RP5-1121A15.1
RNA175289|ENST00000507152	0.003358	0.00068	2.09422	up	up	ENST00000507152	lincRNA	RP11-366M4.3
RNA175775|ENST00000551334	0.000348	3E-05	2.33028	up	up	ENST00000551334	lincRNA	CTD-2314B22.3
RNA176183|ENST00000418499	0.000606	6.4E-05	2.88154	up	up	ENST00000418499	lincRNA	AL589743.1
RNA178505|ENST00000515218	0.046628	0.02339	3.01837	up	up	ENST00000515218	lincRNA	RP11-129M6.1
RNA178124|ENST00000457958	3.96E-05	1.5E-06	8.80807	up	up	ENST00000457958	lincRNA	LINC00511
RNA161113|XLOC_012981	0.019206	0.00744	2.04587	up	up	ENST00000397381	lincRNA	UCA1
RNA161288|XLOC_001956	0.003444	0.0007	2.47077	up	up	ENST00000412134	lincRNA	AC022311.1
RNA159487|XLOC_010204	0.000192	1.3E-05	5.0037	up	up	ENST00000547963	lincRNA	RP11-438N16.1
RNA39479|RefSeq_1464_2231	0.004506	0.00102	2.02099	up	up	ENST00000412816	lincRNA	LINC00470
RNA39389|RefSeq_1365_2322	0.009798	0.00301	2.02642	up	up	ENST00000518580	lincRNA	RP11-109J4.1
RNA51387|UCSC_10907_544	0.003012	0.00058	2.04264	up	up	ENST00000564830	lincRNA	RP11-417E7.2
RNA49434|UCSC_8513_1156	0.016069	0.00588	2.07988	up	up	ENST00000295549	lincRNA	AC017048.3
RNA34338|NRED_469_2211	9.77E-05	5.5E-06	2.12103	up	up	ENST00000562231	lincRNA	RP11-314O13.1
RNA44372|UCSC_2317_2957	0.009009	0.00268	2.30783	up	up	ENST00000436078	lincRNA	RP11-91K9.1
RNA49073|UCSC_8082_1260	0.001842	0.00029	2.31032	up	up	ENST00000414416	lincRNA	LINC00116
RNA35643|ENCODE_511_1113	0.02516	0.01063	2.61594	up	up	ENST00000423943	lincRNA	RP11-48O20.4

RNA38362|RefSeq_277_4745	0.020518	0.00813	2.6793	up	up	ENST00000445791	lincRNA	AC074093.1
RNA48055|UCSC_6856_1569	0.005243	0.00126	2.98037	up	up	ENST00000417483	lincRNA	RP11-557H15.3
RNA48920|UCSC_7911_1298	0.000385	3.4E-05	3.01295	up	up	ENST00000411439	lincRNA	AL022344.4
RNA47413|UCSC_6067_1745	0.002986	0.00058	3.1225	up	up	ENST00000428651	lincRNA	AC093375.1
RNA46618|UCSC_5122_1962	0.005578	0.00137	3.12882	up	up	ENST00000500092	lincRNA	RP11-519M16.1
RNA176860|ENST00000428411	0.002283	0.0004	2.00439	up	up	ENST00000428411	processed_transcript	RP11-31F15.1
RNA178210|ENST00000449469	4.78E-05	1.9E-06	2.01252	up	up	ENST00000449469	processed_transcript	RP4-564F22.2
RNA178297|ENST00000422059	0.011892	0.00391	2.10177	up	up	ENST00000422059	processed_transcript	RP5-1120P11.1
RNA178154|ENST00000519104	0.014798	0.00526	2.23398	up	up	ENST00000519104	processed_transcript	RP3-399L15.3
RNA177921|ENST00000423737	0.001227	0.00017	2.273	up	up	ENST00000423737	processed_transcript	RP11-191L9.4
RNA176052|ENST00000500538	0.008295	0.00239	2.28825	up	up	ENST00000500538	processed_transcript	RP11-453E17.1
RNA177413|ENST00000437488	0.00645	0.00168	2.52078	up	up	ENST00000437488	processed_transcript	RP11-385J1.2
RNA176656|ENST00000458151	0.0004	3.6E-05	2.68575	up	up	ENST00000458151	processed_transcript	RP5-1024G6.5
RNA178537|ENST00000521951	0.000375	3.3E-05	2.83275	up	up	ENST00000521951	processed_transcript	PVT1
RNA175036|ENST00000452148	6.05E-05	2.8E-06	3.56189	up	up	ENST00000452148	processed_transcript	RP11-342C23.4
RNA175016|ENST00000508406	9.04E-05	4.9E-06	5.51791	up	up	ENST00000508406	processed_transcript	RP11-8L2.1
RNA177524|ENST00000498731	0.001283	0.00018	6.47473	up	up	ENST00000498731	processed_transcript	SOX2-OT
RNA177061|ENST00000451937	2.09E-05	5.9E-07	8.52219	up	up	ENST00000366437	processed_transcript	MIR205HG
RNA178455|ENST00000393515	0.01651	0.0061	2.03192	up	up	ENST00000393515	processed_transcript	ANKRD36BP2
RNA161747|XLOC_001671	0.000986	0.00012	2.77822	up	up	ENST00000325390	processed_transcript	AC018865.8
RNA50171|UCSC_9396_963	0.008209	0.00236	2.01293	up	up	ENST00000457079	processed_transcript	AC139666.1
RNA46605|UCSC_5108_1965	0.001913	0.00031	2.01551	up	up	ENST00000500447	processed_transcript	MIR210HG
RNA35318|ENCODE_177_2333	0.005684	0.00141	2.03518	up	up	ENST00000439050	processed_transcript	AC009299.3
RNA37495|ENCODE_2366_407	0.00549	0.00134	2.38721	up	up	ENST00000412855	processed_transcript	RP11-396C23.2
RNA48100|UCSC_6908_1558	0.000935	0.00012	2.44248	up	up	ENST00000504230	processed_transcript	CTC-497E21.4
RNA51636|UCSC_11214_442	0.001164	0.00016	3.90575	up	up	ENST00000456627	processed_transcript	LINC00355
RNA47704|UCSC_6437_1662	0.000958	0.00012	2.17959	up	up	ENST00000427391	processed_transcript	RP11-453F18__B.1
RNA47767|UCSC_6512_1645	0.00256	0.00046	2.28853	up	up	ENST00000547804	processed_transcript	RP11-77I22.3
RNA35277|ENCODE_136_2597	0.000944	0.00012	5.63041	up	up	ENST00000431554	processed_transcript	RP11-411K7.1
RNA44606|UCSC_2594_2810	0.00011	6.5E-06	5.92076	up	up	ENST00000518128	processed_transcript	RP11-758M4.4
RNA40573|RefSeq_2638_1143	0.017895	0.00678	2.18056	up	up	ENST00000489520	processed_transcript	RPSAP52
RNA48548|UCSC_7456_1419	8.55E-05	4.6E-06	2.26337	up	up	ENST00000442266	processed_transcript	CCT6P1
RNA40061|RefSeq_2087_1675	0.003193	0.00063	2.45471	up	up	ENST00000435380	processed_transcript	NEURL3
RNA40275|RefSeq_2316_1473	0.025771	0.01096	2.78773	up	up	ENST00000494676	processed_transcript	RPL13AP3
RNA38691|RefSeq_624_3525	0.002021	0.00033	2.88781	up	up	ENST00000496593	processed_transcript	RPLP0P2
RNA39743|RefSeq_1746_1992	6.12E-05	2.8E-06	3.51771	up	up	ENST00000438684	processed_transcript	MSL3P1

RNA38111|RefSeq_11_11312	0.022032	0.00892	4.01913	up	up	ENST00000423298	processed_transcript	RP11-443P15.2
RNA51496|UCSC_11050_498	0.000267	2.1E-05	6.928	up	up	ENST00000556890	processed_transcript	CTD-3006G17.2
RNA176137|ENST00000441069	0.002937	0.00056	2.40482	up	up	ENST00000441069	antisense	RP11-108L7.11
RNA178523|ENST00000523825	0.002682	0.0005	3.76667	up	up	ENST00000523825	antisense	RP11-382A18.1
RNA177363|ENST00000490916	0.002186	0.00037	2.20567	up	up	ENST00000490916	antisense	RP11-204J18.3
RNA160868|XLOC_012342	0.000618	6.6E-05	2.00282	up	up	ENST00000583492	antisense	MAFG-AS1
RNA59103|asoverlaps_775_970	0.000292	2.4E-05	3.26294	up	up	ENST00000414354	antisense	MFI2-AS1
RNA47054|UCSC_5633_1845	9.44E-06	1.9E-07	8.76097	up	up	ENST00000380330	antisense	RP11-116G8.4
RNA177829|ENST00000546117	0.012983	0.00441	2.80853	up	up	ENST00000546117	lincRNA	RP11-173C20.2
RNA175797|ENST00000520619	0.000796	9.3E-05	3.39773	up	up	ENST00000520619	lincRNA	SNHG6
RNA177252|ENST00000433116	0.006784	0.0018	3.427	up	up	ENST00000433116	lincRNA	RP11-637A17.2
RNA178046|ENST00000537925	1.1E-06	5.7E-09	2.05374	up	up	ENST00000537869	lincRNA	SNHG1
RNA176971|ENST00000432536	0.000591	6.2E-05	2.46112	up	up	ENST00000432536	lincRNA	GAS5
RNA36722|ENCODE_1592_546	0.014087	0.00494	2.17139	up	up	ENST00000414401	lincRNA	CECR7
RNA38637|RefSeq_565_3678	0.00462	0.00105	2.2283	up	up	ENST00000509760	lincRNA	RP11-143E21.7
RNA36696|ENCODE_1566_551	1.57E-05	3.9E-07	2.32145	up	up	ENST00000446578	lincRNA	Z83851.3
RNA34694|NRED_826_1613	0.000458	4.4E-05	2.87539	up	up	ENST00000546186	lincRNA	CTD-2335A18.1
RNA49481|UCSC_8570_1142	0.000393	3.5E-05	13.7697	up	up	ENST00000523313	lincRNA	RP11-697M17.1
RNA175889|ENST00000445415	0.004101	0.00089	2.0609	up	up	ENST00000445415	processed_transcript	AC004383.4
RNA175951|ENST00000444958	0.000188	1.3E-05	2.08582	up	up	ENST00000444958	processed_transcript	DANCR
RNA176037|ENST00000445737	0.003256	0.00065	2.1253	up	up	ENST00000445737	processed_transcript	UBAC2-AS1
RNA178450|ENST00000517864	0.000296	2.4E-05	2.26485	up	up	ENST00000517864	processed_transcript	RP11-320N21.1
RNA174235|ENST00000505877	0.012679	0.00427	2.26975	up	up	ENST00000505877	processed_transcript	RP11-308B16.1
RNA178090|ENST00000526611	0.001796	0.00028	2.33668	up	up	ENST00000526611	processed_transcript	RP1-179N16.6
RNA178600|ENST00000453023	0.001027	0.00013	2.38858	up	up	ENST00000453023	processed_transcript	MIAT
RNA174250|ENST00000414438	0.006103	0.00156	3.47235	up	up	ENST00000414438	processed_transcript	AC069277.2
RNA175183|ENST00000456880	0.002352	0.00041	3.50553	up	up	ENST00000456880	processed_transcript	AP001065.2
RNA176105|ENST00000534398	7.25E-05	3.6E-06	4.40055	up	up	ENST00000435097	processed_transcript	RP11-429J17.6
RNA49675|UCSC_8800_1093	0.001613	0.00024	2.08653	up	up	ENST00000455304	processed_transcript	LINC00466
RNA49668|UCSC_8792_1094	0.000732	8.3E-05	2.36888	up	up	ENST00000464958	processed_transcript	RP5-1157M23.2
RNA36250|ENCODE_1120_674	0.003813	0.00081	3.28333	up	up	ENST00000437892	processed_transcript	RP11-124L5.7
RNA37628|ENCODE_2500_381	0.002355	0.00041	2.00799	up	up	ENST00000420819	processed_transcript	RP1-27K12.2
RNA34176|NRED_307_2612	0.000396	3.6E-05	2.11901	up	up	ENST00000437681	processed_transcript	SNHG3
RNA43707|UCSC_1502_3551	0.000462	4.4E-05	3.51678	up	up	ENST00000444312	processed_transcript	ATG9B
RNA51286|UCSC_10777_583	0.00365	0.00076	2.08727	up	up	ENST00000483169	processed_transcript	DLEU1
RNA46249|UCSC_4663_2070	0.032009	0.01449	2.17025	up	up	ENST00000449066	processed_transcript	AC140481.1

RNA39060|RefSeq_1014_2768	0.027822	0.01209	2.53129	up	up	ENST00000575787	processed_transcript	ALOX12P2
RNA39123|RefSeq_1085_2653	0.003359	0.00068	5.4234	up	up	ENST00000428449	antisense	RP11-560I19.4
RNA158247|XLOC_000455	0.000316	2.6E-05	5.01154	up	up	ENST00000420691	lincRNA	RP1-10C16.2
RNA39969|RefSeq_1993_1751	0.003885	0.00083	2.00738	up	up	ENST00000580197	lincRNA	RP11-146G7.3
RNA35280|ENCODE_139_2562	0.013585	0.0047	2.11371	up	up	ENST00000412609	lincRNA	AC093063.1
RNA177566|ENST00000548760	0.006682	0.00176	2.36517	up	up	ENST00000548760	processed_transcript	RP11-181C3.2
RNA178673|ENST00000430666	0.003163	0.00062	2.39282	up	up	ENST00000430666	processed_transcript	AC092937.2
RNA162064|XLOC_013669	0.042974	0.02112	2.43175	up	up	ENST00000412553	processed_transcript	RP5-1068E13.3
RNA35147|ENCODE_1_9124	0.00067	7.3E-05	2.06376	up	up	ENST00000427447	processed_transcript	AP000569.8
RNA49246|UCSC_8284_1209	0.032805	0.01497	2.41758	up	up	ENST00000430694	processed_transcript	IGKJ5
RNA46160|UCSC_4545_2096	0.005664	0.0014	2.01181	up	up	ENST00000455565	processed_transcript	AC126544.2
RNA160294|XLOC_011306	4.75E-05	1.9E-06	2.57754	up	up	ENST00000562965	antisense	RP11-941F15.1
RNA40876|RefSeq_2969_845	0.001135	0.00015	2.0675	up	up	ENST00000382313	antisense	CTD-2270P14.3
RNA40191|RefSeq_2223_1560	0.000146	9.4E-06	5.22318	up	up	ENST00000434346	antisense	RP11-184I16.2
RNA177387|ENST00000526617	0.0046	0.00105	2.92211	up	up	ENST00000526617	processed_transcript	RP11-540A21.3
RNA175489|ENST00000427794	0.000423	3.9E-05	3.04933	up	up	ENST00000427794	processed_transcript	RP5-907D15.2
RNA177484|ENST00000437589	0.005381	0.0013	2.07623	up	up	ENST00000424351	retained_intron	AC017076.4
RNA35572|ENCODE_440_1351	5.41E-05	2.3E-06	2.35376	down	down	ENST00000437232	antisense	RP11-124N14.4
RNA43178|UCSC_866_4298	0.000183	1.3E-05	3.35489	down	down	ENST00000438047	antisense	AC011899.9
RNA174886|ENST00000522193	0.000213	1.5E-05	2.03319	down	down	ENST00000523364	antisense	HOXA-AS2
RNA50695|UCSC_10058_789	0.001352	0.00019	2.41154	down	down	ENST00000549804	antisense	SNHG14
RNA40525|RefSeq_2585_1194	6.78E-07	2.6E-09	2.75274	down	down	ENST00000413221	lincRNA	EPB41L4A-AS1
RNA35355|ENCODE_217_2186	0.000489	4.8E-05	2.42212	down	down	ENST00000421976	lincRNA	AC109642.1
RNA36716|ENCODE_1586_547	0.001024	0.00013	2.02561	down	down	ENST00000424725	lincRNA	RP4-782L23.1
RNA37453|ENCODE_2324_415	5.19E-05	2.2E-06	2.32066	down	down	ENST00000426012	lincRNA	RP1-122P22.2
RNA39266|RefSeq_1234_2469	0.000349	3E-05	2.57356	down	down	ENST00000427111	lincRNA	RP11-118B22.2
RNA38798|RefSeq_739_3273	0.012277	0.00409	2.02128	down	down	ENST00000435944	lincRNA	CASC2
RNA41002|RefSeq_3109_693	4.7E-07	1.1E-09	11.0439	down	down	ENST00000446372	lincRNA	SFTA1P
RNA177347|ENST00000480258	0.000218	1.6E-05	2.05292	down	down	ENST00000480258	lincRNA	DCP1A
RNA45432|UCSC_3636_2366	1.91E-05	5.3E-07	7.04537	down	down	ENST00000499425	lincRNA	RP11-16M8.2
RNA160542|XLOC_011755	0.005374	0.0013	2.74947	down	down	ENST00000499966	lincRNA	CTD-2258A20.4
RNA33799|lncRNAdb_6_8708	0.002021	0.00033	2.24484	down	down	ENST00000534336	lincRNA	MALAT1
RNA33806|lncRNAdb_13_3454	0.007435	0.00205	2.02964	down	down	ENST00000553575	lincRNA	DIO3OS
RNA40182|RefSeq_2214_1569	3.52E-05	1.2E-06	2.68879	down	down	ENST00000318186	processed_transcript	WDFY3-AS2
RNA42917|UCSC_560_4983	1.1E-06	5.8E-09	6.7169	down	down	ENST00000342456	processed_transcript	ALDH3B1
RNA43360|UCSC_1084_3948	0.000309	2.5E-05	2.26916	down	down	ENST00000420823	processed_transcript	AC090044.1

RNA44760|UCSC_2792_2713	0.004424	0.00099	2.07682	down	down	ENST00000441044	processed_transcript	MTMR9LP
RNA40205|RefSeq_2240_1546	1.29E-05	2.9E-07	5.44076	down	down	ENST00000443779	processed_transcript	RP11-327L3.1
RNA46961|UCSC_5521_1870	2.31E-06	2.2E-08	6.70408	down	down	ENST00000446840	processed_transcript	AC003090.1
RNA35681|ENCODE_549_1016	0.045391	0.02262	2.01697	down	down	ENST00000447250	processed_transcript	RP4-625H18.2
RNA38794|RefSeq_735_3278	0.001972	0.00032	3.14006	down	down	ENST00000458468	processed_transcript	LINC00478
RNA176057|ENST00000517335	0.000451	4.3E-05	3.23374	down	down	ENST00000499583	processed_transcript	RP11-175K6.1
RNA175046|ENST00000502083	0.000711	8E-05	2.16245	down	down	ENST00000502083	processed_transcript	RP11-875O11.1
RNA177407|ENST00000503469	0.018786	0.00722	2.03449	down	down	ENST00000503469	processed_transcript	CTD-2003C8.2
RNA176292|ENST00000513055	1.64E-05	4.2E-07	2.70468	down	down	ENST00000504474	processed_transcript	RP11-65F13.2
RNA40958|RefSeq_3056_748	9.48E-05	5.3E-06	2.58879	down	down	ENST00000506741	processed_transcript	RP11-701P16.1
RNA176114|ENST00000508111	0.009894	0.00305	2.36013	down	down	ENST00000508111	processed_transcript	RP11-93L9.1
RNA40416|RefSeq_2471_1330	0.001819	0.00029	3.09191	down	down	ENST00000527780	processed_transcript	NAPSB
RNA41226|RefSeq_3355_426	3.42E-05	1.2E-06	3.26311	down	down	ENST00000565768	processed_transcript	MT1L
RNA43345|UCSC_1064_3978	5.13E-06	7.6E-08	3.39169	down	down	ENST00000452320	retained_intron	MAGI2-AS3
RNA34333|NRED_464_2213	5.76E-05	2.6E-06	2.29934	down	down	ENST00000318291	antisense	RP11-218M22.1
RNA177677|ENST00000413969	0.002188	0.00037	2.78631	down	down	ENST00000413969	antisense	HOXD-AS1
RNA50370|UCSC_9646_902	0.000223	1.6E-05	2.52161	down	down	ENST00000519753	antisense	CTD-3107M8.4
RNA36220|ENCODE_1090_686	0.002328	0.00041	2.72957	down	down	ENST00000421019	lincRNA	RP11-290F20.3
RNA39869|RefSeq_1882_1847	0.000178	1.2E-05	2.07798	down	down	ENST00000571091	lincRNA	MIR22HG
RNA37004|ENCODE_1874_487	9.36E-05	5.2E-06	3.39702	down	down	ENST00000429601	processed_transcript	RP11-368D24__A.1
RNA48896|UCSC_7884_1306	0.009793	0.00301	2.1665	down	down	ENST00000437183	processed_transcript	HLA-DRB6
RNA38622|RefSeq_550_3708	0.005378	0.0013	2.04683	down	down	ENST00000445317	processed_transcript	RP1-163G9.1
RNA40251|RefSeq_2290_1495	0.003438	0.0007	2.42008	down	down	ENST00000454875	processed_transcript	TTTY14
RNA175673|ENST00000486954	0.000589	6.1E-05	2.07437	down	down	ENST00000486954	processed_transcript	RP11-511P7.2
RNA50174|UCSC_9400_961	0.000248	1.9E-05	2.09445	down	down	ENST00000496640	processed_transcript	RP11-81N13.1
RNA176133|ENST00000504984	0.003424	0.0007	2.21949	down	down	ENST00000504984	processed_transcript	RP11-420A23.1
RNA175975|ENST00000519898	0.009816	0.00302	2.10265	down	down	ENST00000519898	processed_transcript	MIR143HG
RNA176082|ENST00000520067	0.045721	0.02283	2.02472	down	down	ENST00000520067	processed_transcript	CTC-308K20.1
RNA178567|ENST00000435523	1.53E-06	1.1E-08	3.06424	down	down	ENST00000579072	lincRNA	AC058791.2
RNA35347|ENCODE_208_2228	5.22E-06	7.7E-08	3.9599	down	down	ENST00000579235	processed_transcript	AC058791.1
RNA35794|ENCODE_663_882	0.002665	0.00049	2.08598	down	down	ENST00000452647	antisense	RP1-199J3.3
RNA40994|RefSeq_3099_702	0.049044	0.02492	2.00011	down	down	ENST00000480632	antisense	RP4-555L14.5
RNA34491|NRED_623_1917	1.7E-05	4.5E-07	3.07111	down	down	ENST00000502125	antisense	RP11-327J17.3
RNA43630|UCSC_1402_3620	1.2E-05	2.6E-07	4.05348	down	down	ENST00000528549	antisense	RP11-385N17.1
RNA34938|NRED_1076_1076	3.42E-05	1.2E-06	5.60282	down	down	ENST00000508269	processed_transcript	RP11-291L15.2
RNA43186|UCSC_876_4275	4.89E-05	2E-06	3.42853	down	down	ENST00000555379	retained_intron	CTD-2552B11.4

RNA178163|ENST00000436602	0.000131	8.2E-06	2.18801	down	down	ENST00000436602	processed_transcript	AC087590.3

Table S6B The lncRNA probes expressed with different direction between our study and TCGA dataset

ProbeName	p (Corr)	p	FC (abs)	Regulati	TCGA regulation	Transcripts Ensemble_68	Biotype-Transcripts Ensemble_68	Name Ensemble_68
RNA176228|ENST00000499143	0.049704	0.02536	3.3318	up	down	ENST00000499143	antisense	RP11-677M14.7
RNA49306|UCSC_8361_1189	0.000362	3.2E-05	4.97104	up	down	ENST00000424208	antisense	SNHG14
RNA34914|NRED_1048_1149	0.003605	0.00075	2.44922	up	down	ENST00000490441	processed_transcript	RP11-446H18.3
RNA34445|NRED_577_1999	4.9E-05	2E-06	2.05821	down	up	ENST00000505731	processed_transcript	NOP14-AS1
RNA178035|ENST00000532353	0.000428	4E-05	2.22087	down	up	ENST00000532353	processed_transcript	TRAF3IP2-AS1
